# Long-Term Outcomes of Left Bundle-Branch Pacing vs Biventricular Pacing in Heart Failure: The HeartSync-LBBP Randomized Clinical Trial

**DOI:** 10.1001/jamacardio.2026.0083

**Published:** 2026-03-11

**Authors:** Xueying Chen, Xi Liu, Ruogu Li, Zhongkai Wang, Yixiu Liang, Lei Zhang, Wei Wang, Jin Bai, Jingfeng Wang, Shengmei Qin, Weiwei Zhang, Tianbao Yao, Dong Huang, Ting Chen, Xianxian Zhao, Dening Liao, Jingbo Li, Jialiang Mao, Mihail G. Chelu, Yangang Su, Kenneth A. Ellenbogen, Junbo Ge

**Affiliations:** 1 Department of Cardiology, Zhongshan Hospital, Fudan University, Shanghai Institute of Cardiovascular Diseases, Shanghai, China; 2 State Key Laboratory of Cardiovascular Diseases, Zhongshan Hospital, Fudan University, Shanghai, China; 3 NHC Key Laboratory of Ischemic Heart Diseases, Shanghai, China; 4 Key Laboratory of Viral Heart Diseases, Chinese Academy of Medical Sciences, Shanghai, China; 5 National Clinical Research Center for Interventional Medicine, Shanghai, China; 6 Department of Cardiology, Shanghai Chest Hospital, School of Medicine, Shanghai Jiao Tong University, Shanghai, China; 7 Department of Cardiology, Changhai Hospital, Second Military Medical University, Shanghai, China; 8 Department of Cardiology, Shanghai Renji Hospital, Shanghai Jiaotong University School of Medicine, Shanghai, China; 9 Department of Cardiology, Shanghai Jiao Tong University Affiliated Sixth People’s Hospital, Shanghai, China; 10 Department of Cardiology, Changzheng Hospital, Affiliated to Naval Medical University, Shanghai, China; 11 Department of Medicine (Section of Cardiology), Baylor College of Medicine, Houston, Texas; 12 Cardiovascular Research Institute, Baylor College of Medicine, Houston, Texas; 13 Texas Heart Institute at Baylor College of Medicine, Houston, Texas; 14 Division of Cardiology, Department of Medicine, Virginia Commonwealth University School of Medicine, Richmond, Virginia

## Abstract

**Importance:**

Left bundle-branch pacing (LBBP) has been proposed as an alternative to biventricular pacing (BiVP) for patients with heart failure with left bundle-branch block (LBBB). However, robust clinical evidence from randomized clinical trials is lacking.

**Objective:**

To evaluate the long-term clinical outcomes of LBBP and BiVP.

**Design, Setting, and Participants:**

This multicenter, prospective, randomized clinical trial enrolled 200 patients at 6 centers in China with a left ventricular ejection fraction (LVEF) of 35% or less and LBBB from October 2020 to March 2022. This study was took place from October 2020 to September 2024. These data were analyzed September 2024 to December 2024.

**Interventions:**

Patients were randomly assigned in a 1:1 ratio to receive either LBBP or BiVP.

**Main Outcomes and Measures:**

The primary end point was the time to death from any cause or heart failure hospitalization (HFH). The secondary end points included all-cause death, HFH, echocardiographic response (absolute increase in LVEF ≥5%), and super response (absolute increase in LVEF ≥15% or improvement of LVEF to ≥50%) rates.

**Results:**

Of the 200 included patients, 136 were male and 64 were female. The success rate was 98% in the LBBP group and 94% in the BiVP group (*P* = .28). The median follow-up duration was 36 (range, 33-39) months. The primary end point of time to death or HFH was significantly lower in the LBBP group compared with BiVP (8% vs 28%; hazard ratio [HR], 0.26; 95% CI, 0.12-0.57; *P* < .001). There was no significant difference in all-cause mortality between the groups (2.0% vs 5.0%; HR, 0.40; 95% CI, 0.08-2.04; *P* = .25). However, LBBP significantly reduced the risk of HFH (7.0% vs 28.0%; HR, 0.23; 95% CI, 0.10-0.52; *P* < .001). The echocardiographic response rates were similar in both groups (86.0% vs 81.0%; *P* = .34) but the super-response rate was higher in the LBBP group (55.0% vs 36.0%; *P* < .007).

**Conclusions and Relevance:**

In this study, LBBP was superior to BiVP in reducing the risk of death or HFH in patients with LBBB and severely reduced LVEF. Further trials are warranted in this patient population.

**Trial Registration:**

Chinese Clinical Trial Registry identifier: ChiCTR2000036554

## Key Points

**Question:**

Whether left bundle-branch pacing (LBBP) yields superior clinical outcomes compared with biventricular pacing (BiVP) in patients with heart failure (HF) with left bundle-branch block (LBBB) and severely reduced left ventricular ejection fraction (LVEF)?

**Findings:**

In this randomized clinical trial, LBBP was associated with a significantly lower risk of all-cause mortality and HF hospitalization when compared with BiVP.

**Meaning:**

These results demonstrated that LBBP was superior to BiVP in reducing the risk of death or HF hospitalization in patients with LBBB and severely reduced LVEF and might be an alternative to BiVP in this patient population.

## Teaser

This study attempts to evaluate the long-term clinical outcomes of left bundle-branch pacing and biventricular pacing in patients with heart failure.

## Introduction

For over 2 decades, biventricular pacing (BiVP) has been the standard method to address left ventricular dyssynchrony in patients with heart failure (HF) with reduced ejection fraction (HFrEF) and left bundle-branch block (LBBB).^1^ Numerous randomized clinical trials have demonstrated the benefit of BiVP to reduce mortality and heart failure hospitalization (HFH).^2,3,4,5,6,7^ The greatest benefit is derived by patients with LBBB and wider QRS duration.^8,9^ However, BiVP has limitations, including imperfect electrical resynchronization and suboptimal treatment responses dependent on QRS morphology, duration, and venous anatomy.

Introduction of left bundle-branch pacing (LBBP) by Huang et al^10,11^ has provided a new avenue for physiologic left ventricular resynchronization that may obviate the limitations of BiVP. This technique involves implantation of a pacing lead through the interventricular septum until it reaches the left ventricular subendocardial left bundle branch (LBB) or its fascicles. Large observational series have demonstrated that LBBP can result in greater improvement in clinical outcomes compared with BiVP.^12,13,14,15^

Consequently, we conducted the HeartSync-LBBP study, a prospective, multicenter, randomized, clinical trial with long-term follow-up to test the hypothesis that LBBP is superior to BiVP in patients with LBBB and HF.

## Methods

### Study Design and Population

This is a multicenter prospective randomized clinical trial designed to evaluate the differences in the long-term clinical outcomes of BiVP and LBBP. Patients who had a left ventricular ejection fraction (LVEF) of 35% or less, a New York Heart Association (NYHA) functional class II to IV, and complete LBBB were enrolled. Further inclusion and exclusion criteria are detailed in eTable 1 in Supplement 1. The study took place between October 2020 and September 2024 at 6 centers in China (eTable 2 in Supplement 1). The study followed the Consolidated Standards of Reporting Trials (CONSORT) reporting guidelines. This study was approved by the ethics committee of the enrolling centers and all patients provided written informed consent. The study complies with the Declaration of Helsinki, and all data are available from the corresponding author on reasonable request.

### Study Procedures

Eligible patients provided consent and were subsequently randomly assigned in a 1:1 ratio to receive either LBBP or BiVP.

LBBP was attempted, as previously described (eFigure 1 in Supplement 1).^16,17,18,19^ The criteria for determining LBB capture were primarily based on our previous findings and Chinese expert consensus on His-Purkinje conduction system pacing.^16,19,20^ LBBP was considered to be successful if the unipolar paced QRS morphology demonstrated a right bundle-branch block (RBBB) pattern and met any of the following criteria: (1) transition from nonselective LBBP to selective LBBP with constant left ventricular activation time (LVAT) during threshold testing and/or (2) transition from nonselective LBBP to left ventricular septal pacing (LVSP) with prolongation of LVAT by more than 10 milliseconds (eMethods in Supplement 1).^10,16,19,20^ For patients in whom LBB capture could not be confirmed by the above criteria, we used methods mentioned in other studies to assist in determining the success of LBBP.^21,22,23,24^ An LBB capture threshold 1.5 or more volts per 0.5 milliseconds was recognized as acceptable. Crossover was allowed when LBB capture could not be achieved. Atrioventricular delay was adjusted to achieve the narrowest QRS duration.^17^

BiVP was performed in a standard fashion with placement of a lead in a lateral or posterolateral branch. Crossover was allowed when a left ventricular (LV) lead could not be implanted due to unfavorable venous anatomy or when there was a high pacing threshold or unavoidable phrenic nerve stimulation. The atrioventricular and ventriculo-ventricular delay were optimized to achieve the shortest paced QRS duration.

Clinical parameters and electrocardiograms were collected at 1, 3, and 6 months postprocedure and every 6 months thereafter. Paced QRS duration was measured from the first deflection (defined as the QRS onset) to the end of the QRS. Echocardiographic measurements were performed by 2 experienced echocardiographers blinded to the study design and were collected at implantation and follow-ups every 6 months. LVEF was calculated using the biplane Simpson method from apical 2-chamber and 4-chamber views via 2-dimensional transthoracic echocardiography. Echocardiographic response was defined as an absolute improvement in LVEF 5% or more. Super-response rate was defined as an absolute improvement in LVEF 15% or more or improvement of LVEF to 50% or more. Procedure-related complications, including pneumothorax, pocket infection, lead dislodgement requiring revision, increased pacing threshold more than 3 volts per 0.5 milliseconds, and pericardial tamponade were documented during follow-up.

### Trial End Points

The primary end point was the composite of all-cause mortality and HFH. HFH was defined as any urgent visit or hospitalization with HF signs or symptoms requiring intravenous diuretic therapy. Secondary end points included all-cause mortality, HFH, and echocardiographic response/super response. Echocardiographic response was assessed at 6-month follow-up. All the efficacy and safety end points were independently adjudicated by a committee whose members were unaware of the trial-group assignments and the identity of the patients. All members reviewed the events independently and rendered a decision. In cases of disagreement, a consensus meeting was convened to reach a final decision.

### Statistical Analysis

When this study was designed, to our knowledge, there were no long-term clinical results on LBBP. Based on its favorable electrical improvement shown in early studies and the preliminary experience of the enrolled centers, the event rate in the LBBP group was assumed to be 10%. The event rates of BiVP reported in previous studies vary significantly, so the event rate in the BiVP group was finally assumed to be 25% based on retrospective data of the enrolling centers. A sample size of 190 patients was calculated to detect a statistically significant difference in the primary end point with 80% power and 2-tailed α level of 5%, assuming 10% event rate in the LBBP and 25% in the BiVP arm with 10% dropout and 5% crossover. To ensure sufficient statistical efficiency, the sample size was determined to be 100 for each group. Continuous variables were described as mean (SD) and categorical variables were described as frequencies or percentages. *t* Test was performed for normally distributed continuous variables while Wilcoxon signed rank test was for nonnormally distributed data. The χ^2^ test or Fisher-exact test was used for categorical variables. All analyses in this study were conducted according to the intention-to-treat principle. For the primary and secondary clinical end points, Kaplan-Meier survival curve and log-rank test were performed to compare the time-to-event between LBBP and BiVP. Subgroup analyses of the relationship between the primary end point and the baseline characteristics including age, sex, etiology, comorbidities, QRS duration, NYHA class, and LVEF were conducted. A 2-sided *P* value <.05 was considered statistically significant. All statistical analyses were performed using SPSS Statistics version 22.0 (IBM).

## Results

### Patients and Follow-Up

**Figure 1.**
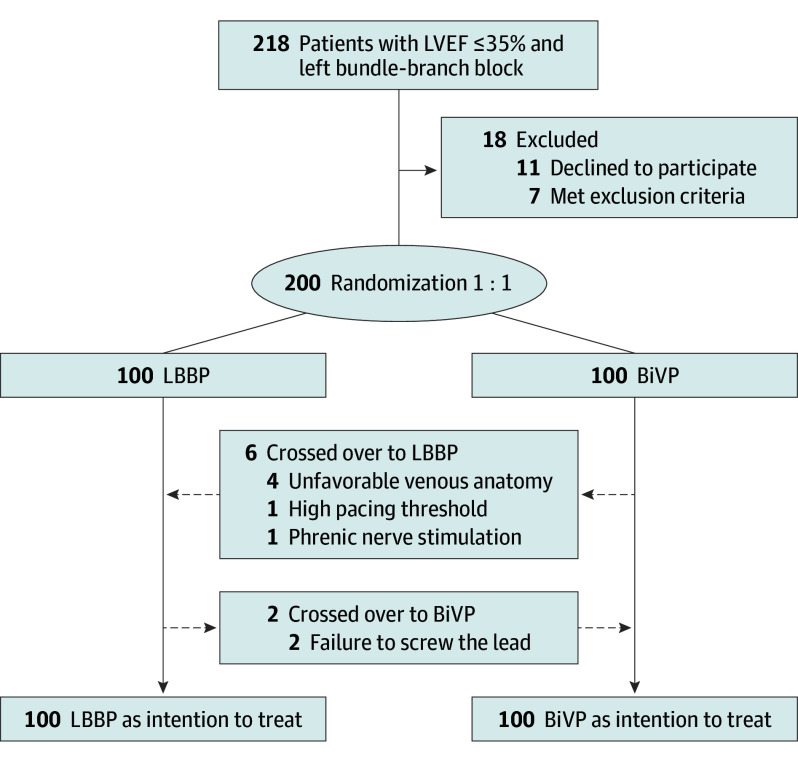
CONSORT Diagram of the Study Cohort BiVP indicates biventricular pacing; LBBP, left bundle-branch pacing; LVEF, left ventricular ejection fraction.

From October 2020 to March 2022, a total of 200 patients were randomized in a 1:1 ratio to receive either LBBP or BiVP (Figure 1). The baseline characteristics were balanced between the 2 groups (Table 1). Overall, the mean (SD) age was 64.8 (9.5) years, 68.0% were male and 32% were female, 82.5% had nonischemic cardiomyopathy (NICM), and the mean (SD) NYHA functional class was 2.9 (0.6). At baseline, mean (SD) LVEF was 28.2% (4.5%) and the mean (SD) QRS duration was 168.4 (18.5) milliseconds. Most patients received optimal medical treatment.

**Table 1.** Baseline Clinical and Demographic Characteristics

Characteristic	No. (%)	
	LBBP (n = 100)	BiVP (n = 100)
Demographics		
Age, y, mean (SD)	64.3 (9.5)	65.3 (9.5)
Sex		
Male	67 (67.0)	69 (69.0)
Female	33 (33.0)	31 (31.0)
Nonischemic cardiomyopathy	84 (84.0)	81 (81.0)
Comorbidities		
Hypertension	26 (26.0)	28 (28.0)
Diabetes	21 (21.0)	23 (23.0)
Baseline QRS duration, ms, mean (SD)	169.8 (19.0)	167.0 (18.0)
NT-pro BNP, pg/mL, mean (SD)	3618.0 (3326.9)	3720.5 (3419.9)
NYHA class, mean (SD)	2.9 (0.5)	3.0 (0.6)
Echocardiography, mean (SD)		
LVEF, %	28.3 (3.8)	28.1 (5.0)
LVEDD, mm	66.1 (7.3)	66.9 (7.0)
LVESD, mm	56.6 (8.3)	56.9 (8.1)
Medications		
ACEI/ARB/ARNI	97 (97.0)	98 (98.0)
β-Blockers	96 (96.0)	95 (95.0)
Spironolactone	94 (94.0)	92 (92.0)
SGLT-2i	41 (41.0)	43 (43.0)

Abbreviations: ACEI, angiotensin-converting enzyme inhibitor; ARB, angiotensin II receptor blocker; ARNI, angiotensin receptor-neprilysin inhibitor; BiVP, biventricular pacing; LBBP, left bundle-branch pacing; LVEDD, left ventricular end-diastolic diameter; LVEF, left ventricular ejection fraction; LVESD, left ventricular end-systolic diameter; NT-pro BNP, N-terminal pro B-type natriuretic peptide; NYHA, New York Heart Association; SGLT-2i, sodium glucose co-transporter 2 inhibitors.

### Treatment Characteristics

**Figure 2.**
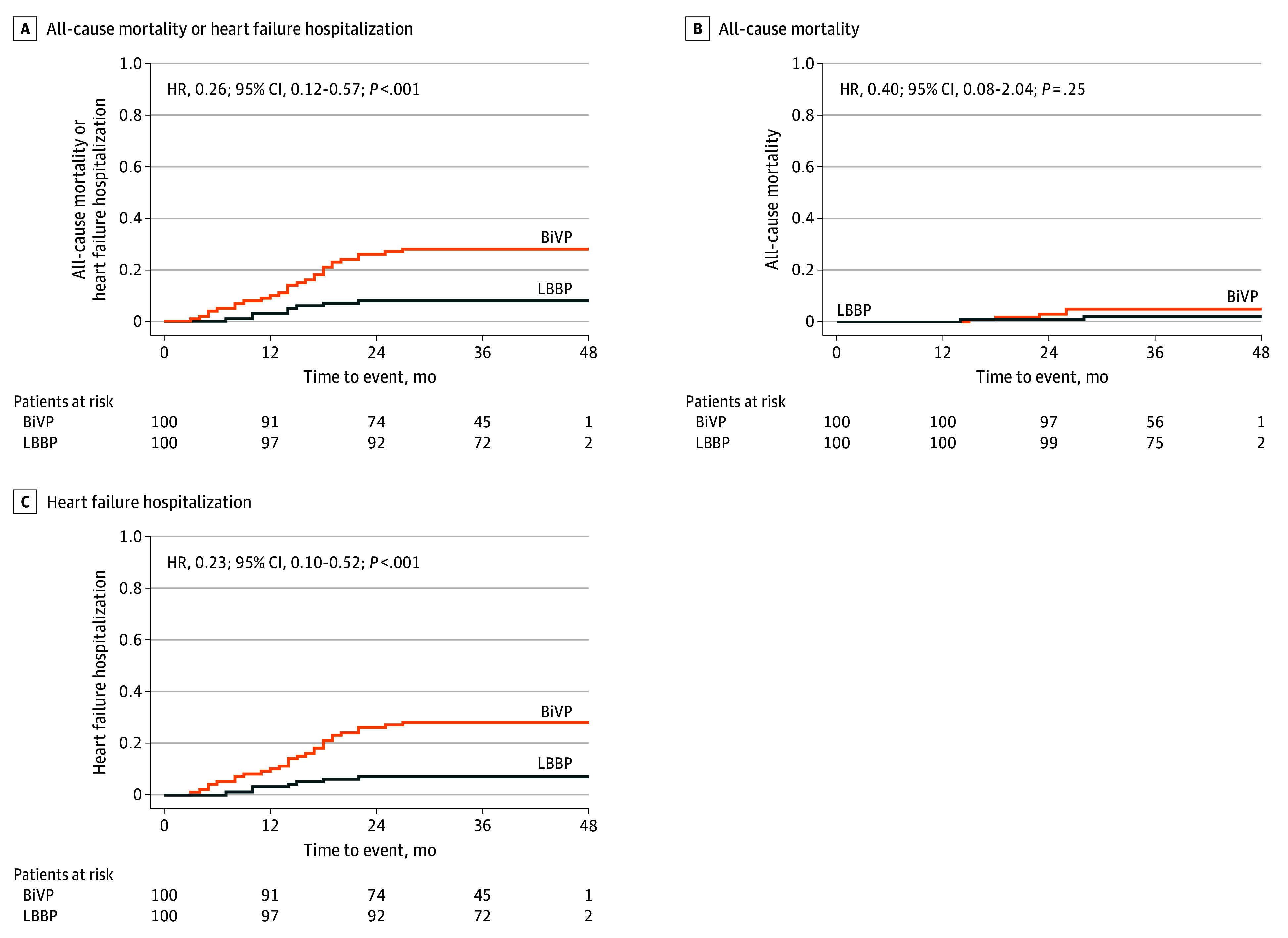
Kaplan-Meier Estimates of All-Cause Mortality and Heart Failure Hospitalization BiVP indicates biventricular pacing; HR, hazard ratio; LBBP, left bundle-branch pacing.

The initial implant attempt success rate was 98% (98 of 100) in the LBBP group and 94% (94 of 100) in the BiVP group. As shown in Figure 1 and Figure 2, patients assigned to the LBBP crossed over to BiVP due to inability to advance the lead deep into the septum and capture the LBB. Among patients who underwent LBBP, His bundle potential was recorded in 92 patients (93.9%), with 90.2% of them demonstrating LBBB correction by high-output temporary His bundle pacing (HBP) (eTable 3 in Supplement 1). All patients demonstrated an RBBB pattern during the procedure and direct evidence for LBB capture was observed in 75.5%, including transition from nonselective LBBP to selective LBBP (73.5%) and nonselective LBBP to LVSP (2.0%) (eTable 3 in Supplement 1). The paced morphology in most patients demonstrated with an R, Rs, or RS shapes in lead II, while 12.2% of the patients present an rS or QS pattern in lead II. All patients demonstrated a nonselective LBBP pattern after the procedure with a mean (SD) LVAT of 84.4 (14.1) milliseconds. In the BiVP group, 6 patients crossed over to LBBP due to unfavorable venous anatomy (n = 4), high pacing threshold (n = 1), or phrenic nerve stimulation (n = 1). All 6 patients underwent the opposite-arm procedure successfully. The mean paced QRS duration for the LBBP arm was significantly shorter than that for the BiVP arm at implantation and at last follow-up (Table 2).

**Table 2.** Procedural and Follow-Up Data

Procedure	No. (%)		*P* value
	LBBP (n = 100)	BiVP (n = 100)	
Type of device			
CRT-P	17 (17.0)	23 (23.0)	.29
CRT-D	83 (83.0)	77 (77.0)	
Paced QRS duration, ms, mean (SD)	120.6 (18.1)	137.4 (15.8)	<.001
Procedural time, min, mean (SD)	100.5 (29.6)	96.5 (28.5)	.20
Fluoroscopy time, min, mean (SD)	13.5 (7.9)	12.7 (7.7)	.11
Pacing threshold, mean (SD)			
At implantation (V per 0.5 ms)	0.9 (0.3)	1.5 (0.3)	<.001
At follow-up (V per 0.5 ms)	0.8 (0.3)	1.5 (0.4)	<.001
Echocardiography at 6-mo follow-up, mean (SD)			
LVEF, %	45.0 (9.6)	39.2 (7.4)	<.001
LVEDD, mm	57.2 (8.6)	61.5 (8.0)	<.001
LVESD, mm	44.1 (10.6)	50.1 (9.9)	<.001
Echocardiography at last follow-up, mean (SD)			
LVEF, %	47.3 (10.6)	41.5 (8.6)	<.001
LVEDD, mm	55.3 (6.7)	60.3 (7.2)	<.001
LVESD, mm	41.8 (9.2)	48.1 (9.9)	<.001
Echocardiographic response	90 (90.0)	84 (84.0)	.21
Echocardiographic super response	65 (65.0)	44 (44.0)	.003
NYHA class at last follow-up	1.9 (0.7)	2.3 (0.8)	<.001
Paced QRS duration at last follow-up, ms	119.4 (14.9)	136.6 (11.6)	<.001
Complications			
Pneumothorax	0	0	NA
Pocket infection	0	0	NA
Pericardial tamponade	0	0	NA
Lead dislodgement	0	1 (1.0)	>.99
Increase in pacing threshold	0	2 (2.0)	.50

Abbreviations: BiVP, biventricular pacing; CRT-D, cardiac resynchronization therapy with defibrillator; CRT-P, cardiac resynchronization therapy with pacemaker; LBBP, left bundle-branch pacing; LVEDD, left ventricular end-diastolic diameter; LVEF, left ventricular ejection fraction; LVESD, left ventricular end-systolic diameter; NA, not applicable; NYHA, New York Heart Association.

### End Points

At a median of 36 months’ follow-up, the primary end point occurred in 8 of 100 patients (8.0%) assigned to undergo LBBP and 28 of 100 patients assigned to undergo BiVP (hazard ratio [HR], 0.26; 95% CI, 0.12-0.56; *P* < .001) (Figure 2A; Table 3). The treatment effect for the primary end point was consistent across most of the prespecified subgroups (eFigure 2 in Supplement 1). There was no significant difference observed in all-cause mortality between LBBP and BiVP (2.0% vs 5.0%; HR, 0.40; 95% CI, 0.08-2.04; *P* = .25) (Figure 2B; Table 3). There was a significantly lower risk of HFH in favor of LBBP (7.0% vs 28.0%; HR, 0.23; 95% CI, 0.10-0.52; *P* < .001) (Figure 2C; Table 3).

**Table 3.** Primary and Secondary End Points

End point	No. of patients (%)		HR (95% CI)	*P* value
	LBBP (n = 100)	BiVP (n = 100)		
Primary end point				
Composite of all-cause mortality and HFH	8 (8.0)	28 (28.0)	0.26 (0.12-0.57)	<.001
Secondary end points				
All-cause mortality	2 (2.0)	5 (5.0)	0.40 (0.08-2.04)	.25
HFH	7 (7.0)	28 (28.0)	0.23 (0.10-0.52)	<.001
Echocardiographic response	86 (86.0)	81 (81.0)	NA	.34
Echocardiographic super response	55 (55.0)	36 (36.0)	NA	.007

Abbreviations: BiVP, biventricular pacing; HFH, heart failure hospitalization; HR, hazard ratio; LBBP, left bundle-branch pacing; NA, not applicable.

Both groups had significant improvements in LVEF, left ventricular end-diastolic diameter (LVEDD), and left ventricular end-systolic diameter (LVESD) compared with baseline with all improvements favoring LBBP compared with BiVP (Table 2). There were no significant differences observed in the echocardiographic response rate between the 2 groups (86.0% vs 81.0%; *P* = .34), but the super-response rate was significantly higher in the LBBP group compared with the BiVP group (55.0% vs 36.0%; *P* < .007) (Table 3).

Both groups demonstrated significant improvements in NYHA functional class at last follow-up compared with baseline (−1.00; 95% CI, −1.18 to −0.82; *P* < .001), with the LBBP group exhibiting a better functional class than the BiVP group (−0.67; 95% CI, −0.86 to −0.48; *P* < .001).

The pacing capture threshold was significantly lower in LBBP than in BiVP at implantation and at the time of the last follow-up (Table 2). There were no major complications in the LBBP group. One lead dislodgement and an increase in pacing threshold in 2 cases were recorded in the BiVP group (Table 2).

## Discussion

To our knowledge, this is the largest prospective, multicenter, randomized, clinical study comparing the long-term clinical outcomes of LBBP vs BiVP in patients with HF with LVEF of 35% or less and LBBB. We demonstrated that LBBP is superior to BiVP for the composite end point of all-cause mortality and HFH. This was driven primarily by a decreased risk of HFH in the LBBP group. These findings might be explained by a greater improvement in electrical synchrony, ventricular remodeling, and consequent higher rate of super response in the LBBP group compared with the BiVP group.

### Modalities of Cardiac Resynchronization Therapy

BiVP is recommended as a first-line therapy by American Heart Association/American College of Cardiology/Heart Rhythm Society and European Society of Cardiology guidelines.^25^ However, the success rate can be limited by anatomical variations in the coronary sinus, high pacing thresholds, and phrenic nerve stimulation.^26^ More importantly, about 30% of cardiac resynchronization therapy (CRT)–eligible patients may not respond to BiVP.^27^ HBP with bundle branch-block correction is the most physiologic pacing treatment as it preserves inter- and intraventricular synchrony.^28^ Certain disadvantages, including higher pacing threshold and risk of lead dislodgment, have greatly limited its application.^29^ LBBP has emerged as a preferred method over HBP due to its broader target area.^30^ This anatomical advantage facilitates higher implantation success rates and enhances long-term safety.^31^ LVSP is also considered as a conduction system pacing (CSP) modality. Unlike LBBP, the lead is positioned at the LV septum myocardium without capturing the LBB. Due to the similar pacing characteristics between LVSP and LBBP, they are collectively referred to as left bundle-branch area pacing (LBBAP).^1,32^

### Success Rate of LBBP

Previous studies reported the success rate of LBBP ranged from 82.2% to 97.8%.^15,31,33,34^ In this study, the success rate of LBBP was 98%, similar to our previous results and higher than those reported by other teams. The above differences in success rates may be related to the implantation experience and the characteristics of enrolled patients. A single-center study reported the learning curve of LBBAP and showed that LVAT tended to stabilize after more than 200 LBBAP implantations.^35^ Results from the multicenter European MELOS study showed that the success rate of LBBAP stabilized after more than 270 implantations.^31^ These studies collectively indicate that LBBAP requires a certain long learning period. LBBP, which requires confirmation of LBB capture, involves higher procedural demands and may require a longer learning curve (eTable 4 in Supplement 1). Therefore, success rates can vary significantly across centers with differing levels of implantation experience. The success rate observed here is consistent with our previous reports.^33^ In addition, patients with LBBB are generally more amenable to LBBP than those with intraventricular conduction delay (IVCD), further contributing to a higher success rate. Furthermore, most participants had NICM. This subgroup has a lower probability of myocardial scar, which may also have facilitated successful lead placement. In addition, we mapped the His bundle in 93.9% of participants, which allows a more accurate localization of the LBB. Taken together, these factors likely account for the relatively high LBBP success rate observed in this study.

### Criteria for Confirmation of LBB Capture

In this study, the LBB capture was confirmed in most patients by direct criteria (eMethods in Supplement 1): paced morphology showing an RBBB pattern with morphology changes—a criterion with both high sensitivity and specificity.^10,19,20^ For the minority of patients in whom LBB capture could not be determined by the direct criteria, we used indirect criteria including LVAT values, V6-V1 interpeak interval, or programmed stimulation.^21,22,23,24^ It should be noted that, recent studies have demonstrated that many of the criteria described originally are not 100% sensitive or specific, especially in patients with advanced conduction disease.^36,37^ This study enrolled patients with LBBB, most of whom could be corrected by high-output temporary HBP, indicating a high proportion of true LBBB. This likely facilitated the identification of LBB capture using conventional criteria.

### Differences Between LBBP and LVSP

Although current CSP guidelines group LBBP and LVSP under the umbrella term *LBBAP*, emerging evidence suggests they may represent fundamentally distinct pacing modalities due to differences in LBB capture.^14,32^ A more consistent finding is that LBBP is associated with a shorter LVAT, suggesting better LV electrical resynchronization.^32,38^ Controversies persist regarding other outcome measures, such as pacing QRS duration and interventricular synchrony.^1,39^ Our previous studies showed that LBBP delivers greater electrical, mechanical and hemodynamic improvement than BiVP in patients with NICM with LBBB.^40^ In contrast, studies focusing on the broader category of LBBAP have reported greater acute hemodynamic benefits with BiVP than with LBBAP.^41^ This discrepancy may be attributable to a higher proportion of LVSP cases in the latter studies, which could have diluted the benefits typically observed with true LBB capture. With respect to clinical outcomes, recent retrospective studies suggest that LVSP may result in worse outcomes compared with both BiVP and LBBP.^14^ These discrepancies highlight the need for trials to clarify the comparative effectiveness of these pacing modalities.

### End Points of LBBP and BiVP

Previous studies have reported that the incidence of the primary end point for BiVP ranges from 26.0% to 42.4%.^13,14,15^ Most published studies assessing outcomes of LBBP included both patients with LBBP and LVSP, with event rates ranging from 7.4% to 24.2%.^13,14,15^ Recent research suggests that LVSP result in poorer clinical outcomes compared with BiVP and LBBP.^14^ The inclusion of patients with LVSP in prior studies may have diluted the overall treatment effect attributed to LBBP and underestimated its clinical benefit. Few studies have evaluated LBBP alone; among them, 1 reported an event rate of 7.4%, which is consistent with our findings in a population of similar ethnic background.^33^ Additionally, most patients in the present study had paced morphologies with R or r waves in lead II, suggesting lead placement near the proximal main trunk of the LBB. This contrasts with the fascicular pacing-dominant approach reported in the MELOS study, highlighting potential better electrical synchronization which might also result in better clinical outcomes.

A distinctive feature of our study is that all enrolled patients had rigorously confirmed true LBBB, as evidenced by correction of LBBB with high-output HBP at implantation, and more than 80% had NICM—2 characteristics associated with better response to CRT and lower mortality. This combination strongly suggests that a substantial proportion of patients had LBBB-induced cardiomyopathy, in which ventricular dysfunction is driven primarily by electrical dyssynchrony rather than irreversible myocardial injury. In such patients, restoring physiologic activation through LBBP directly eliminates the causal substrate for ventricular dysfunction. Thus, LBBP in this population functions not merely as supportive therapy but as a mechanistic, disease-modifying intervention, addressing the root cause of the cardiomyopathy. This pathophysiologic alignment likely explains both the profound reverse remodeling observed and the remarkably low long-term mortality, consistent with near-complete correction of the underlying disease process. In addition, it should be noted that although this study is a randomized clinical trial, several factors in the BiVP group at the final enrollment may lead to the poorer prognosis of this group, including a slightly older age, a higher proportion of male, a lower proportion of NICM, a higher proportion of diabetes, a slightly higher NYHA functional class, a slightly lower LVEF, slightly higher LVEDD and LVESD, as well as slightly lower usage rates of spironolactone, β blockers, and defibrillator.

### Future Perspectives

Although large observational studies have evaluated the long-term end points of CSP in CRT-eligible patients, current results suggest that differences in pacing modalities (LBBP or LVSP), types of conduction system diseases (LBBB or IVCD), and etiologies of HF may all lead to different study outcomes. Our findings support the clinical benefit of LBBP in patients with LBBB and NICM. Future studies in more diverse populations with different etiologies are needed to comprehensively assess the efficacy of LBBP in HF.

### Limitations

Our trial has several limitations. First, all participants were Chinese. Although consecutive enrollment was used, the cohort included a relatively high proportion of patients with NICM, likely reflecting regional differences in disease distribution. Therefore, the results of this study may not be generalizable to patients with ischemic cardiomyopathy or other ethnic groups. However, these findings may offer valuable treatment insights, particularly for those with NICM. Second, all procedures were performed at experienced centers with high implant success rates and low crossover rates. Thus, the results may not be generalizable to centers without similar technical expertise. Third, the study did not include a systematic assessment of LBB capture during follow-up, though the stable paced QRS duration over time suggests that a substantial proportion of patients likely maintained LBB capture. Lastly, the lack of cardiac magnetic resonance imaging data to assess myocardial scar burden limits our ability to determine factors underlying LBBP failure or lack of efficacy.

## Conclusions

In this randomized clinical trial involving patients with HFrEF and LVEF of 35% or less and LBBB, treatment with LBBP compared with BiVP yielded superior long-term outcomes. Further trials are warranted in this patient population.

## Notes

Supplement 1. eTable 1. Inclusion and exclusion criteria eTable 2. Participating centers eTable 3. Procedure-related characteristics in the LBBP group eTable 4. LBBP implantation experience before this study eFigure 1. Procedure process in the LBBP group eFigure 2. Subgroup analyses of the primary endpoint

Supplement 2. Trial Protocol and Statistical Analysis Plan

Supplement 3. Data sharing statement
