# Supplementary material for: Long-Term Outcomes of Left Bundle-Branch Pacing vs Biventricular Pacing in Heart Failure: The HeartSync-LBBP Randomized Clinical Trial
Source: JAMA Cardiol. 2026 Mar 11;11(4):352–9. doi: 10.1001/jamacardio.2026.0083 (PMC12980356; doi:10.1001/jamacardio.2026.0083)
Supplement: Supplement 1. — eTable 1. Inclusion and exclusion criteria eTable 2. Participating centers eTable 3. Procedure-related characteristics in the LBBP group eTable 4. LBBP implantation experience before this study eFigure 1. Procedure process in the LBBP group eFigure 2. Subgroup analyses of the primary endpoint [file jamacardiol-e260083-s001.pdf]

Supplemental Online Content

Chen X, Liu X, Li R, et al. Long-term outcomes of left bundle branch pacing vs biventricular pacing in heart failure: the HeartSync-LBBP randomized clinical trial. *JAMA Cardiol*. Published online March 11, 2026. doi:10.1001/jamacardio.2026.0083

eTable 1. Inclusion and exclusion criteria
eTable 2. Participating centers
eTable 3. Procedure-related characteristics in the LBBP group
eTable 4. LBBP implantation experience before this study
eFigure 1. Procedure process in the LBBP group
eFigure 2. Subgroup analyses of the primary endpoint
eMethods. ECG data in the LBBP group

This supplemental material has been provided by the authors to give readers additional information about their work.

© 2026 Chen X et al. *JAMA Cardiol*.

eTable 1. Inclusion and exclusion criteria

**Inclusion and exclusion criteria**
**Inclusion criteria**
1) age 18 to 80 years;
2) sinus rhythm, LBBB as defined by Strauss criteria (QRS duration ≥ 140 ms for men or ≥ 130 ms for women, QS or rS in leads V1 and V2, and mid-QRS notching or slurring in ≥ 2 of leads V1, V2, V5, V6, I, and aVL);
3) LVEF ≤ 35% and NYHA functional class II to IV despite receiving at least 3 months of maximally tolerated guideline directed medical therapy;
4) expected survival time > 1 year;
5) stable residency and ability to complete follow-up requirements during the study period.
**Exclusion criteria**
1) inability to provide written informed consent;
2) participation in other clinical studies;
3) persistent or permanent atrial fibrillation;
4) patients with heart transplant or awaiting heart transplantation;
5) previous pacemaker implantation;
6) prior mechanical tricuspid valve implantation;
7) severe renal insufficiency;
8) severe liver dysfunction;
9) pregnancy.

LBBB, left bundle branch block; LVEF, left ventricular ejection fraction; NYHA, New York Heart Association.

© 2026 Chen X et al. *JAMA Cardiol*.

eTable 2. Participating centers

No.	Participating center
1	Zhongshan Hospital of Fudan University
2	Shanghai Chest Hospital, Shanghai Jiao Tong University
3	Shanghai Changhai Hospital, Naval Medical University
4	Shanghai Changzheng Hospital, Second Affiliated Hospital of Naval Medical University
5	Renji Hospital, Shanghai Jiao Tong University School of Medicine
6	Shanghai Jiao Tong University Affiliated Sixth People's Hospital

© 2026 Chen X et al. *JAMA Cardiol*.

eTable 3. Procedure-related characteristics in the LBBP group

	LBBP (n=98)
His bundle potential recorded	92 (93.9%)
LBBB correction	83 (90.2%)
Criteria for confirm LBB capture	
Unipolar paced morphology demonstrated a RBBB pattern	98 (100%)
Transition from nonselective LBBP to selective LBBP	72 (73.5%)
Transition from nonselective LBBP to LVSP	2 (2.0%)
Indirect criteria	24 (24.5%)
Characteristics of paced morphology	
“R” or “Rs” or “RS” shapes in lead II	86 (87.8%)
“rS” or “QS” shapes in lead II	12 (12.2%)
Left ventricular activation time (ms)	84.4 ± 14.1
V6-V1 interpeak interval	44.0 ± 13.4
Final capture type after the procedure	
Selective LBBP	0 (0.0%)
Nonselective LBBP	98 (100.0%)
LVSP	0 (0.0%)

LBB, left bundle branch; LBBB, left bundle branch block; LBBP, left bundle branch pacing; LVSP, left ventricular septal pacing; RBBB, right bundle branch block.

© 2026 Chen X et al. *JAMA Cardiol*.

eTable 4. LBBP implantation experience before this study

Participating center	Operator	Number of cases
Zhongshan Hospital of Fudan University	Xueying Chen	652
Shanghai Chest Hospital, Shanghai Jiao Tong University	Ruogu Li	379
Shanghai Changhai Hospital, Naval Medical University	Zhongkai Wang	297
Shanghai Changzheng Hospital, Second Affiliated Hospital of Naval Medical University	Ting Chen	278
Renji Hospital, Shanghai Jiao Tong University School of Medicine	Tianbao Yao	355
Shanghai Jiao Tong University Affiliated Sixth People's Hospital	Dong Huang	286

LBBP, left bundle branch pacing.

© 2026 Chen X et al. *JAMA Cardiol*.

Online-Only Figures

eFigure 1. Procedure process in the LBBP group.

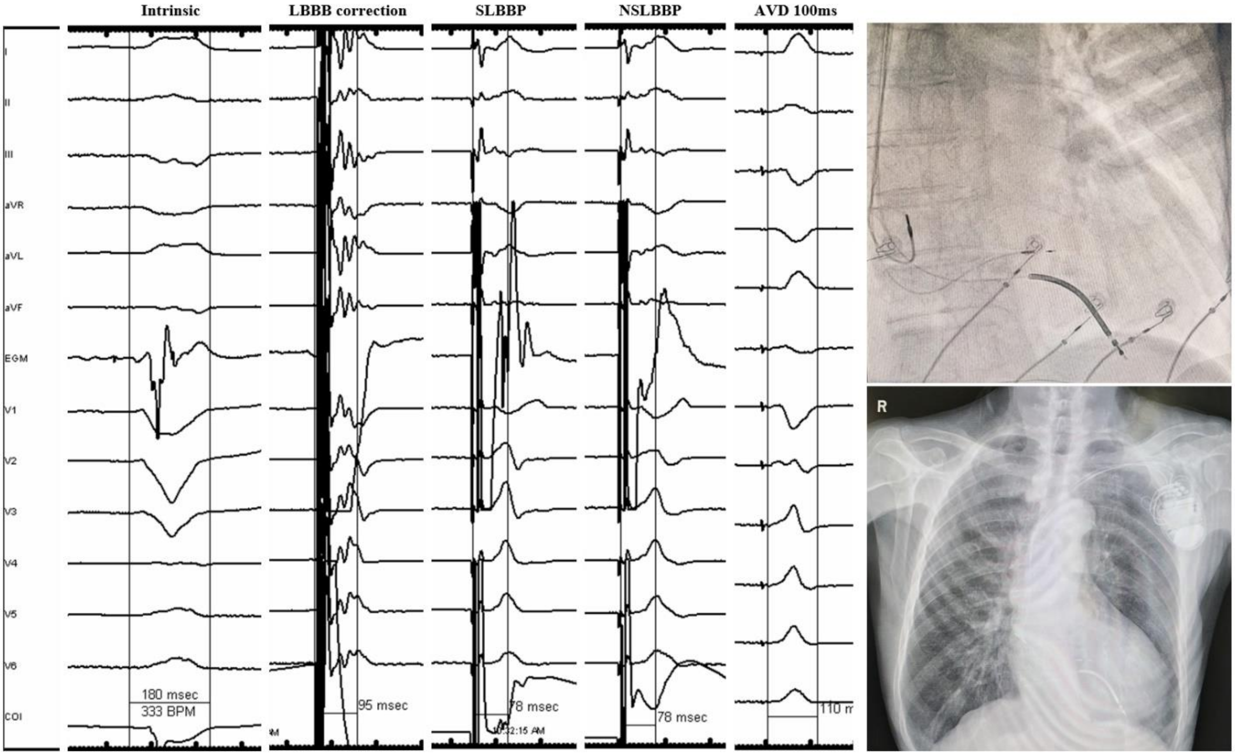

eFigure 1. Procedure process in the LBBP group. LBBP was attempted using the Select Secure 3830 pacing lead (Medtronic Inc, Minneapolis, MN) delivered through a fixed curve C315 HIS sheath (Medtronic Inc, Minneapolis, MN). In brief, the His bundle potential was first mapped with the pacing lead through the sheath, followed by pacing at high output to observe whether LBBB could be corrected. Then the lead was moved from the His bundle potential location towards the right ventricular apex approximately 1 to 2 cm in right anterior oblique 30° fluoroscopic view and was advanced deeply into the interventricular septum. AVD, AV delay; LBBB, left bundle branch block; LBBP, left bundle branch pacing; NSLBBP, nonselective left bundle branch pacing; SLBBP, selective left bundle branch pacing.

© 2026 Chen X et al. *JAMA Cardiol*.

eFigure 2. Subgroup analyses of the primary endpoint.

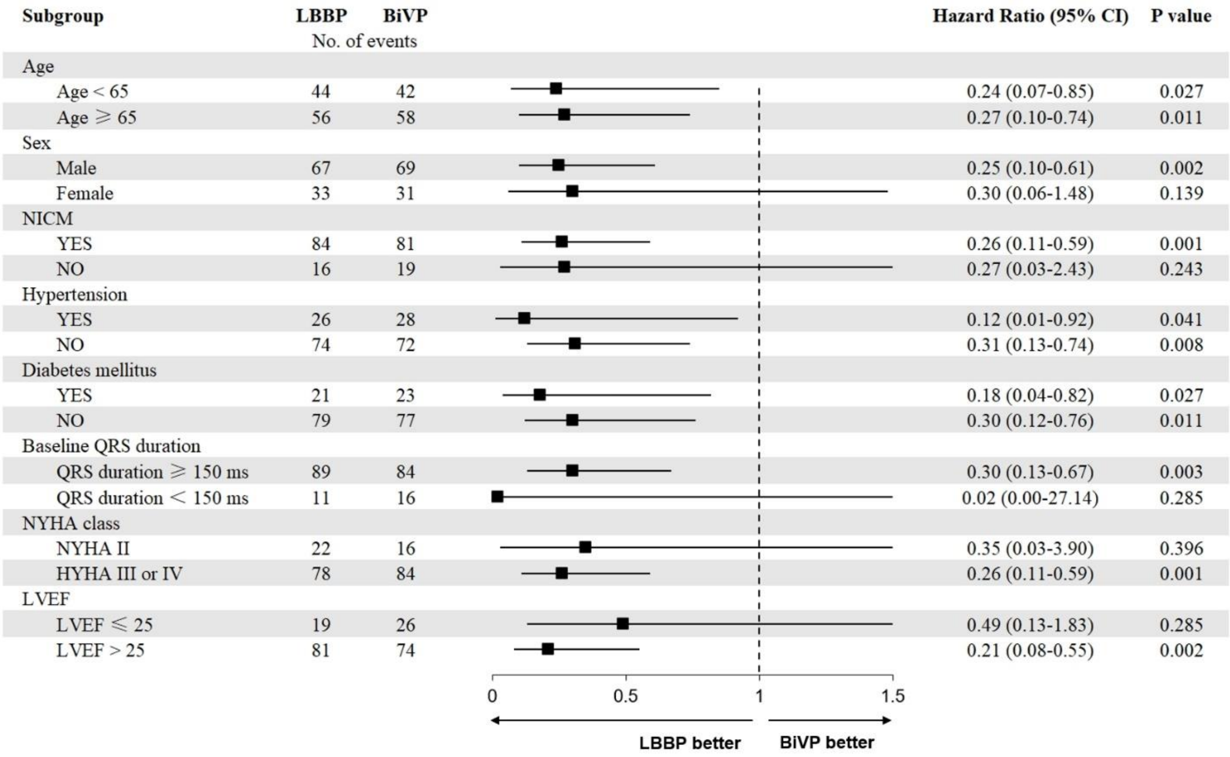

eFigure 2. Subgroup analyses of the primary endpoint. BiVP, biventricular pacing; CI, confidence interval; LBBP, left bundle branch pacing; LVEF, left ventricular ejection fraction; NICM, nonischemic cardiomyopathy; NYHA, New York Heart Association.

eMethods. ECG data in the LBBP group

2 failures switch to BiVP

Success LBBP: 98

1. Transitions: 74

(1) Selective to nonselective LBBP at different pacing output: 72

(2) LVSP to nonselective LBBP at different pacing output: 2

2. Indirect criteria: 24

Patient 1:
Pre-ECG

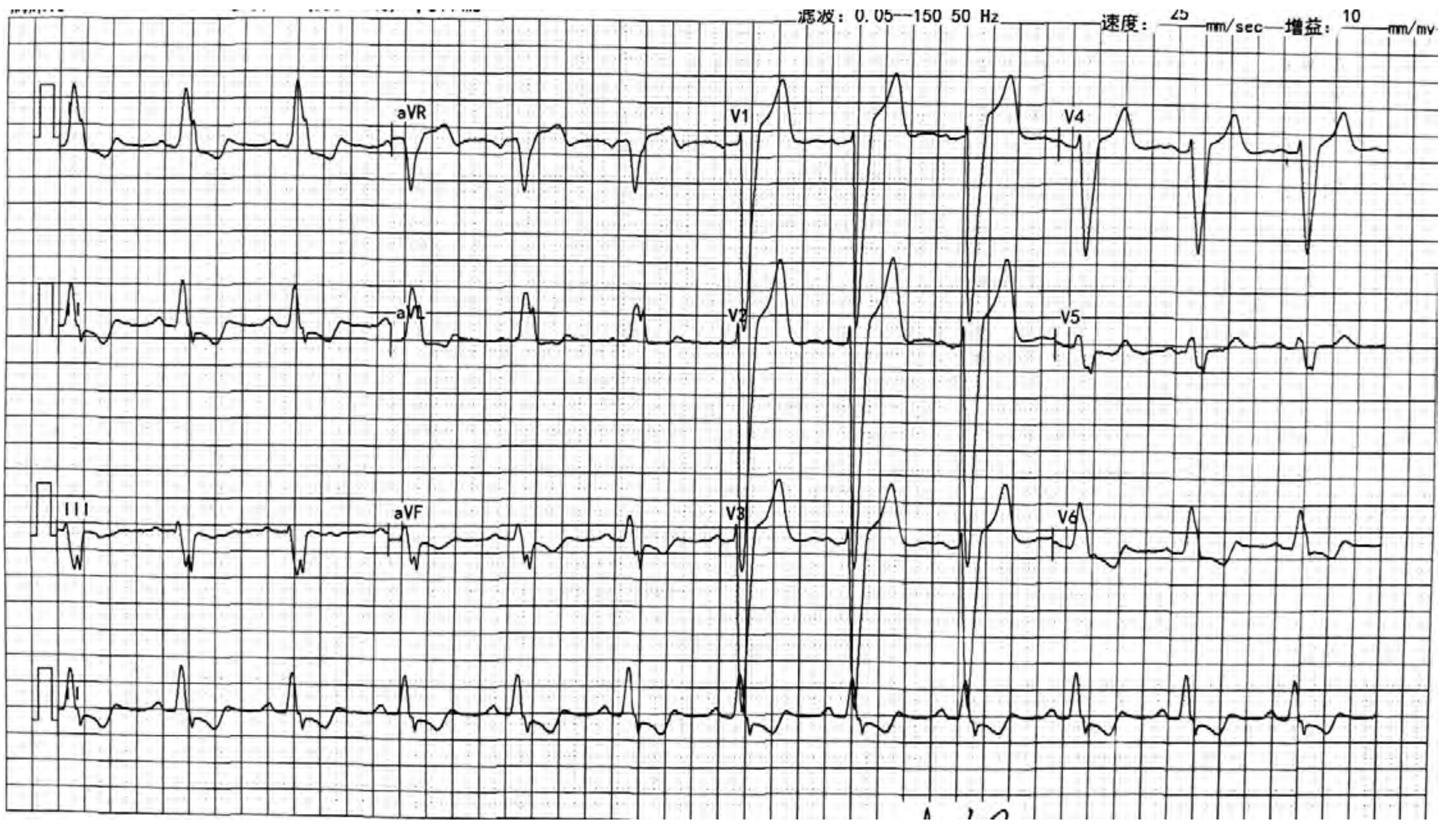

Post ECG

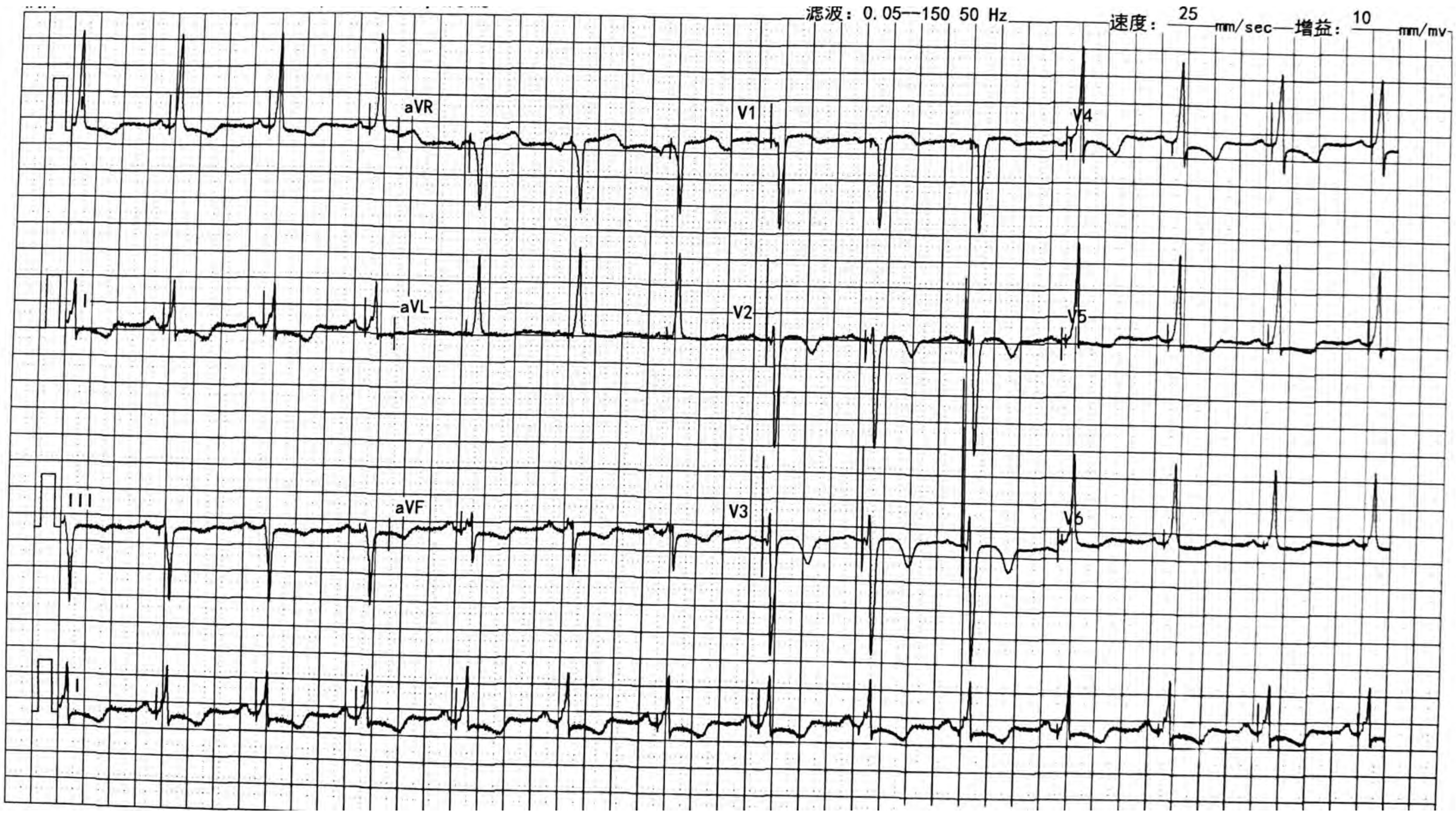

Patient 2:
Pre-ECG

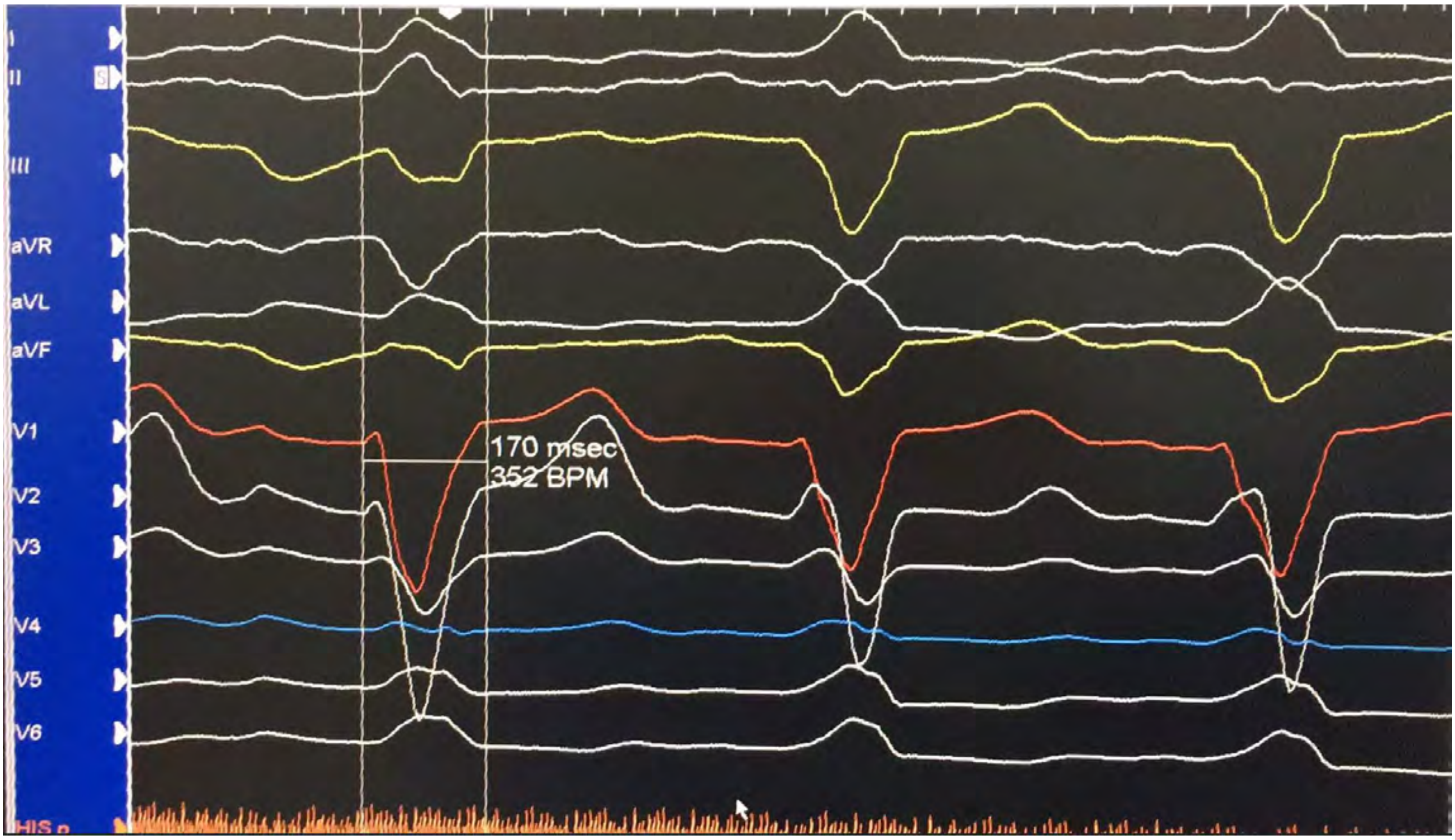

Post ECG

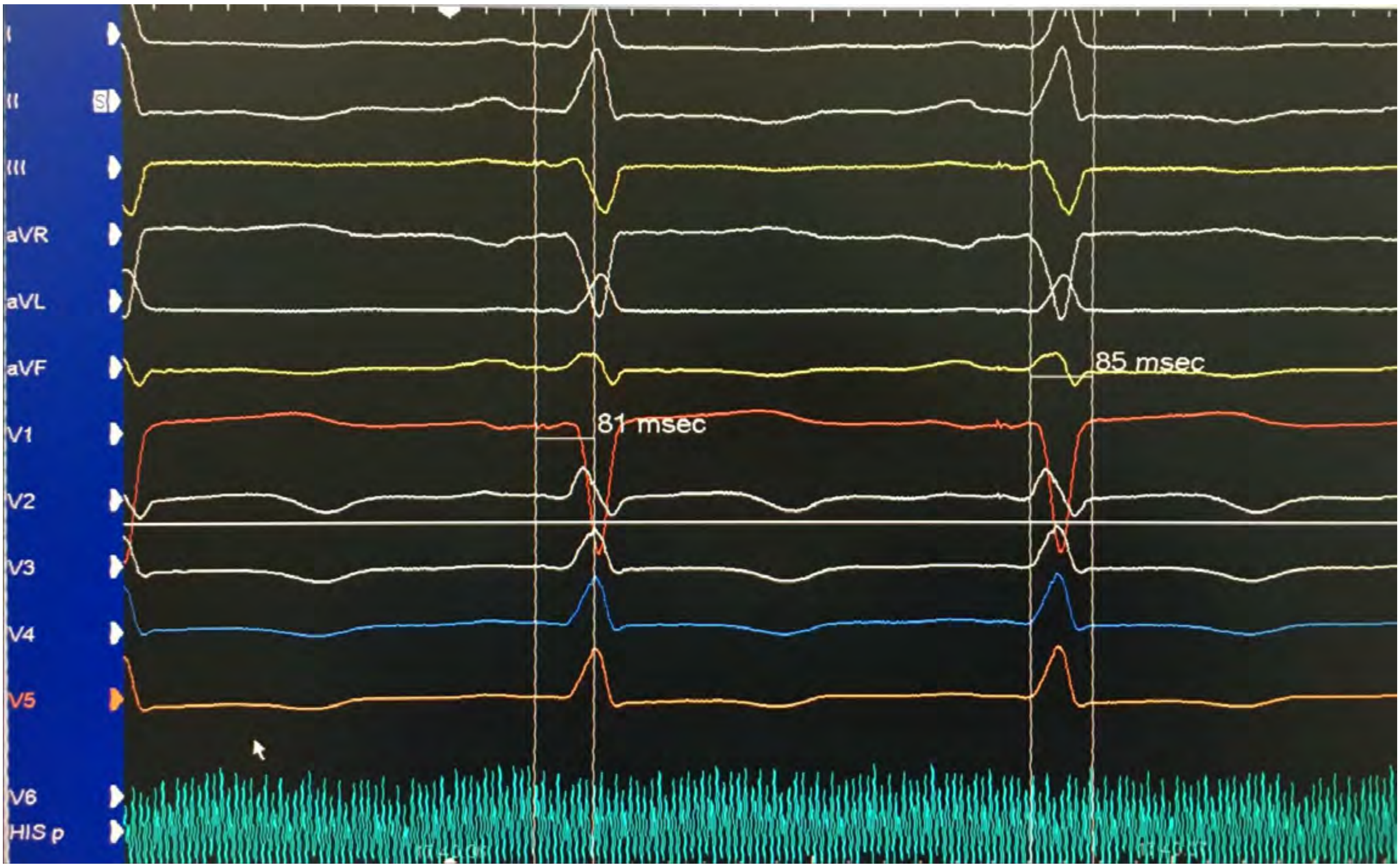

Patient 2:
Transitions

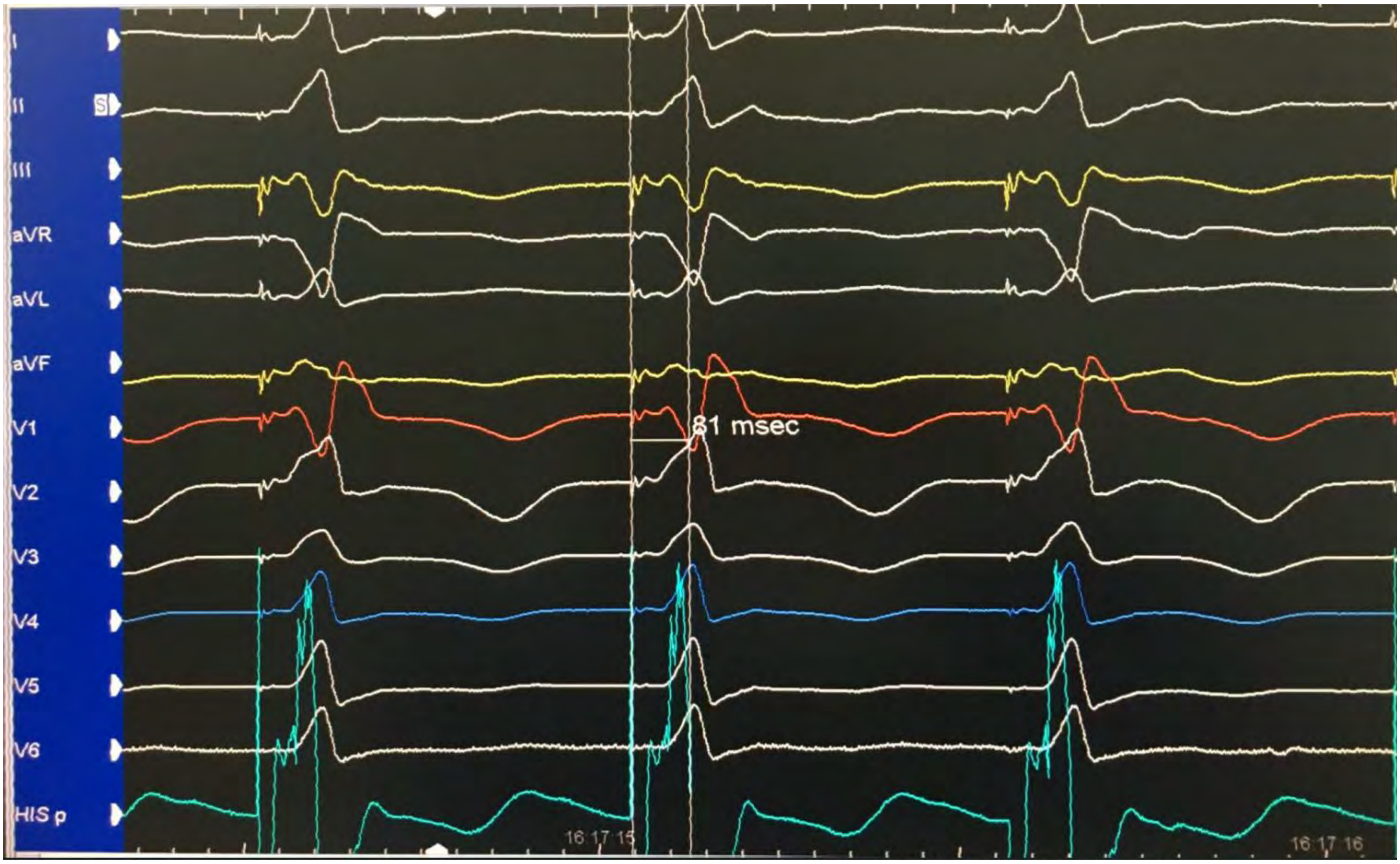

Transitions

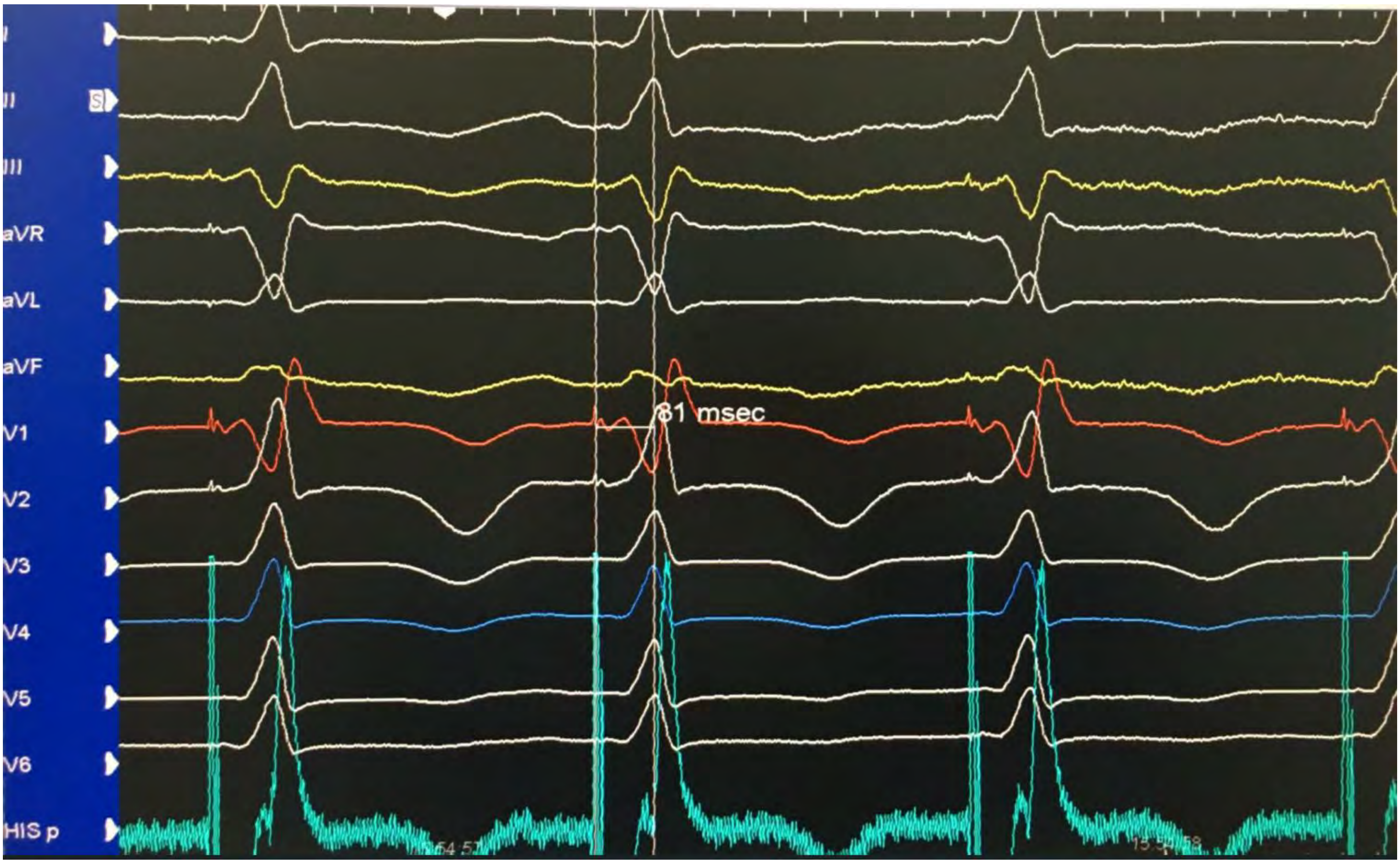

Patient 3:

Pre-ECG

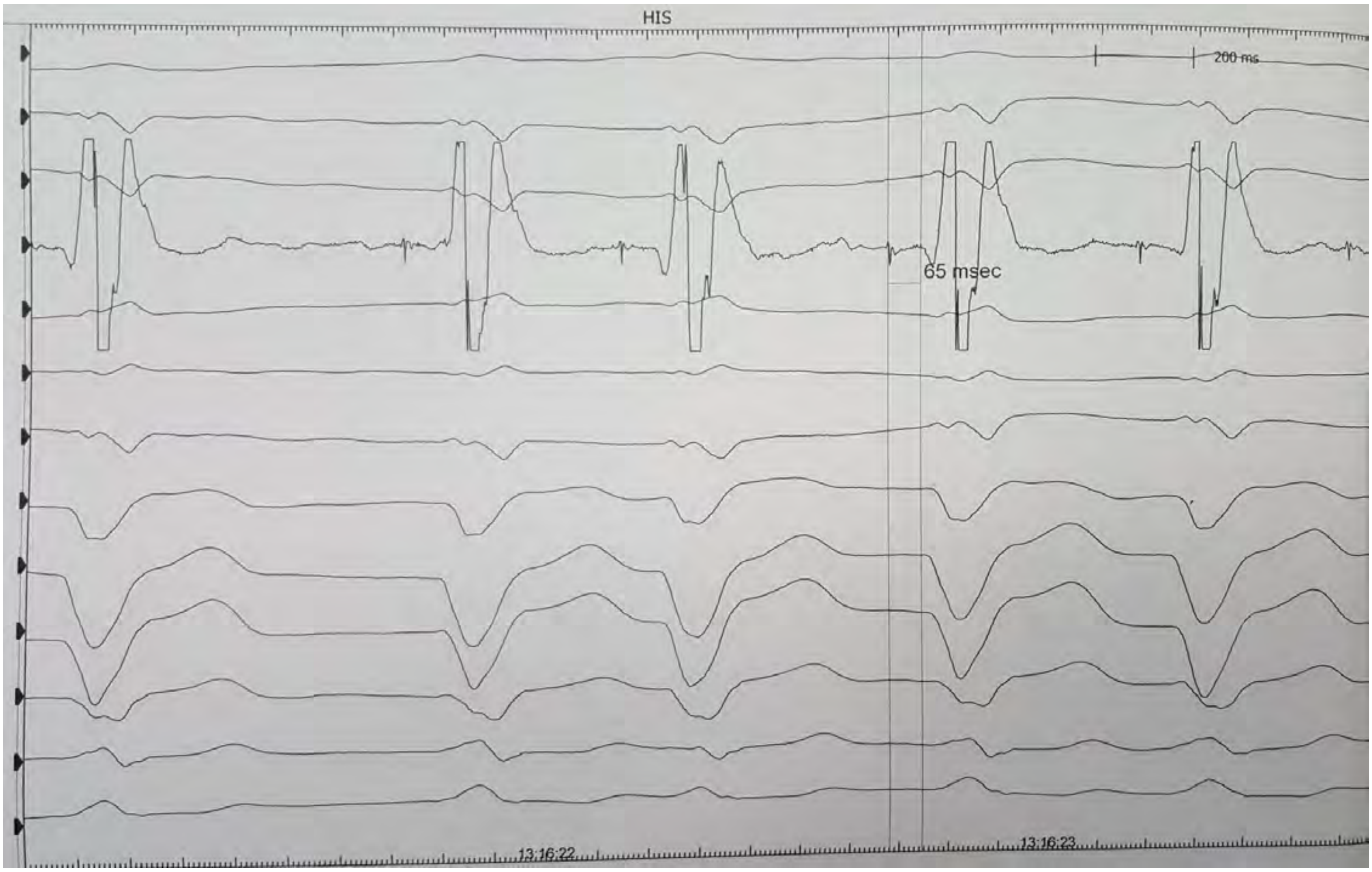

Transitions

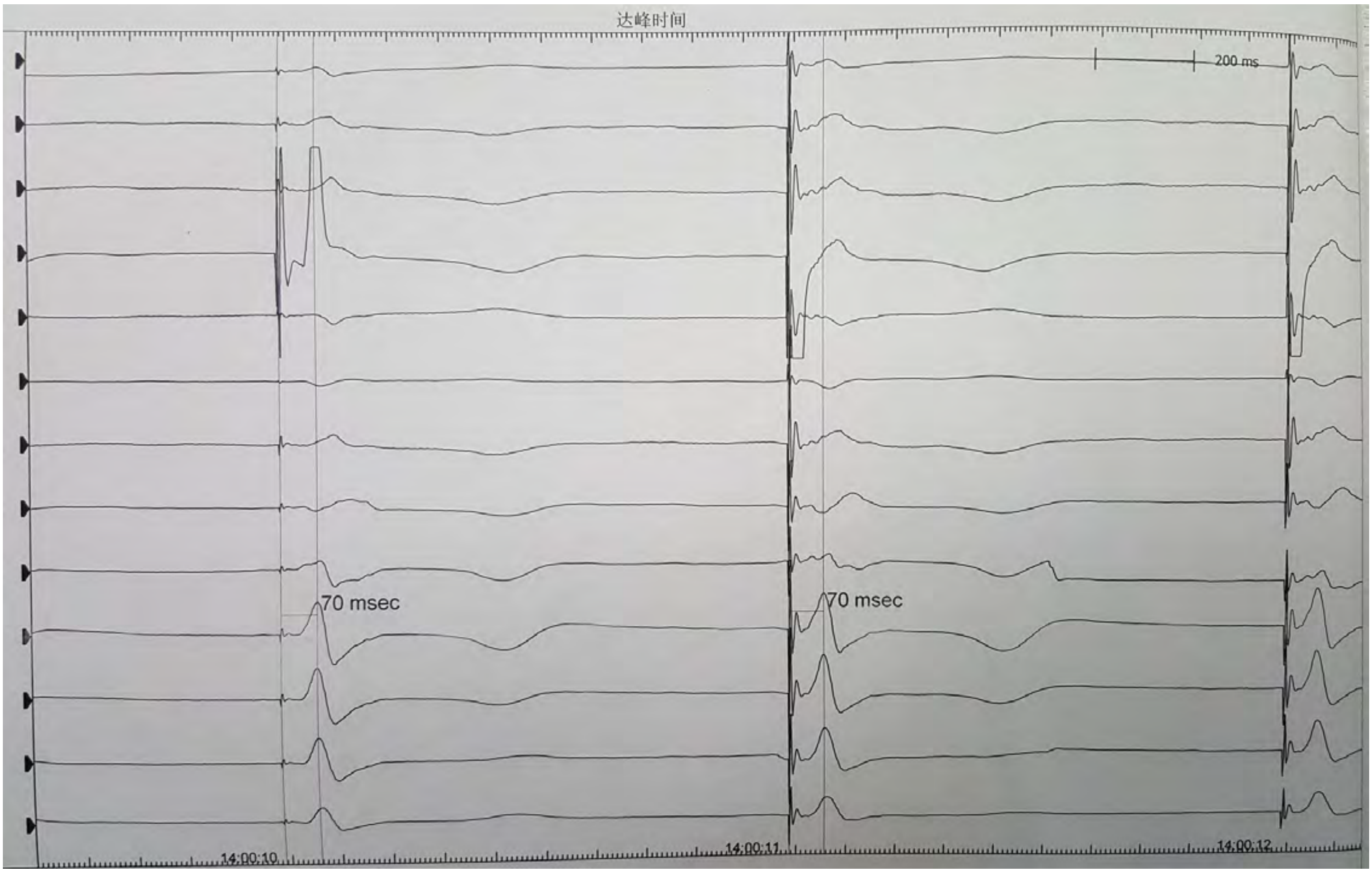

Patient 4:
Pre-ECG

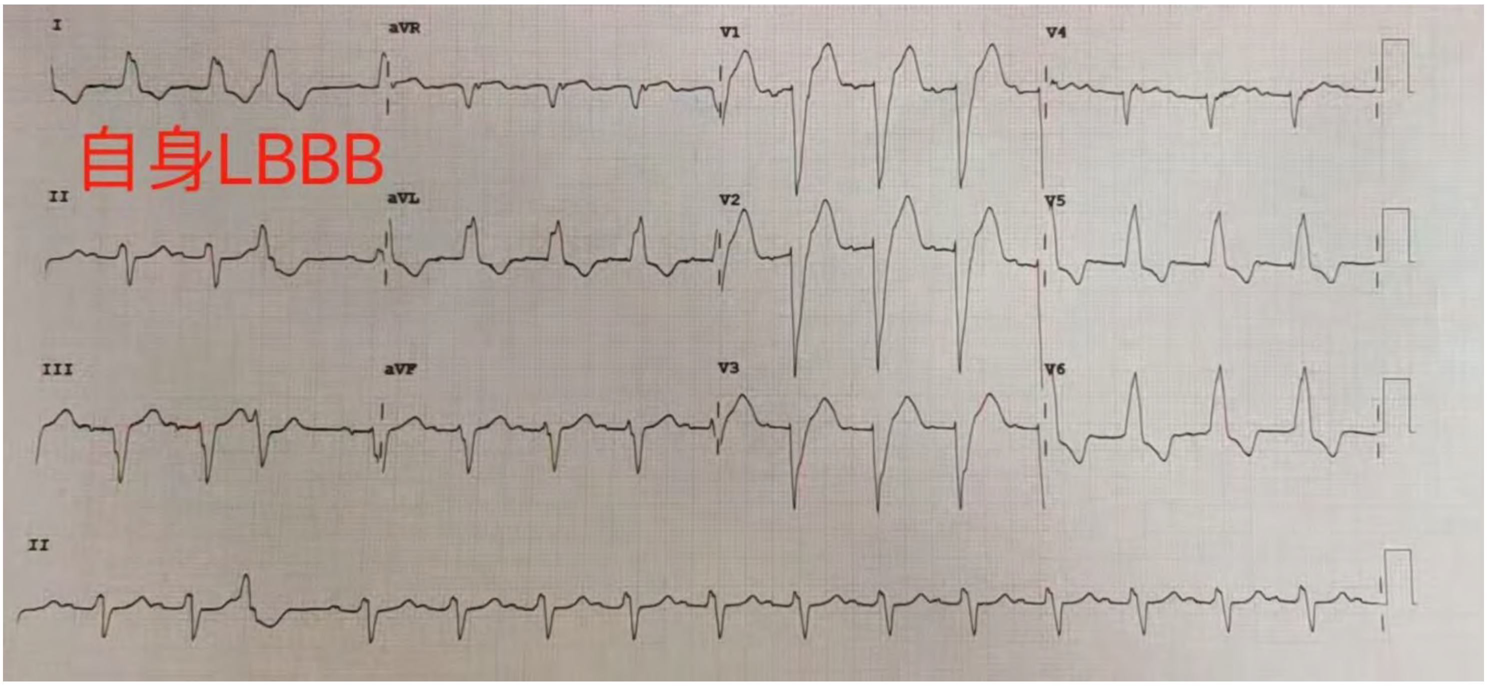

Post ECG

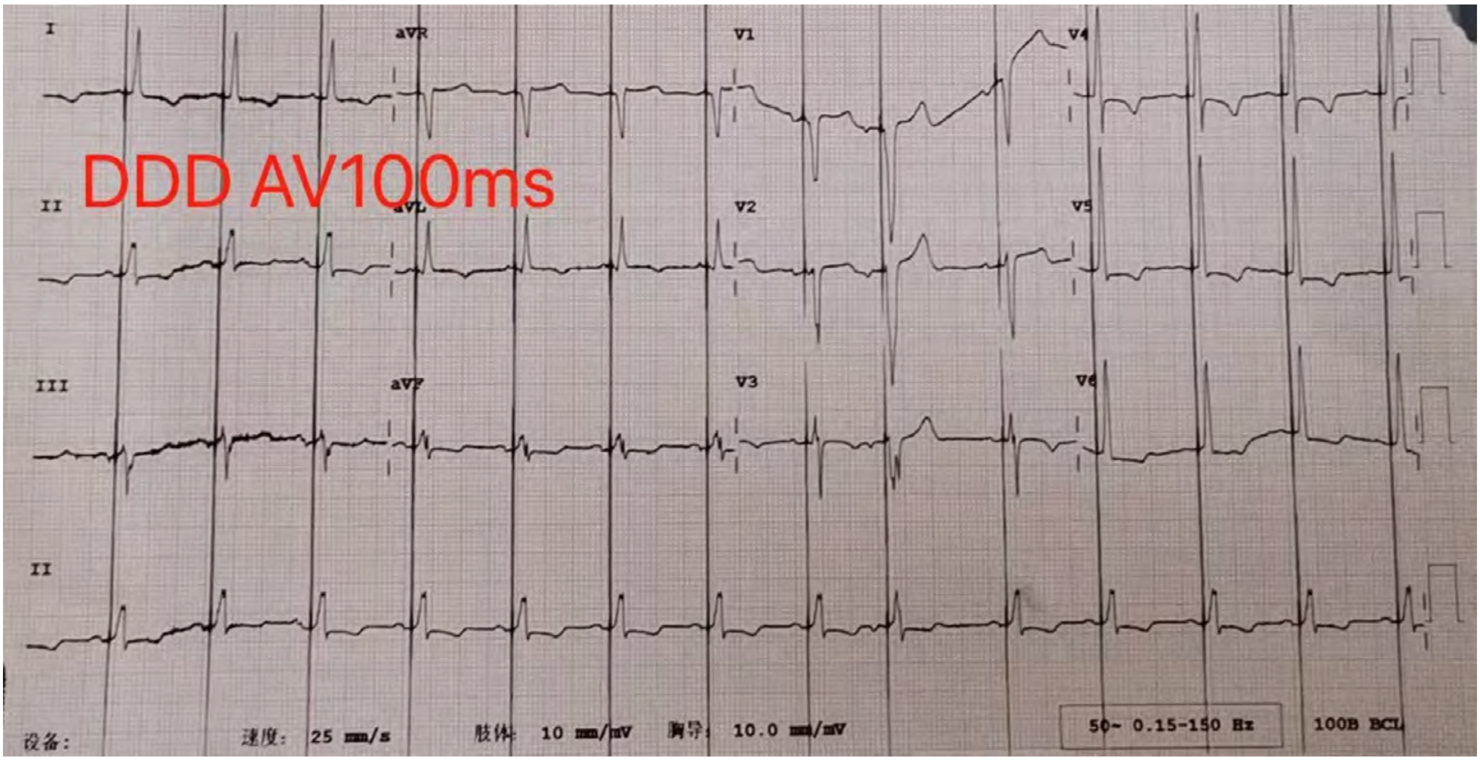

Patient 4:
Transitions

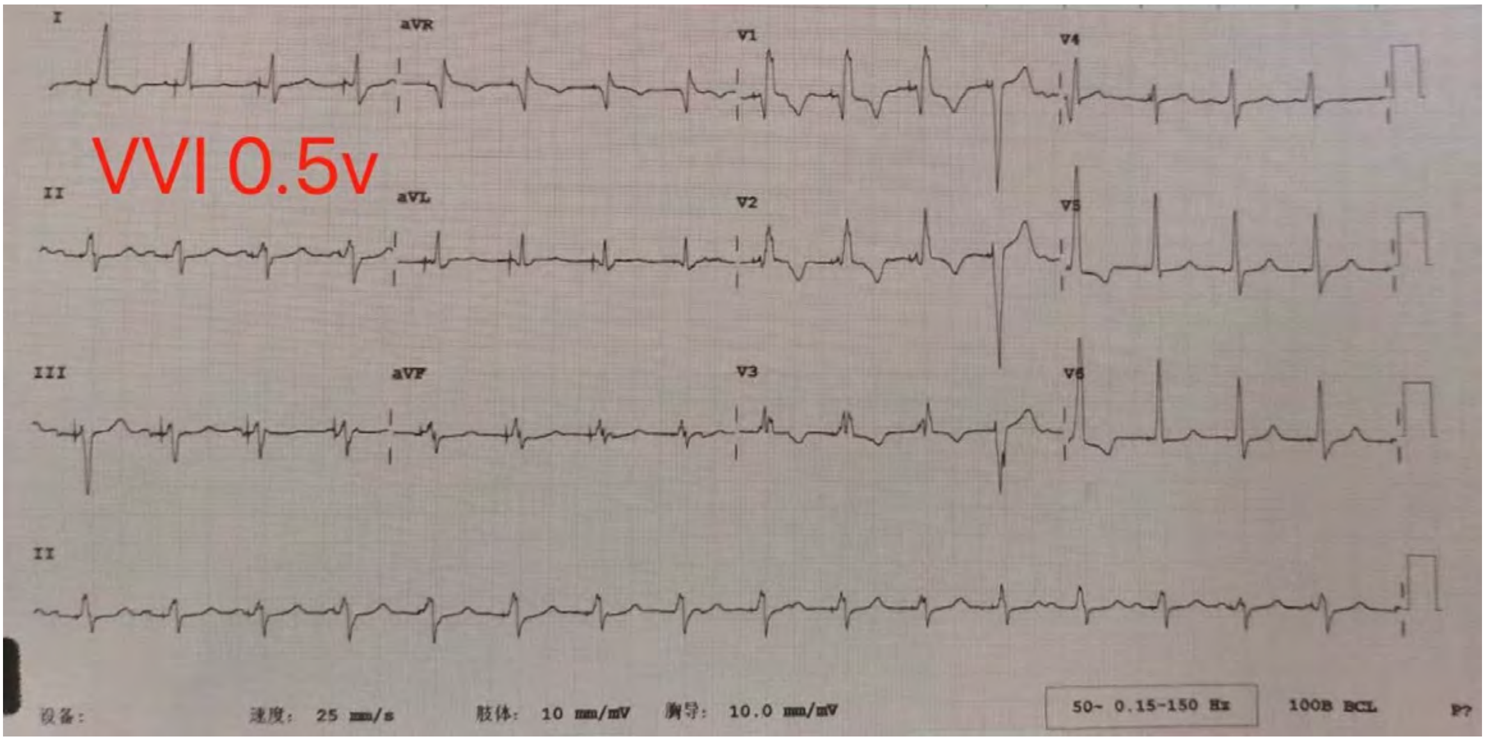

Transitions

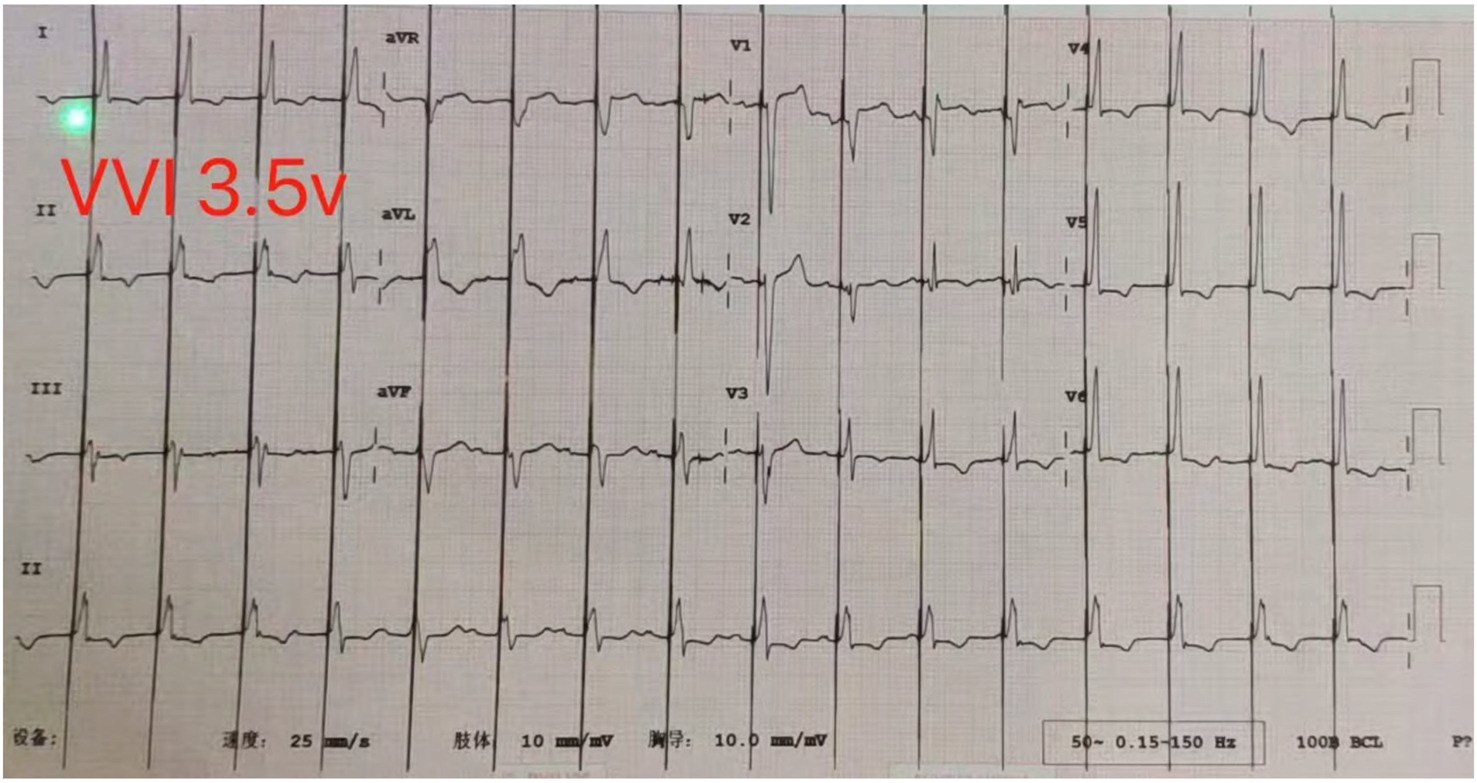

Patient 5:
Pre-ECG

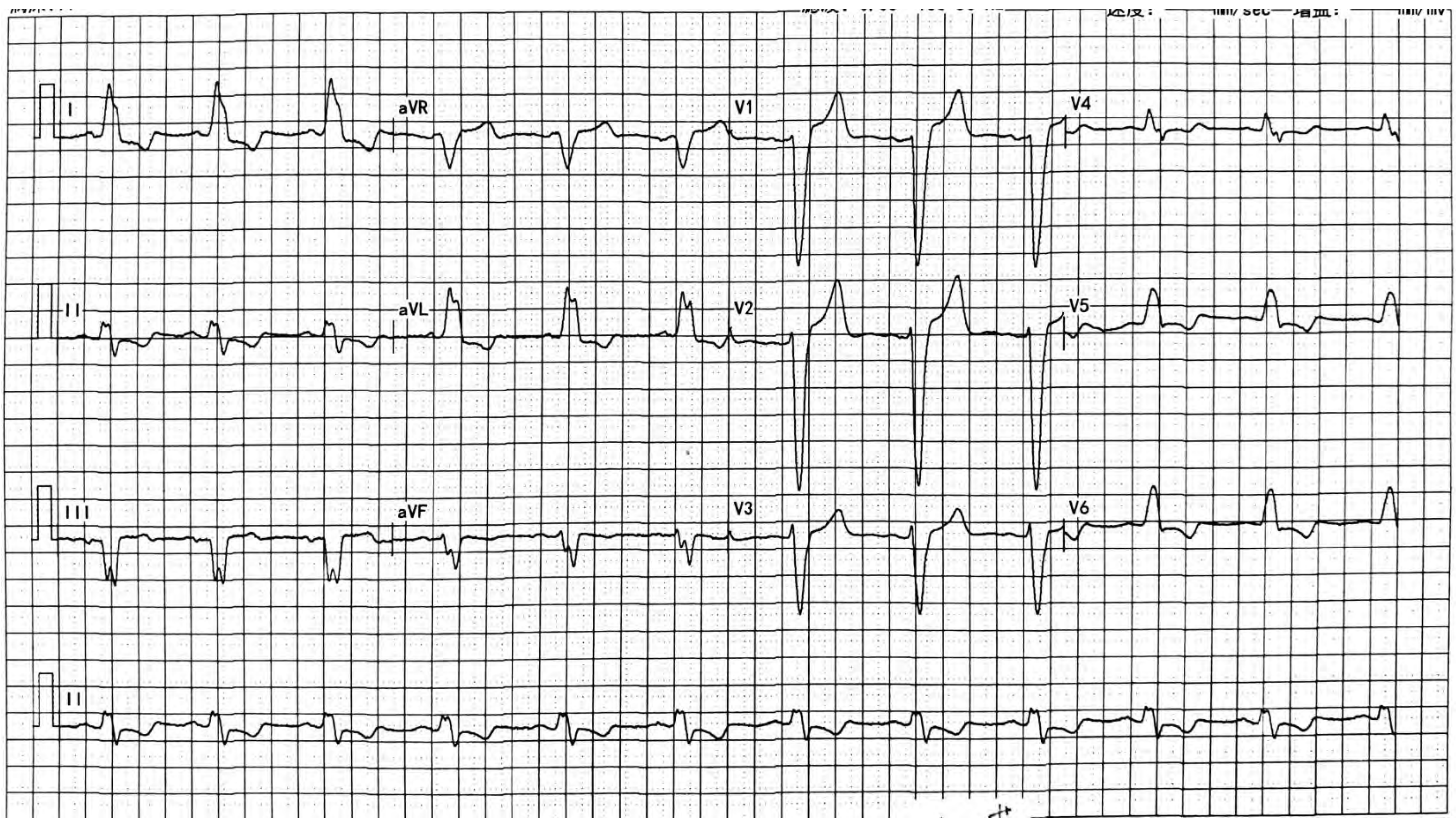

Post ECG

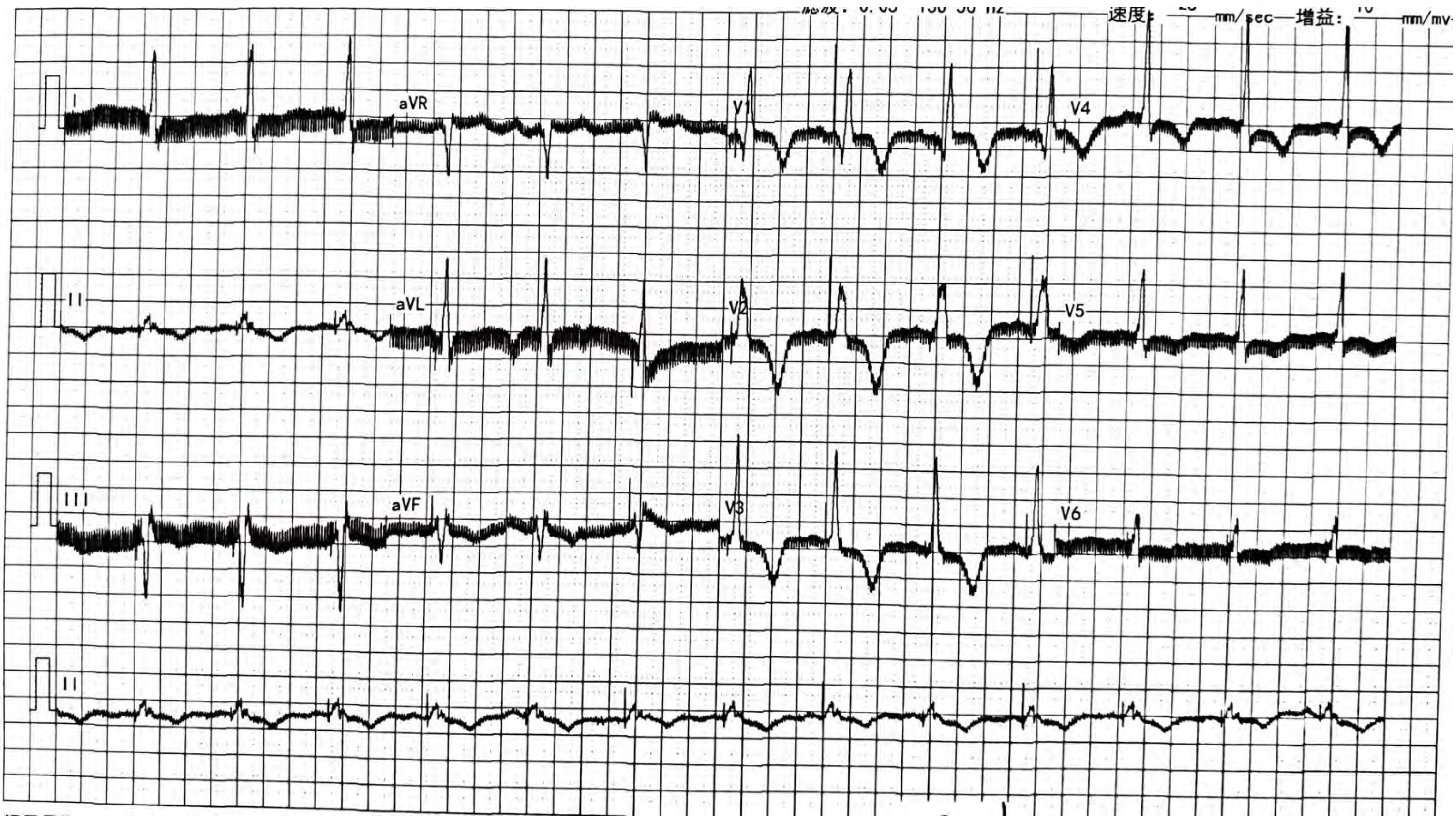

Patient 6:
Pre-ECG

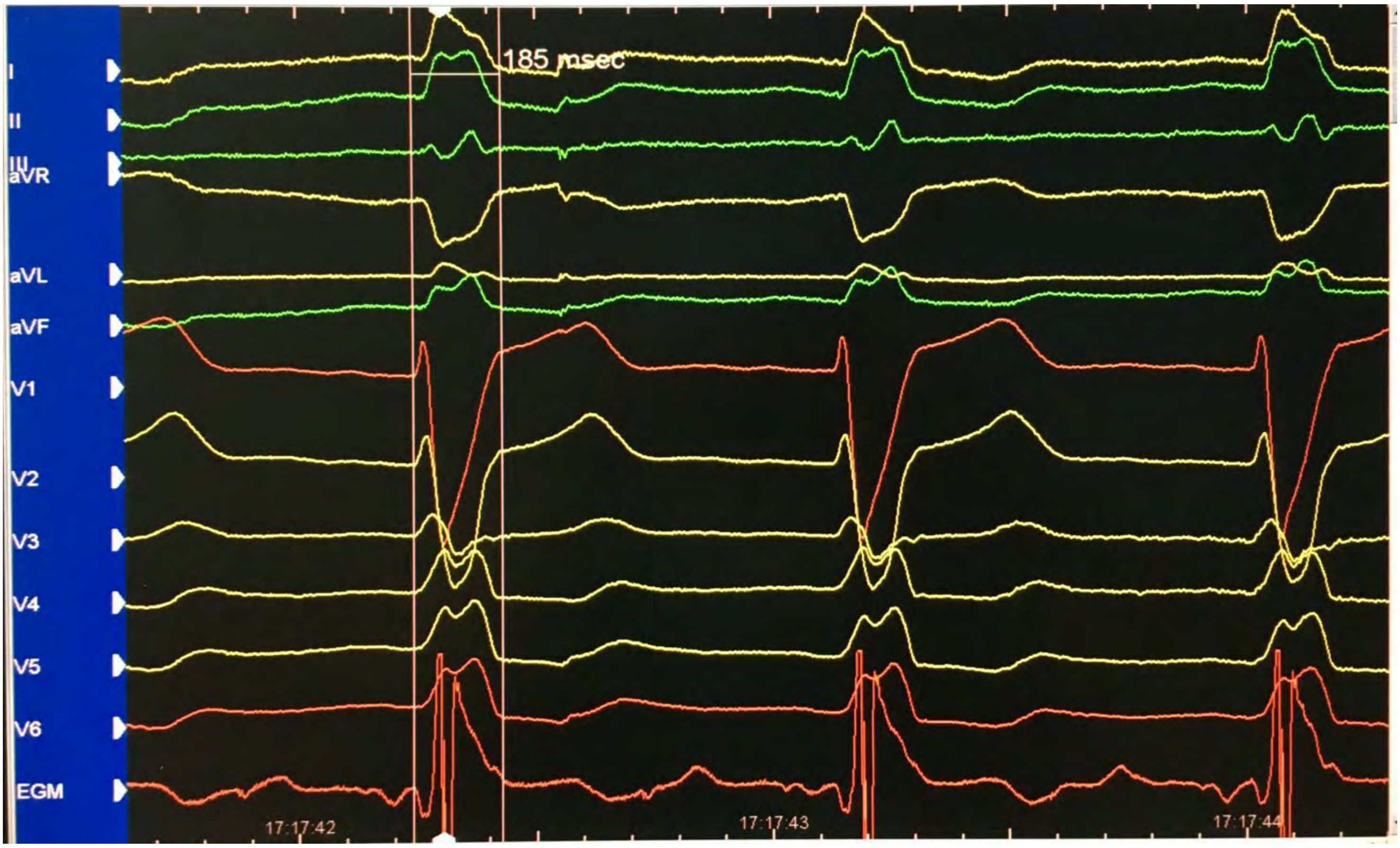

Transitions

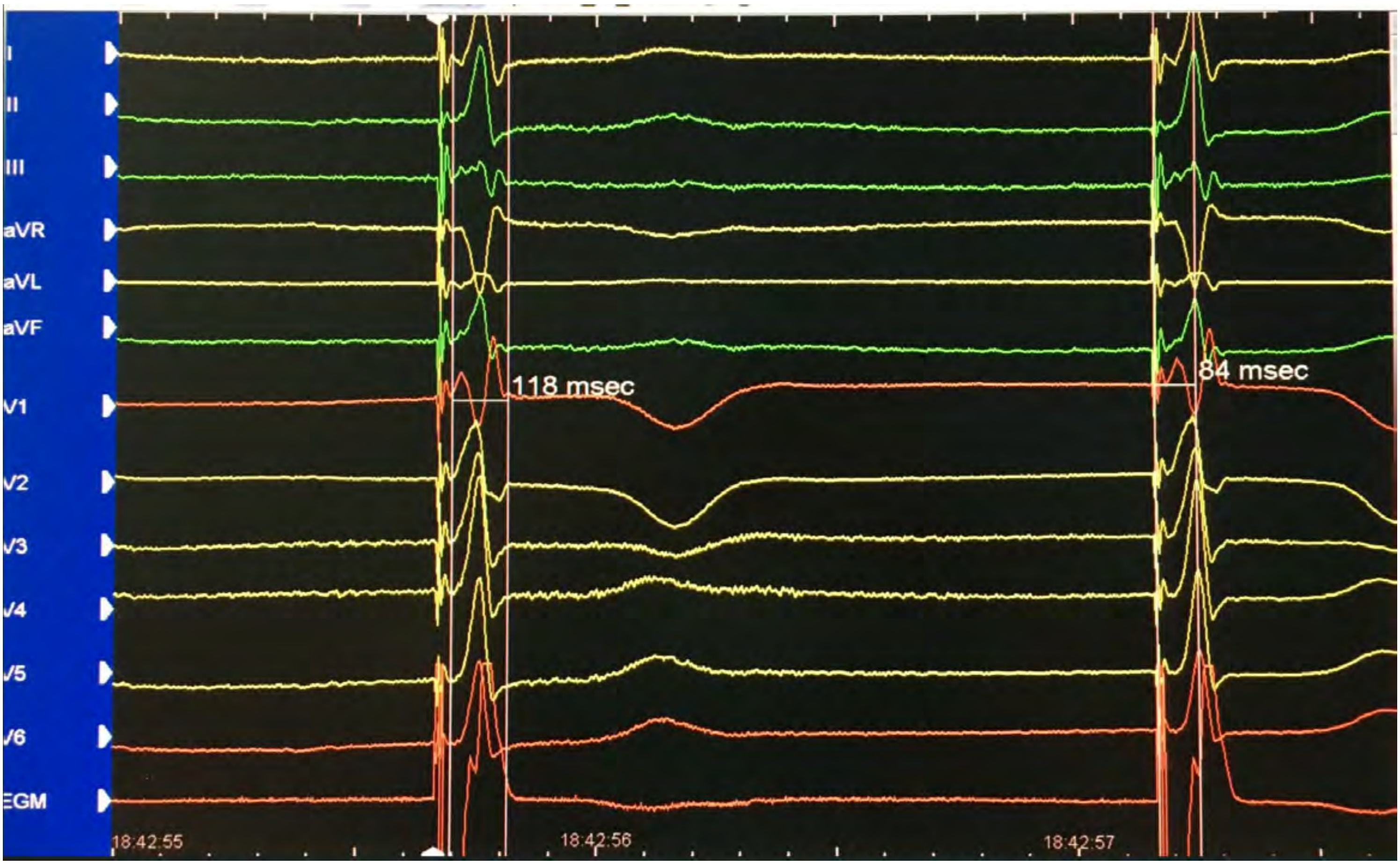

Patient 7:
Pre-ECG

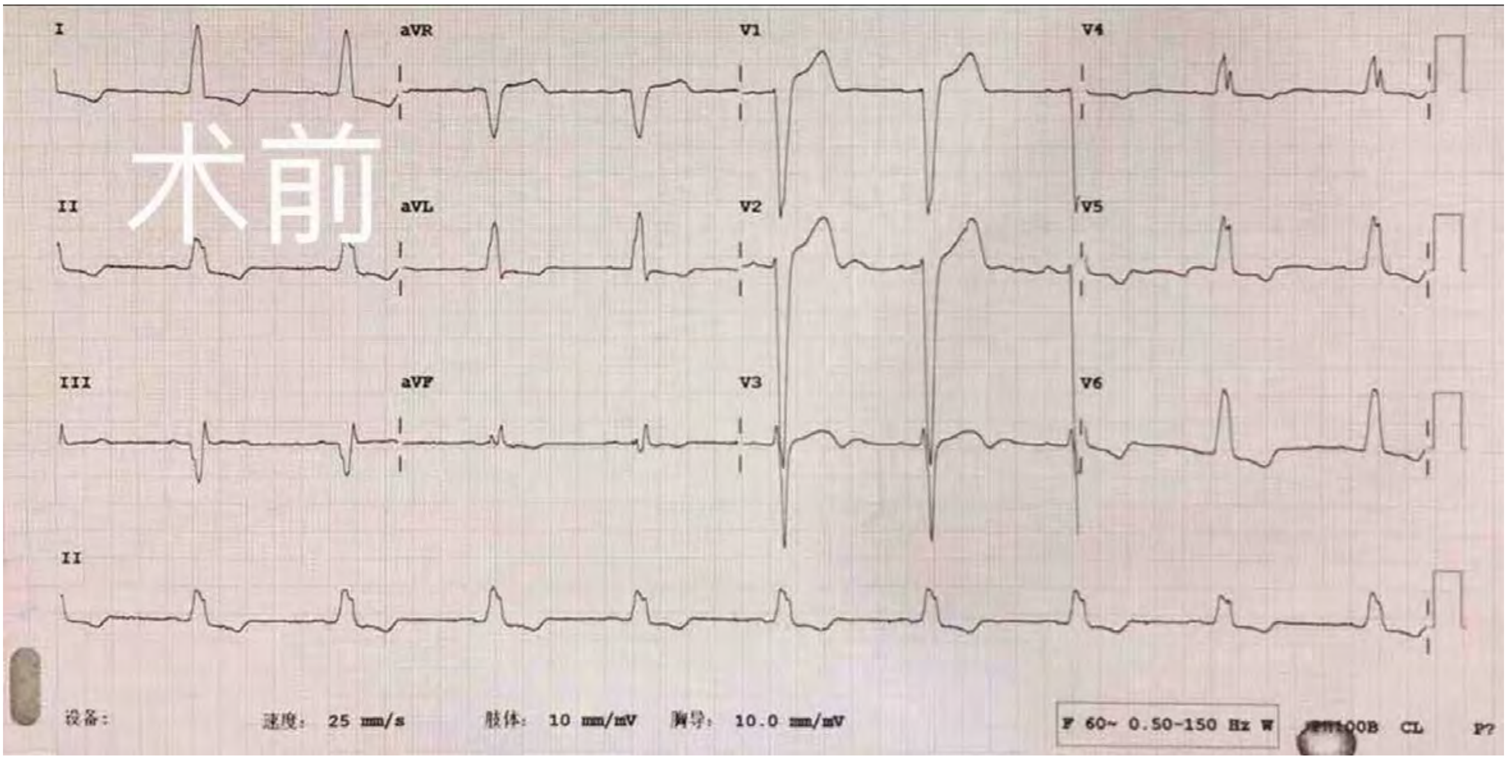

Post ECG

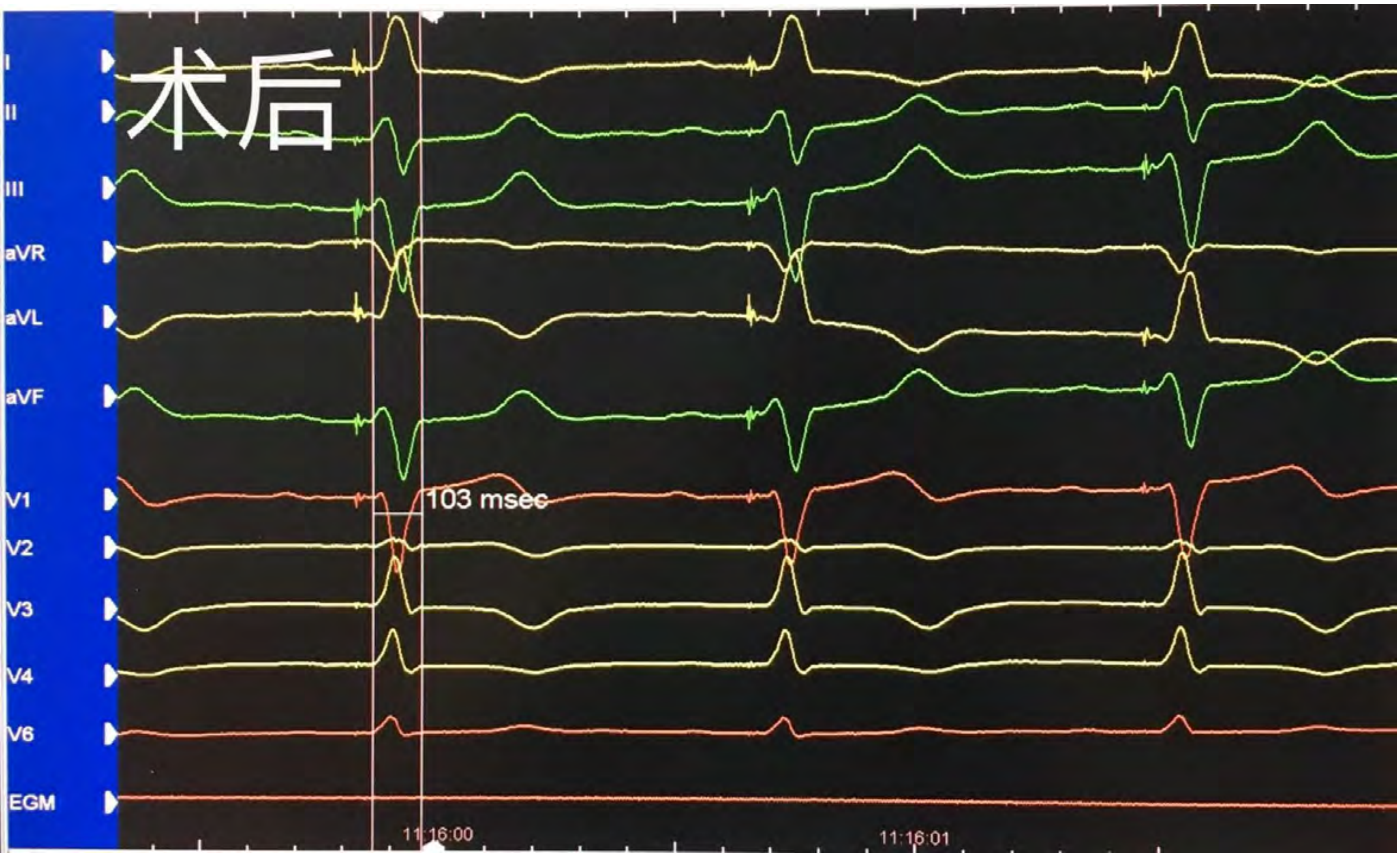

Patient 7:
Transitions

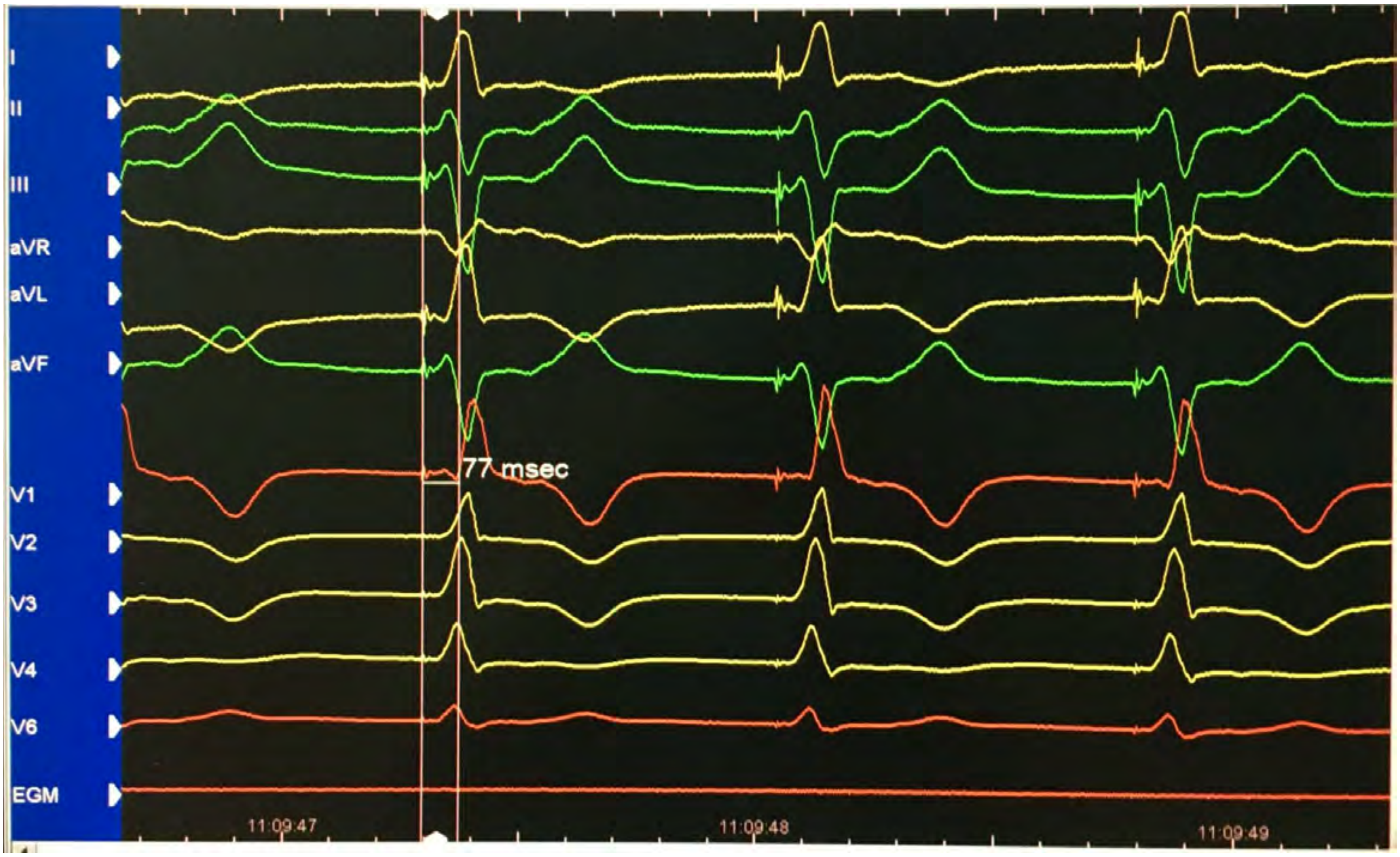

Transitions

Patient 8:
Pre-ECG

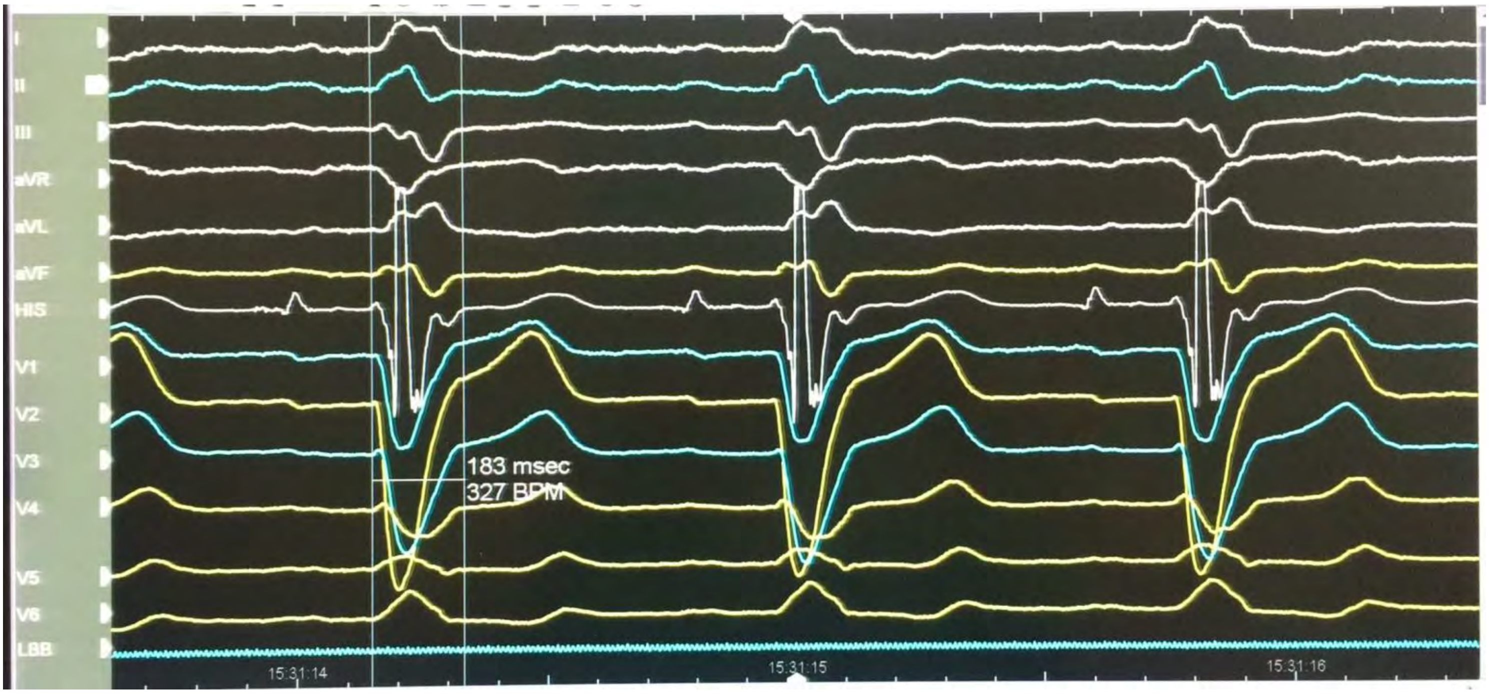

Post ECG

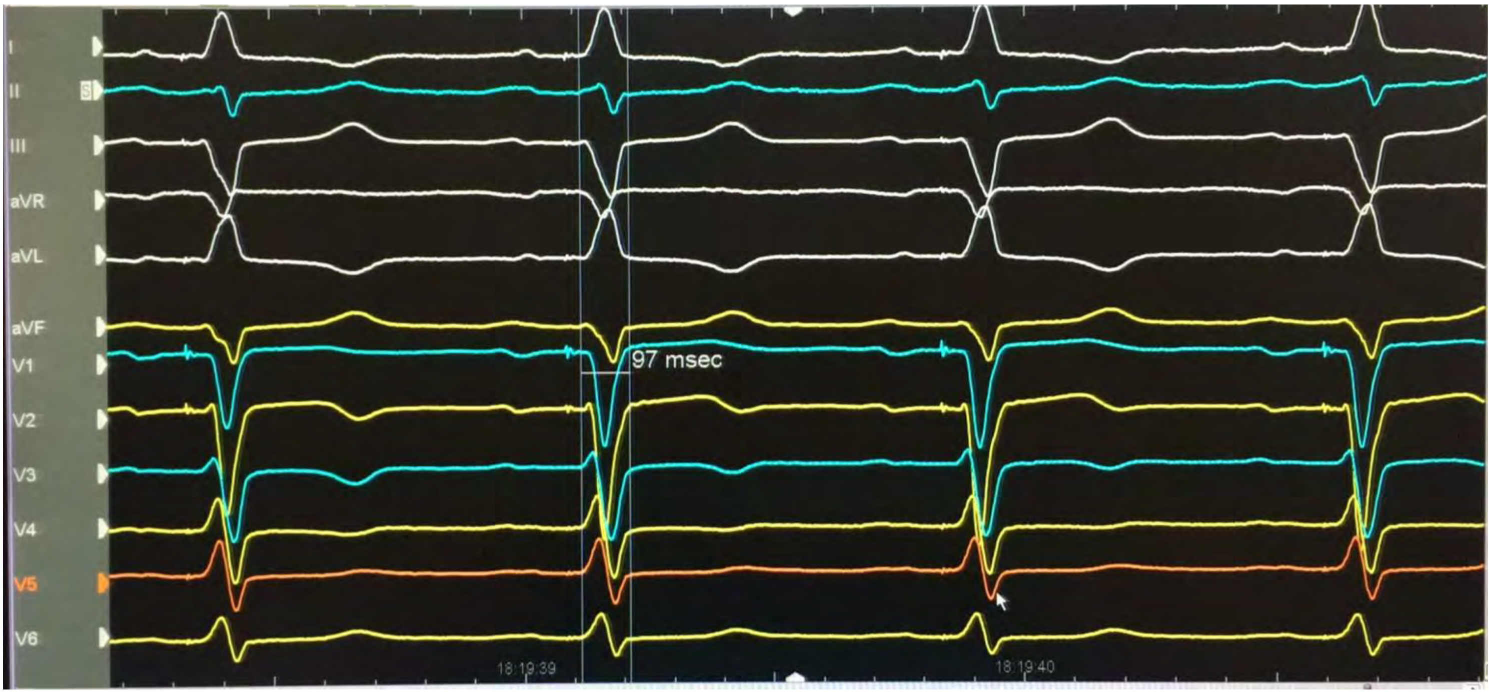

Patient 8:
Transitions

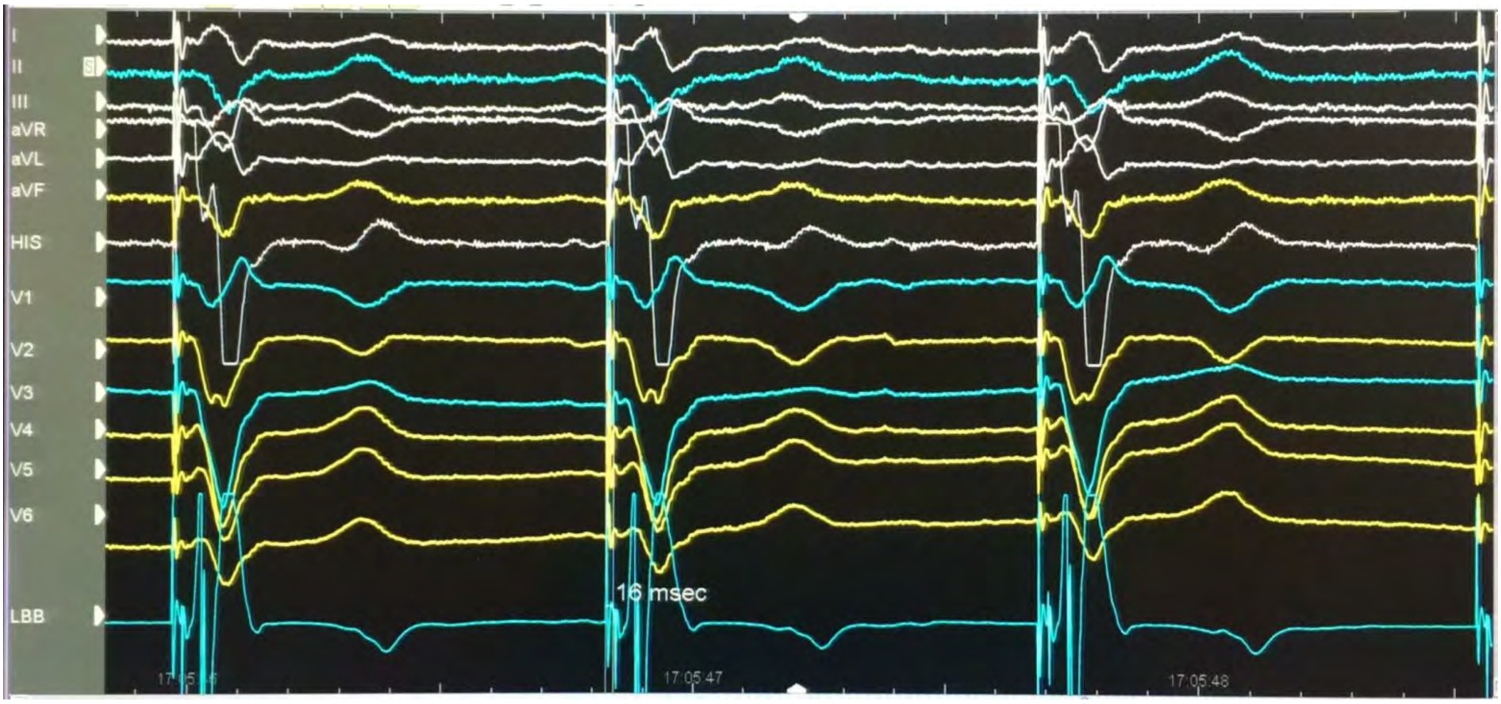

Transitions

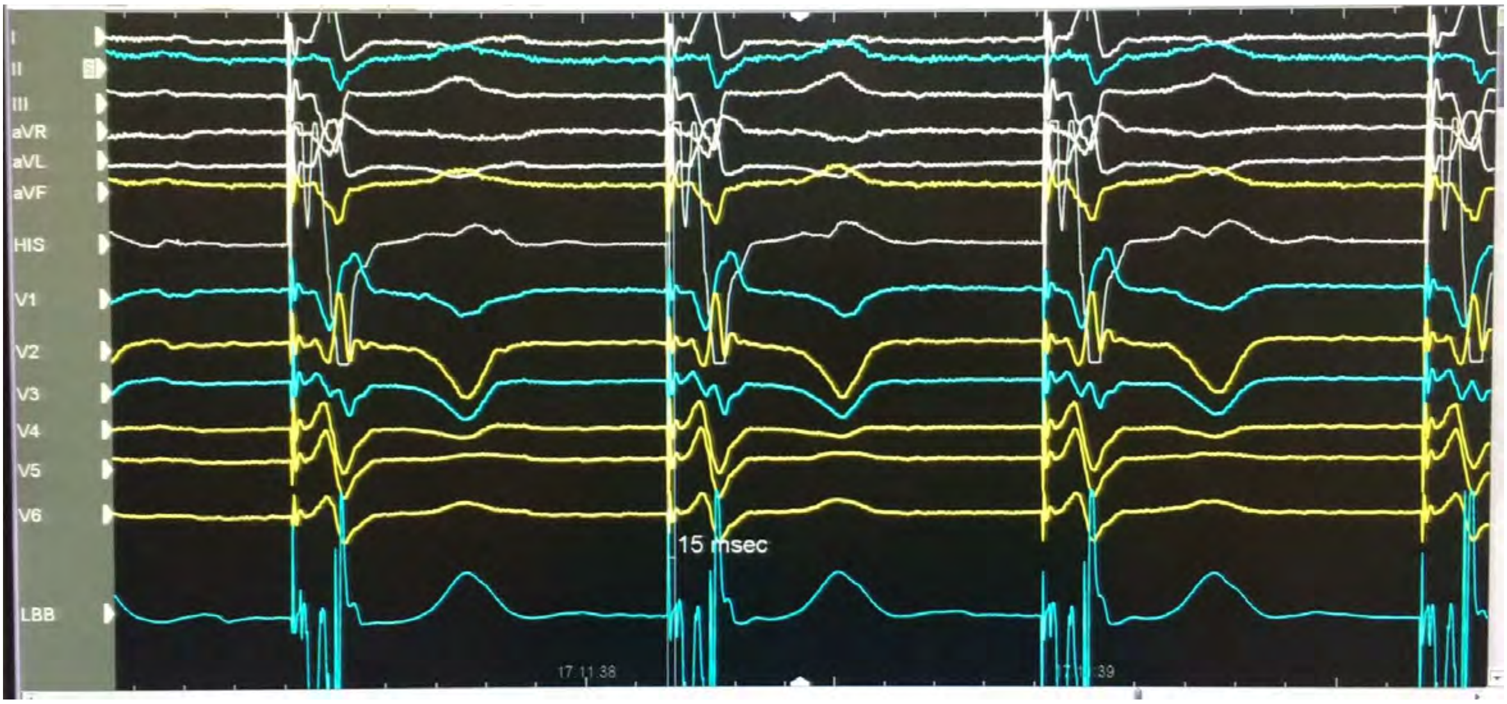

Patient 9:

Pre-ECG

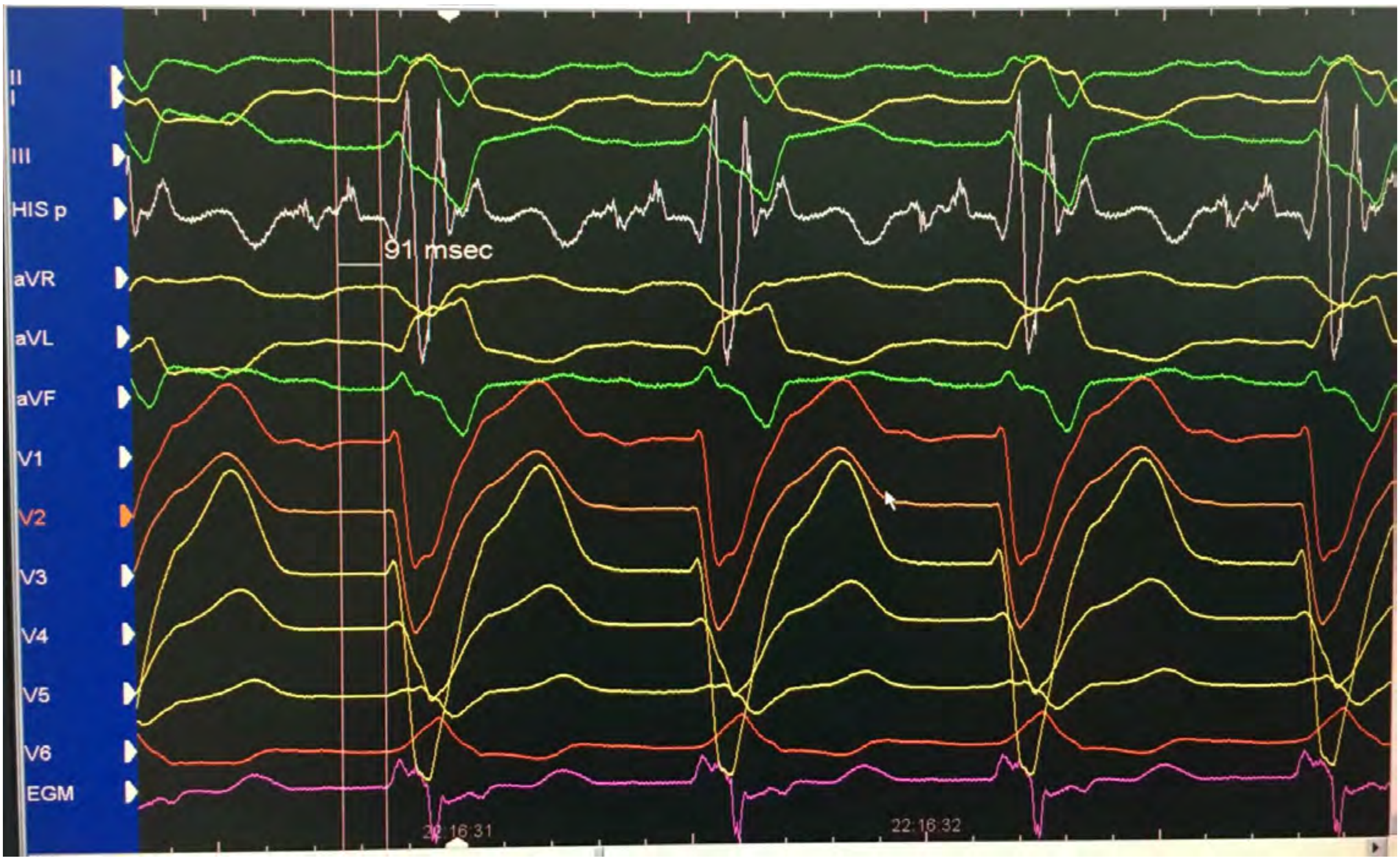

Post ECG

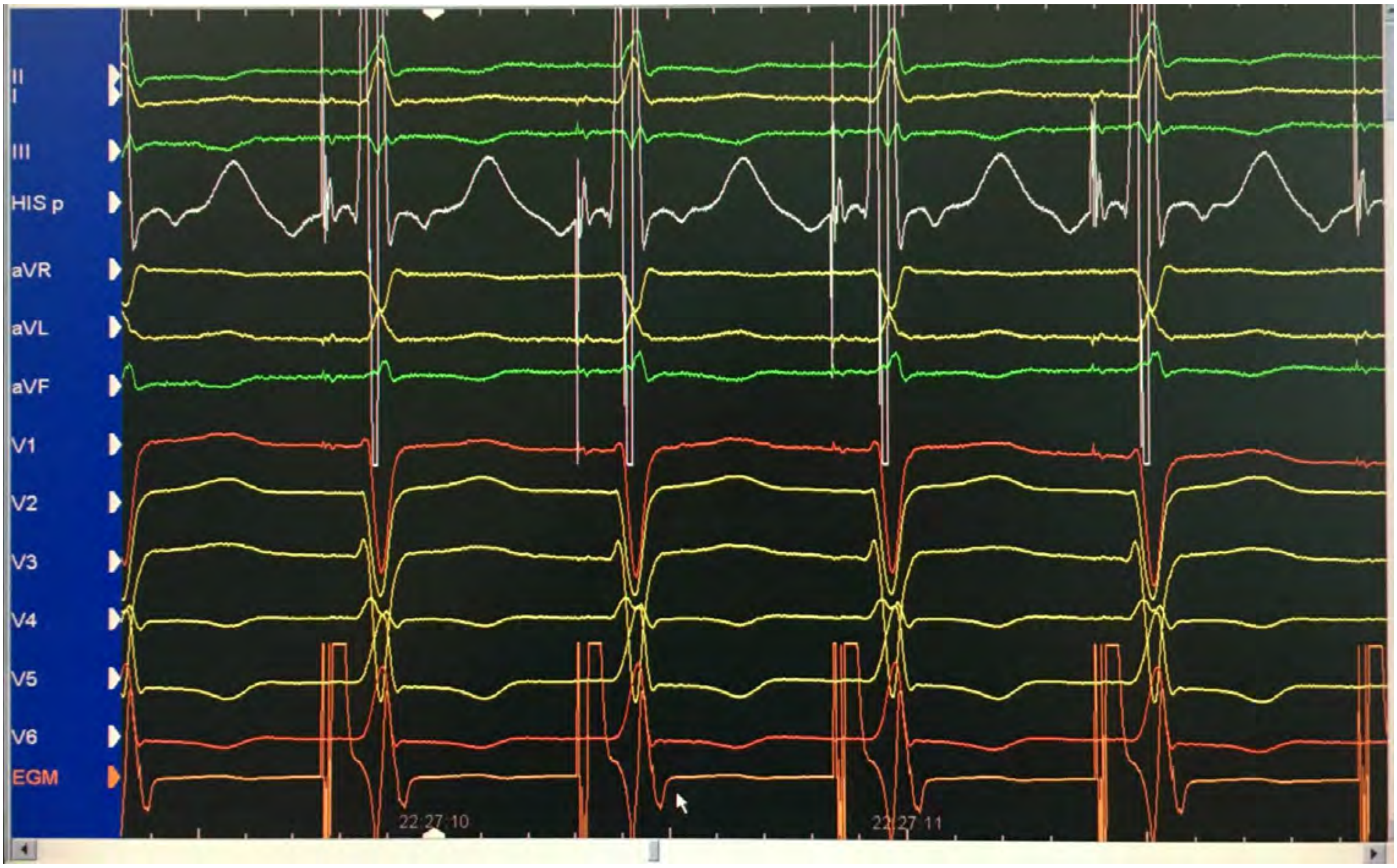

Patient 9:

Transitions

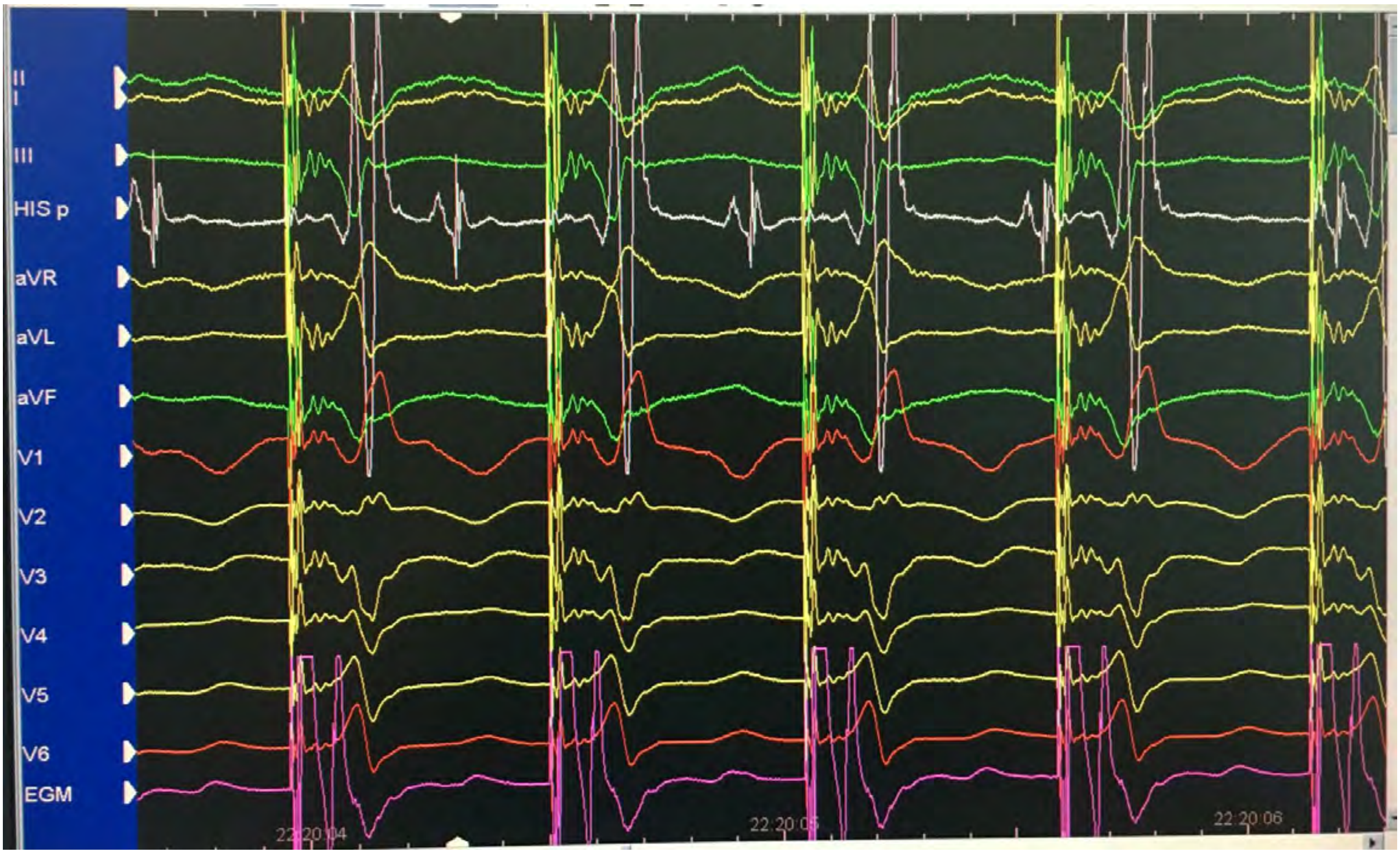

Transitions

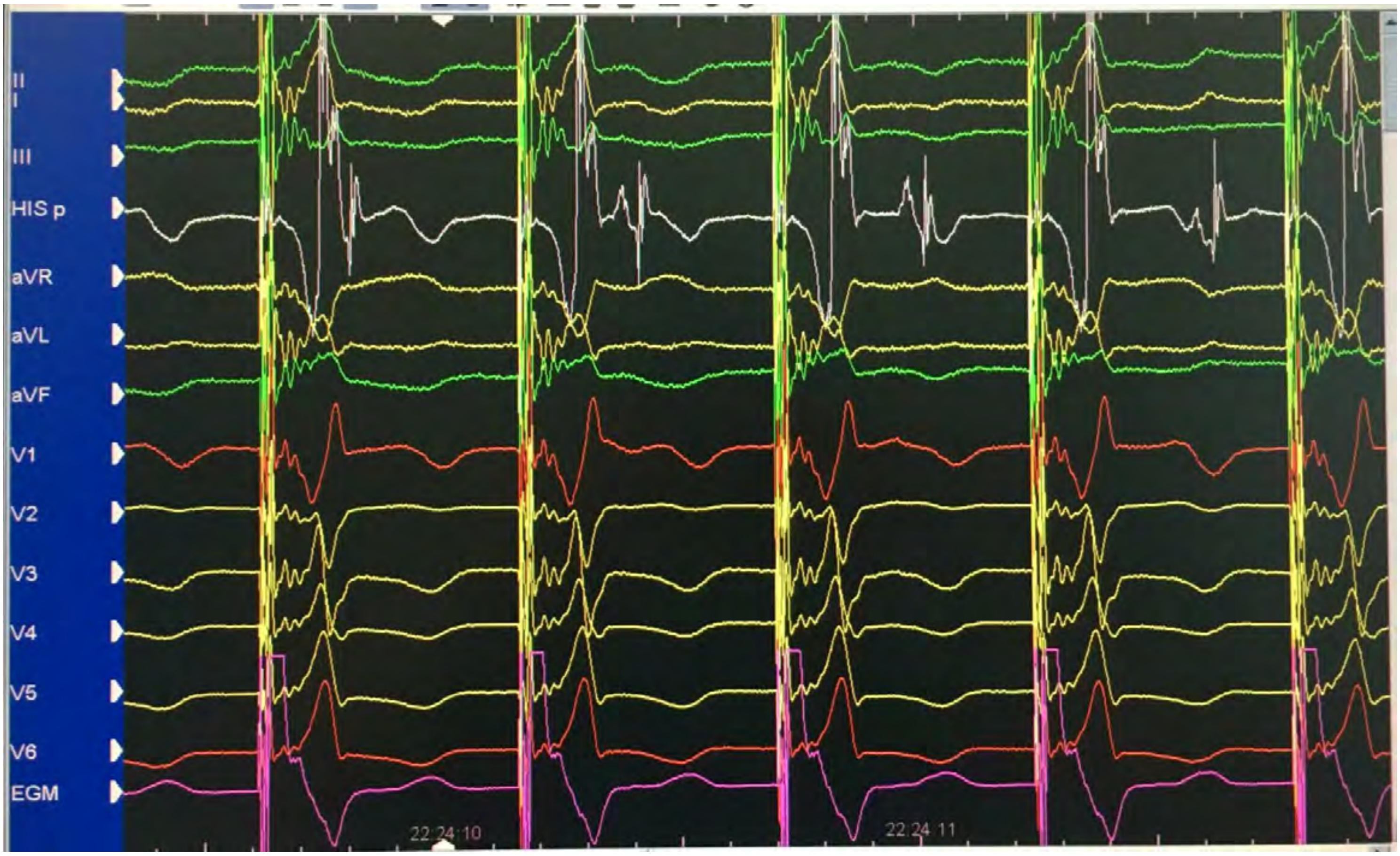

Patient 10:
Pre-ECG

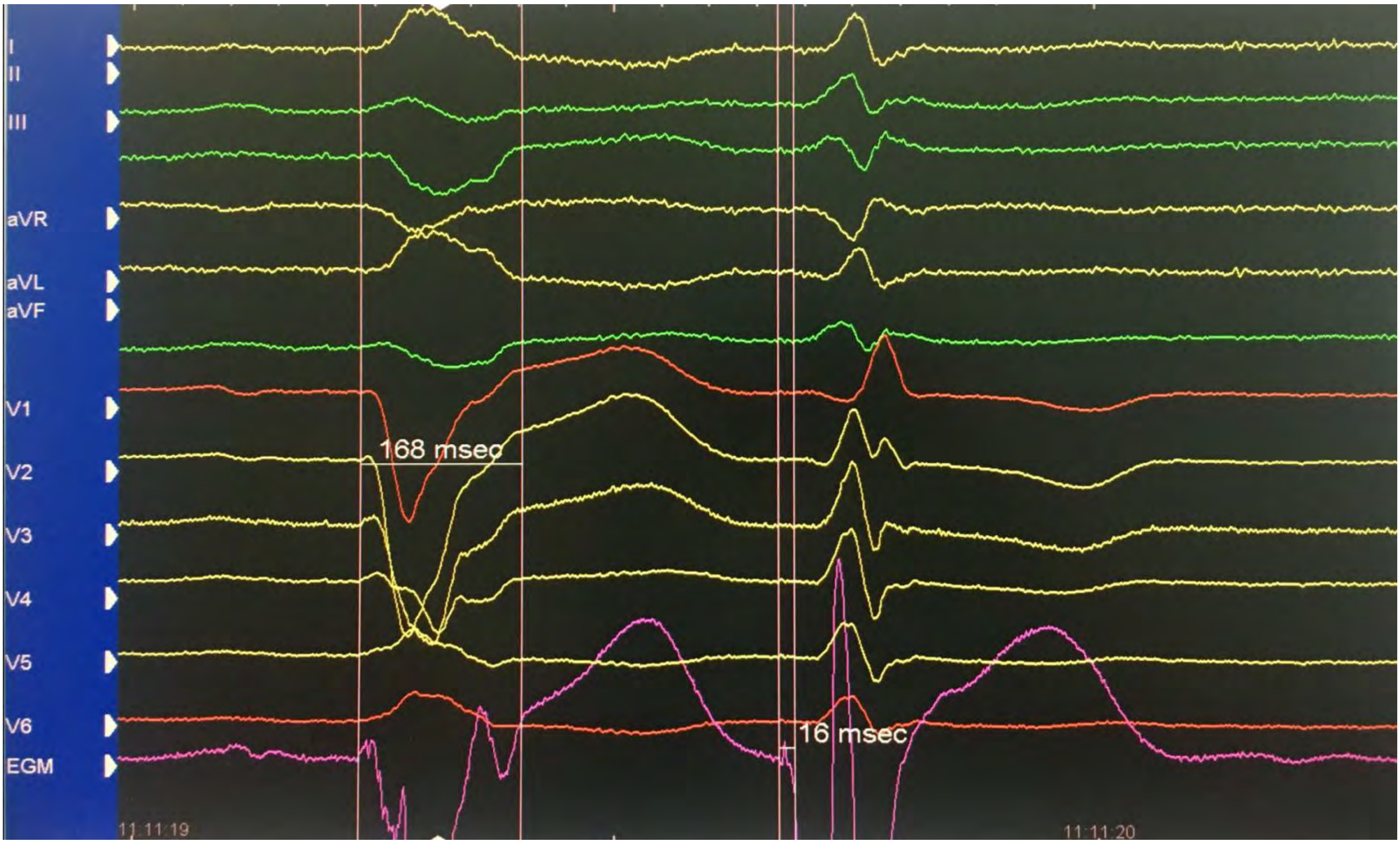

Post ECG

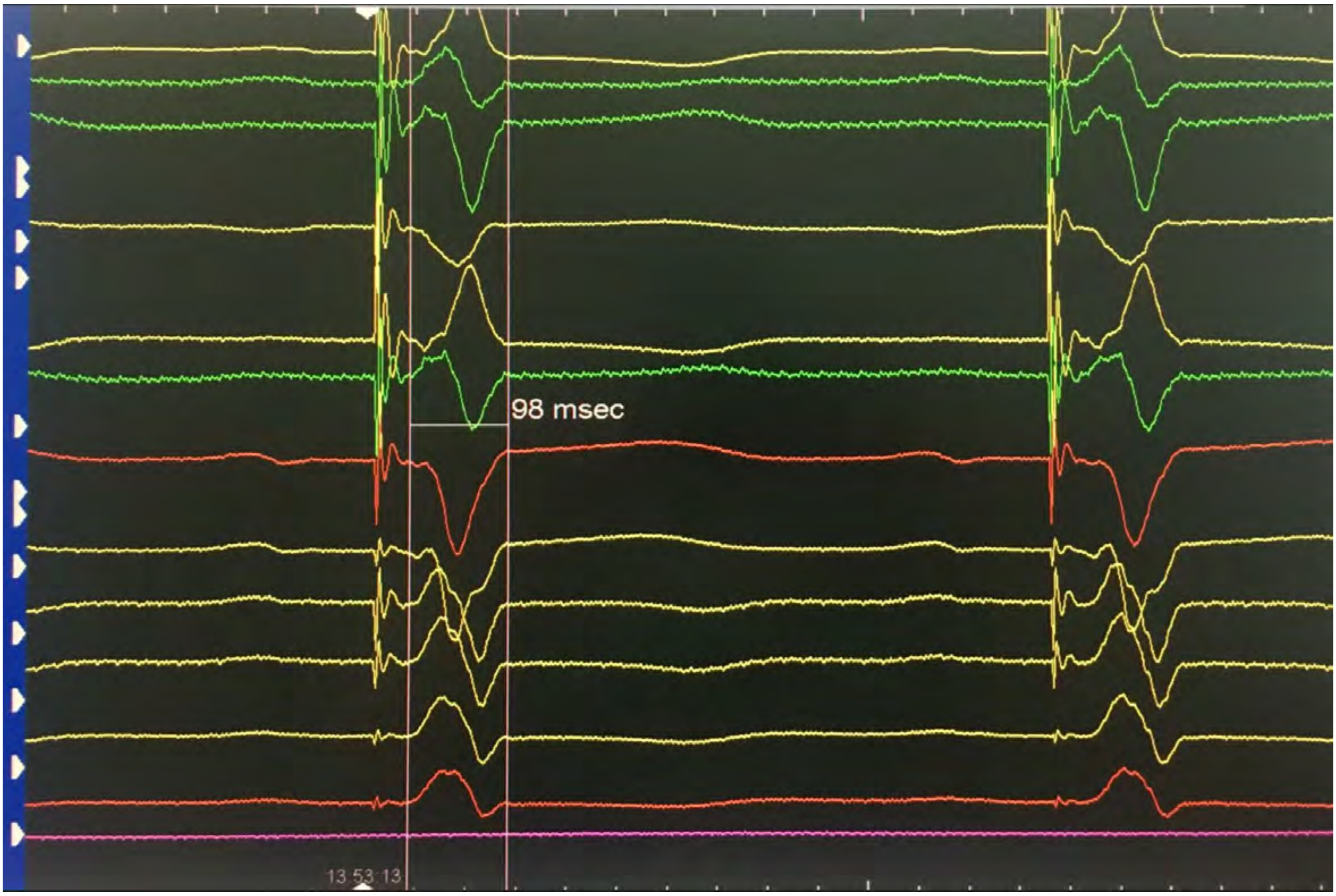

Patient 10:
Transitions

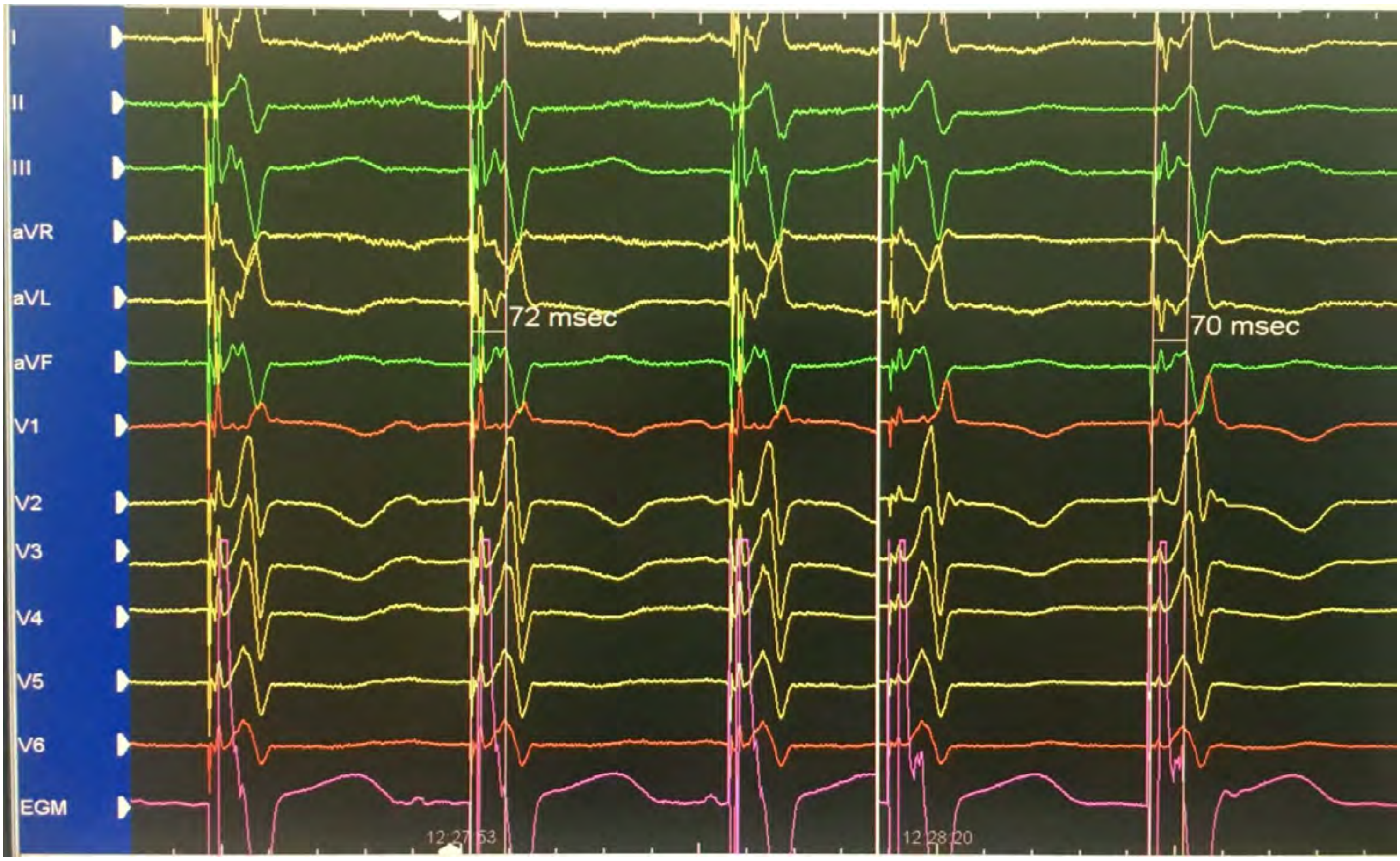

Transitions

Patient 11:

Pre-ECG

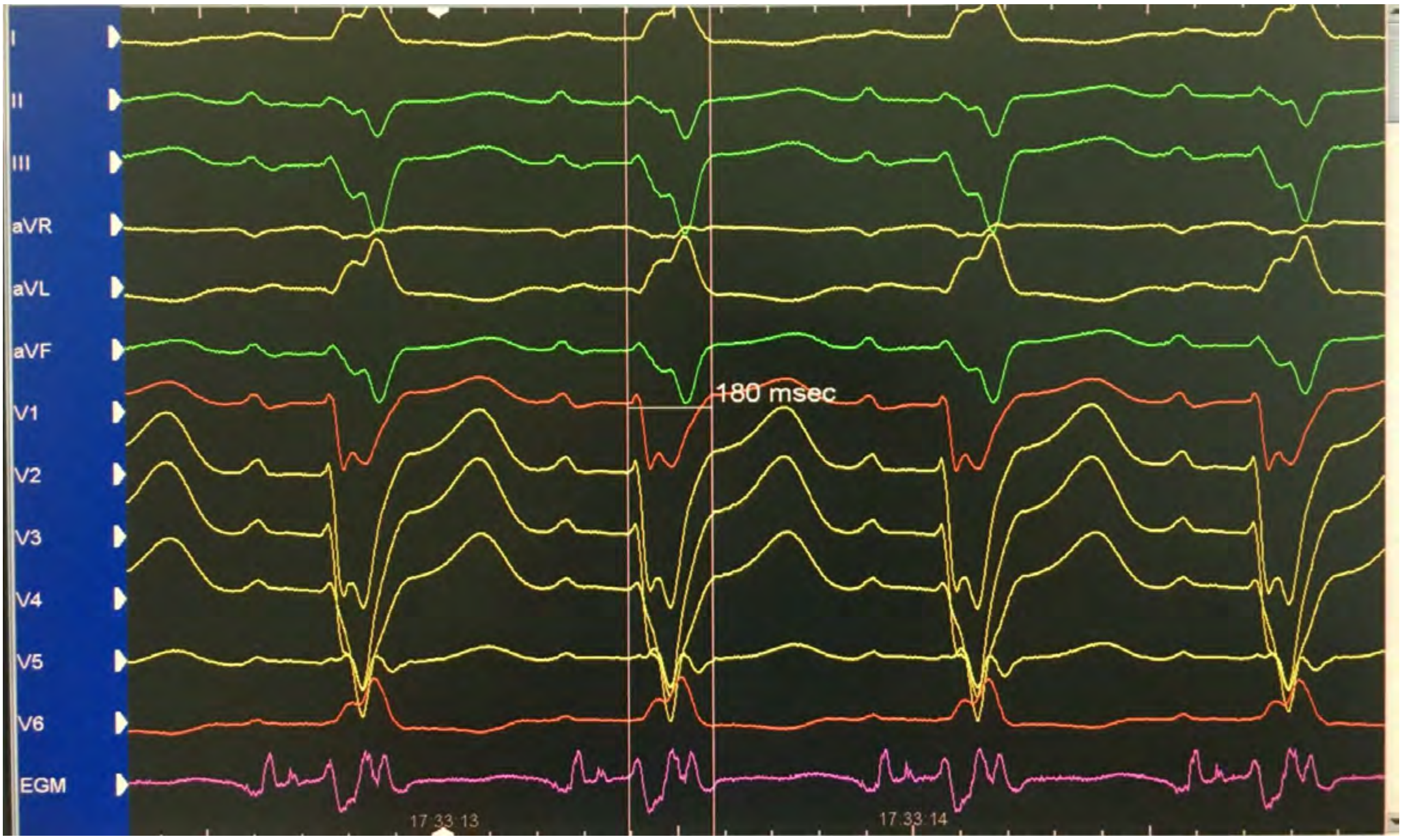

Post ECG

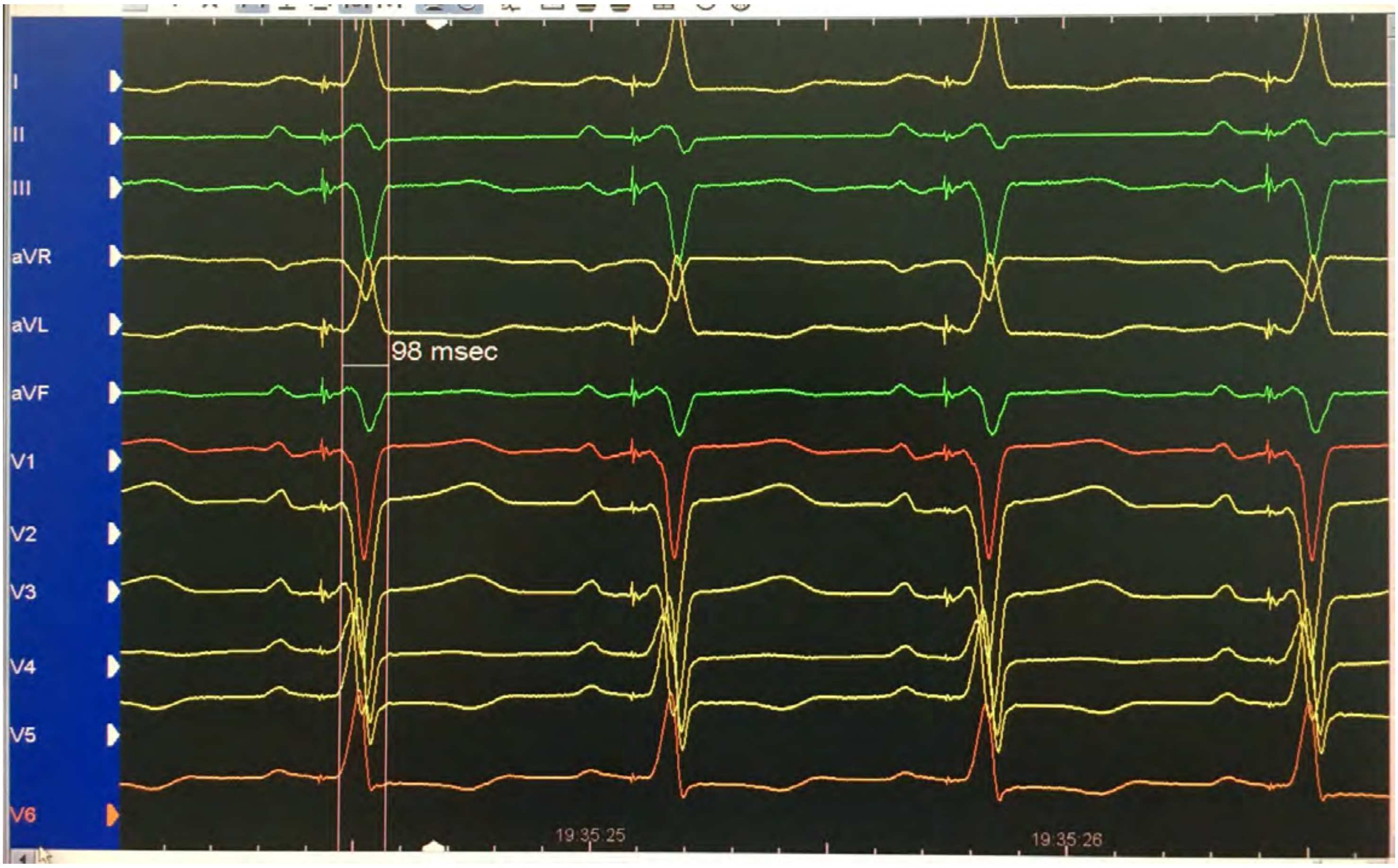

Patient 11:
Transitions

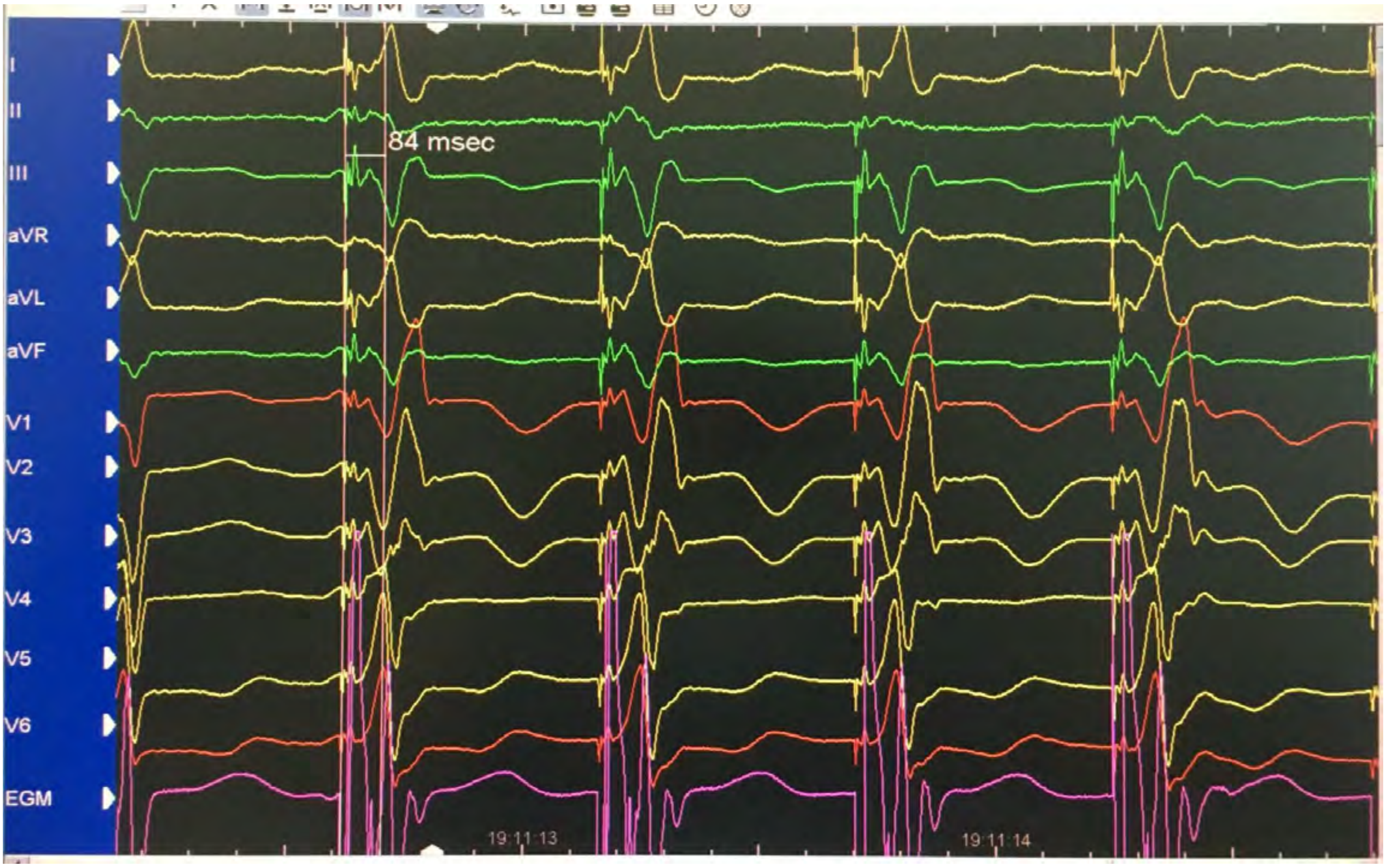

Transitions

Patient 12:
Pre-ECG

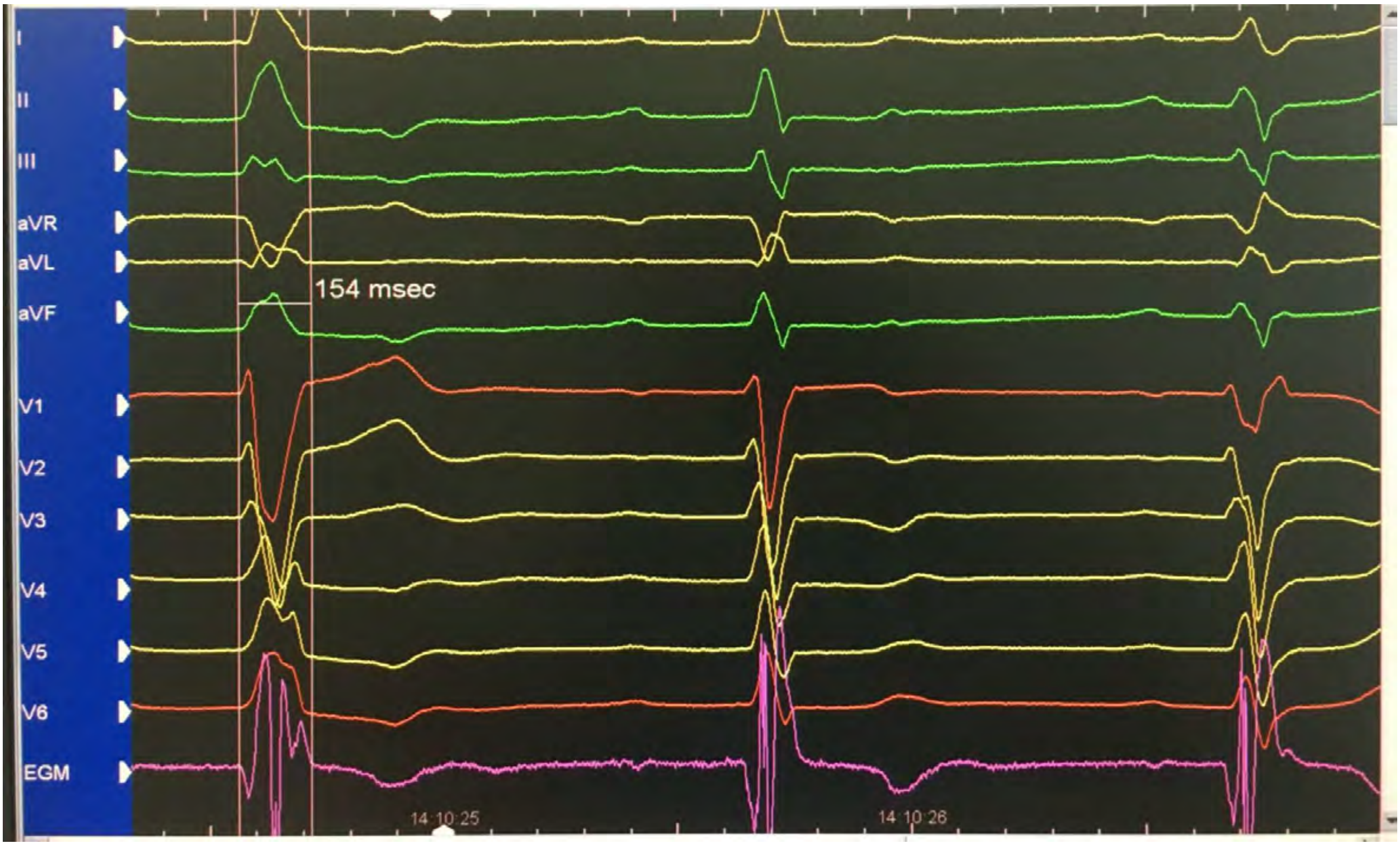

Post ECG

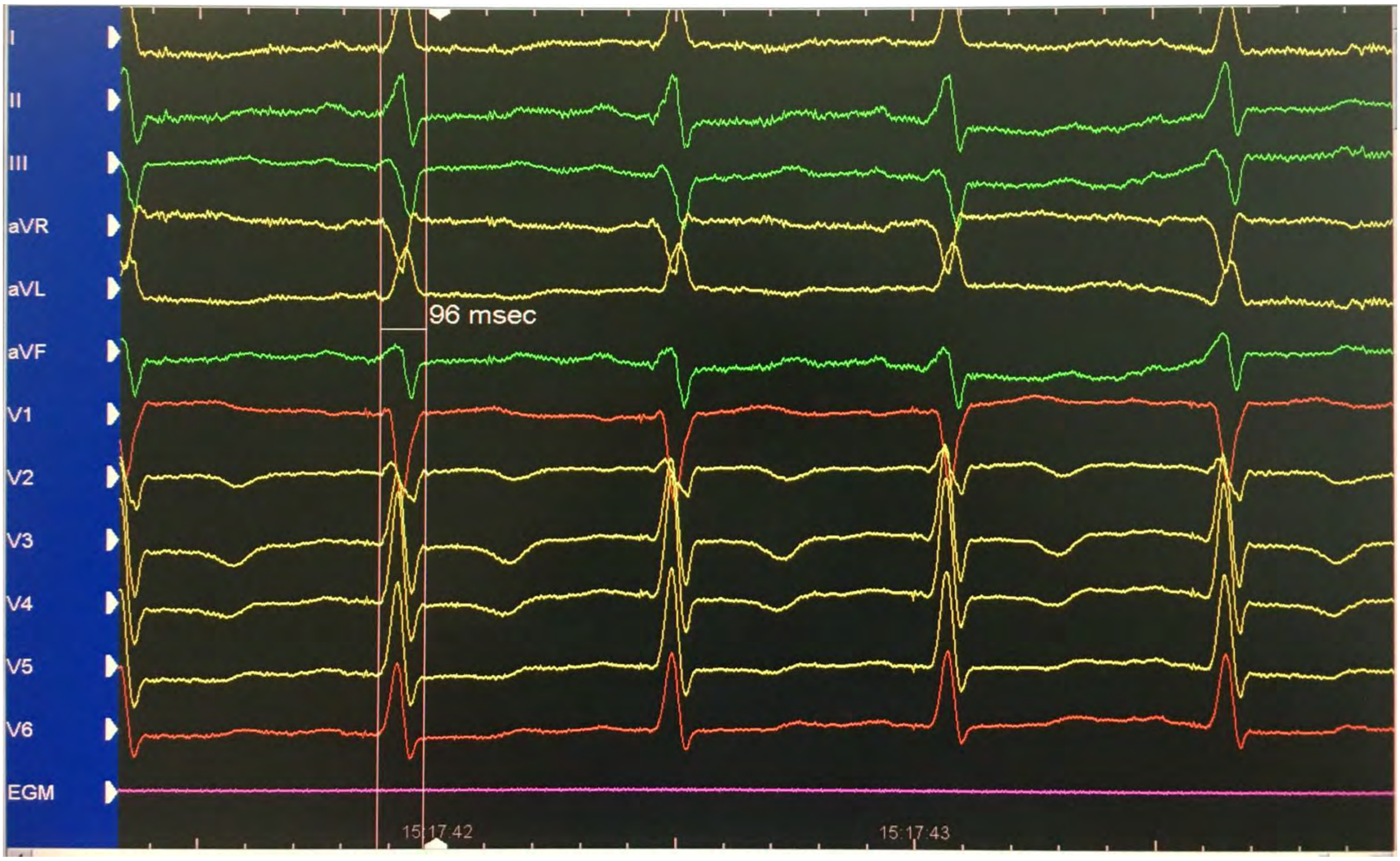

Patient 12:
Transitions

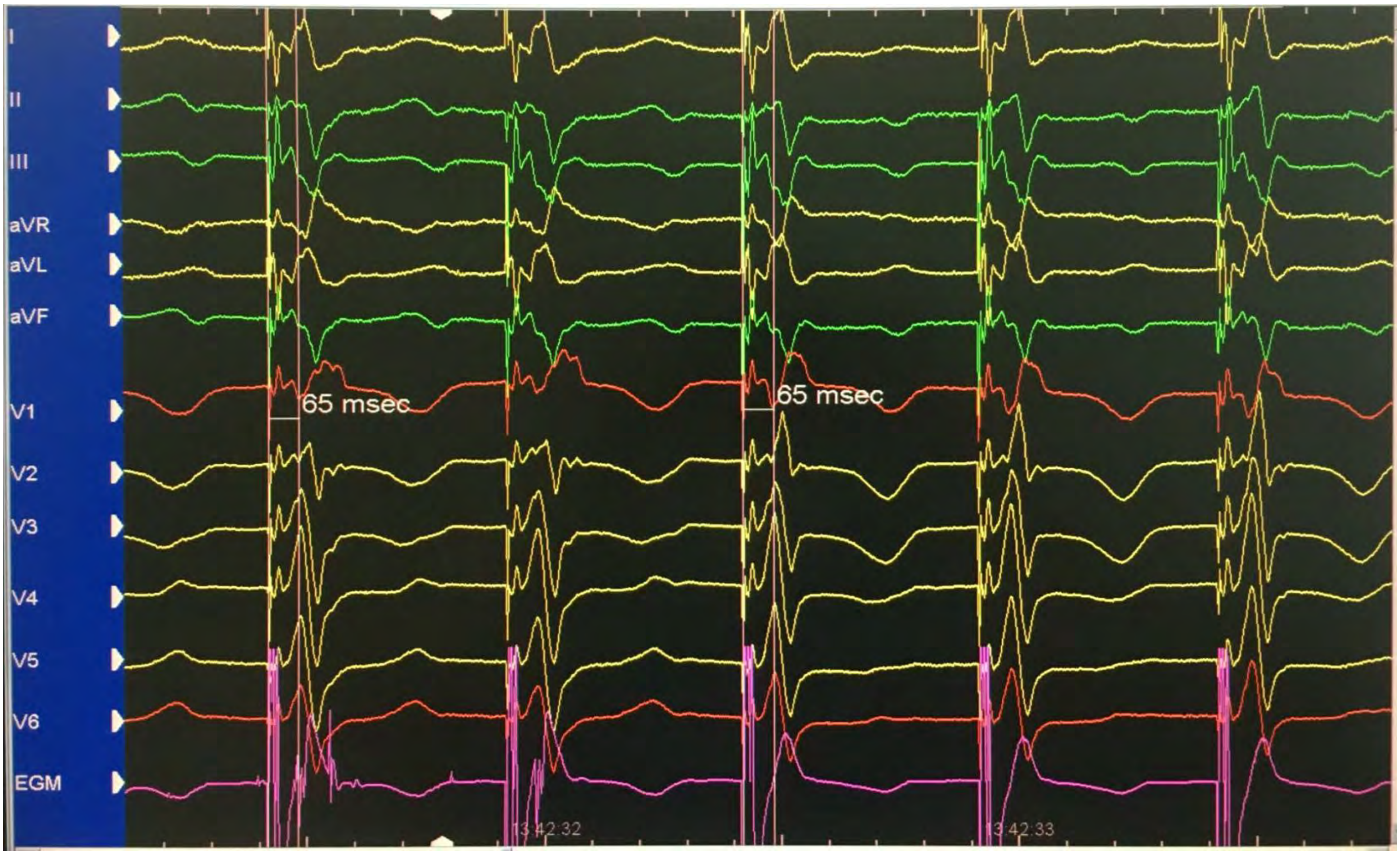

Transitions

Patient 13:
Pre-ECG

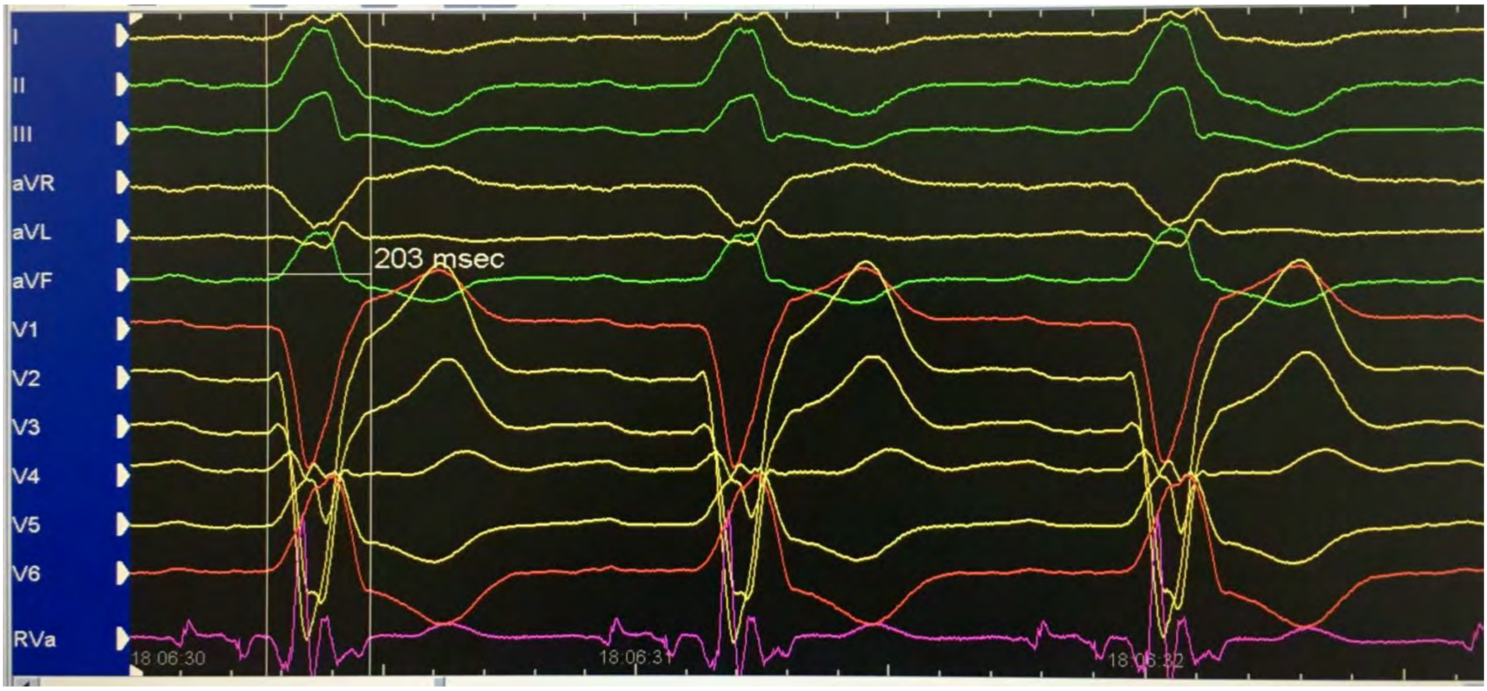

Post ECG

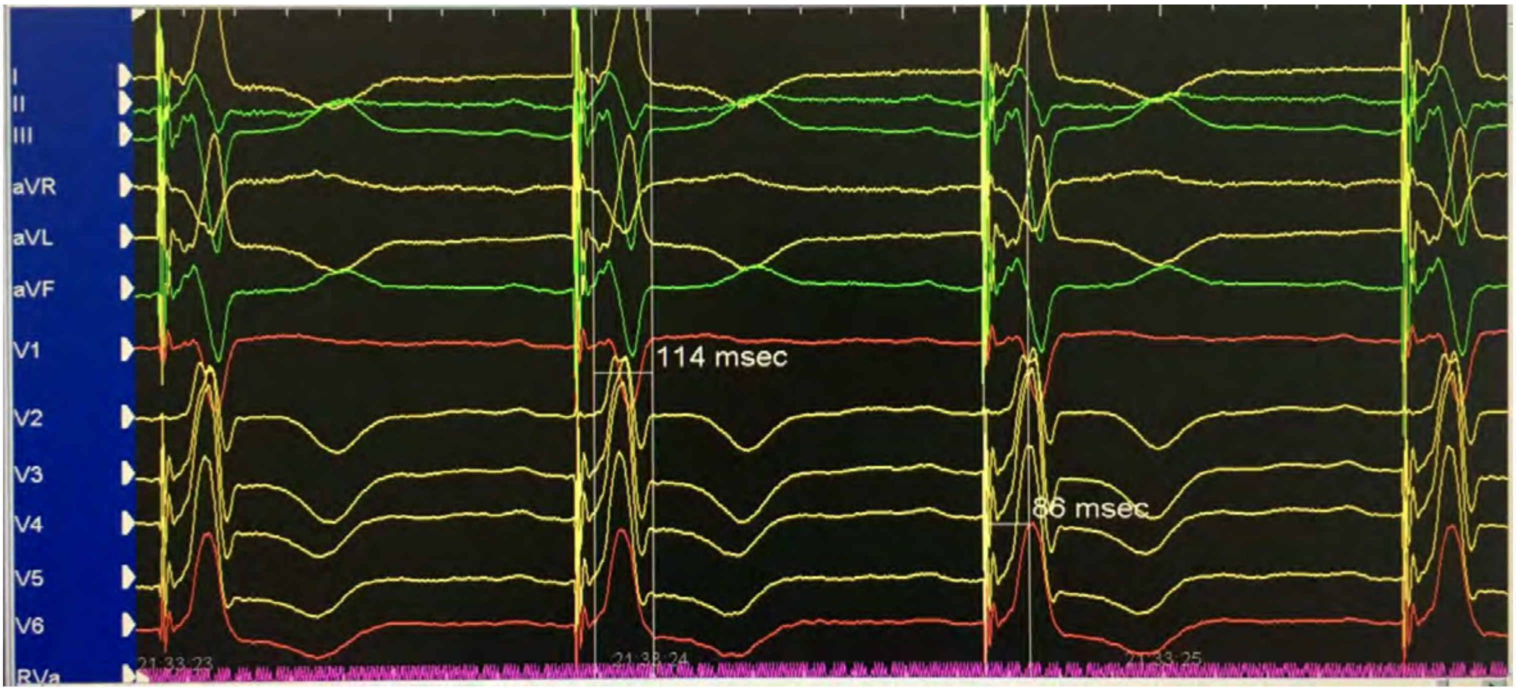

Patient 13:
Transitions

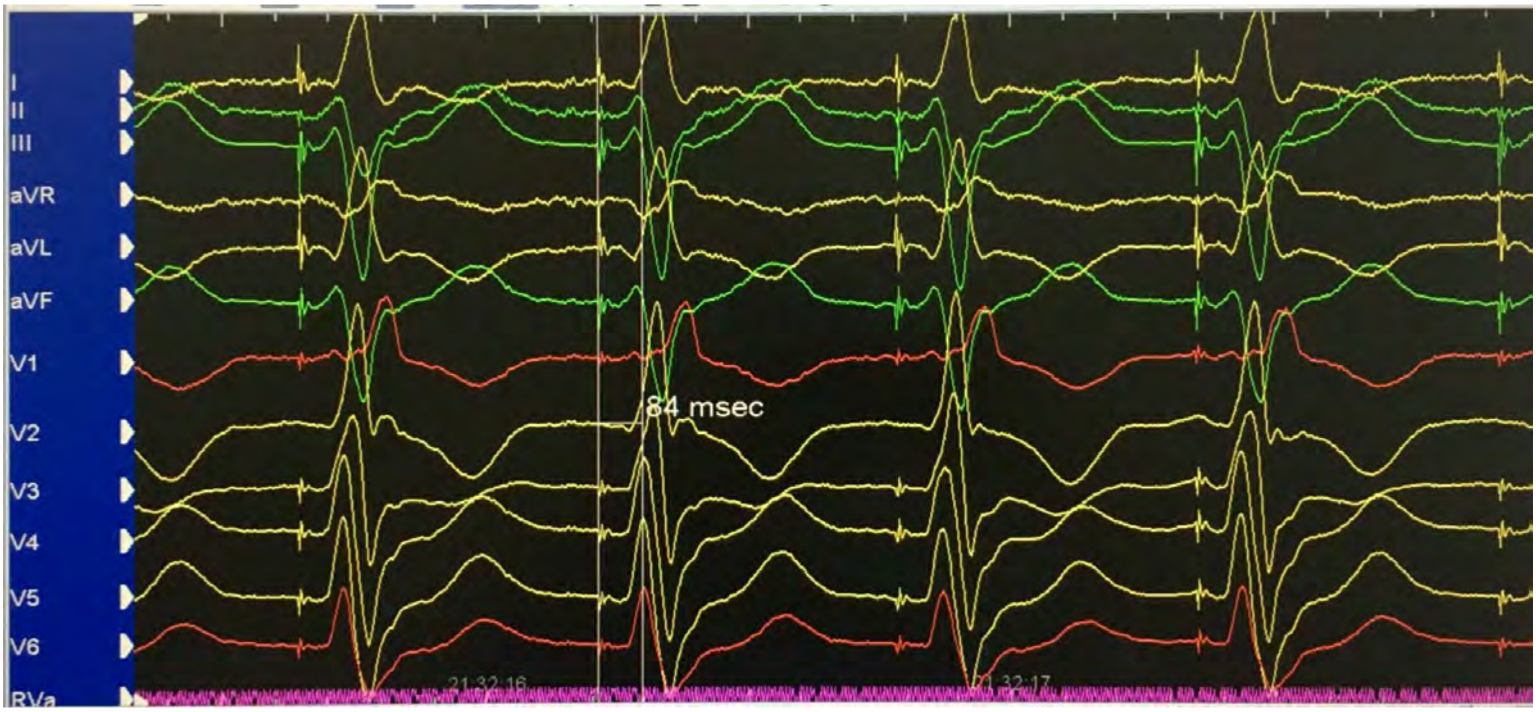

Transitions

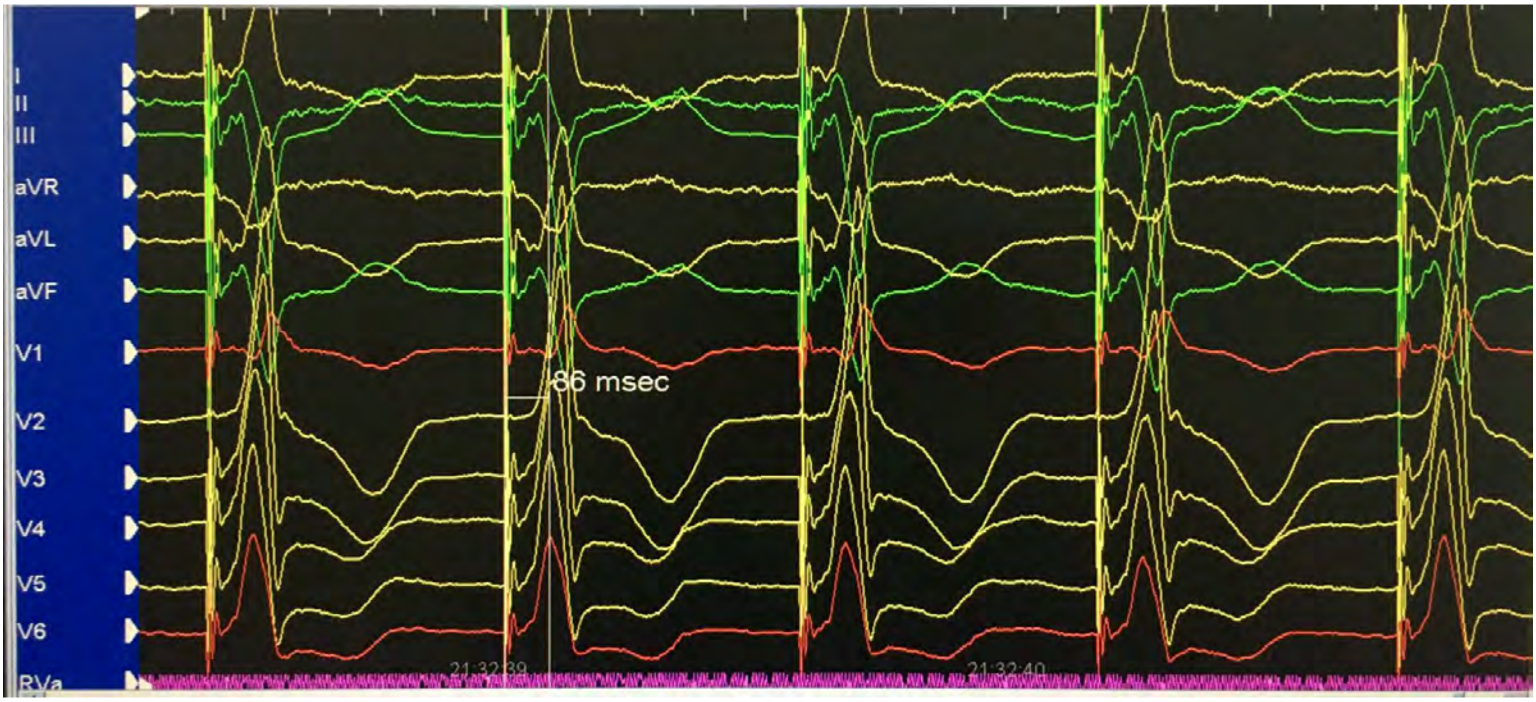

Patient 14:

Pre-ECG

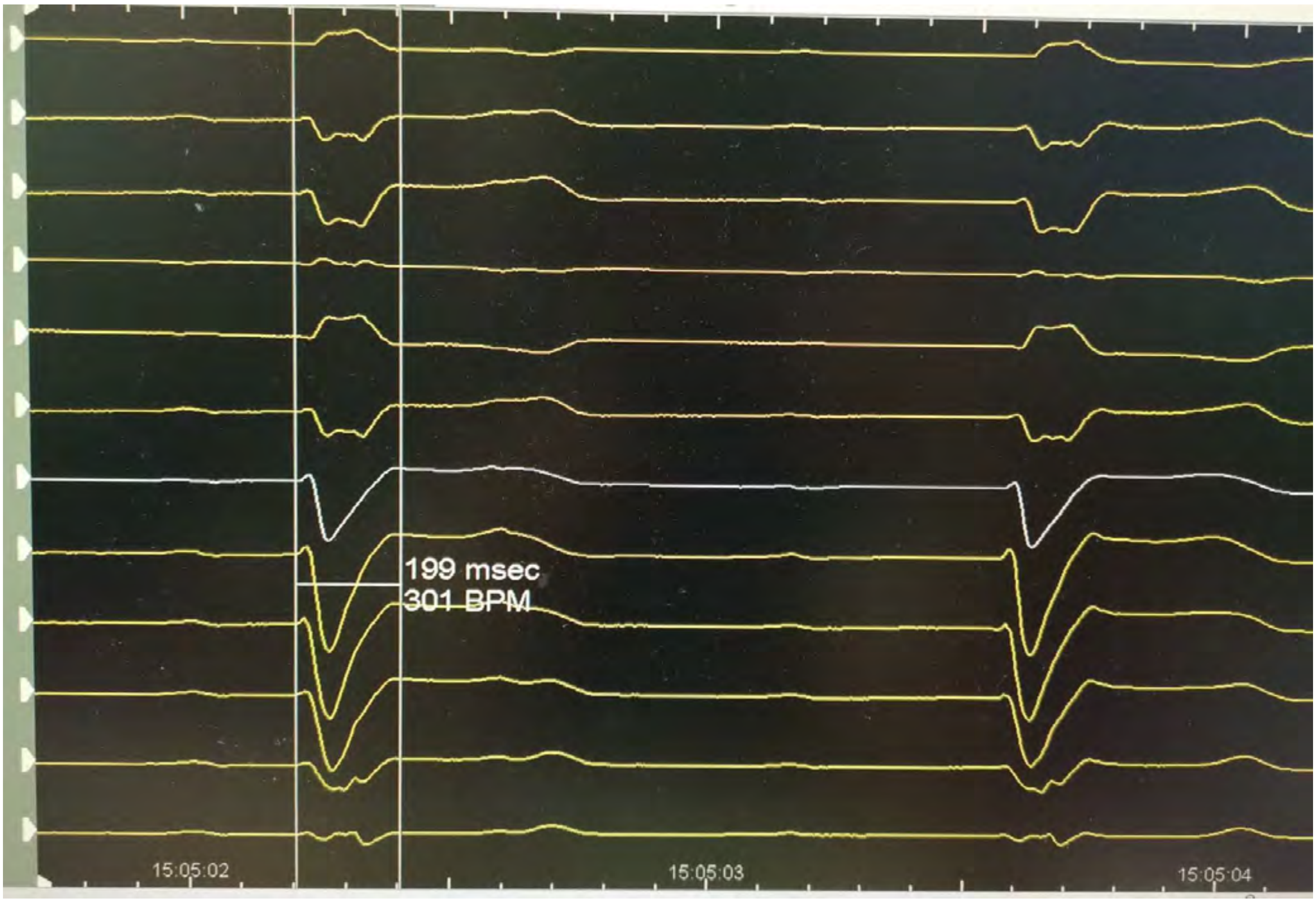

Transitions

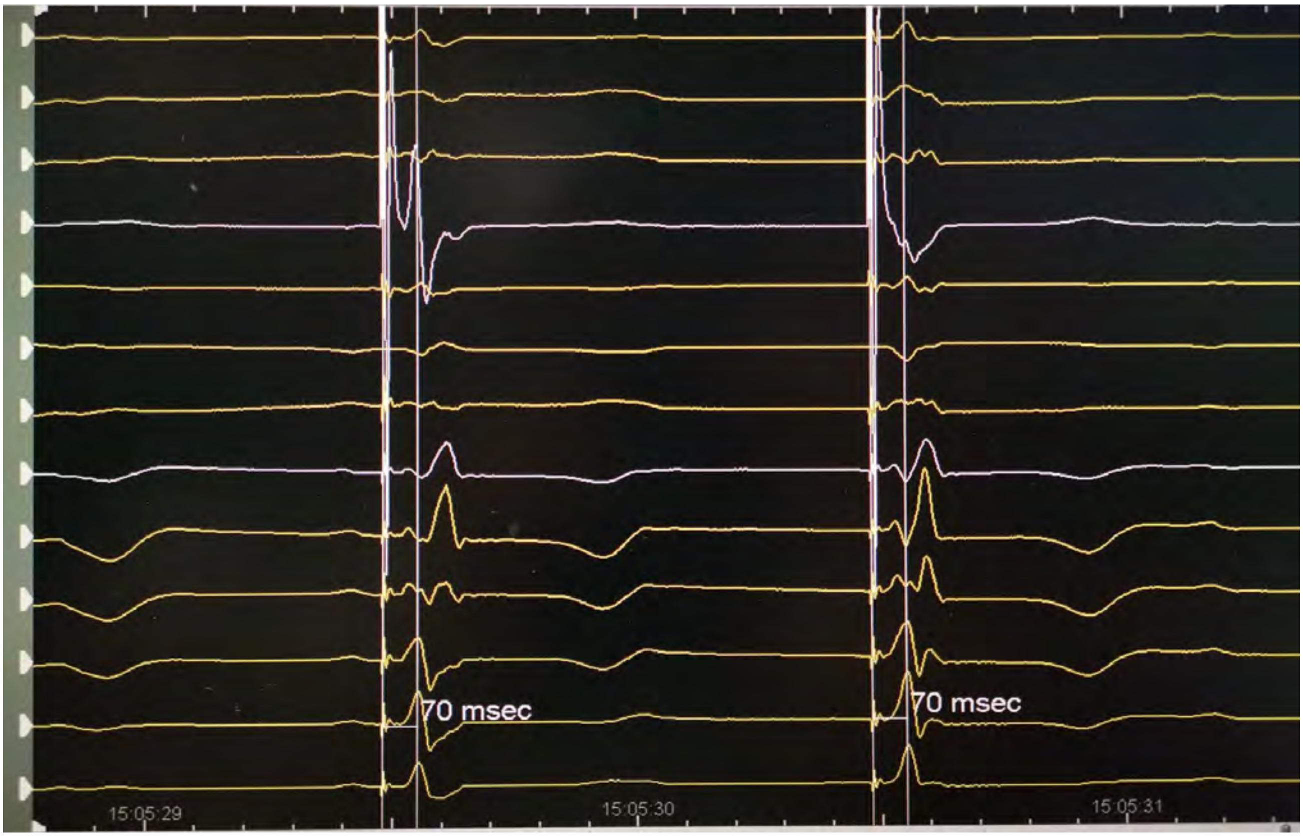

Patient 15:

Pre-ECG

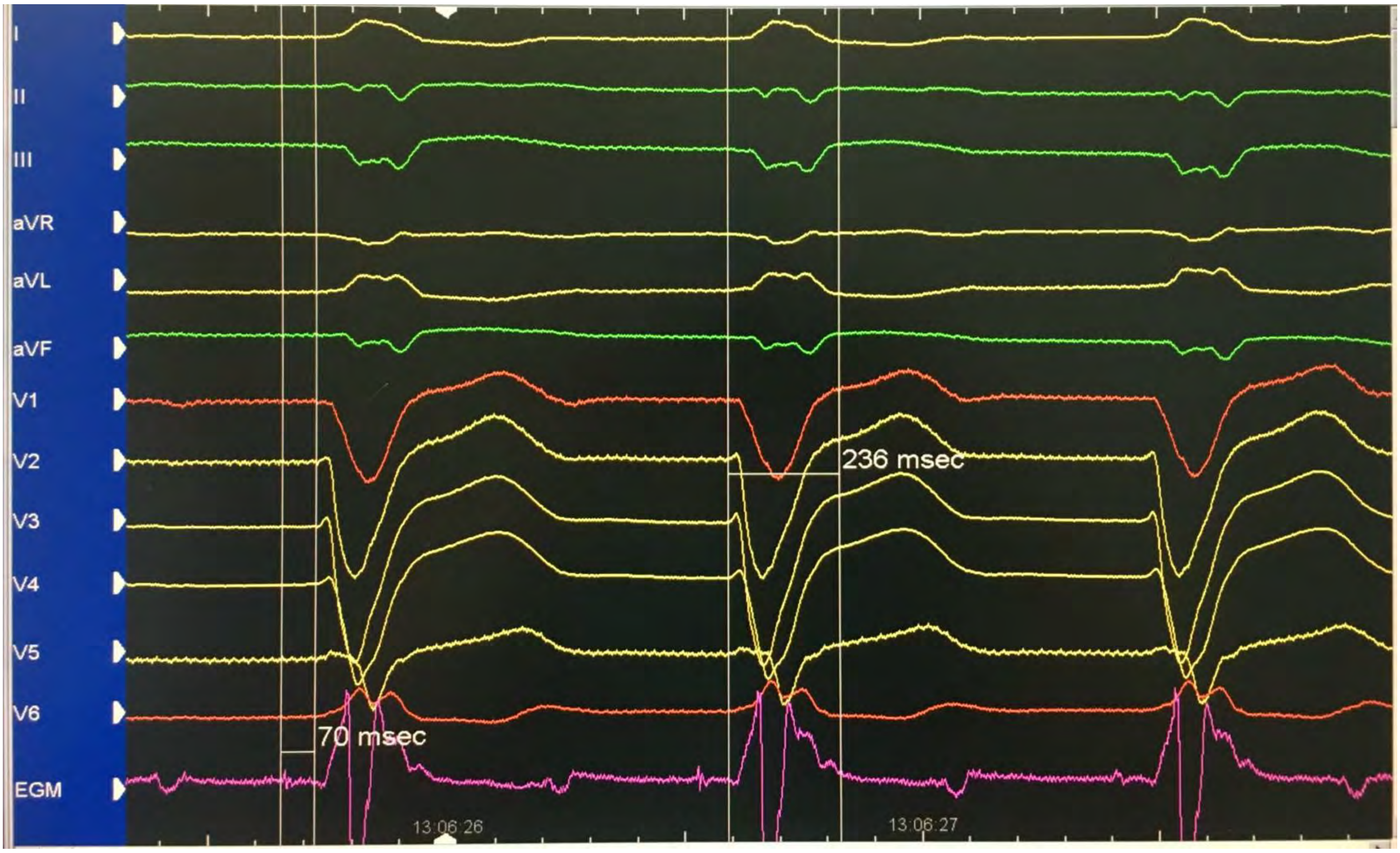

Post ECG

Patient 15:
Transitions

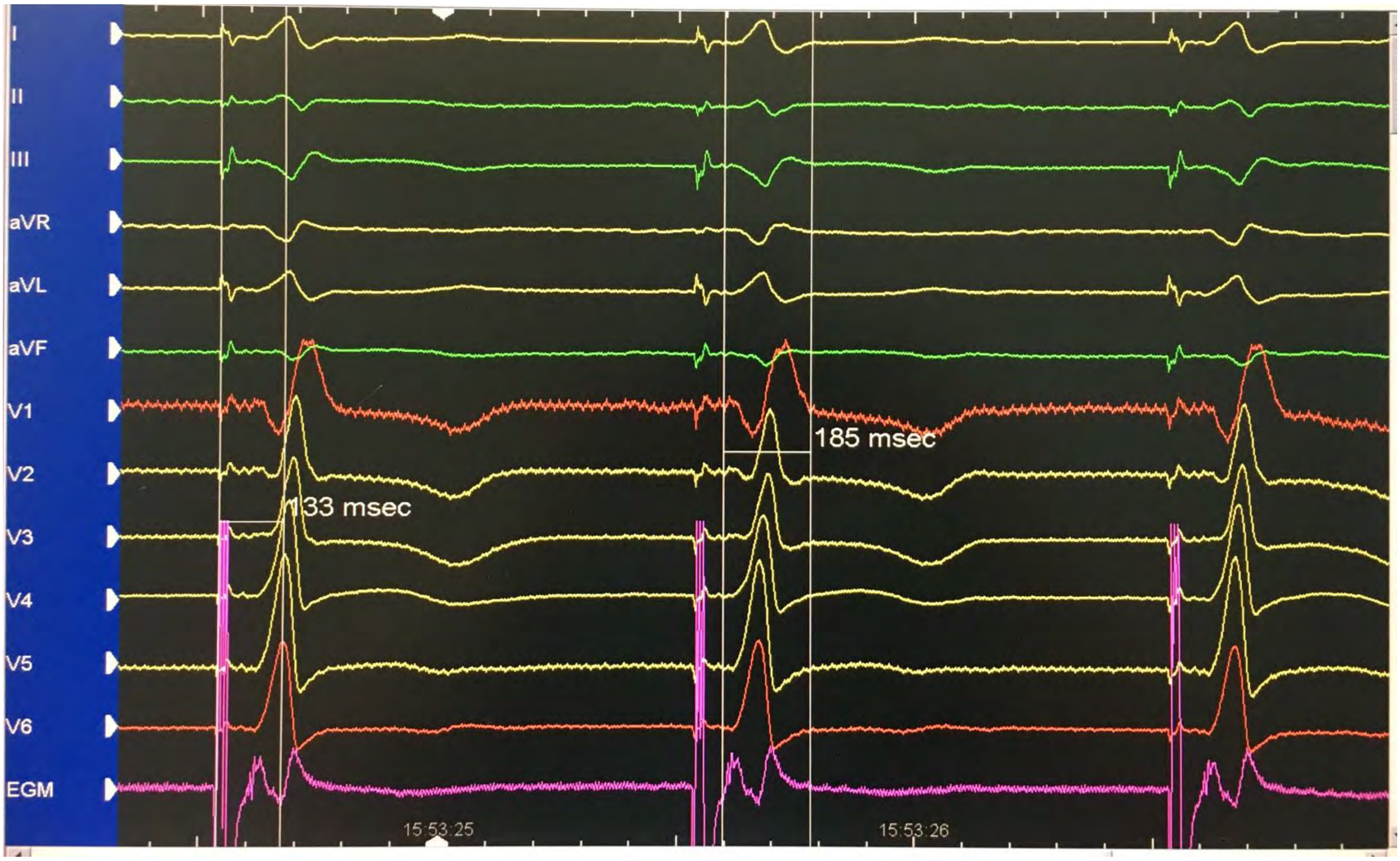

Transitions

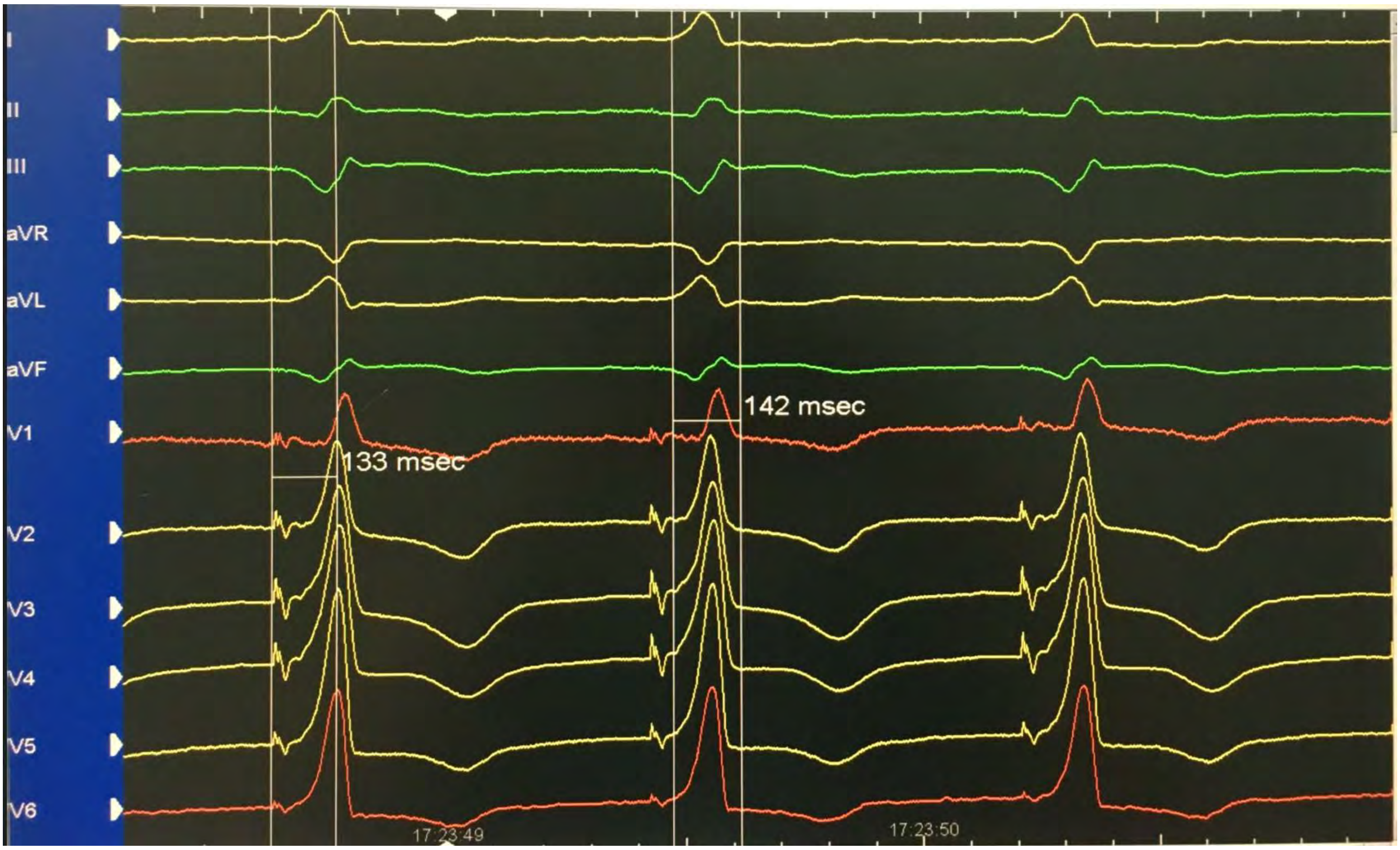

Patient 16:
Pre-ECG

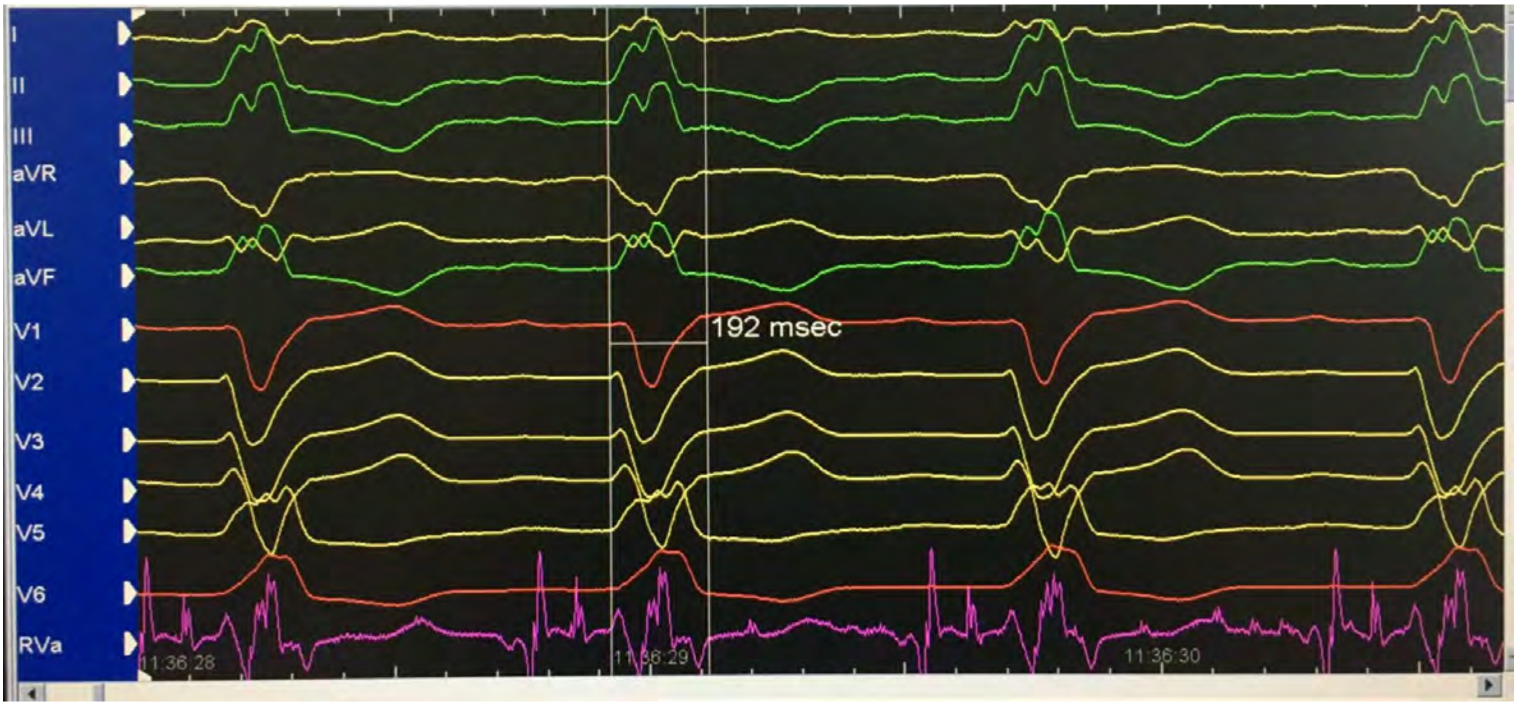

Transitions

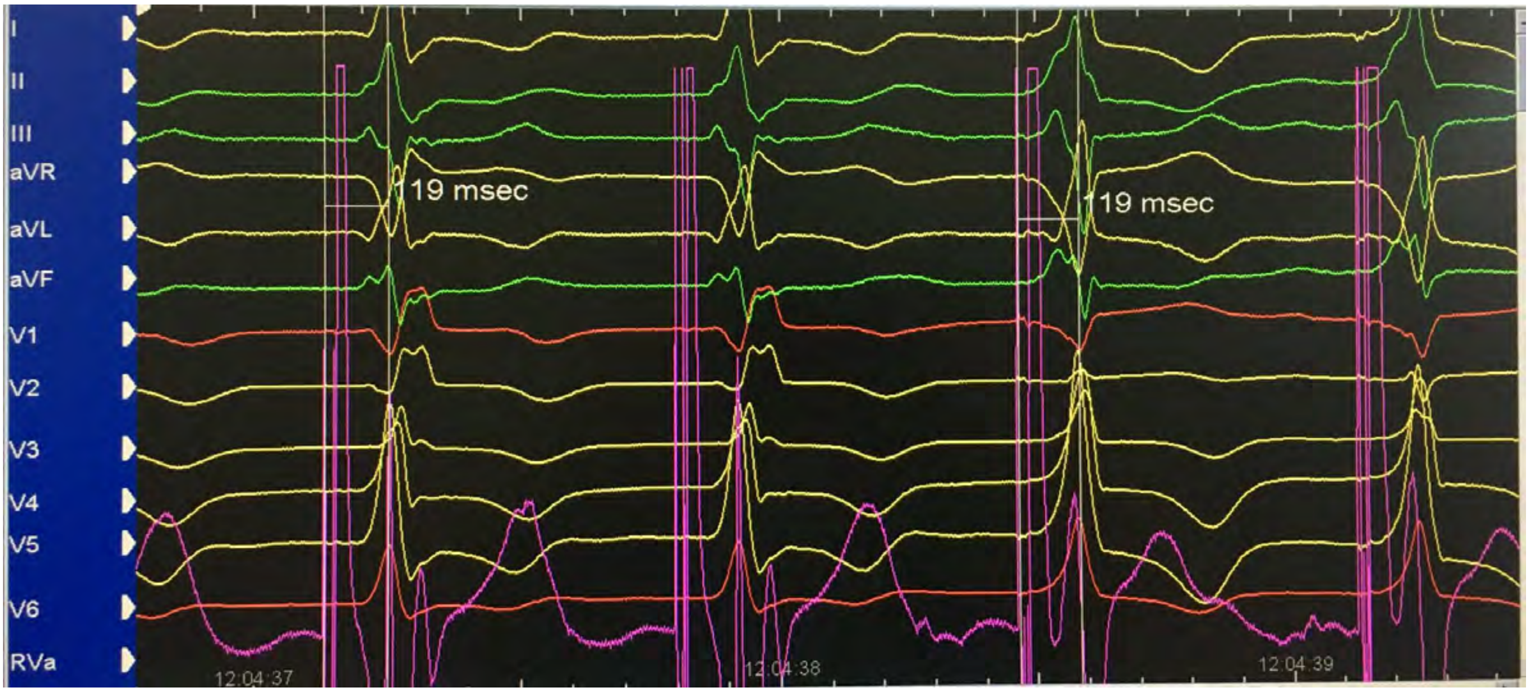

Patient 17:

Pre-ECG

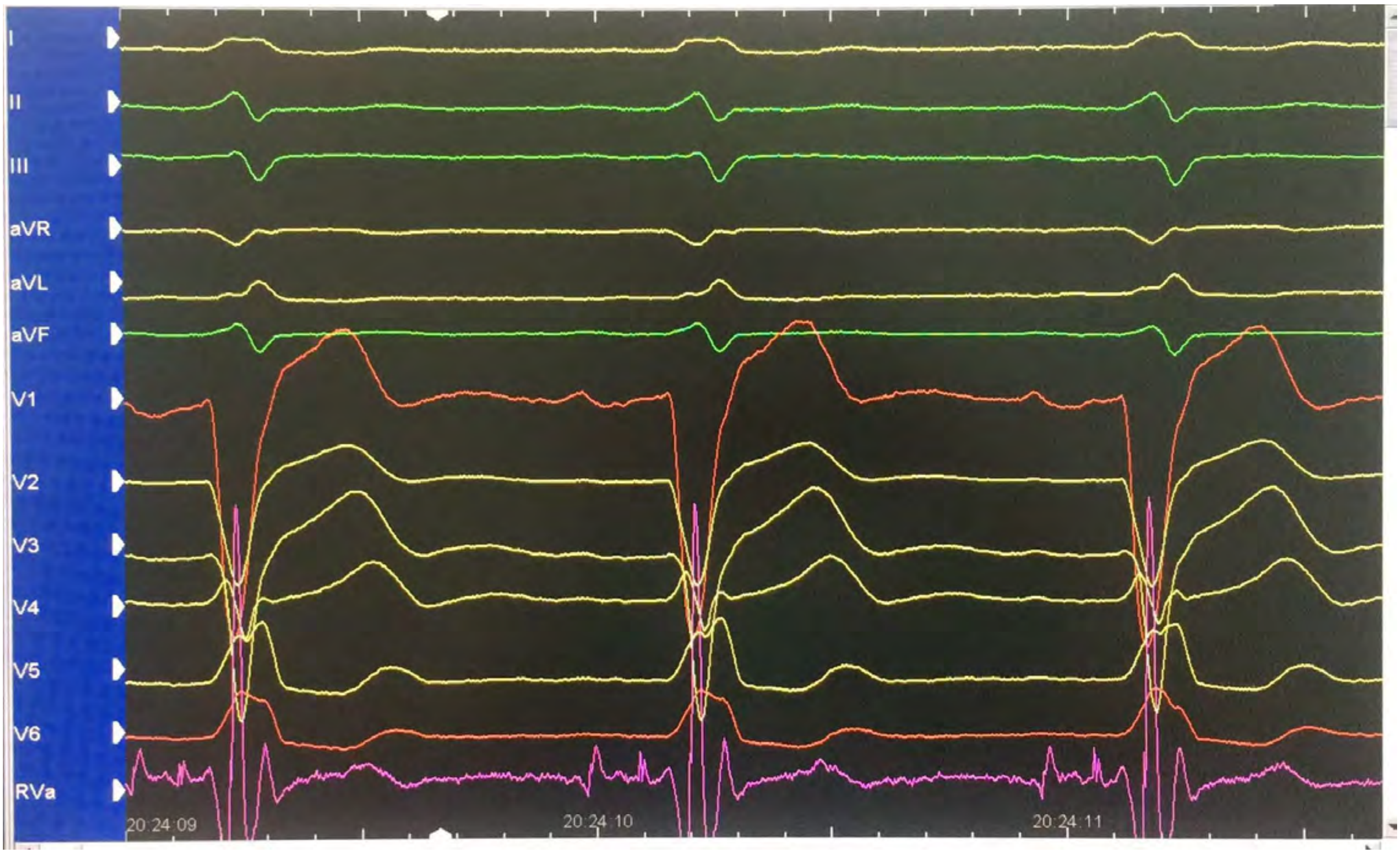

Post ECG

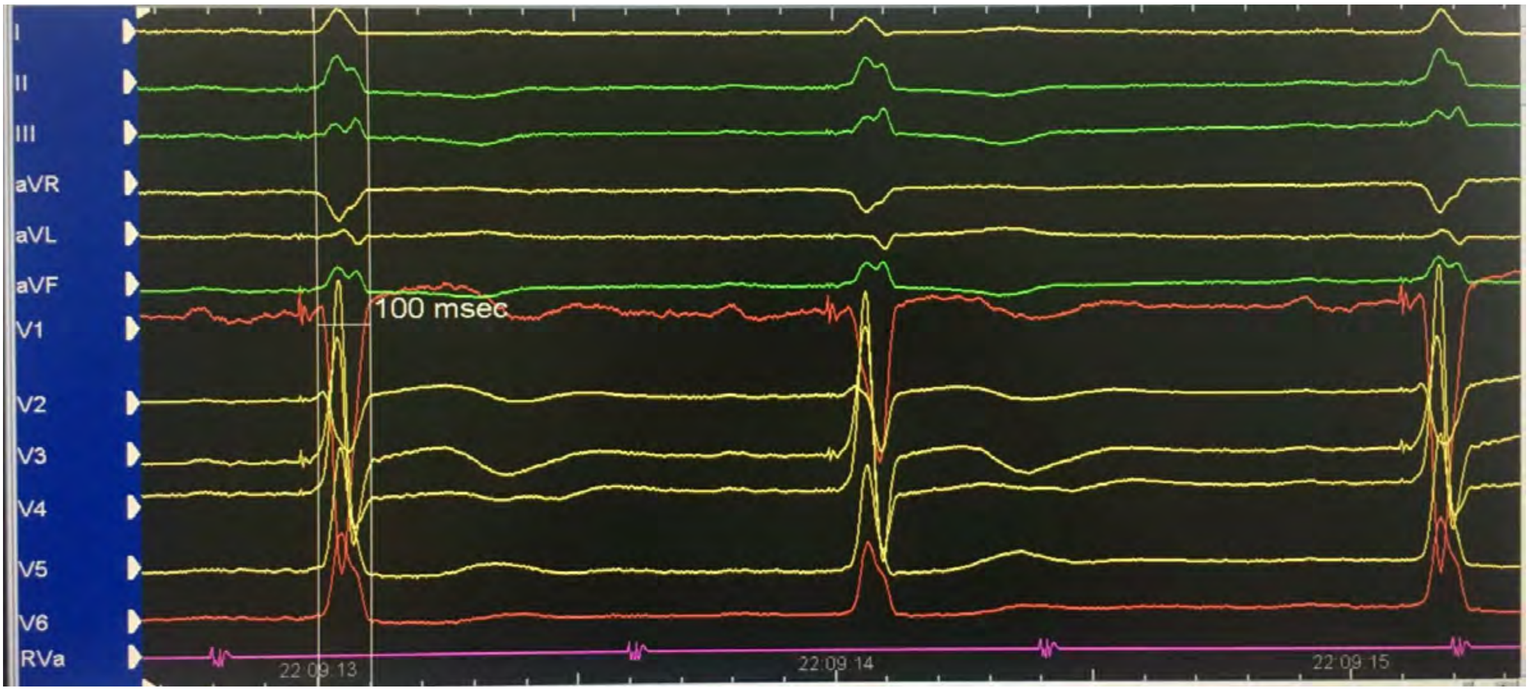

Patient 17:
Transitions

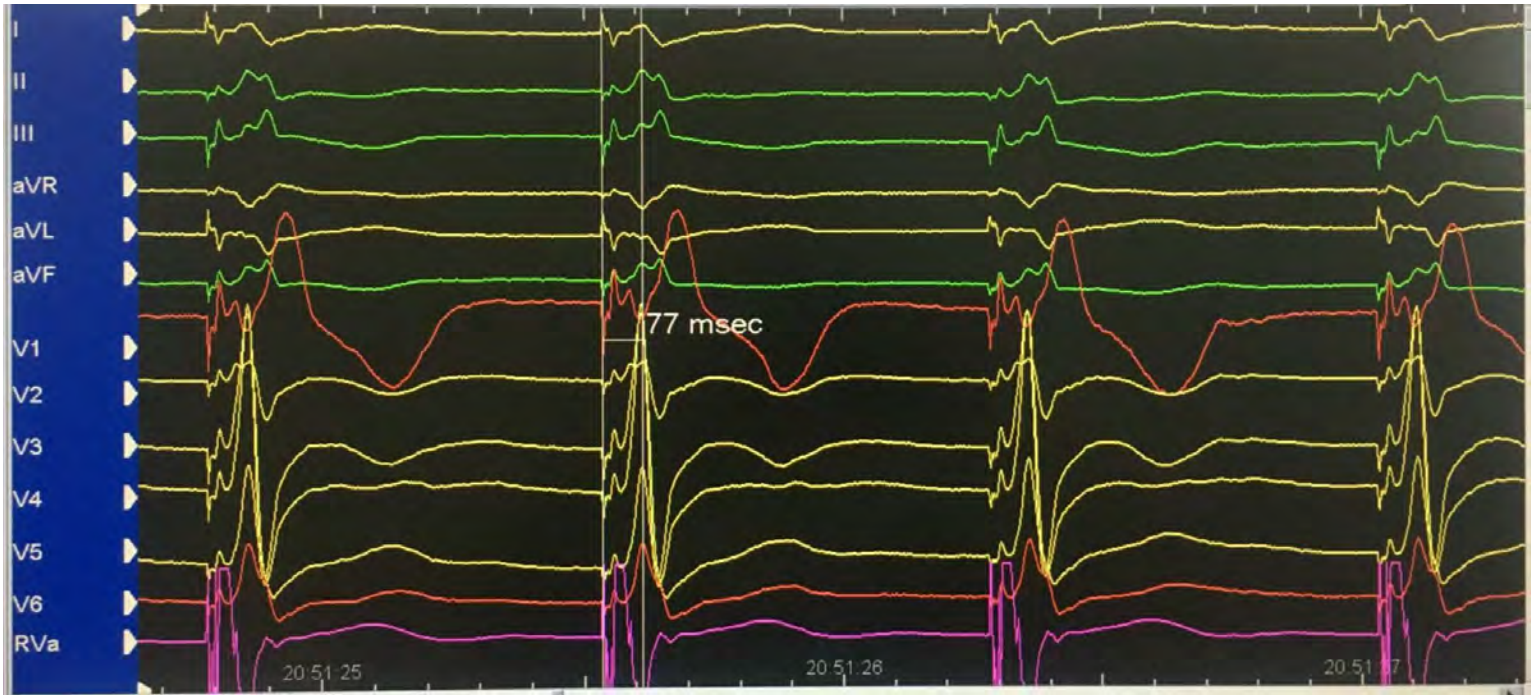

Transitions

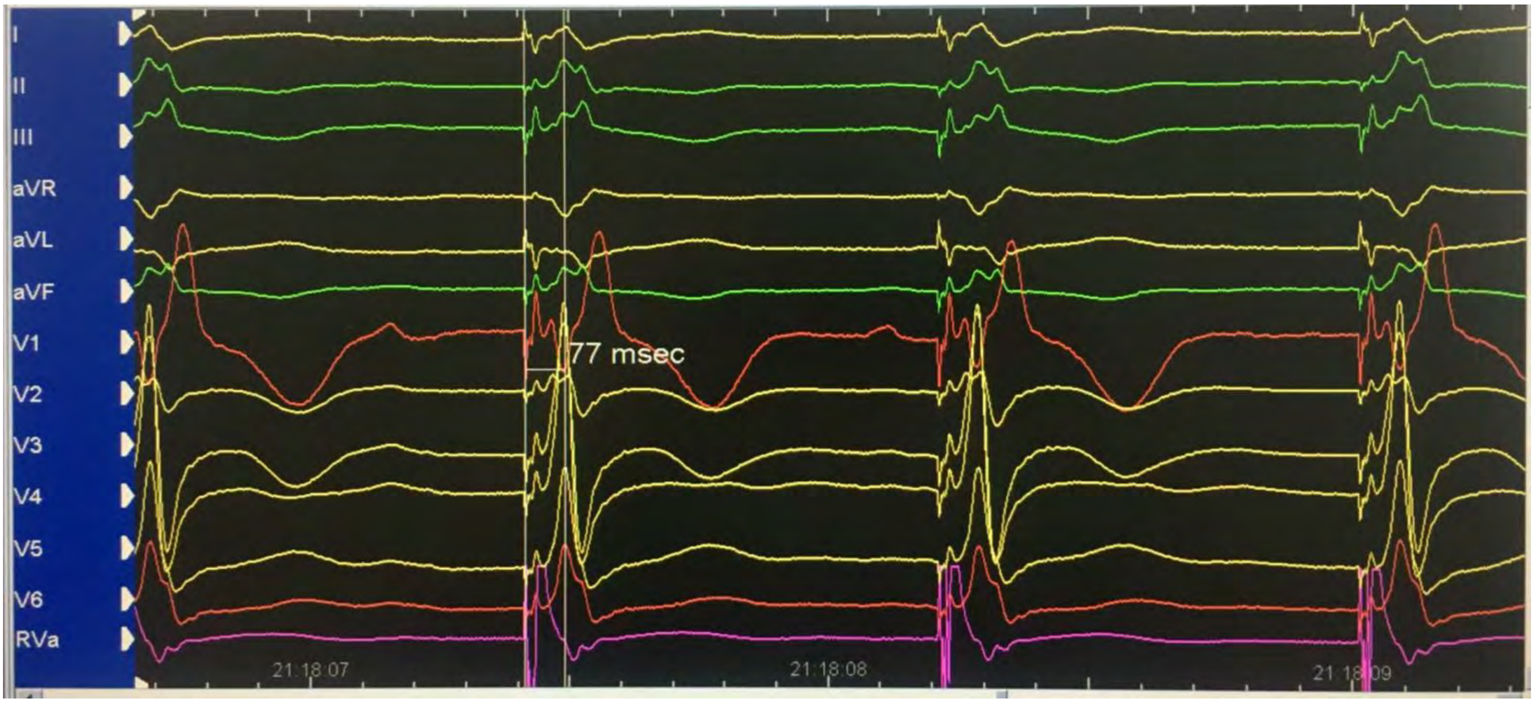

Patient 18:
Pre-ECG

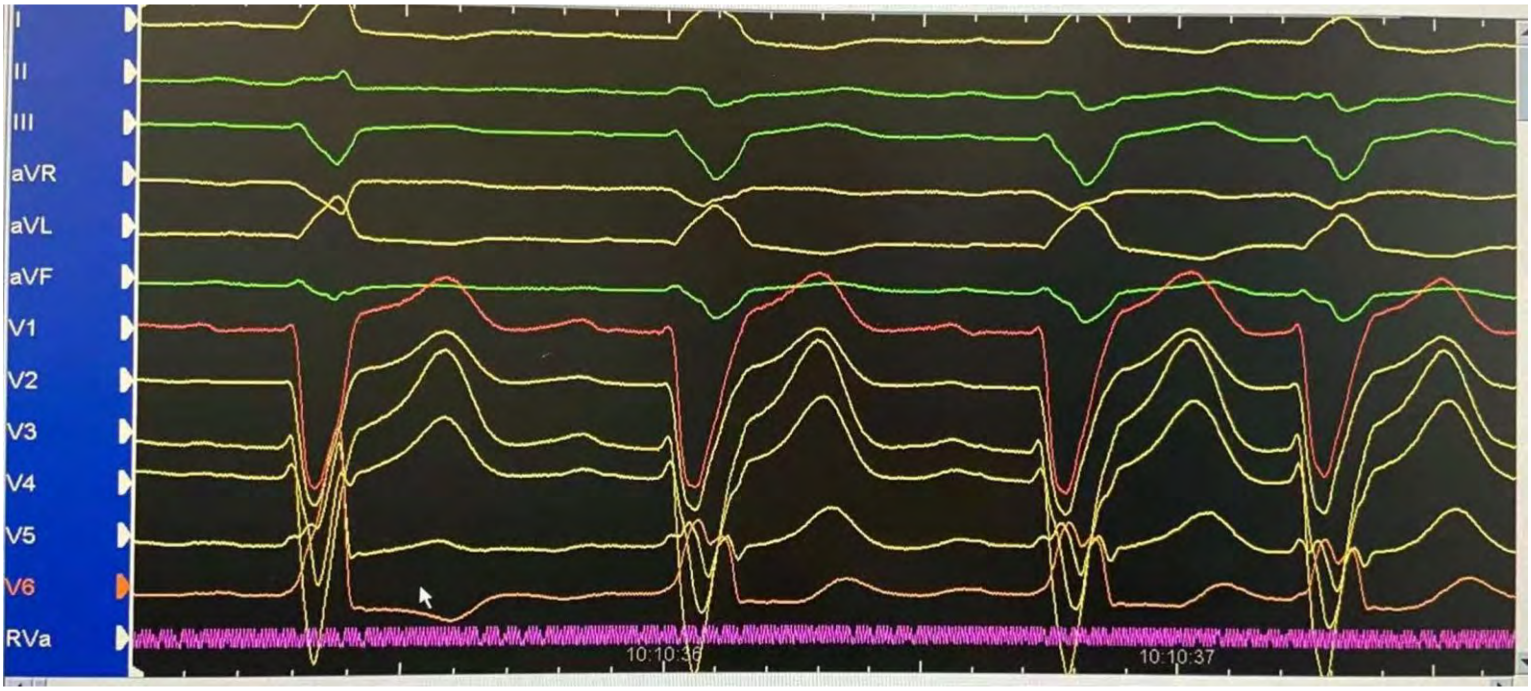

Post ECG

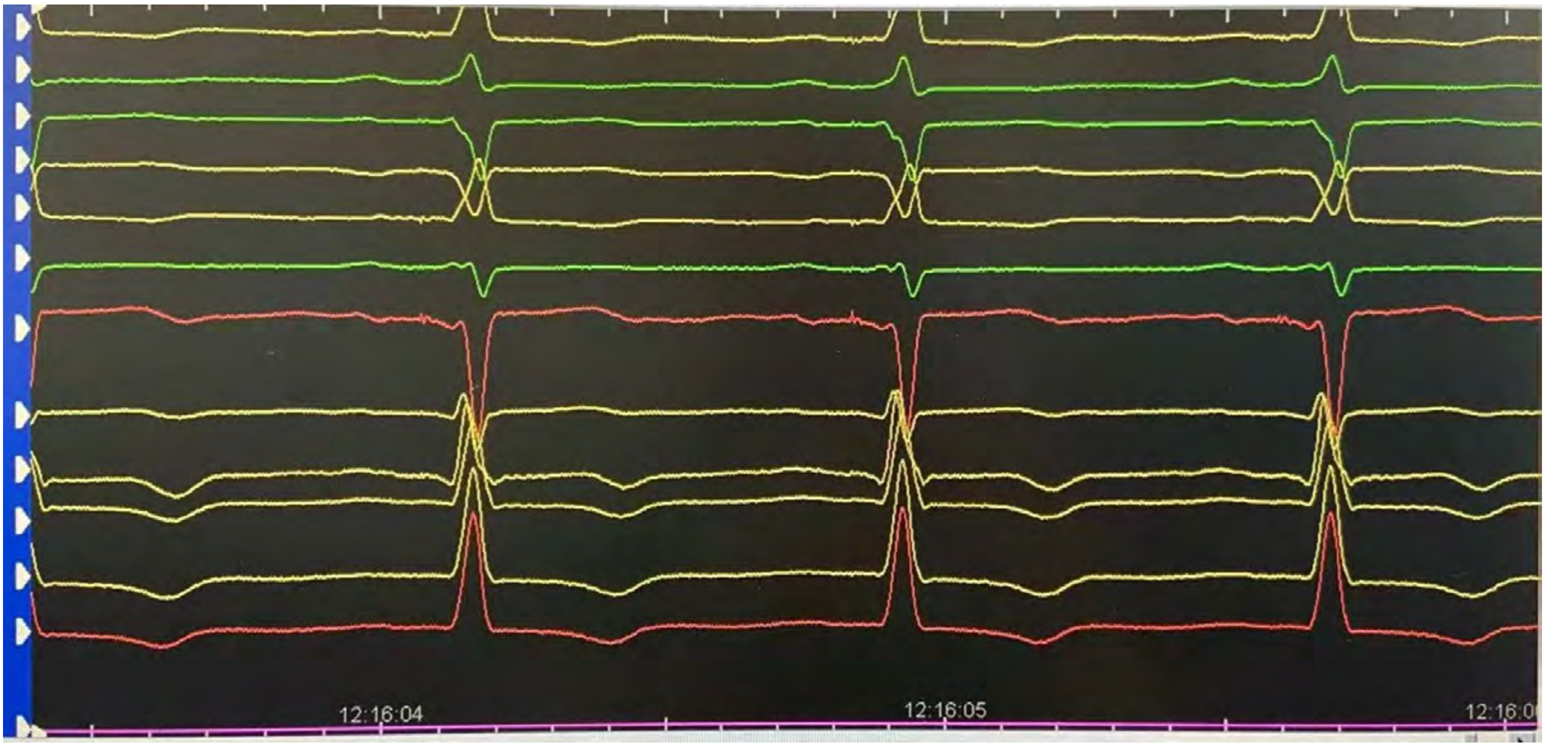

Patient 19:
Pre-ECG

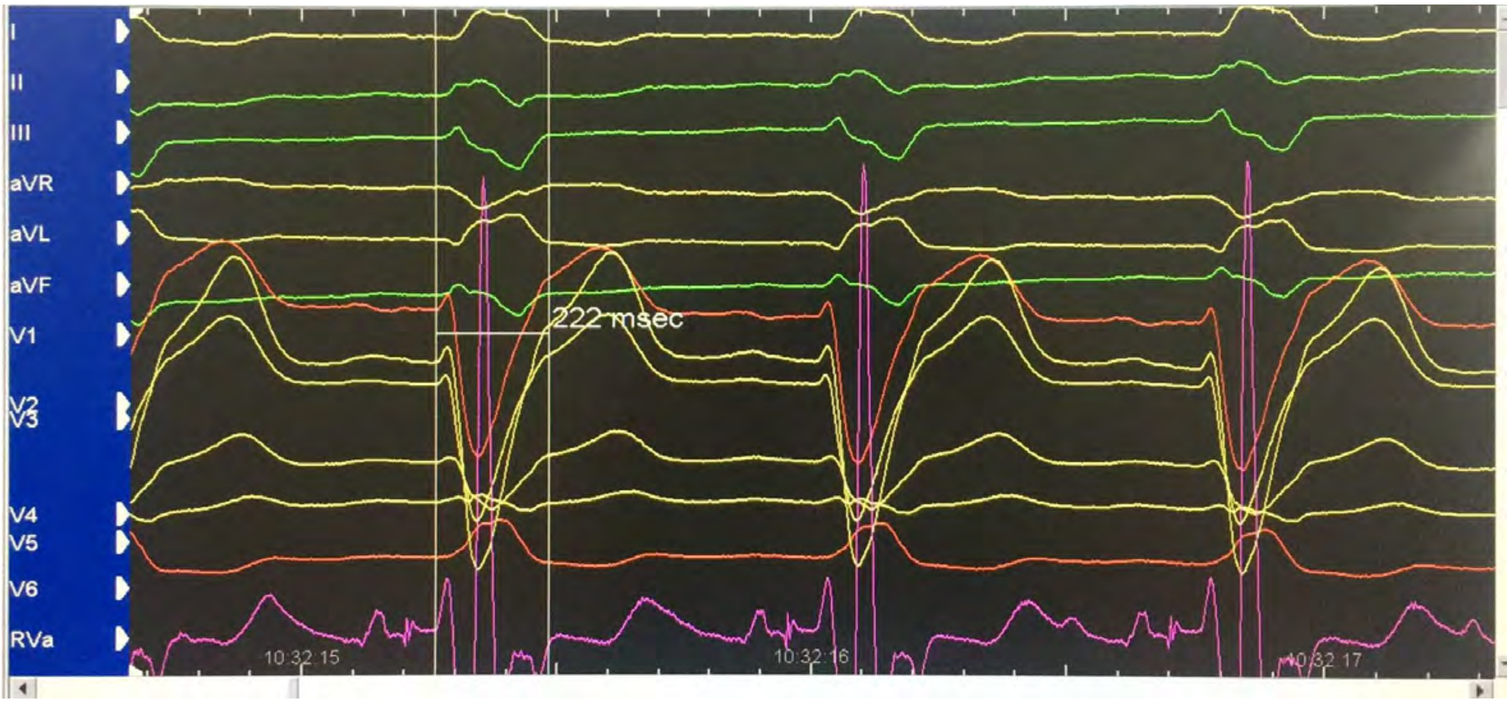

Post ECG

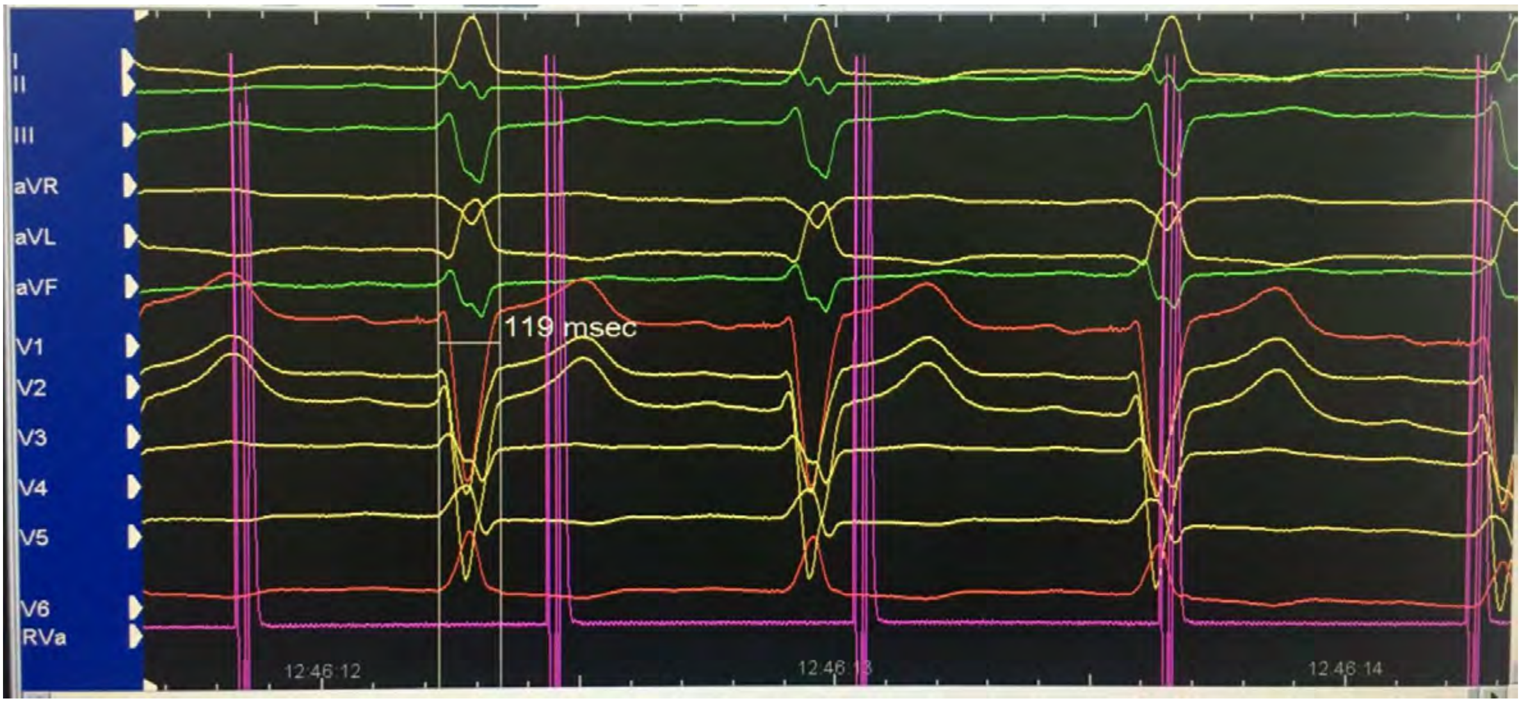

Patient 19:
Transitions

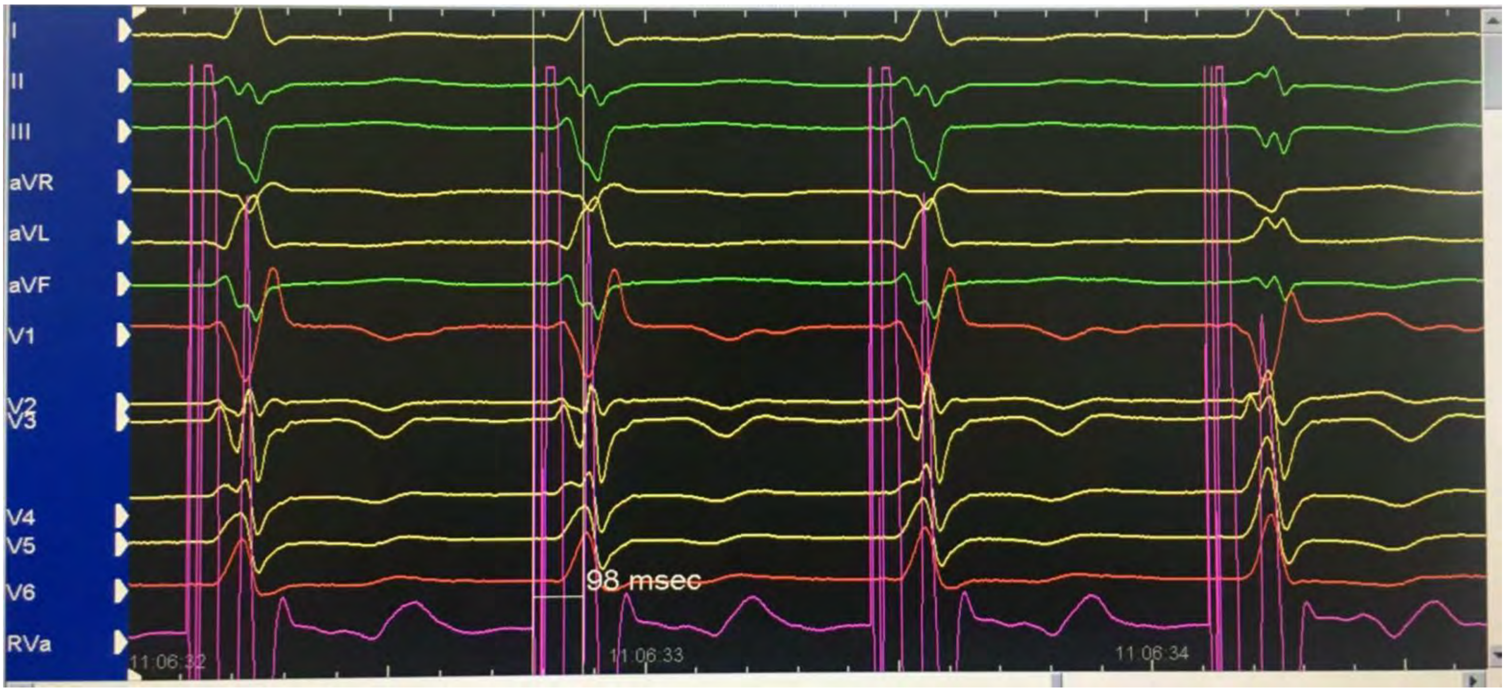

Transitions

Patient 20:
Pre-ECG

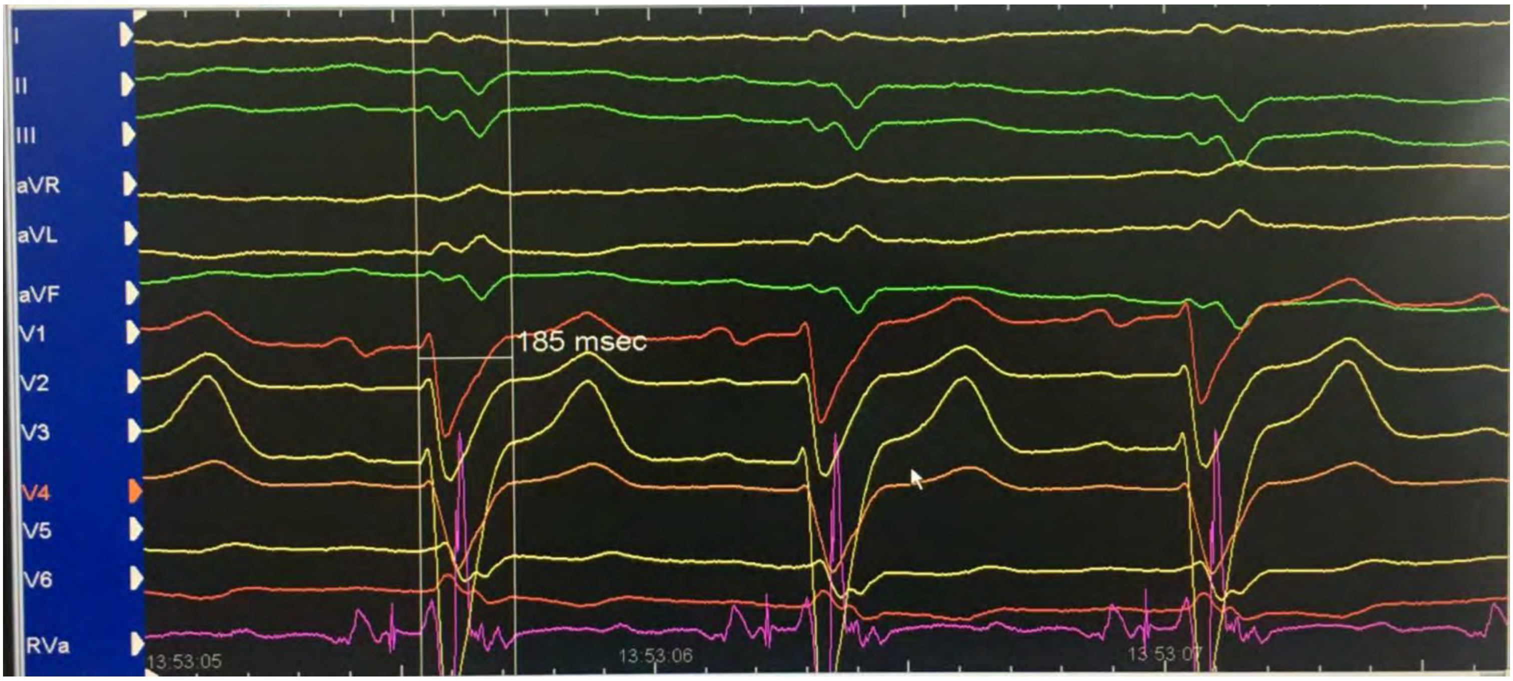

Post ECG

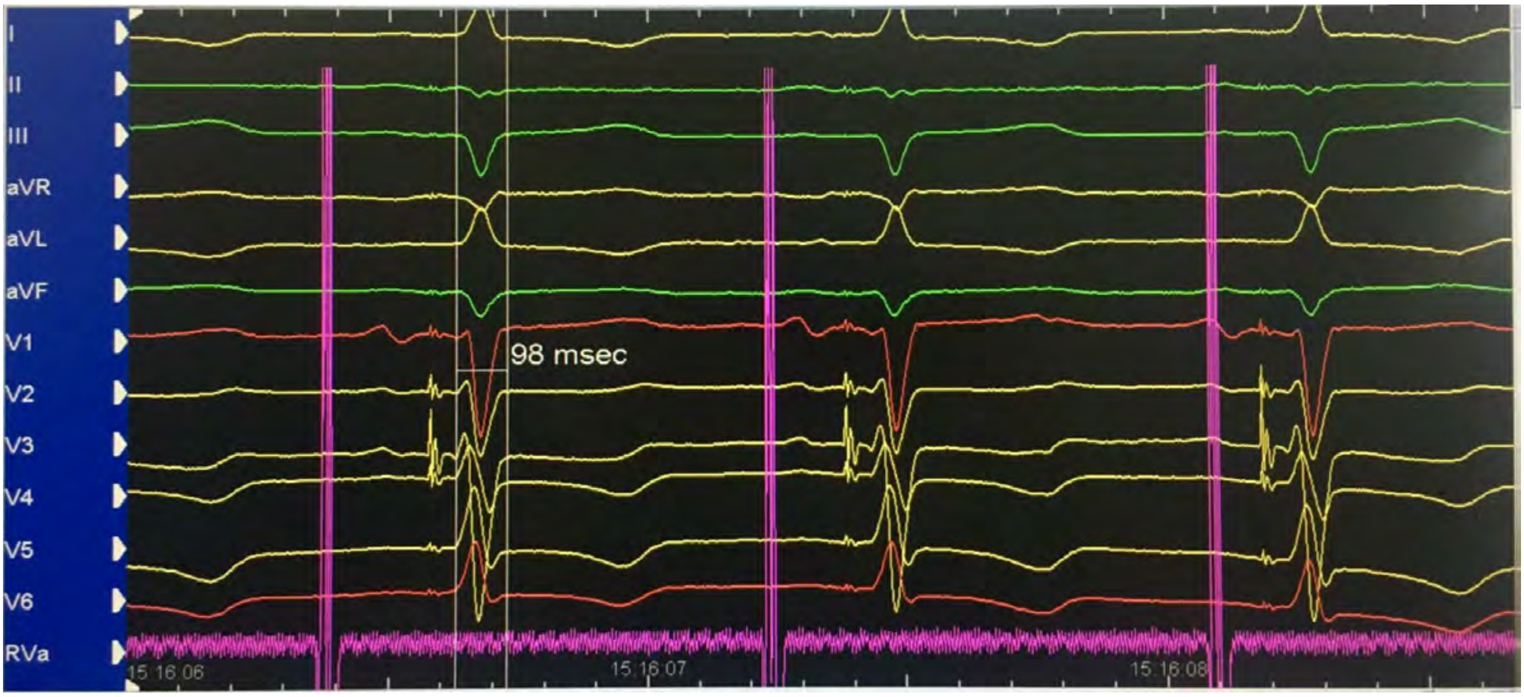

Patient 20:
Transitions

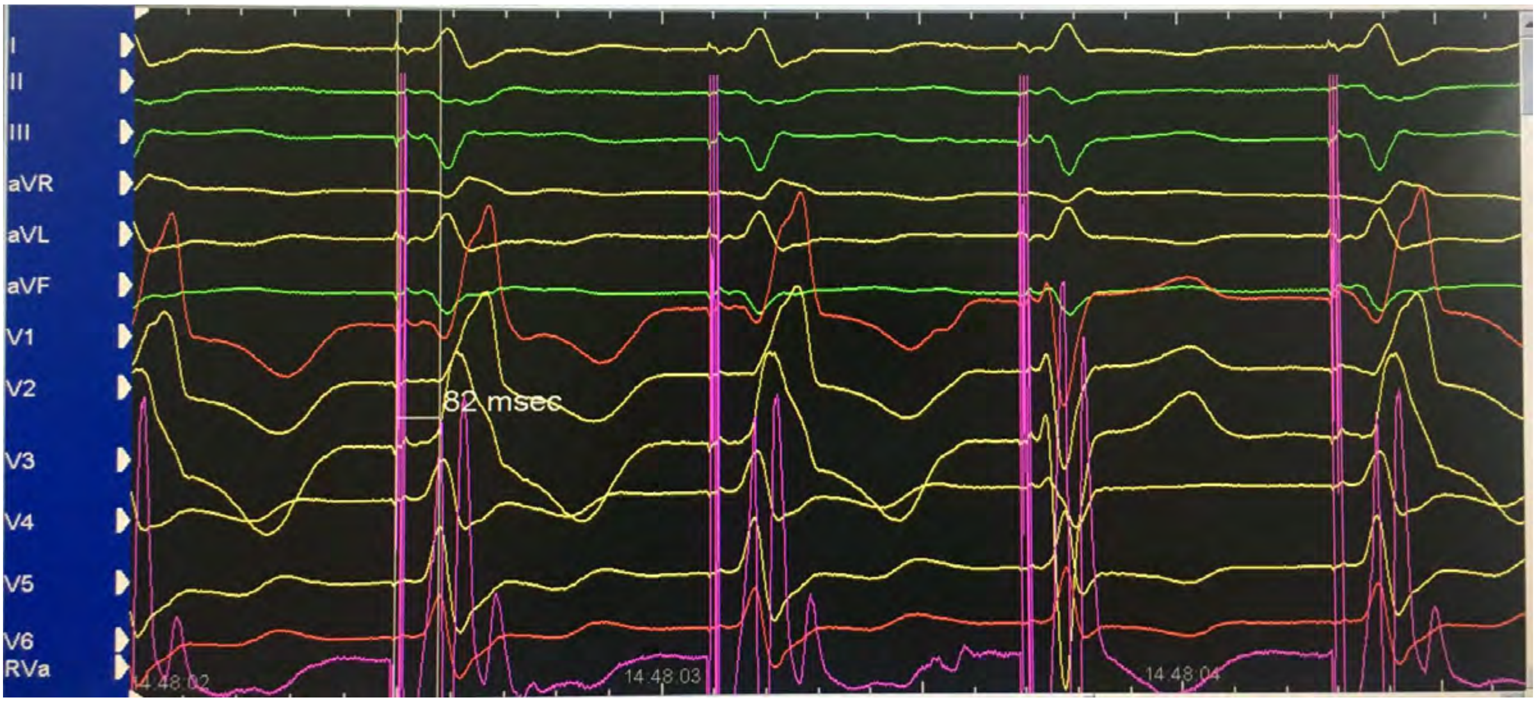

Transitions

Patient 21:
Pre-ECG

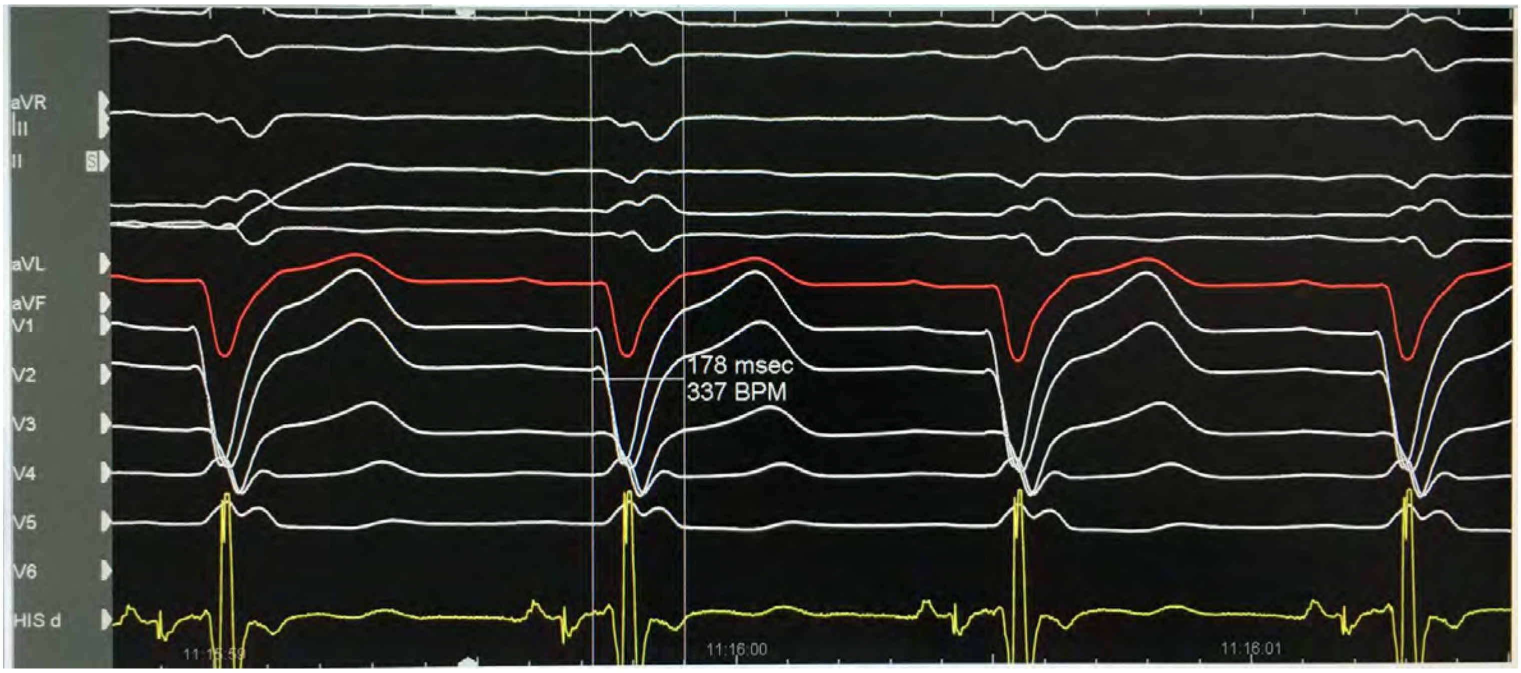

Post ECG

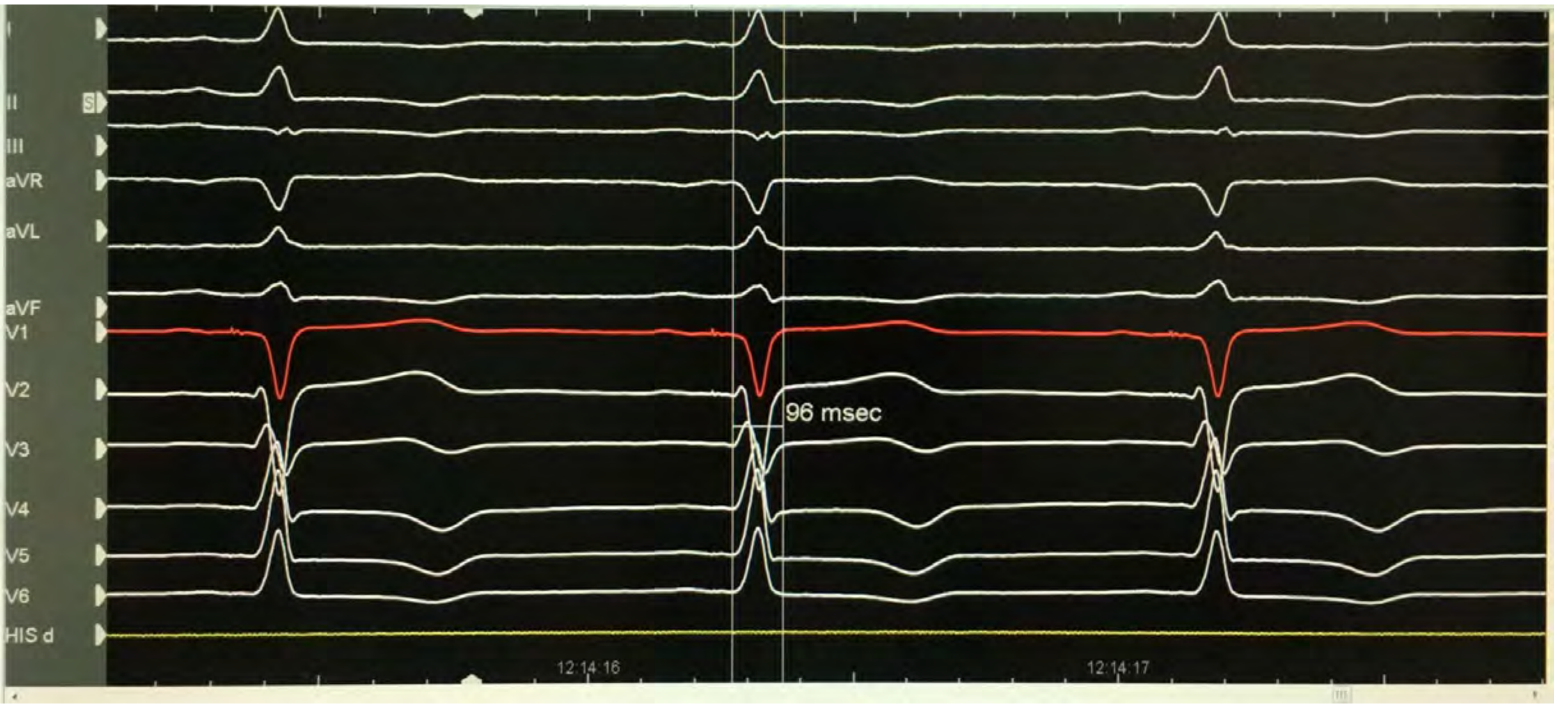

Patient 21:
Transitions

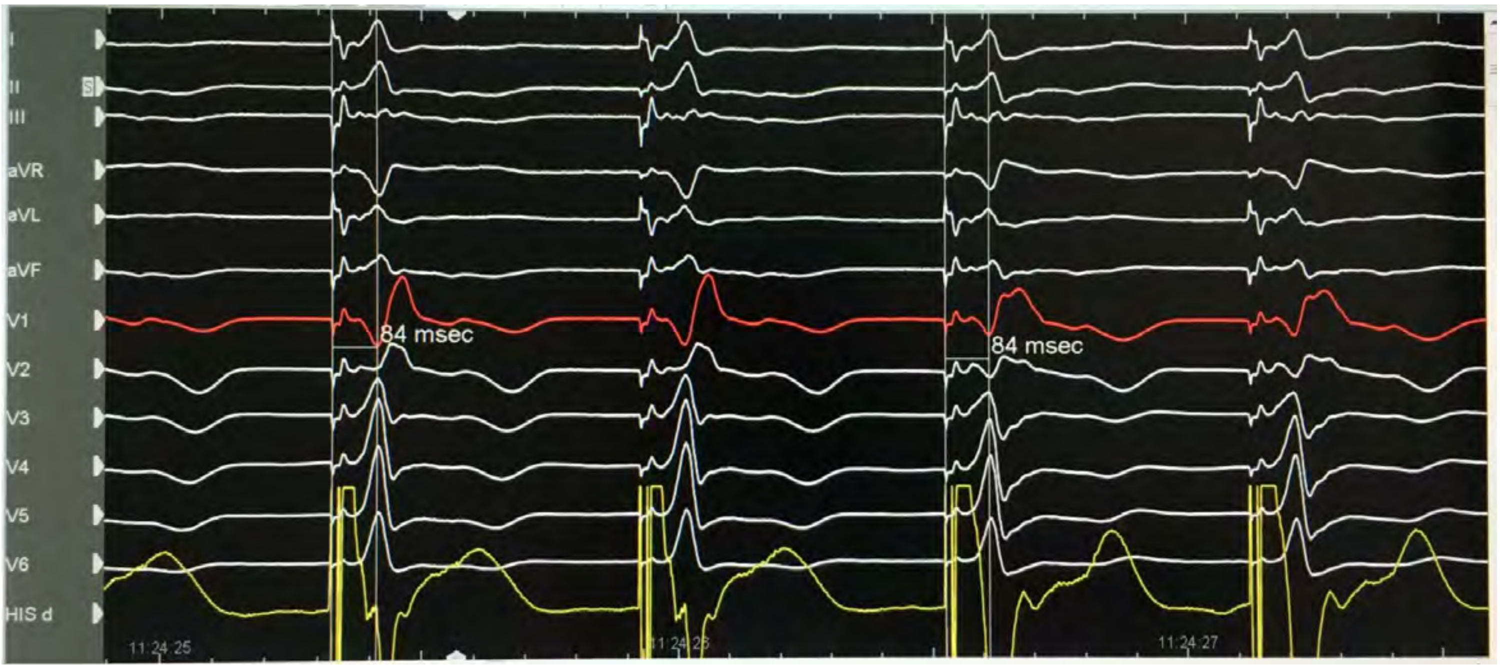

Transitions

Patient 22:
Pre-ECG

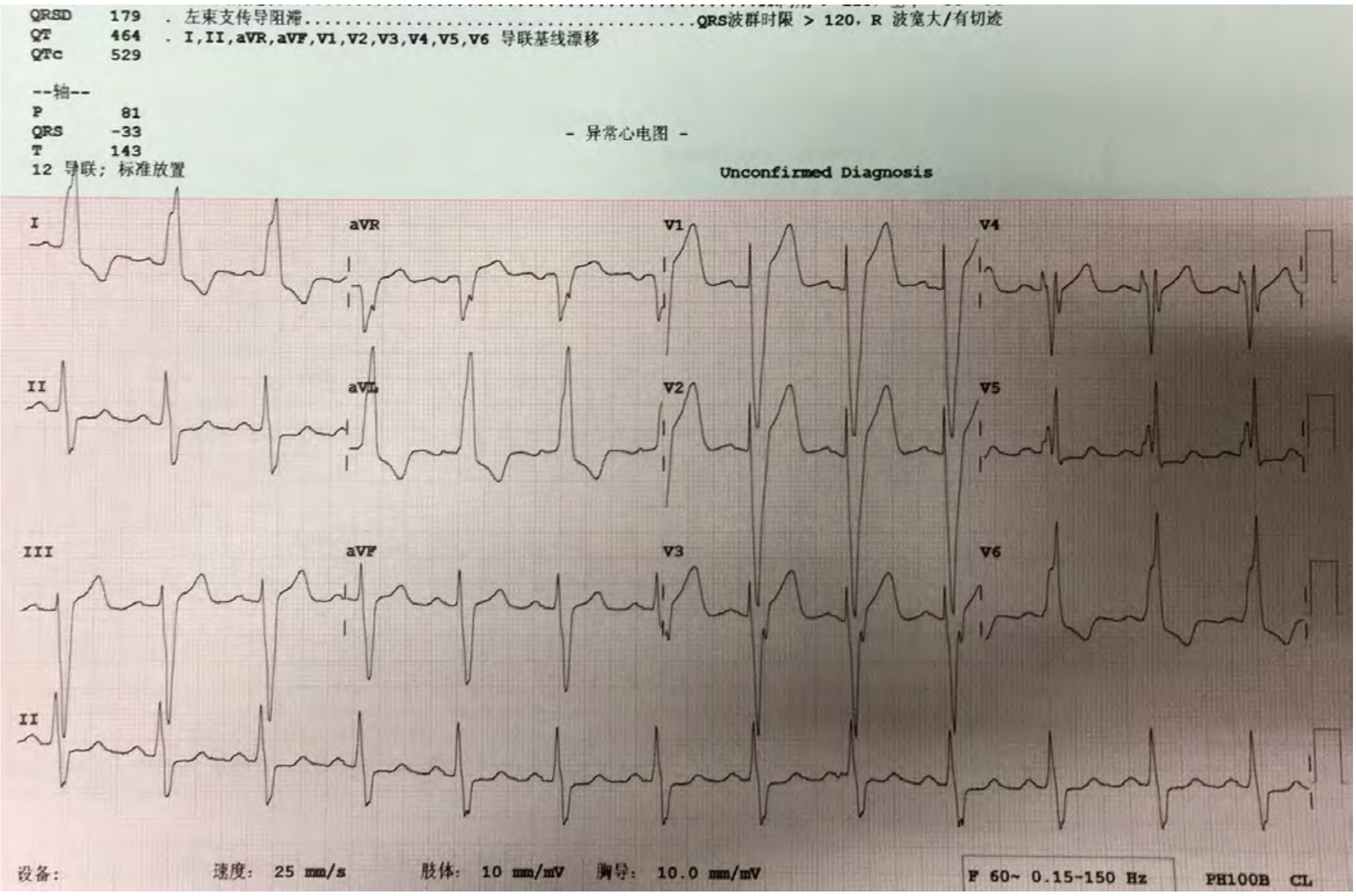

Post ECG

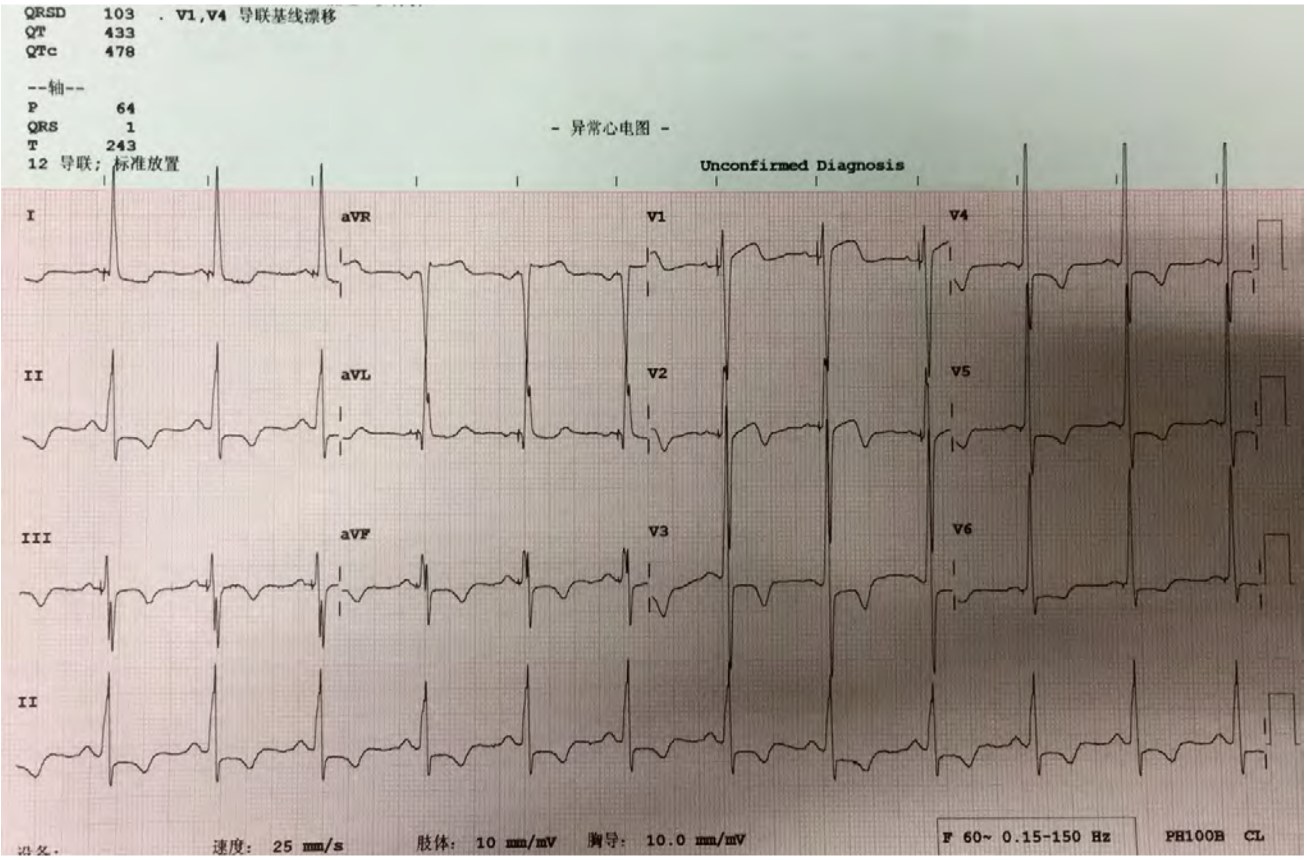

Patient 23:
Pre-ECG

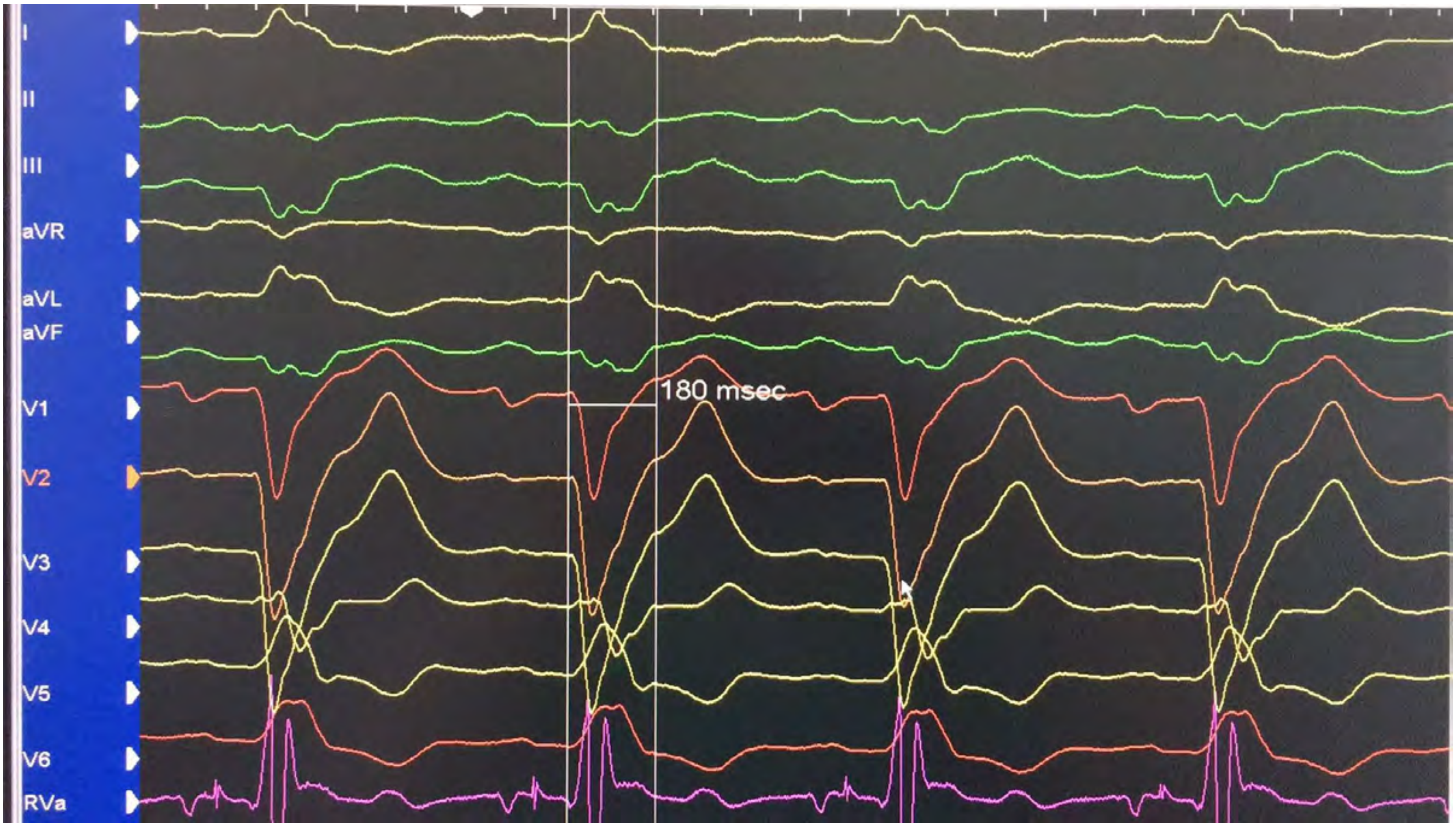

Post ECG

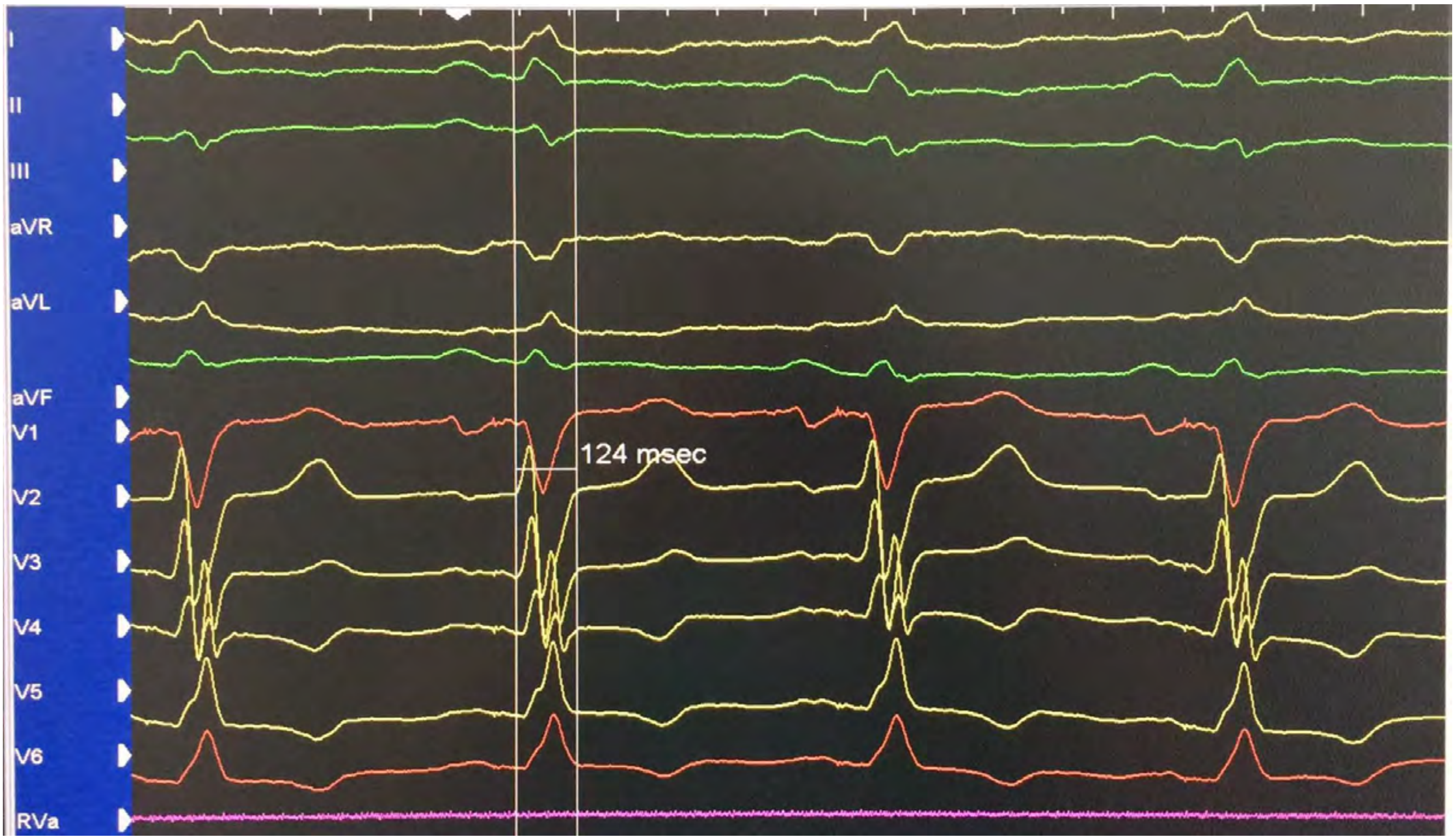

Patient 23:
Transitions

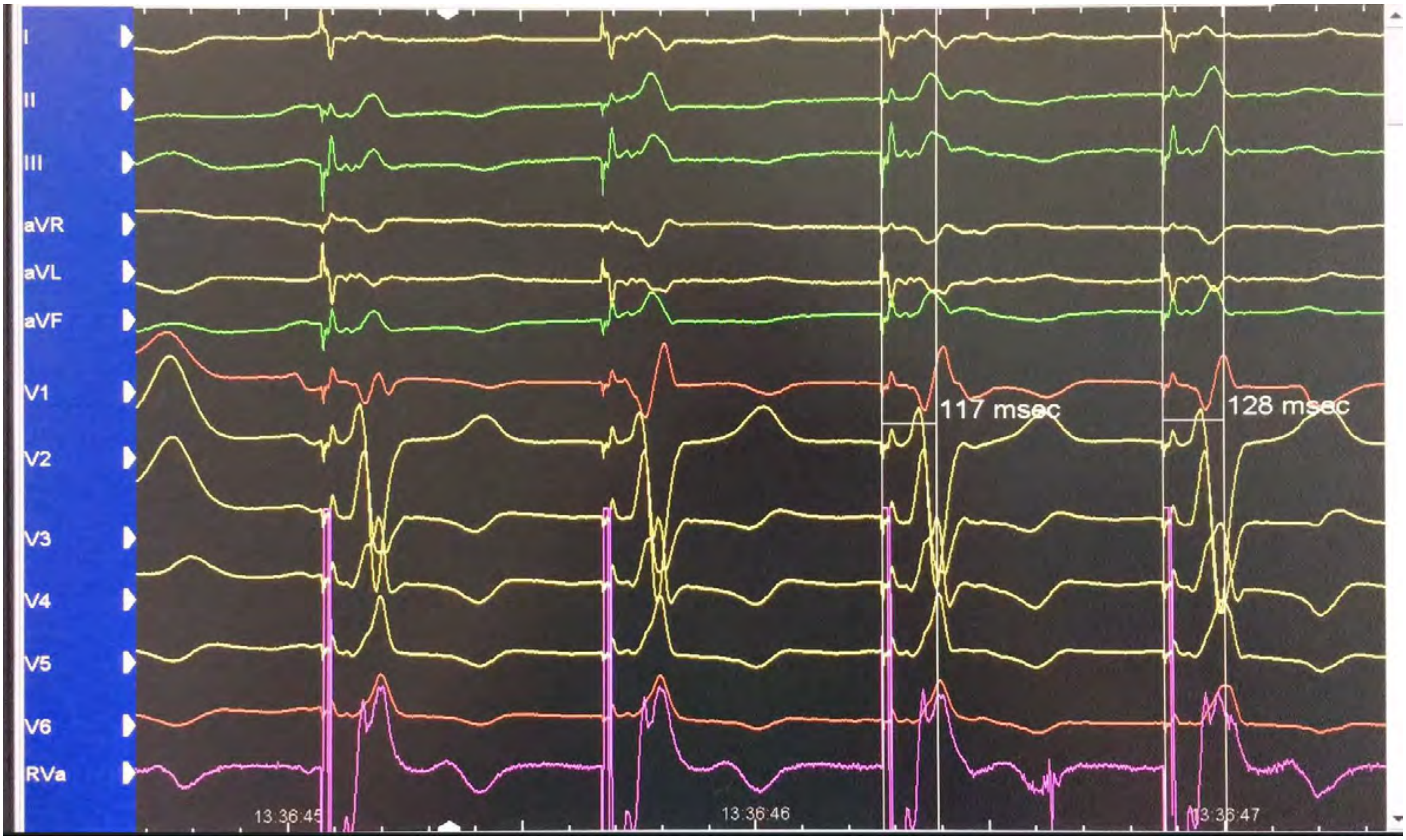

Transitions

Patient 24:
Pre-ECG

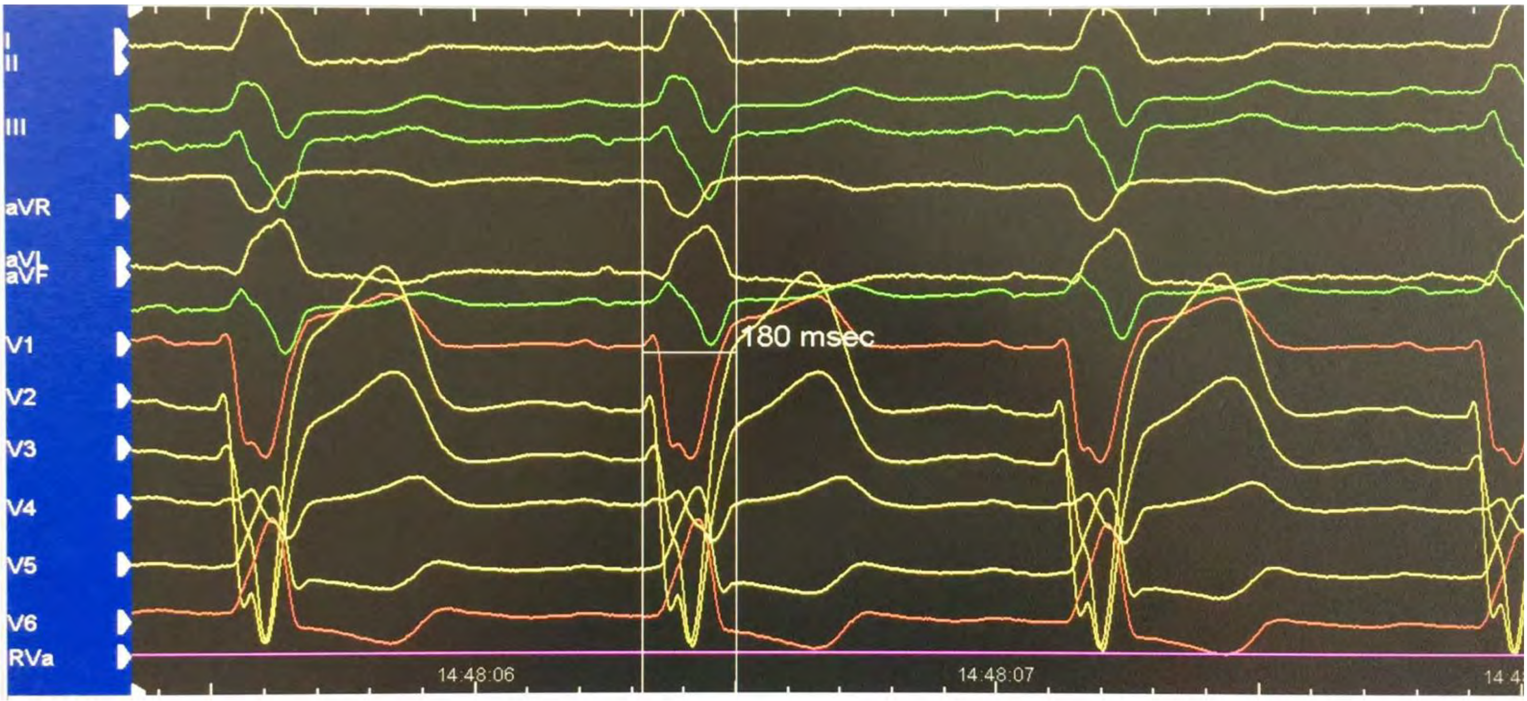

Post ECG

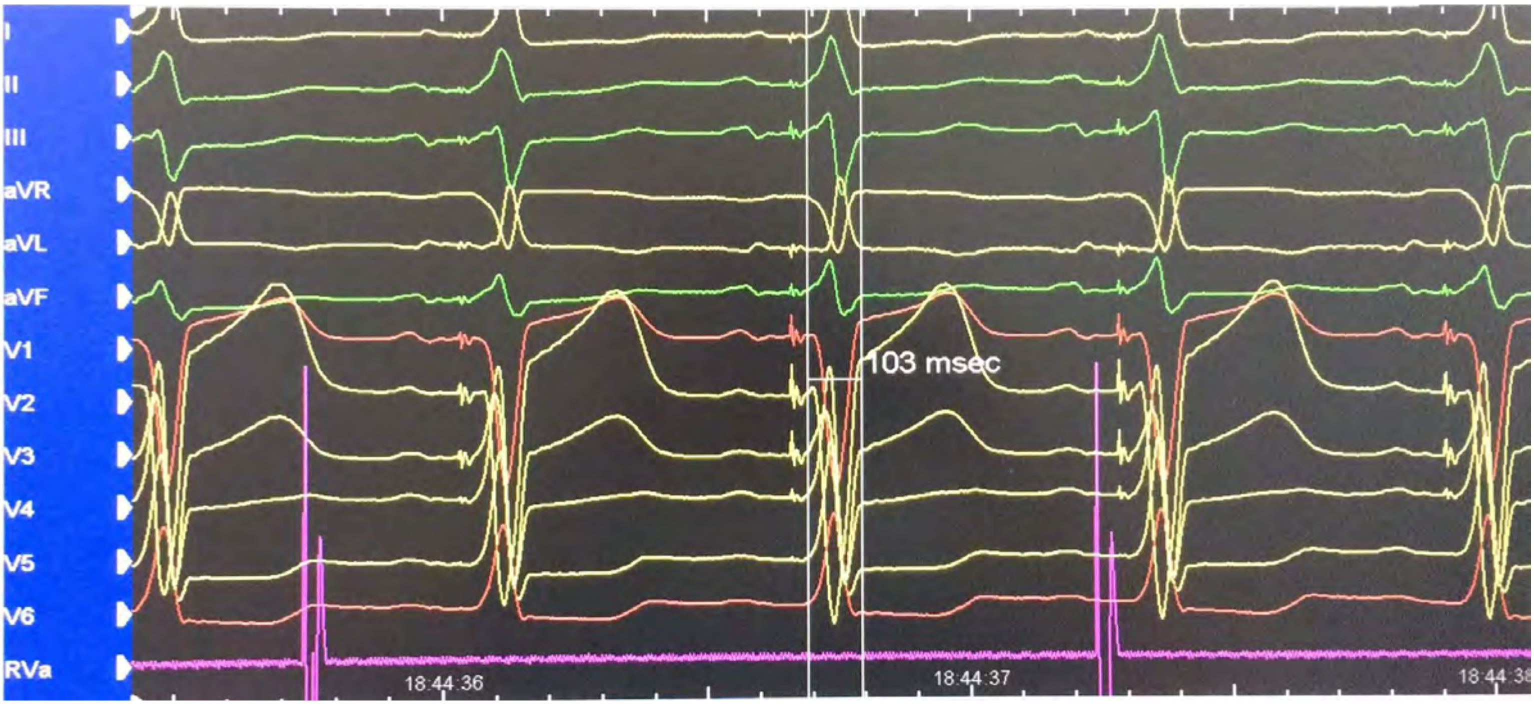

Patient 24:
Transitions

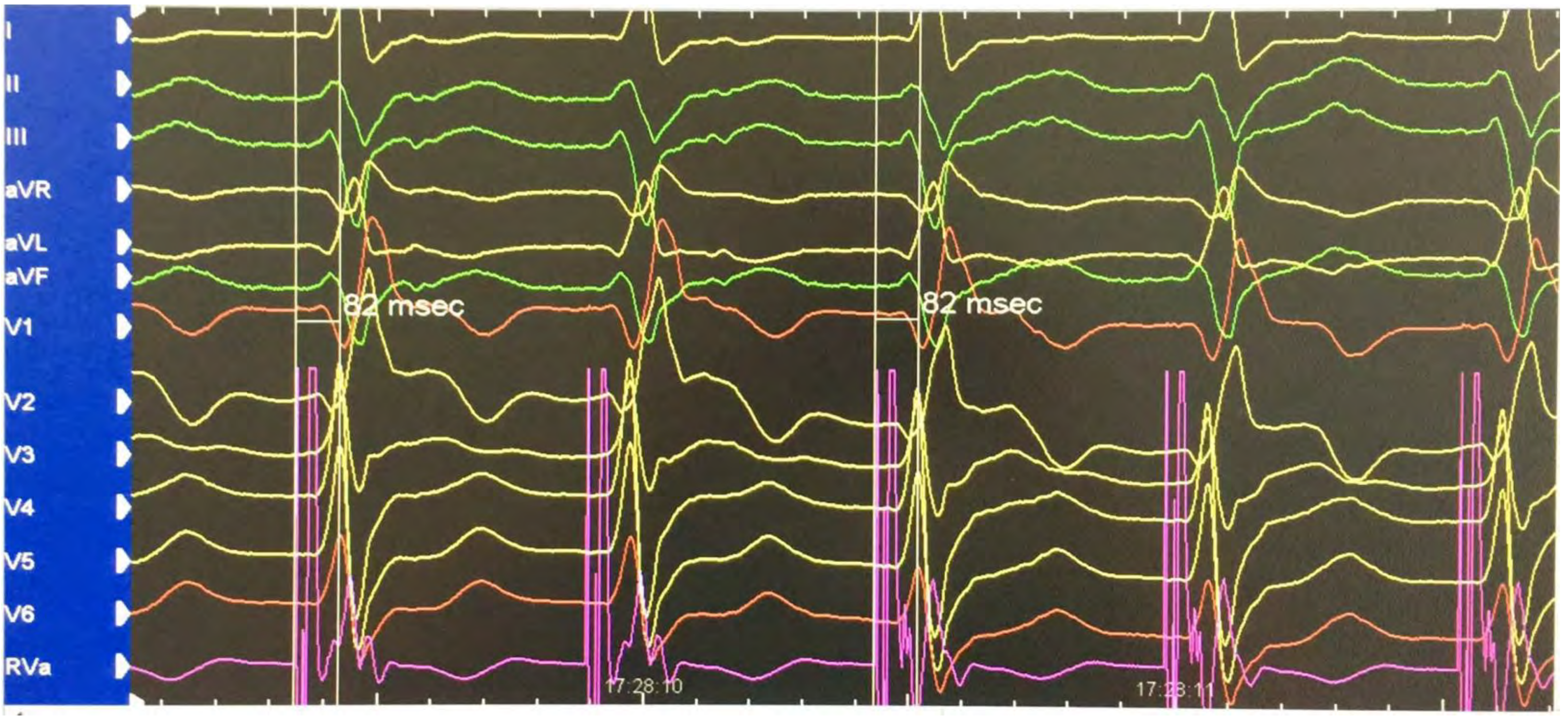

Transitions

Patient 25:
Pre-ECG

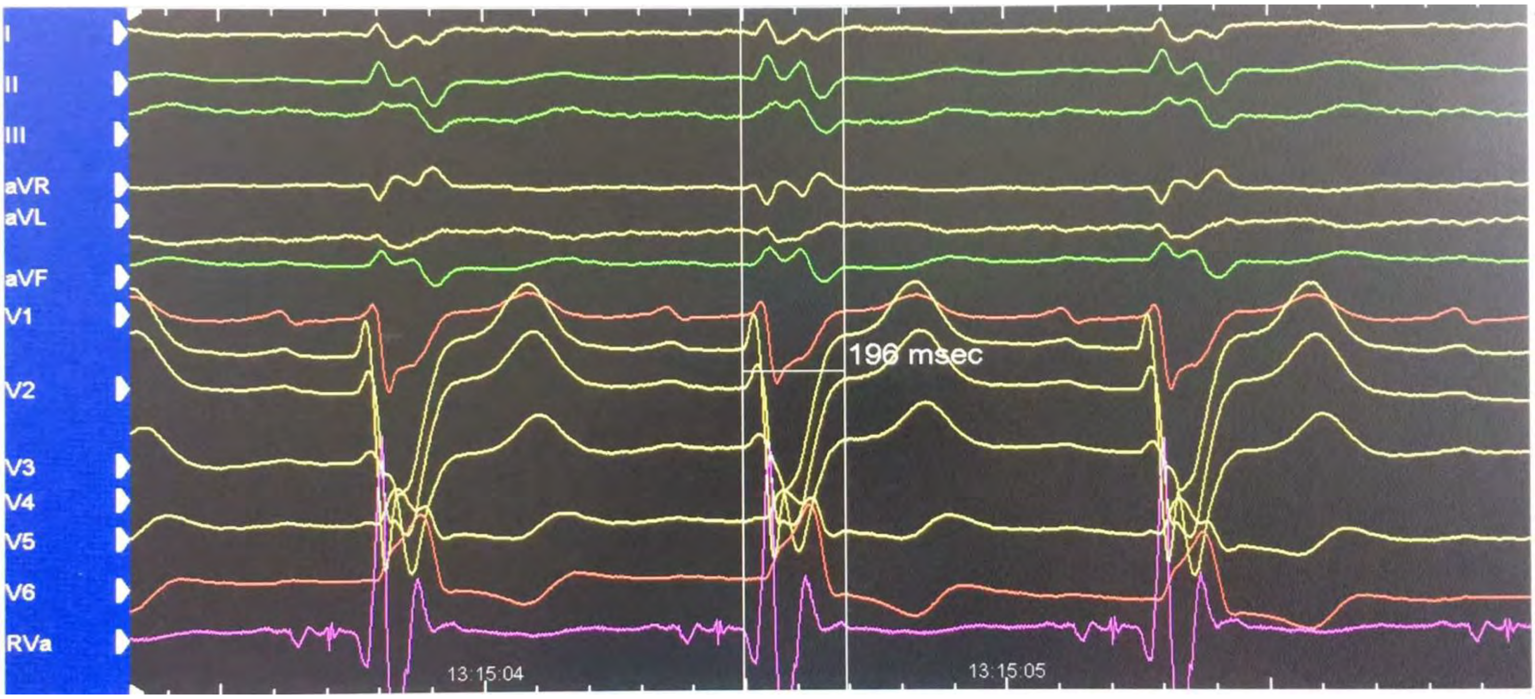

Transitions

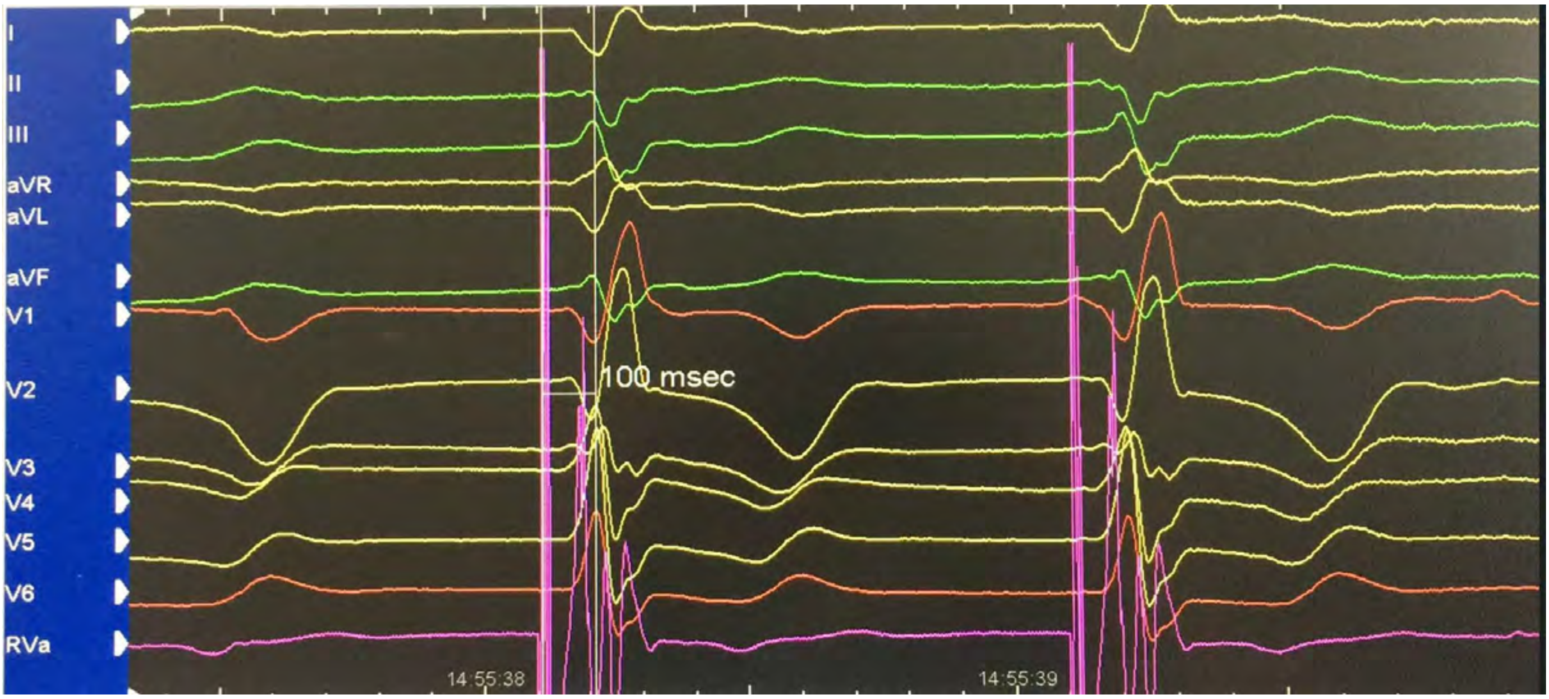

Patient 26:
Pre-ECG

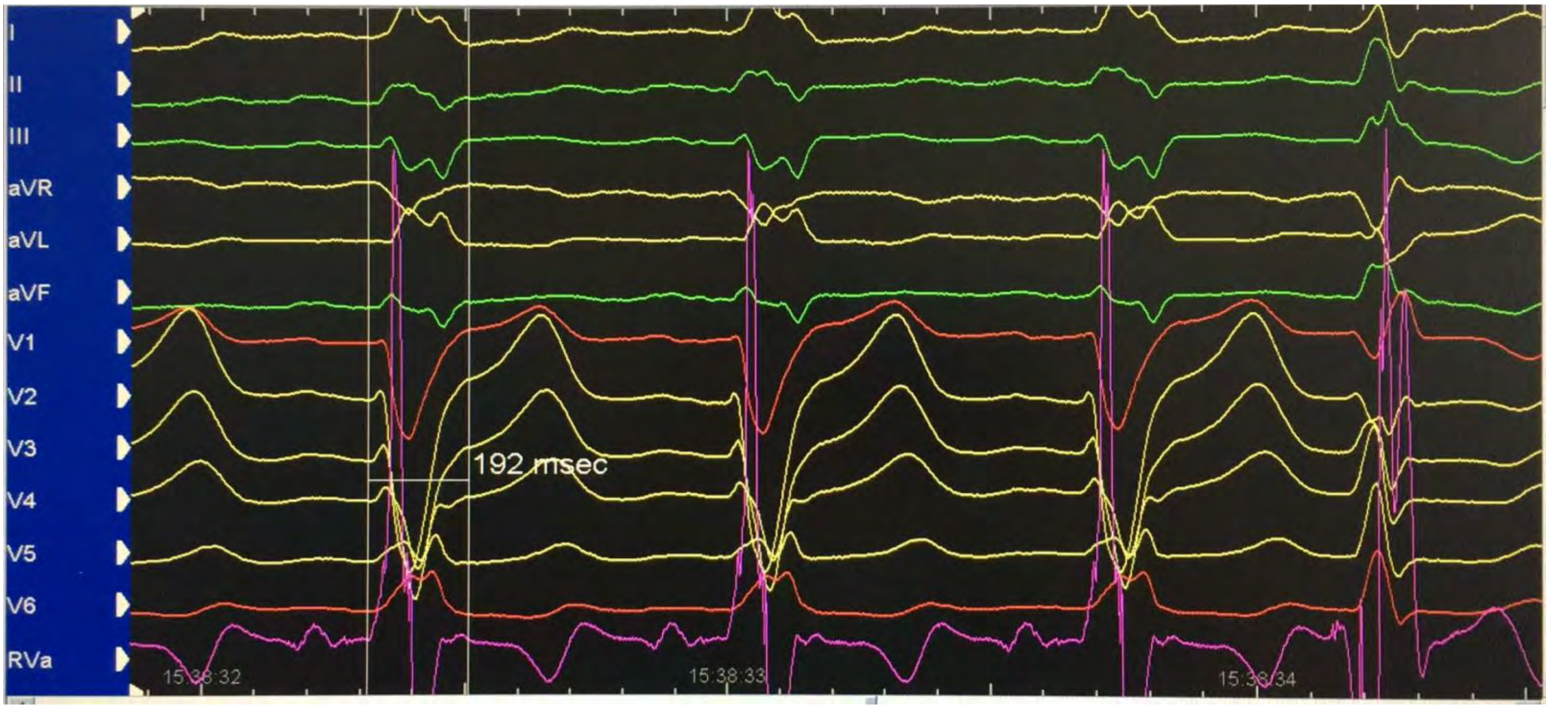

Post ECG

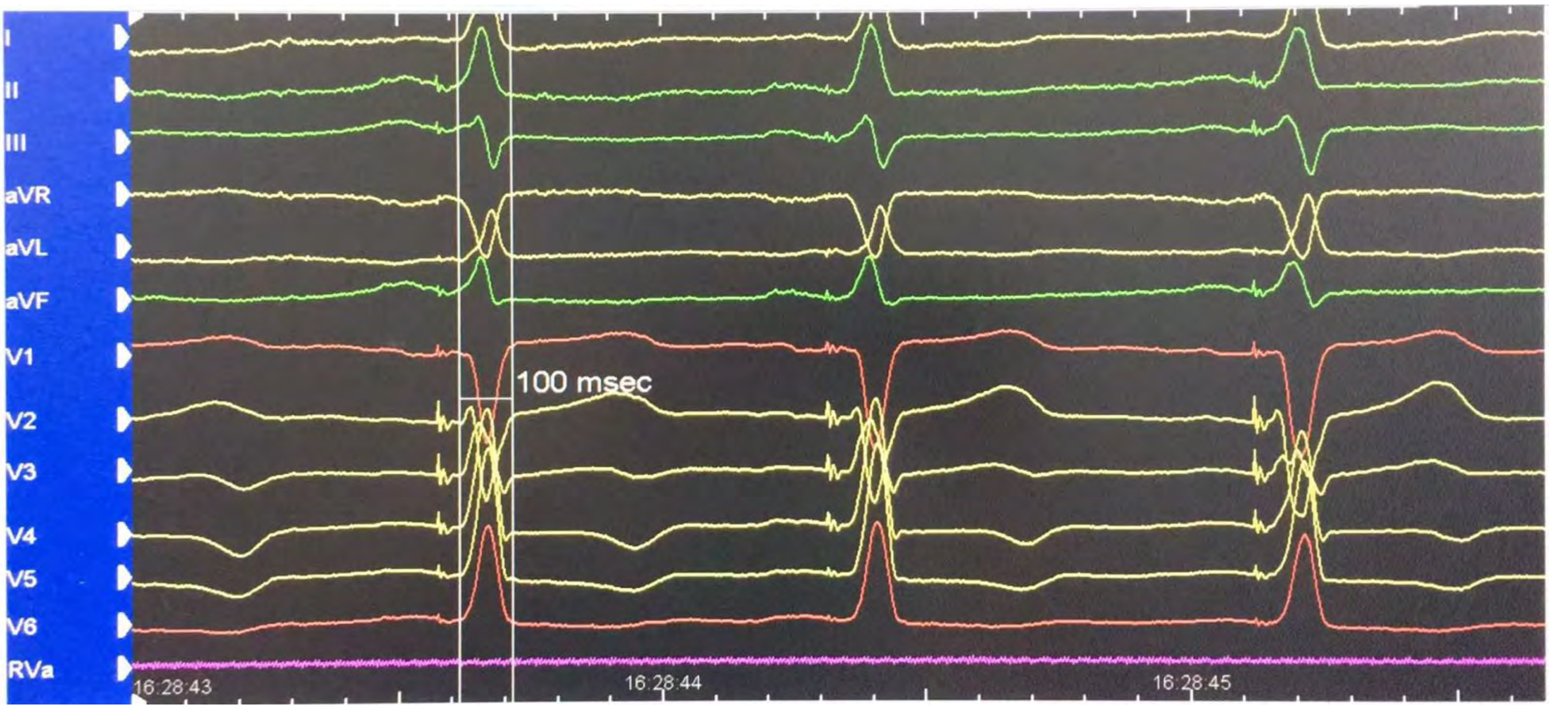

Patient 26:
Transitions

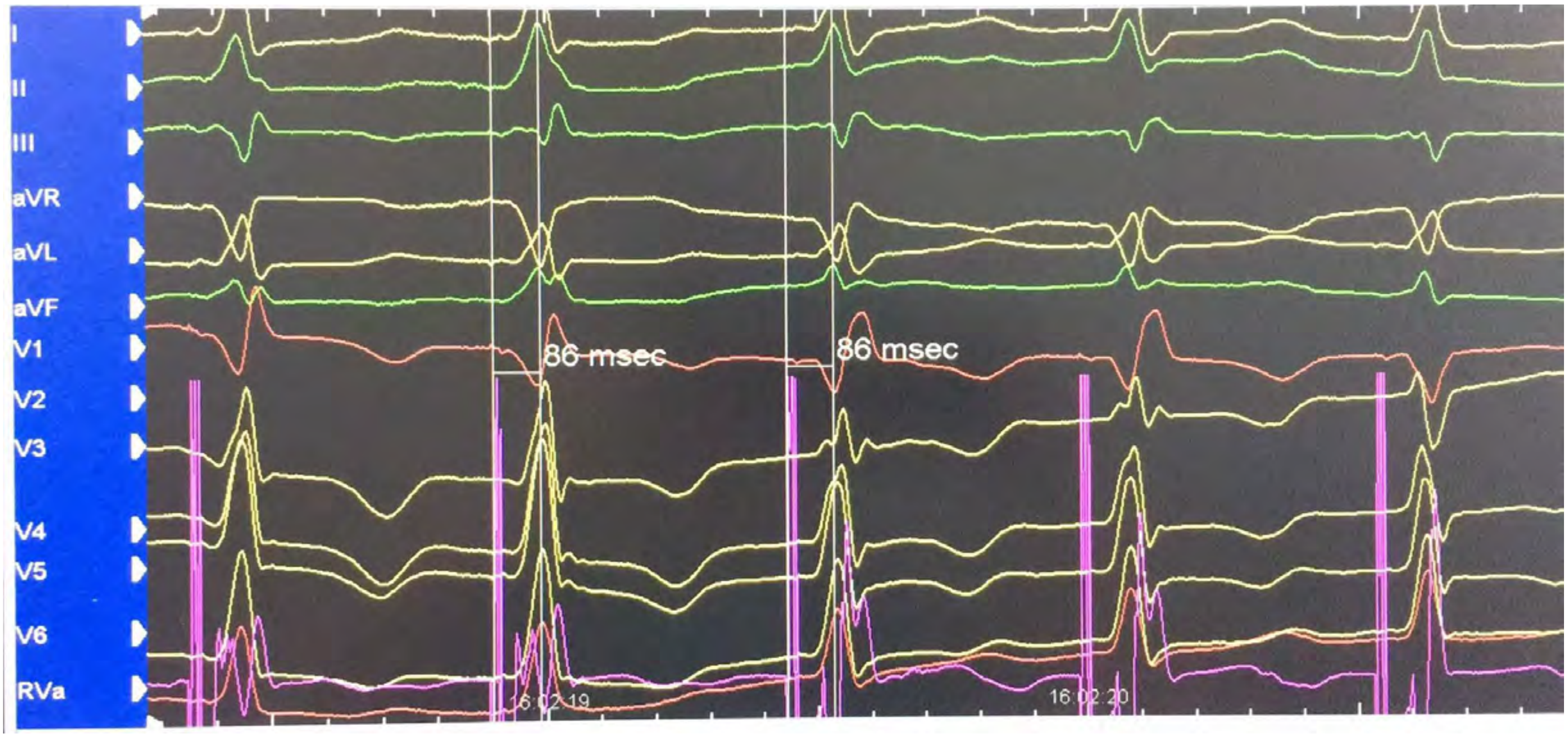

Transitions

Patient 27:
Pre-ECG

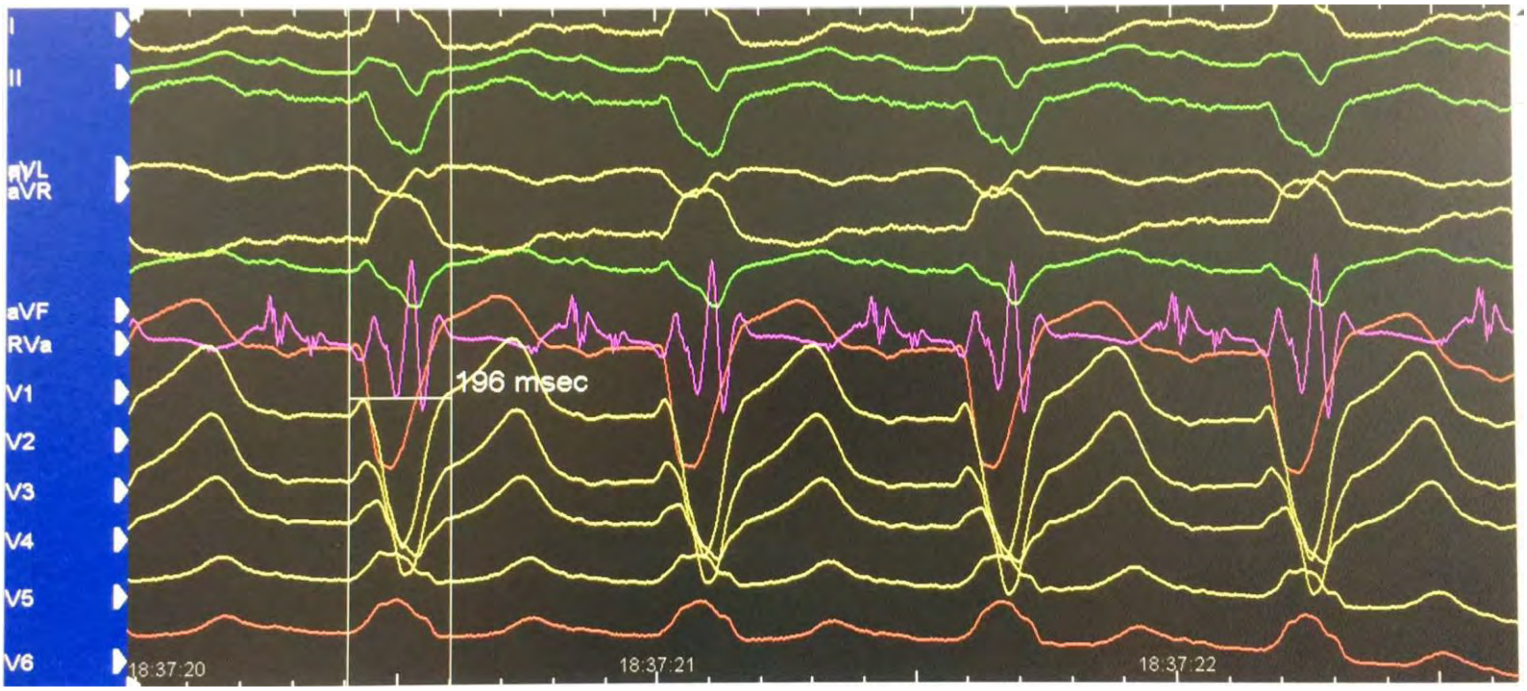

Post ECG

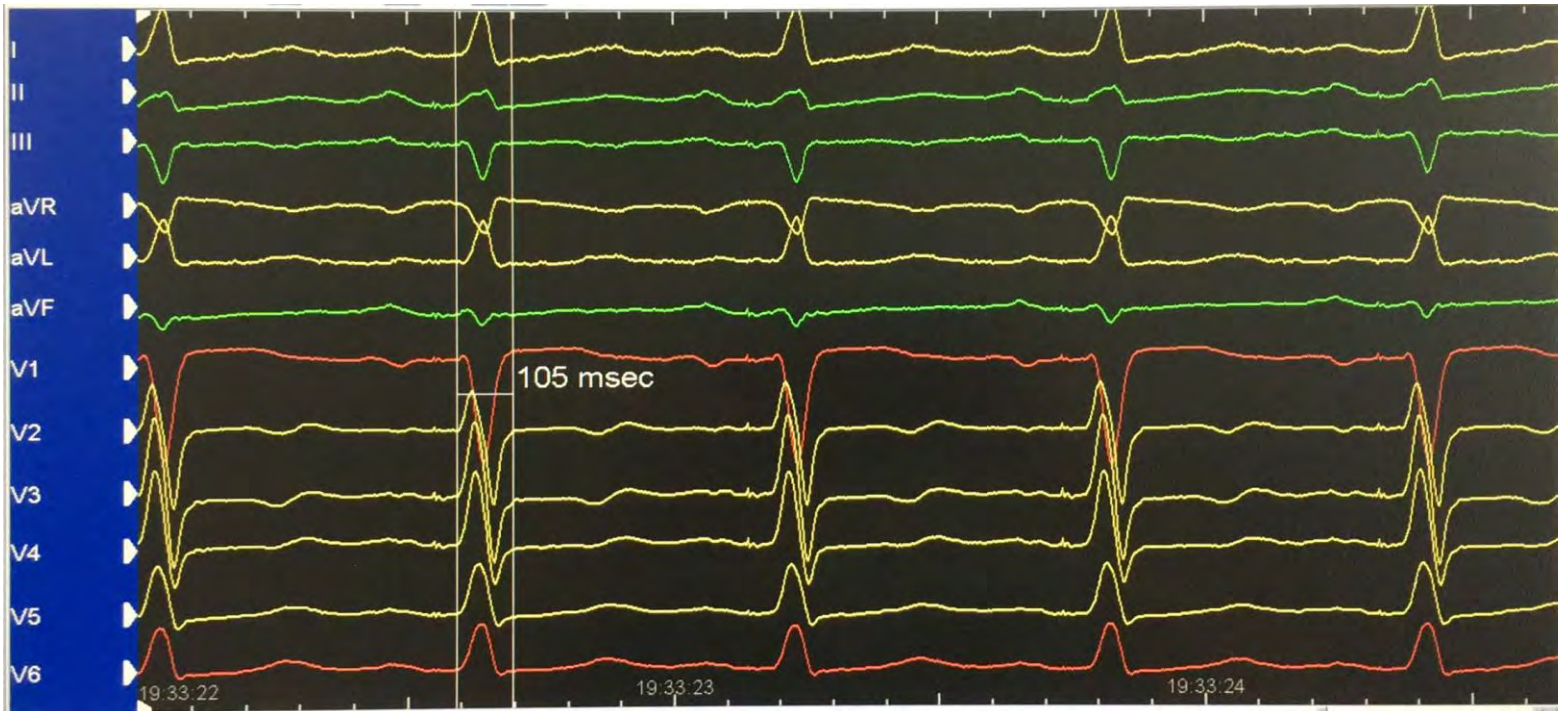

Patient 27:
Transitions

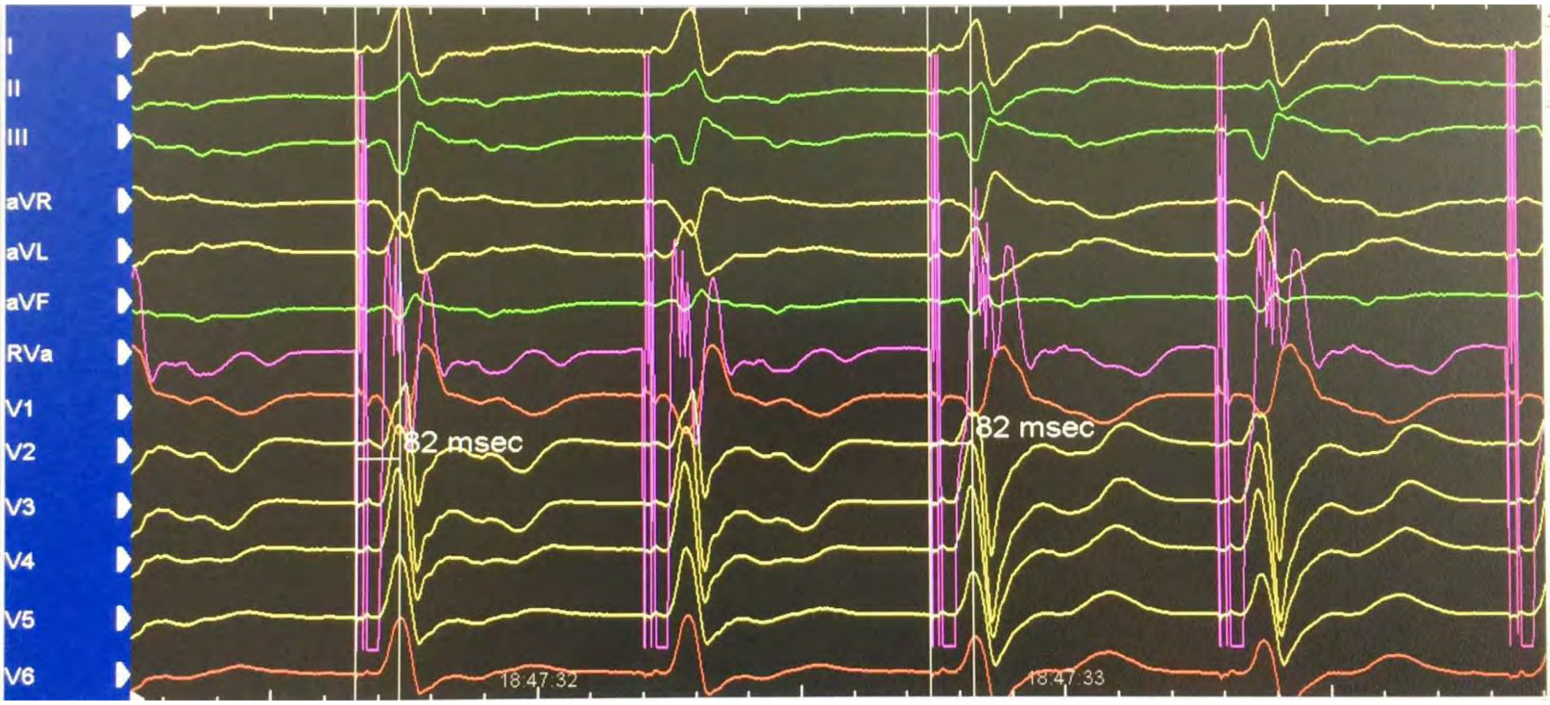

Transitions

Patient 28:
Pre-ECG

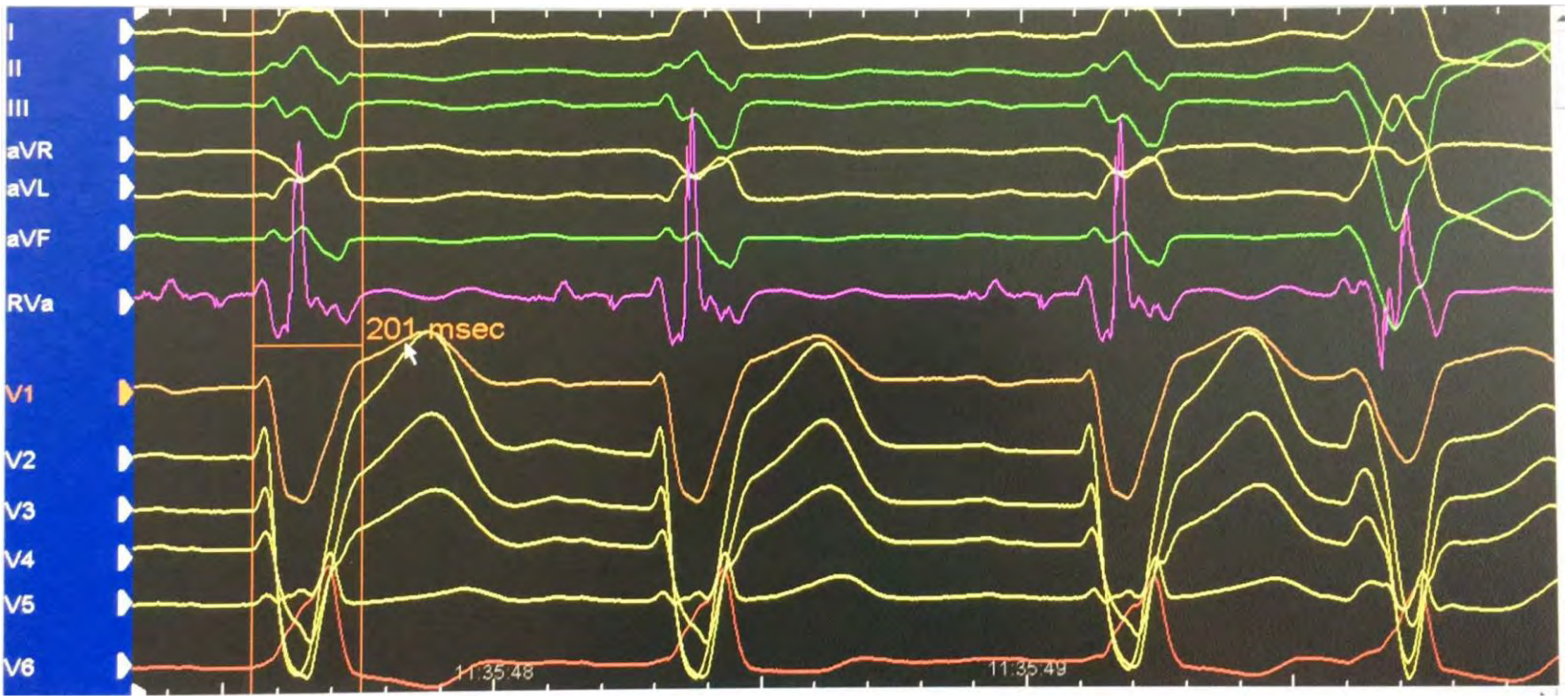

Post ECG

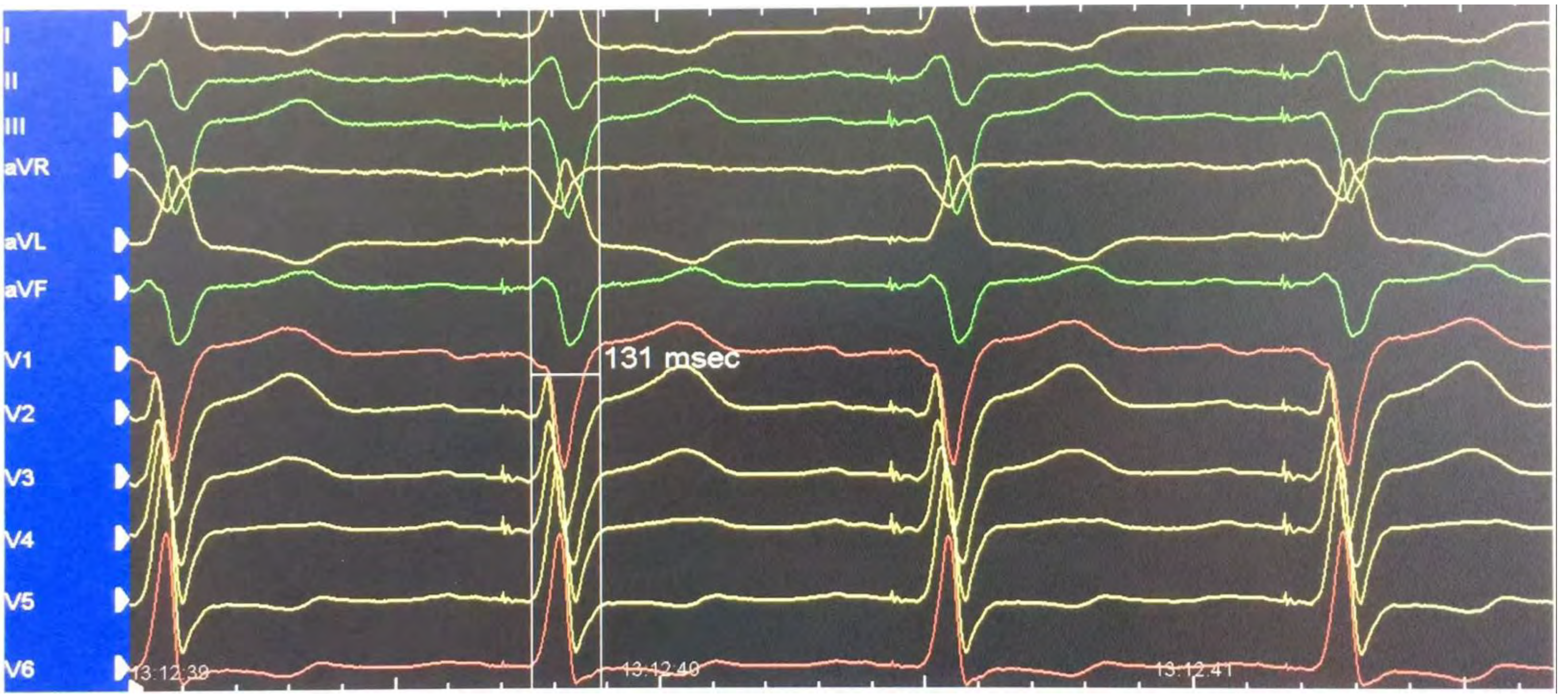

Patient 28:
Transitions

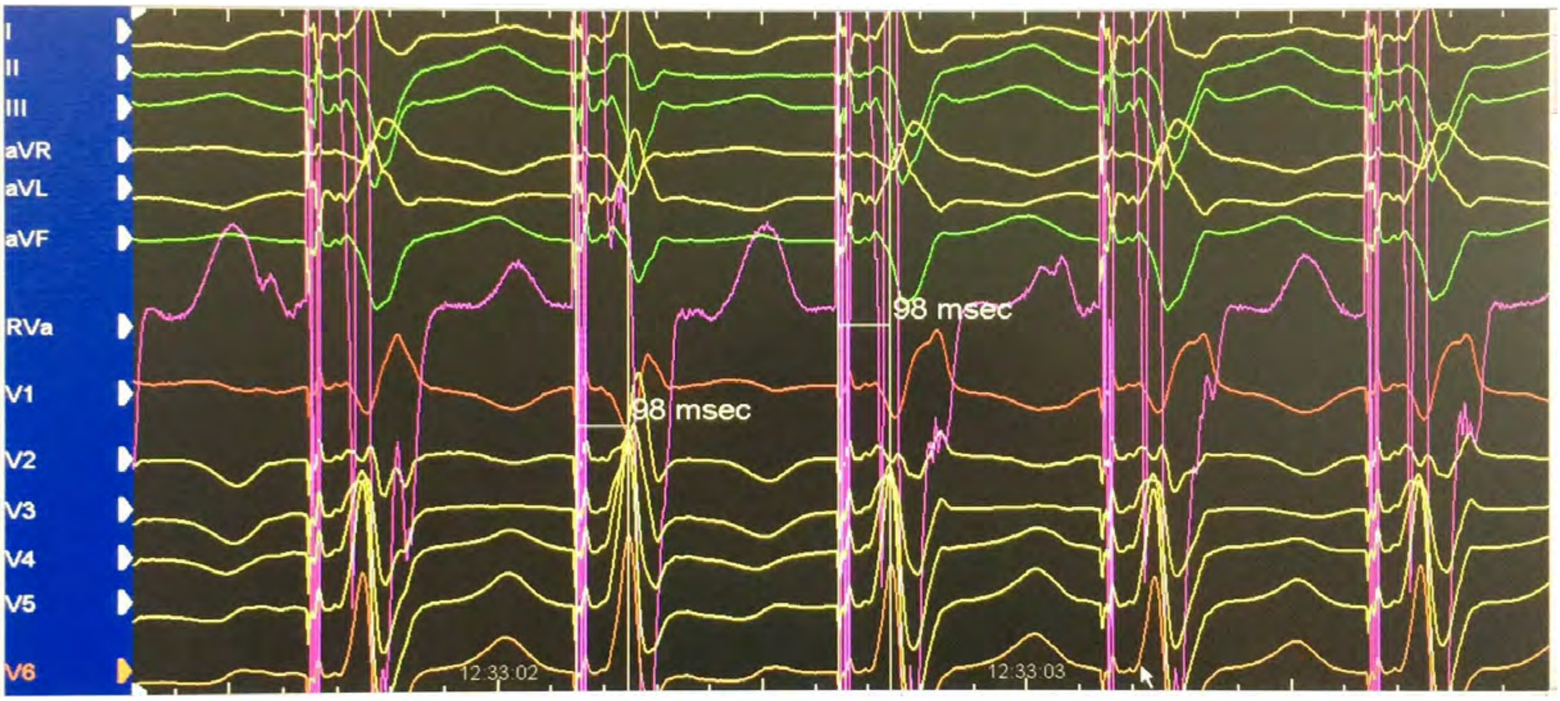

Transitions

Patient 29:
Pre-ECG

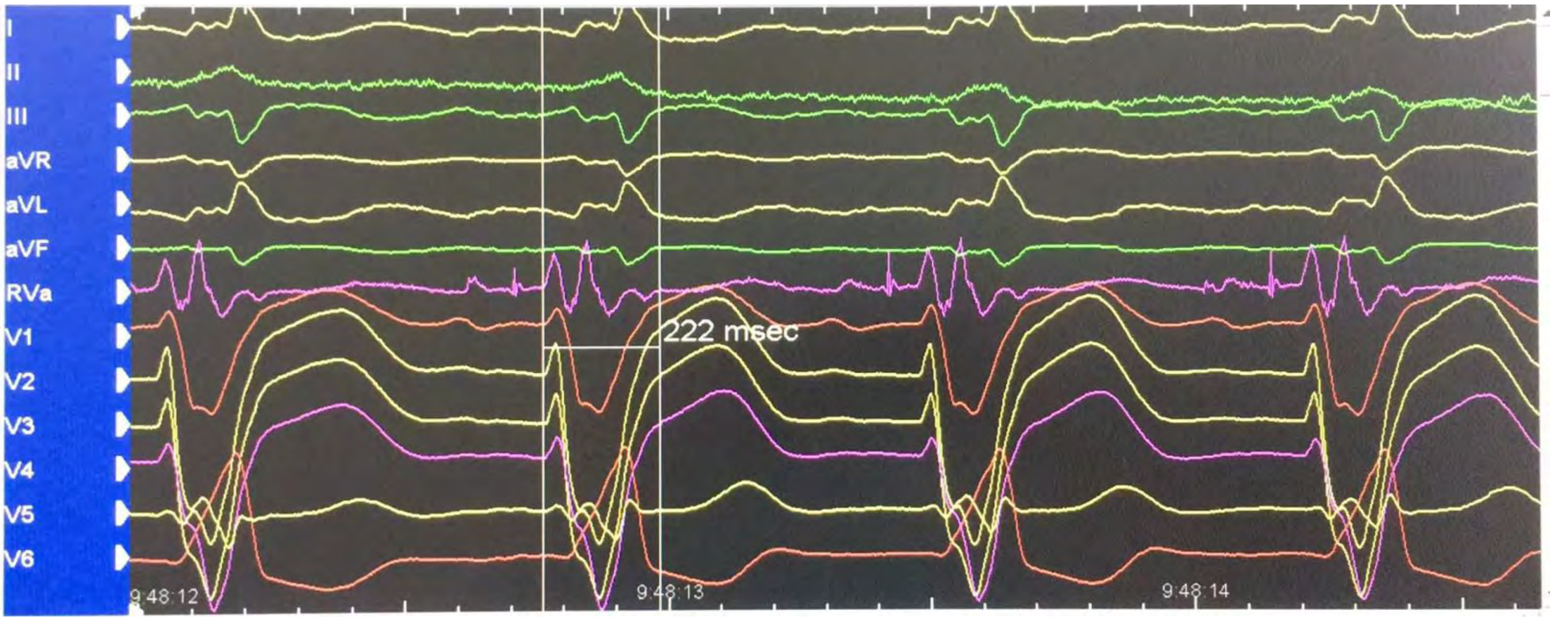

Transitions

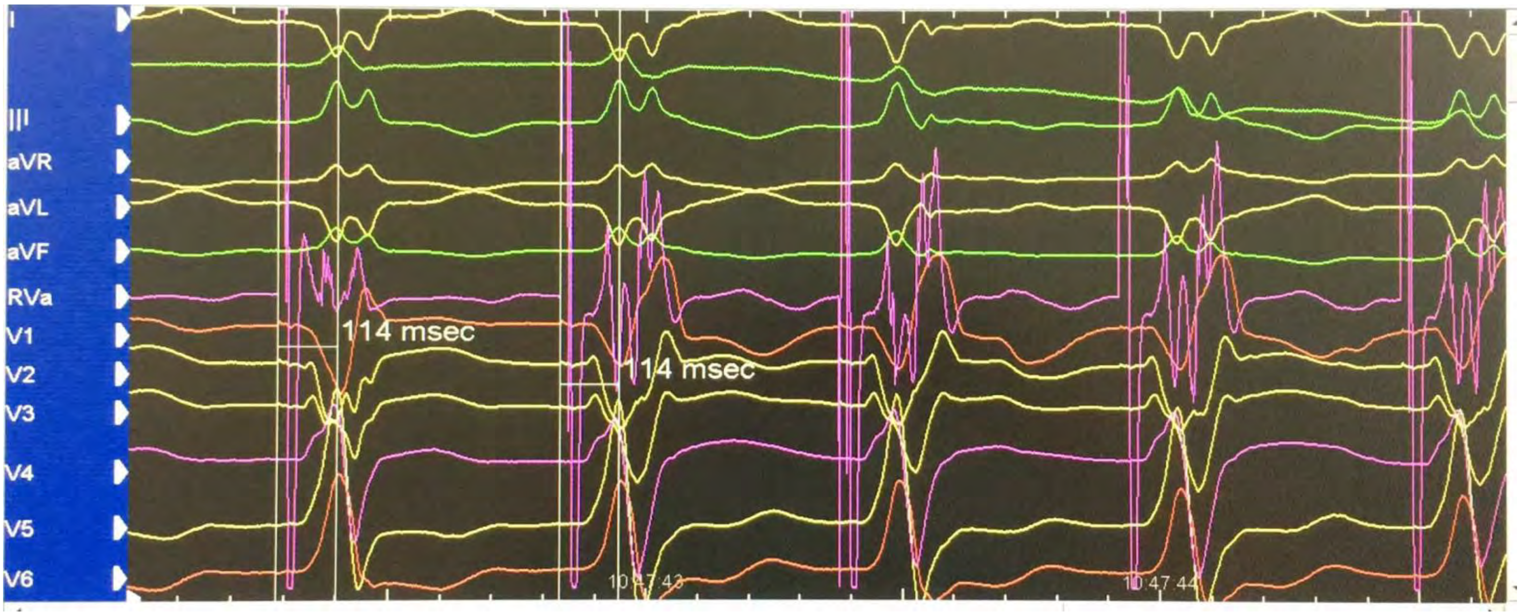

Patient 30:
Pre-ECG

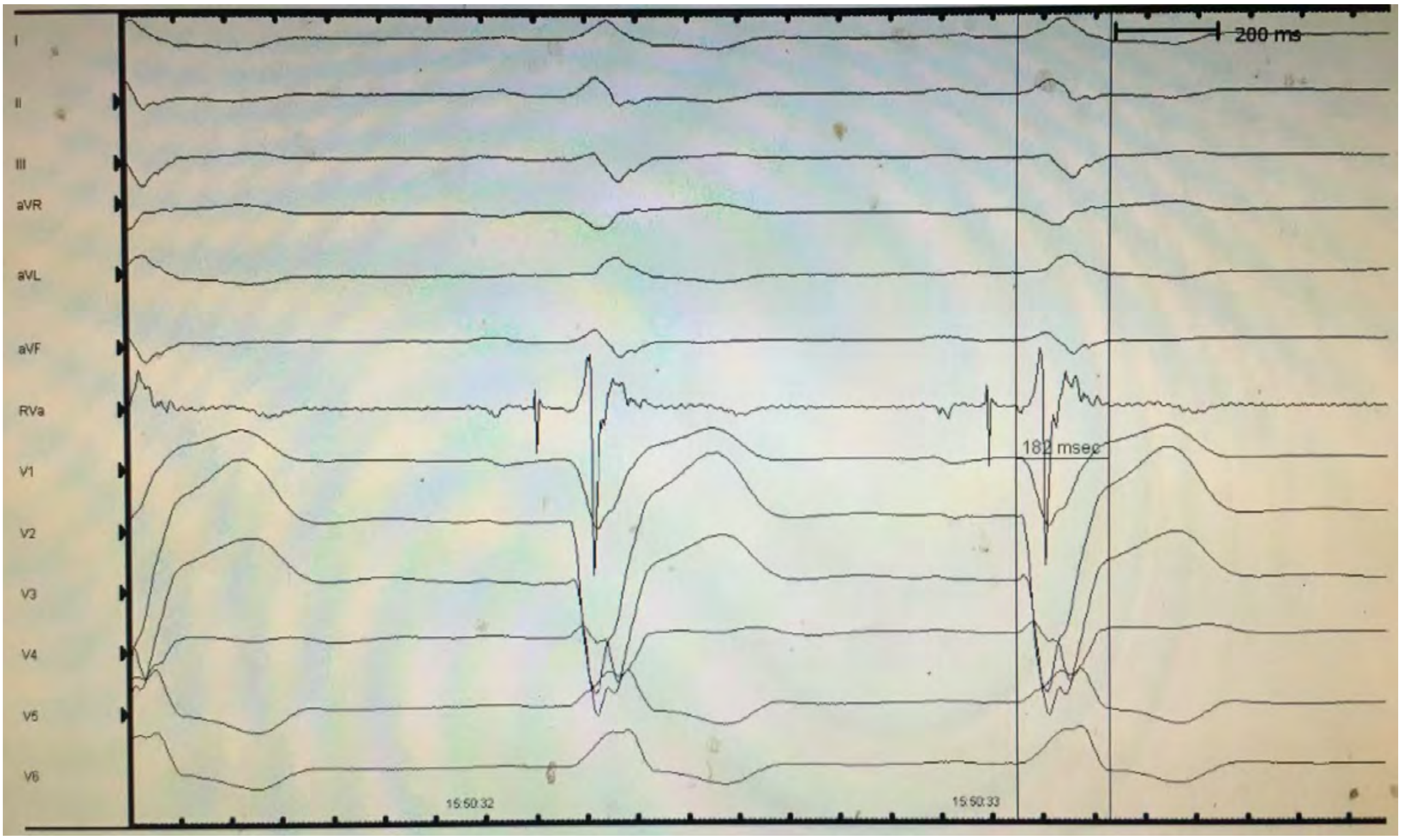

Post ECG

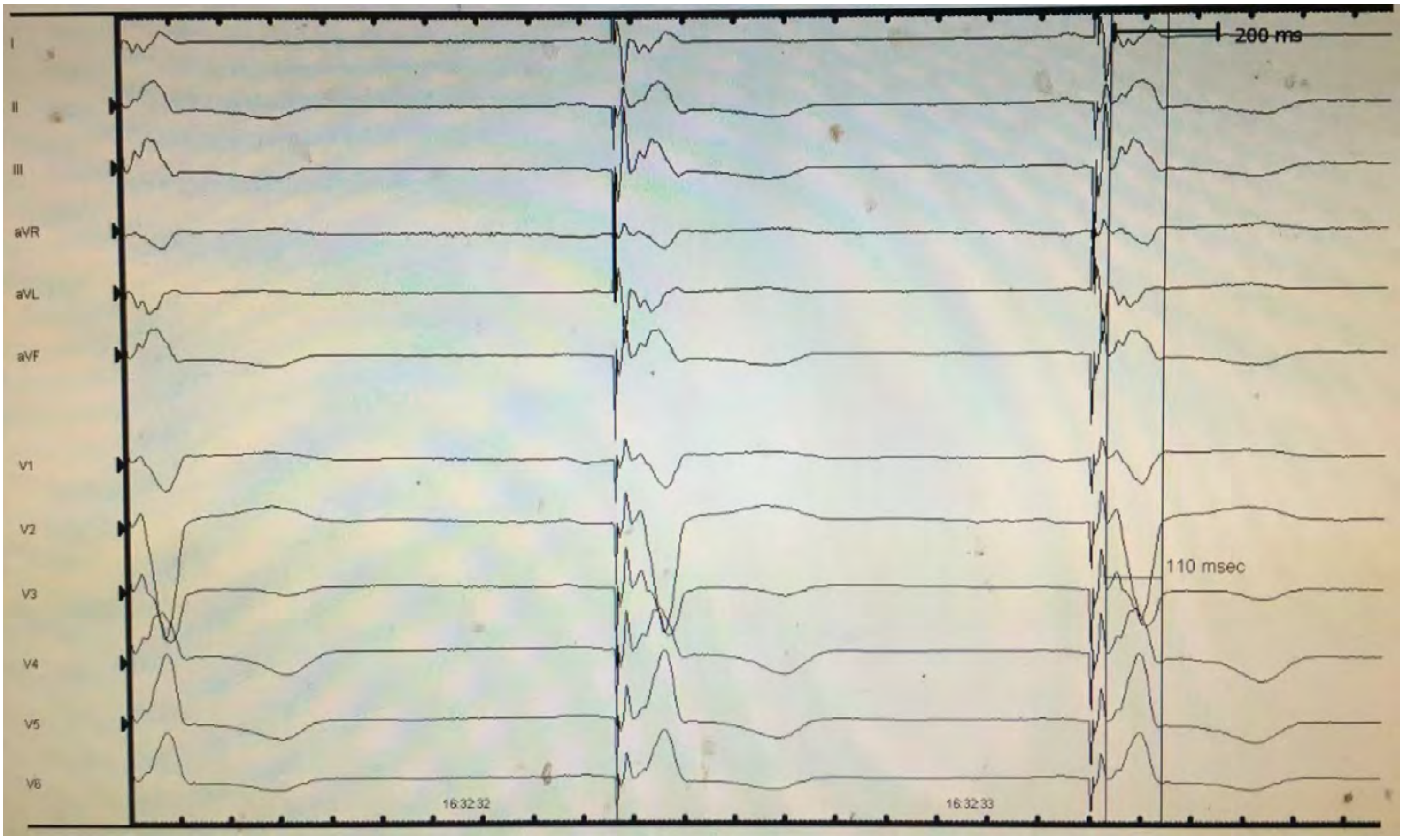

Patient 30:
Transitions

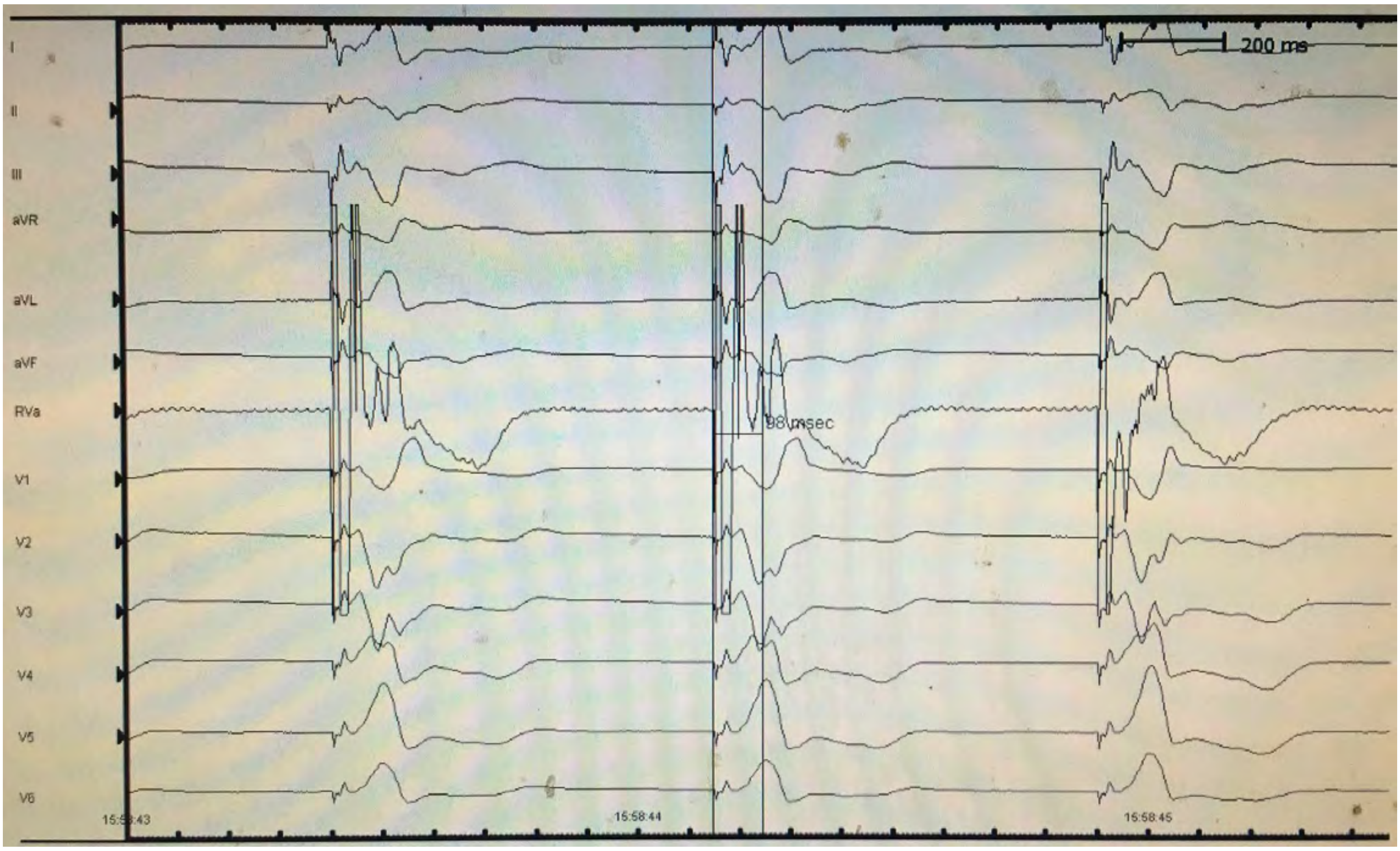

Transitions

Patient 31:
Pre-ECG

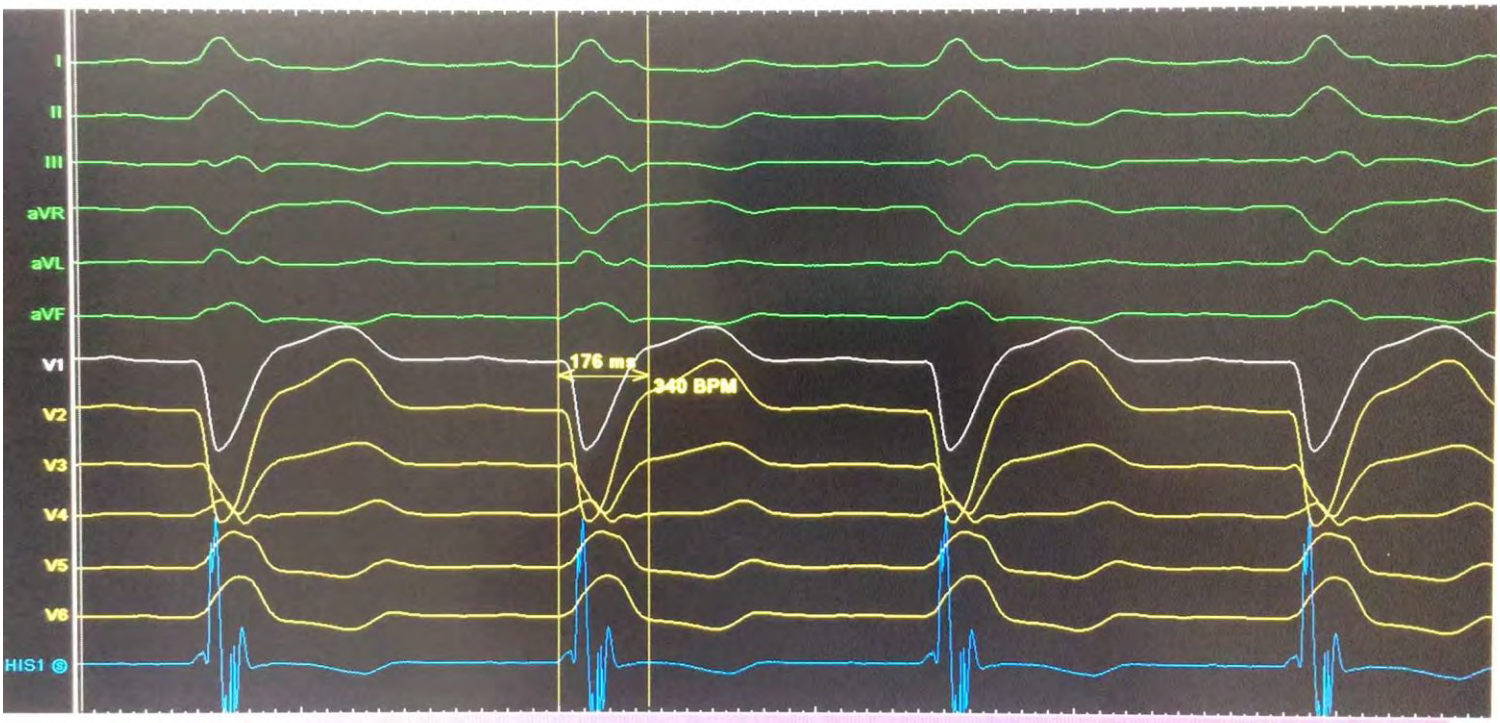

Transitions

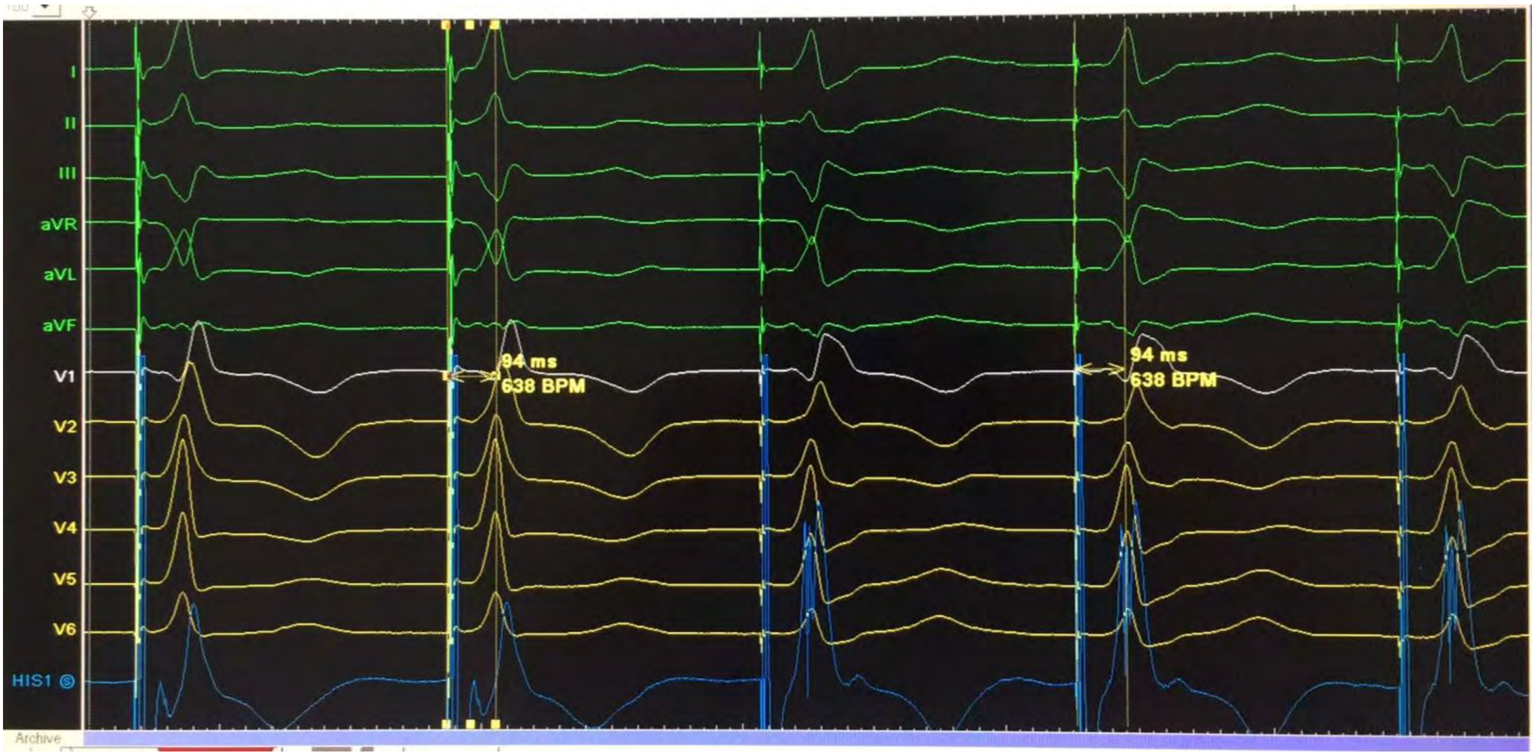

Patient 32:
Pre-ECG

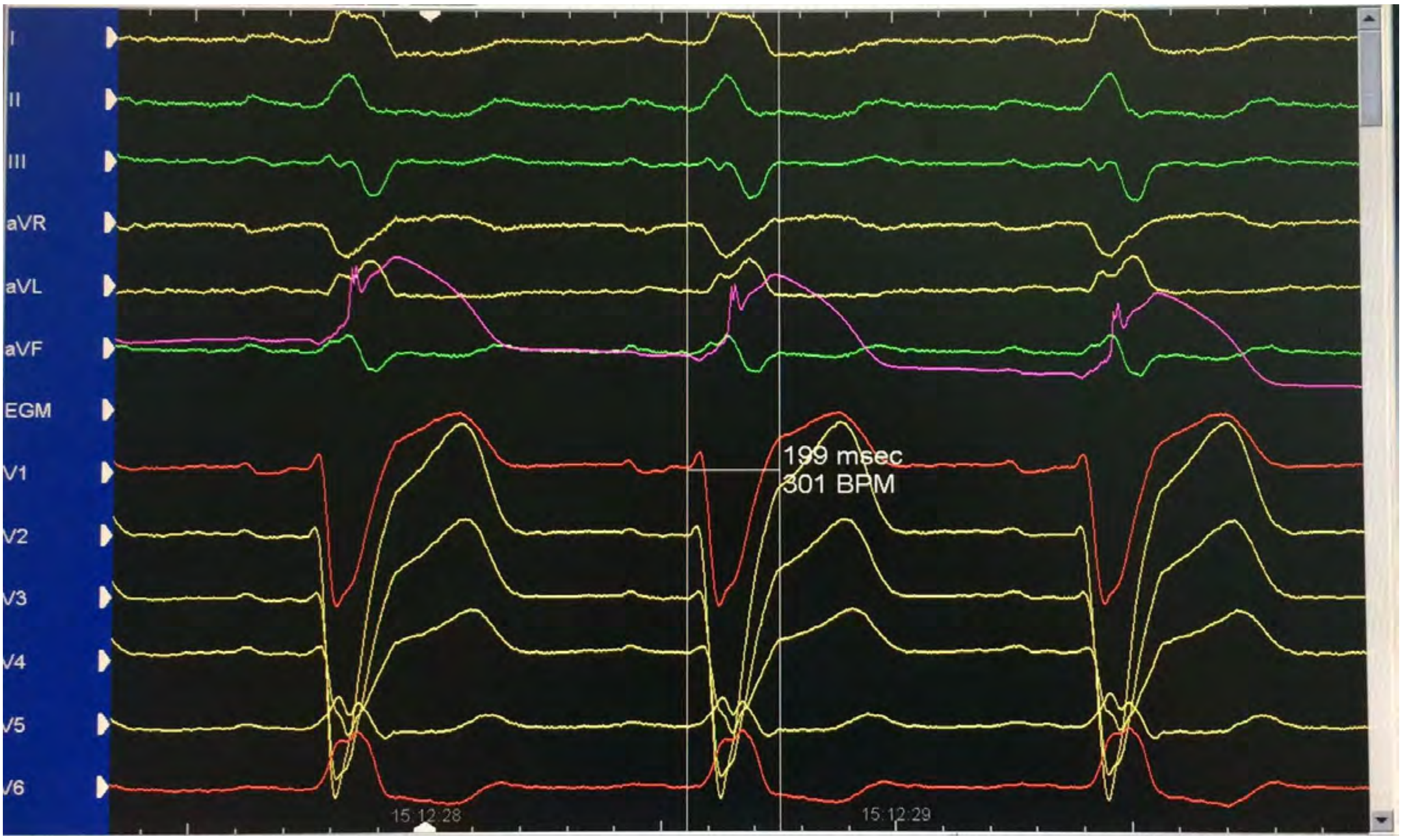

Post ECG

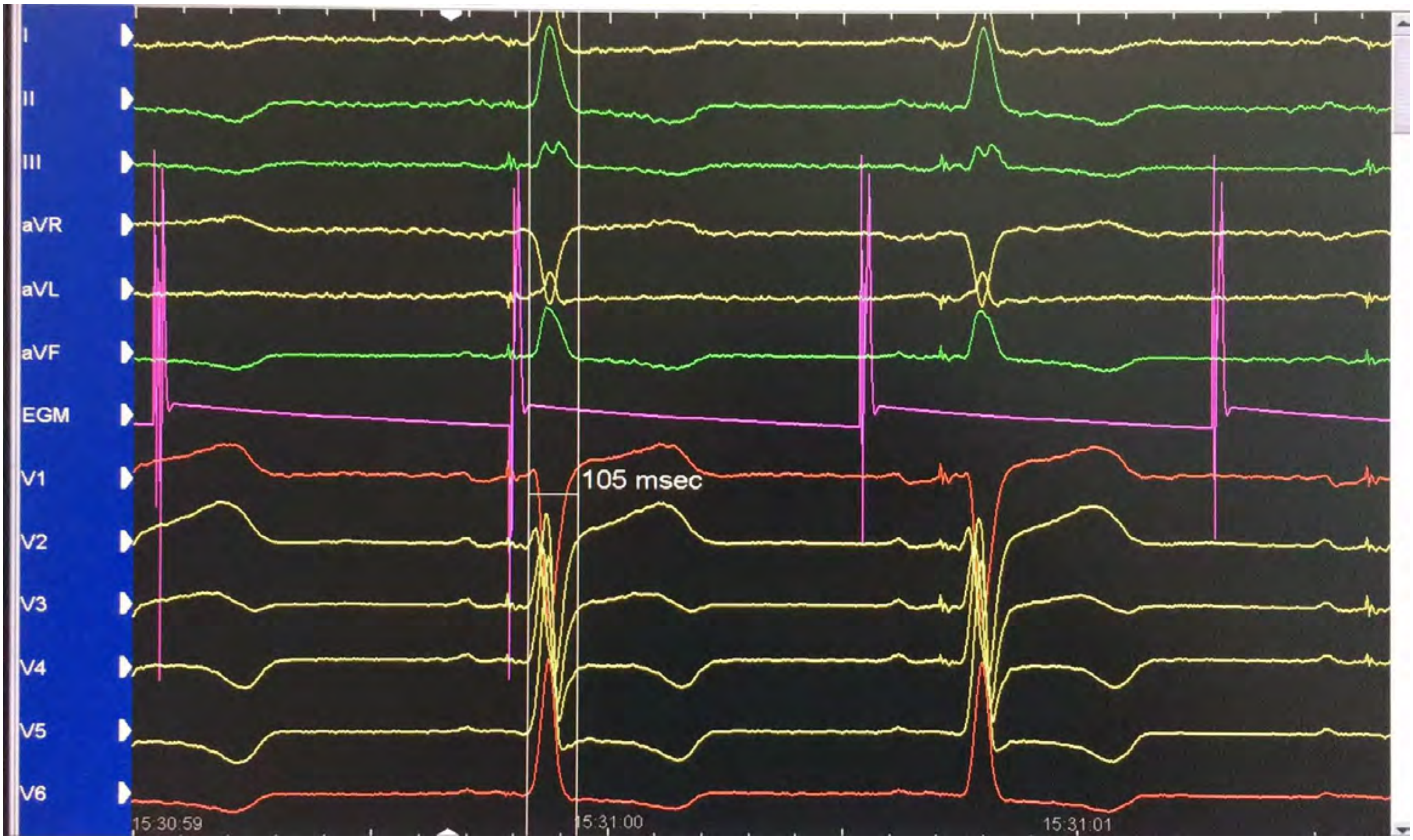

Patient 32:
Transitions

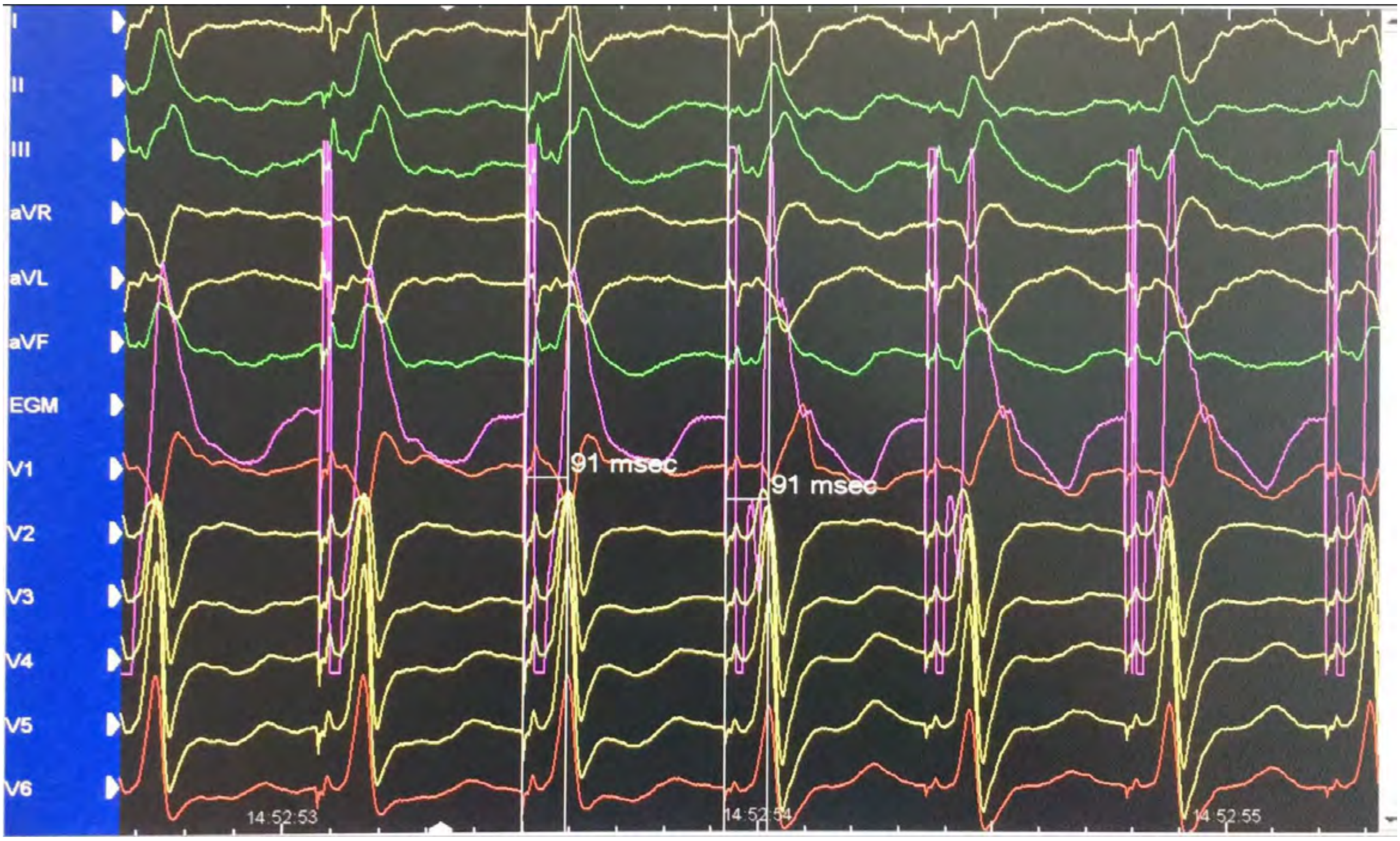

Transitions

Patient 33:
Pre-ECG

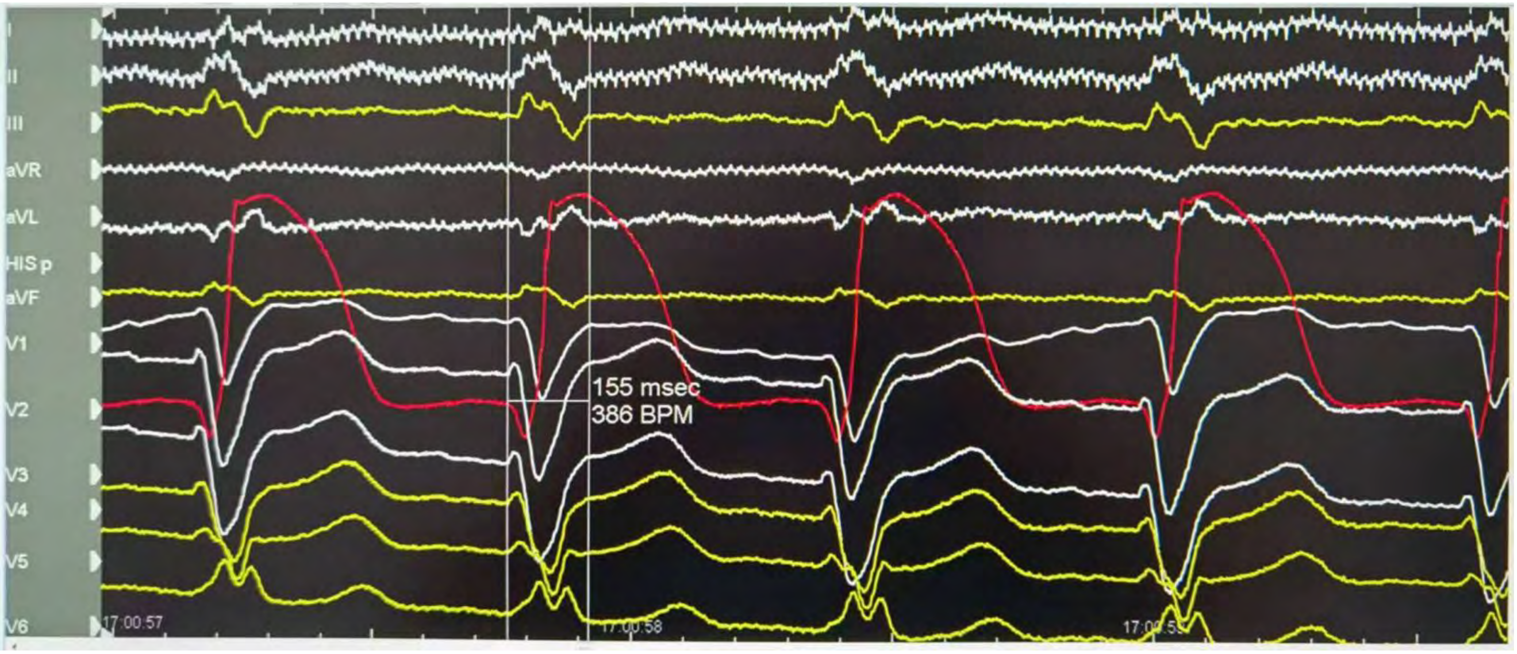

Post ECG

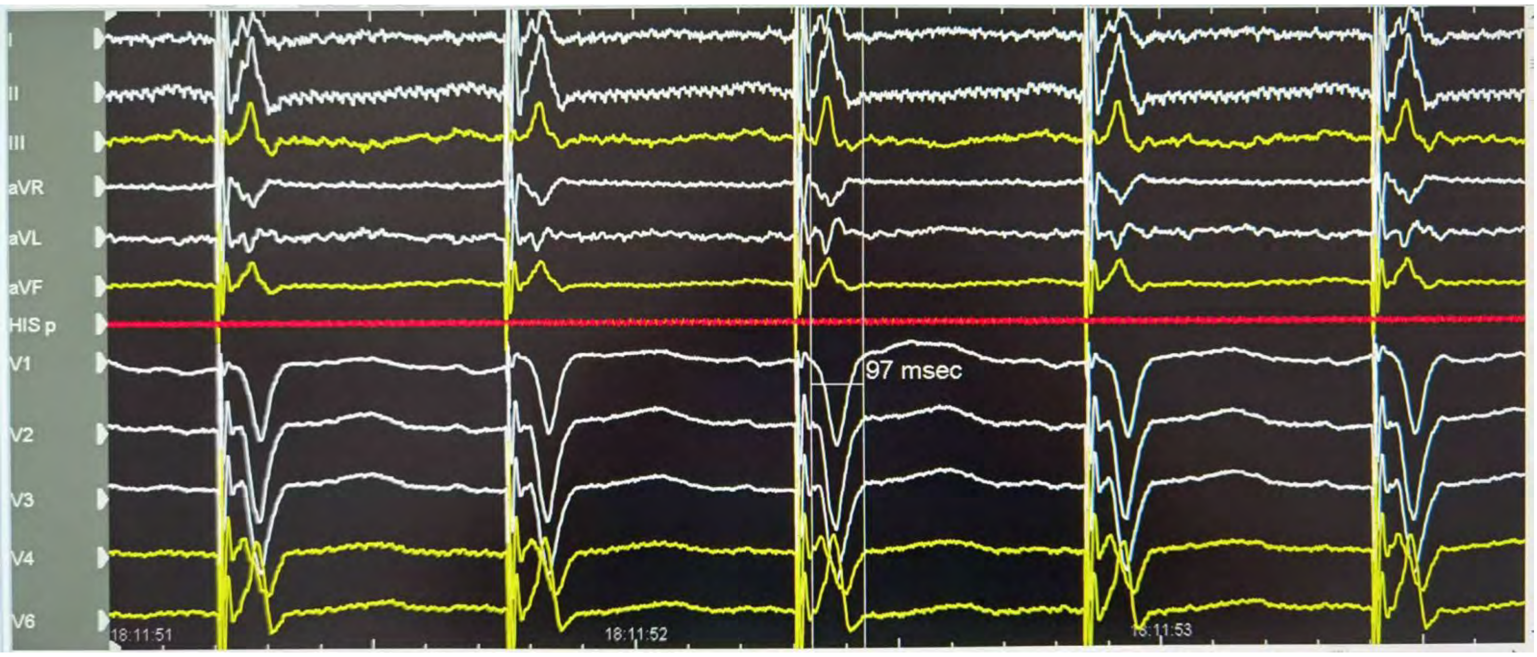

Patient 33:
Transitions

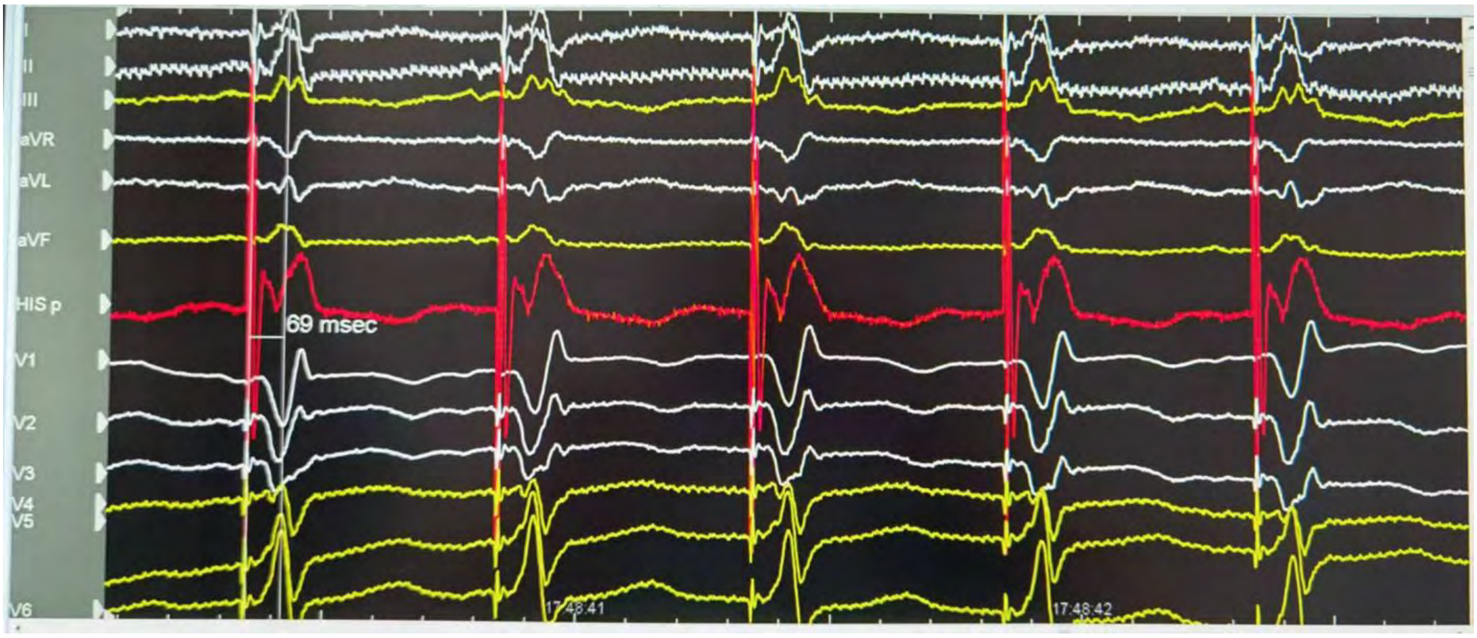

Transitions

Patient 34:

Pre-ECG

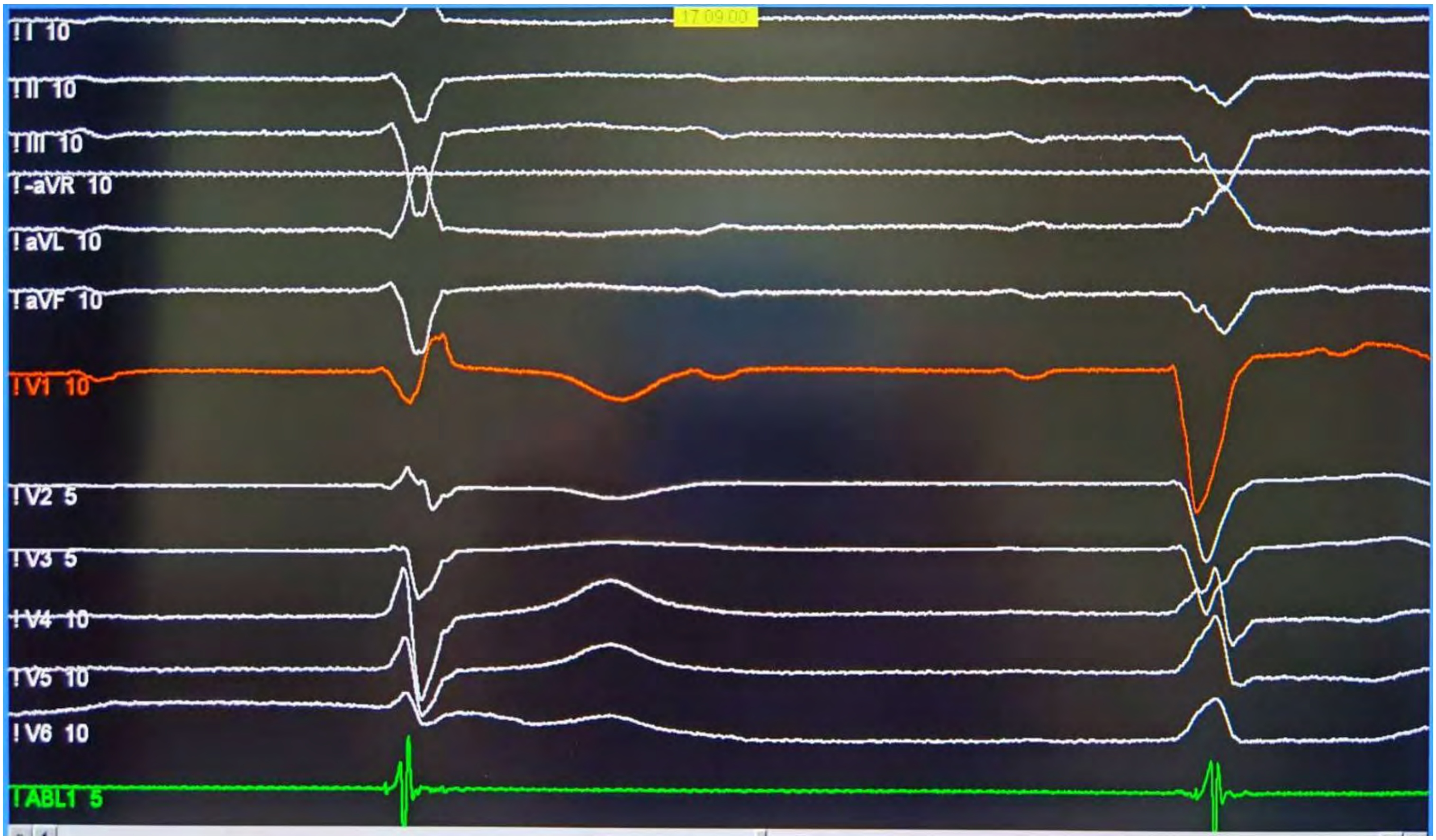

Post ECG

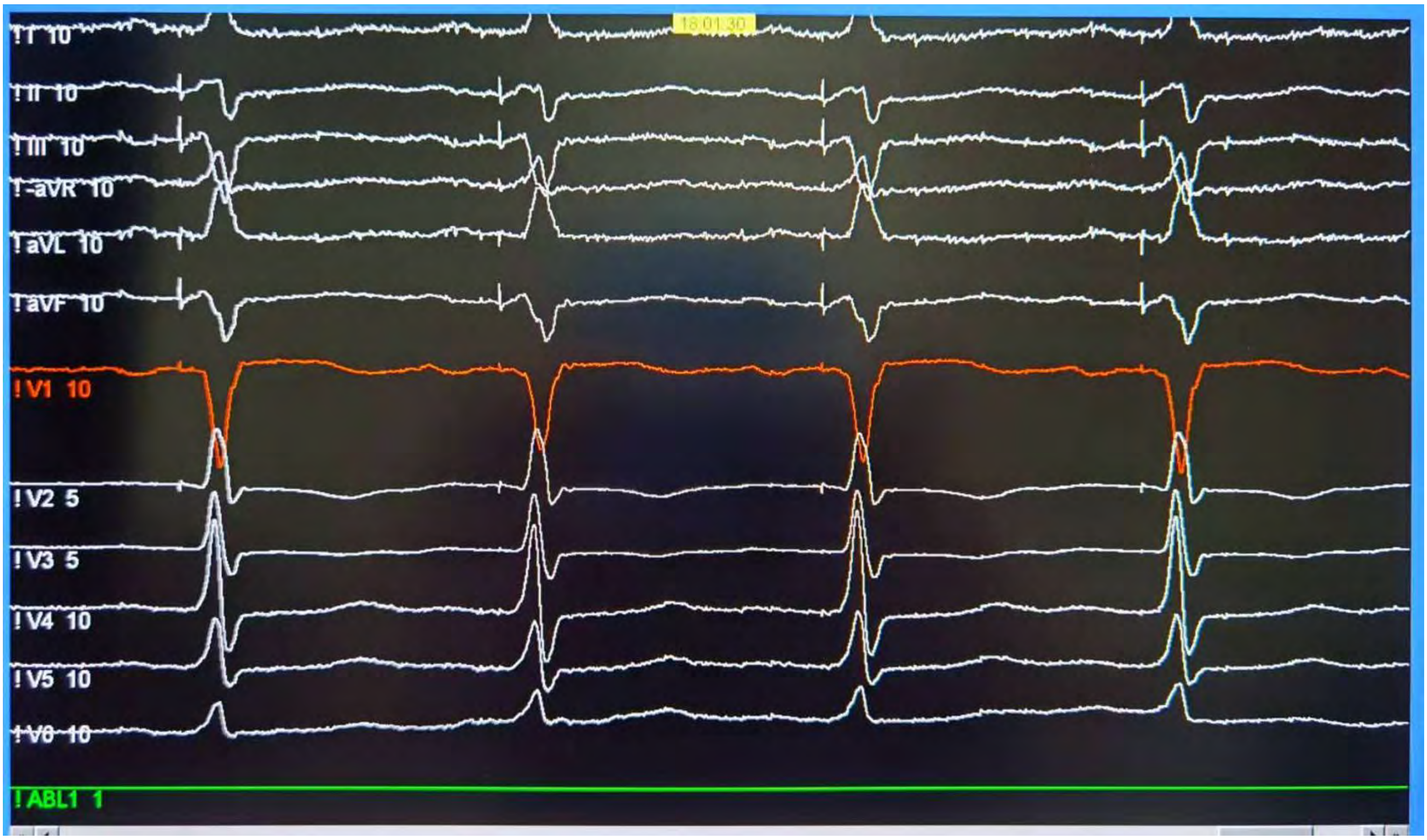

Patient 34:
Transitions

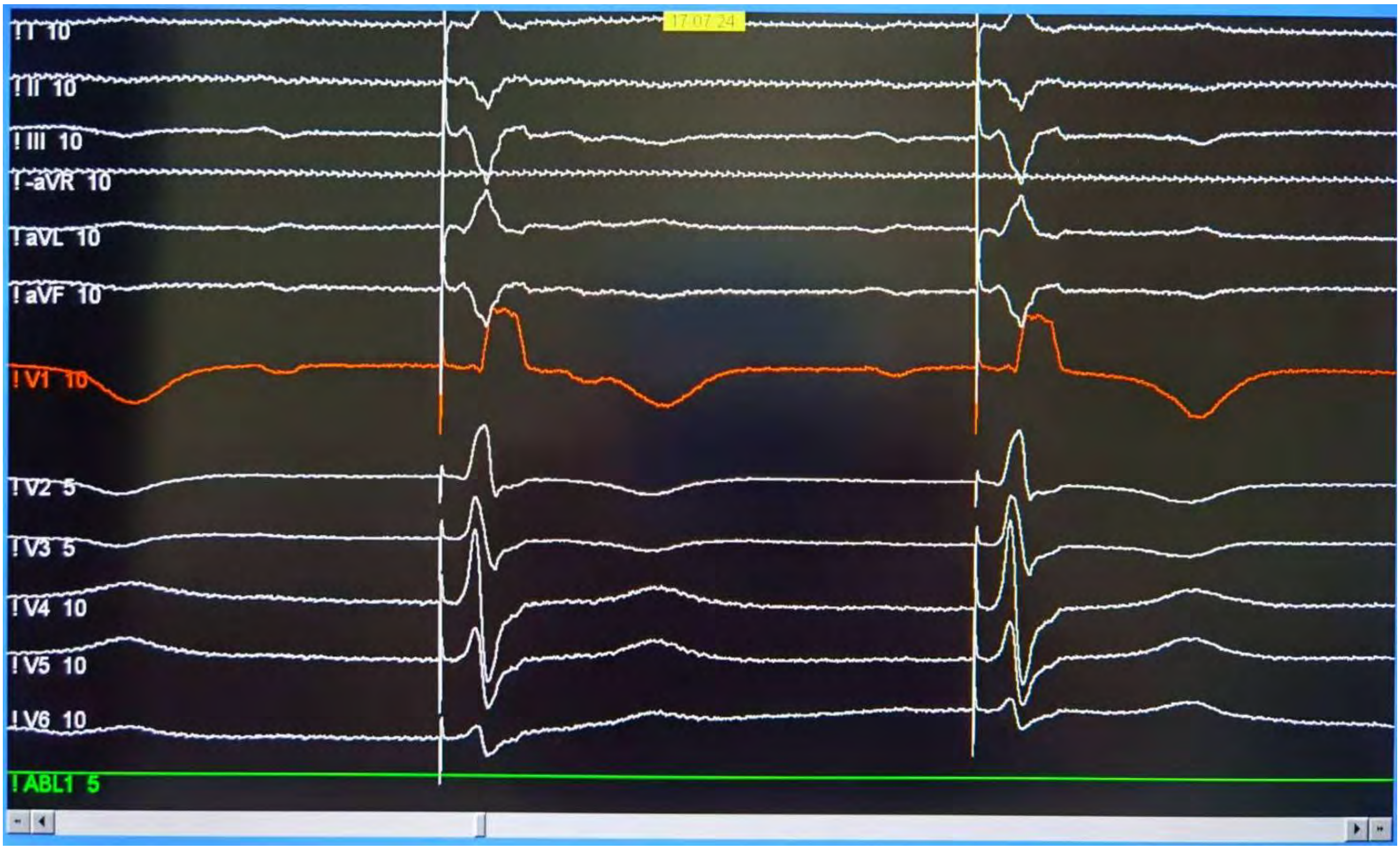

Transitions

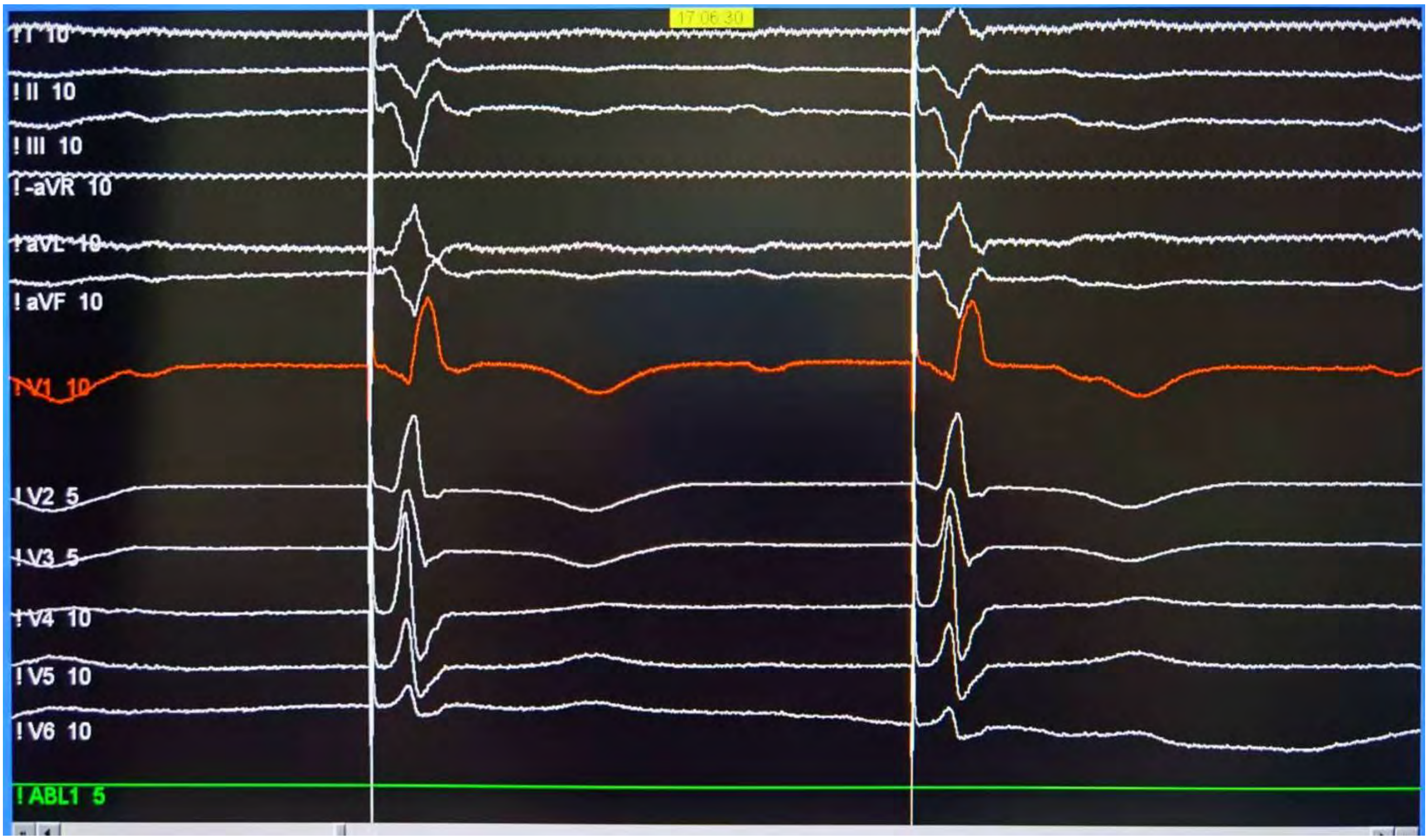

Patient 35:
Pre-ECG

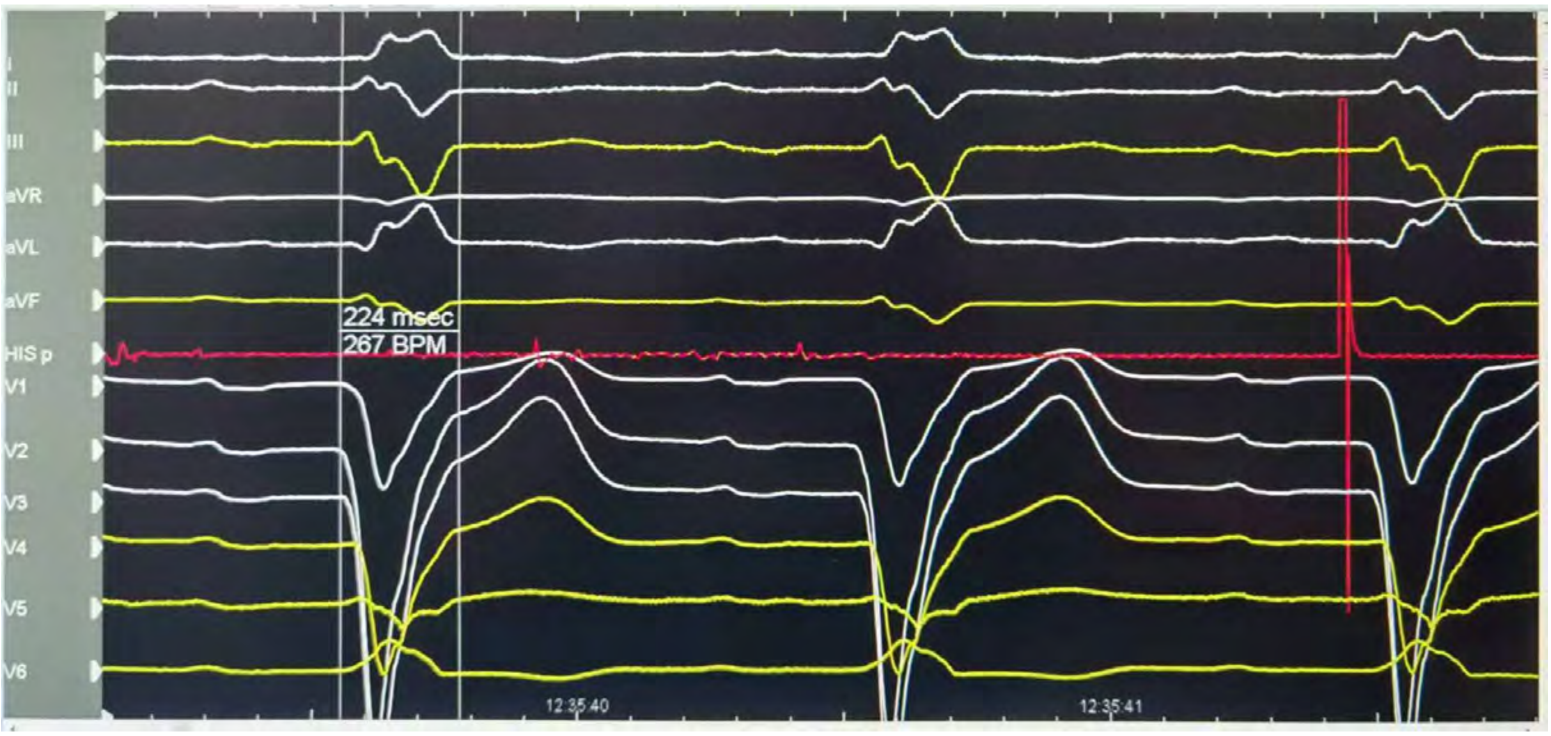

Post ECG

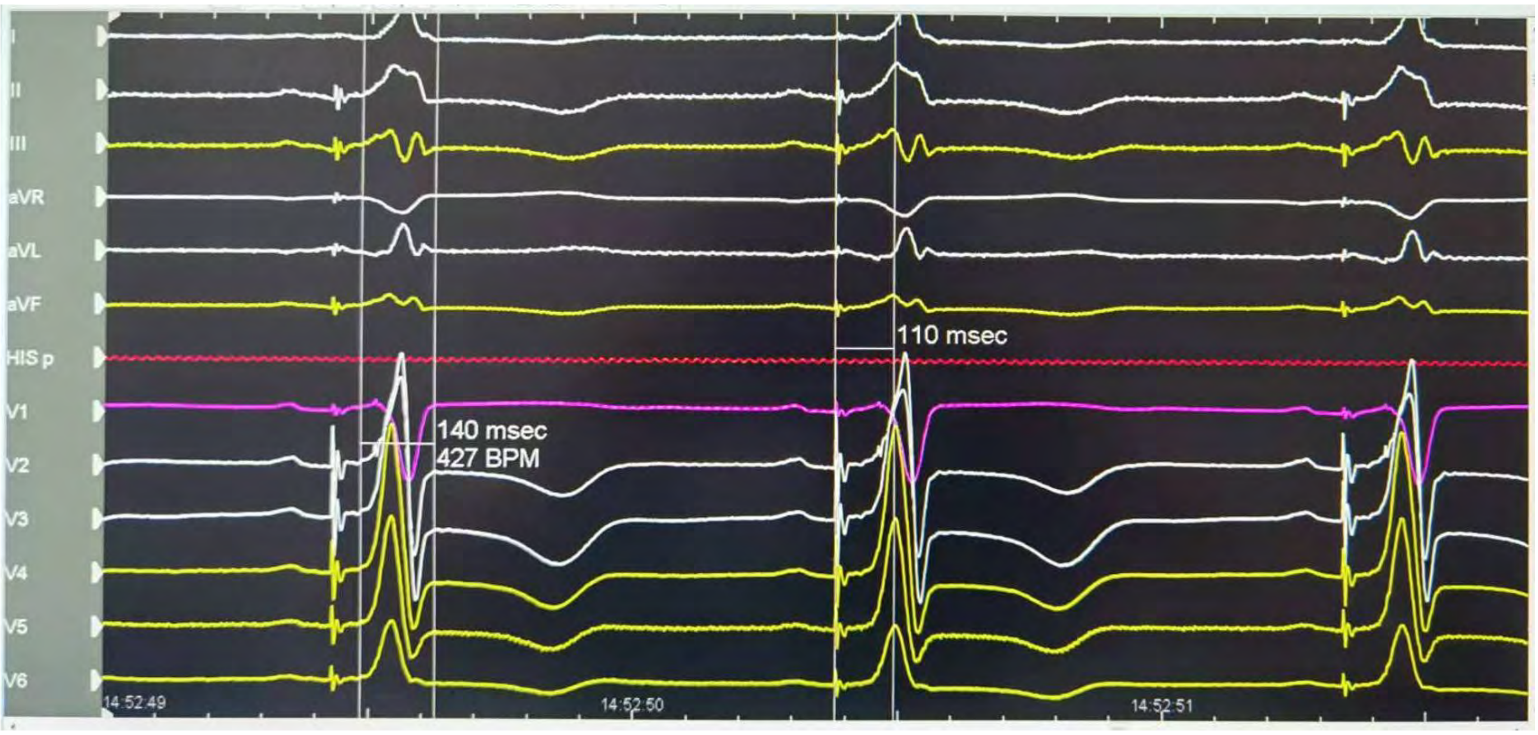

Patient 35:
Transitions

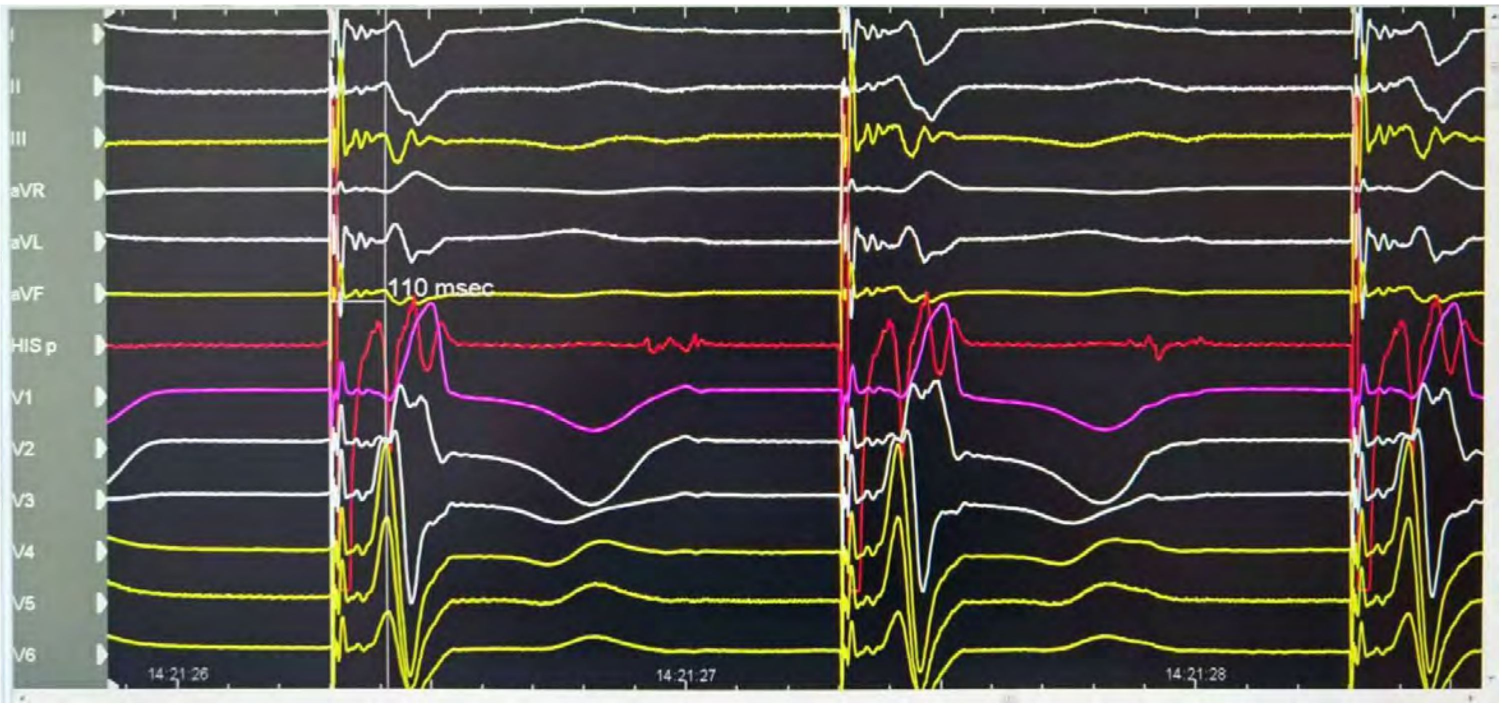

Transitions

Patient 36:
Pre-ECG

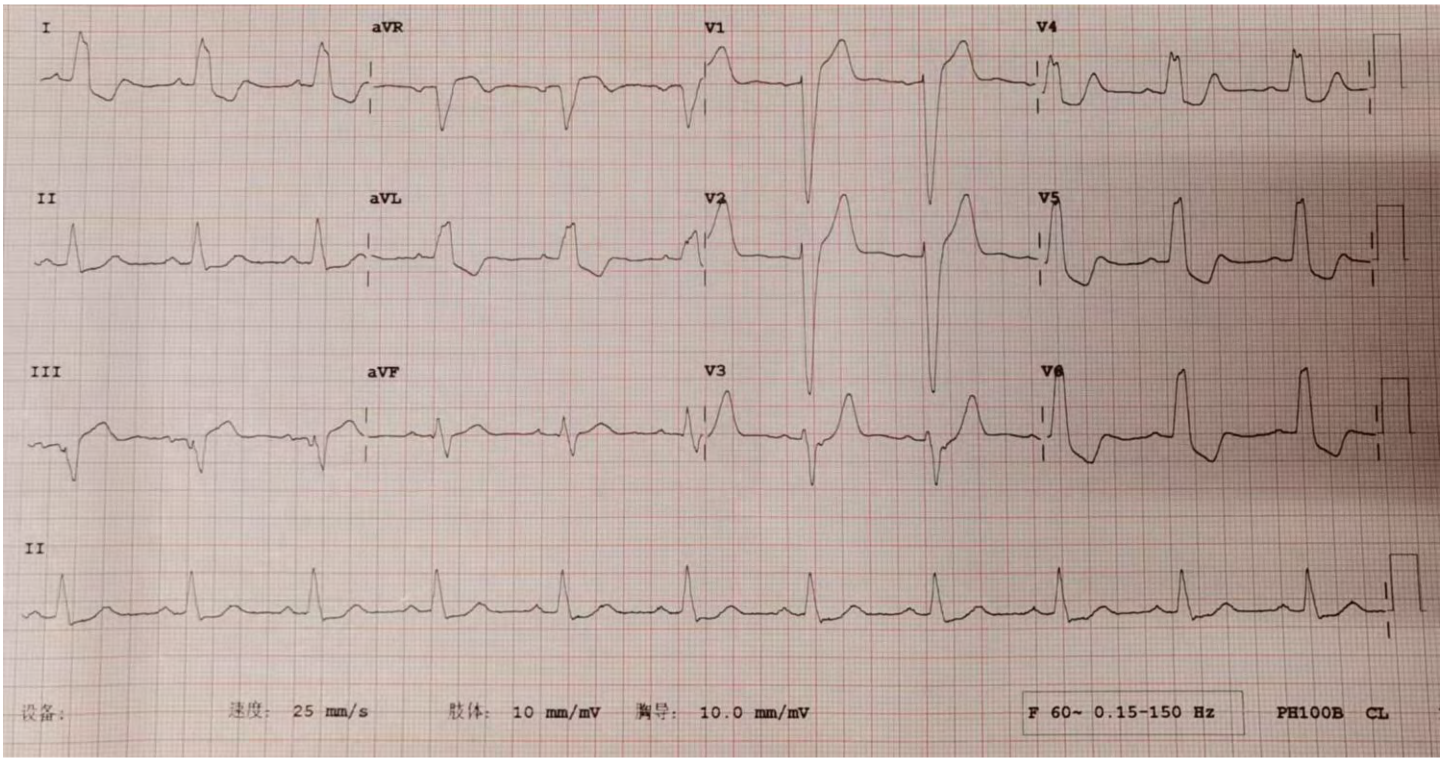

Post ECG

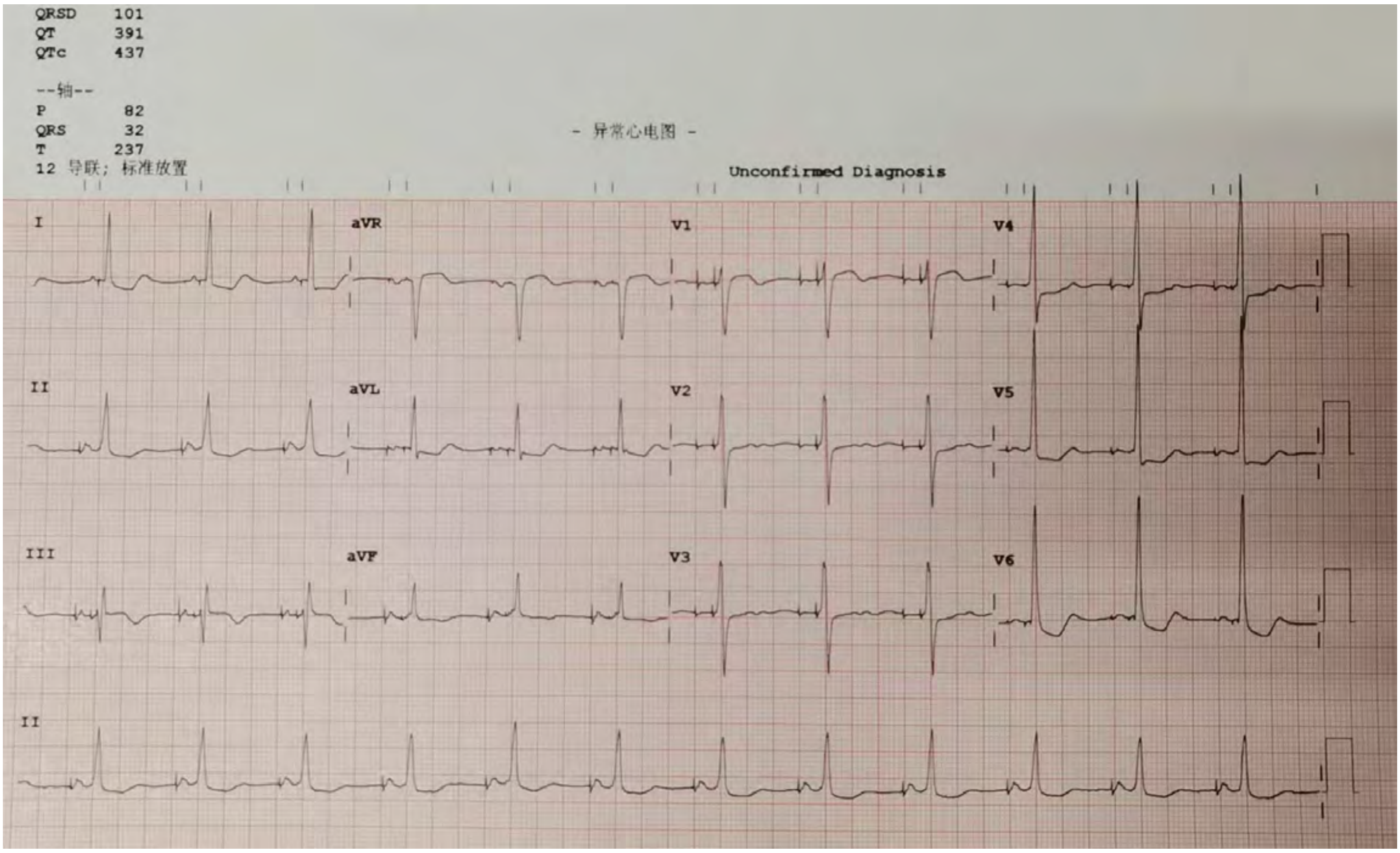

Patient 37:
Pre-ECG

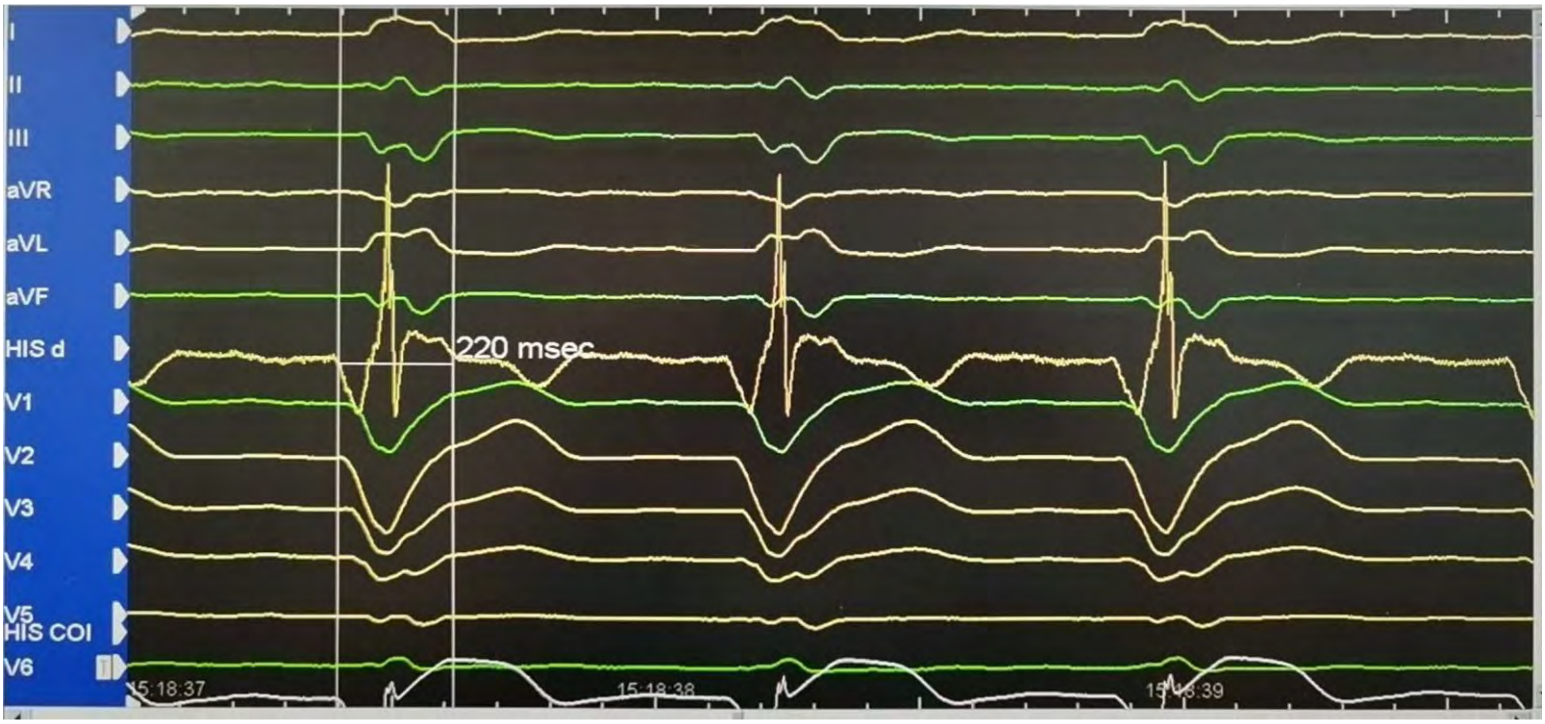

Post ECG

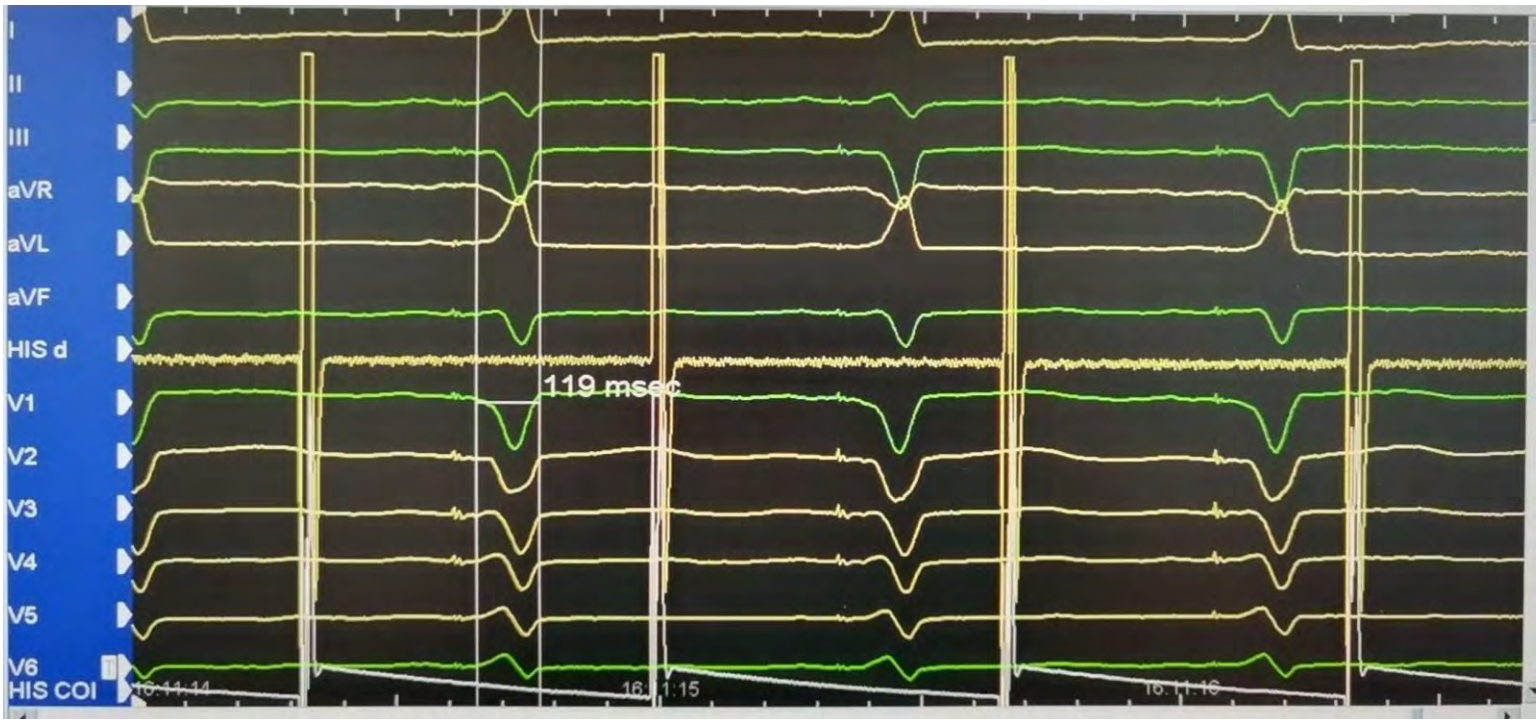

Patient 37:
Transitions

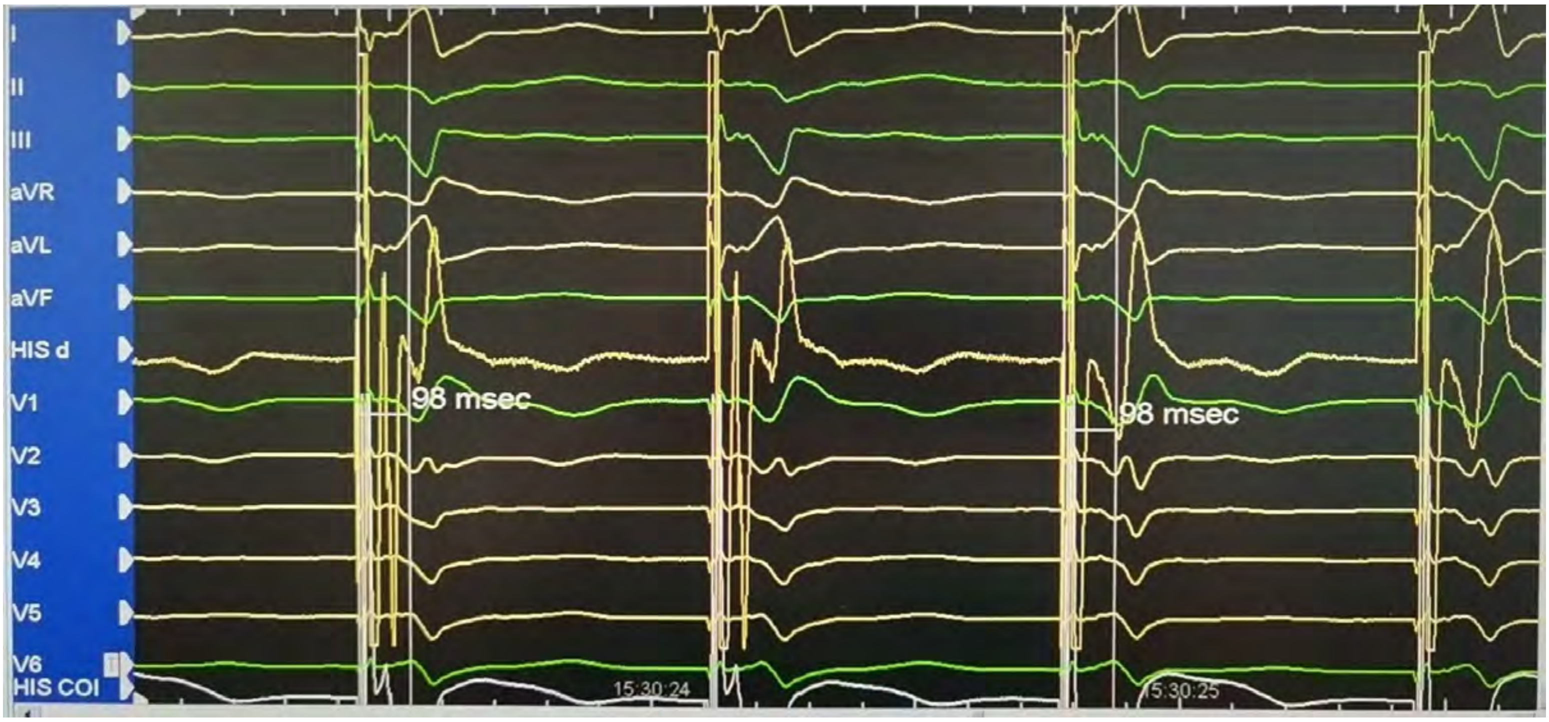

Transitions

Patient 38:
Pre-ECG

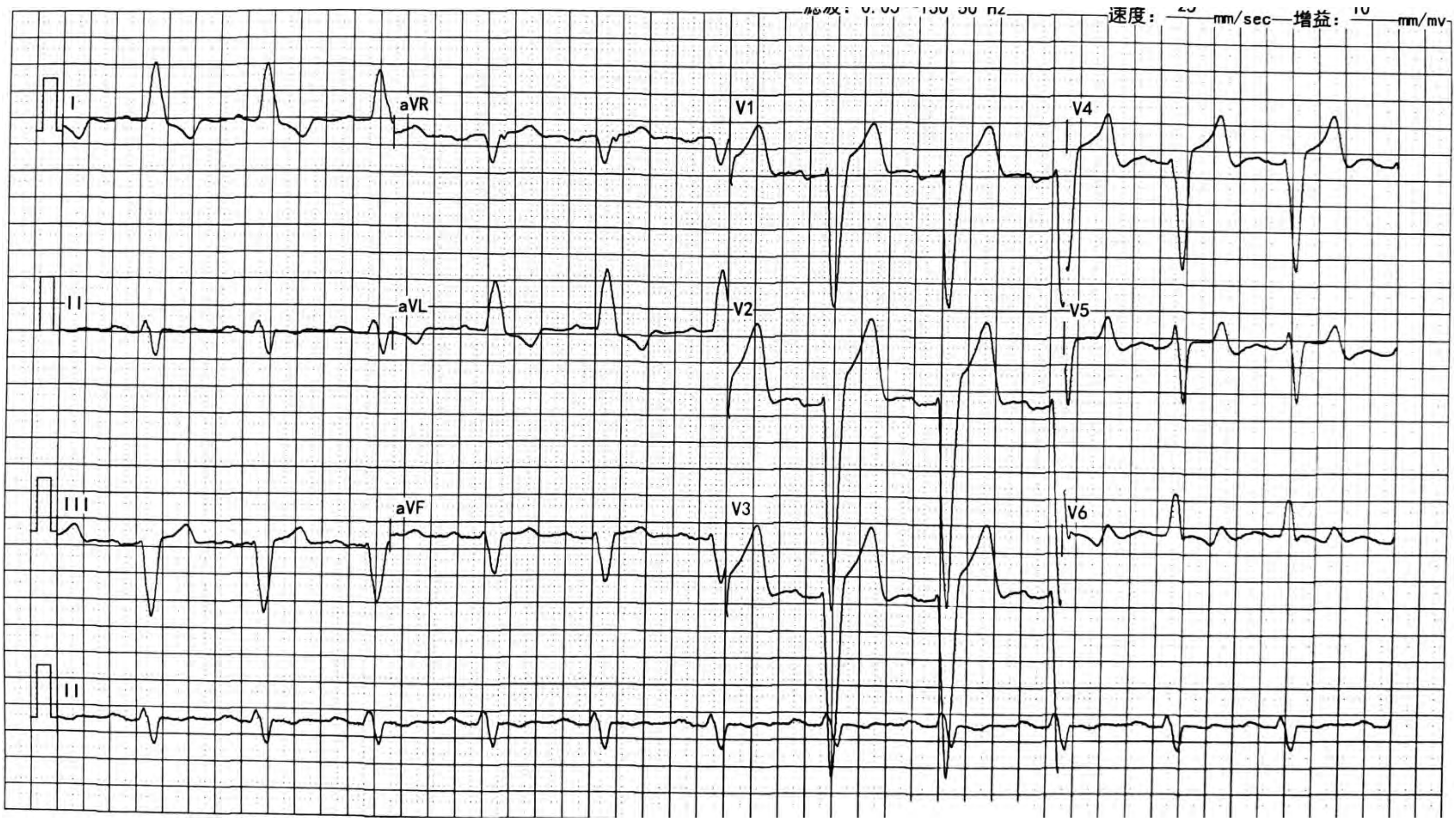

Post ECG

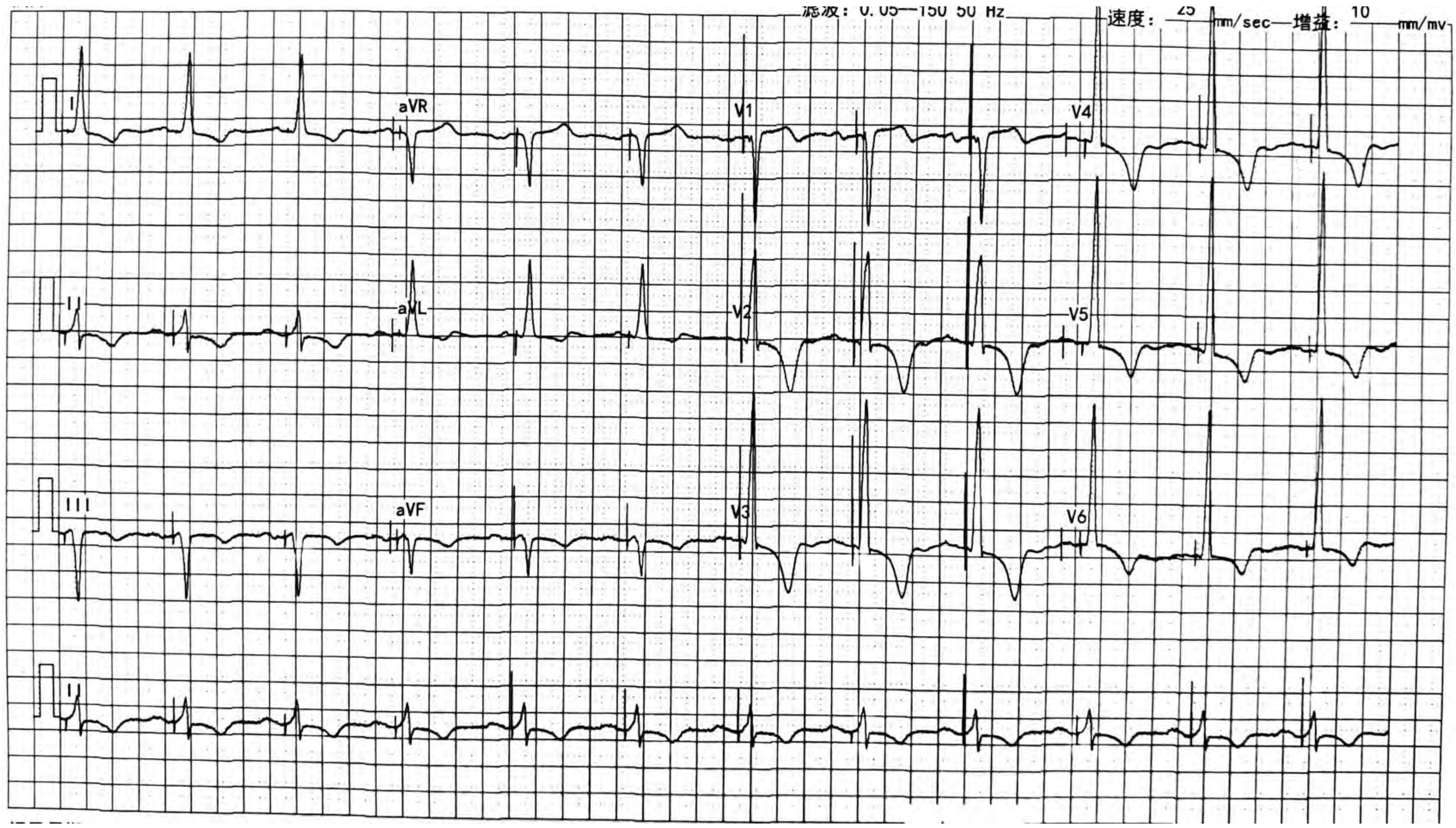

Patient 39:
Pre-ECG

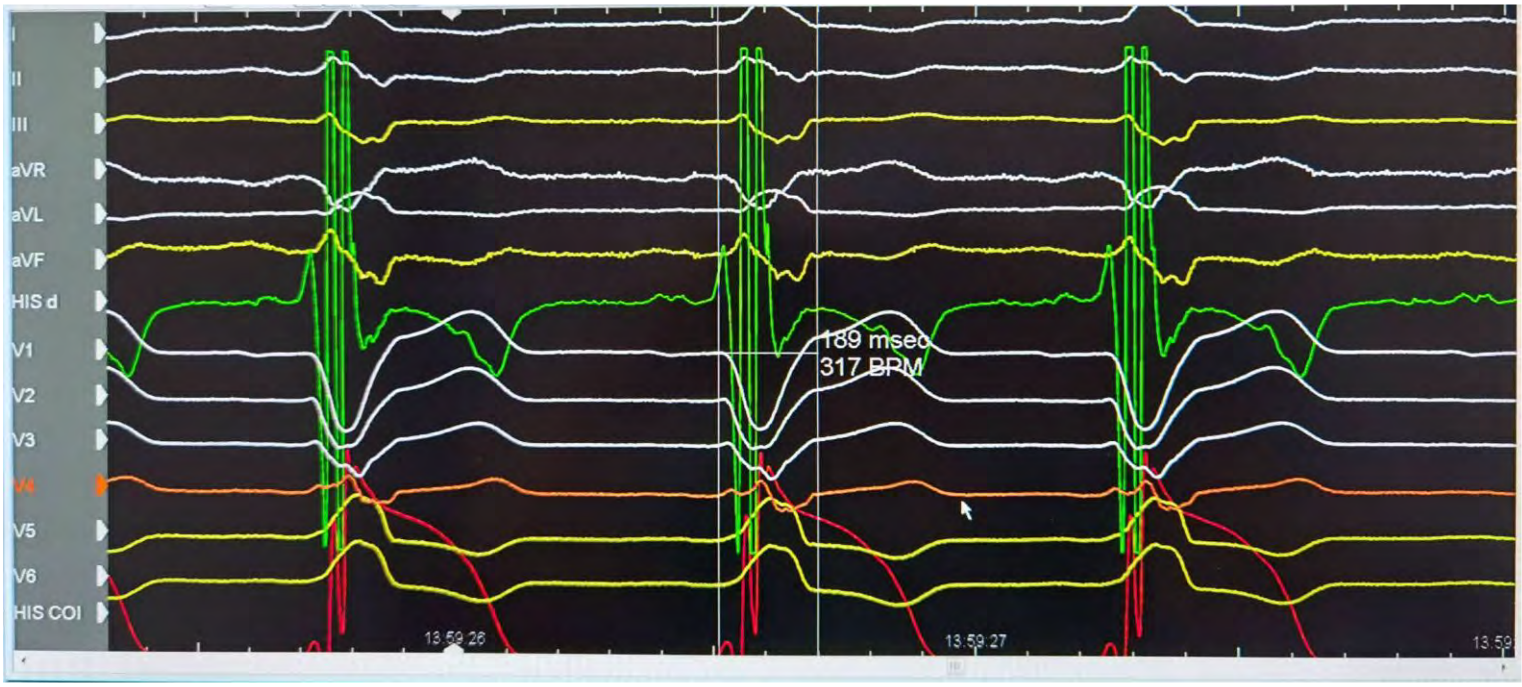

Post ECG

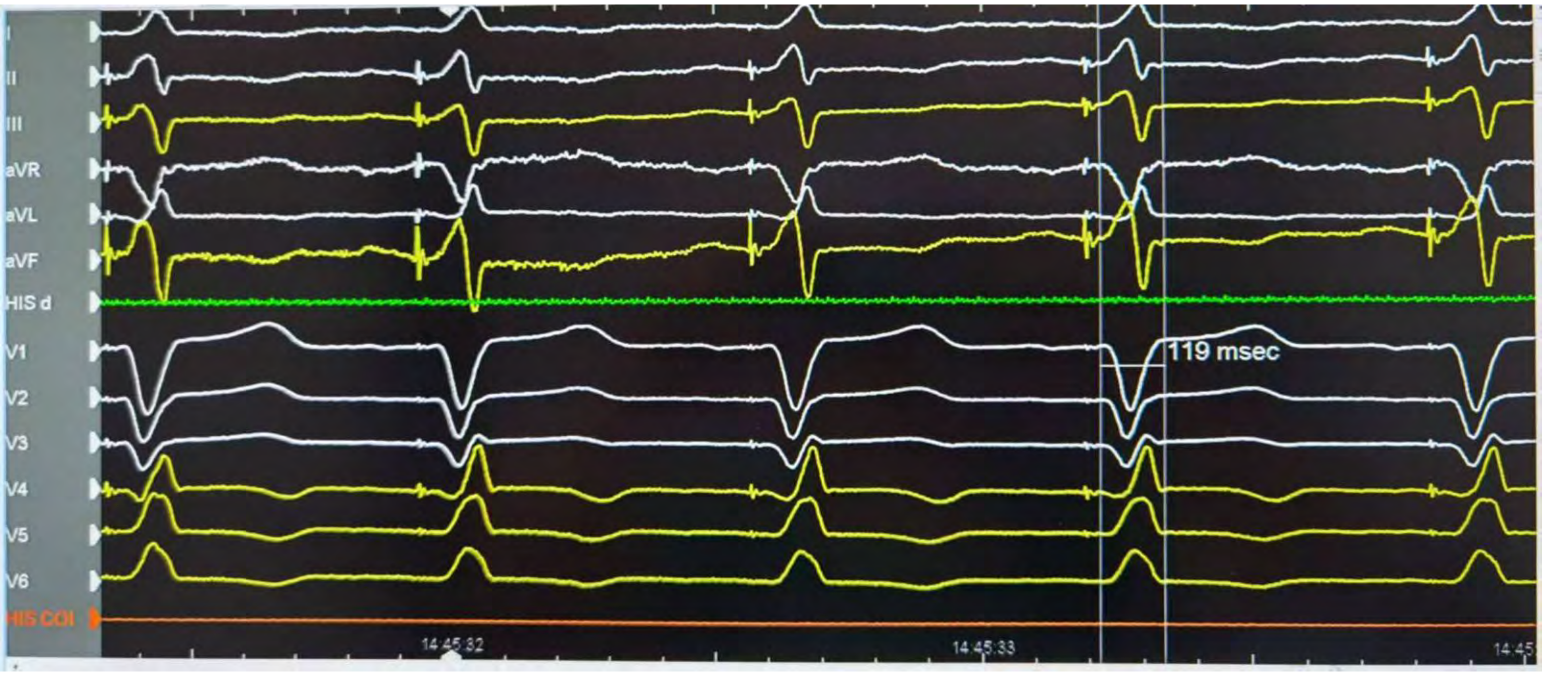

Patient 40:

Pre-ECG

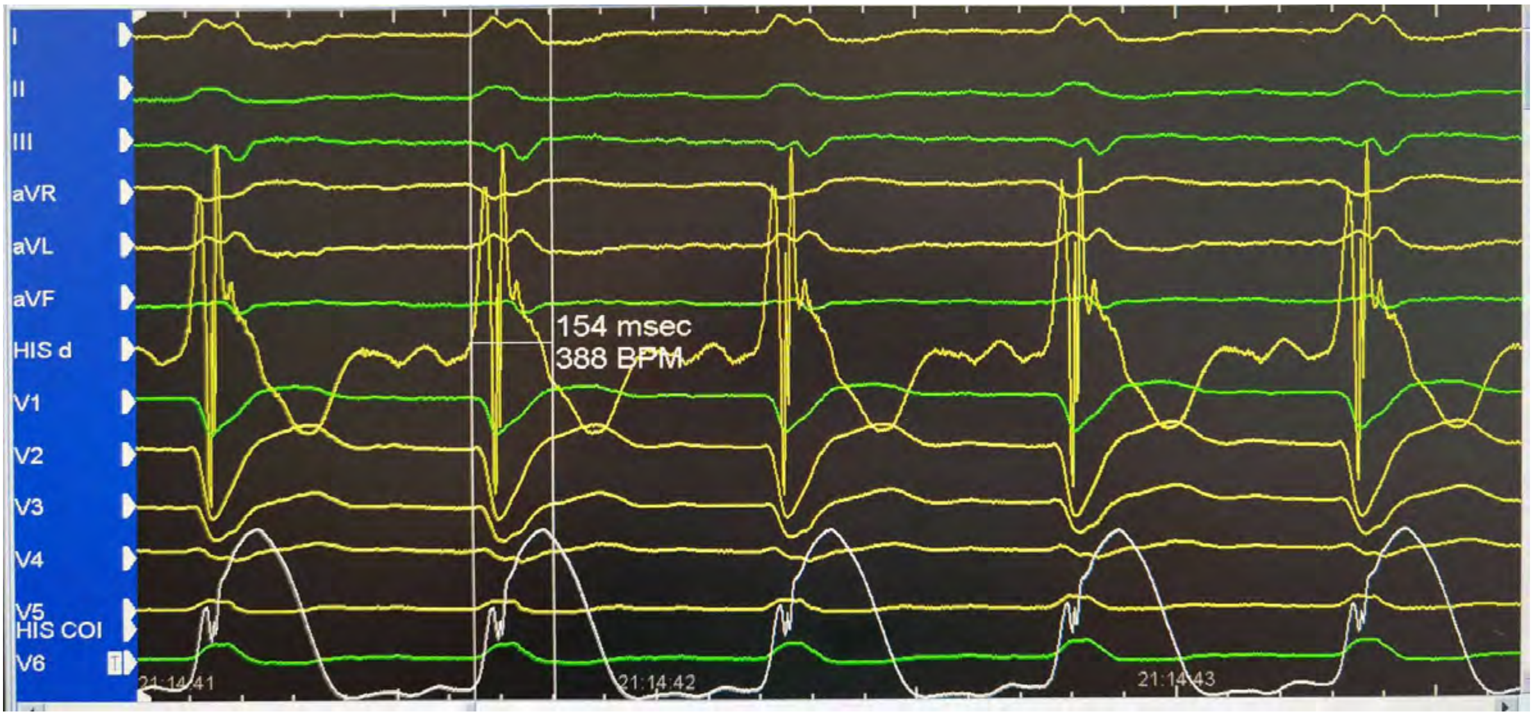

Post ECG

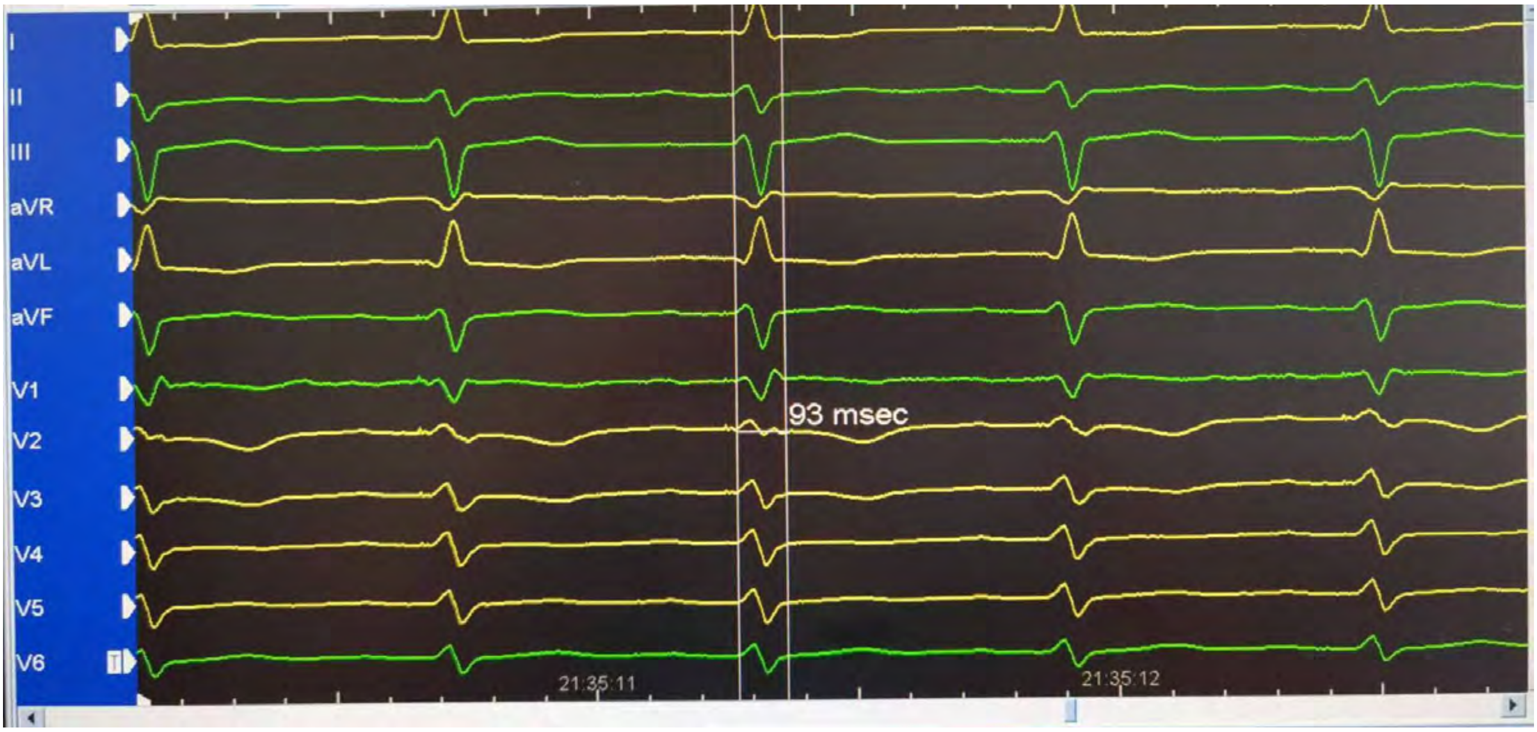

Patient 41:
Pre-ECG

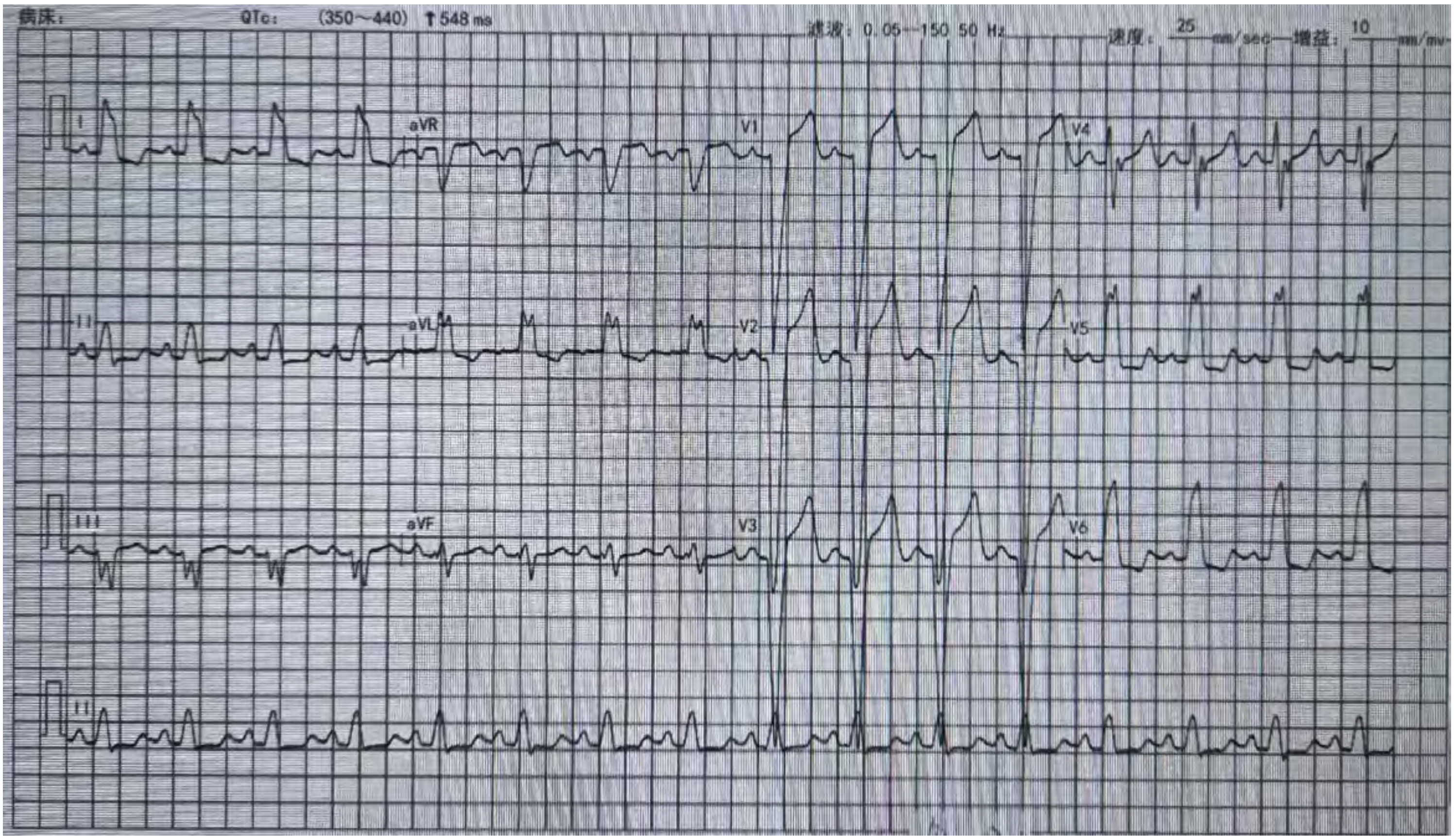

Post ECG

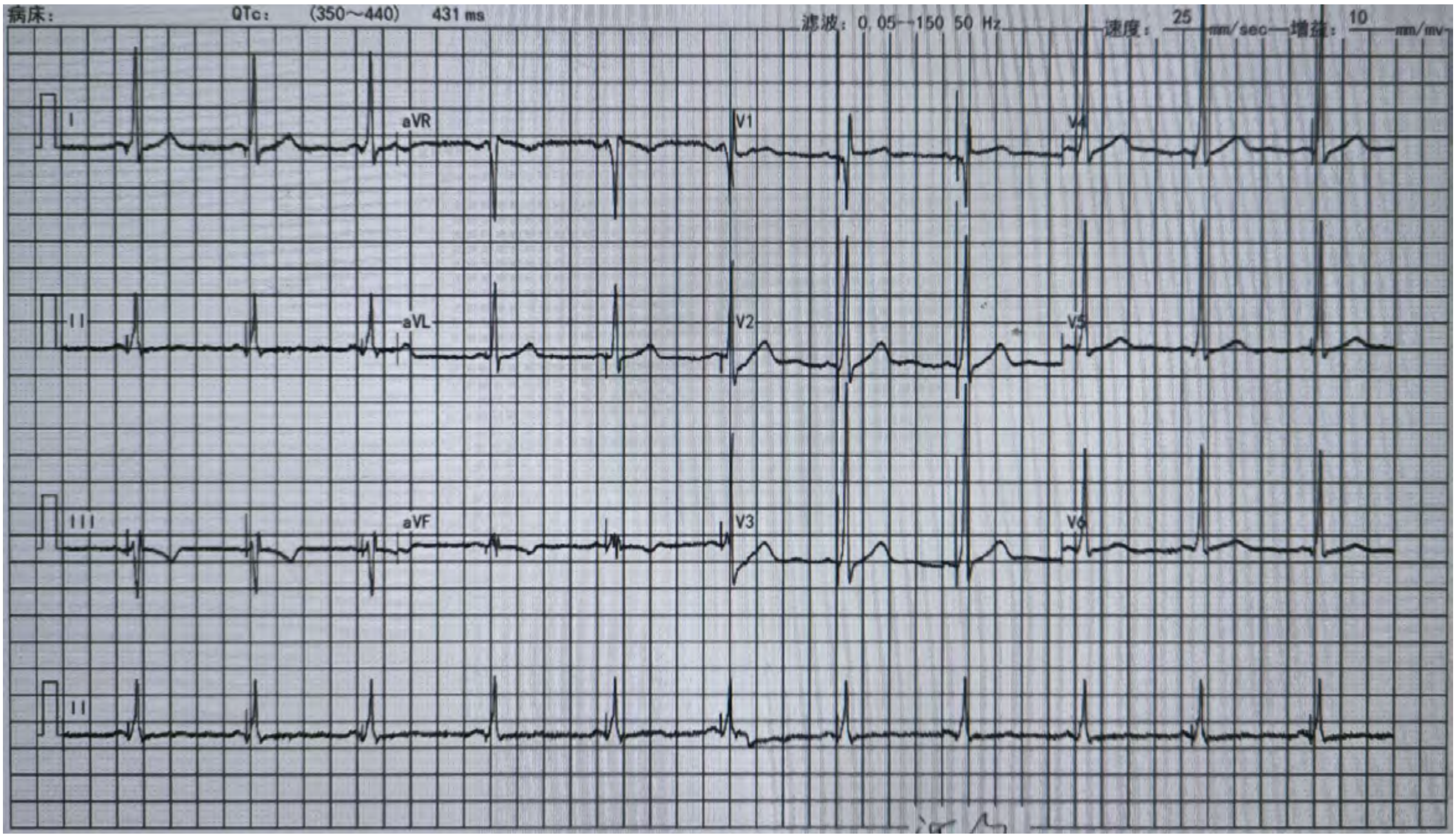

Patient 41:
Transitions

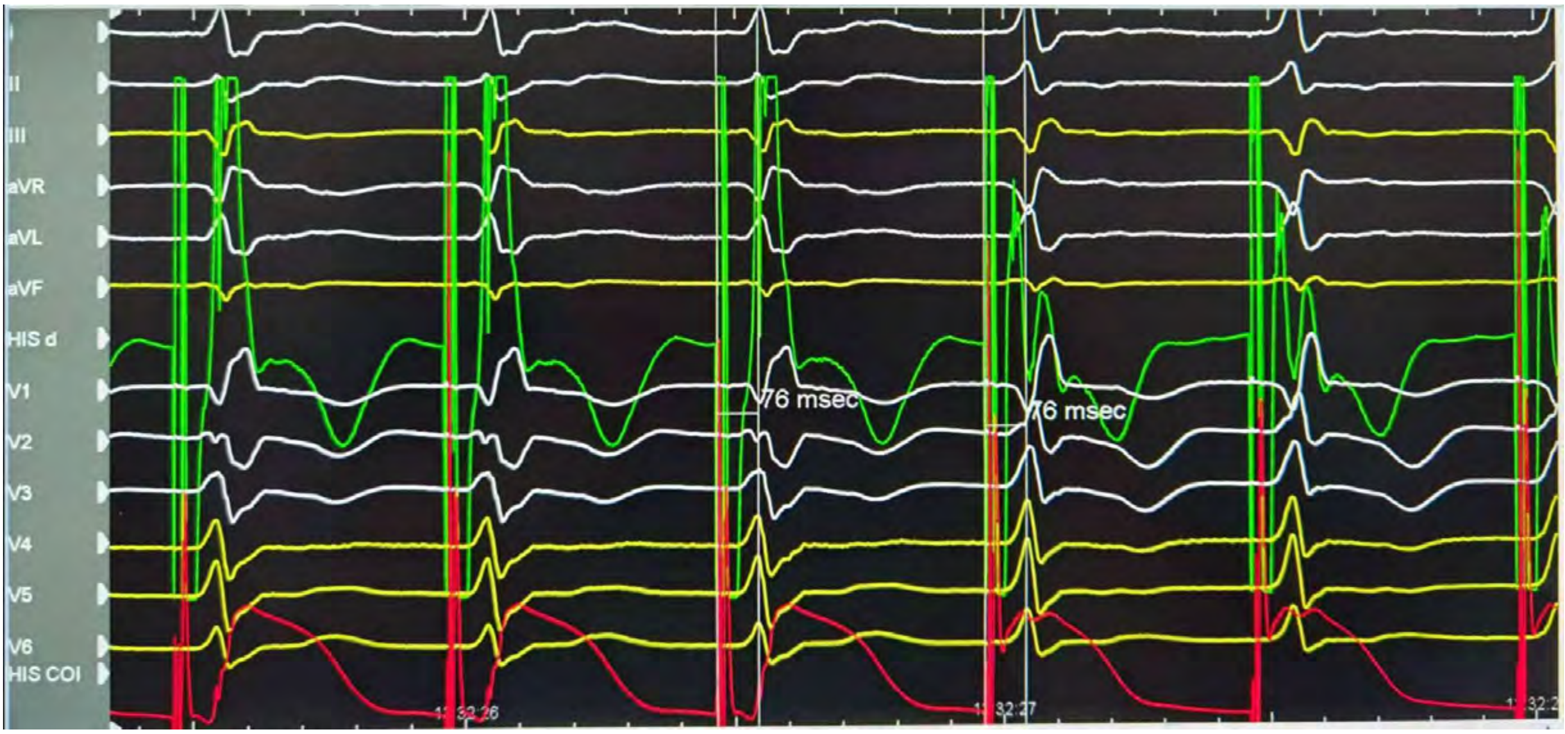

Transitions

Patient 42:
Pre-ECG

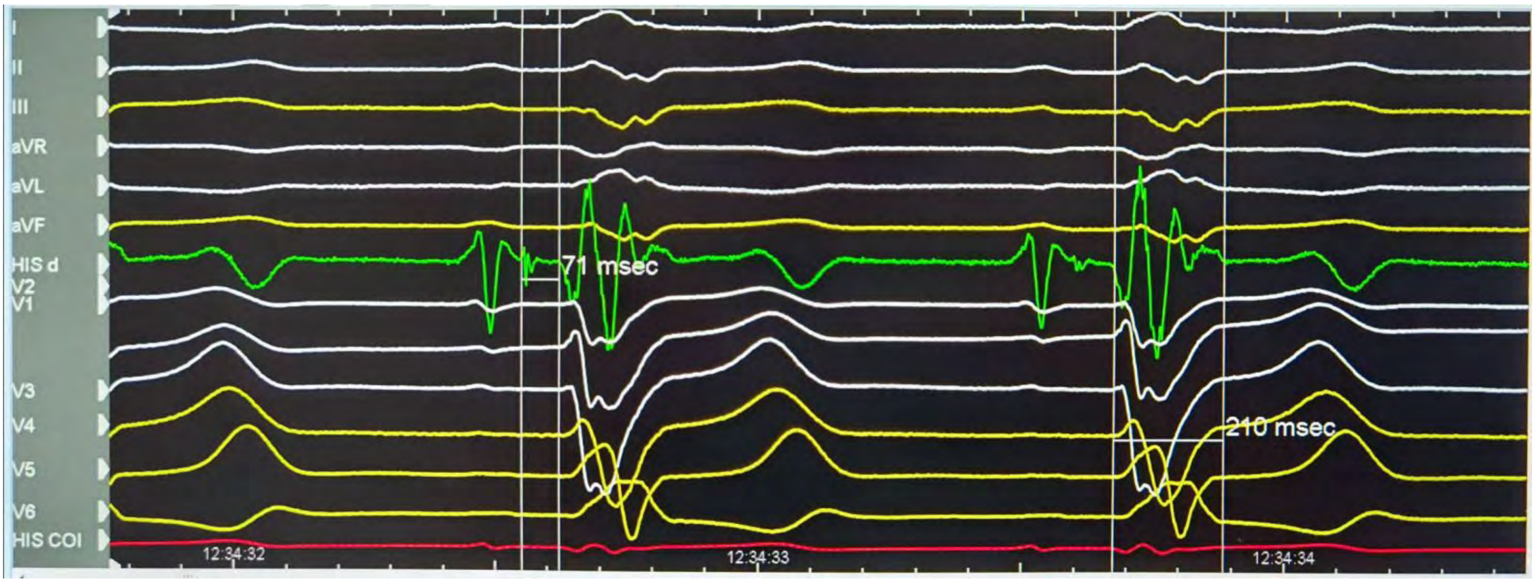

Post ECG

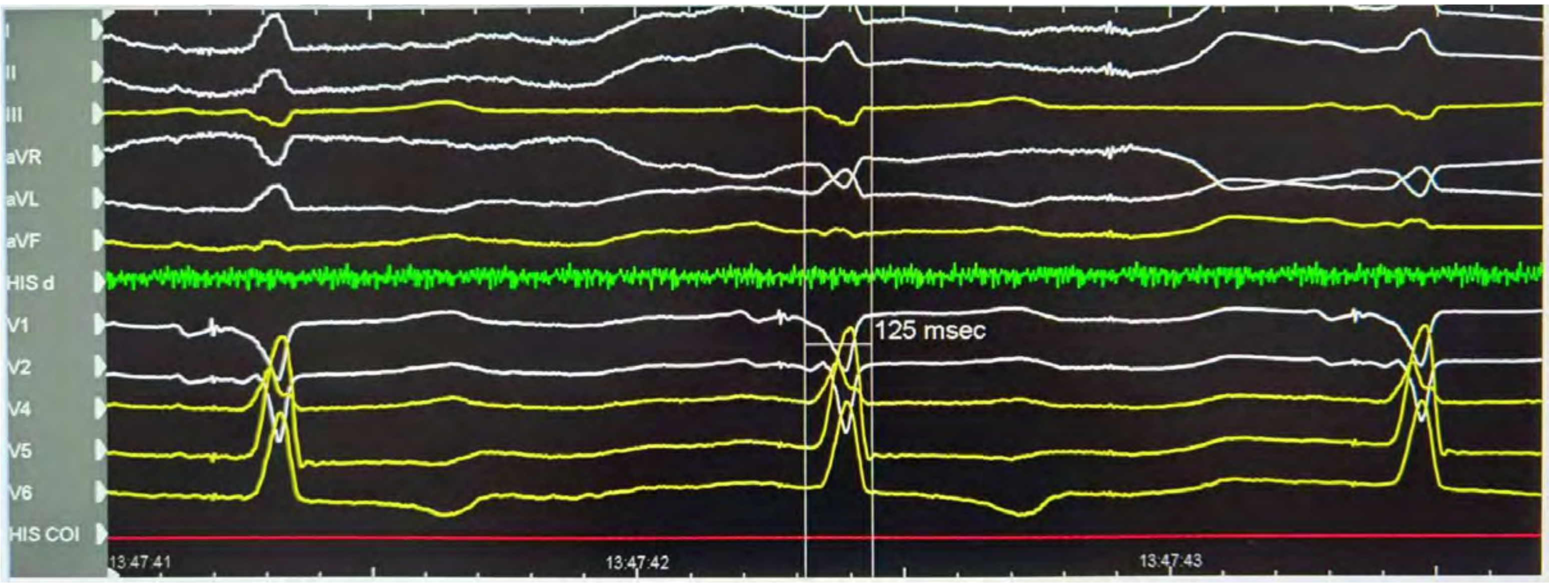

Patient 42:
Transitions

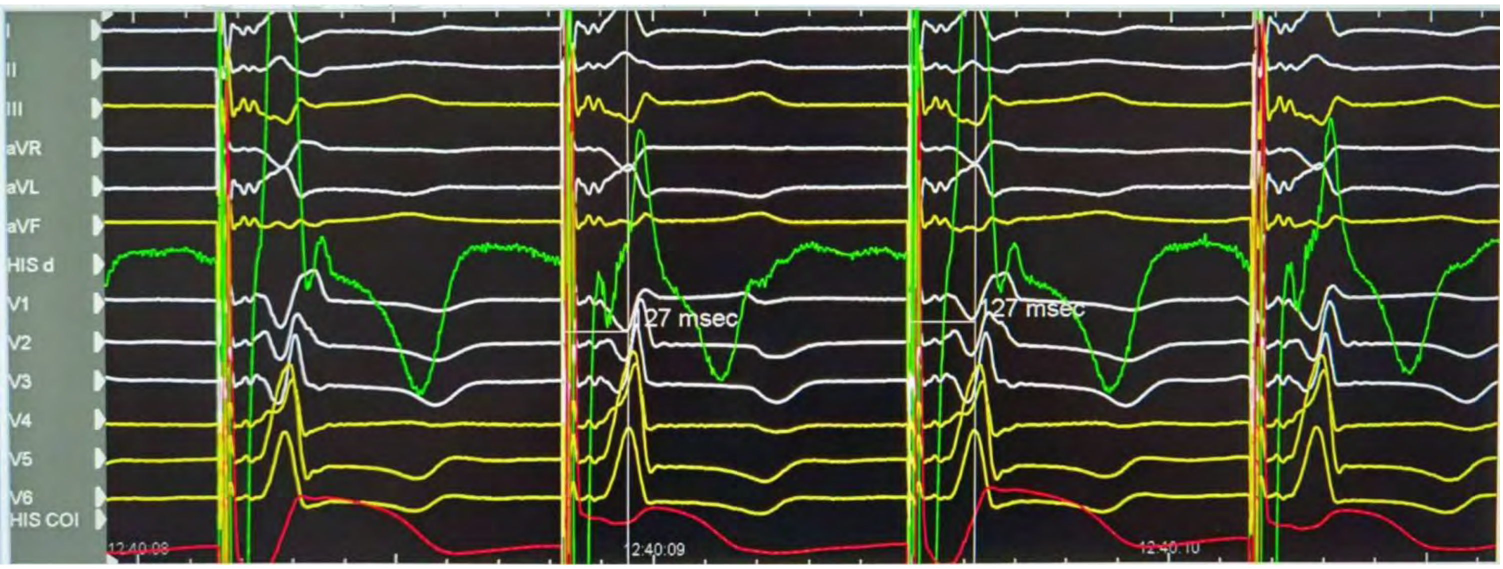

Transitions

Patient 43:
Pre-ECG

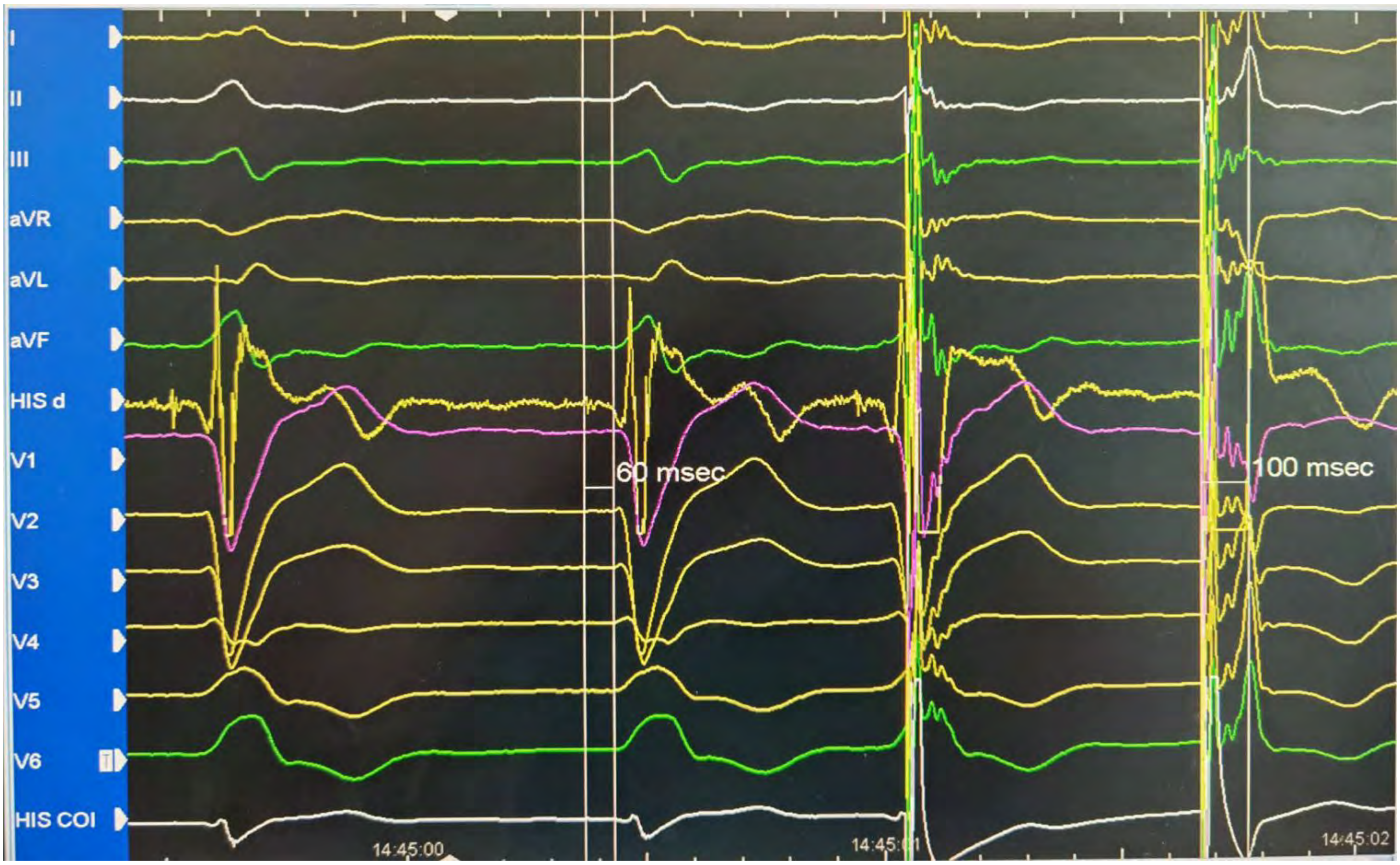

Post ECG

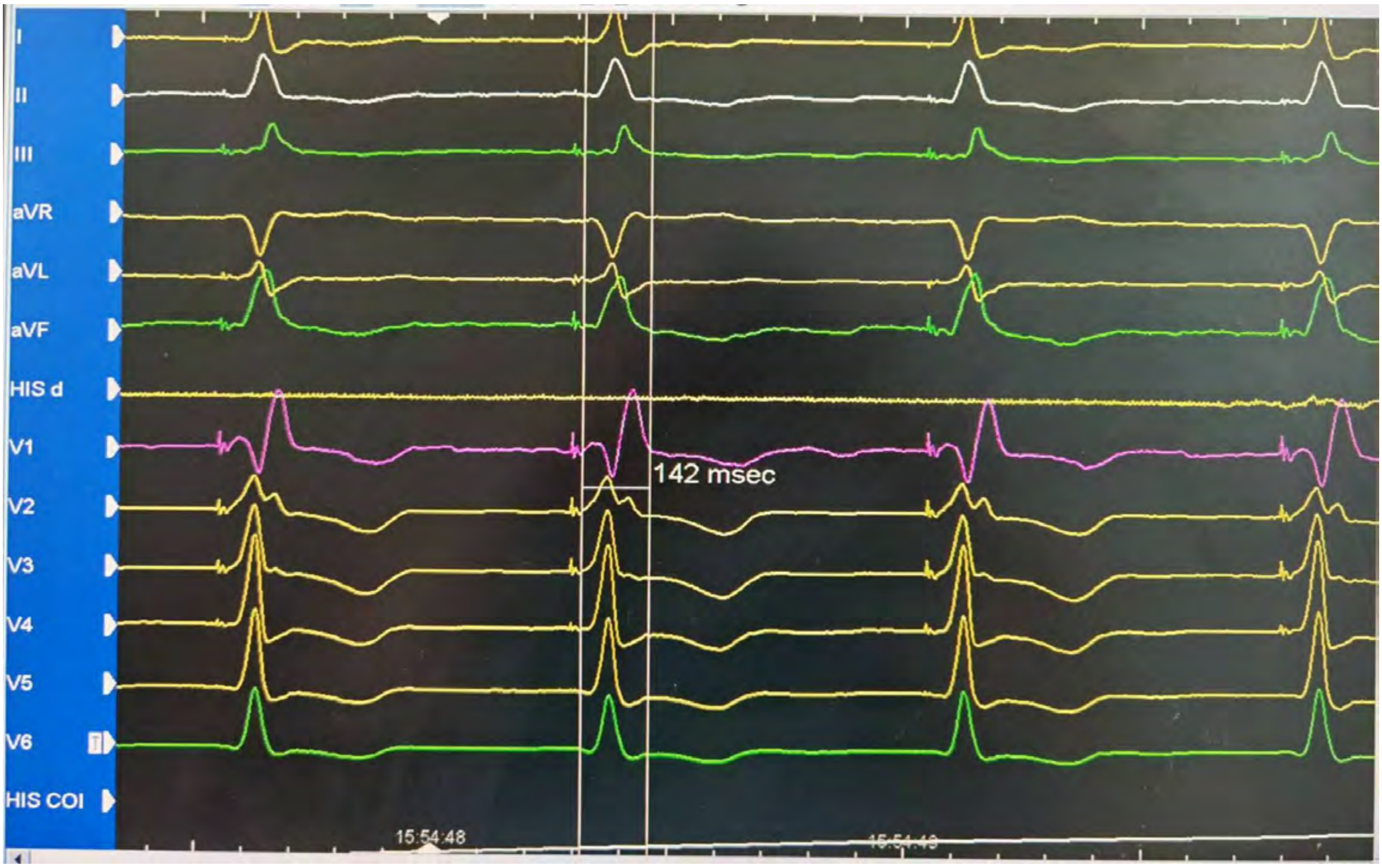

Patient 43:
Transitions

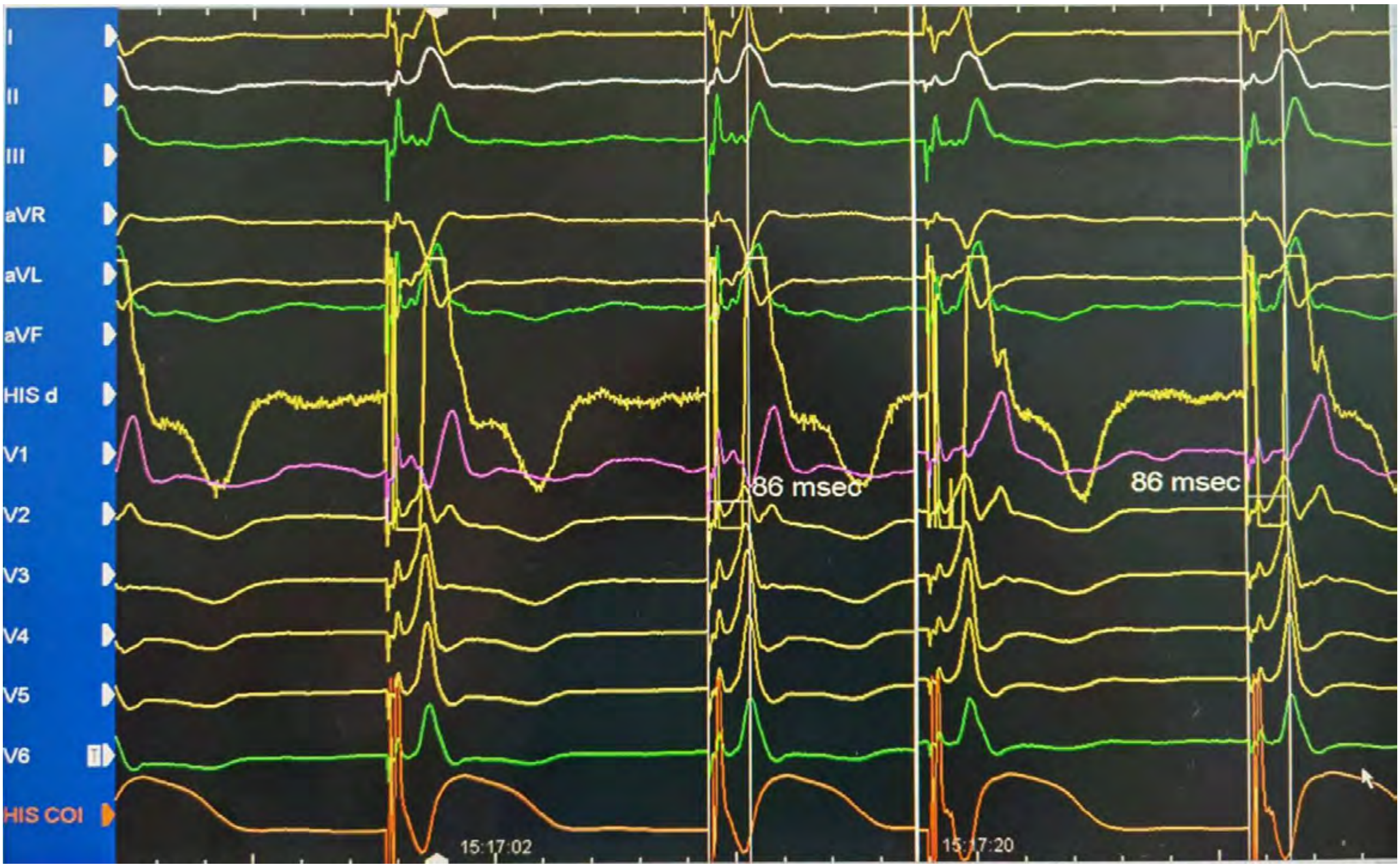

Transitions

Patient 44:
Pre-ECG

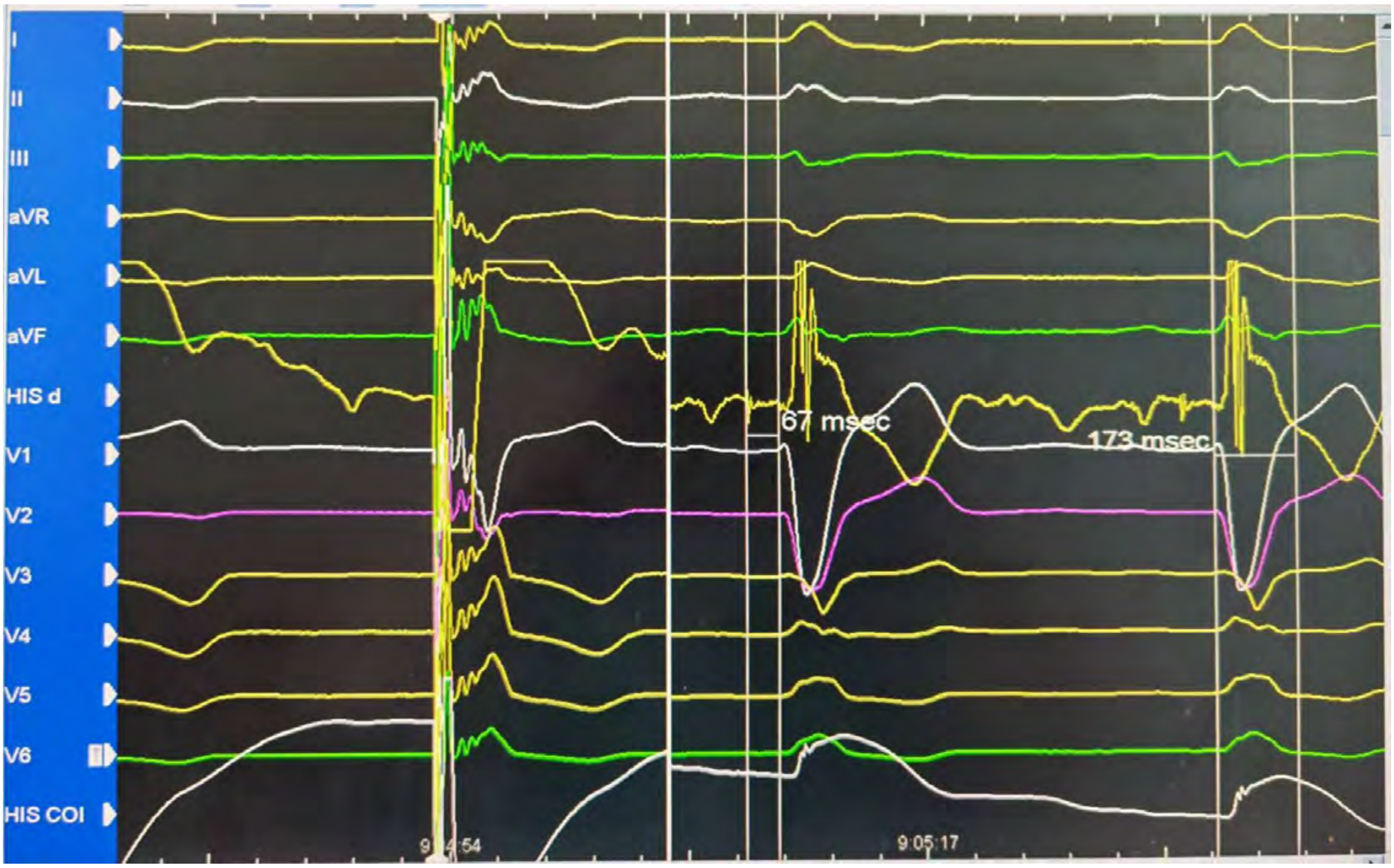

Post ECG

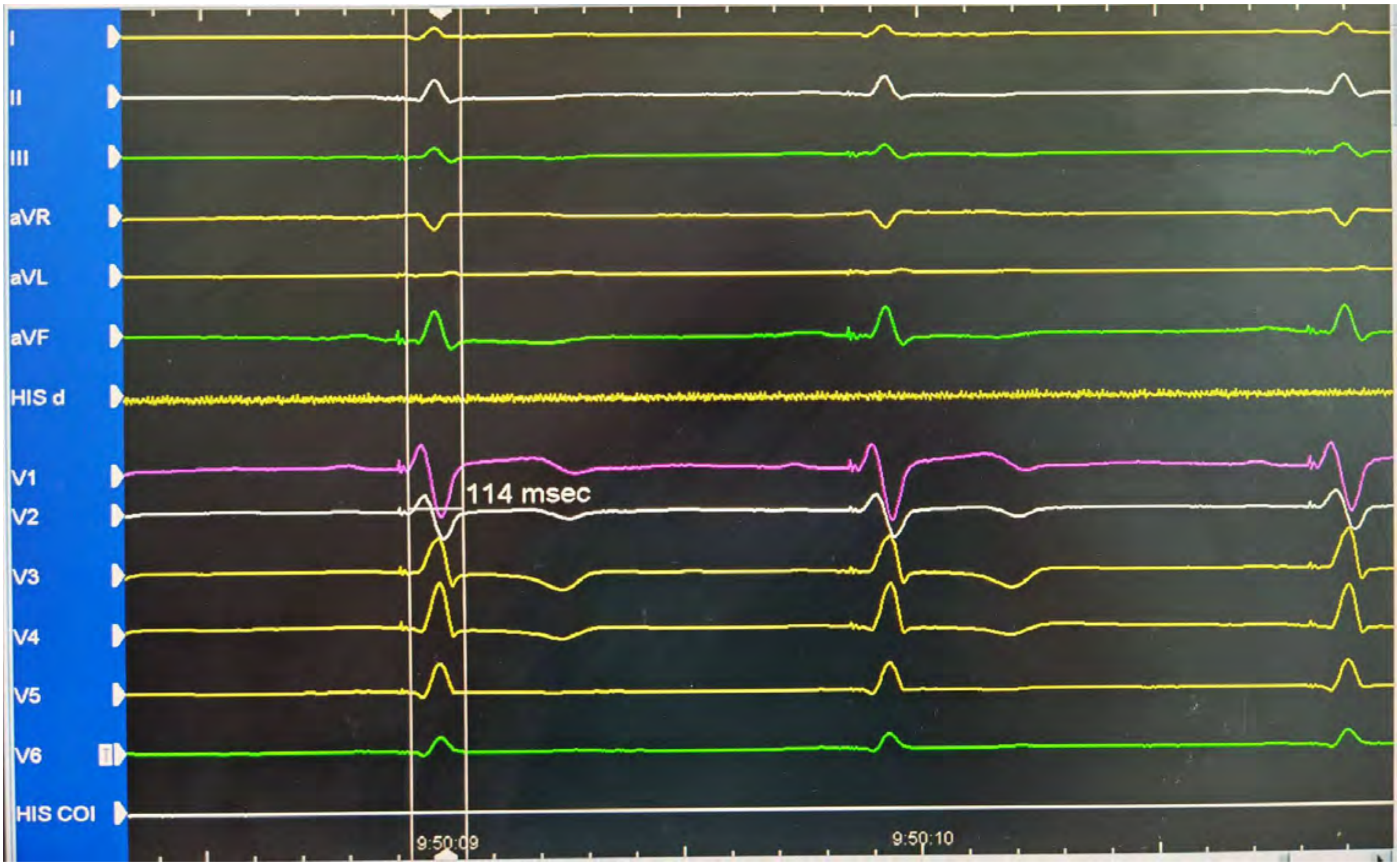

Patient 44:
Transitions

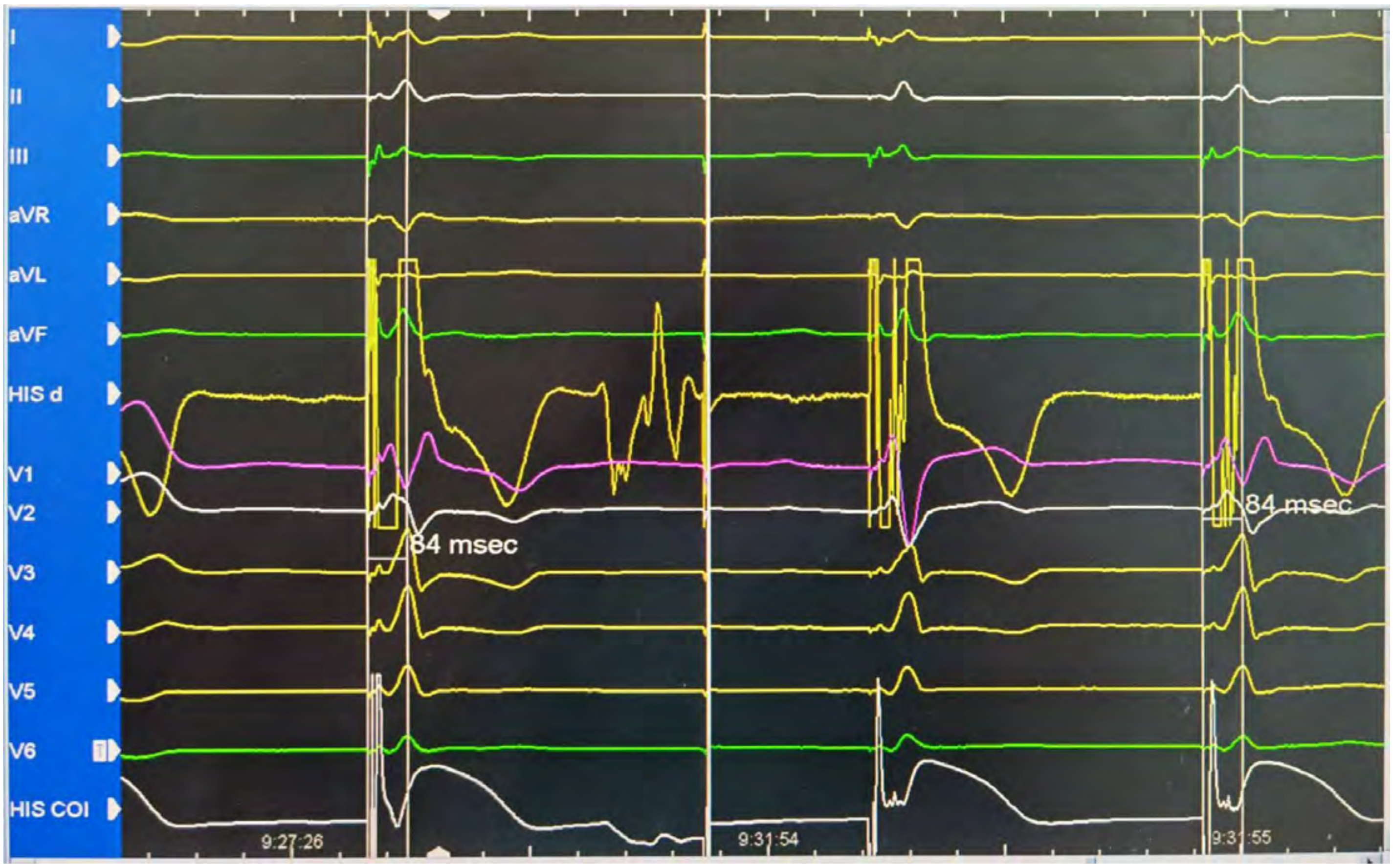

Transitions

Patient 45:
Pre-ECG

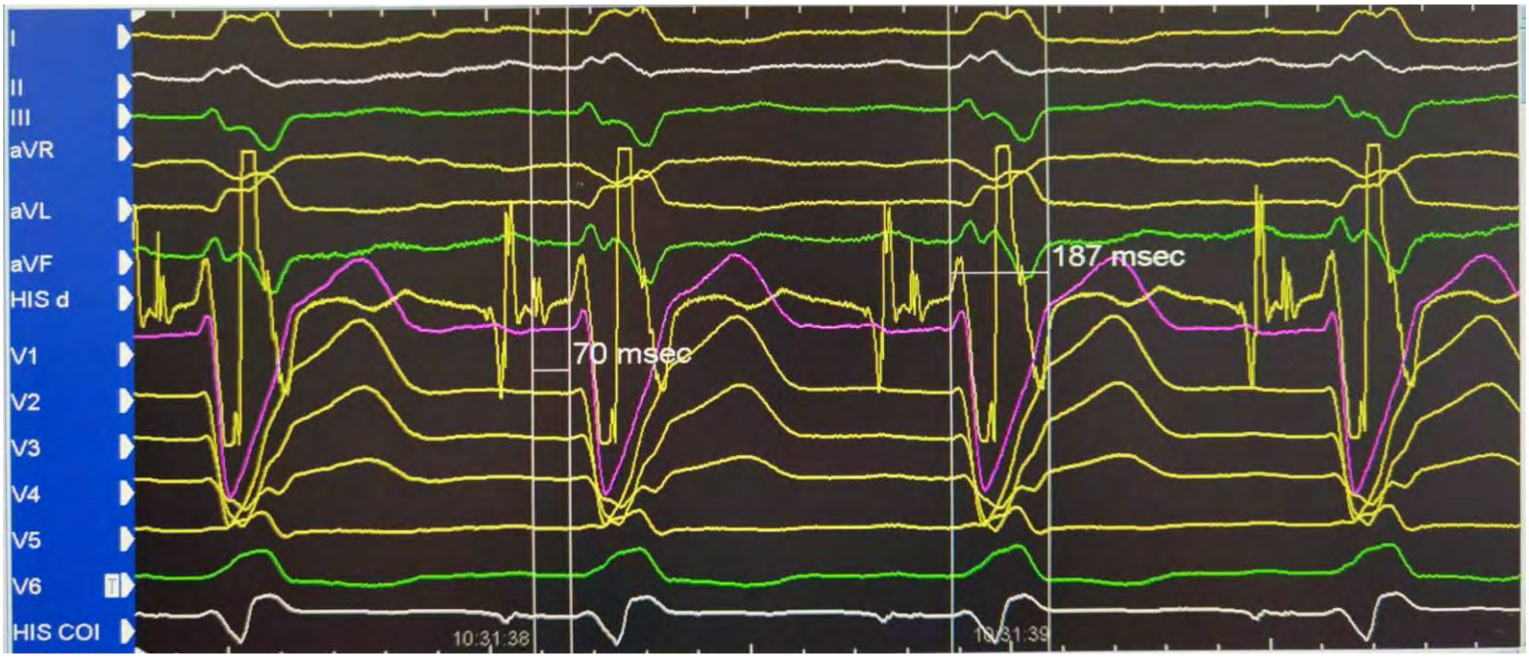

Post ECG

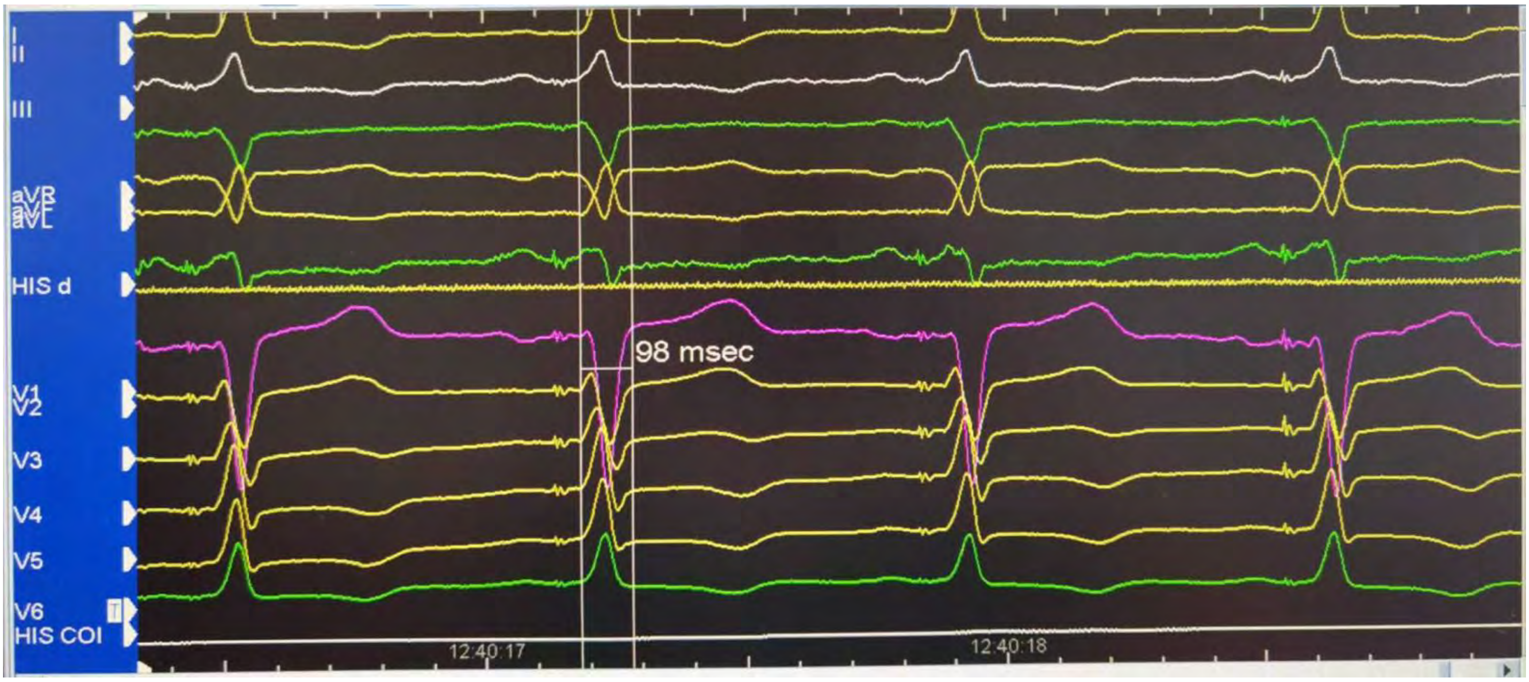

Patient 45:
Transitions

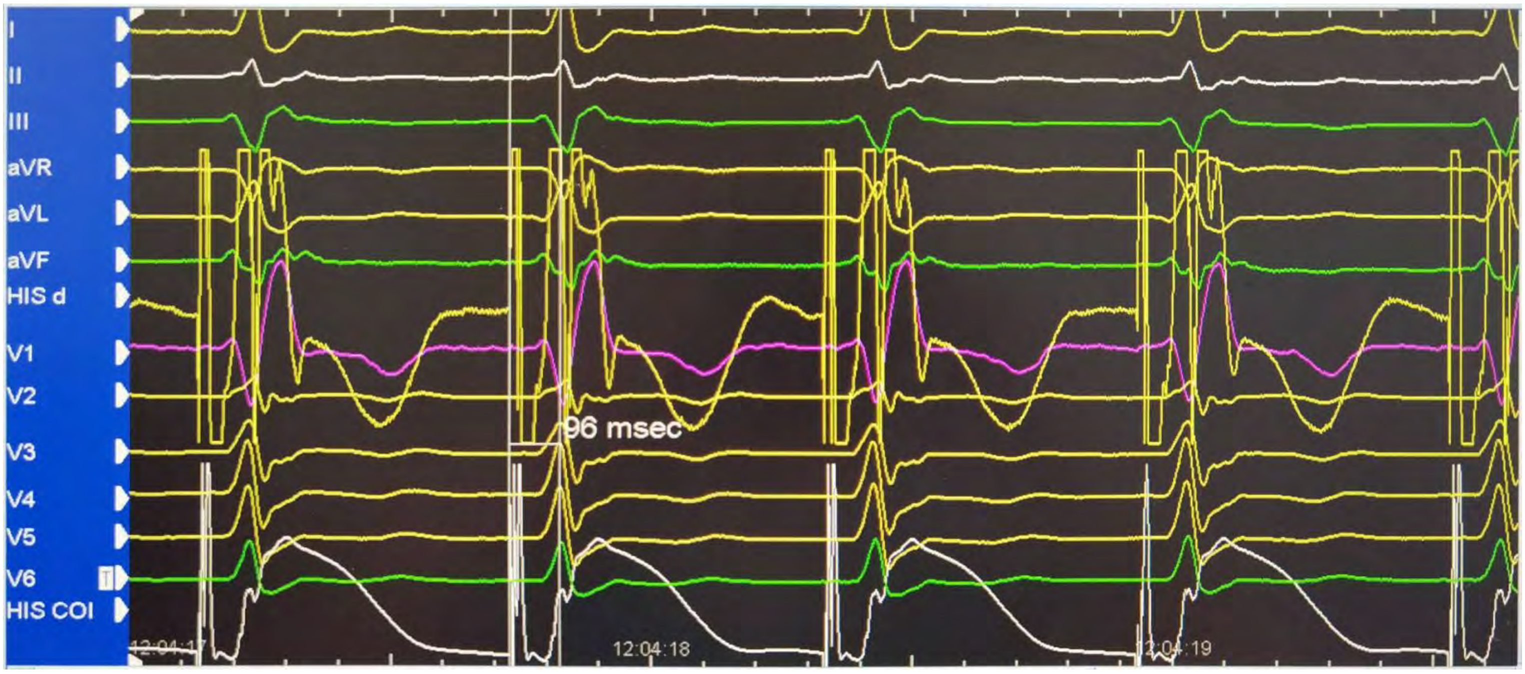

Transitions

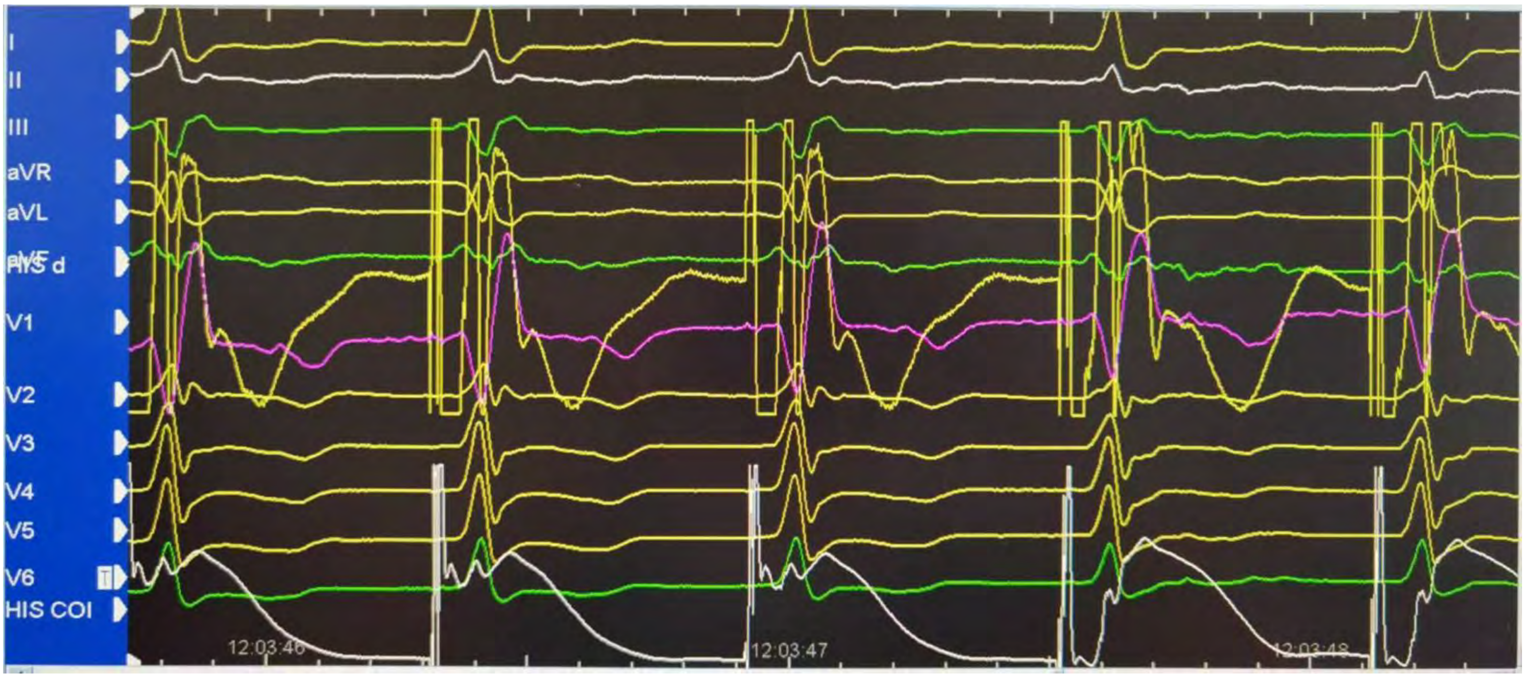

Patient 46:
Pre-ECG

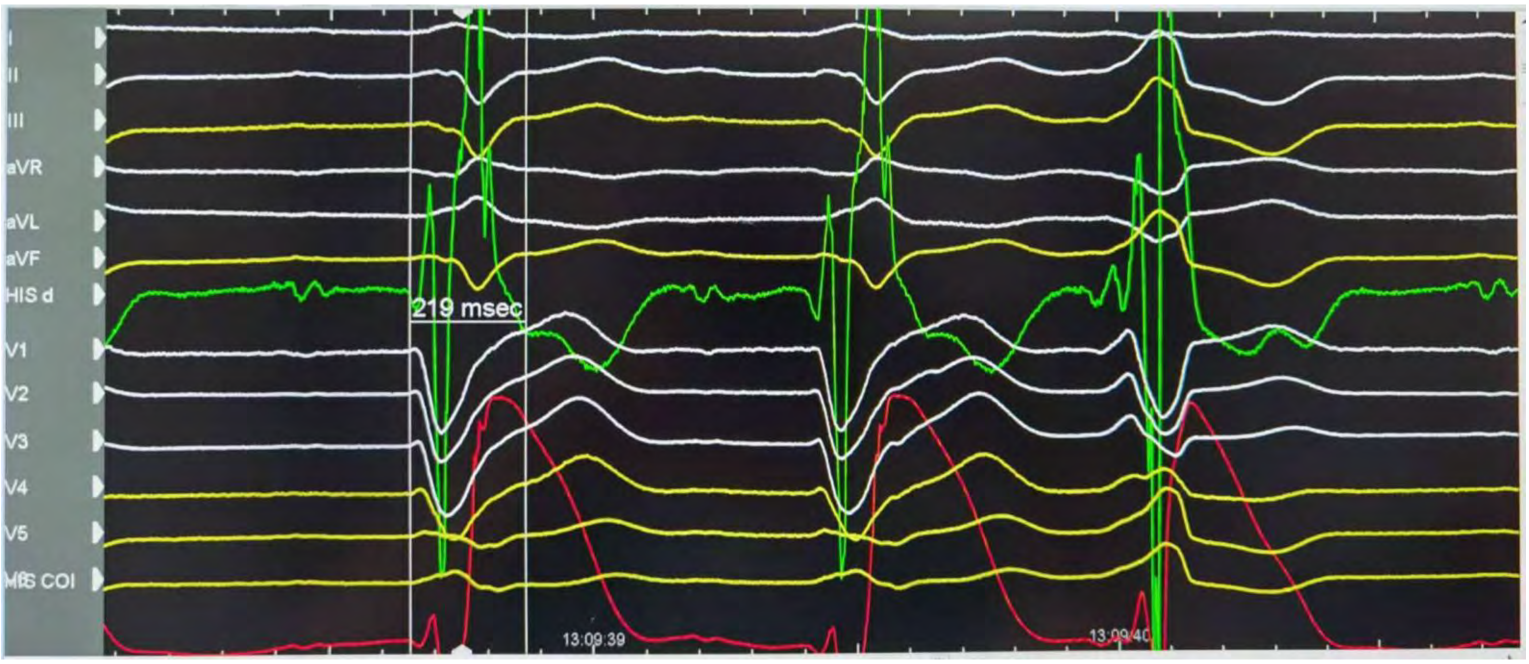

Post ECG

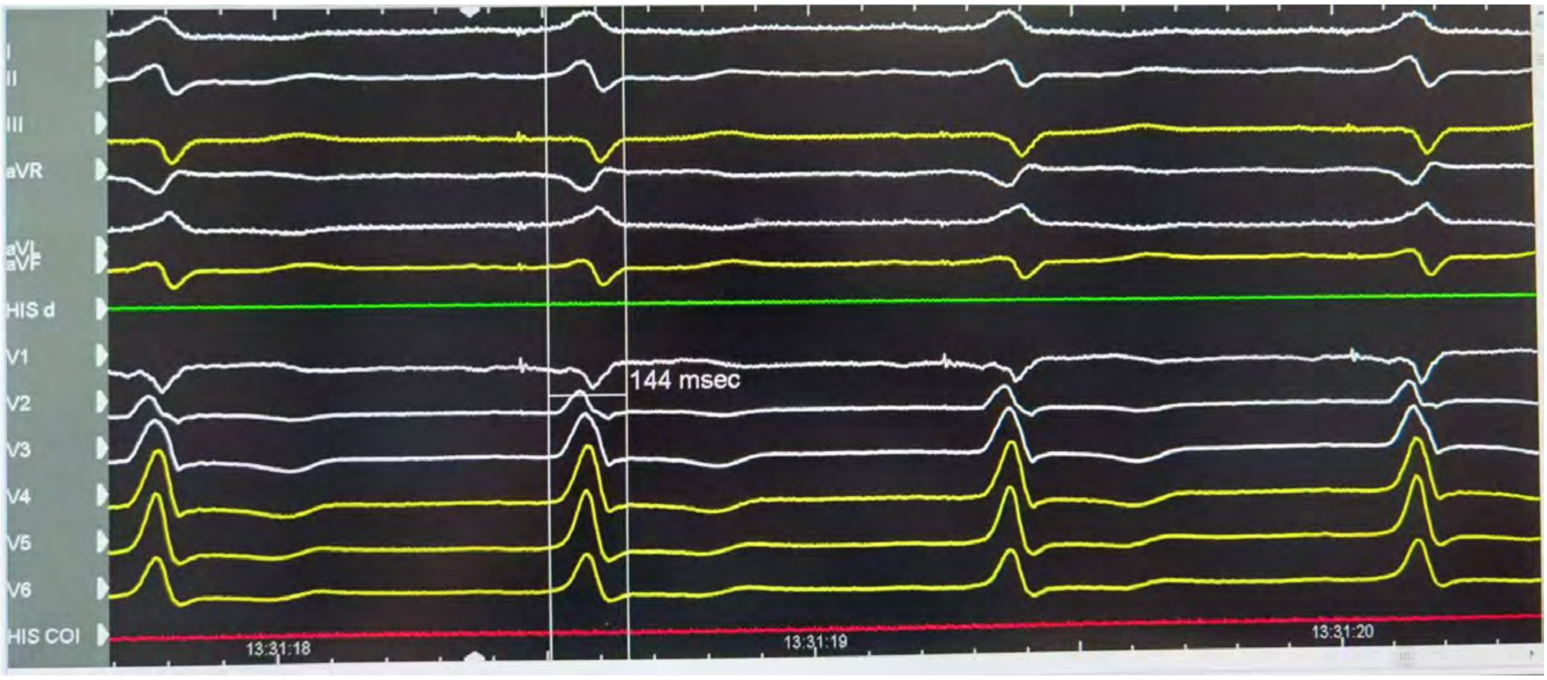

Patient 46:
Transitions

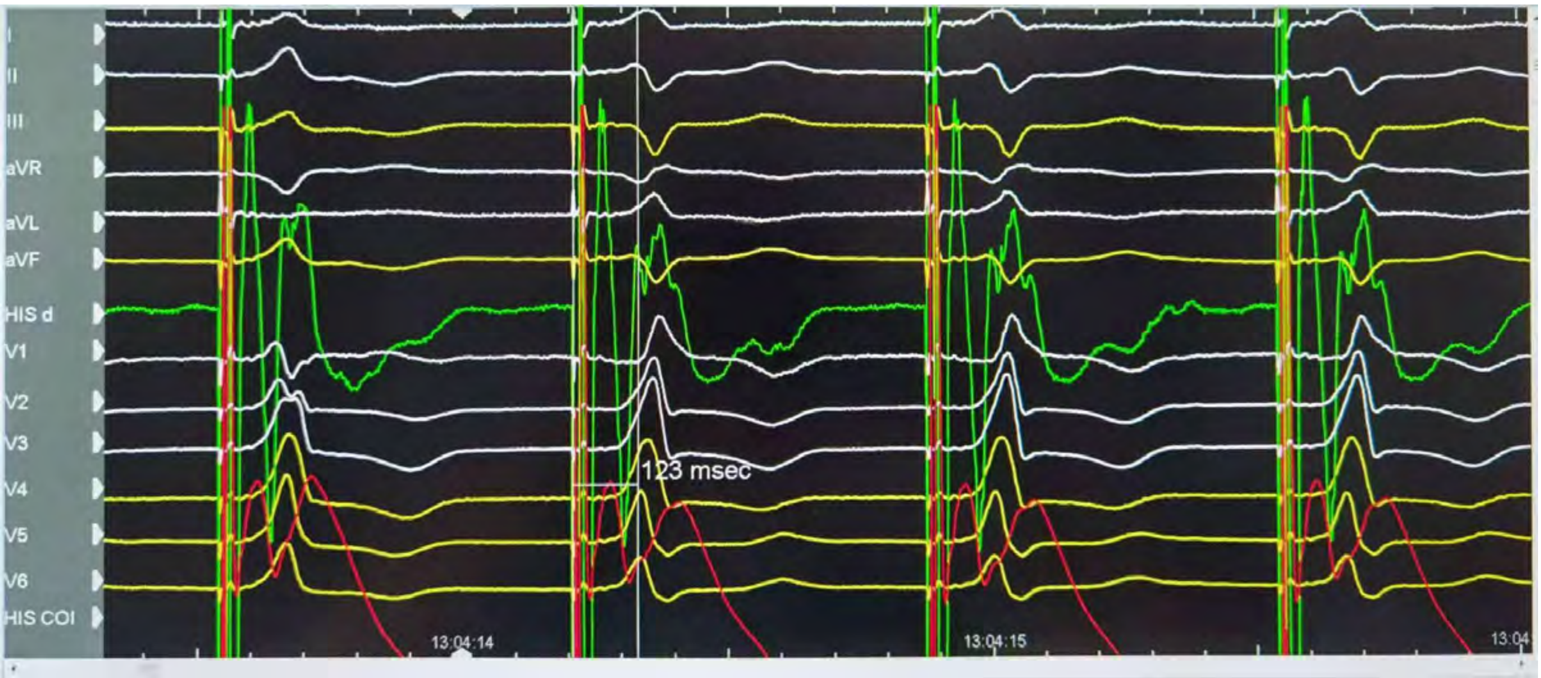

Transitions

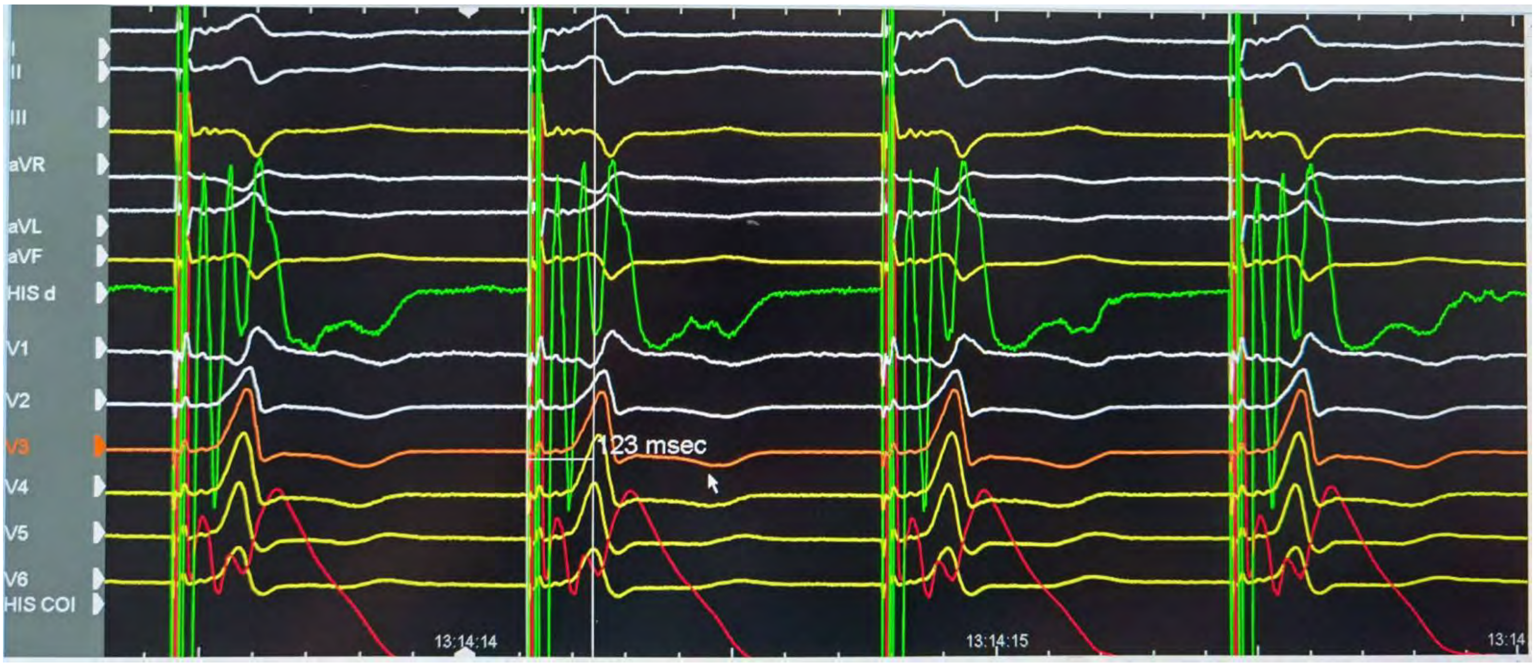

Patient 47:
Pre-ECG

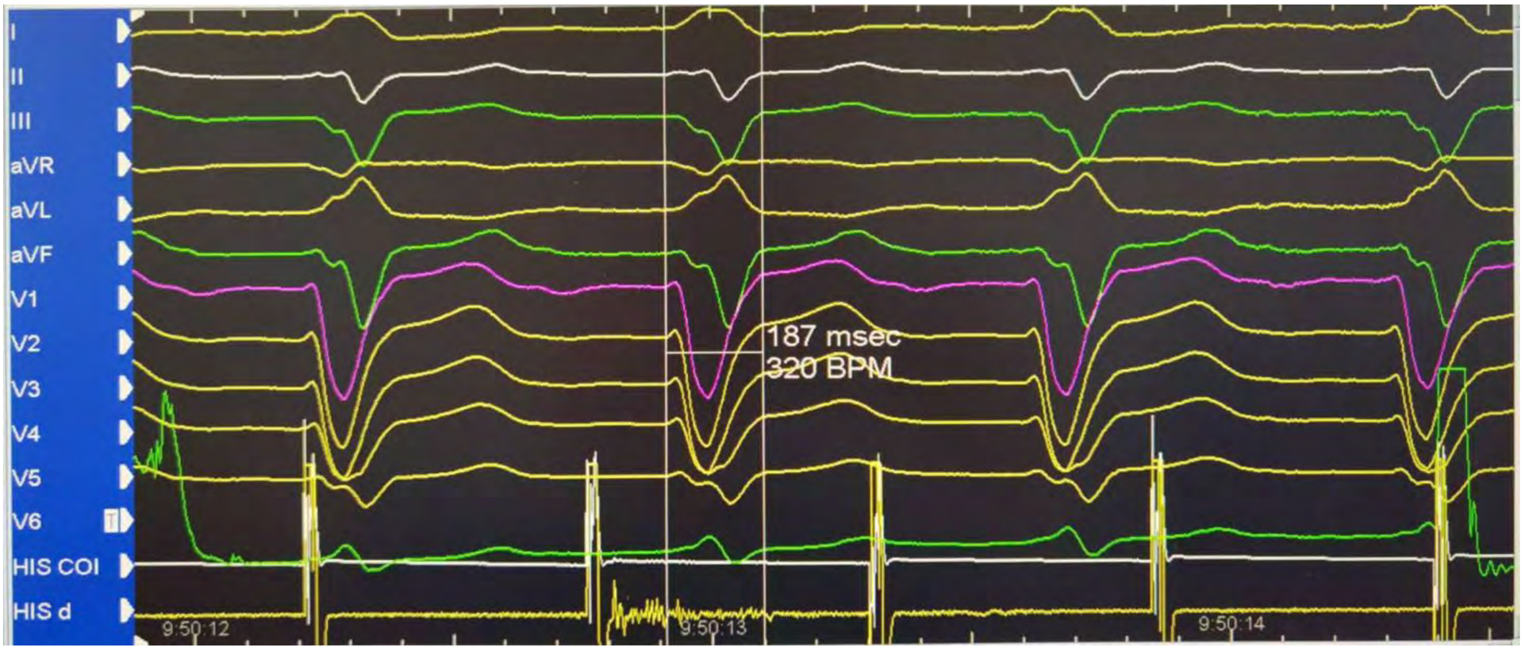

Post ECG

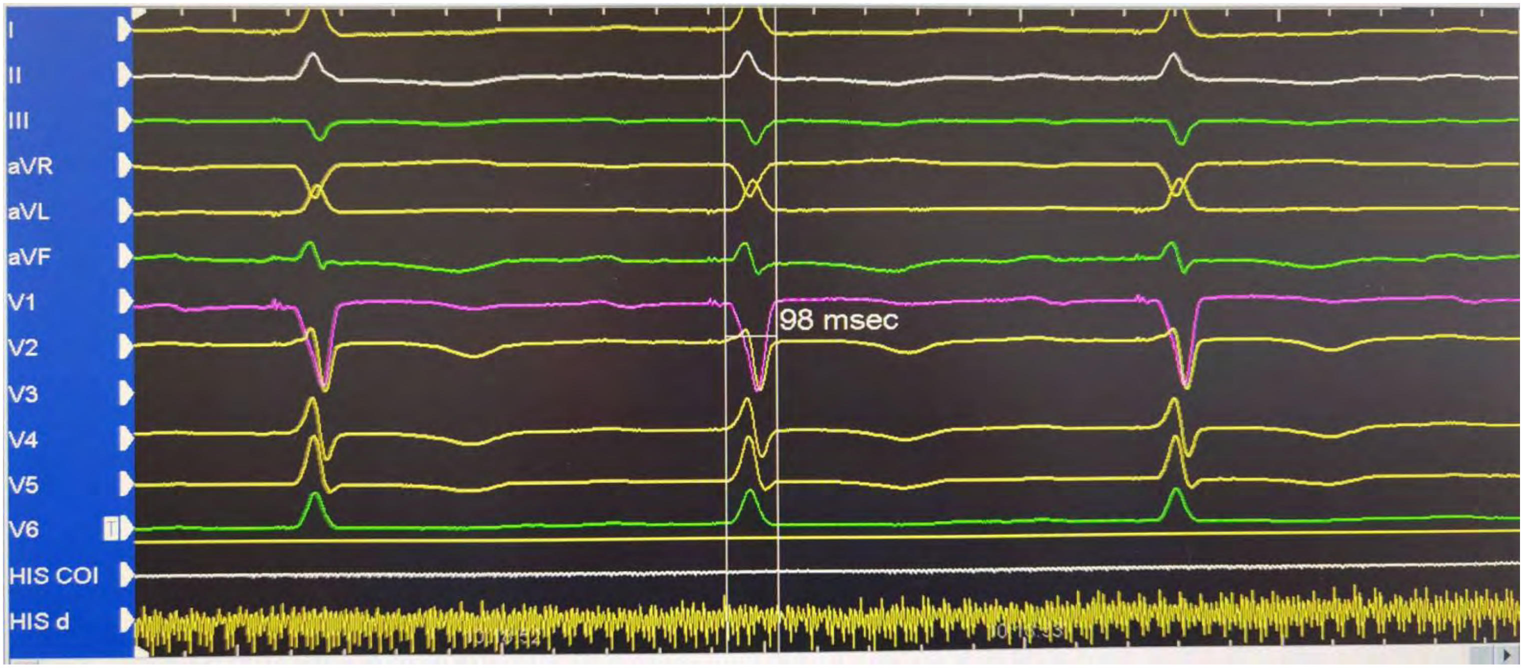

Patient 47:
Transitions

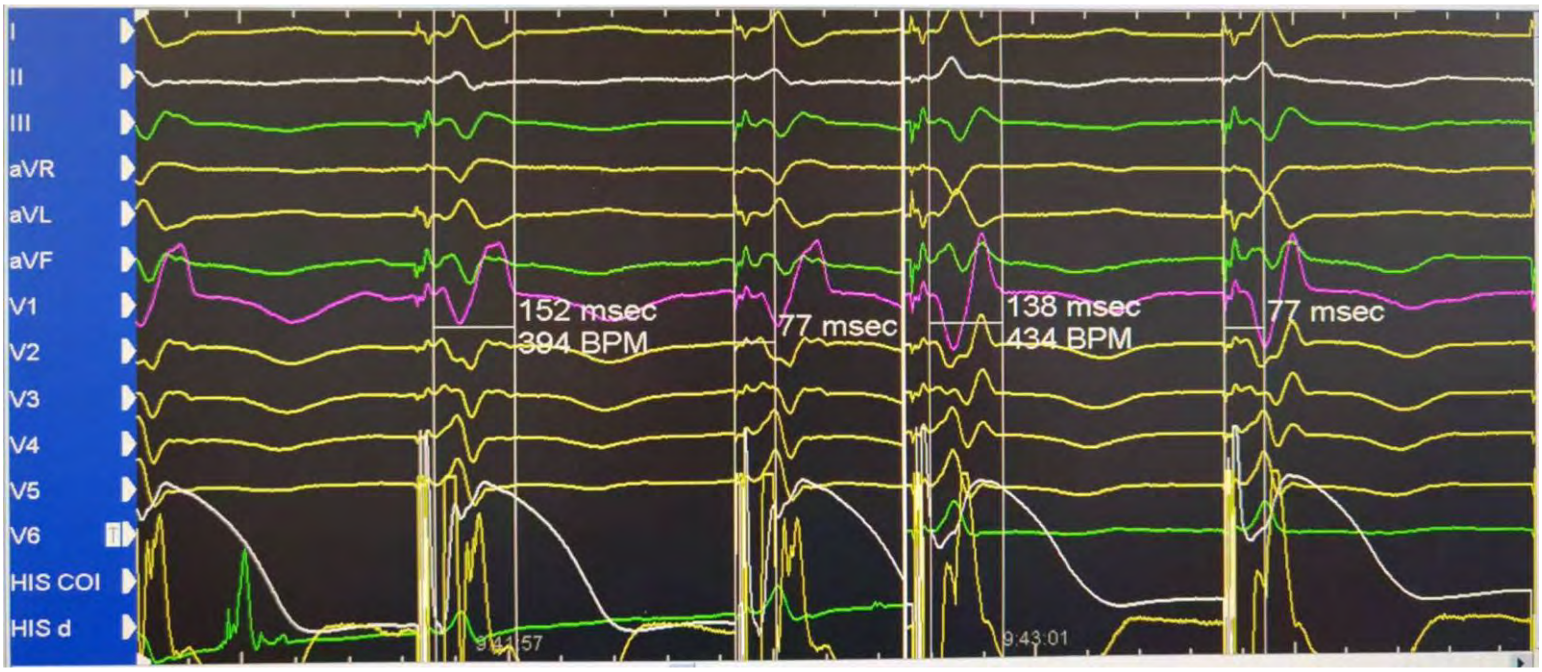

Transitions

Patient 48:
Pre-ECG

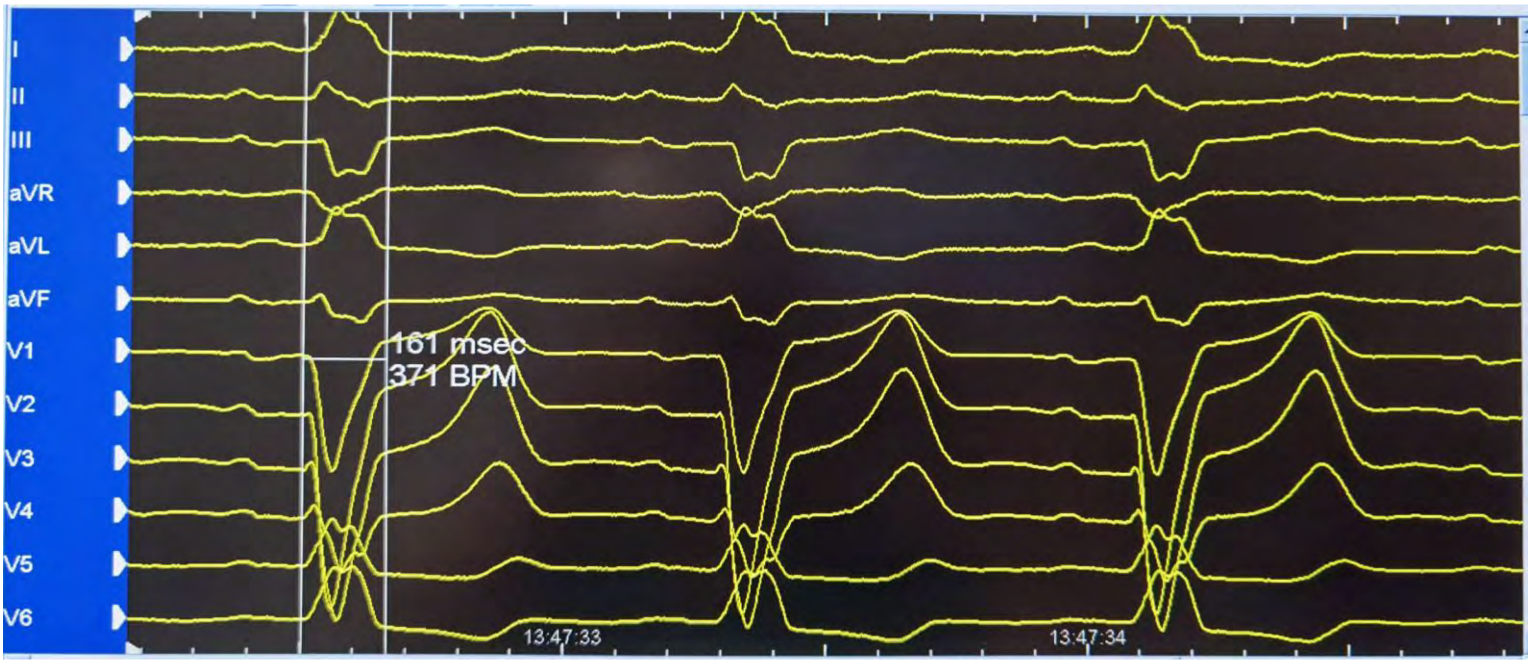

Post ECG

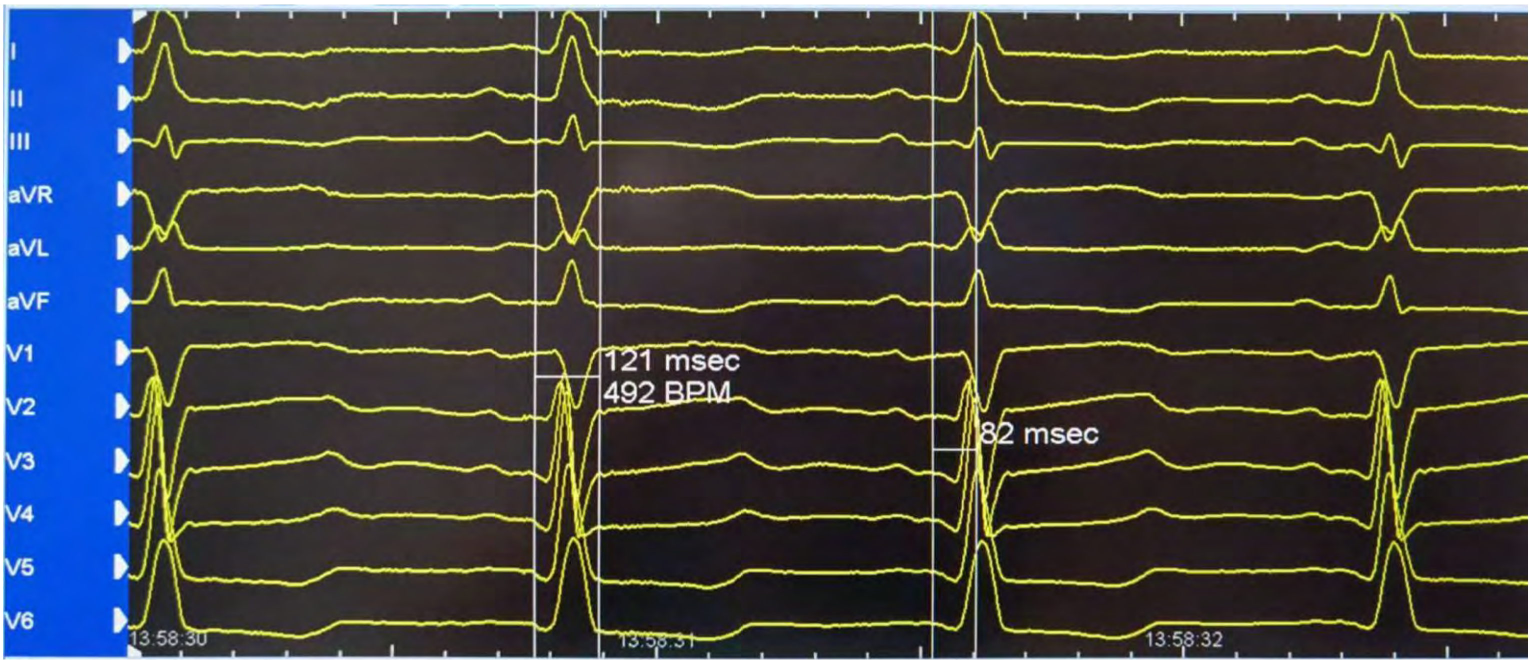

Patient 48:
Transitions

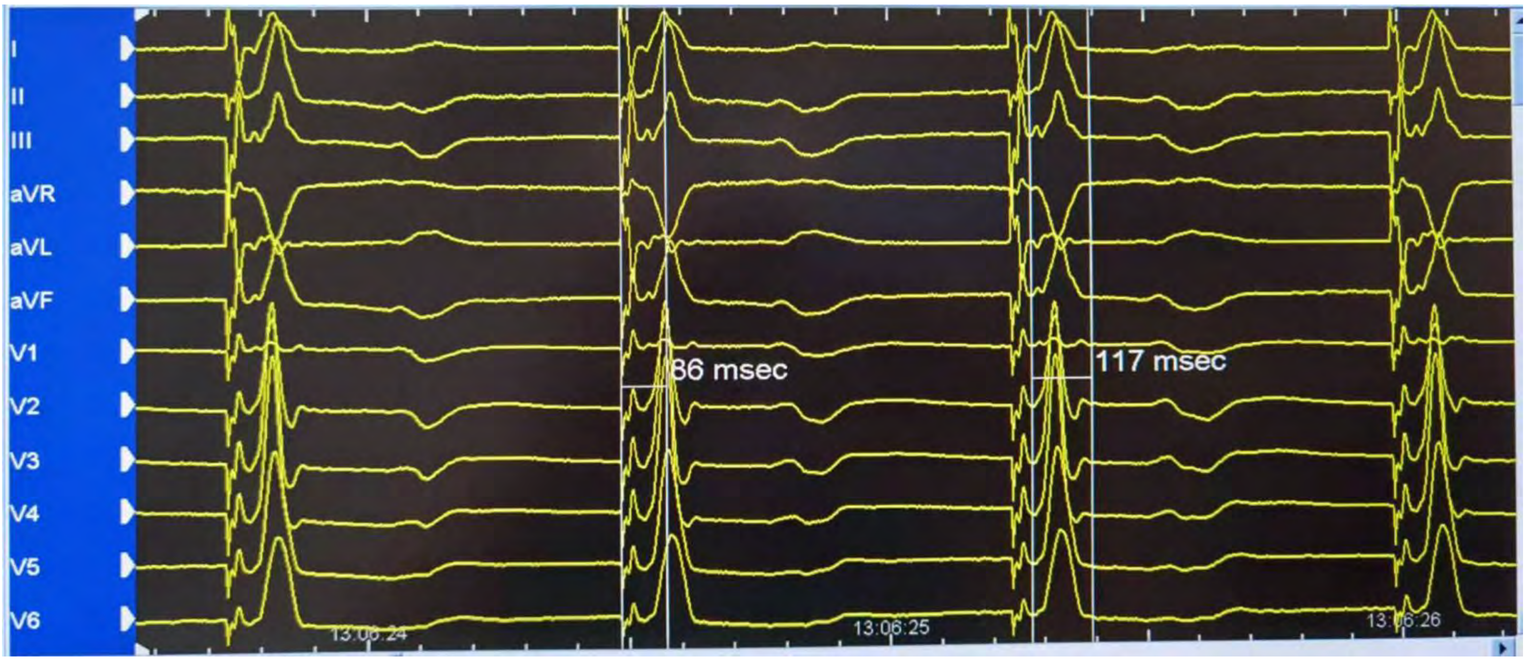

Transitions

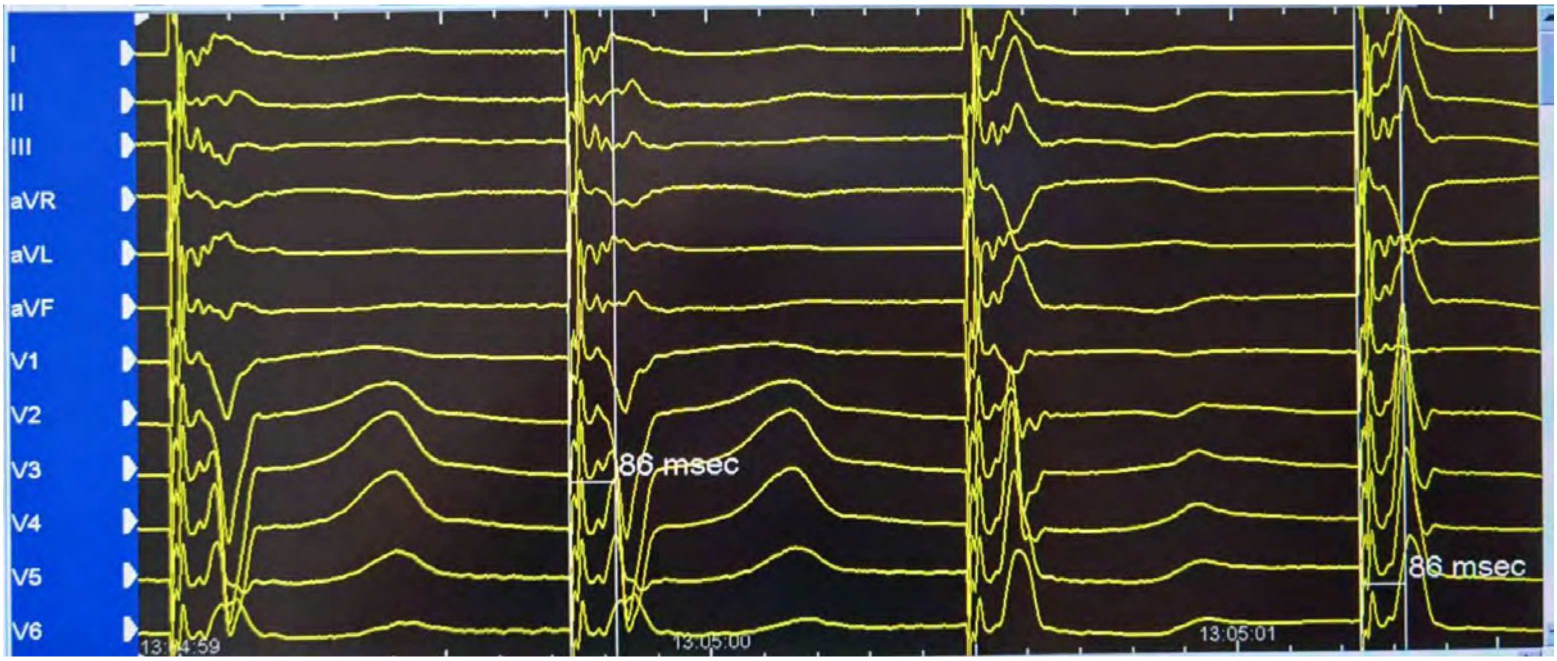

Patient 49:
Pre-ECG

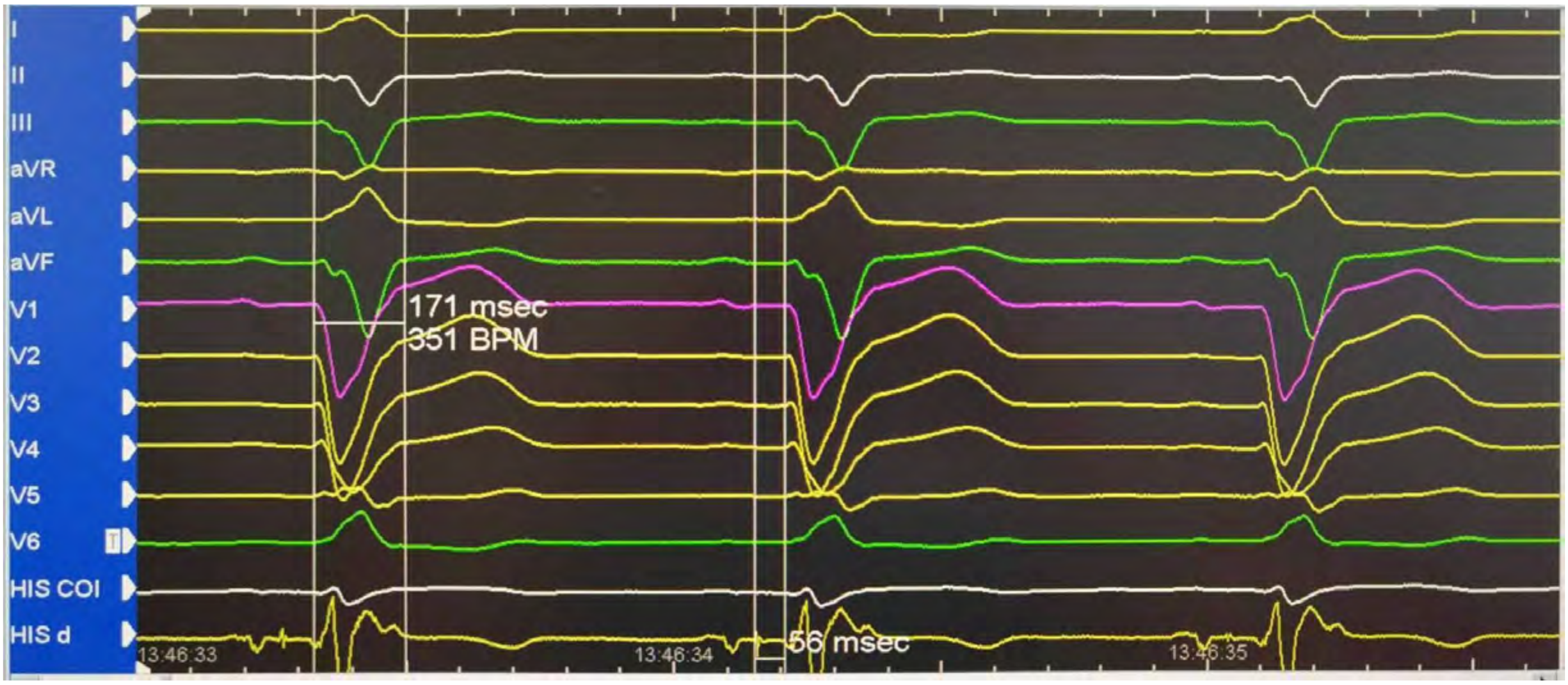

Post ECG

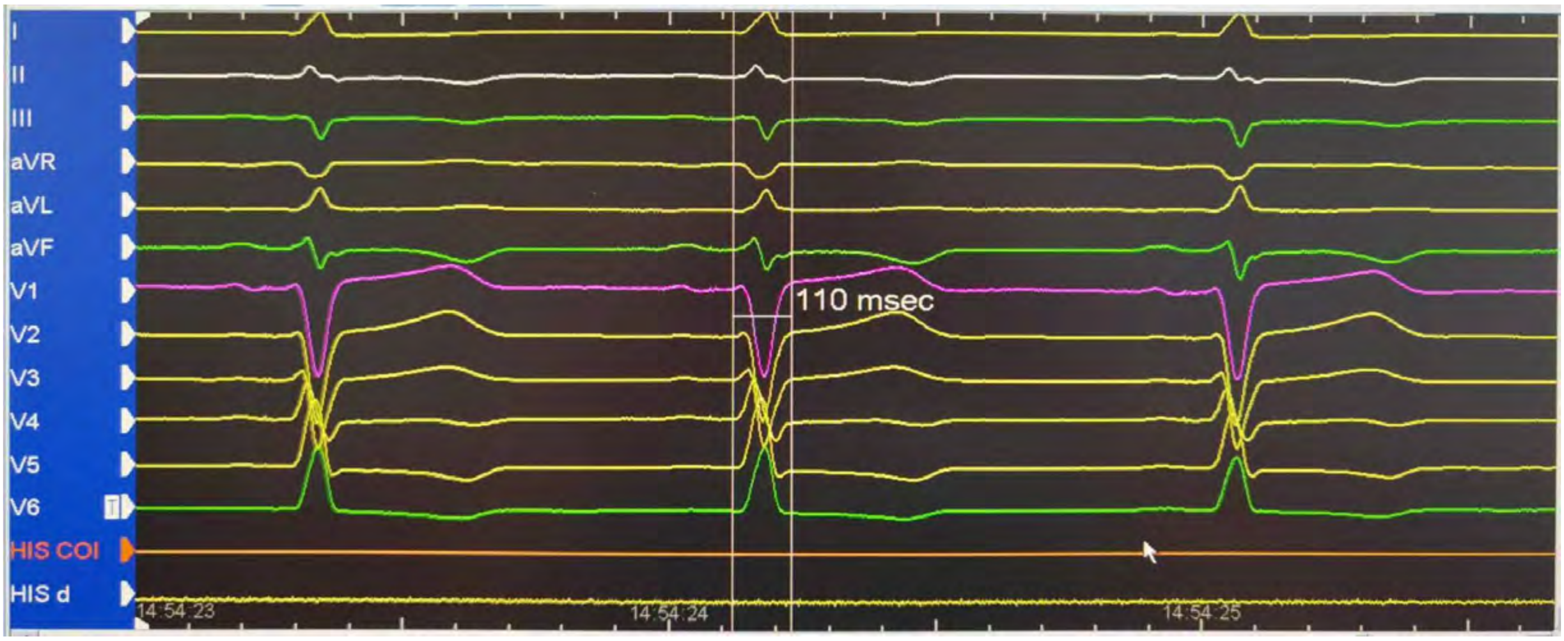

Patient 49:
Transitions

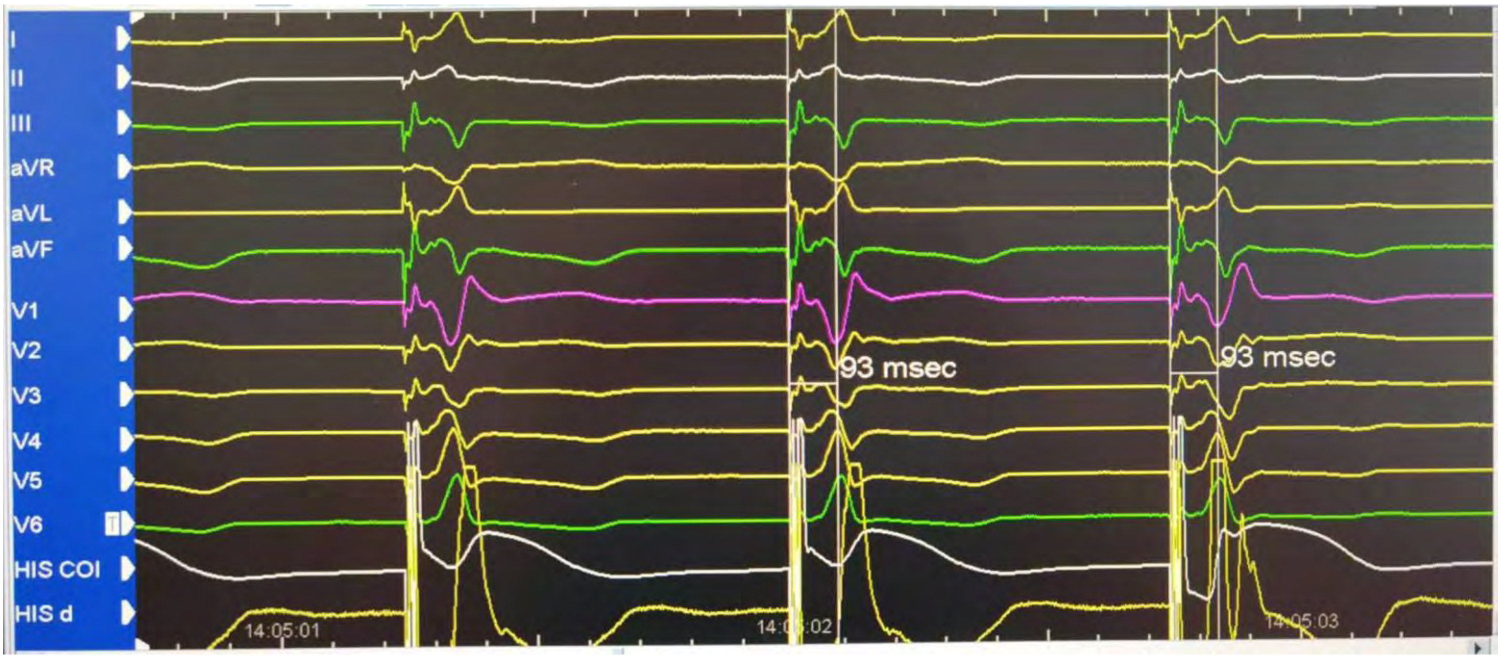

Transitions

Patient 50:
Pre-ECG

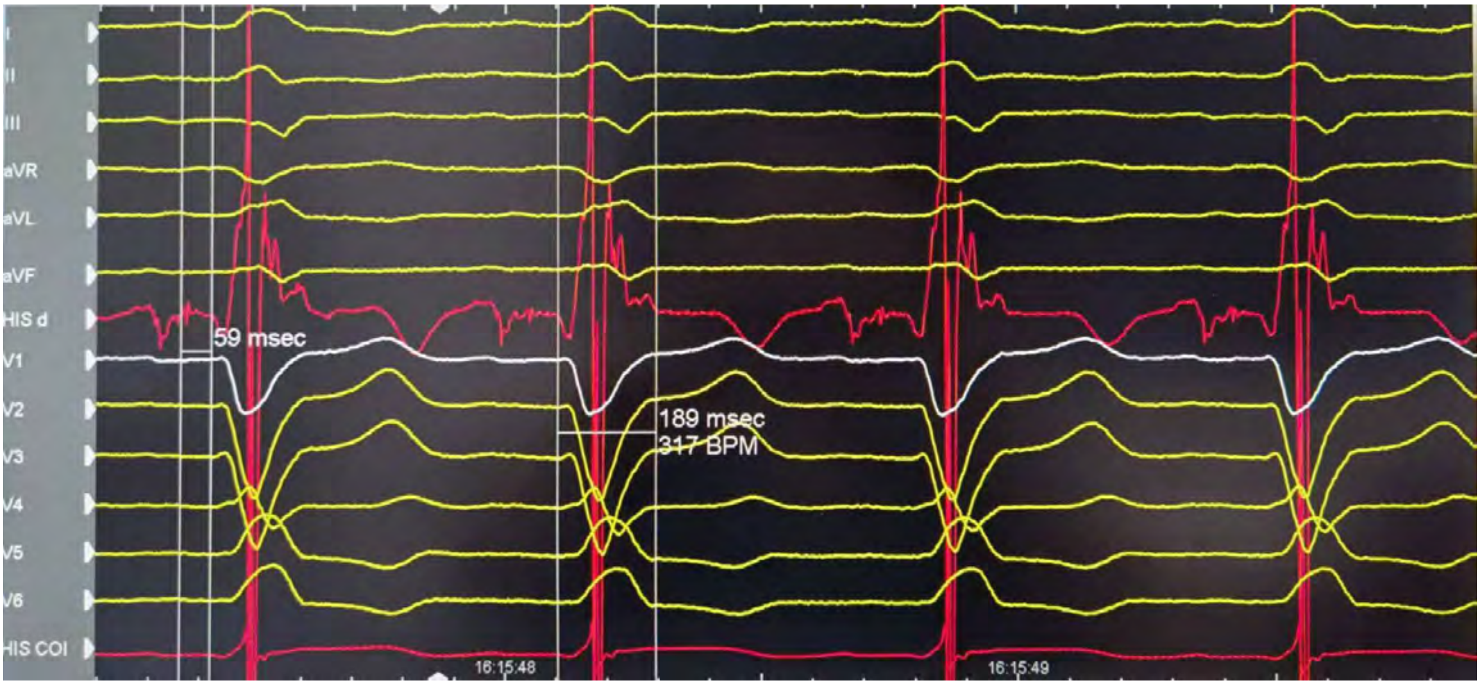

Post ECG

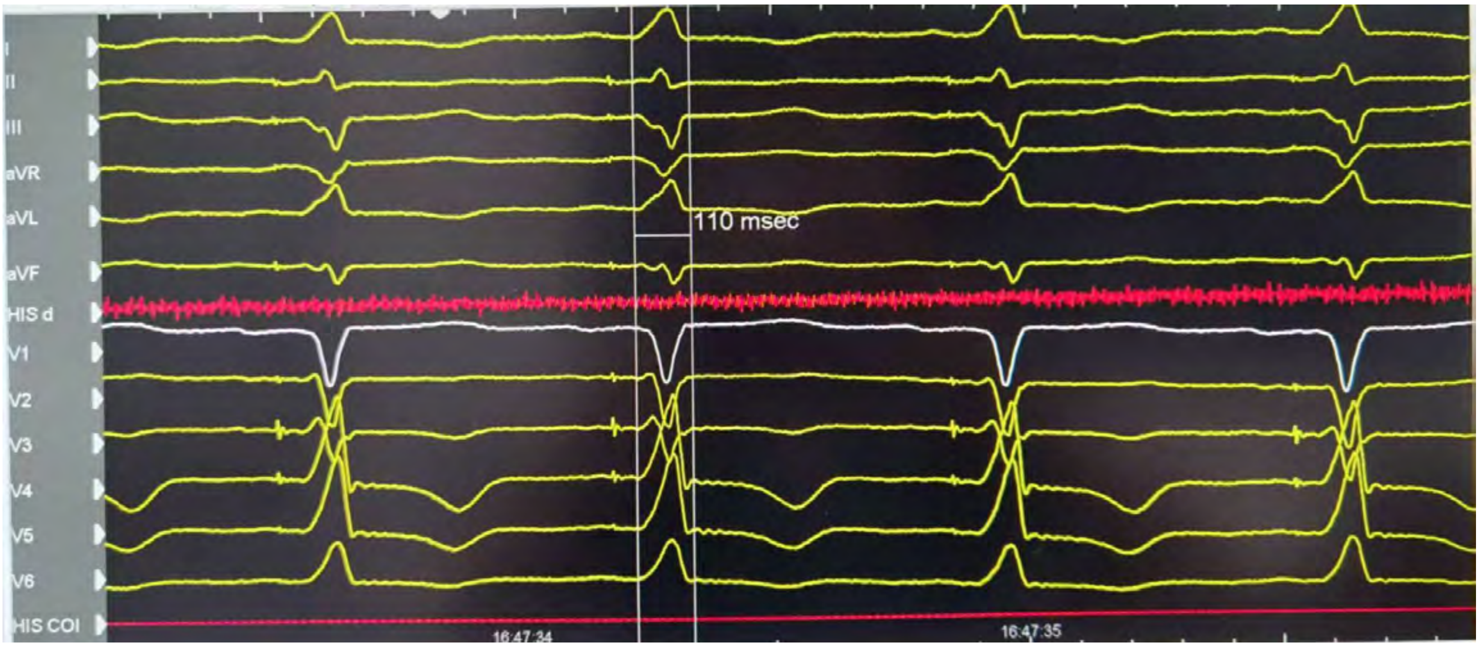

Patient 50:
Transitions

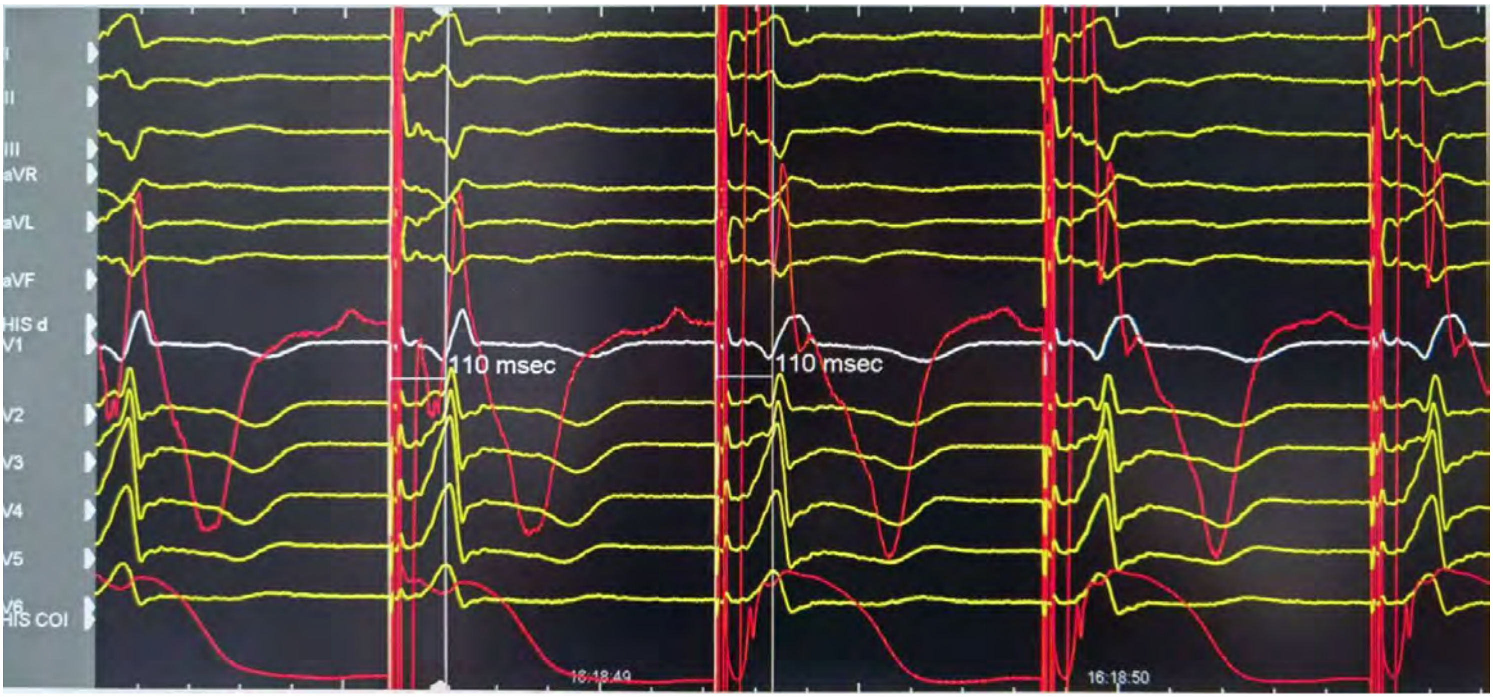

Transitions

Patient 51:
Pre-ECG

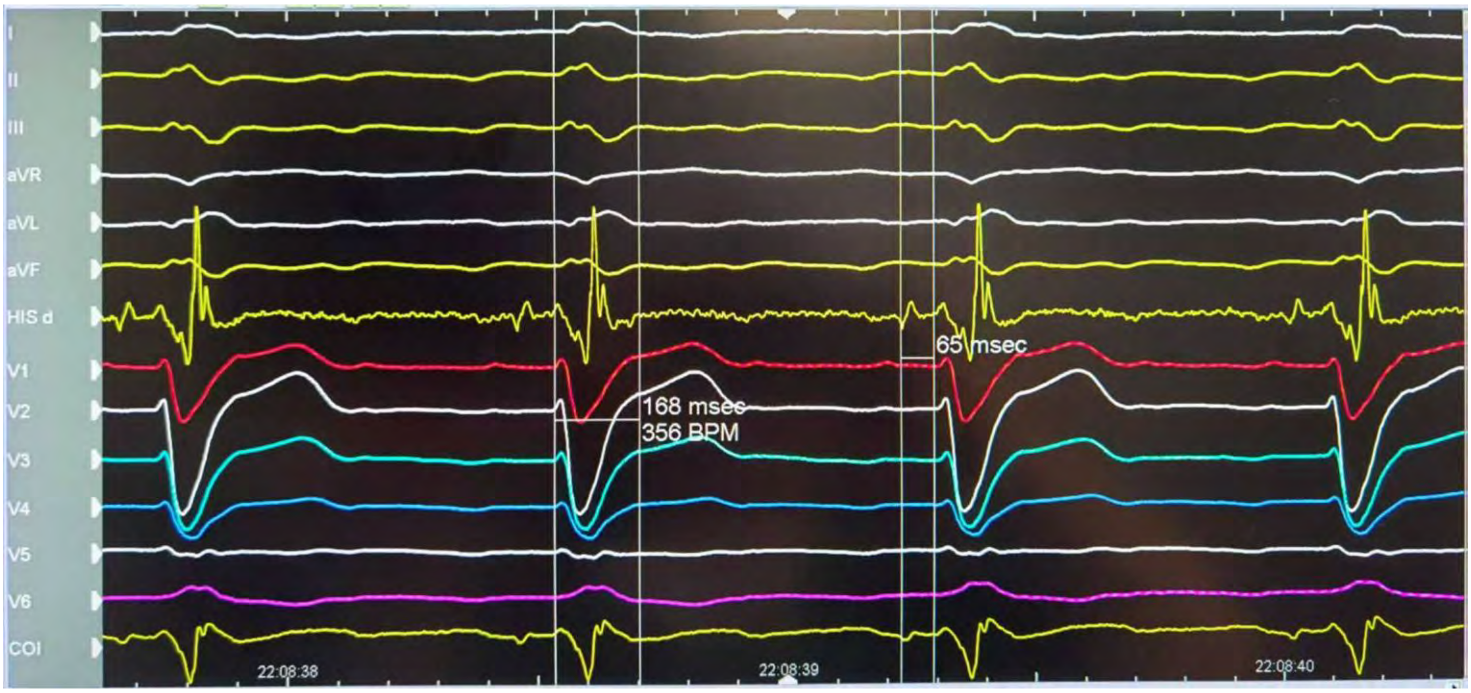

Transitions

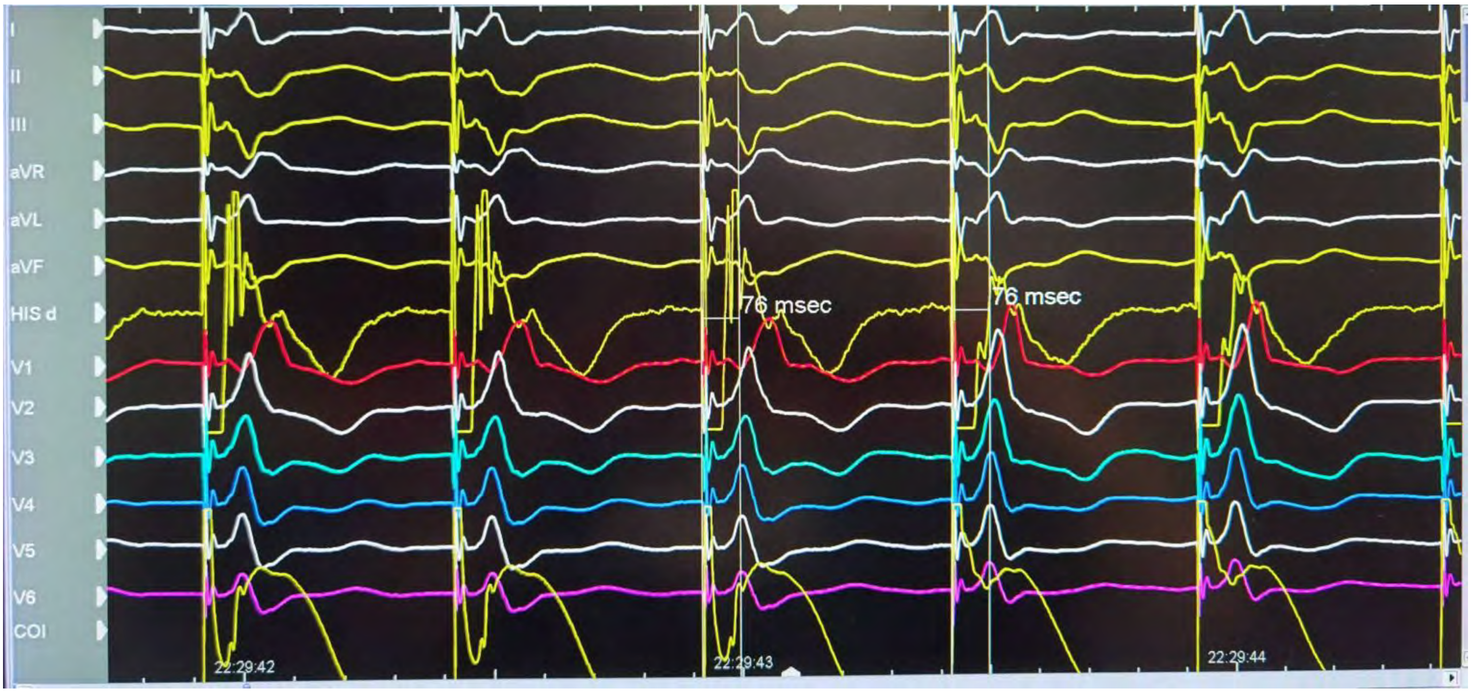

Patient 52:
Pre-ECG

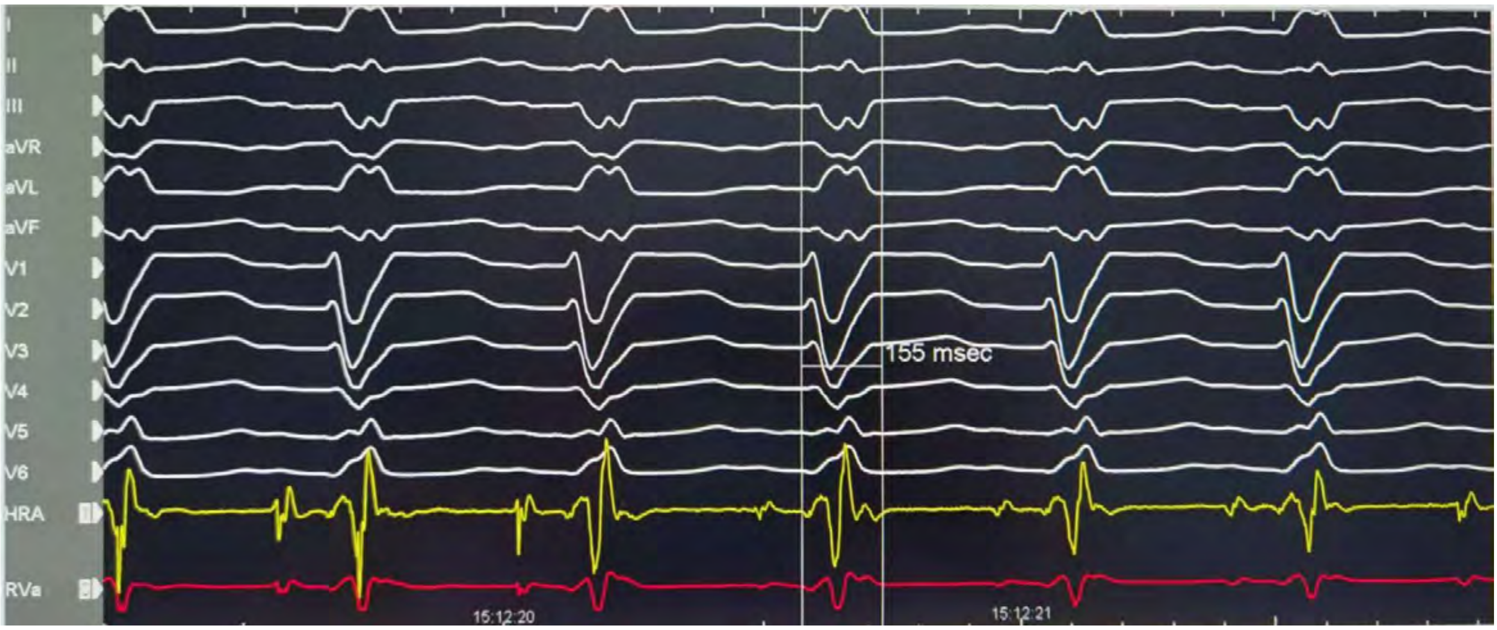

Post ECG

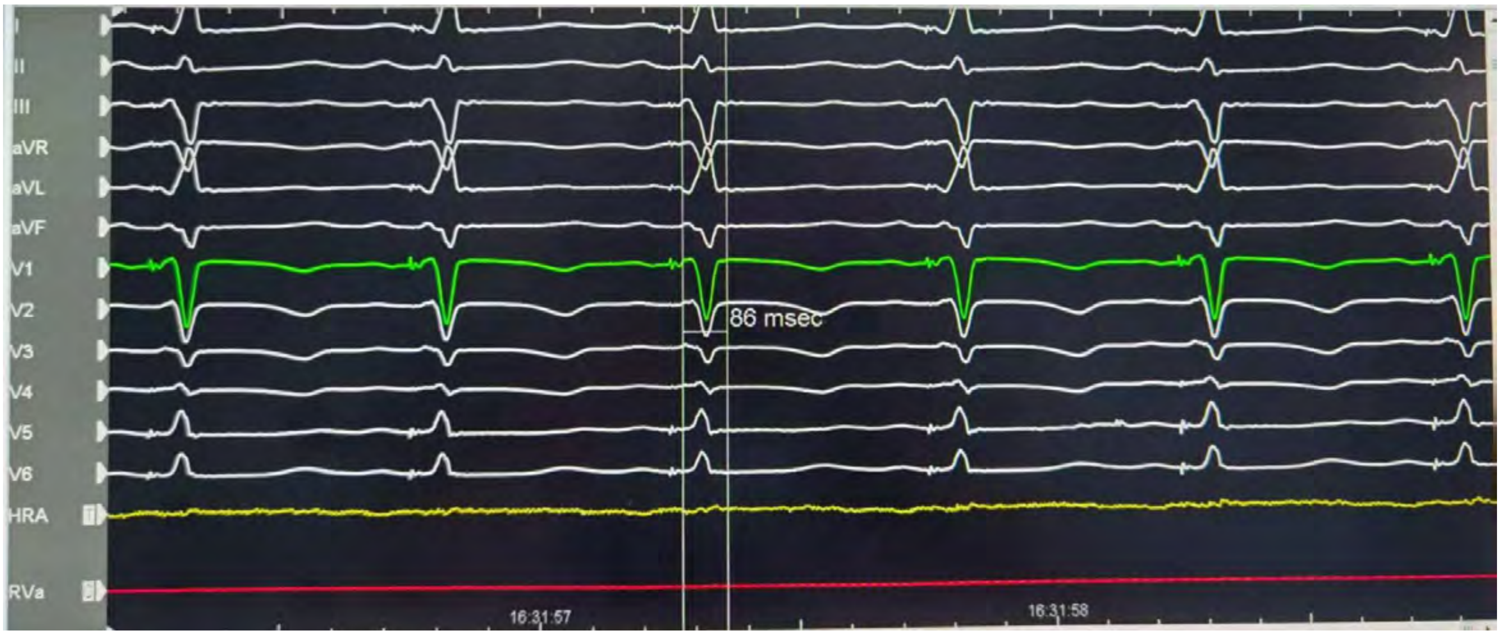

Patient 52:
Transitions

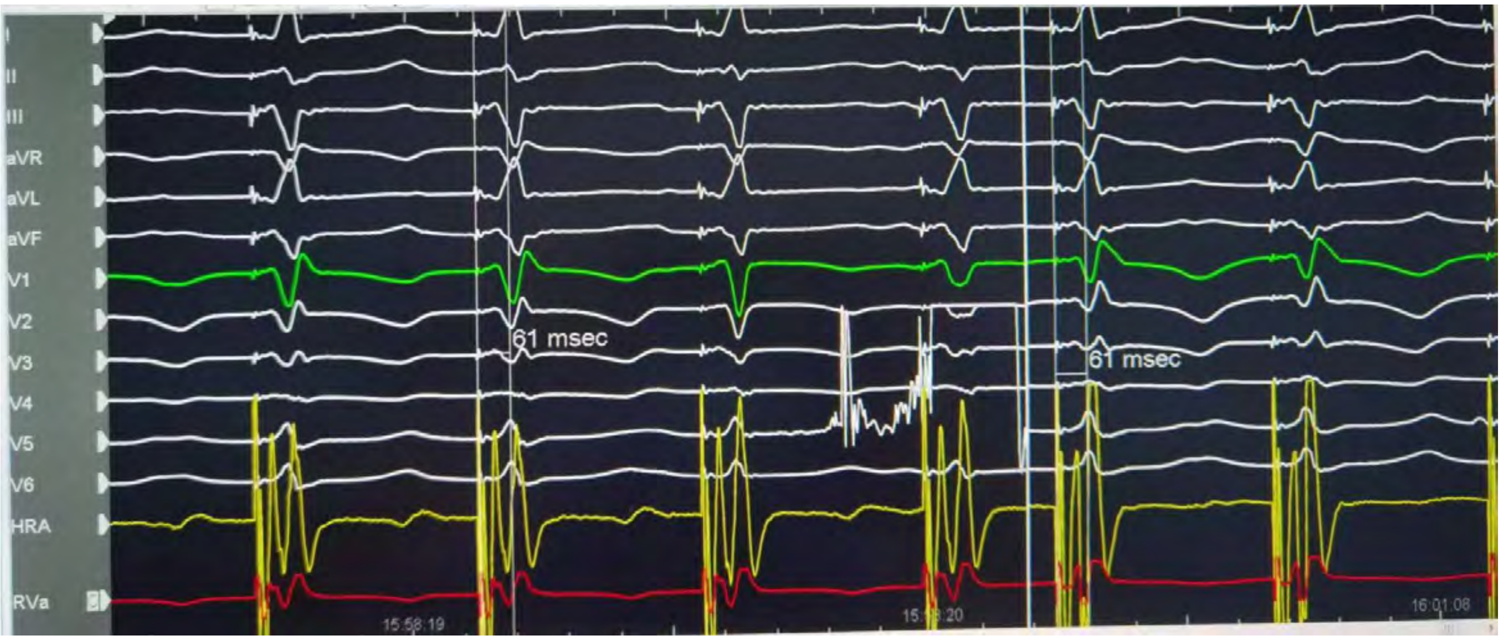

Transitions

Patient 53:
Pre-ECG

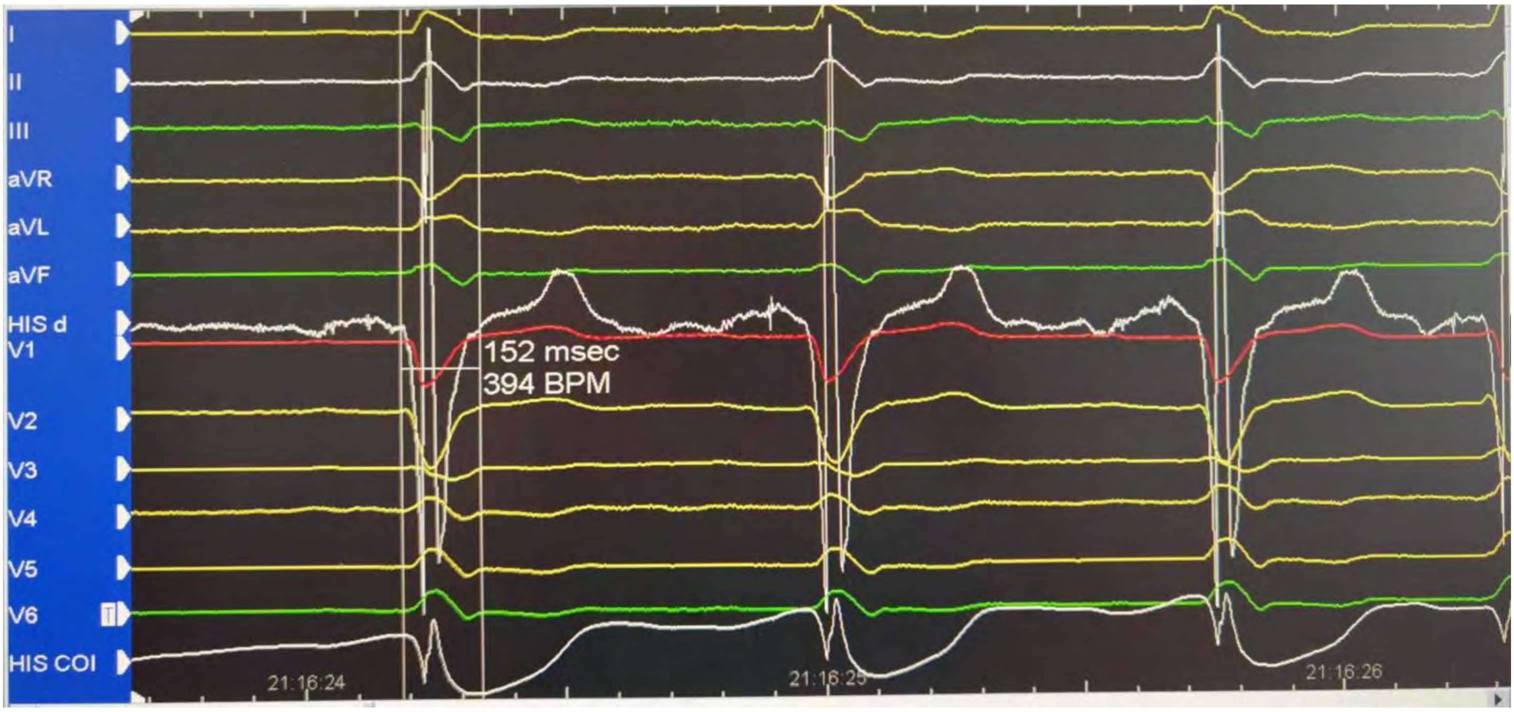

Post ECG

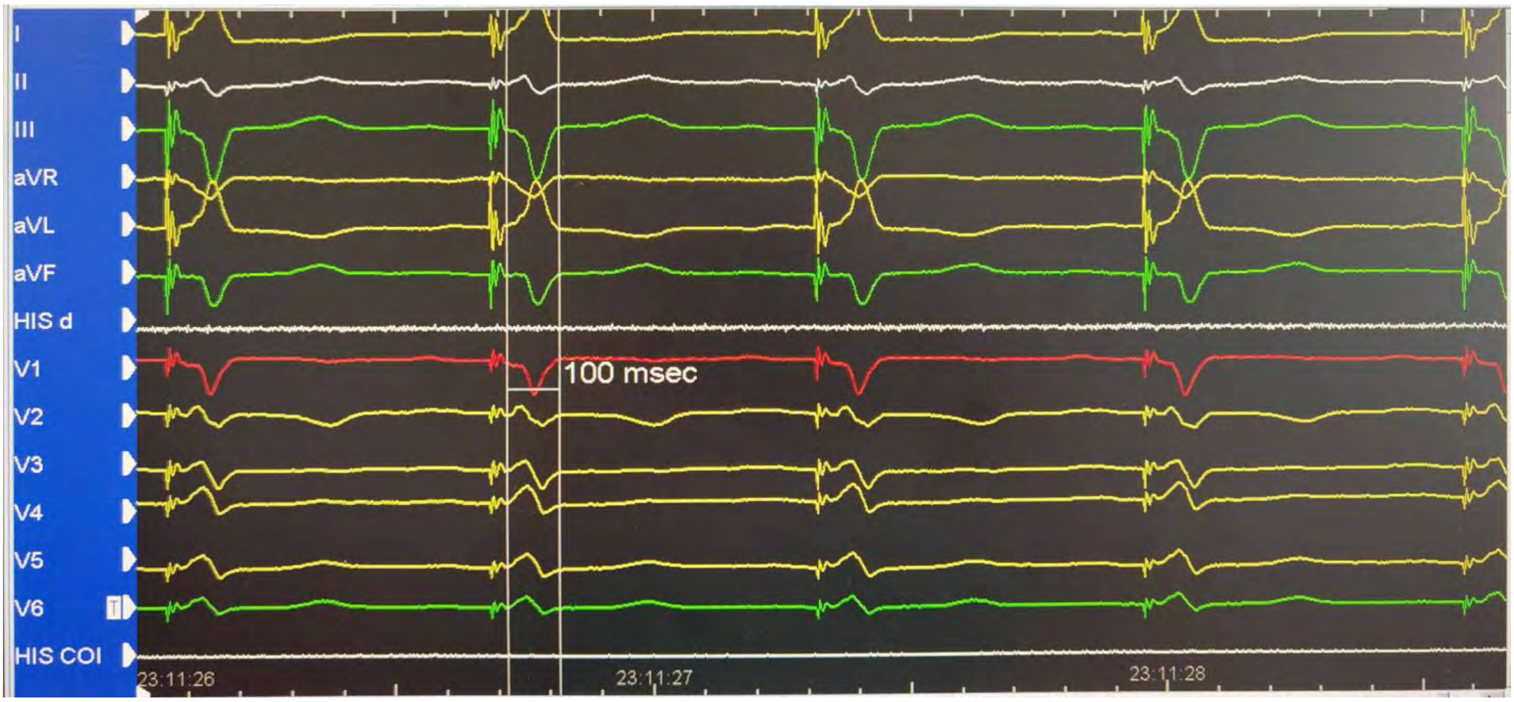

Patient 53:
Transitions

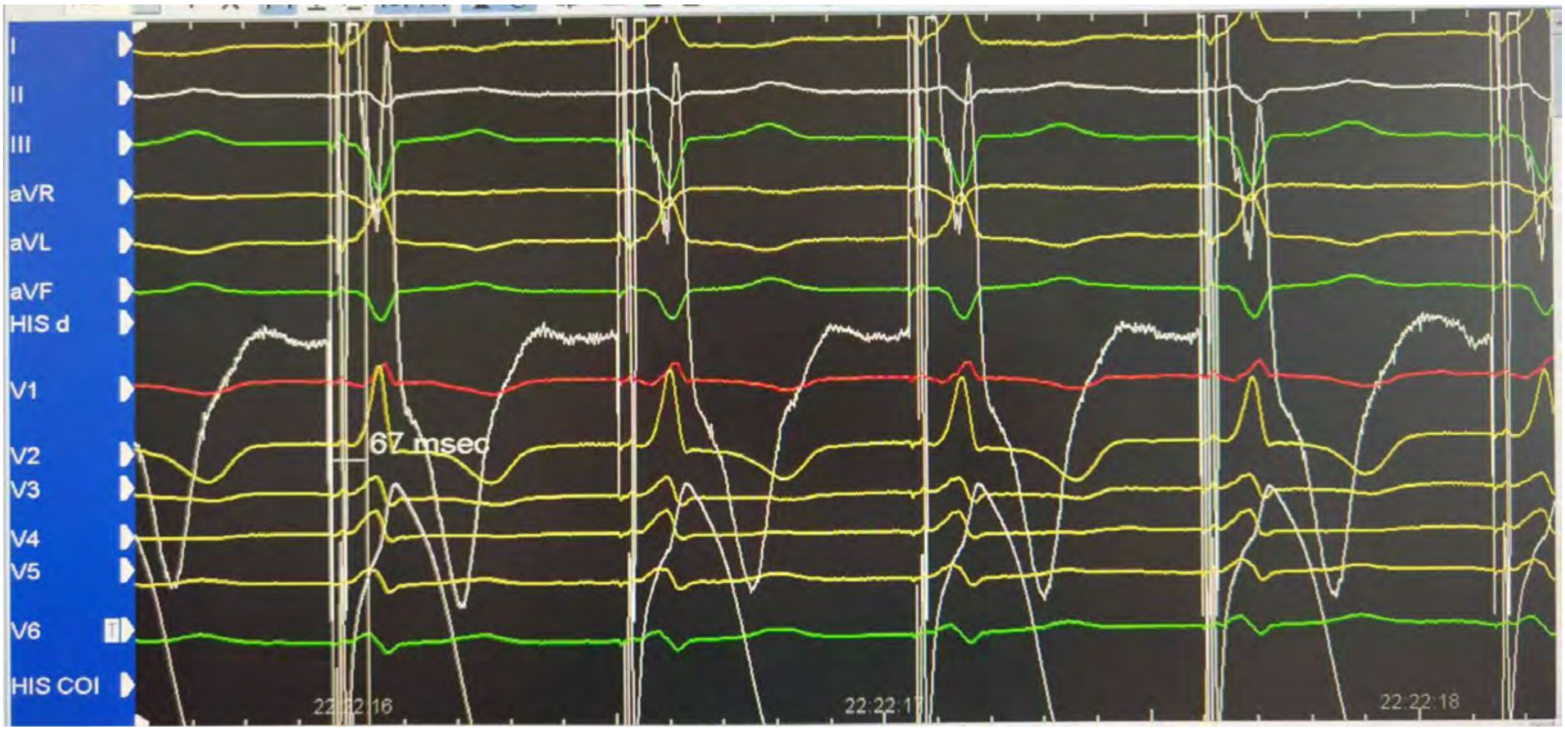

Transitions

Patient 54:
Pre-ECG

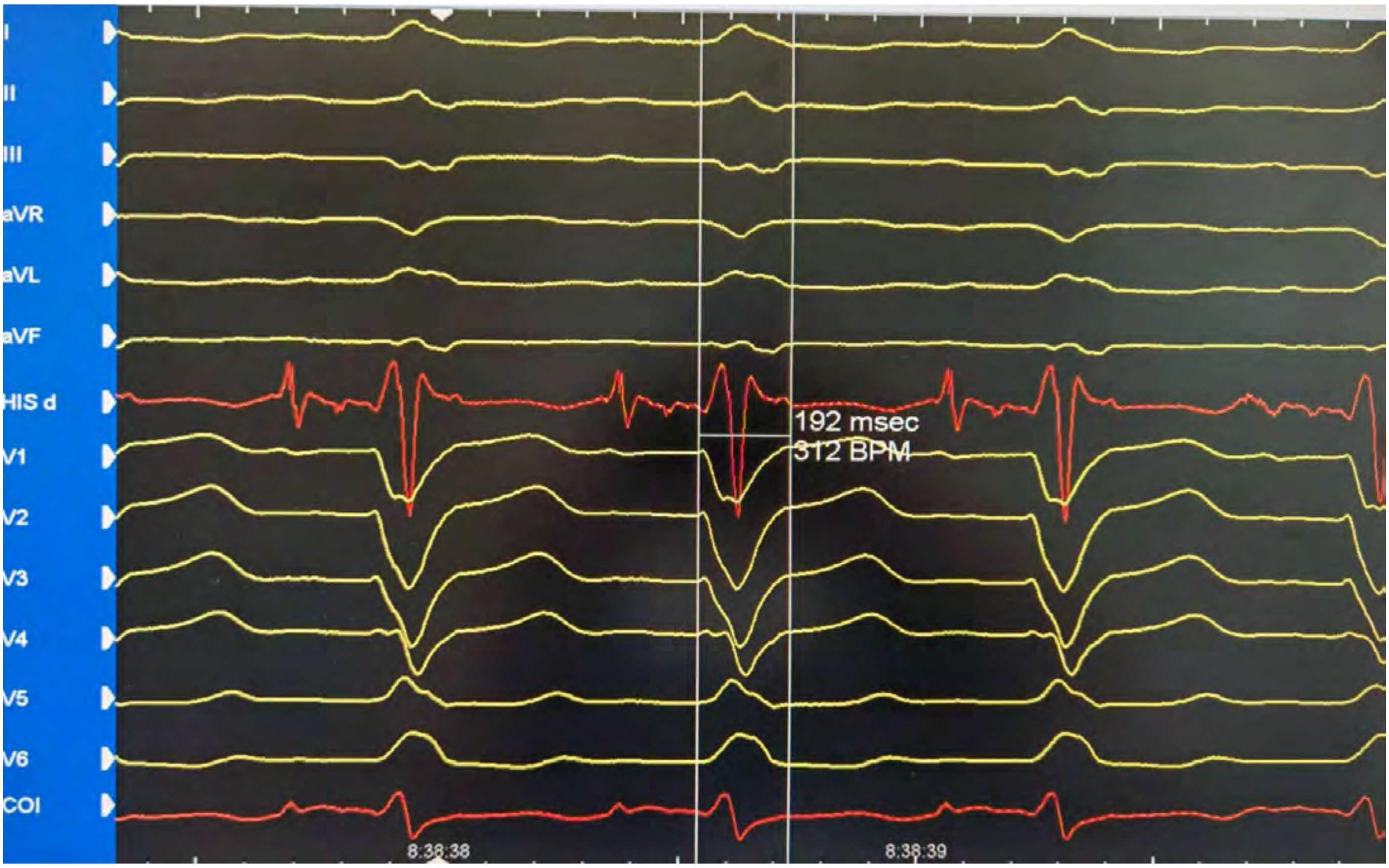

Post ECG

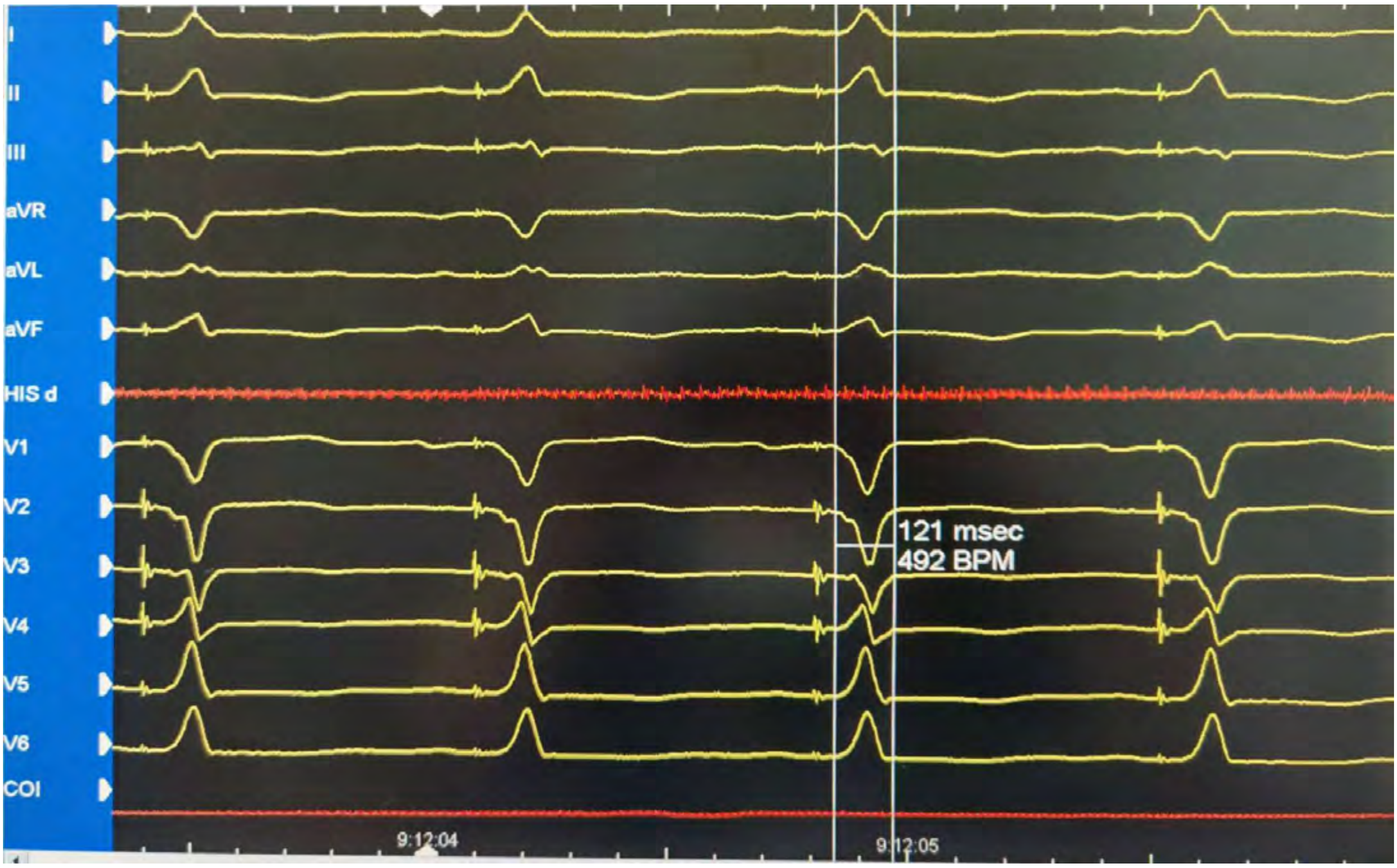

Patient 54:
Transitions

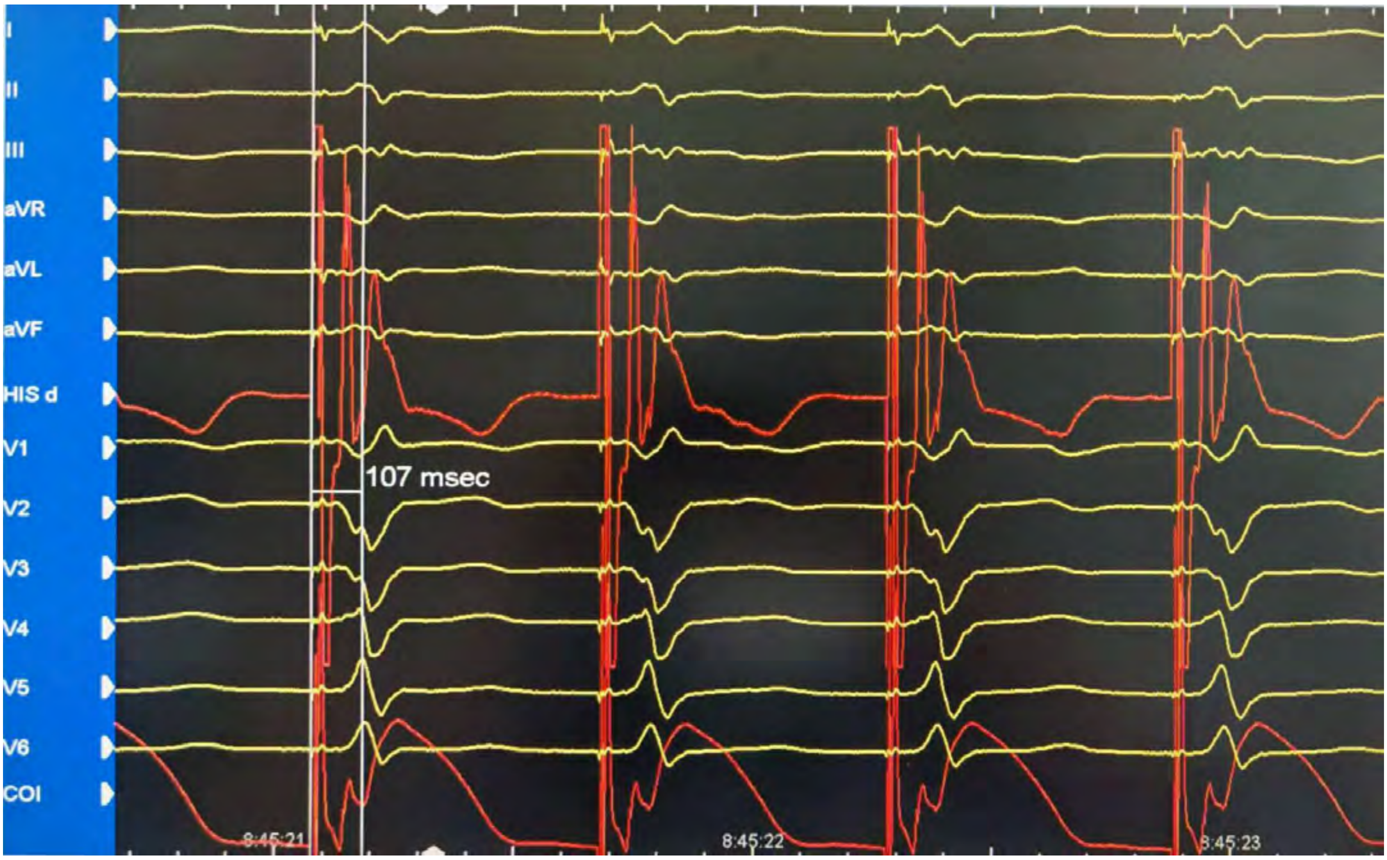

Transitions

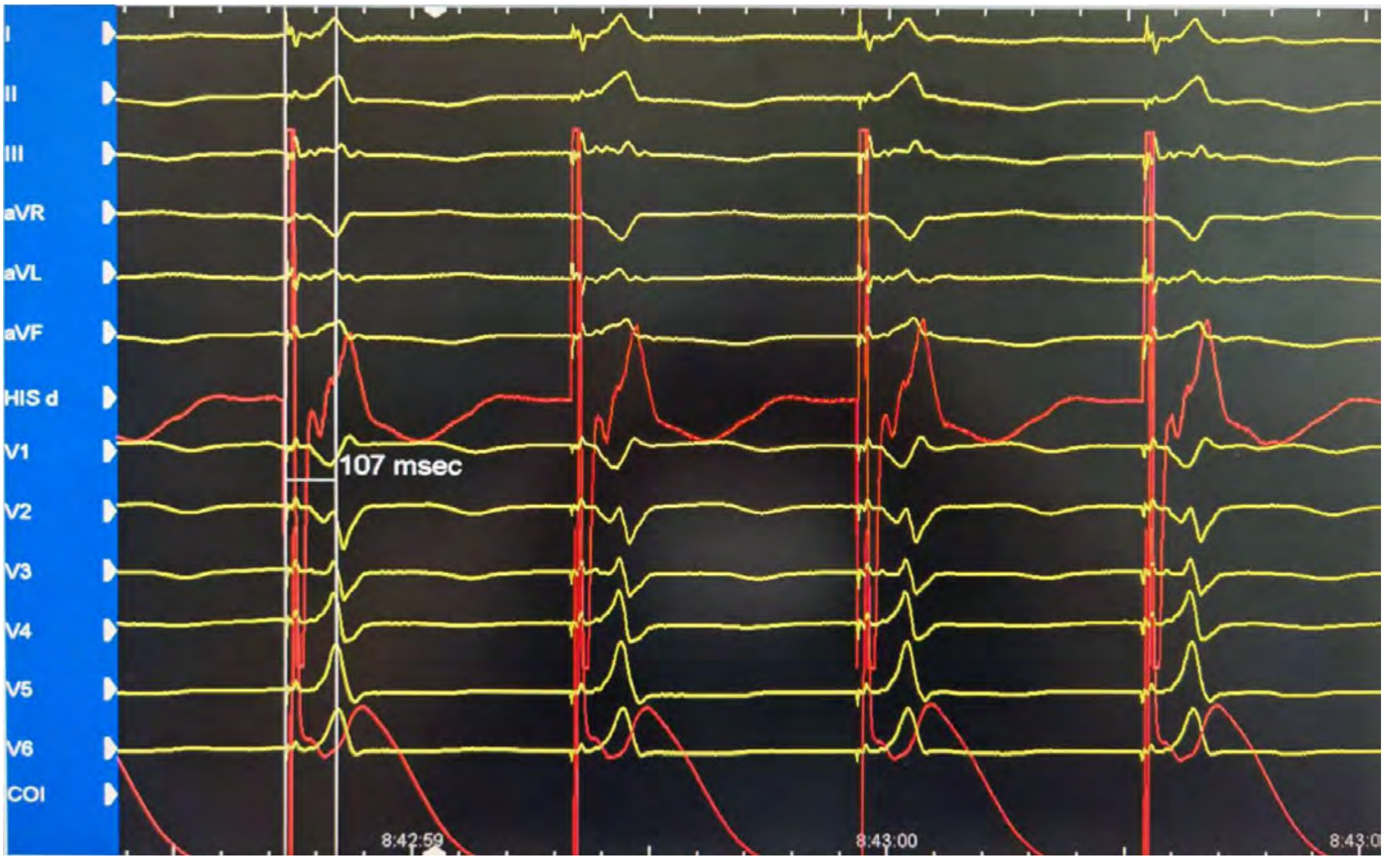

Patient 55:
Pre-ECG

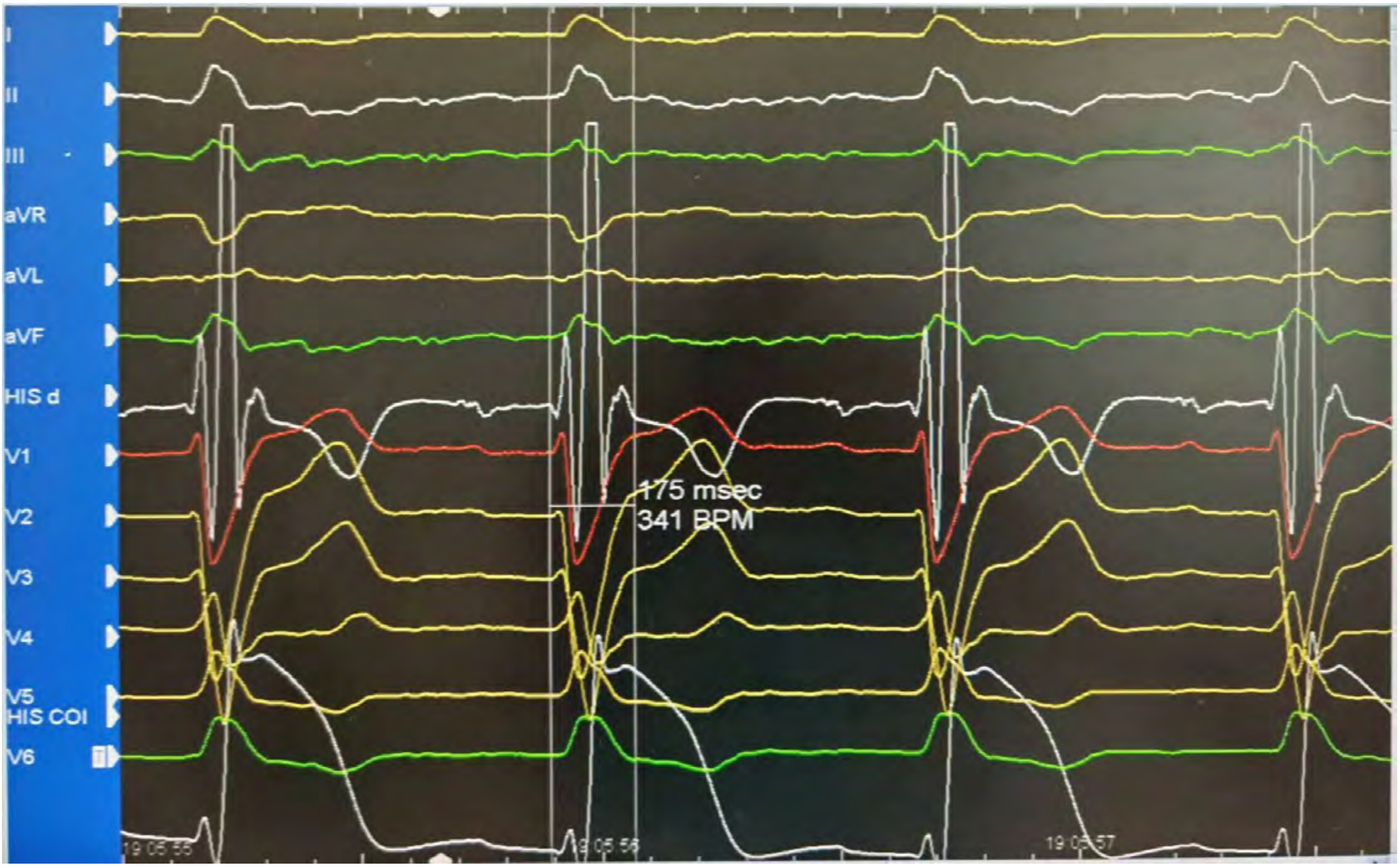

Post ECG

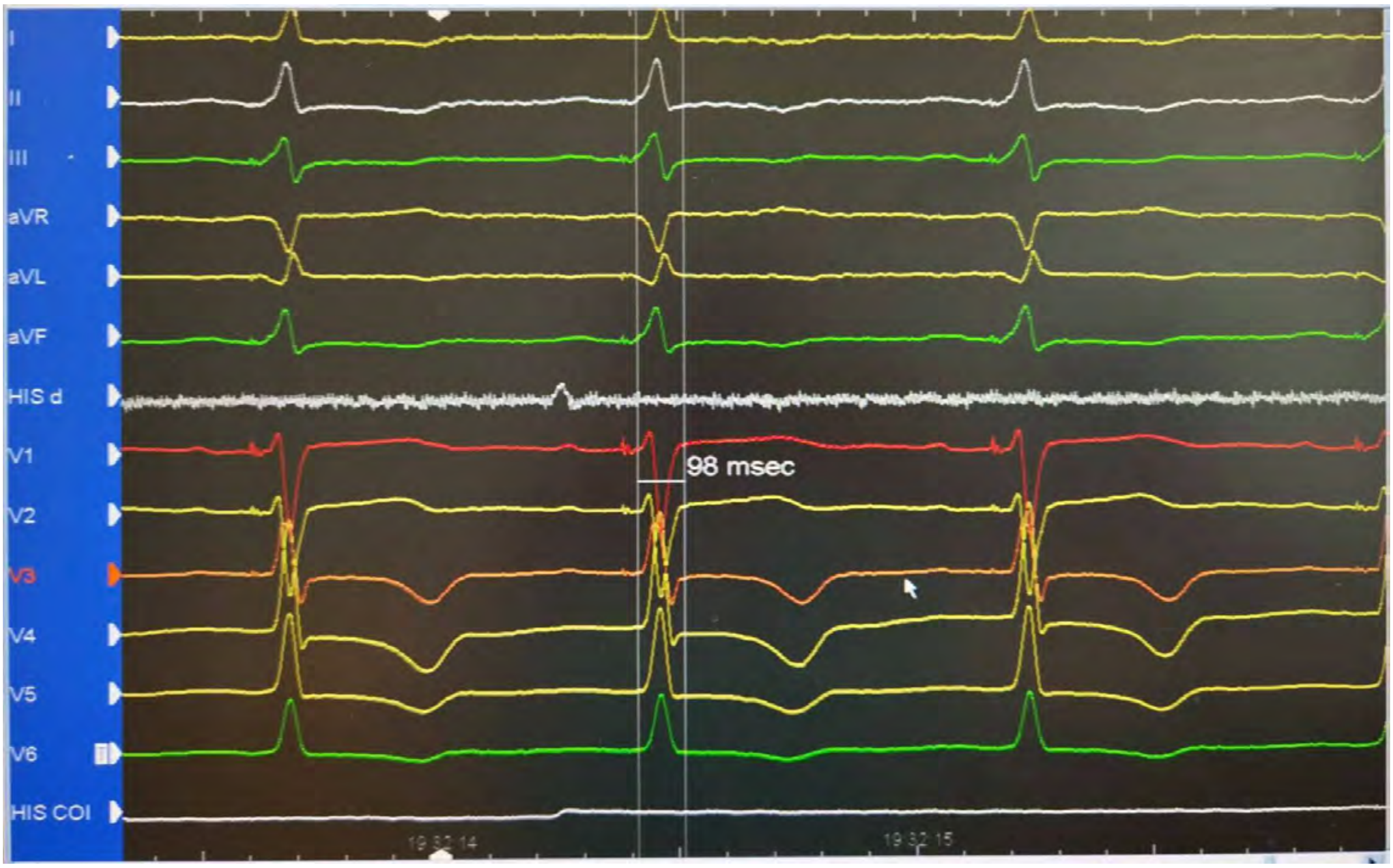

Patient 55:
Transitions

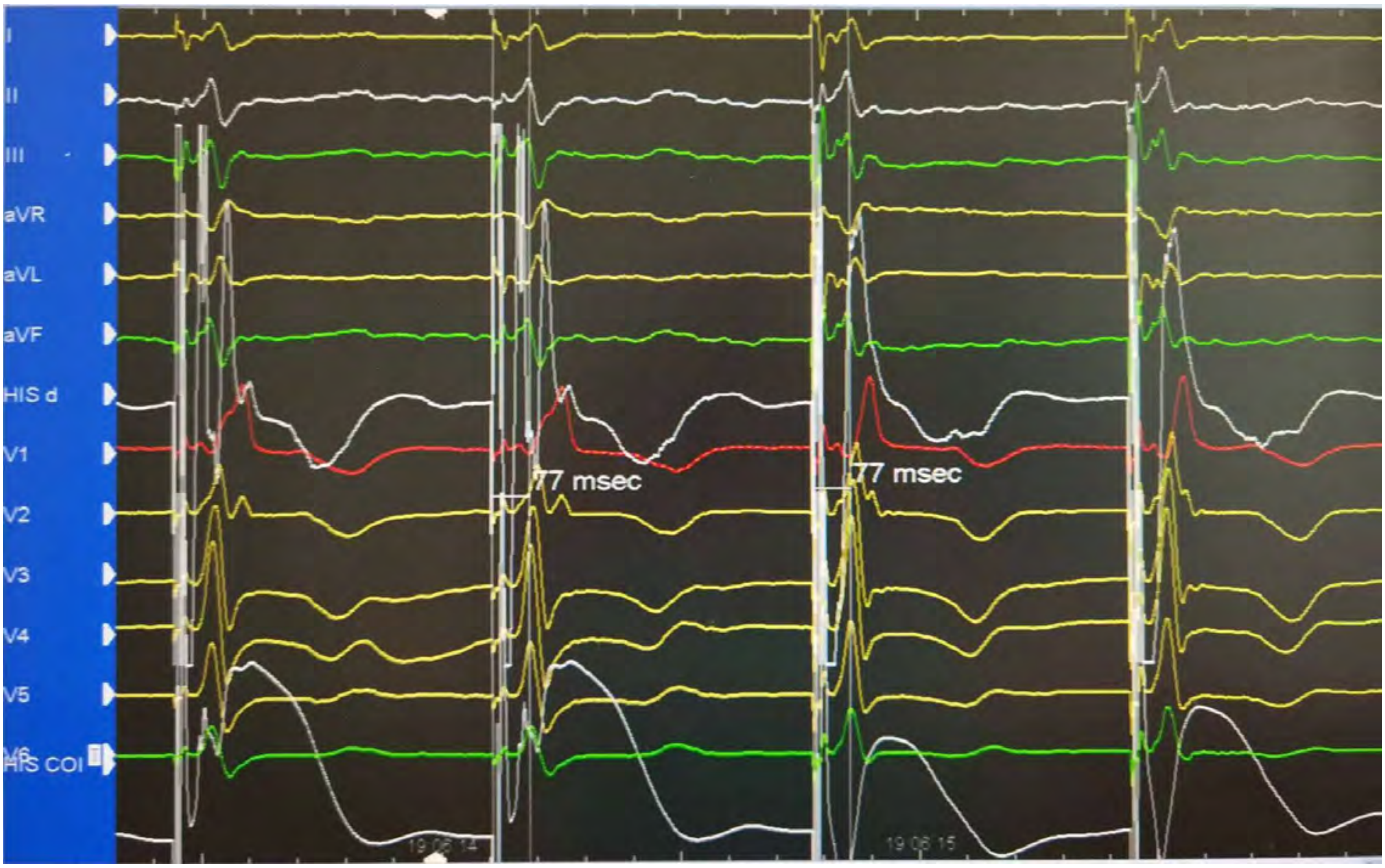

Transitions

Patient 56:
Pre-ECG

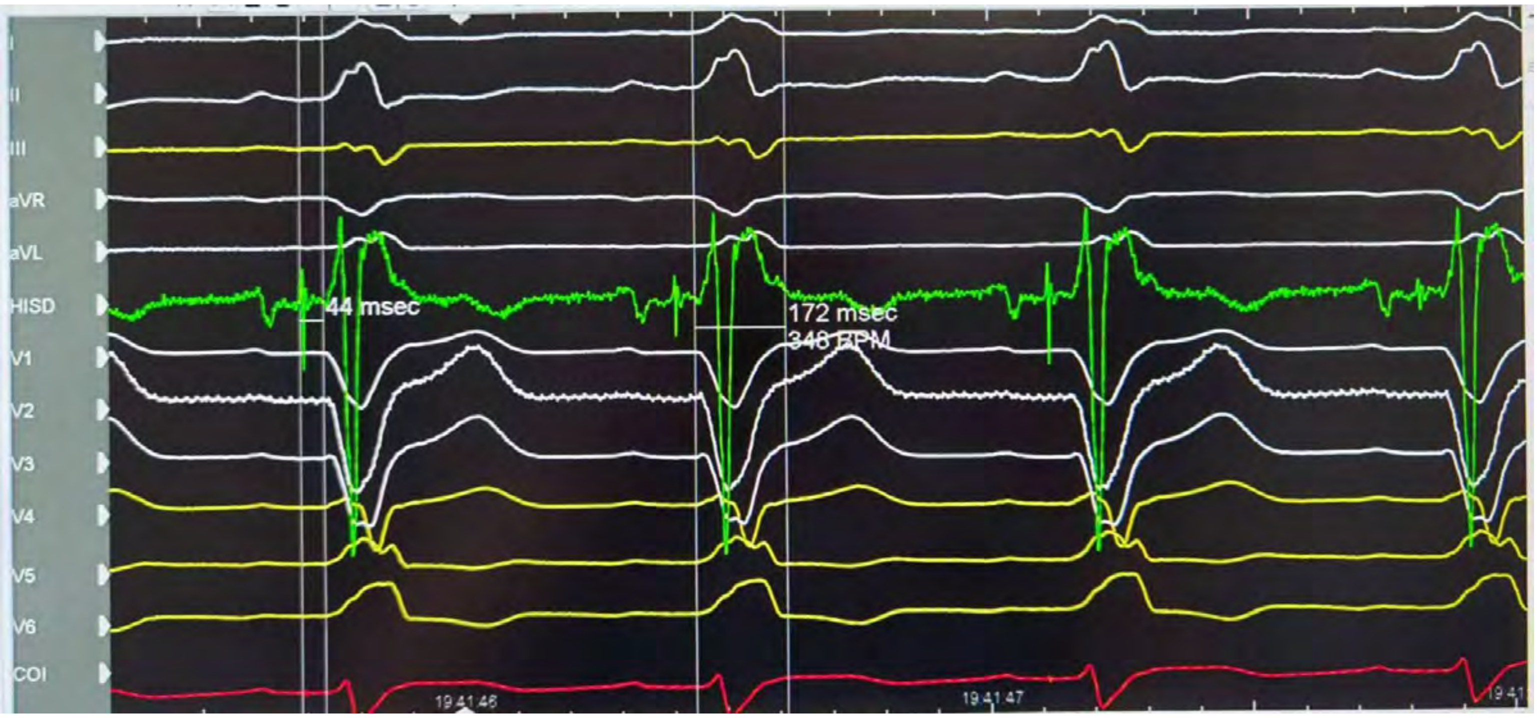

Post ECG

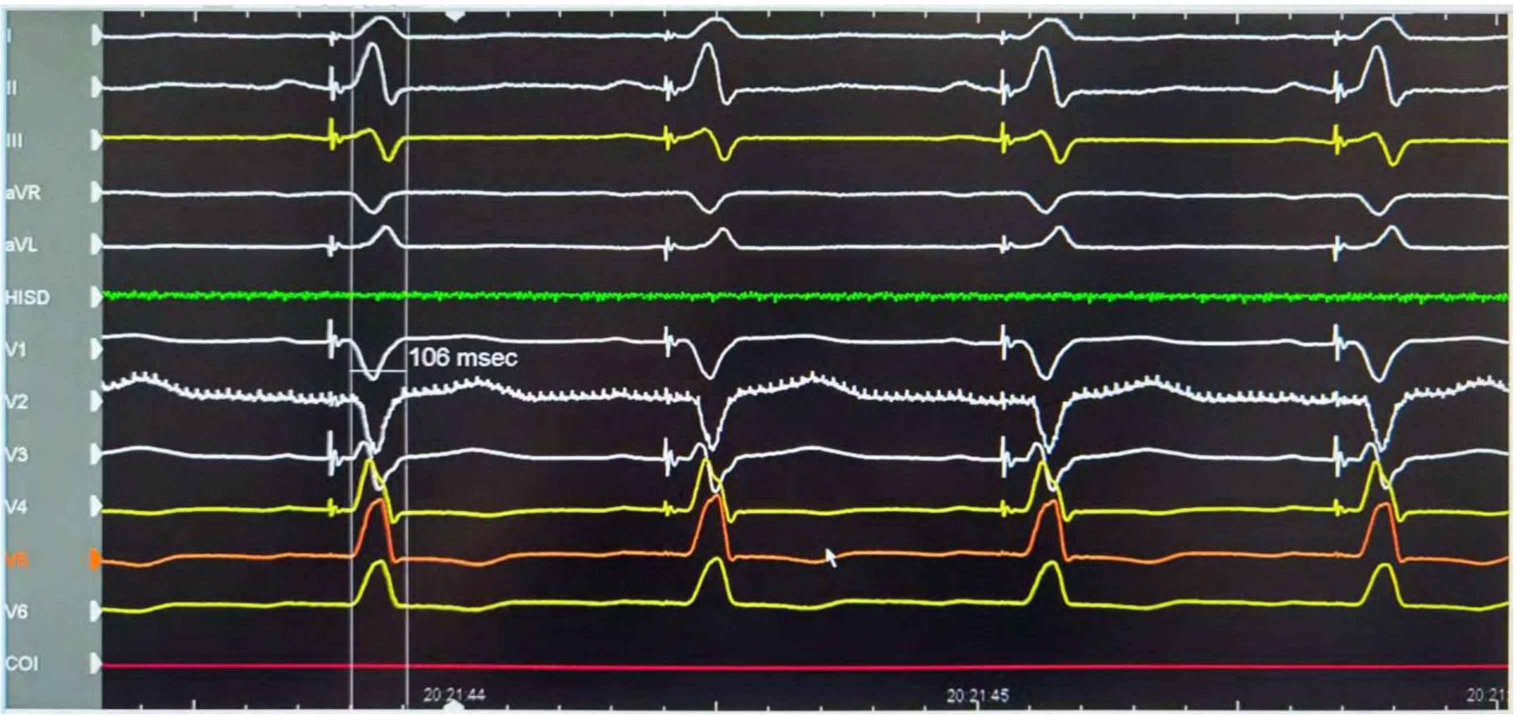

Patient 56:
Transitions

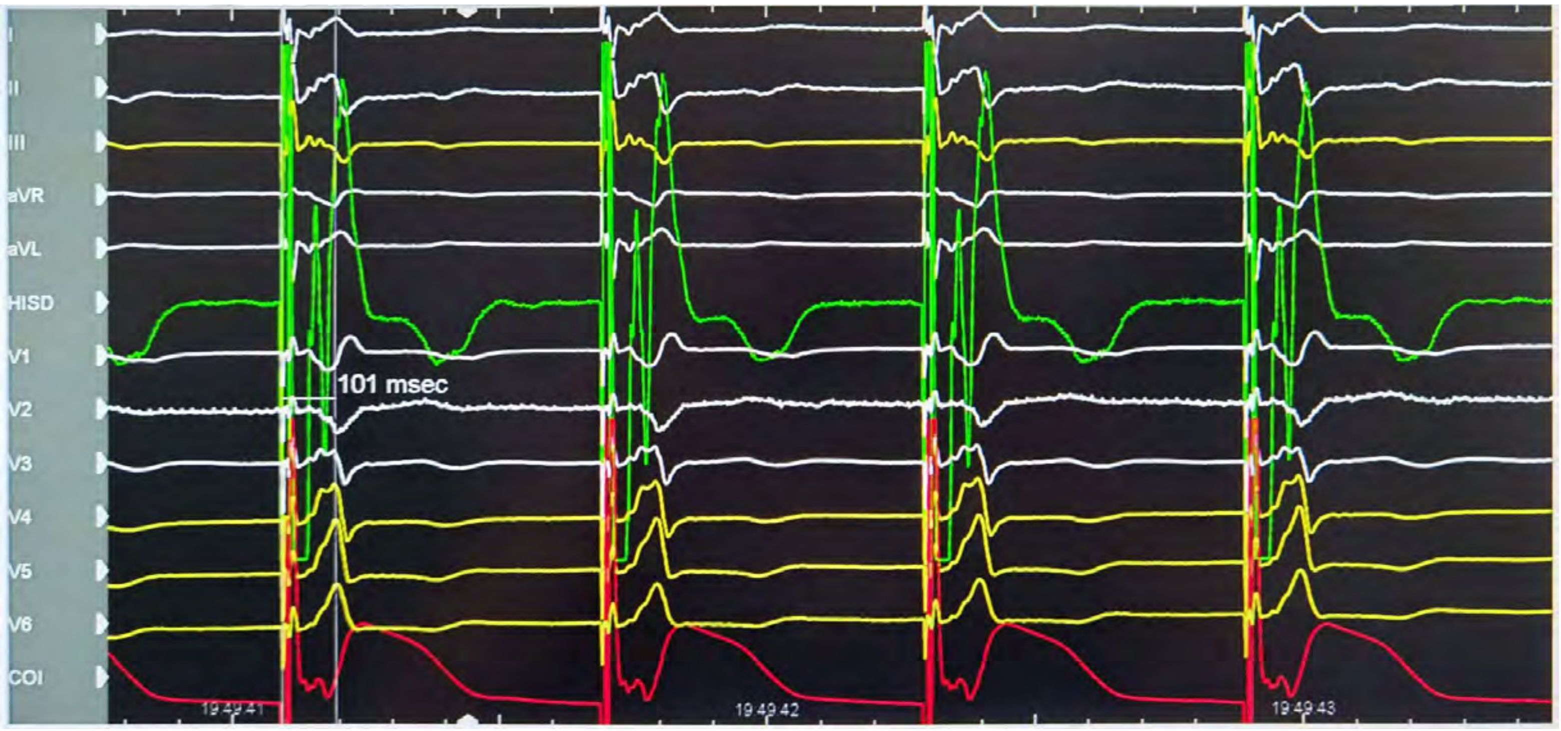

Transitions

Patient 57:
Pre-ECG

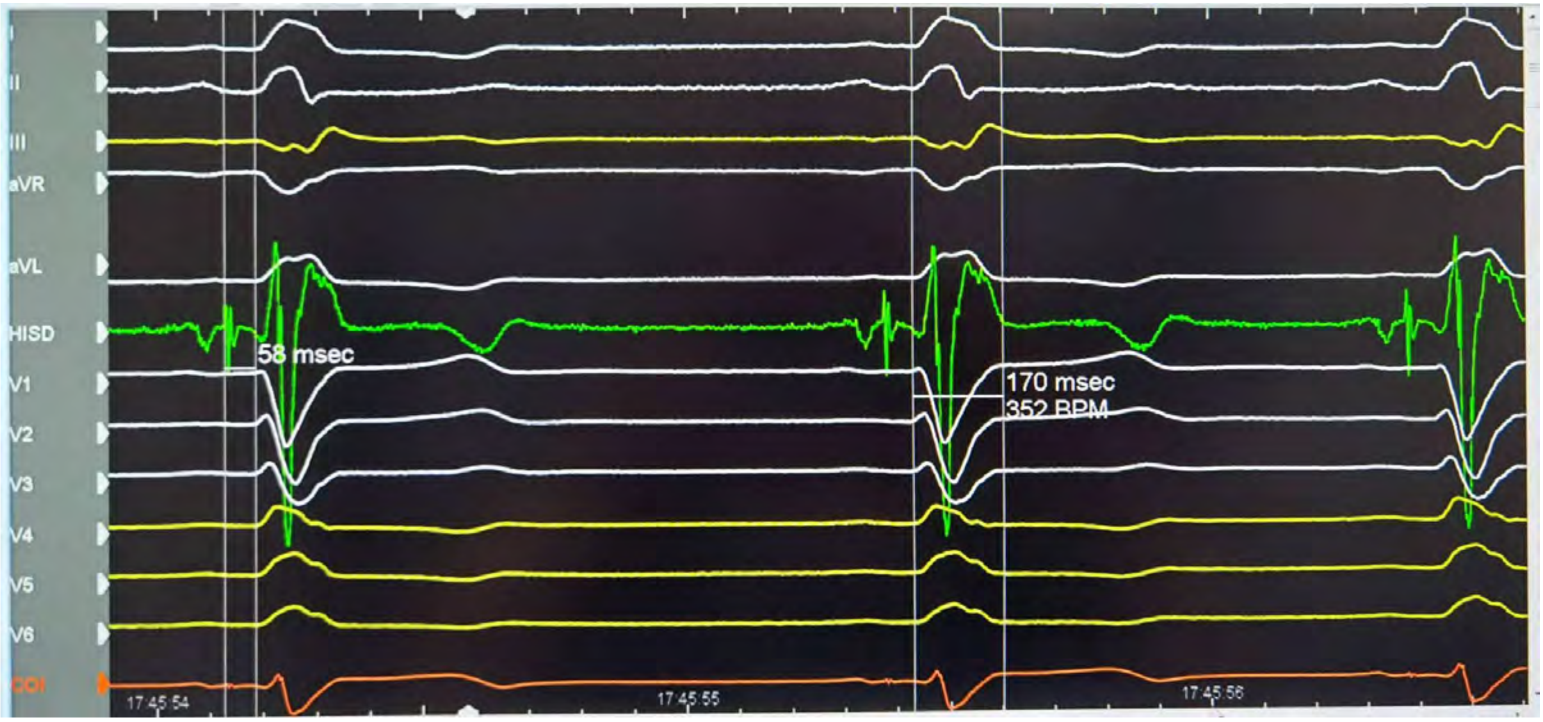

Post ECG

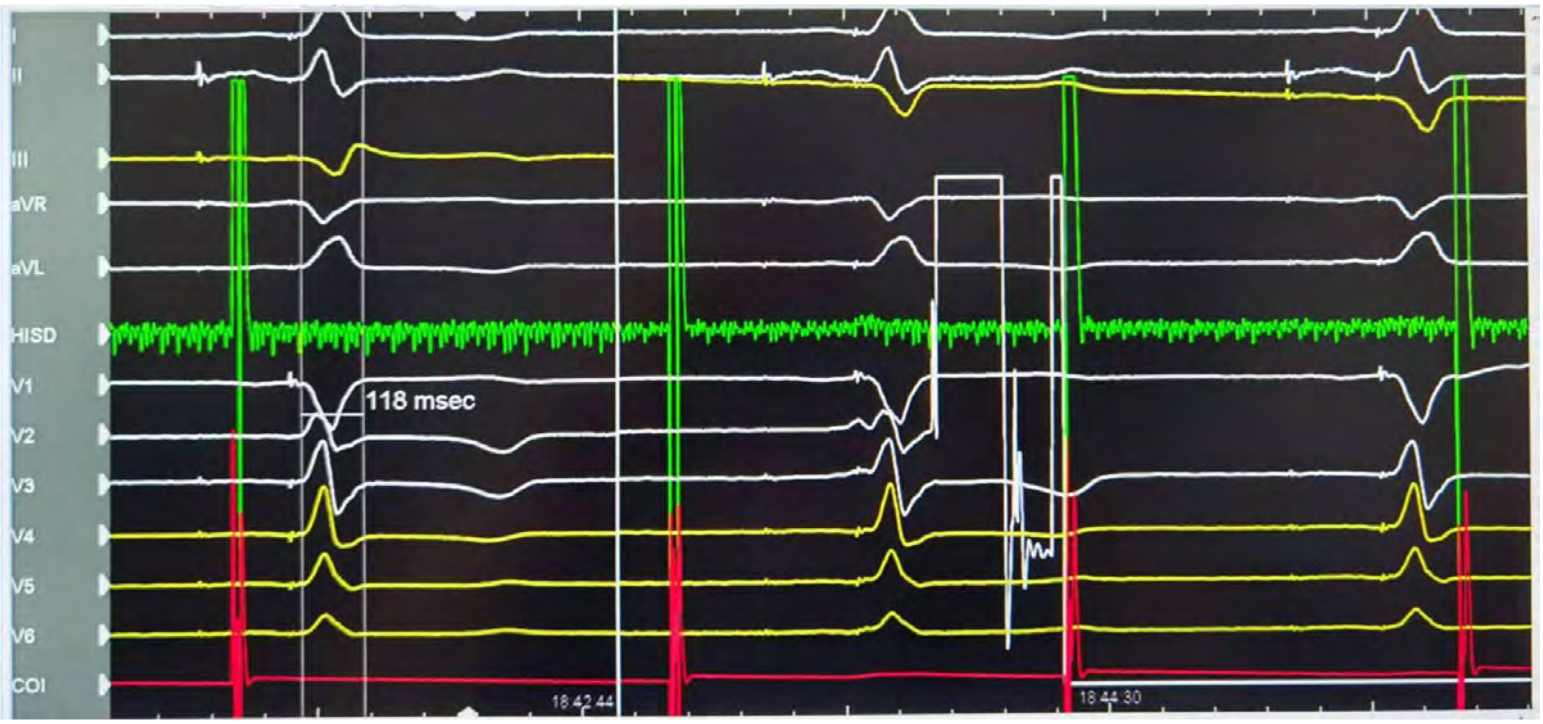

Patient 57:
Transitions

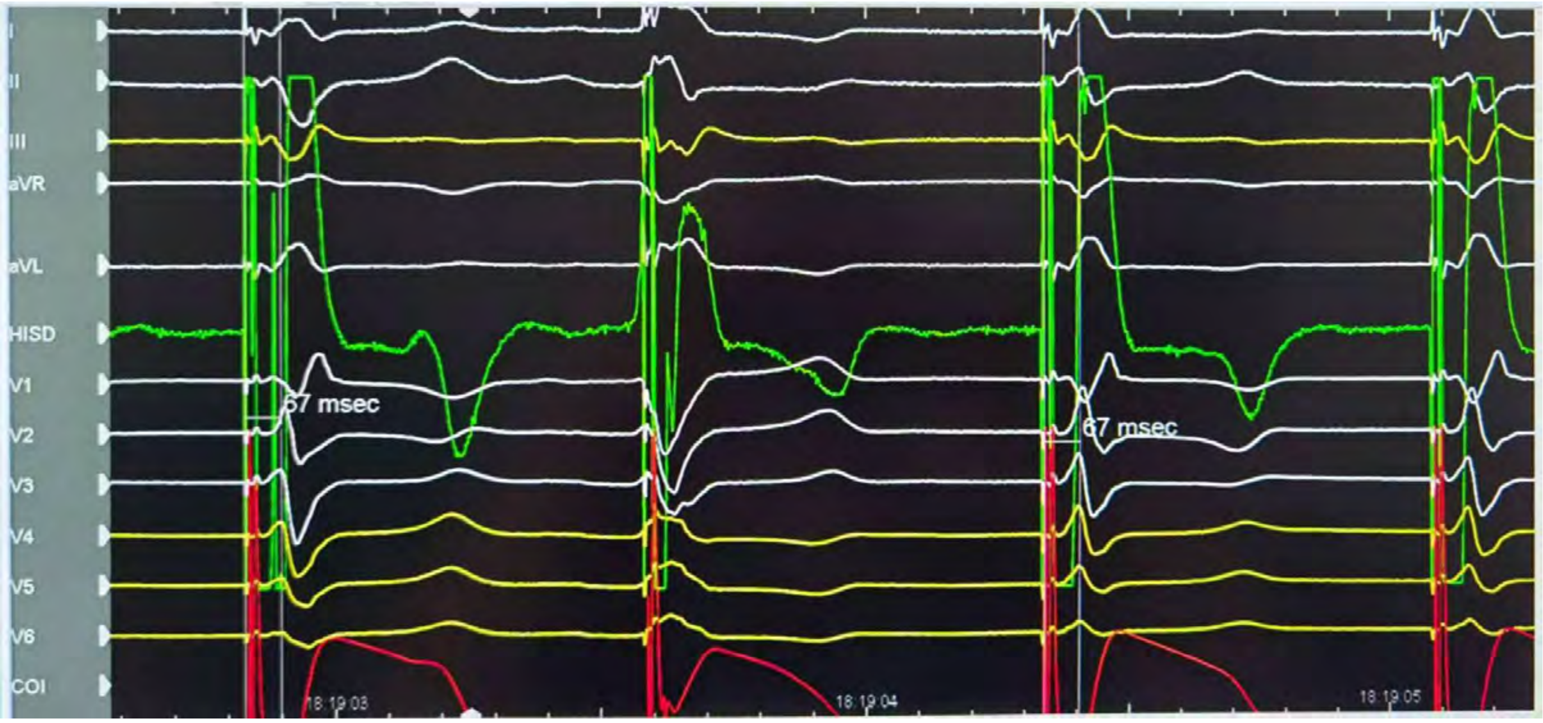

Transitions

Patient 58:
Pre-ECG

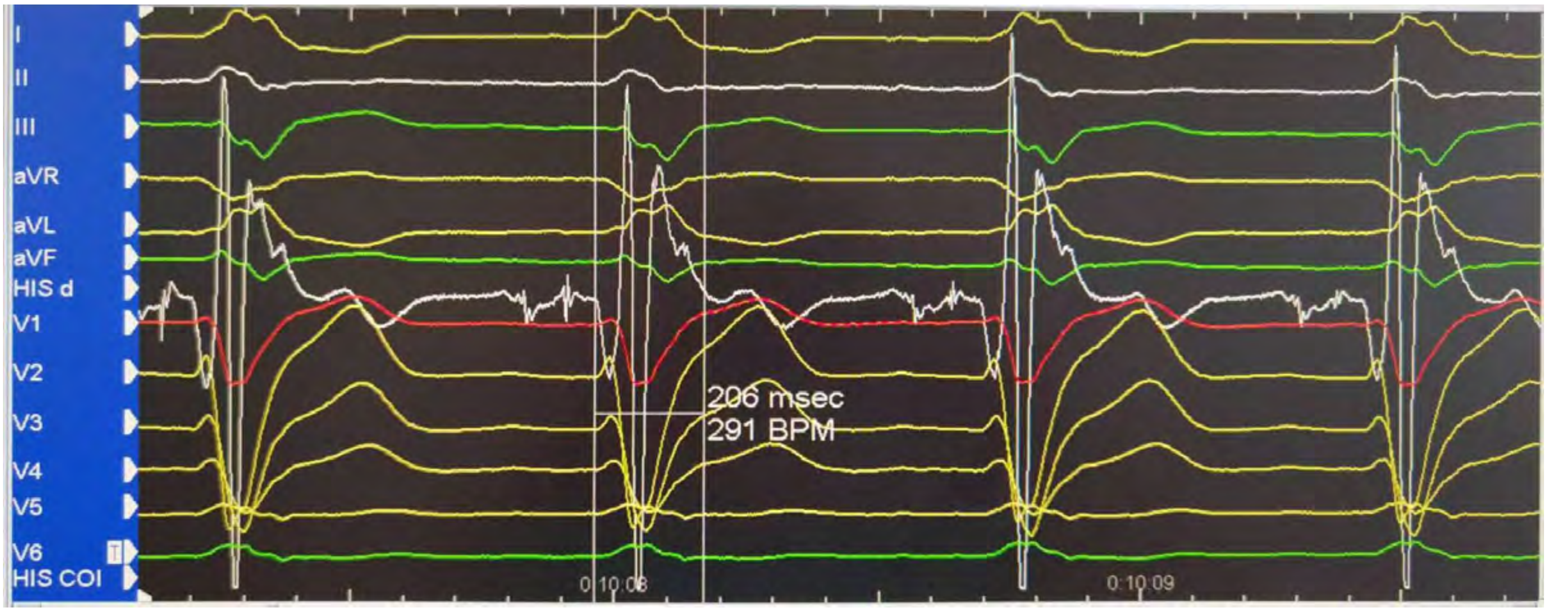

Post ECG

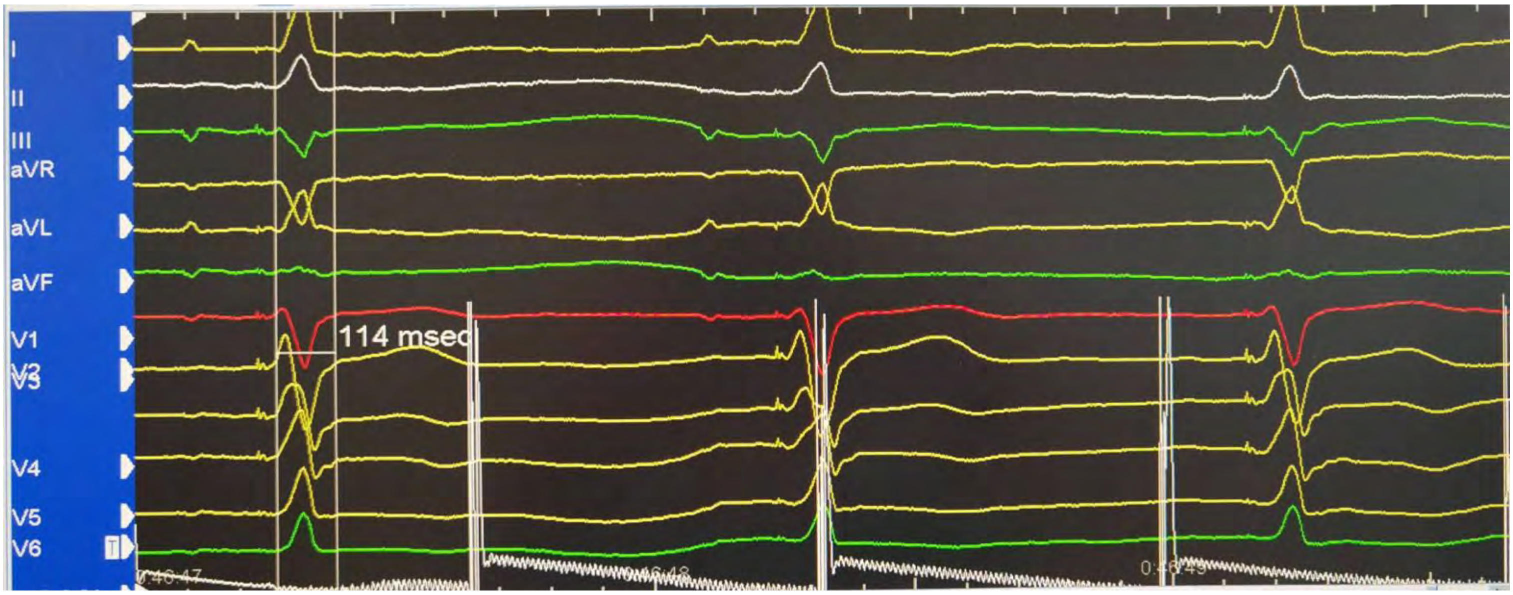

Patient 58:
Transitions

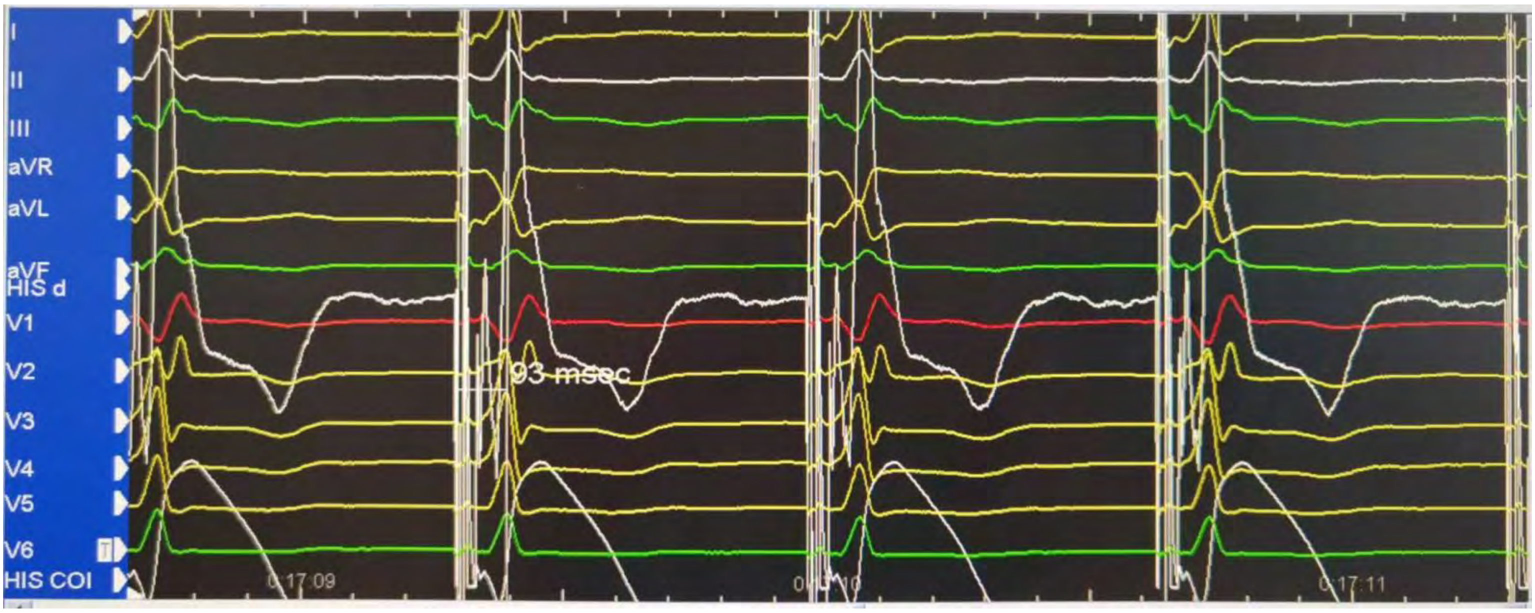

Transitions

Patient 59:
Pre-ECG

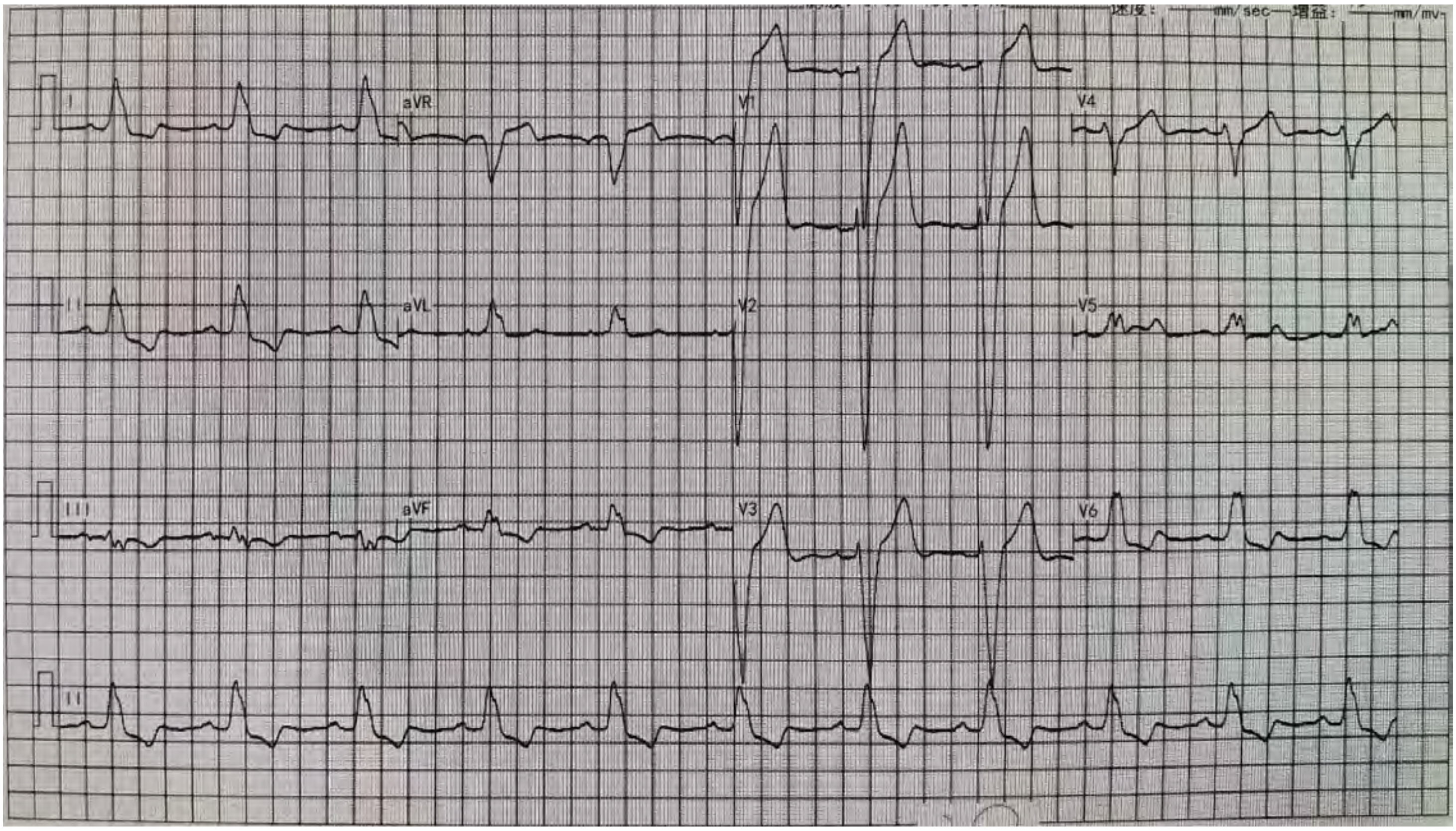

Post ECG

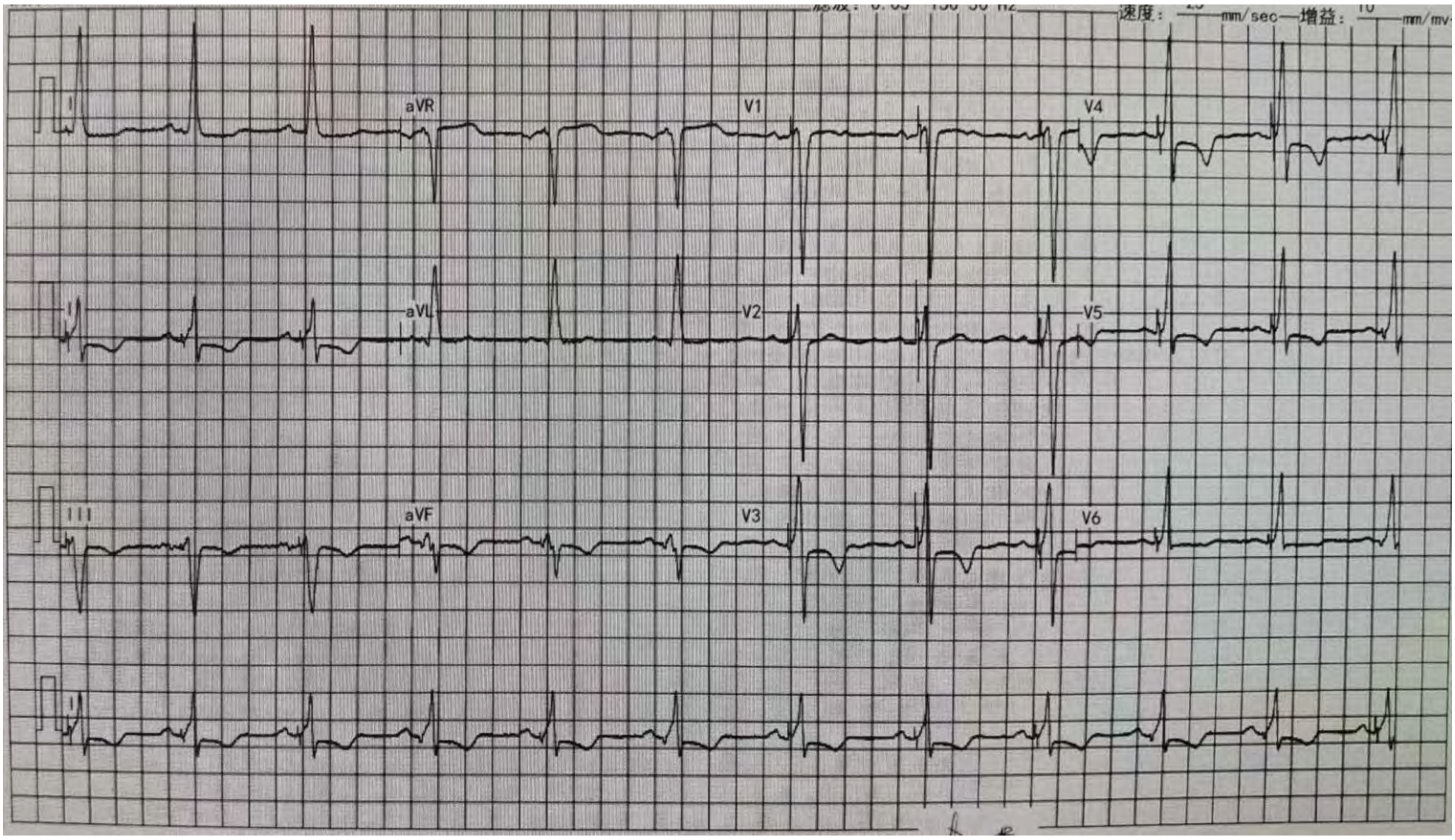

Patient 60:

Pre-ECG

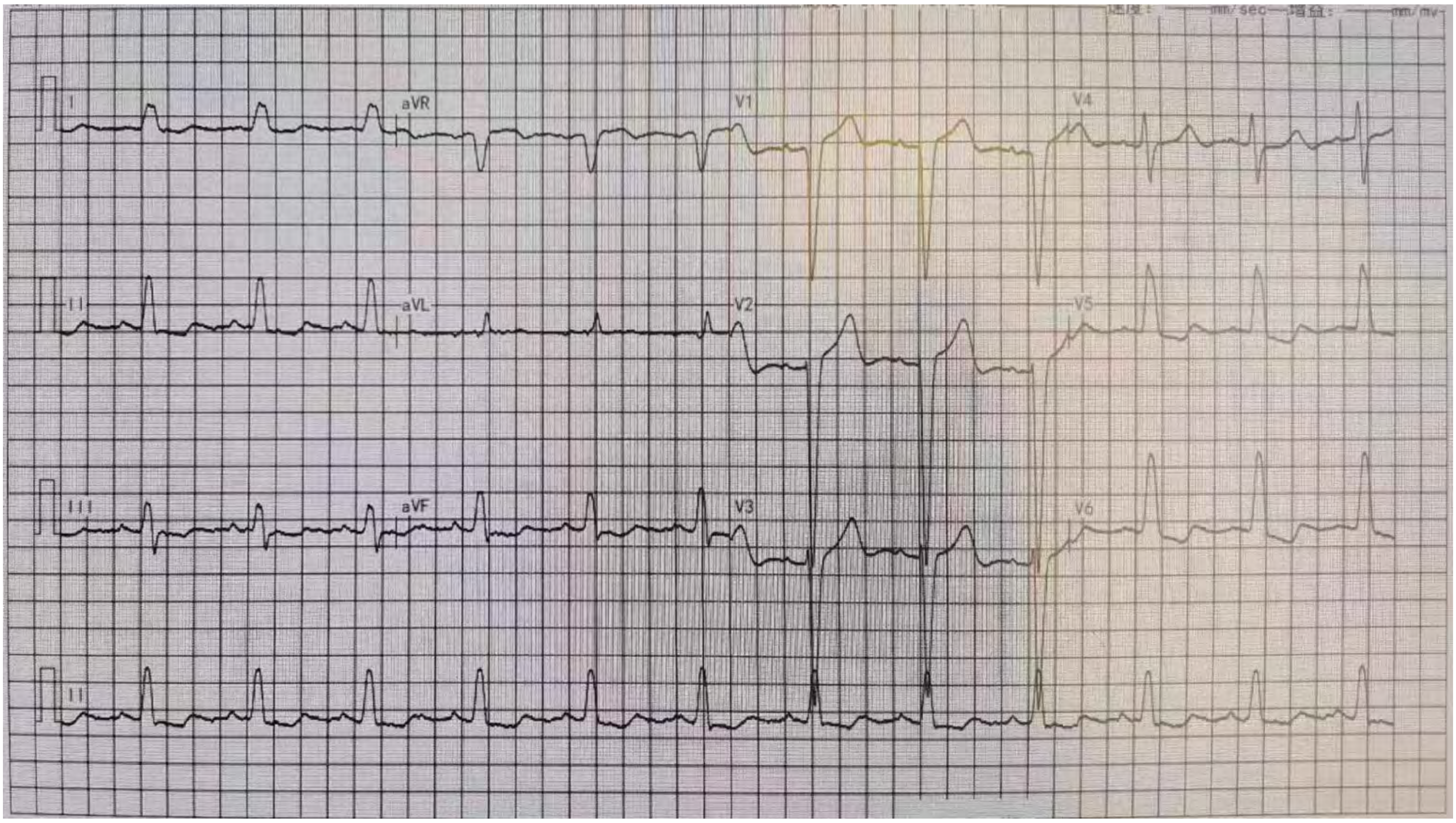

Post ECG

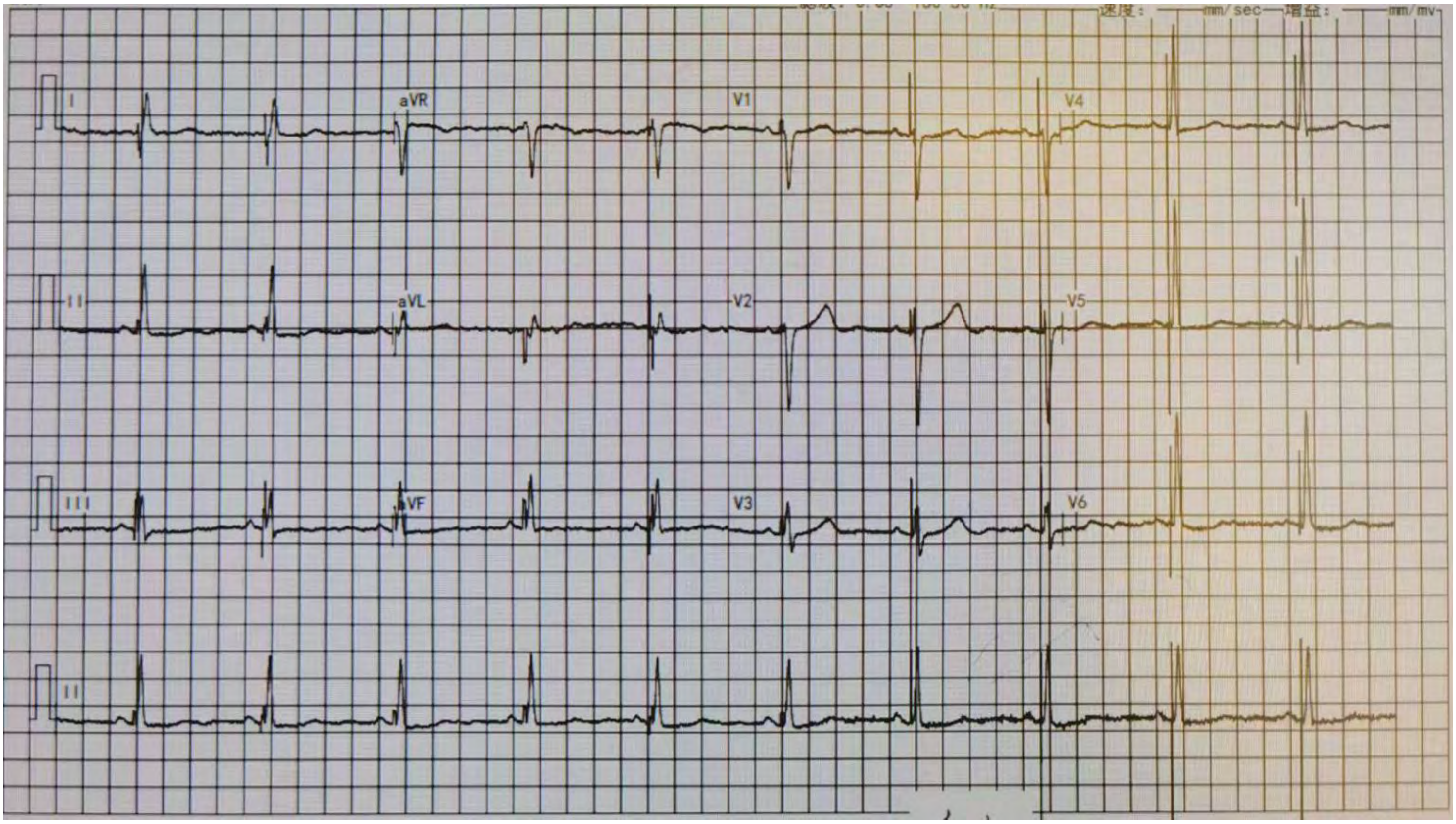

Patient 61:
Pre-ECG

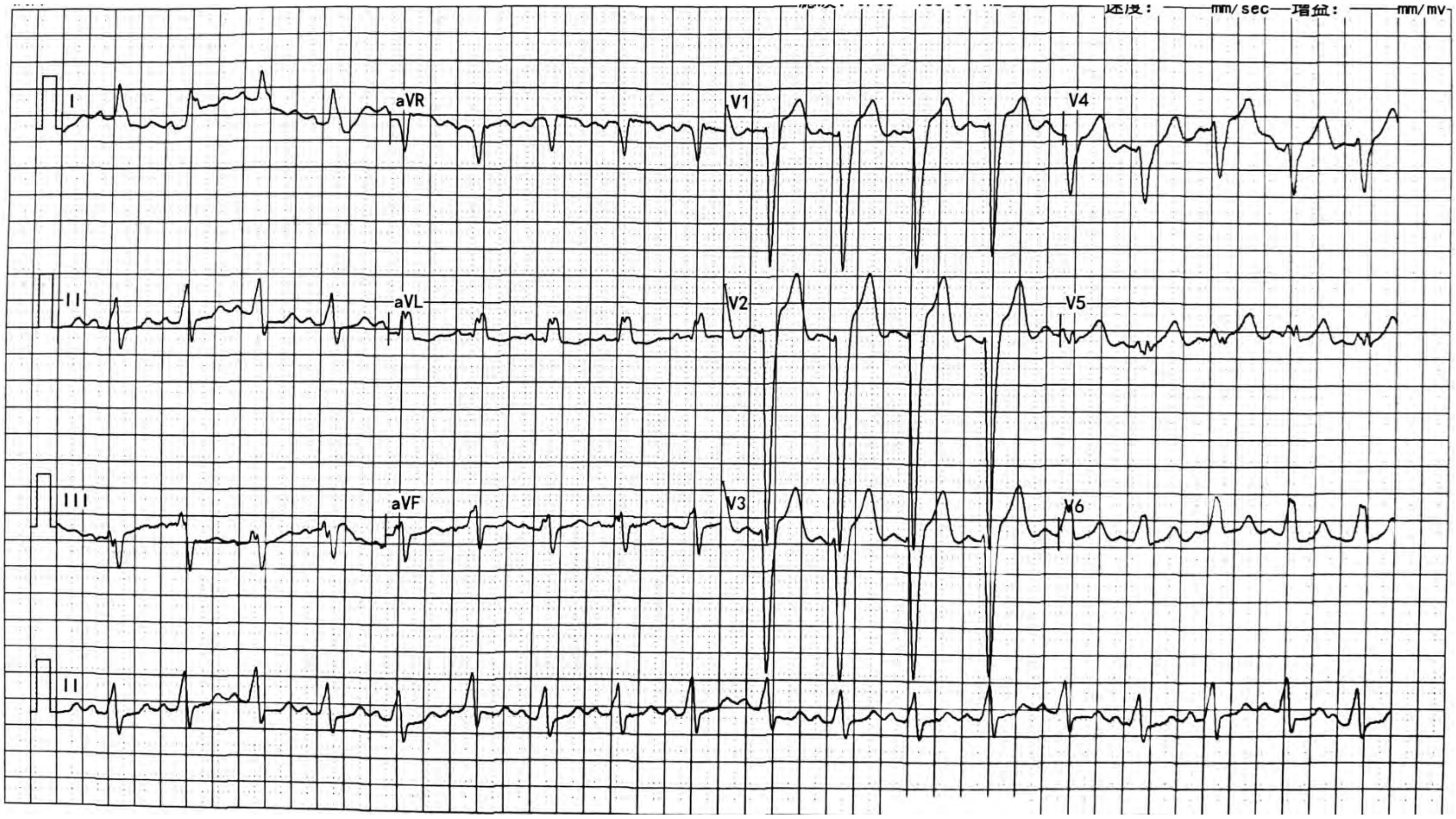

Post ECG

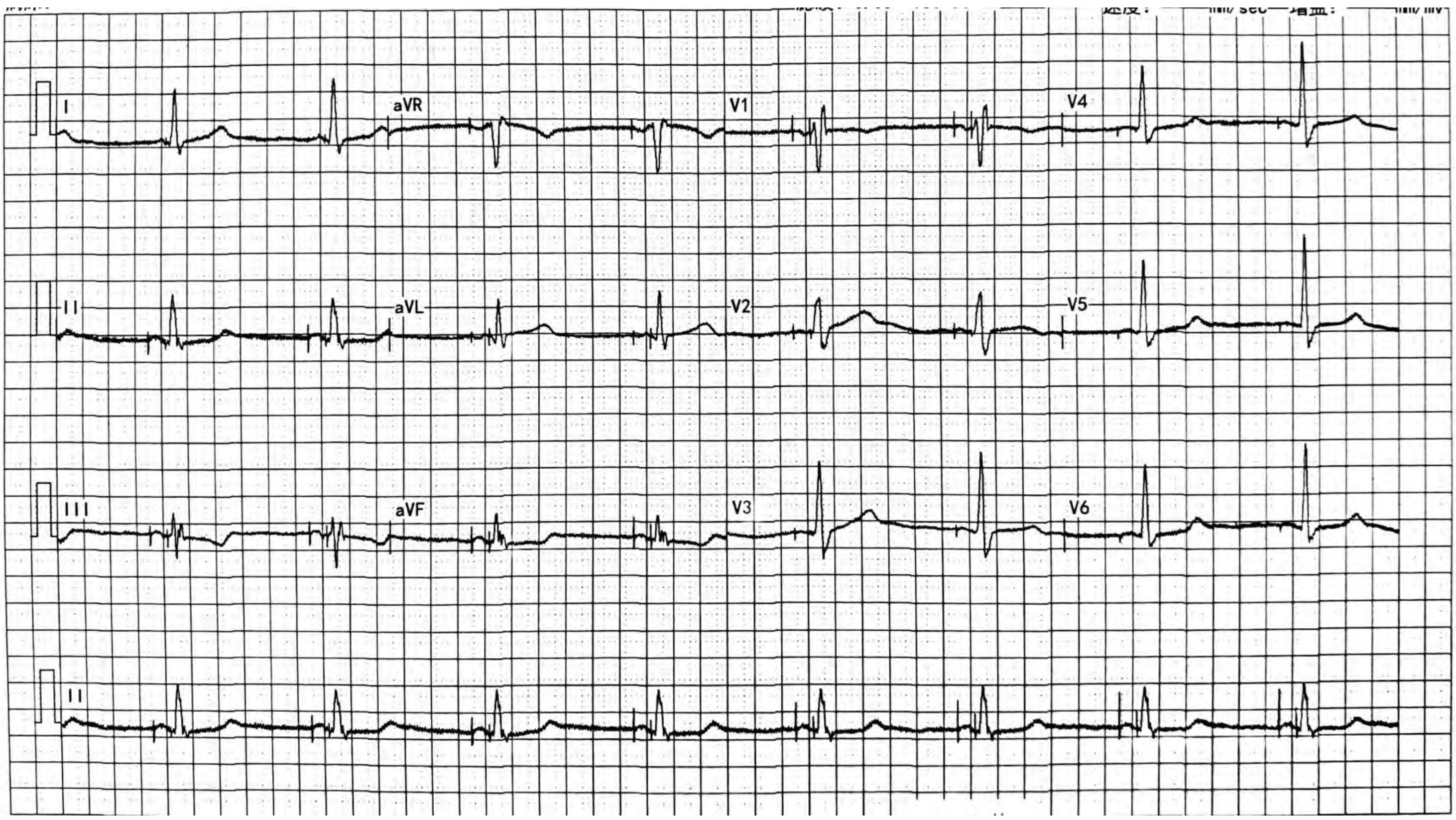

Patient 62:
Pre-ECG

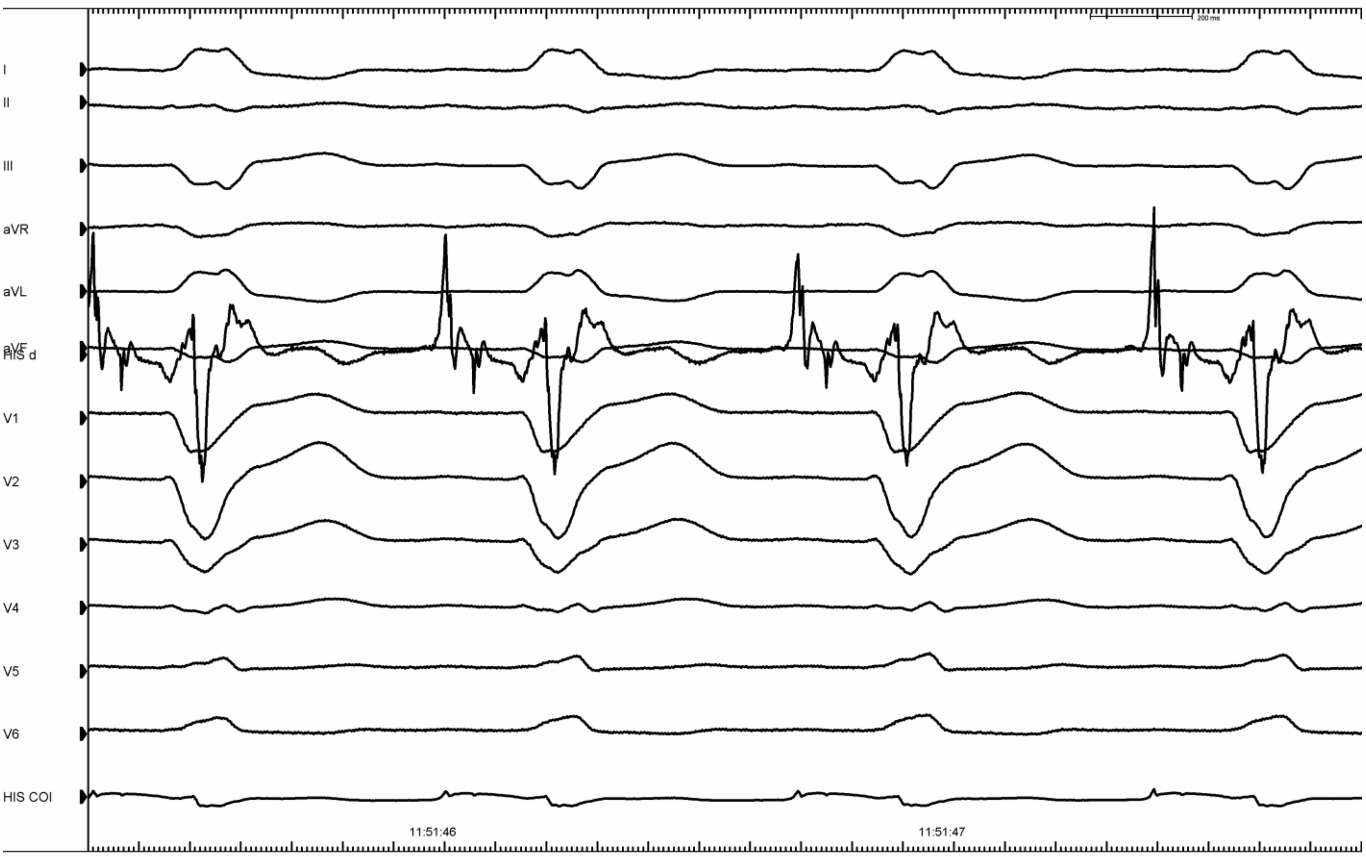

Post ECG

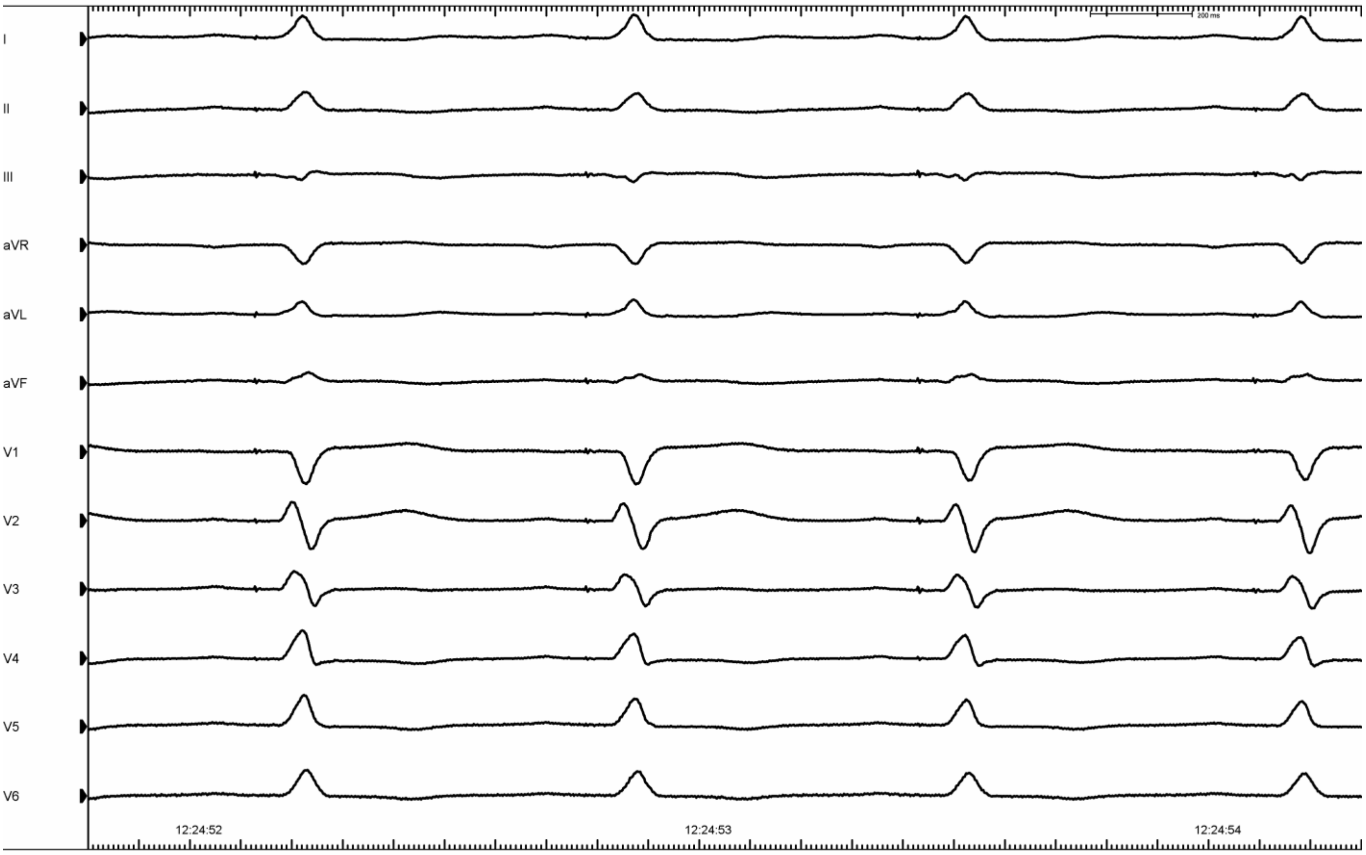

Patient 62:
Transitions

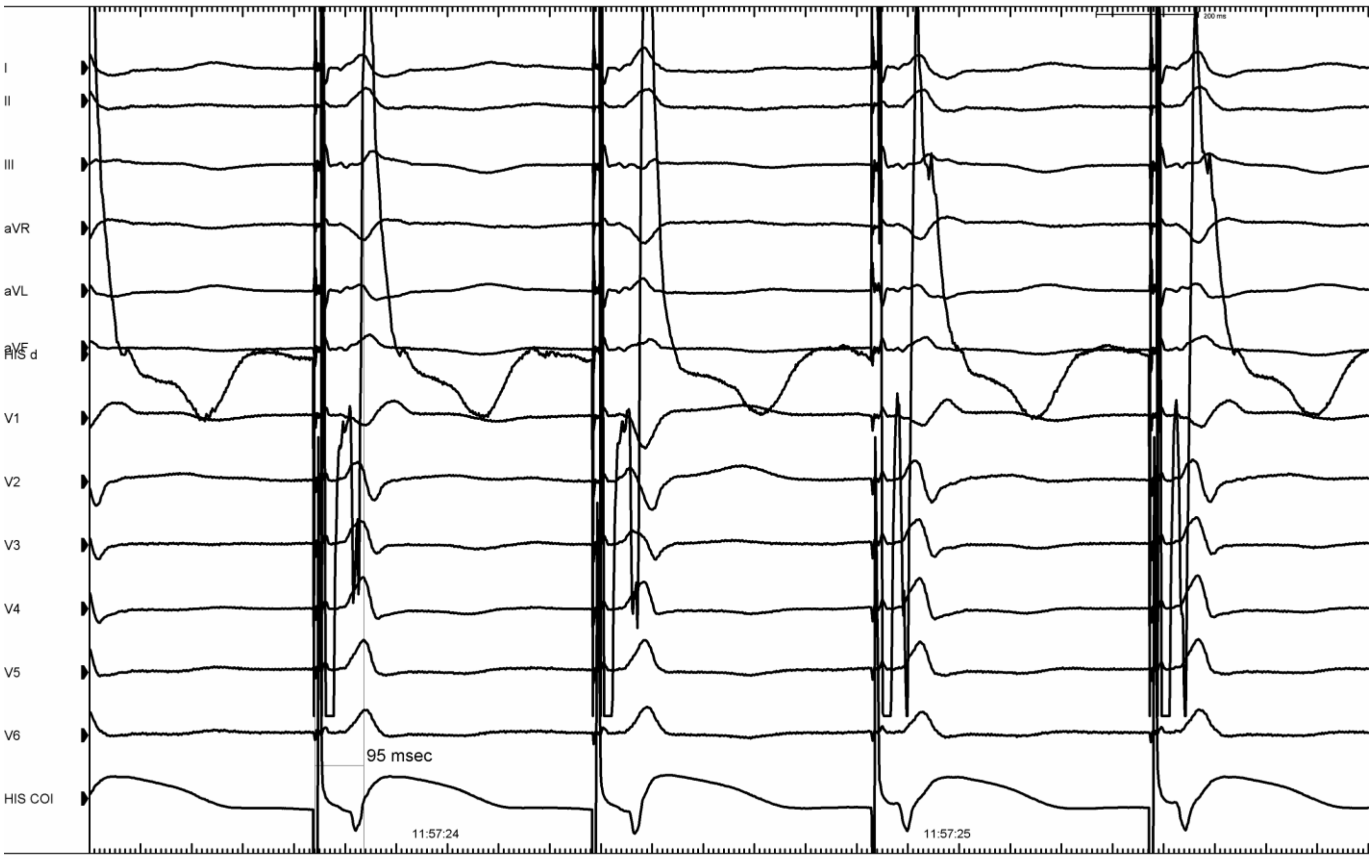

Transitions

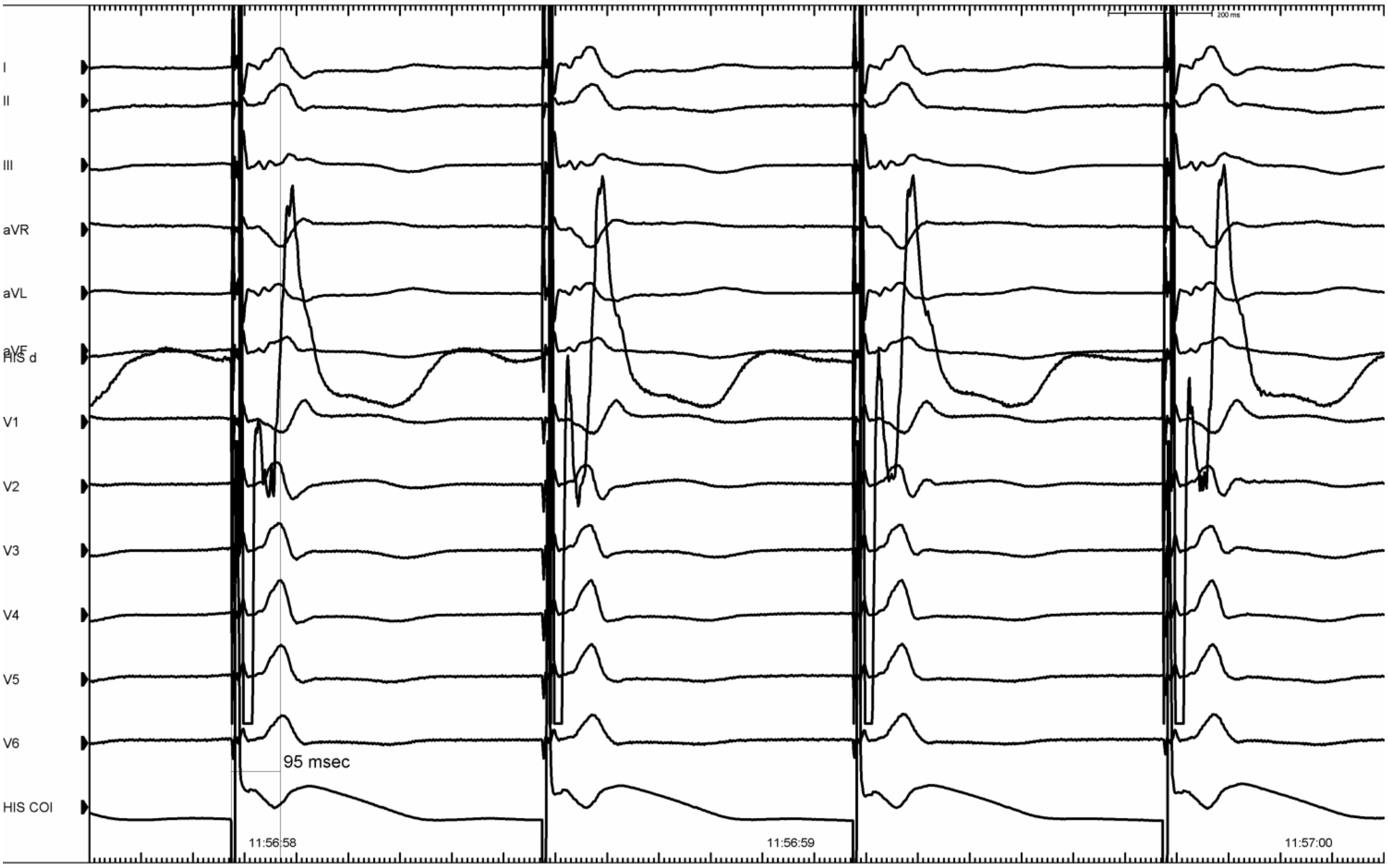

Patient 63:
Pre-ECG

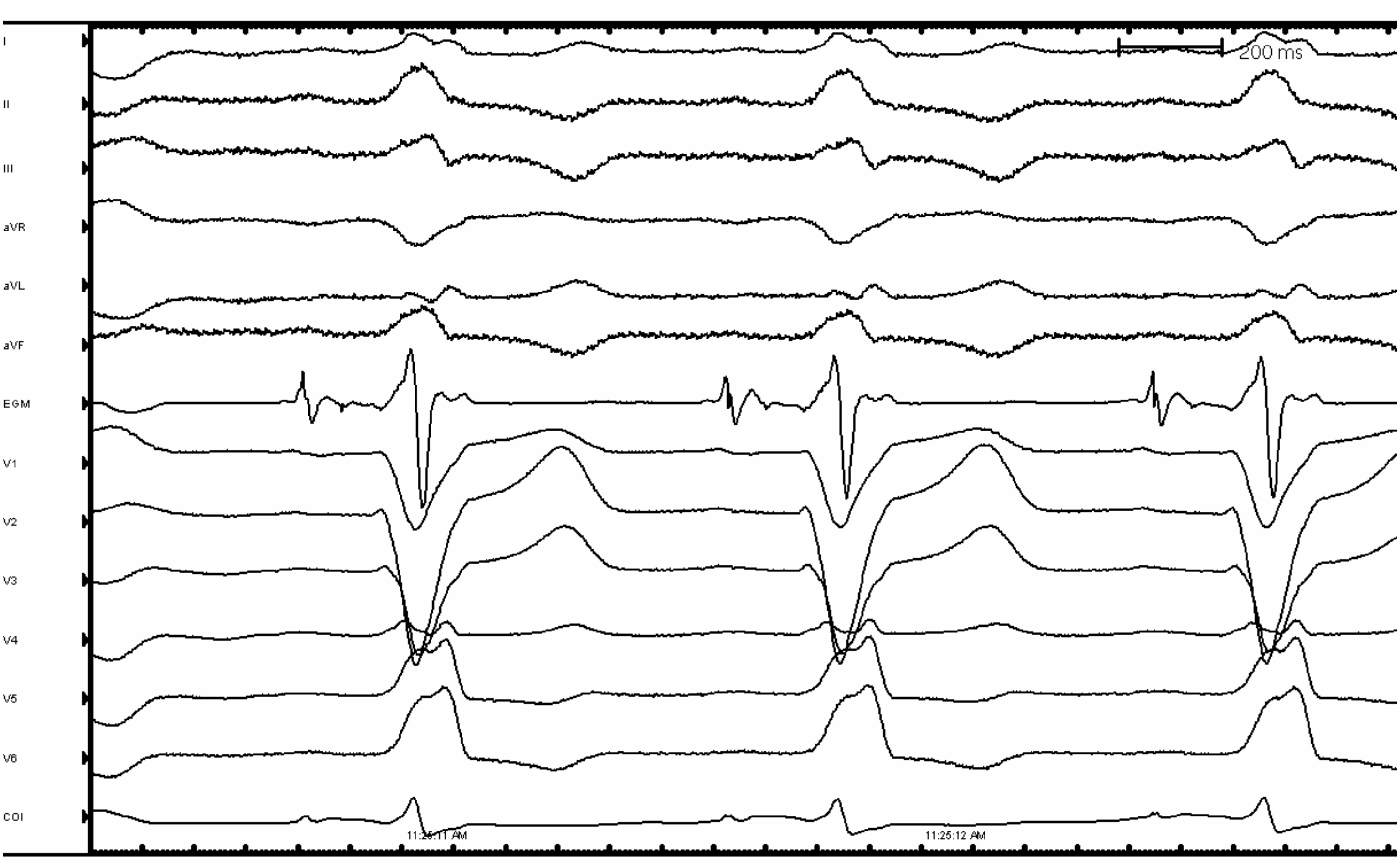

Post ECG

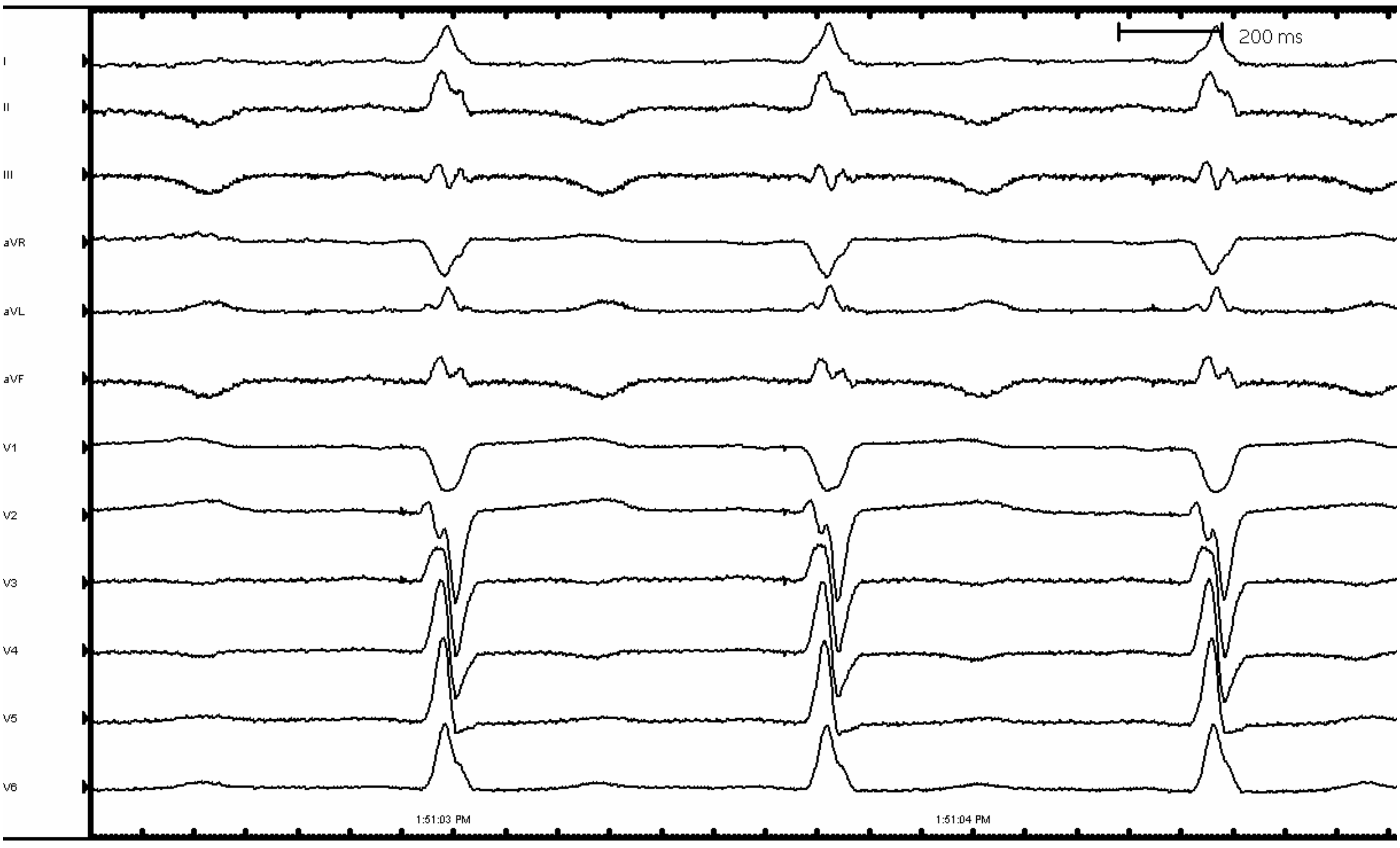

Patient 63:
Transitions

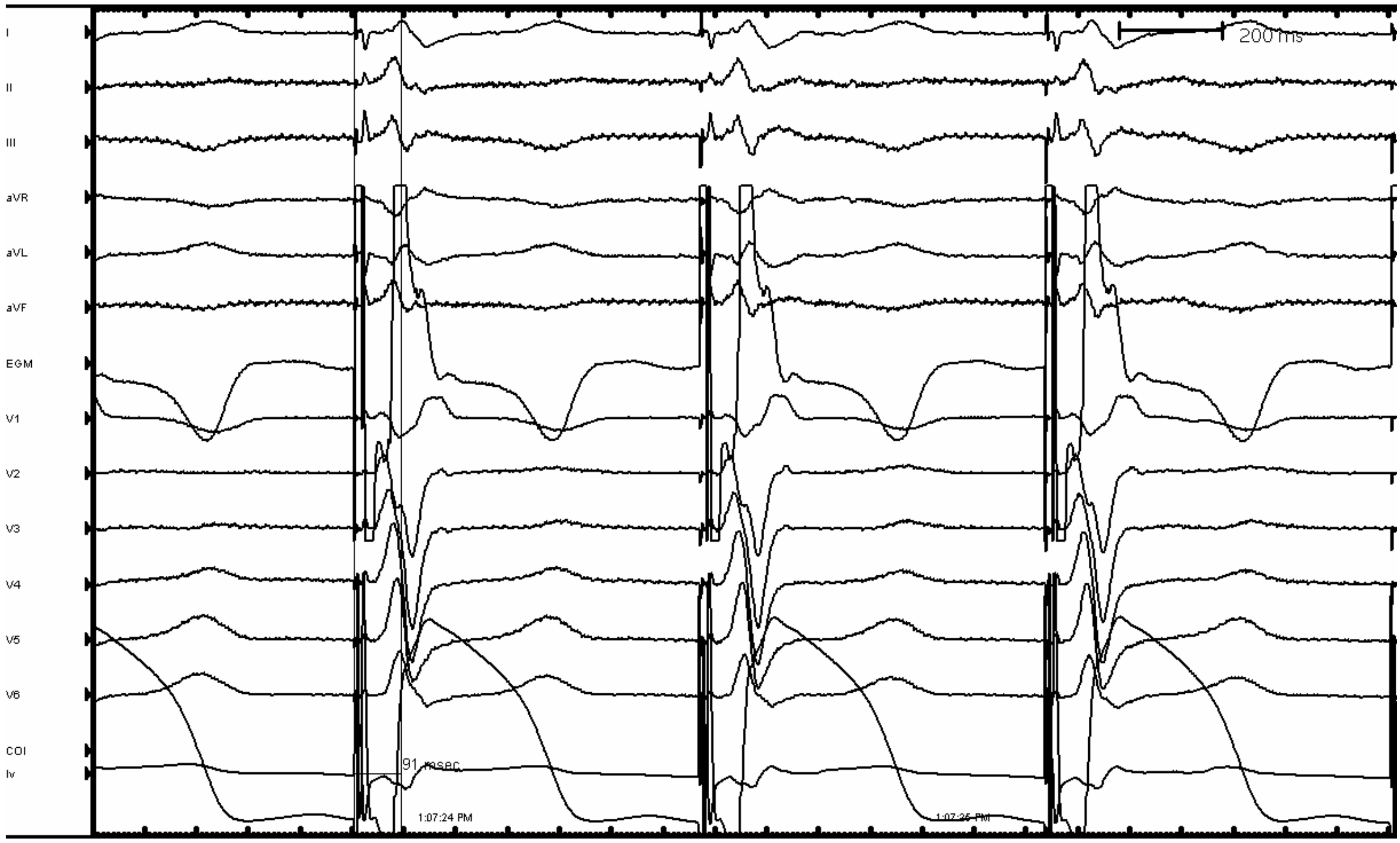

Transitions

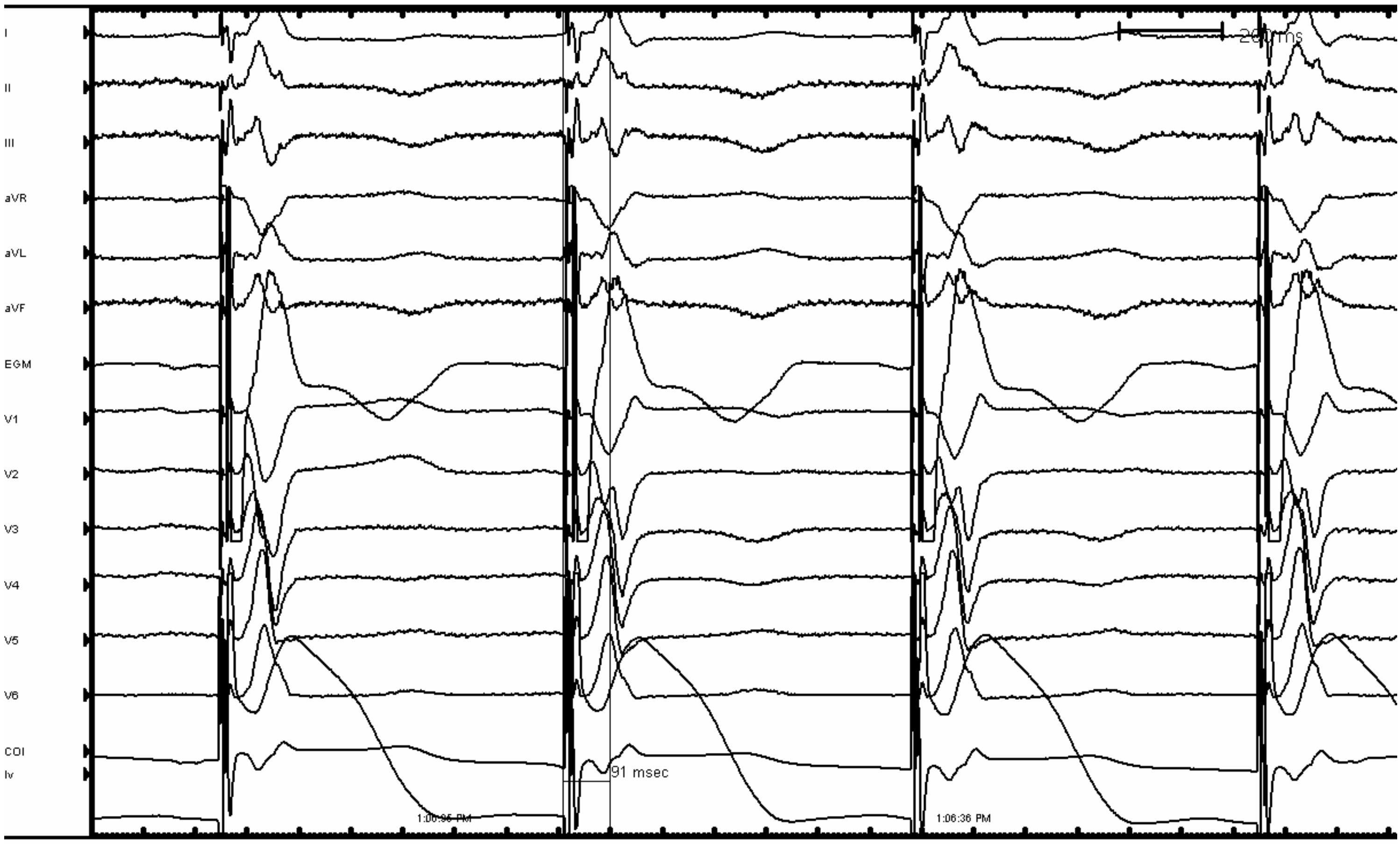

Patient 64:
Pre-ECG

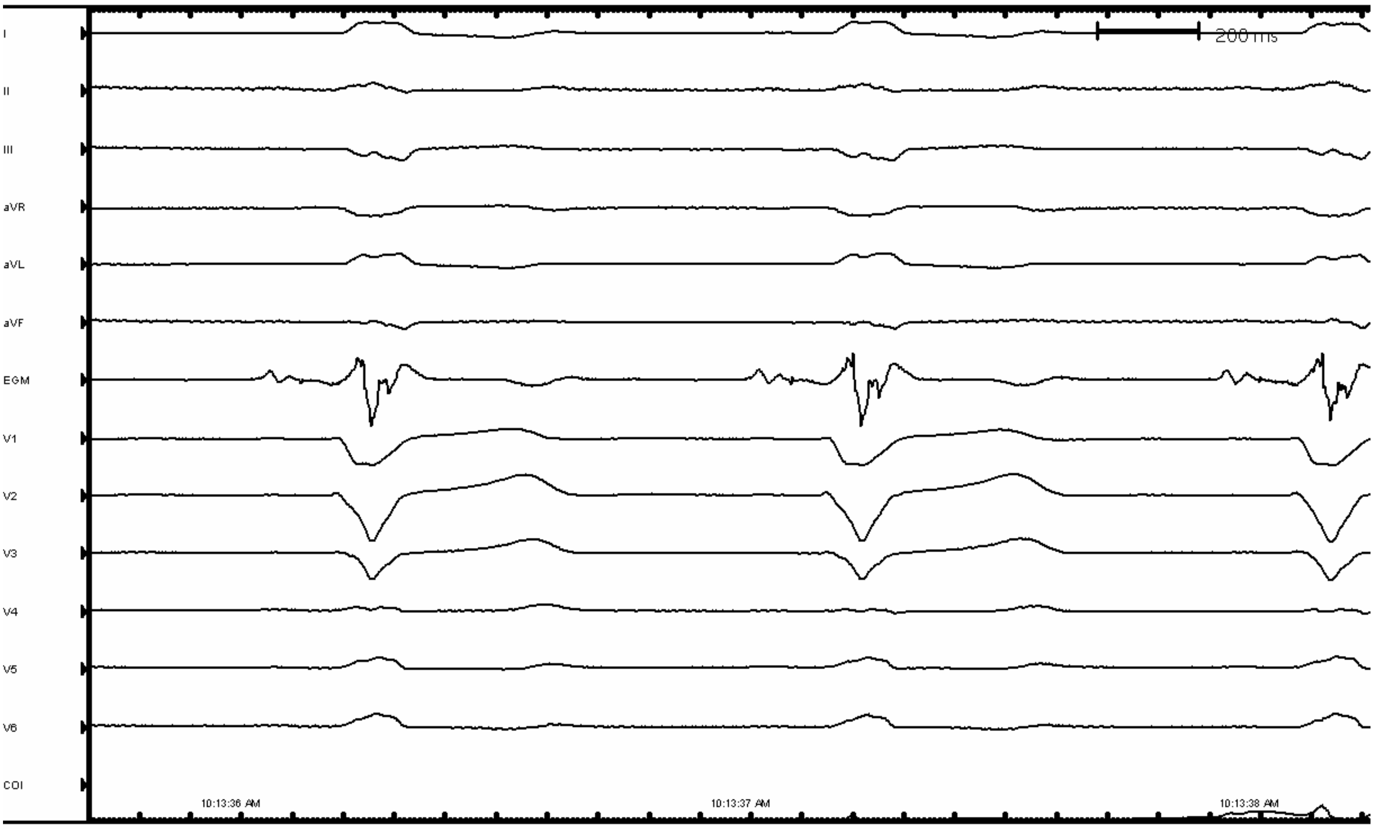

Post ECG

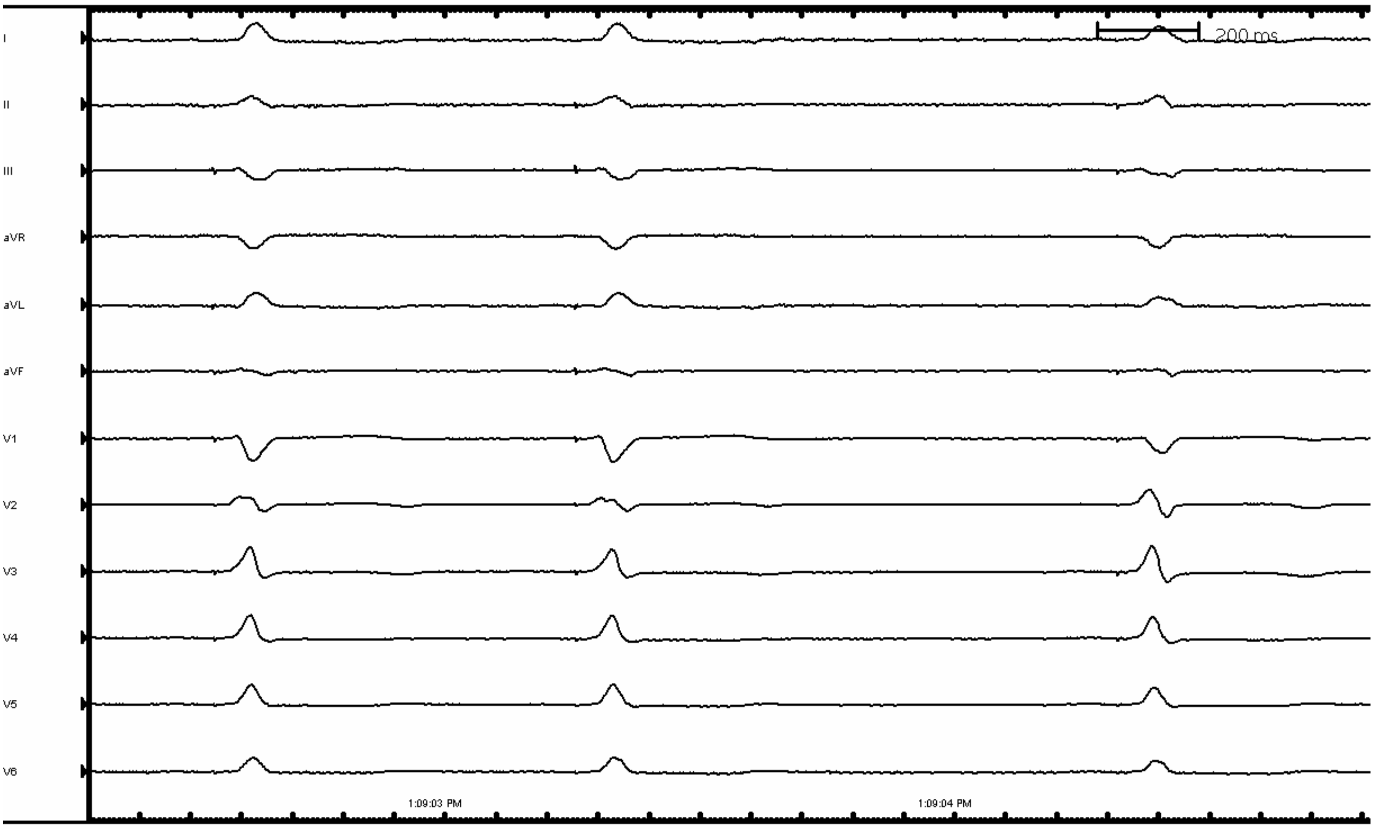

Patient 64: Transitions

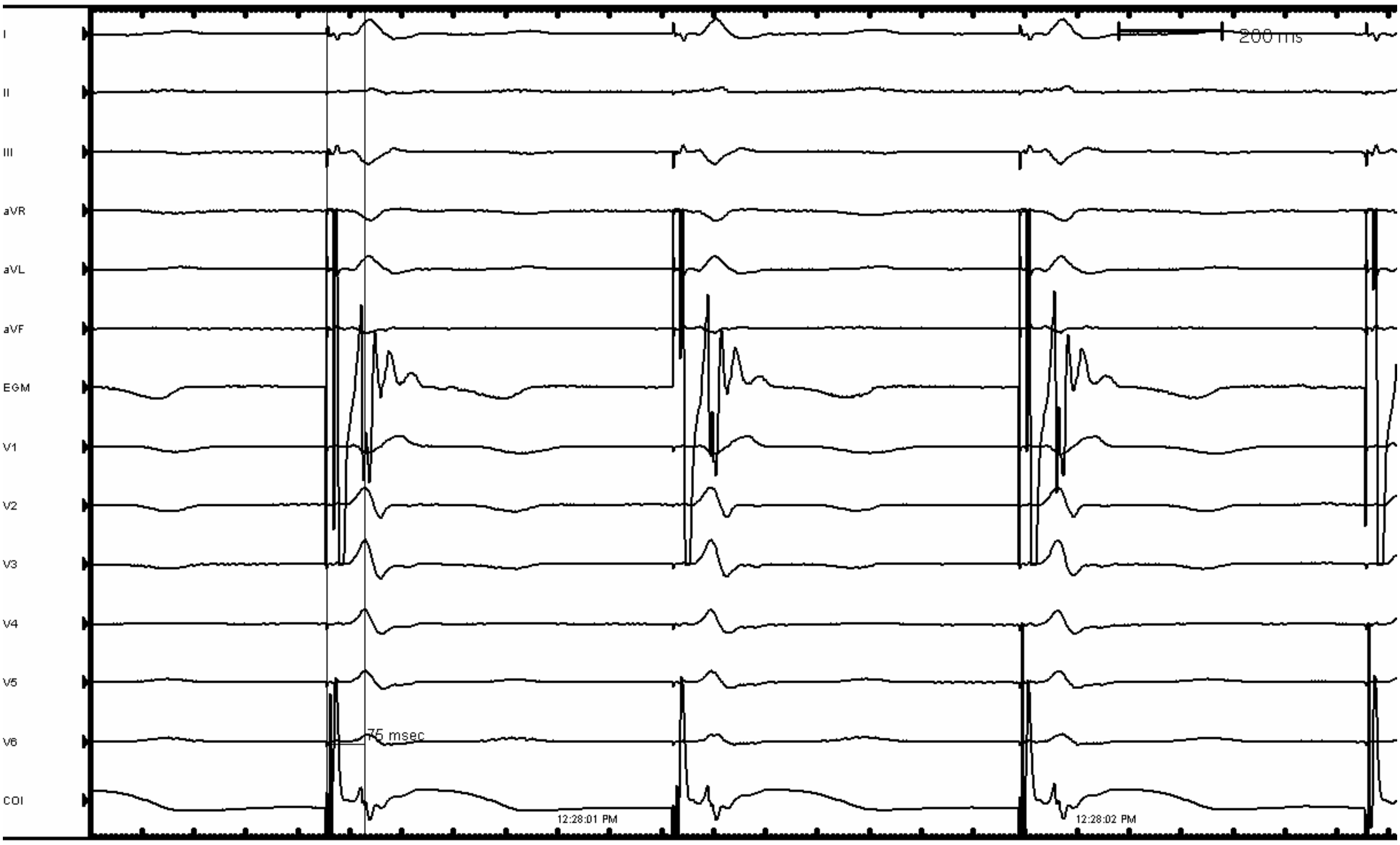

Transitions

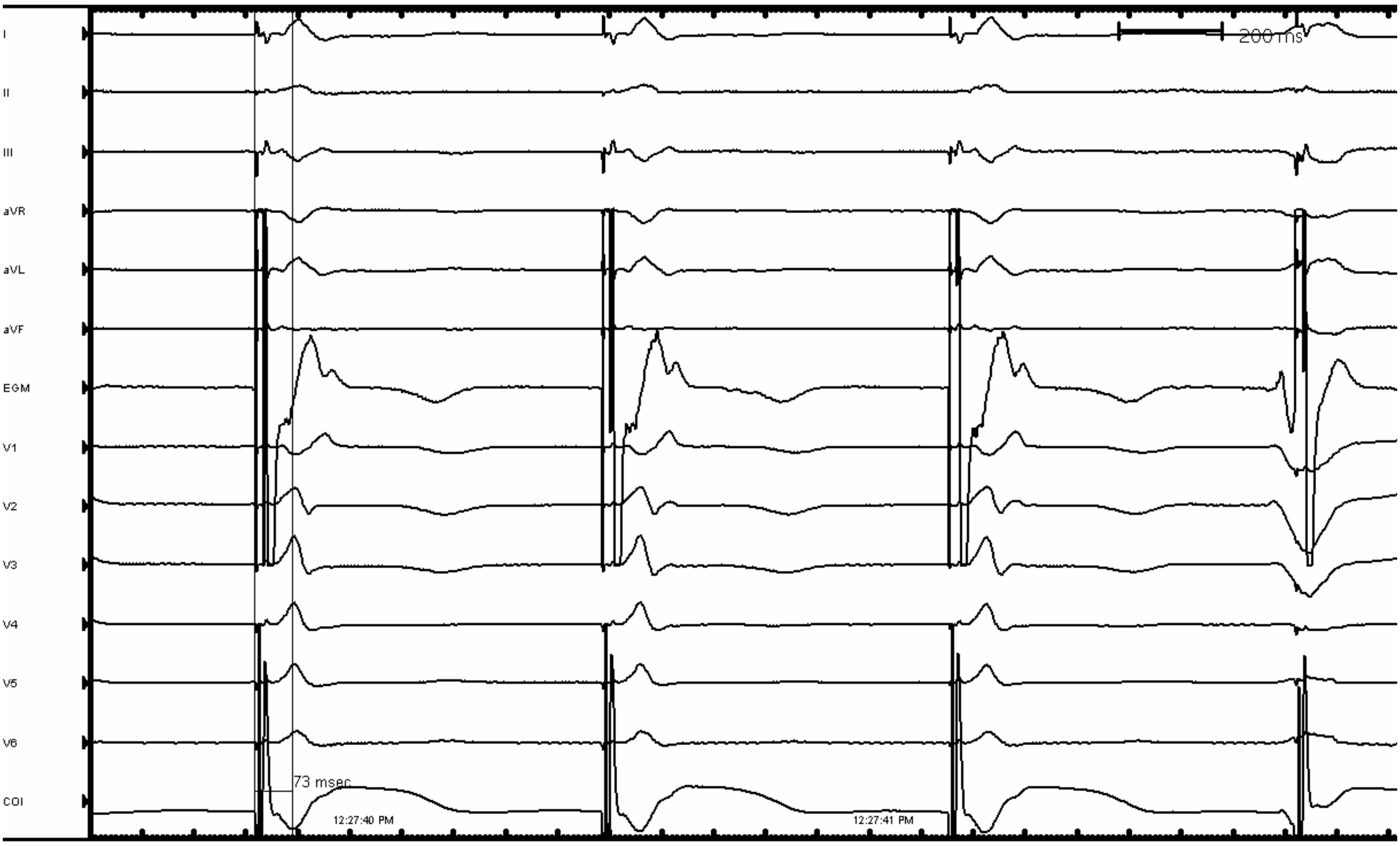

Patient 65:
Pre-ECG

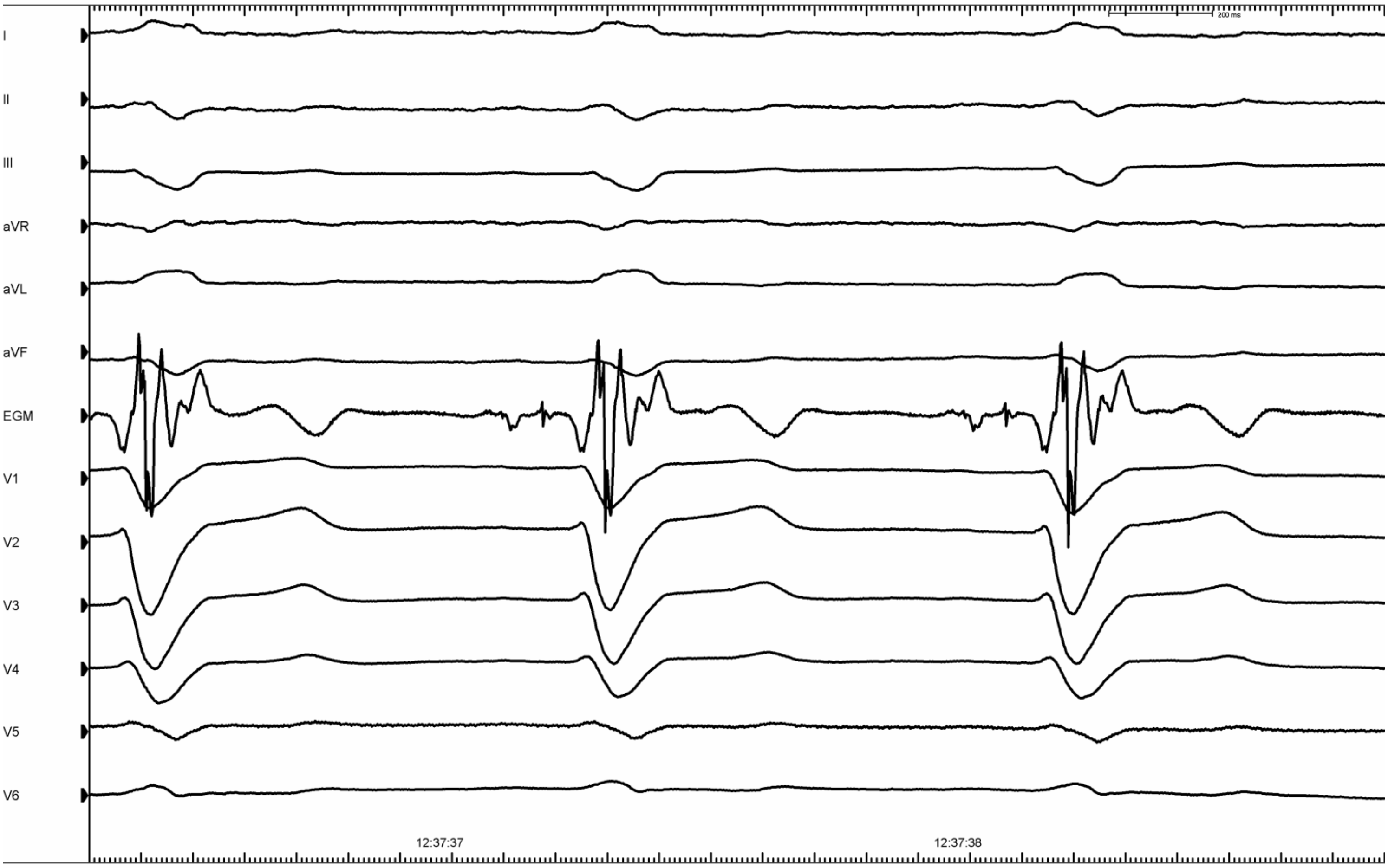

Post ECG

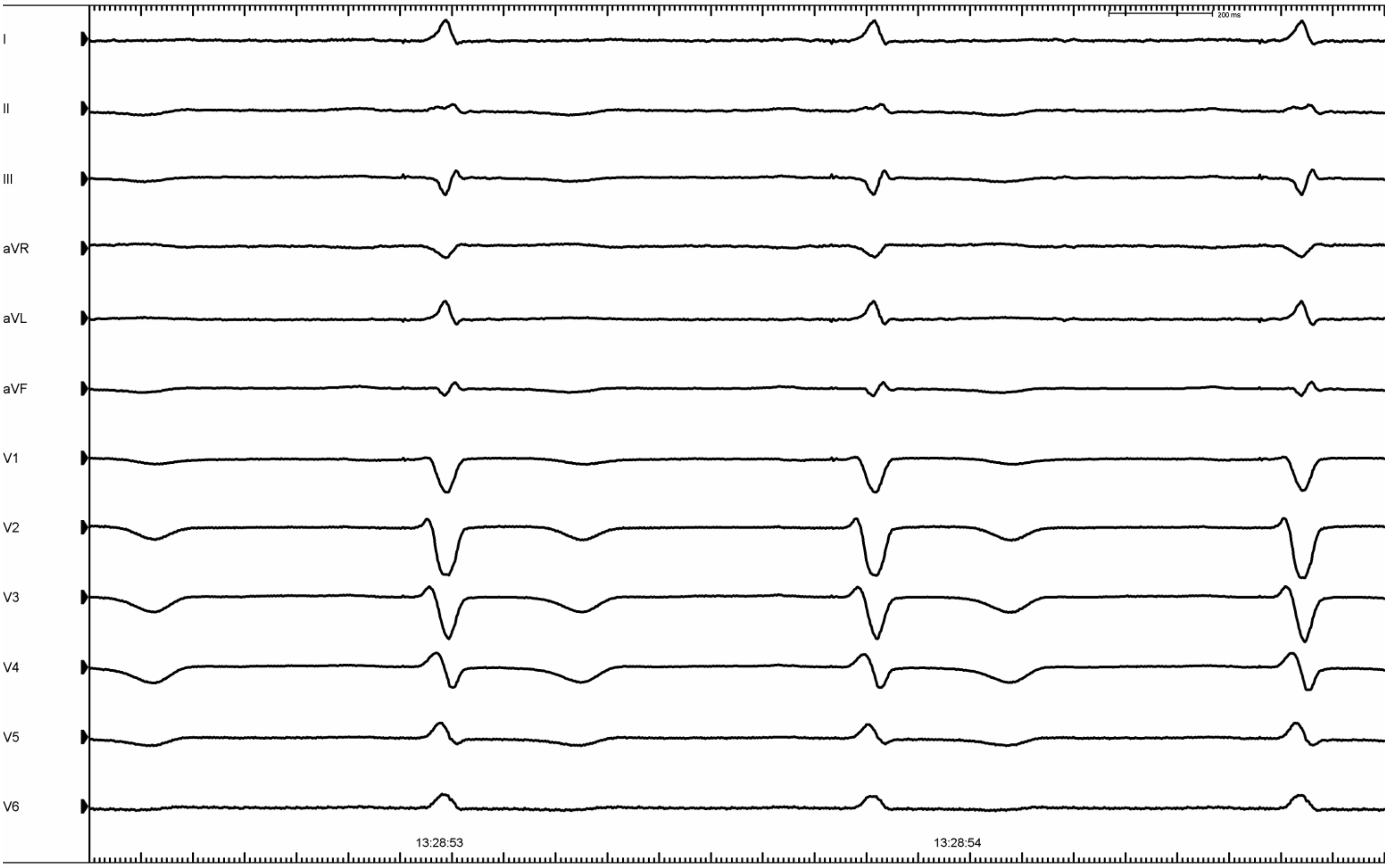

Patient 65: Transitions

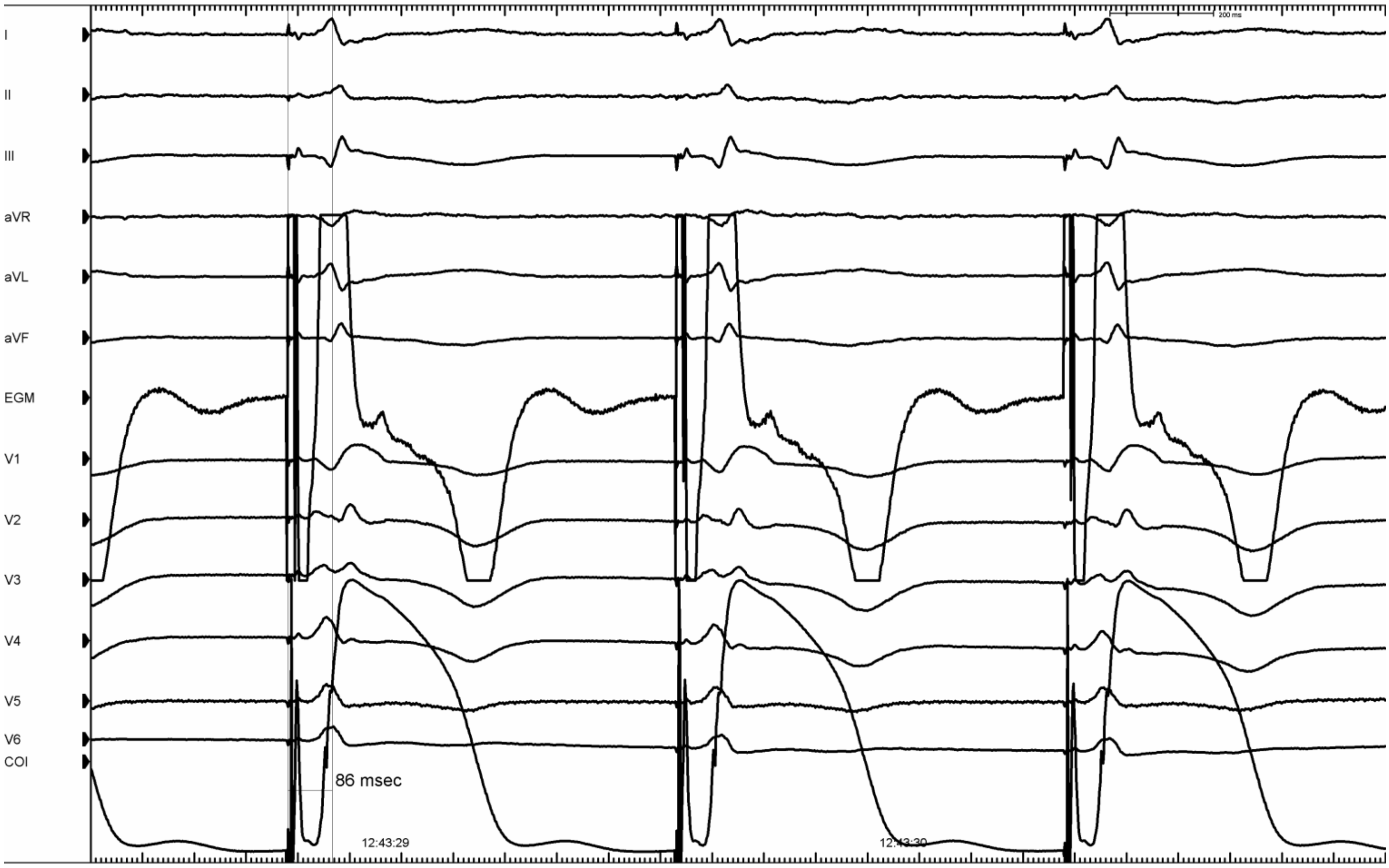

Transitions

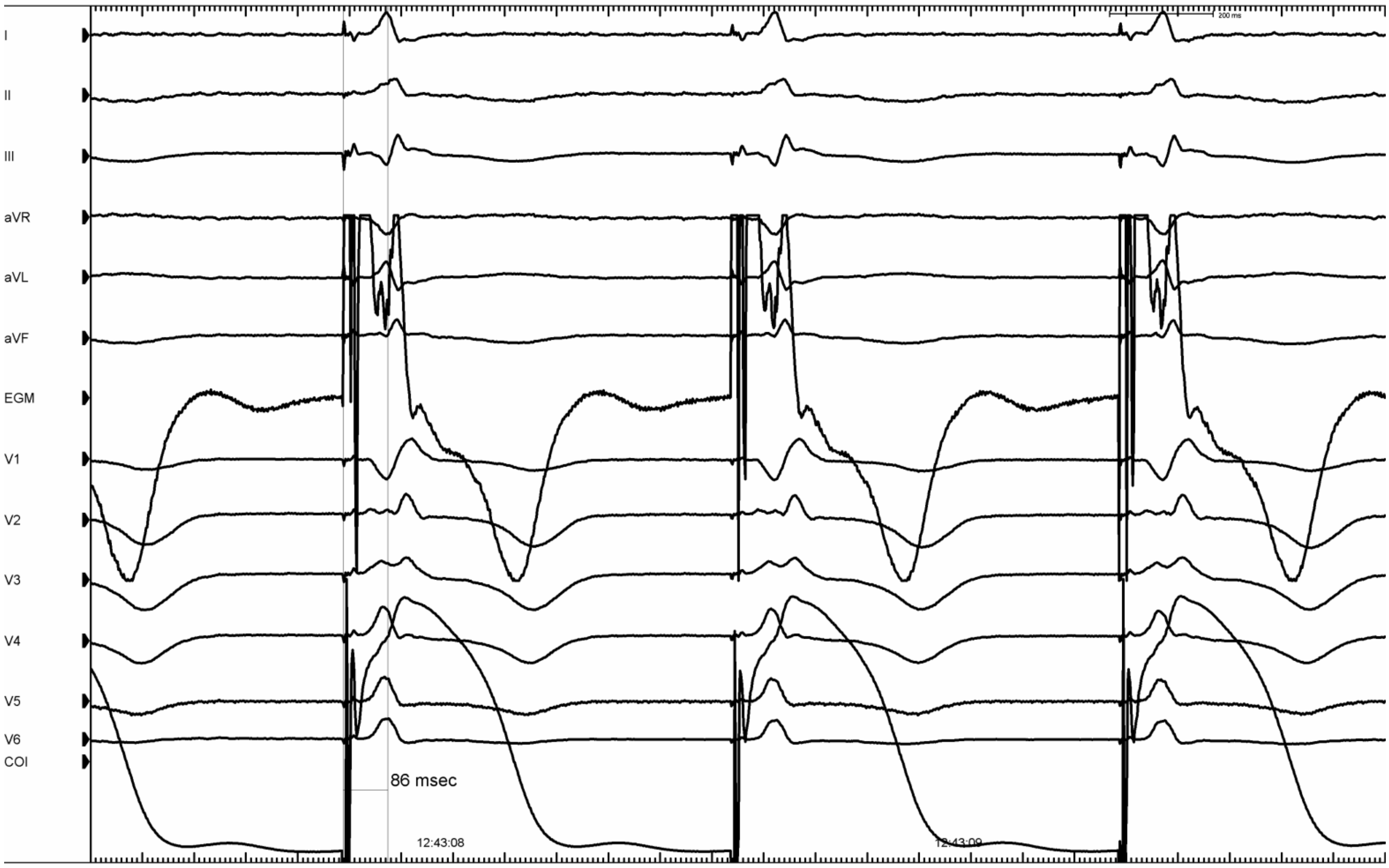

Patient 66:
Pre-ECG

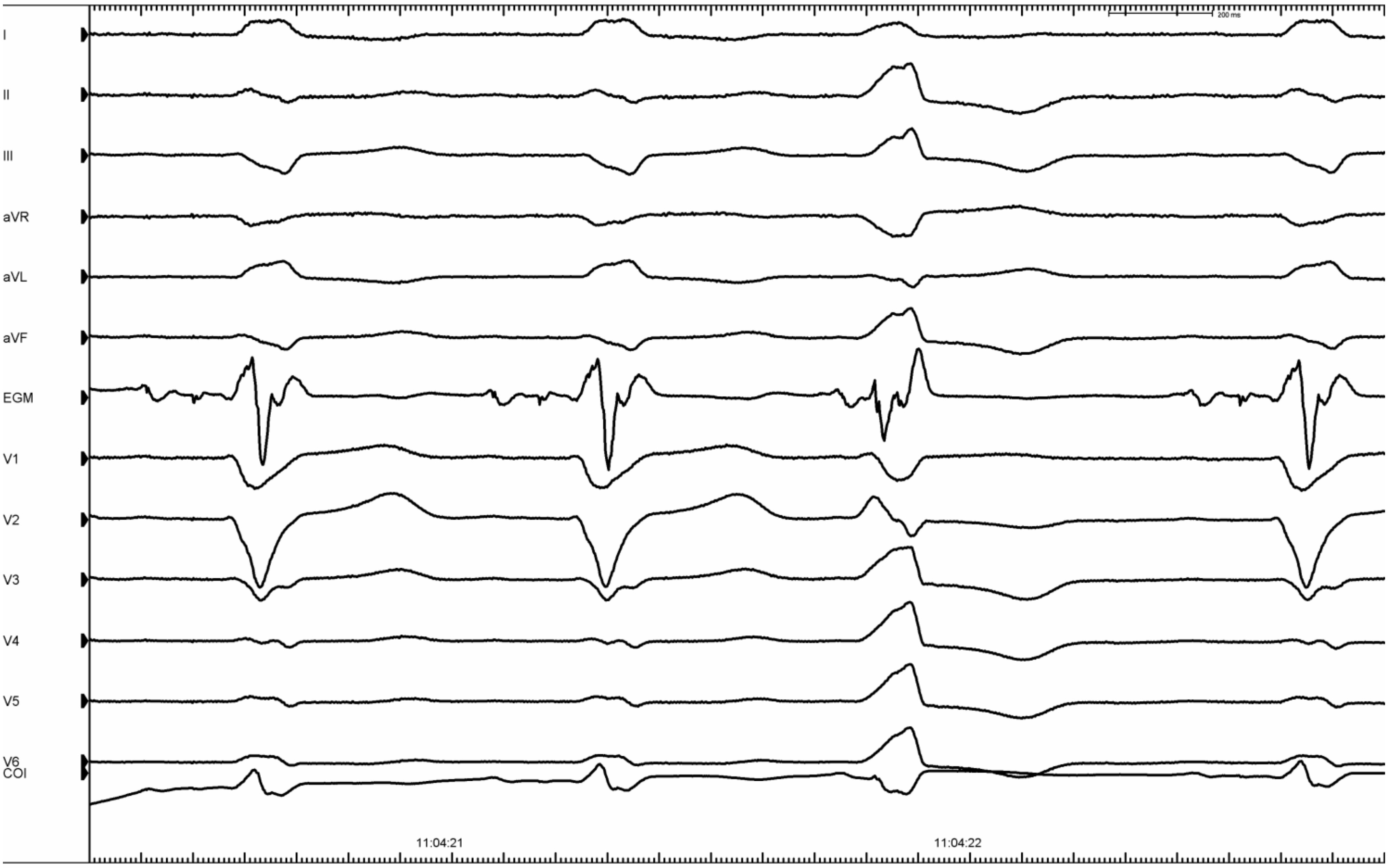

Post ECG

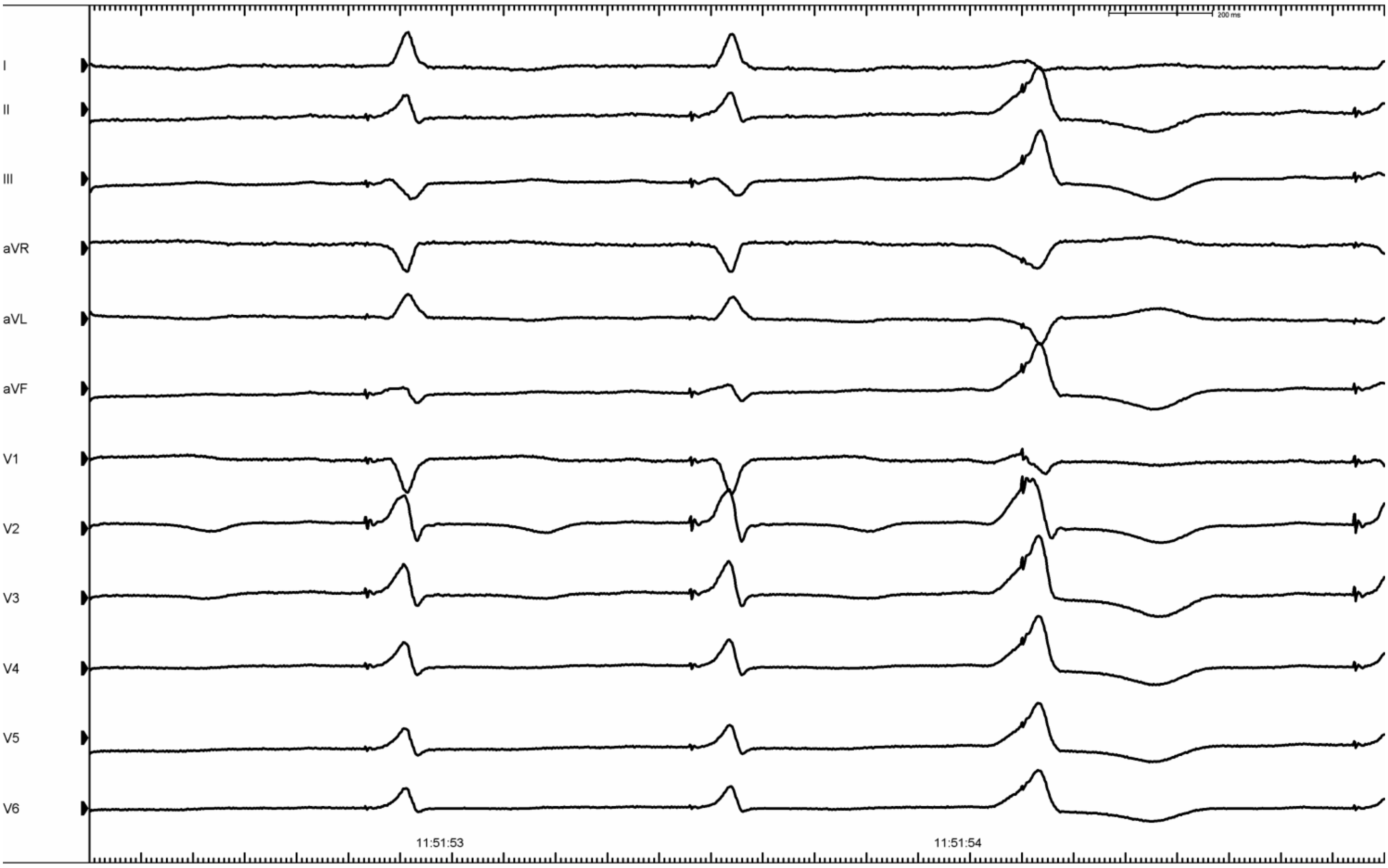

Patient 66:
Transitions

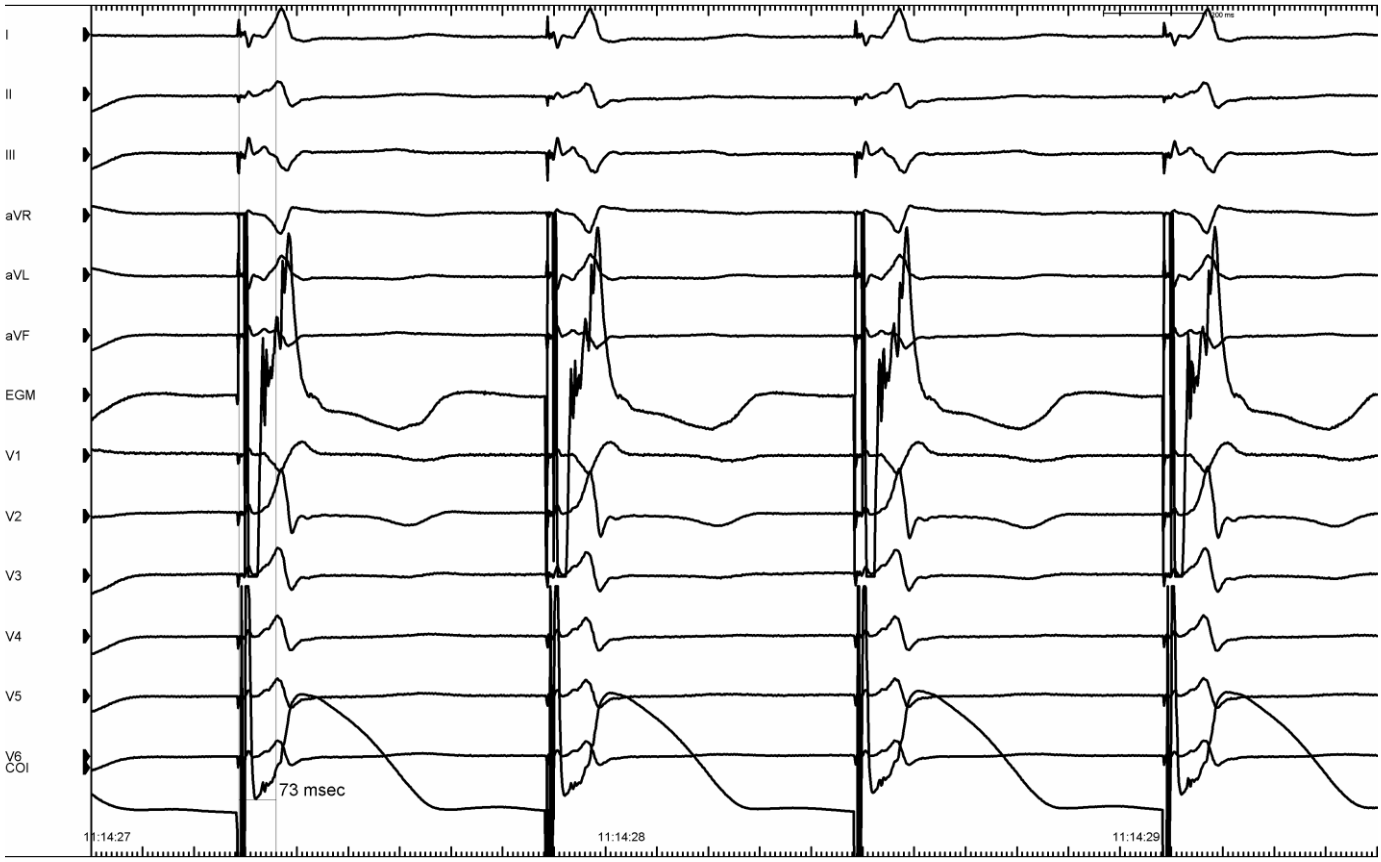

Transitions

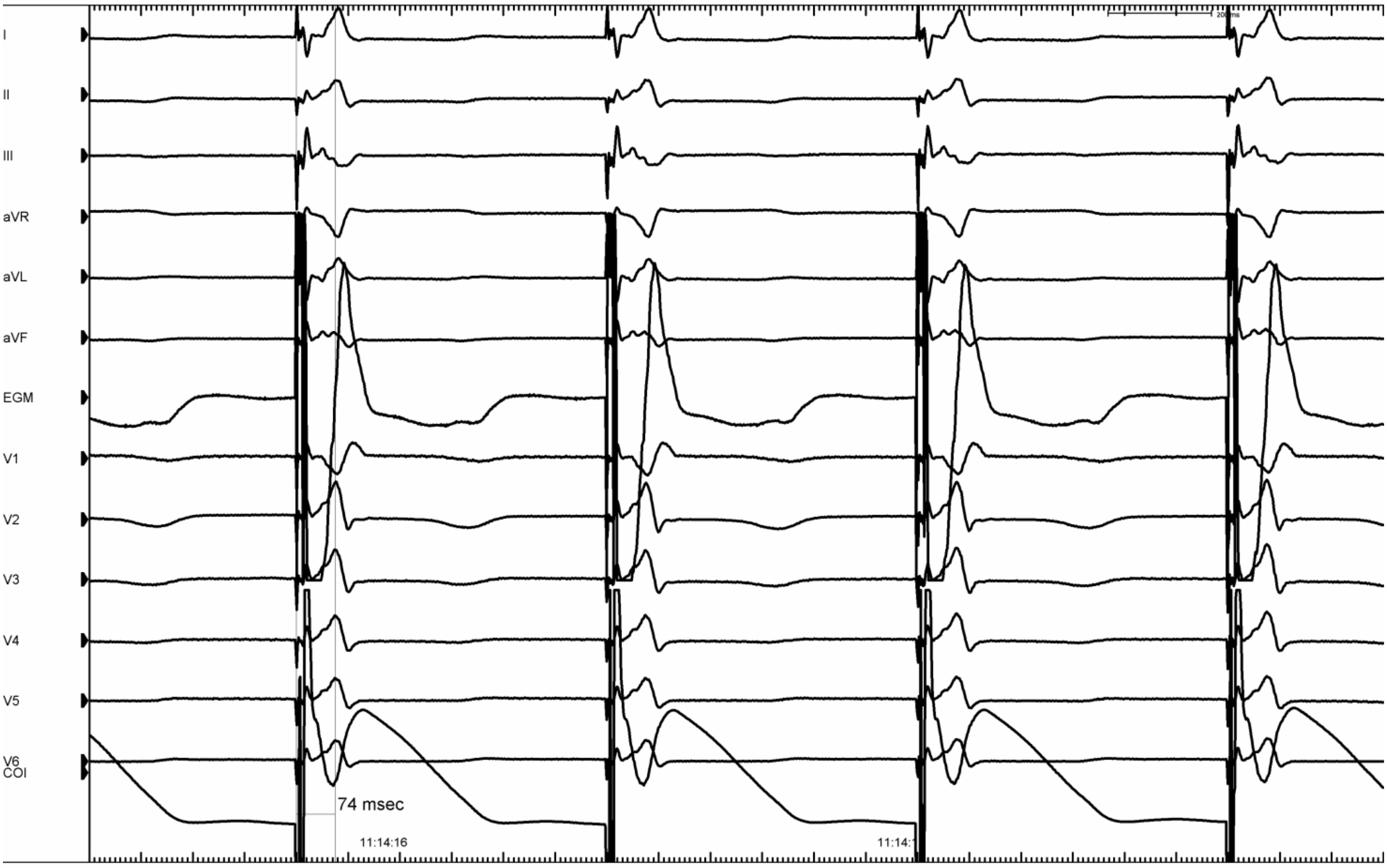

Patient 67:
Pre-ECG

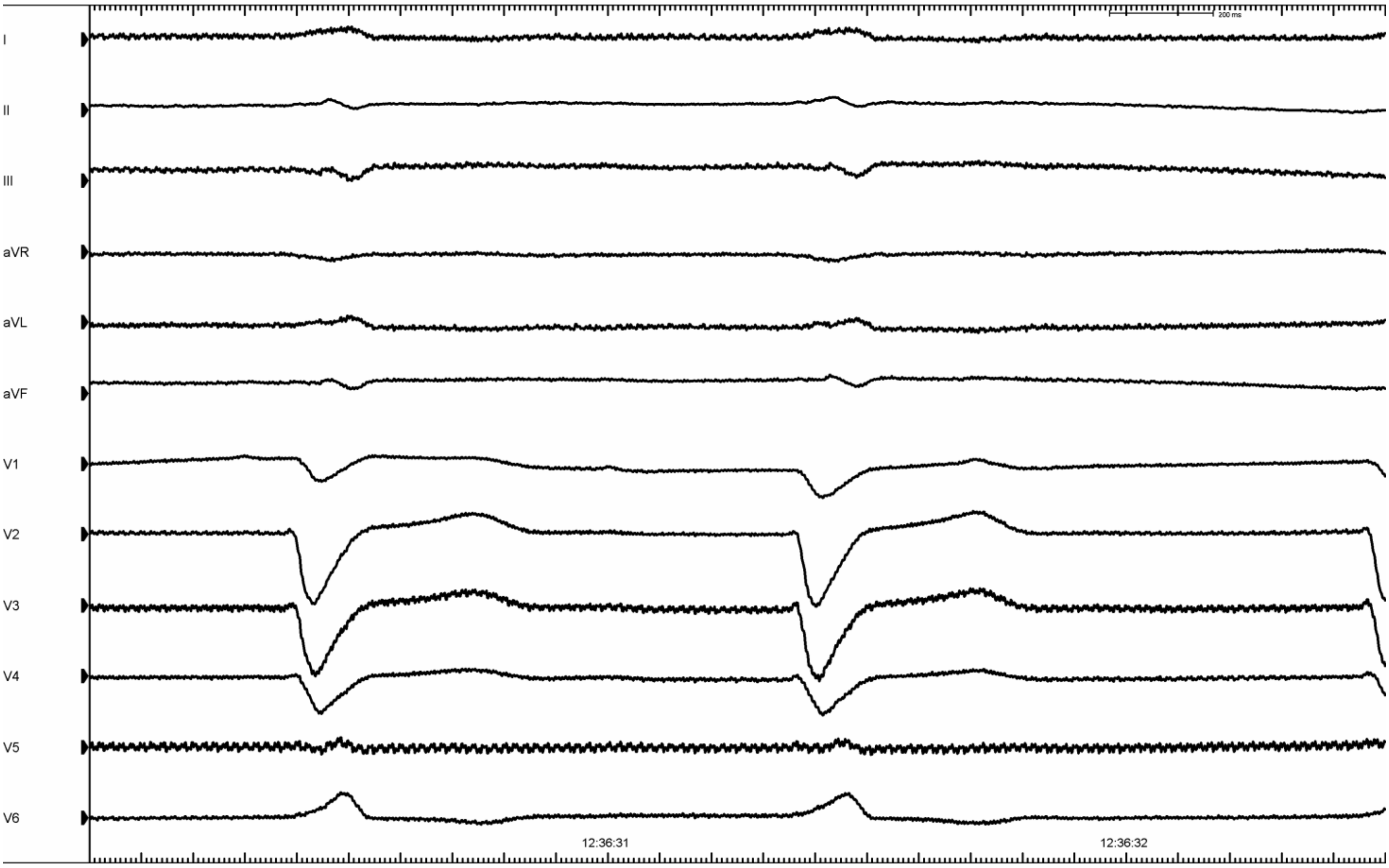

Post ECG

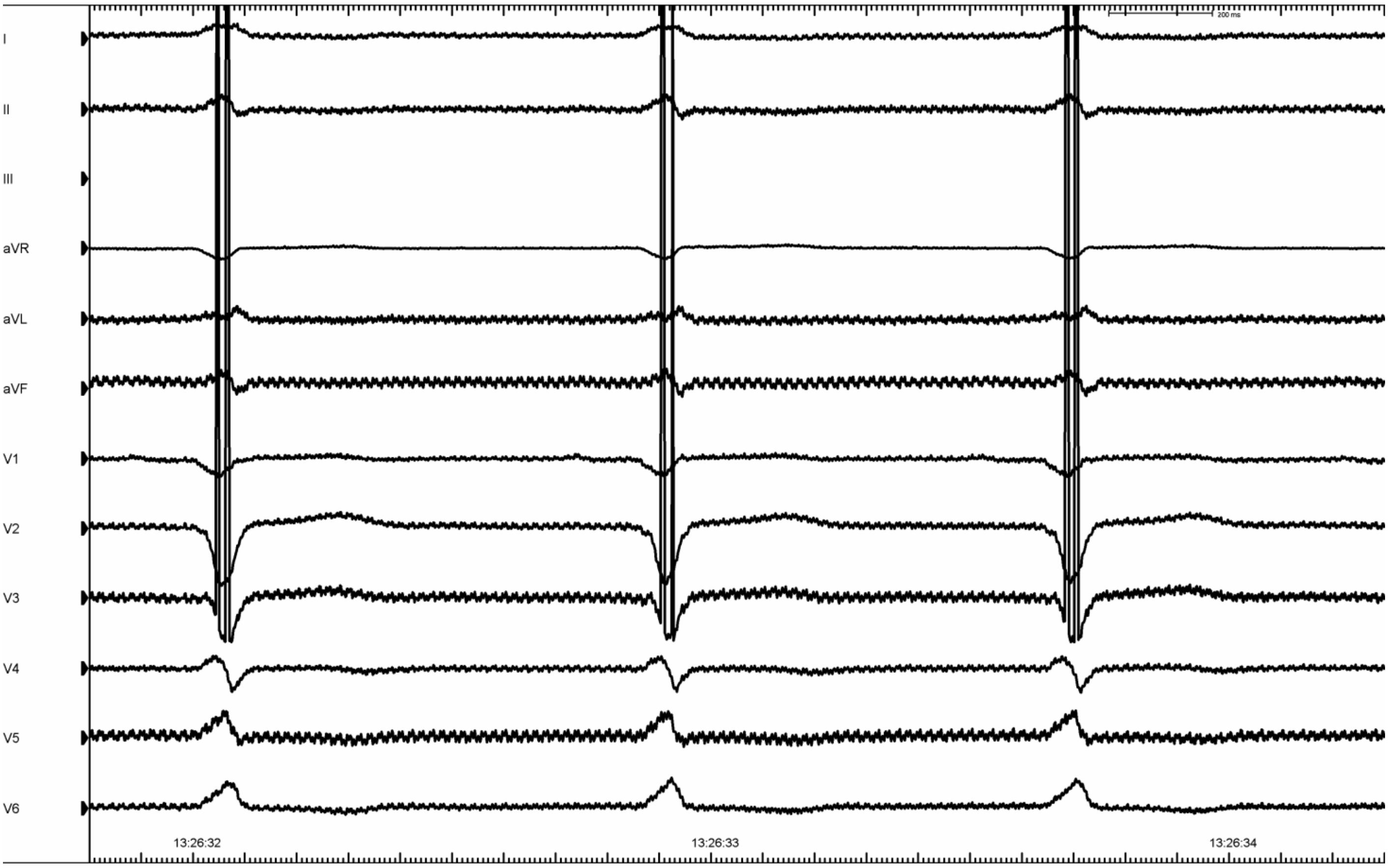

Patient 67:
Transitions

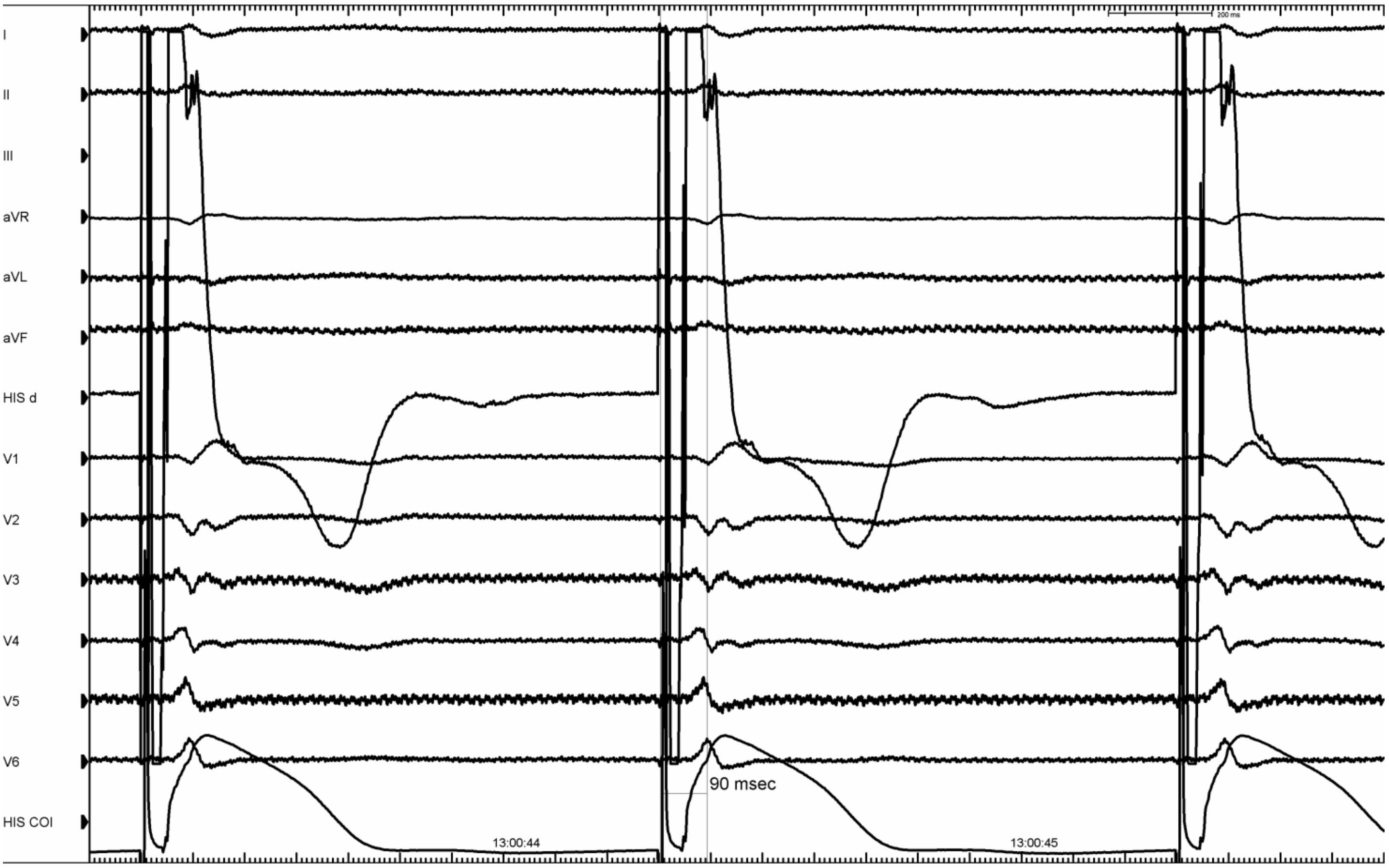

Transitions

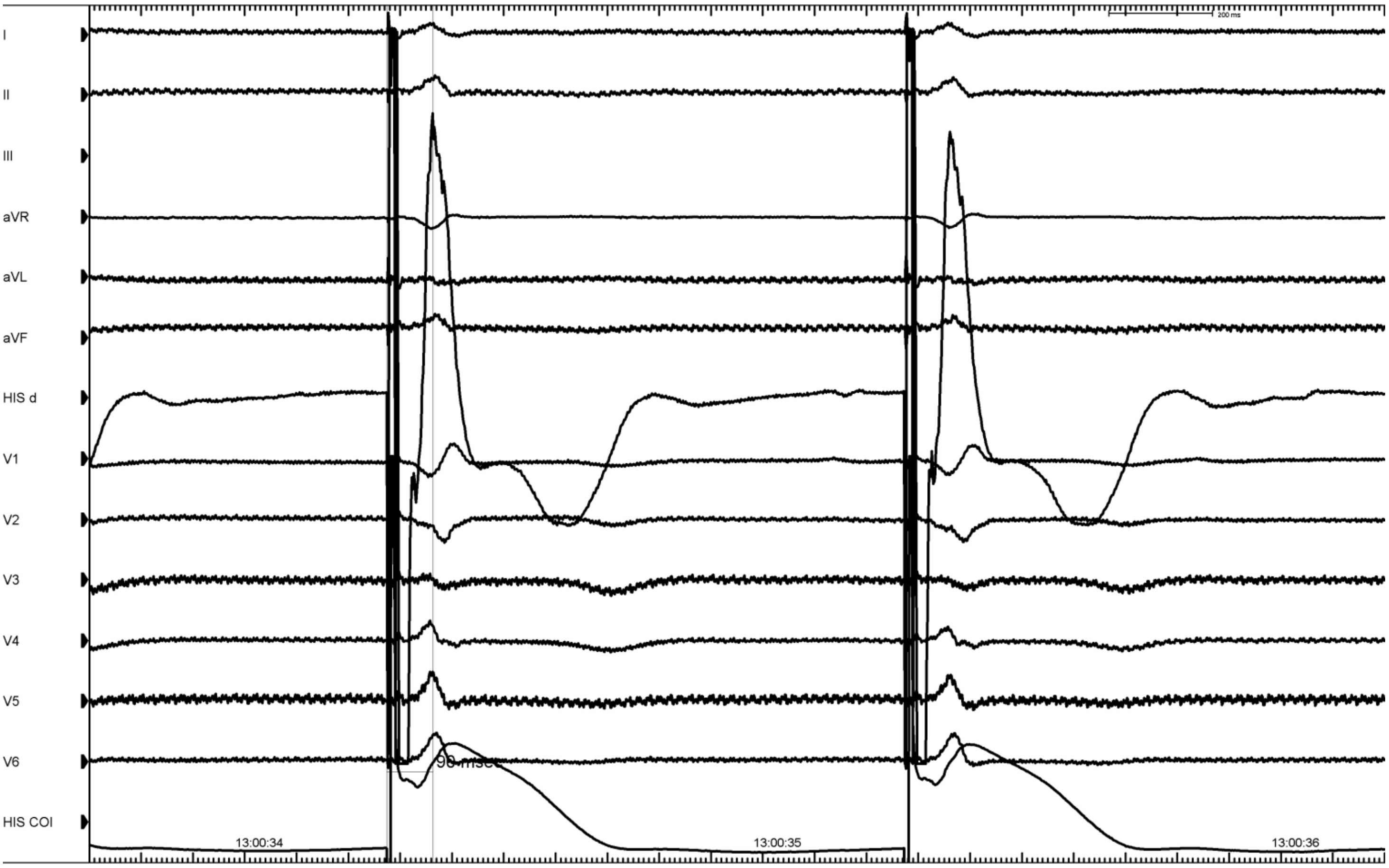

Patient 68:
Pre-ECG

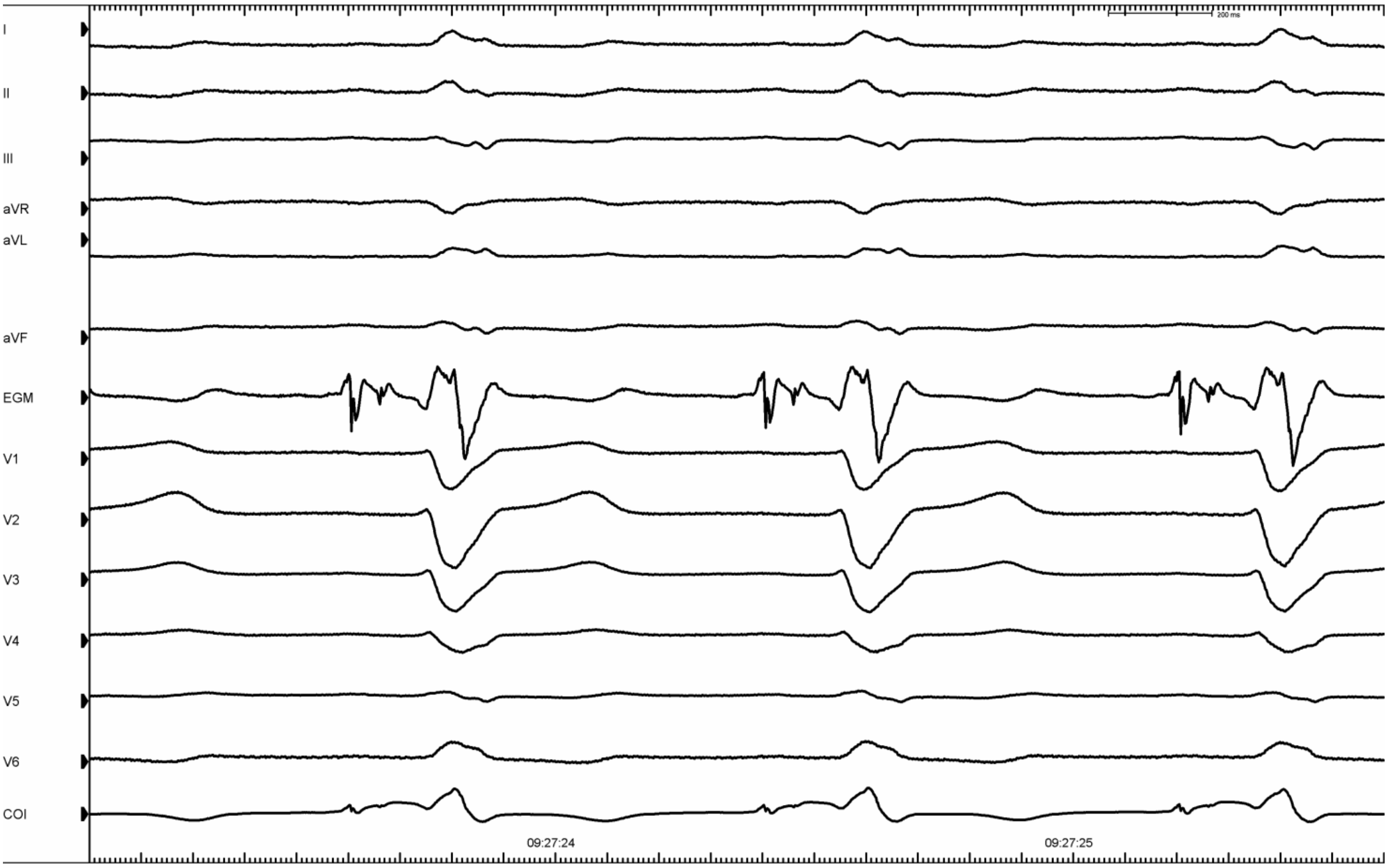

Post ECG

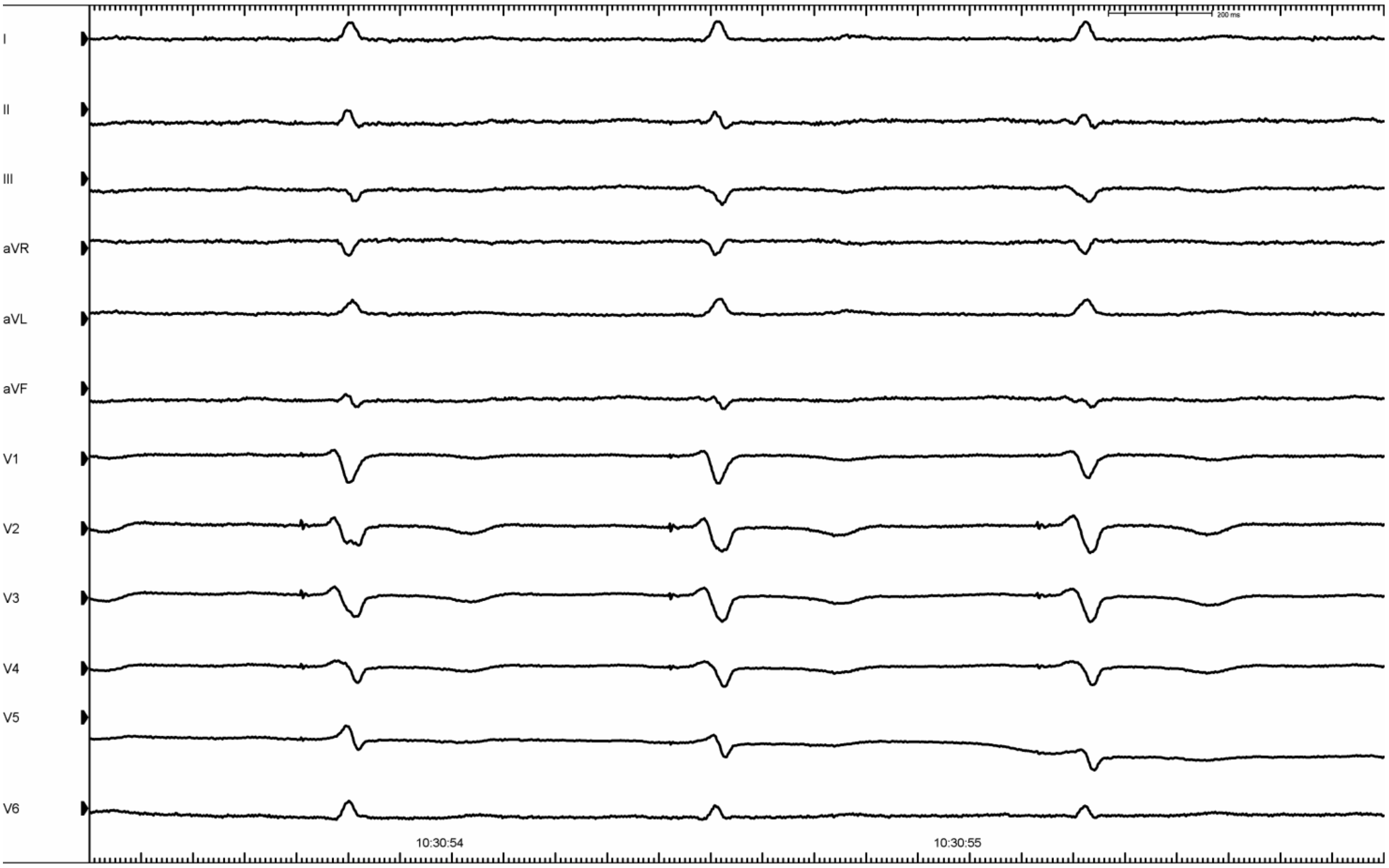

Patient 68:
Transitions

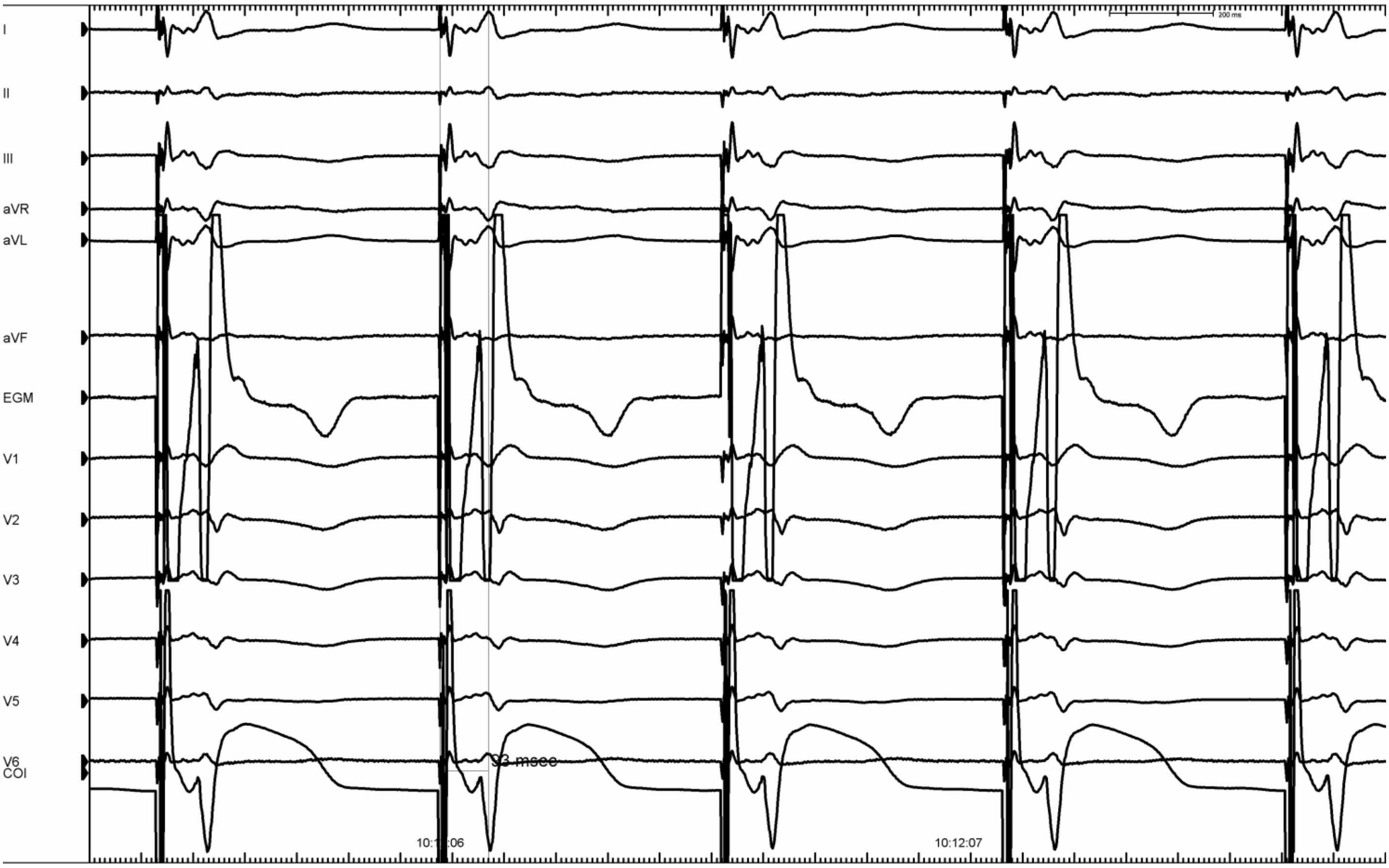

Transitions

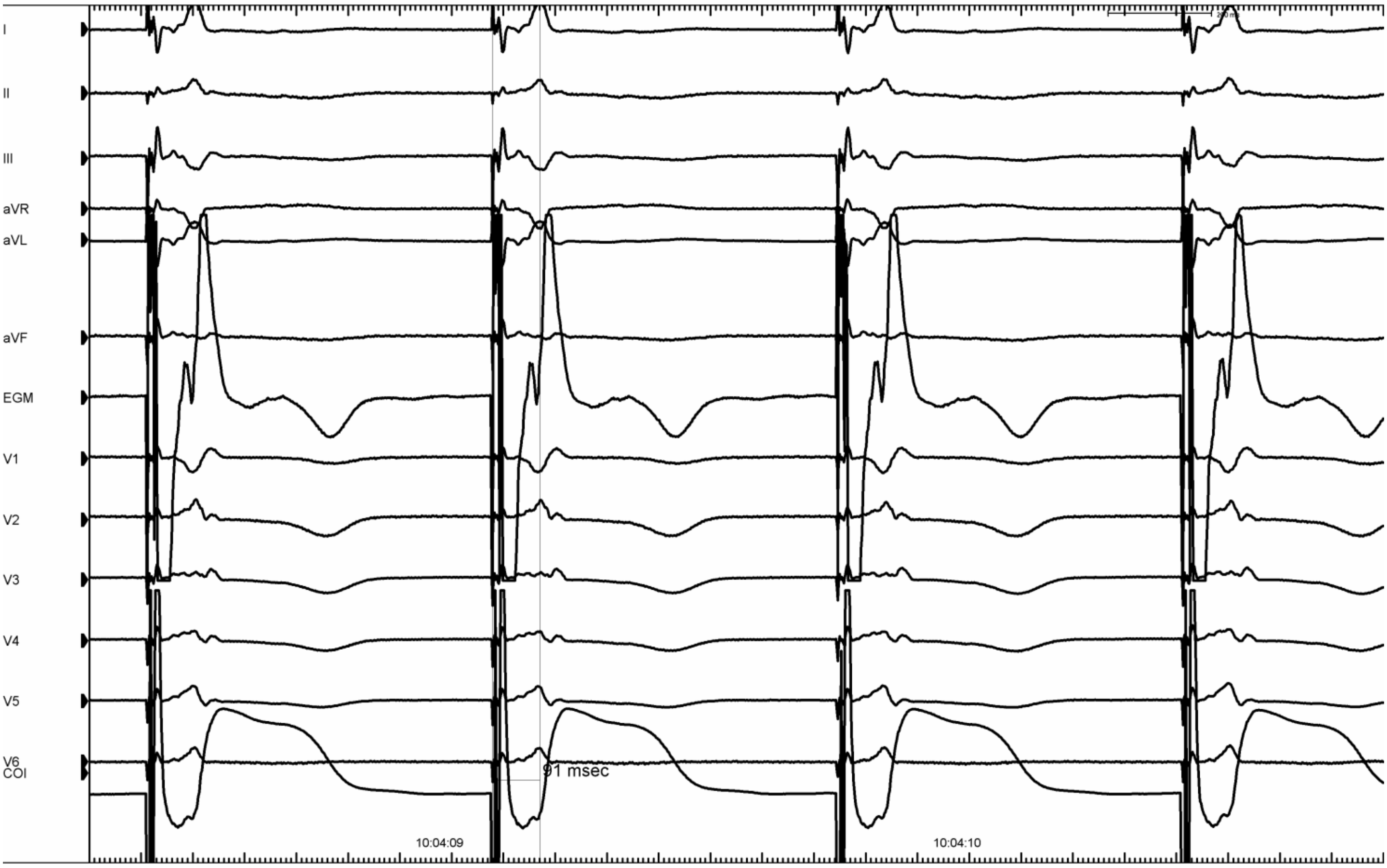

Patient 69:
Pre-ECG

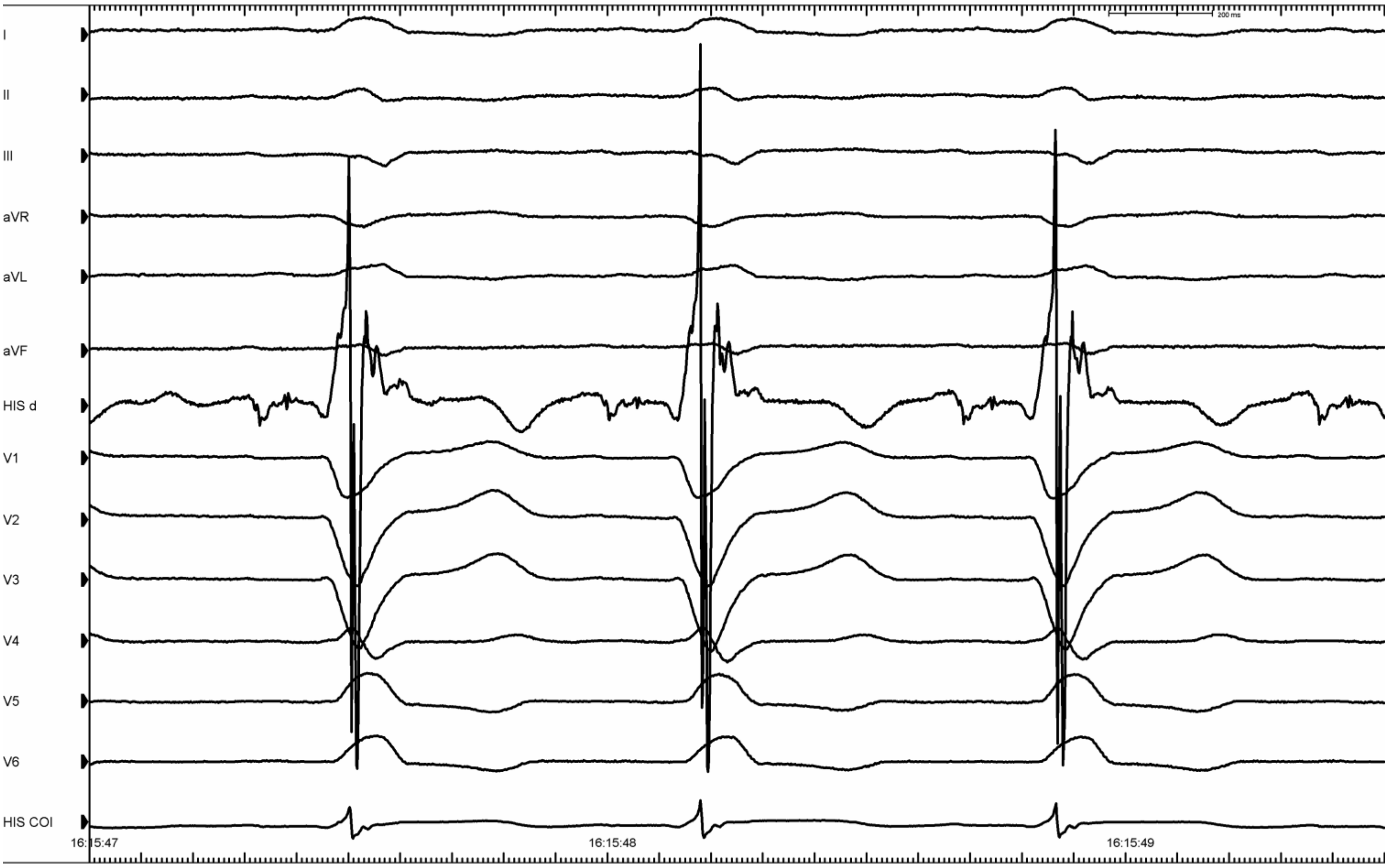

Post ECG

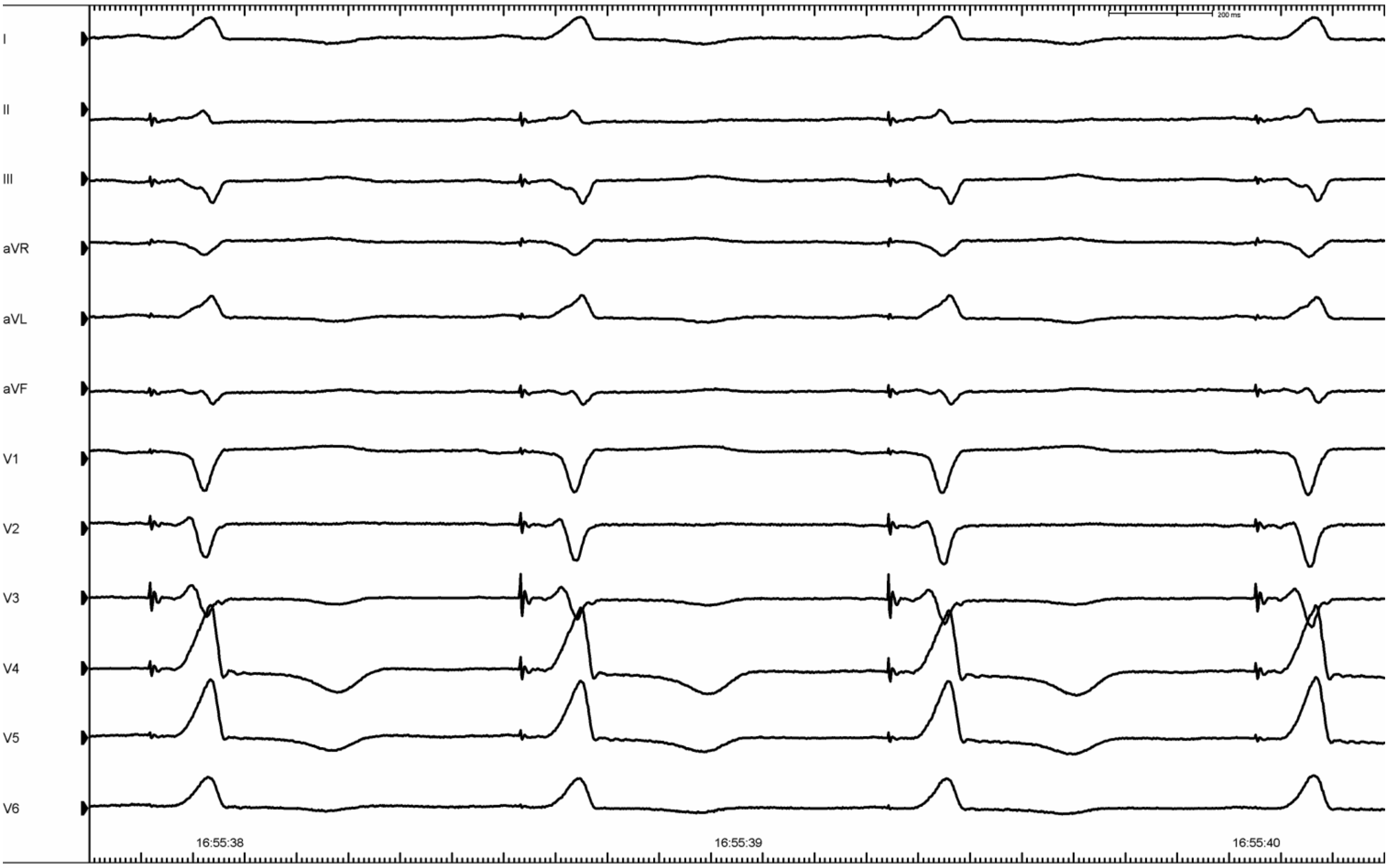

Patient 69:
Transitions

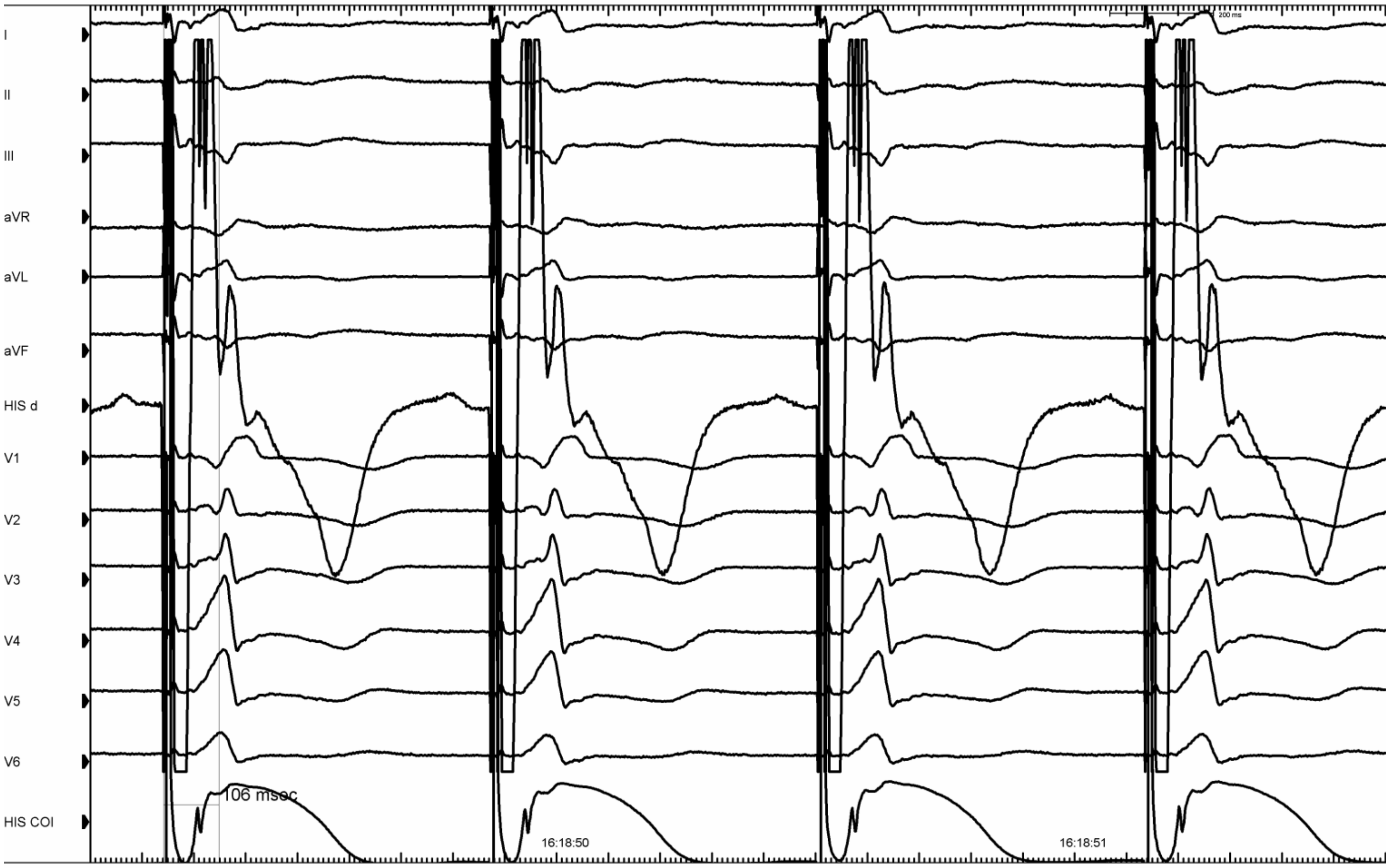

Transitions

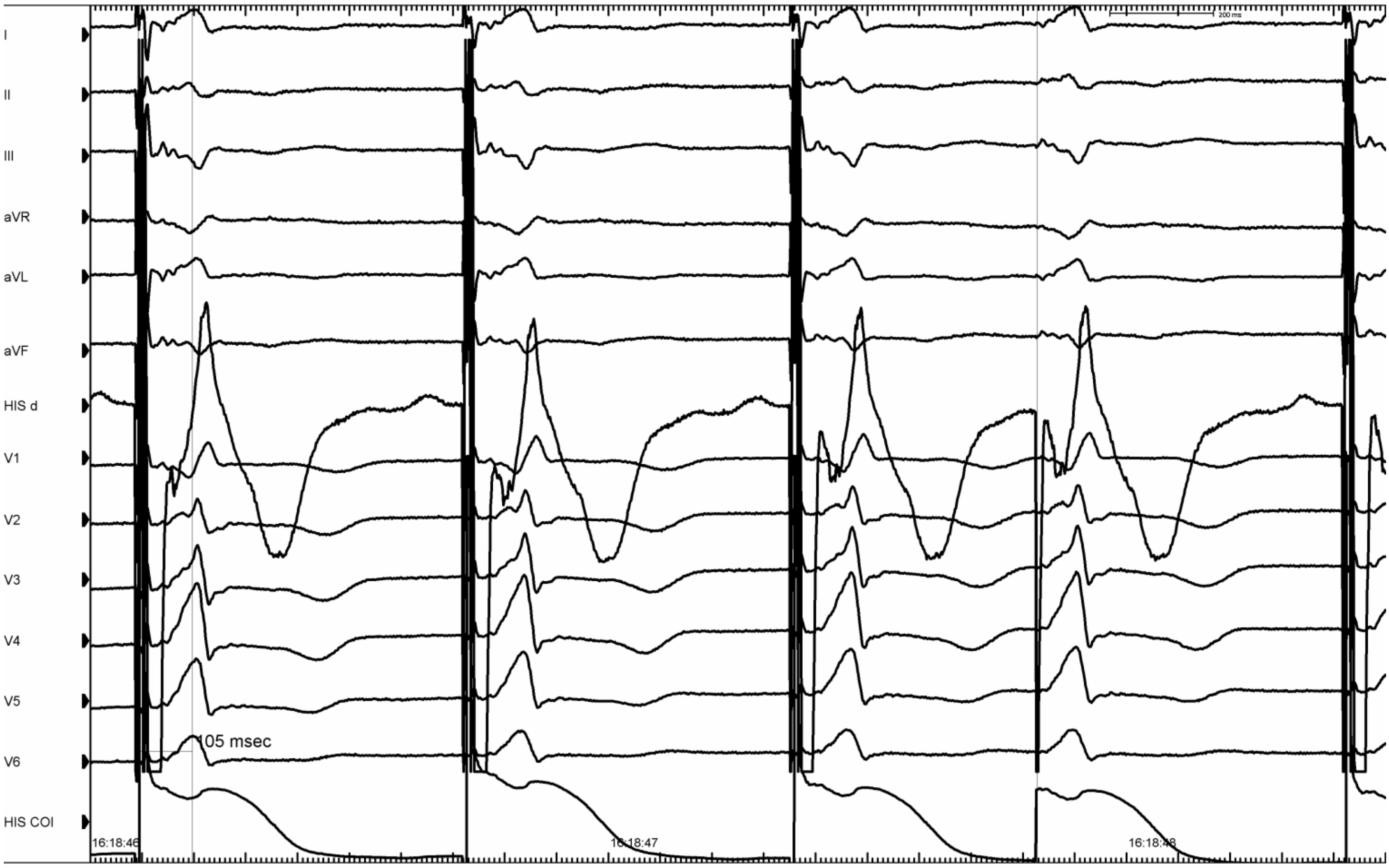

Patient 70:

Pre-ECG

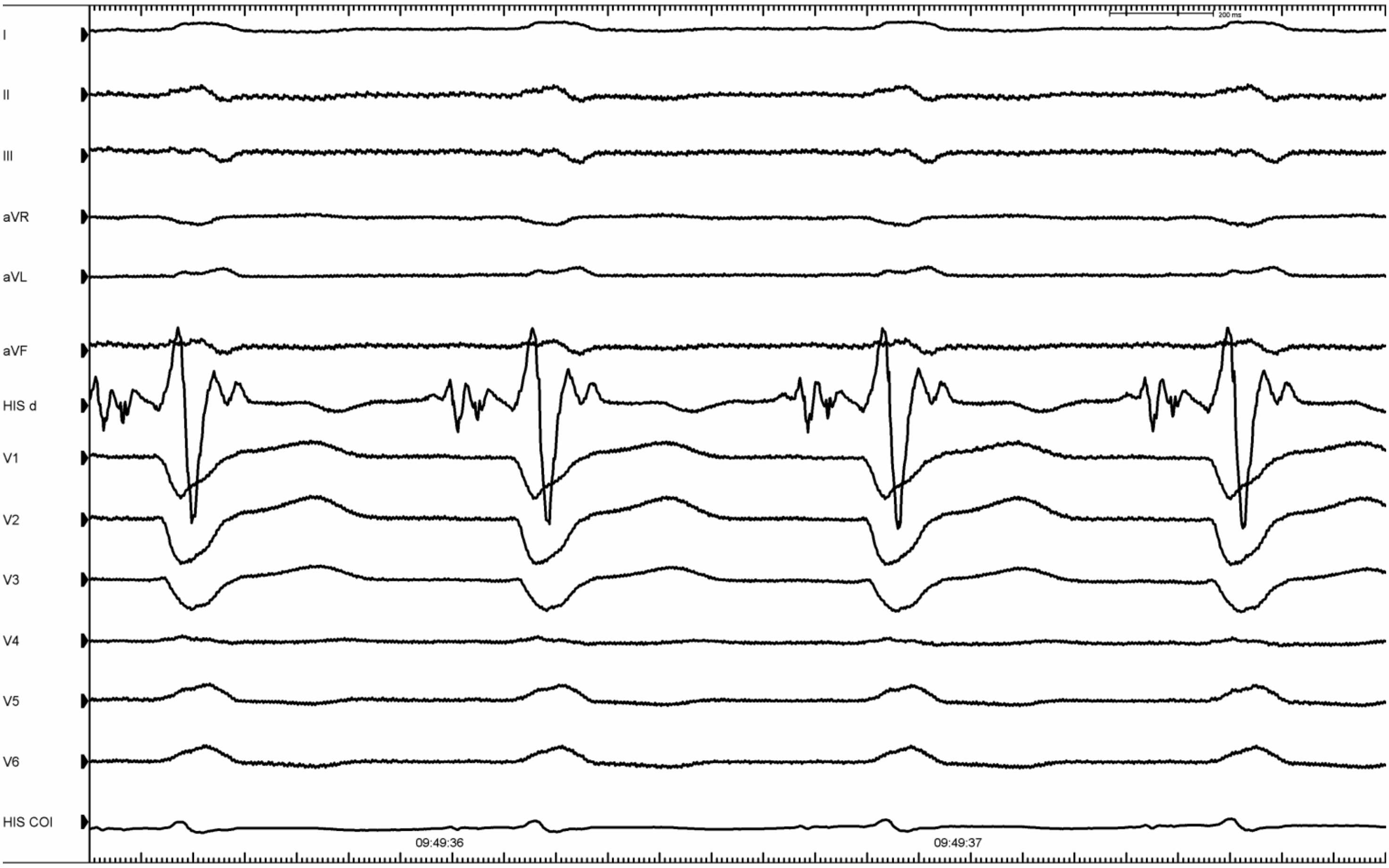

Post ECG

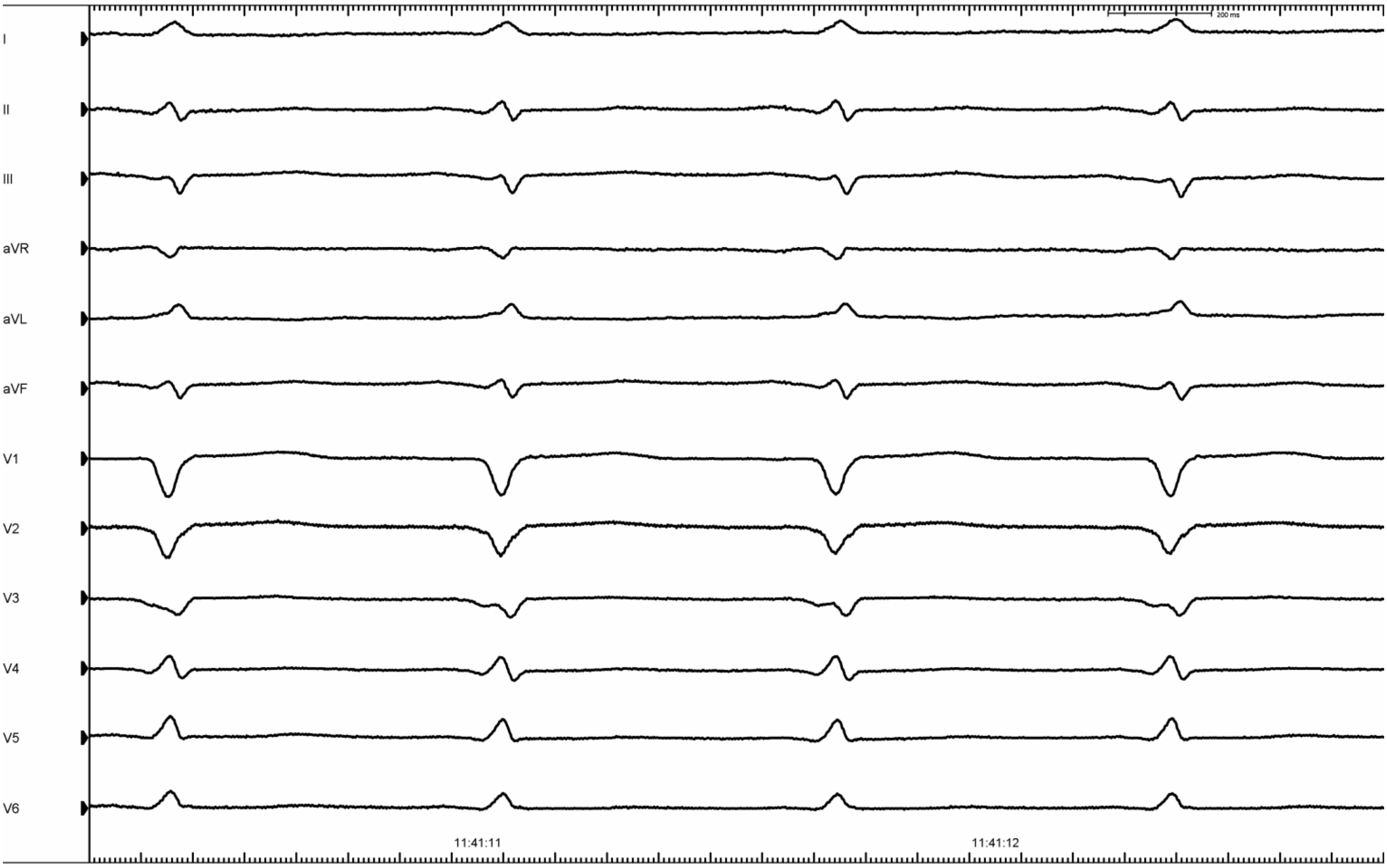

Patient 70:
Transitions

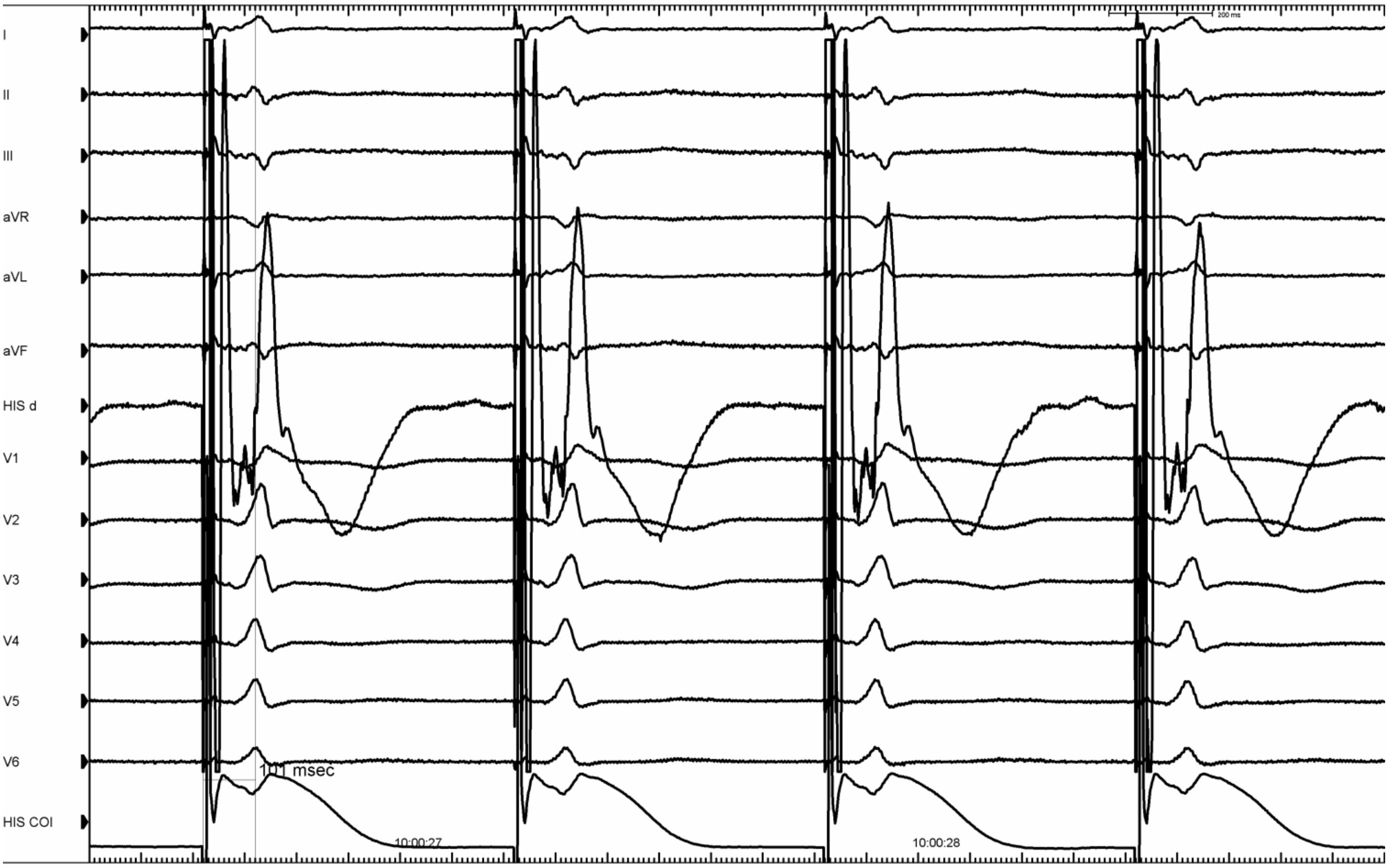

Transitions

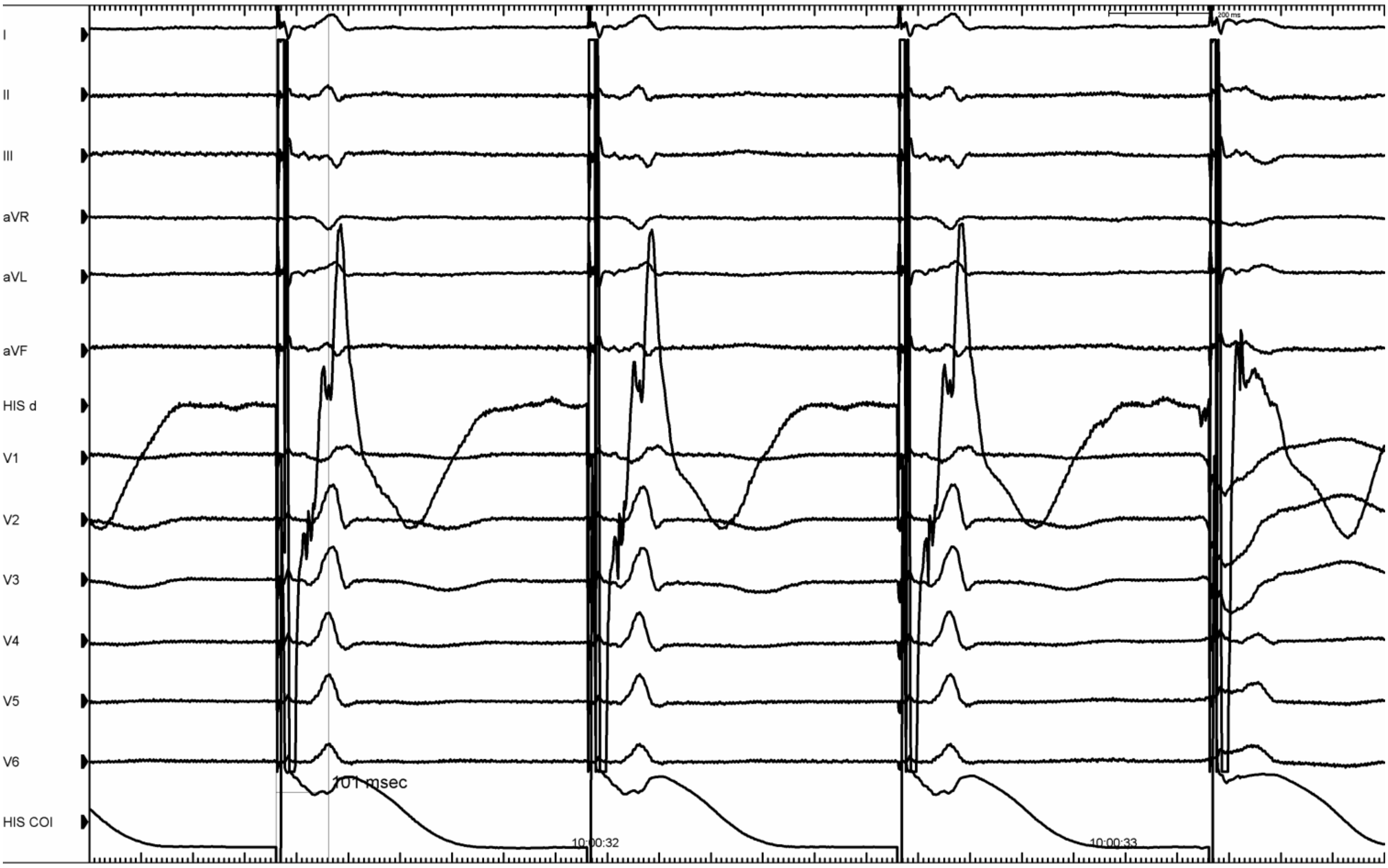

Patient 71:
Pre-ECG

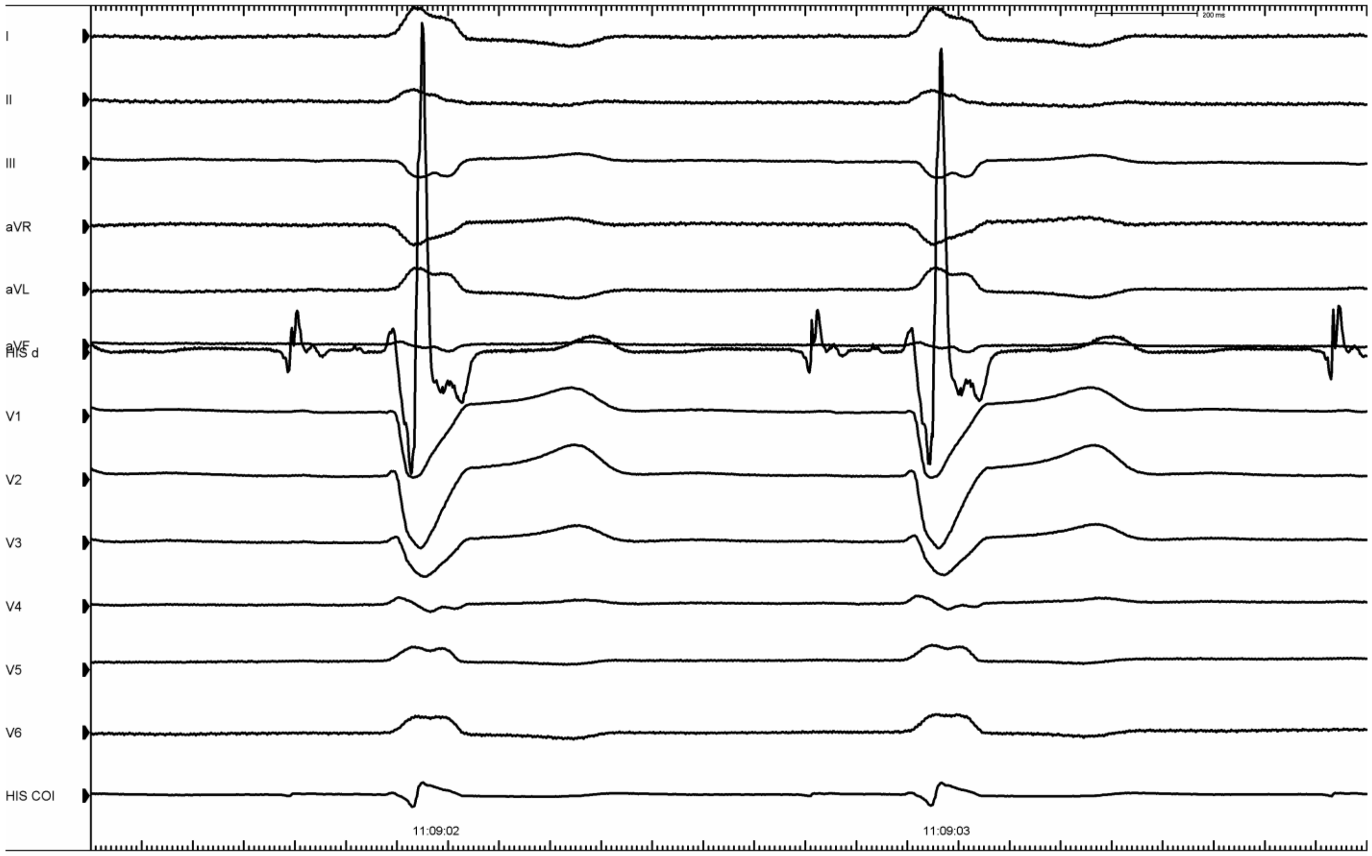

Post ECG

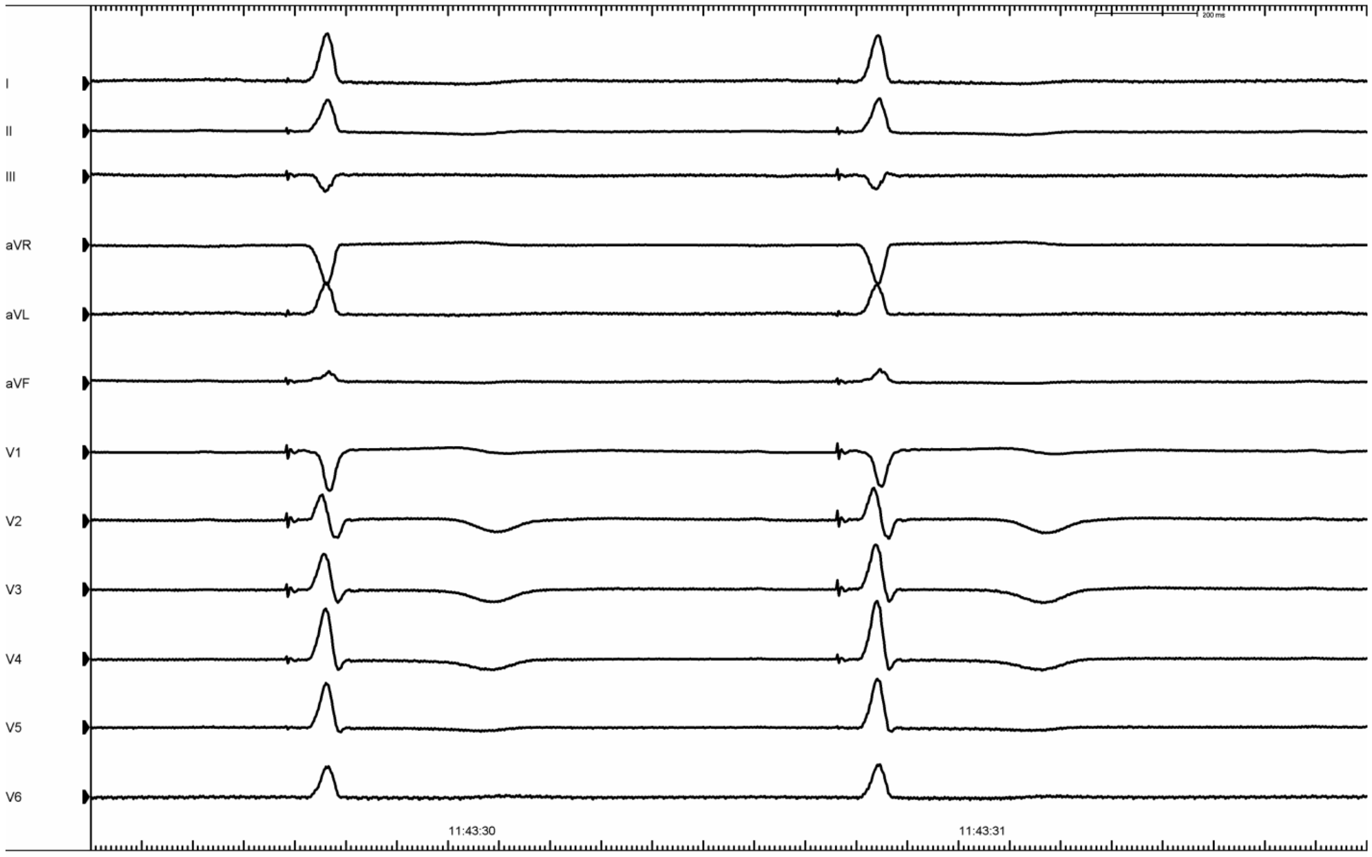

Patient 71:
Transitions

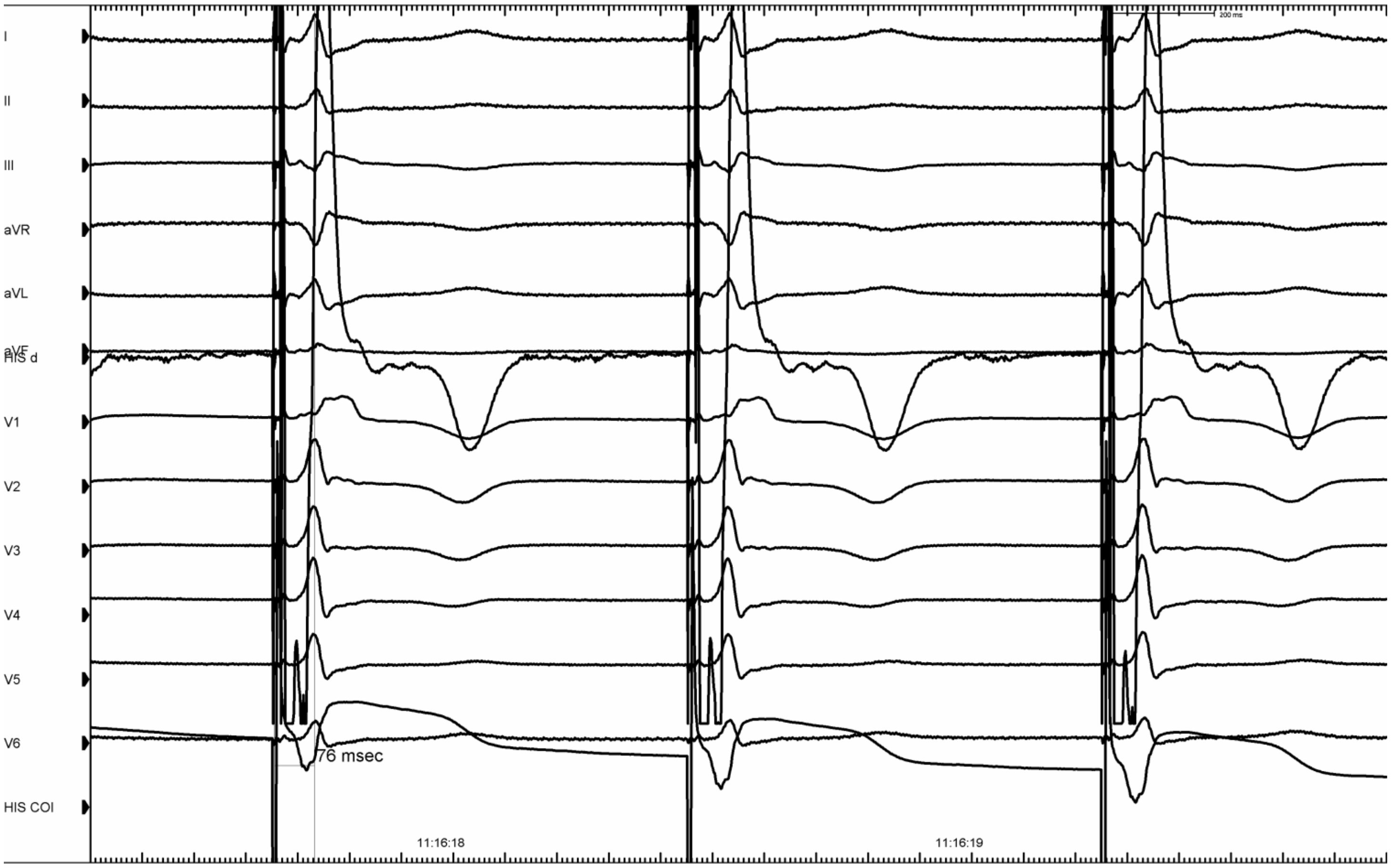

Transitions

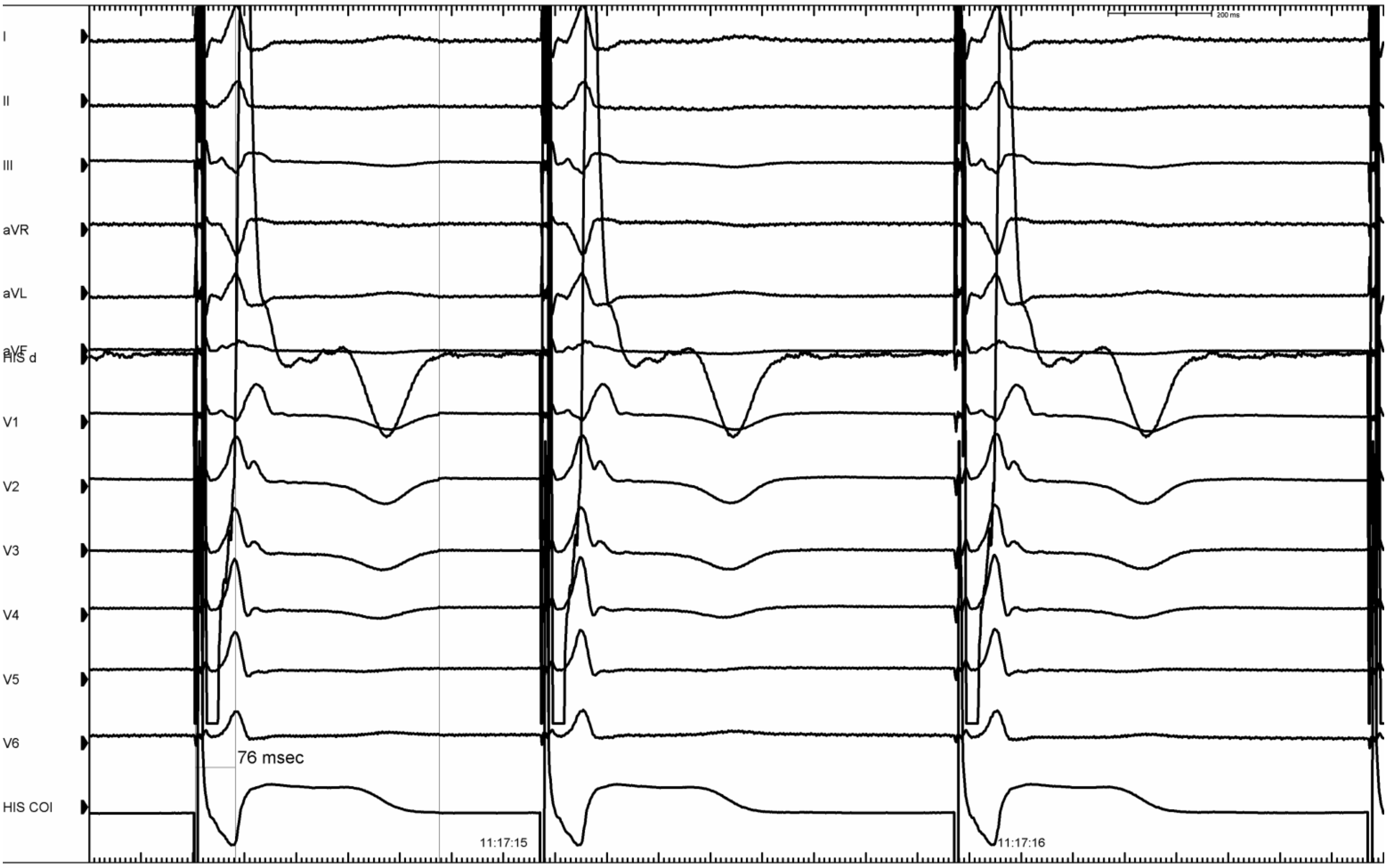

Patient 72:
Pre-ECG

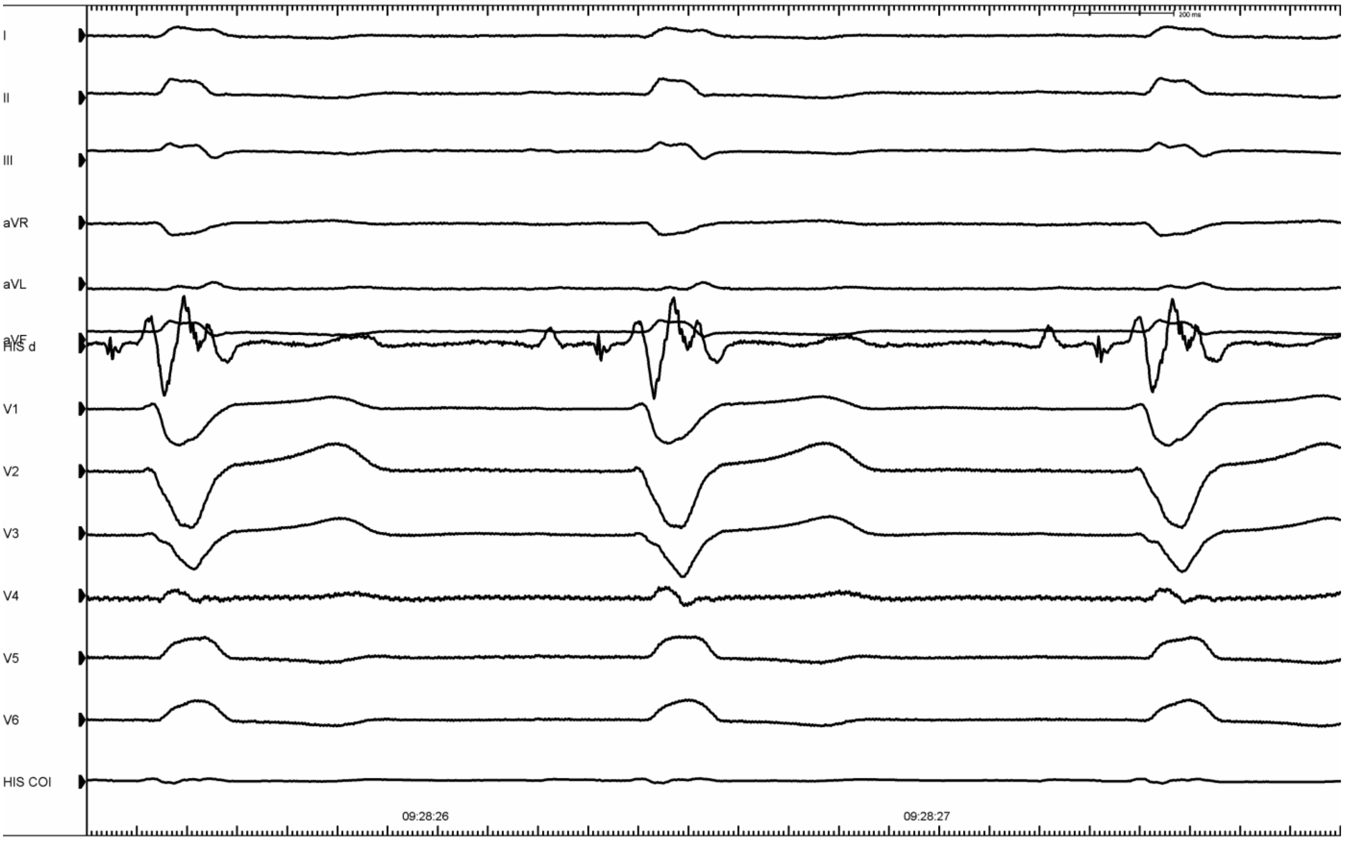

Post ECG

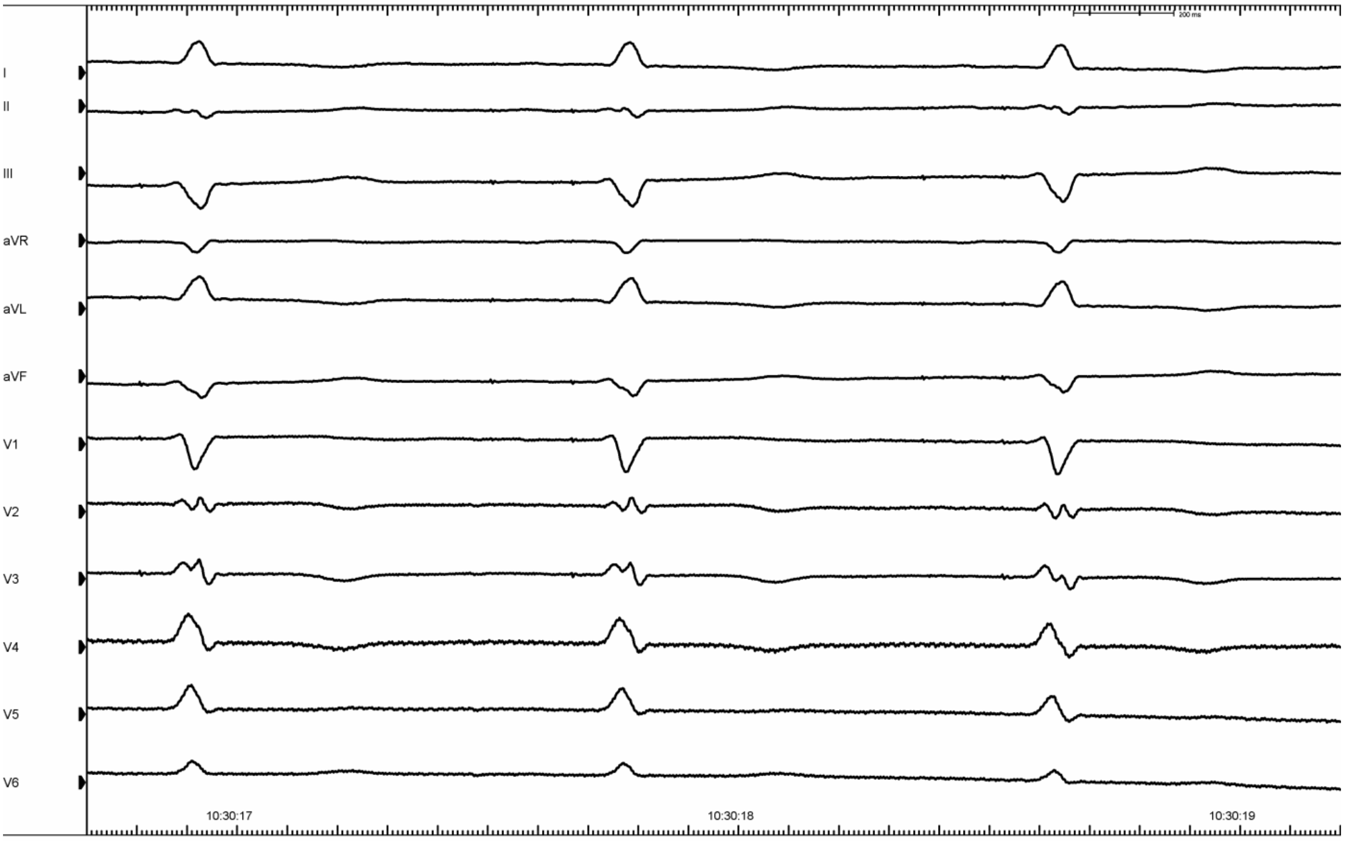

Patient 72:
Transitions

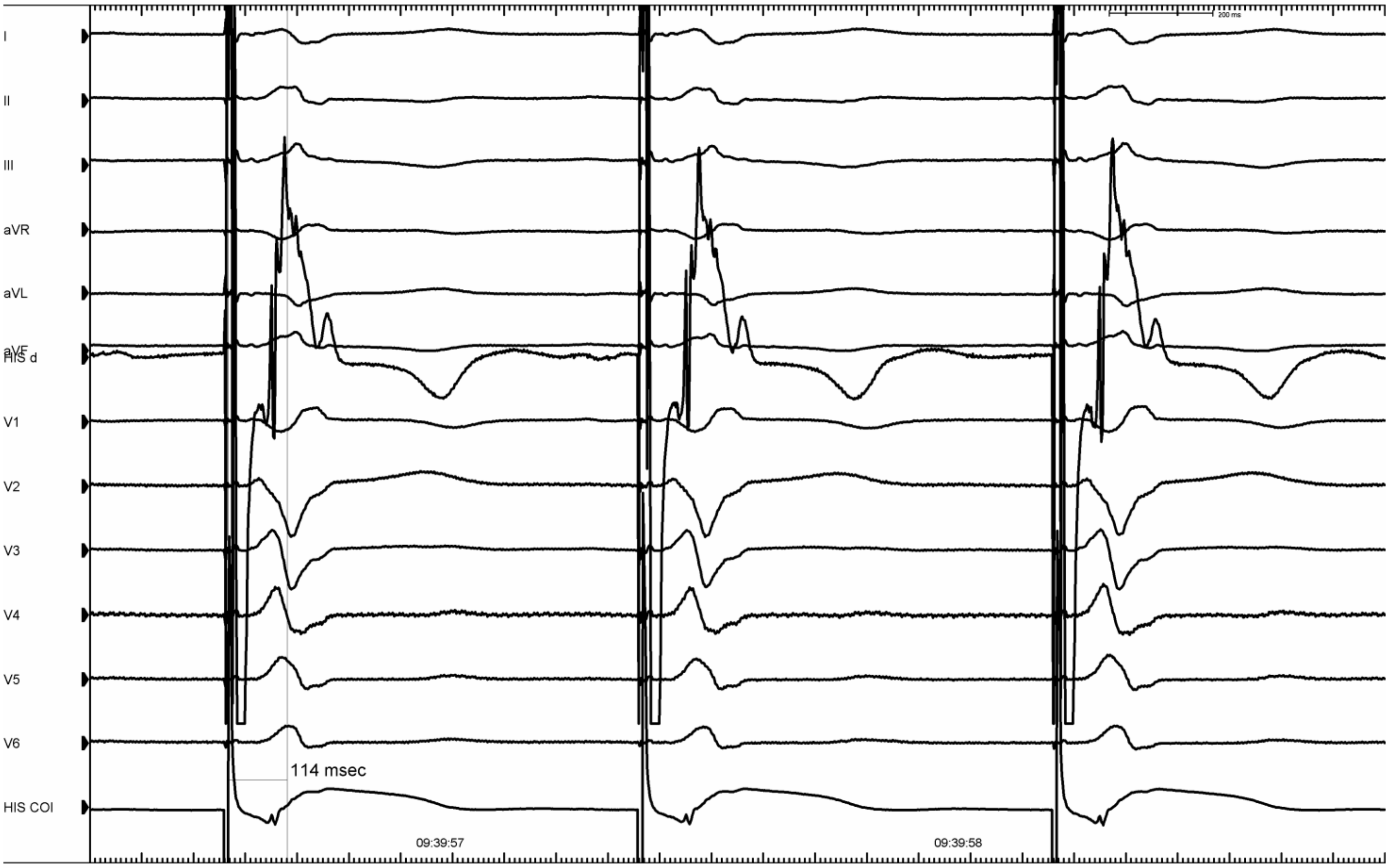

Transitions

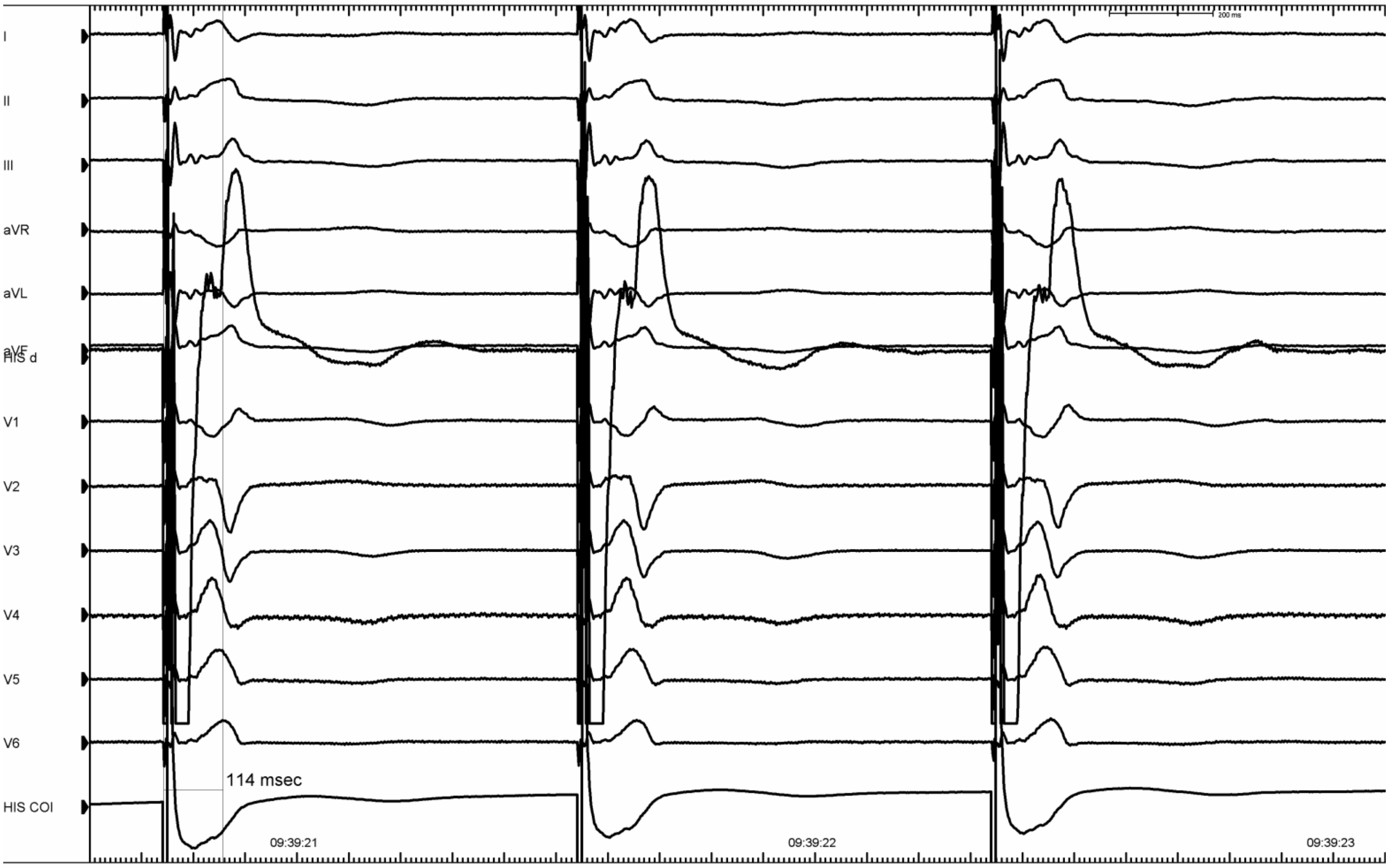

Patient 73:
Pre-ECG

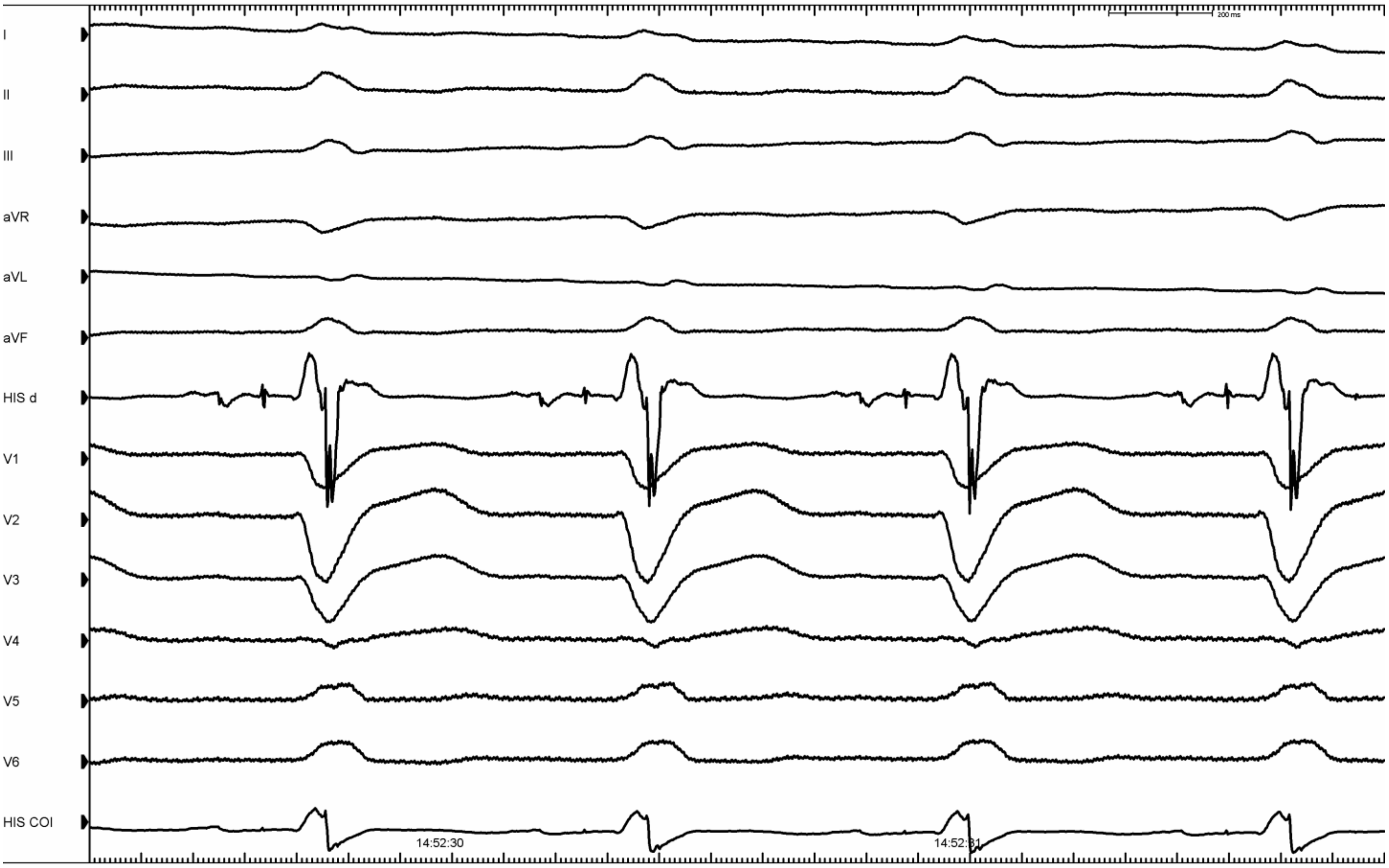

Post ECG

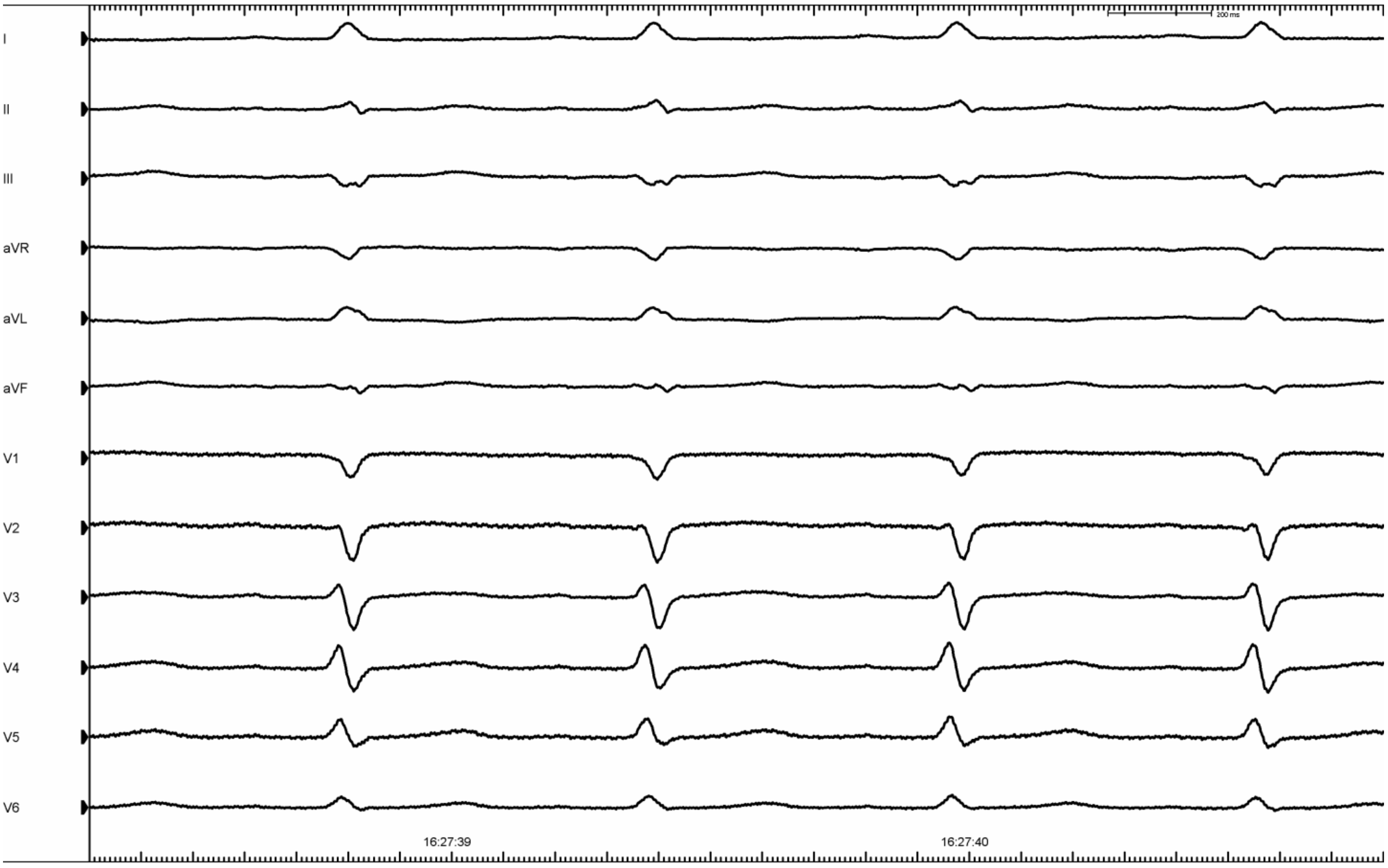

Patient 73:
Transitions

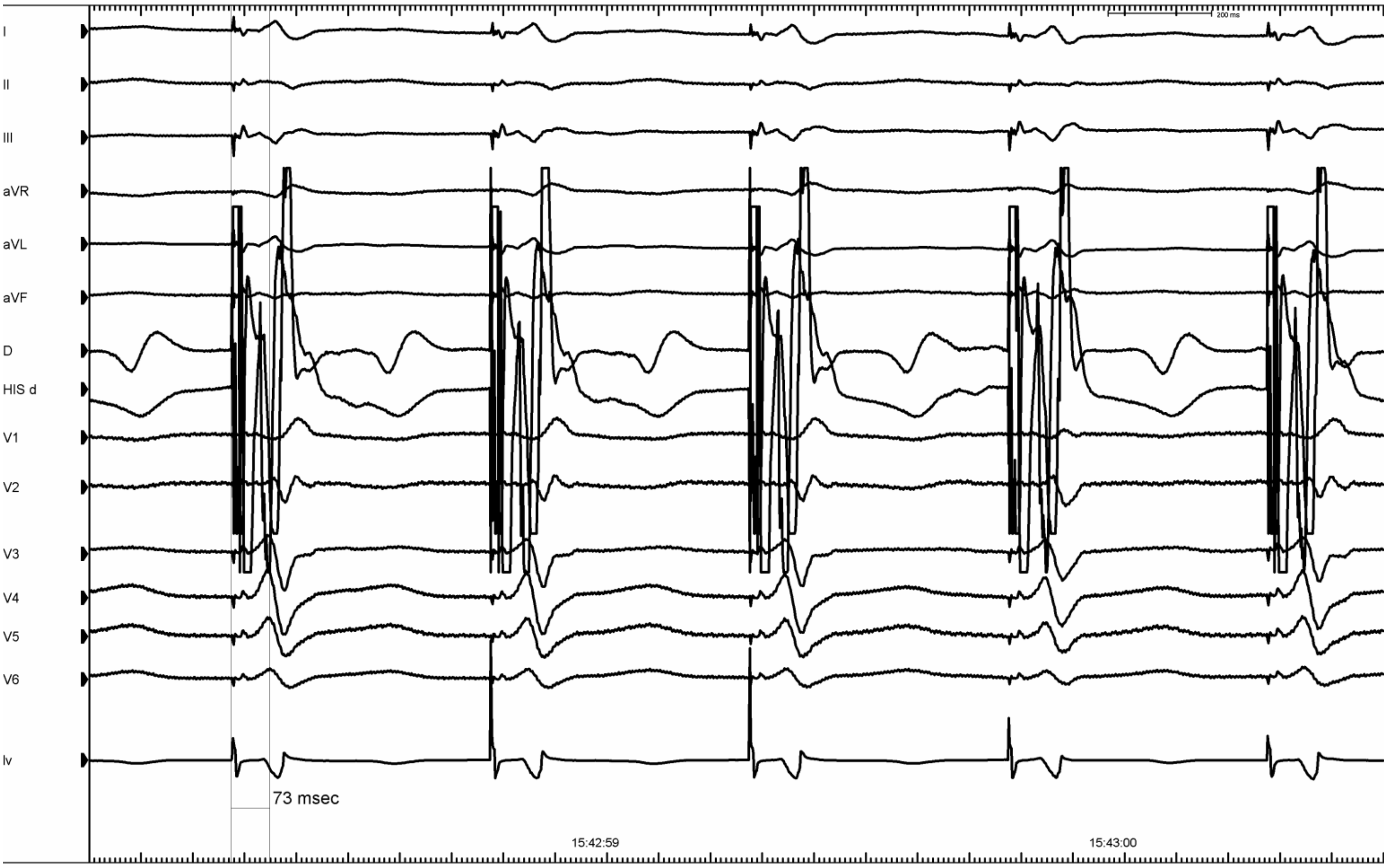

Transitions

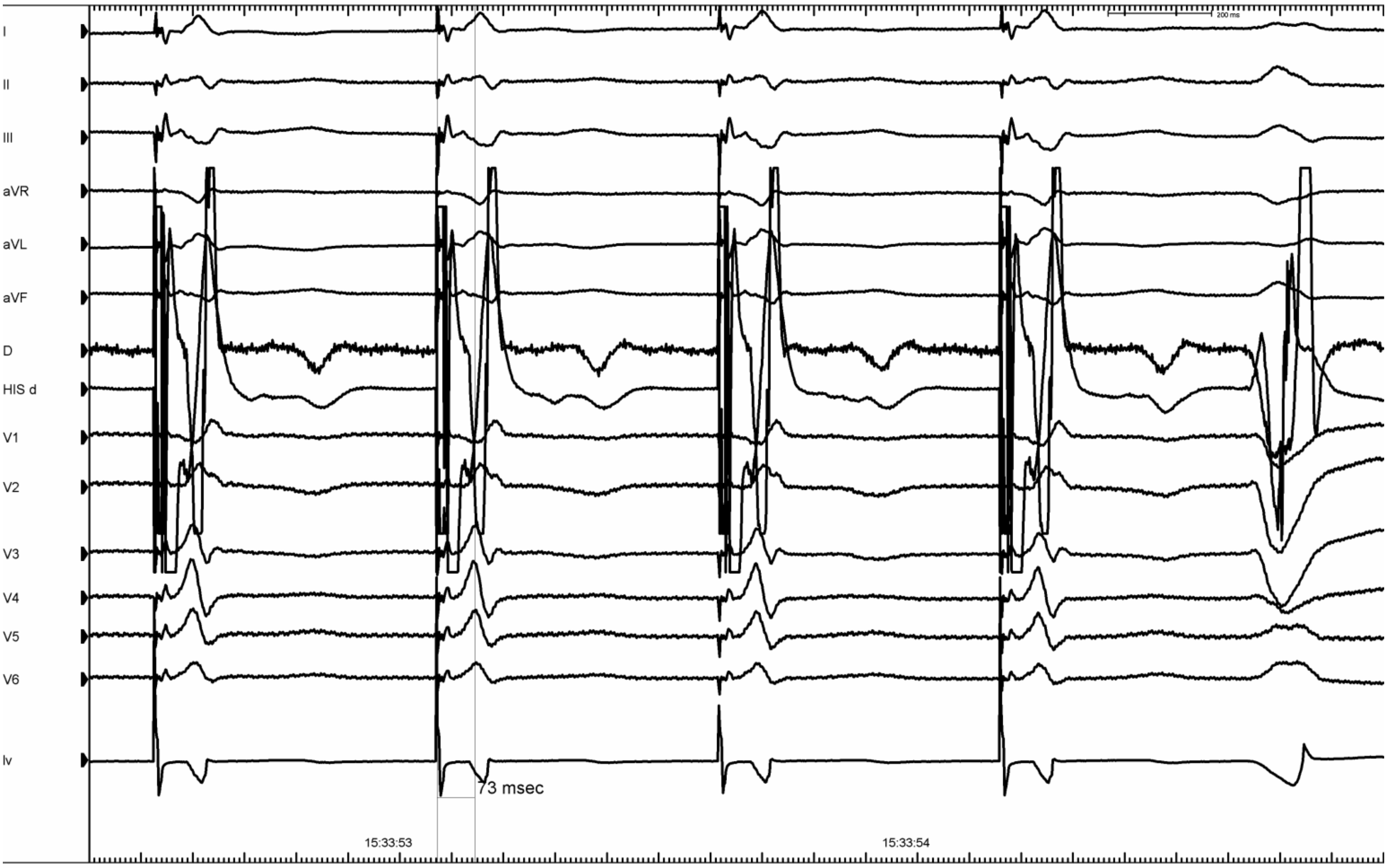

Patient 74:
Pre-ECG

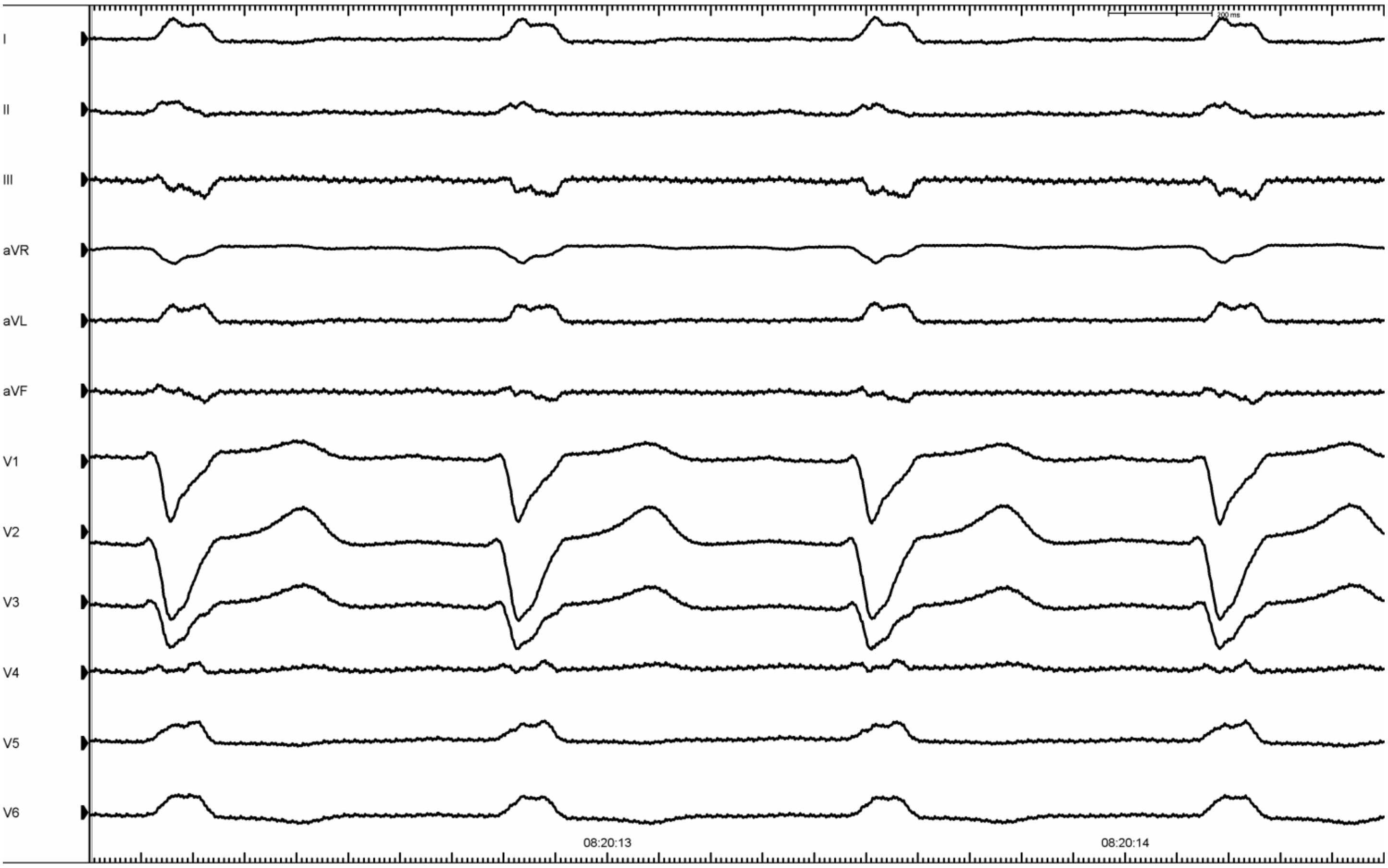

Post ECG

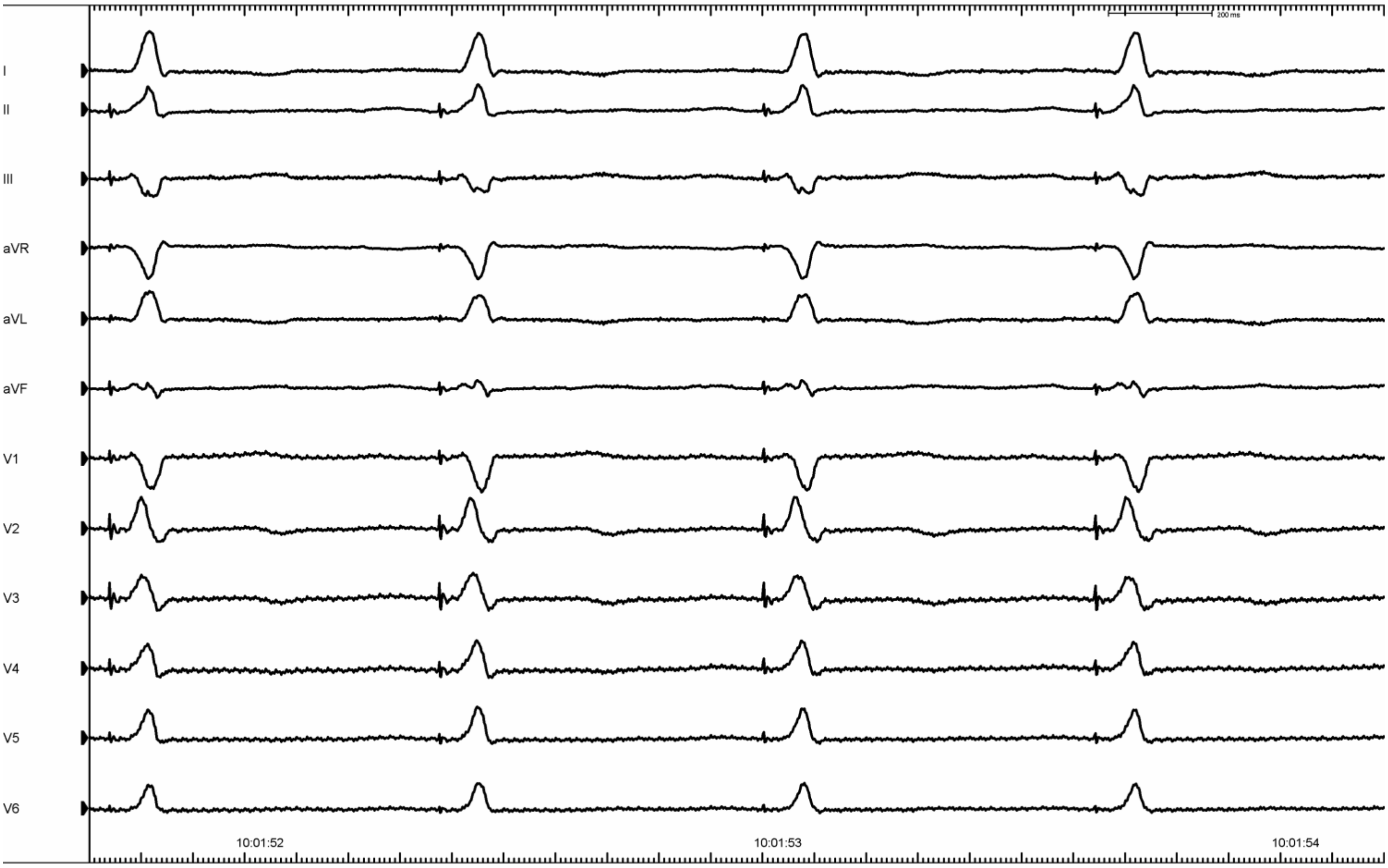

Patient 74:

Transitions

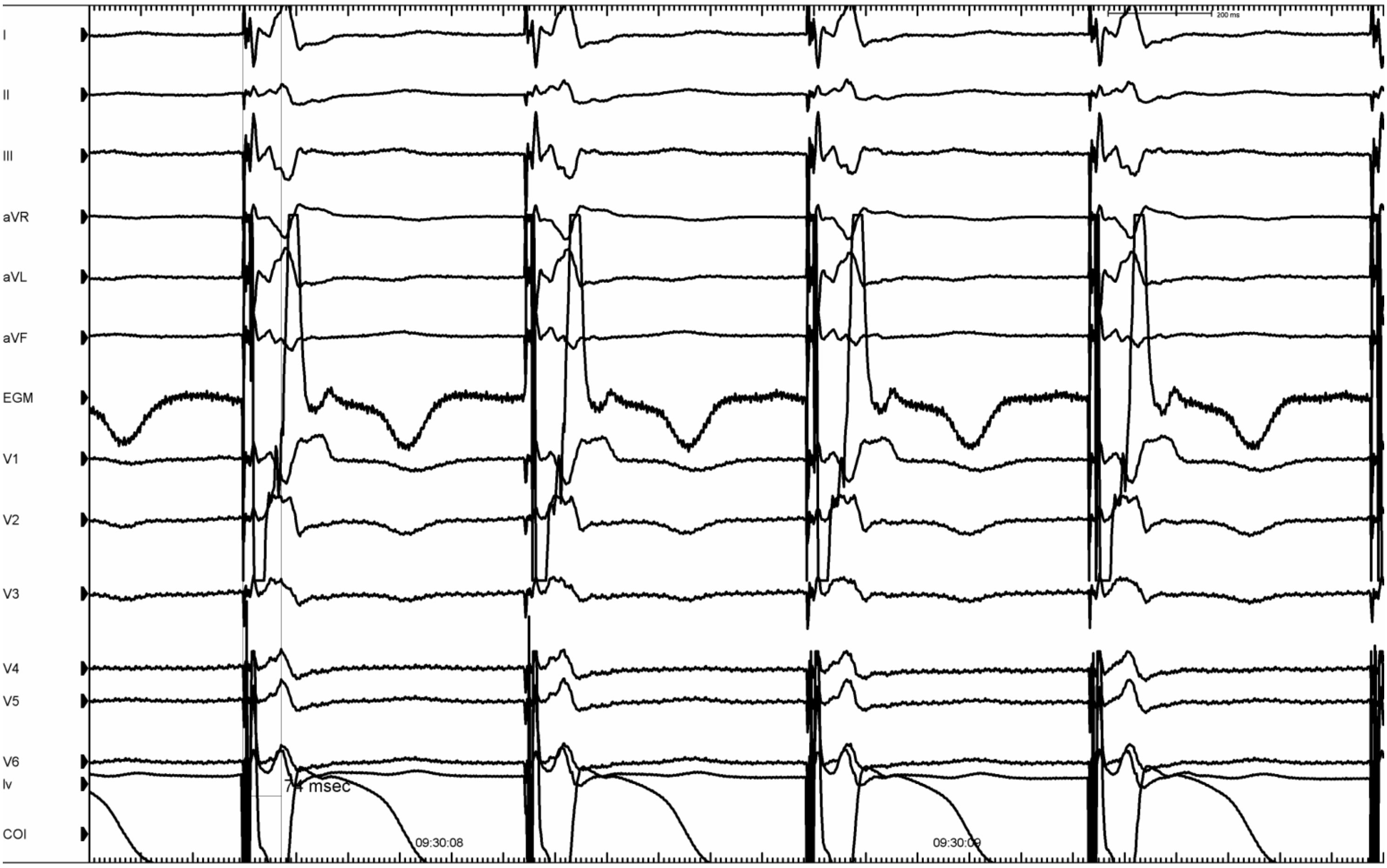

Transitions

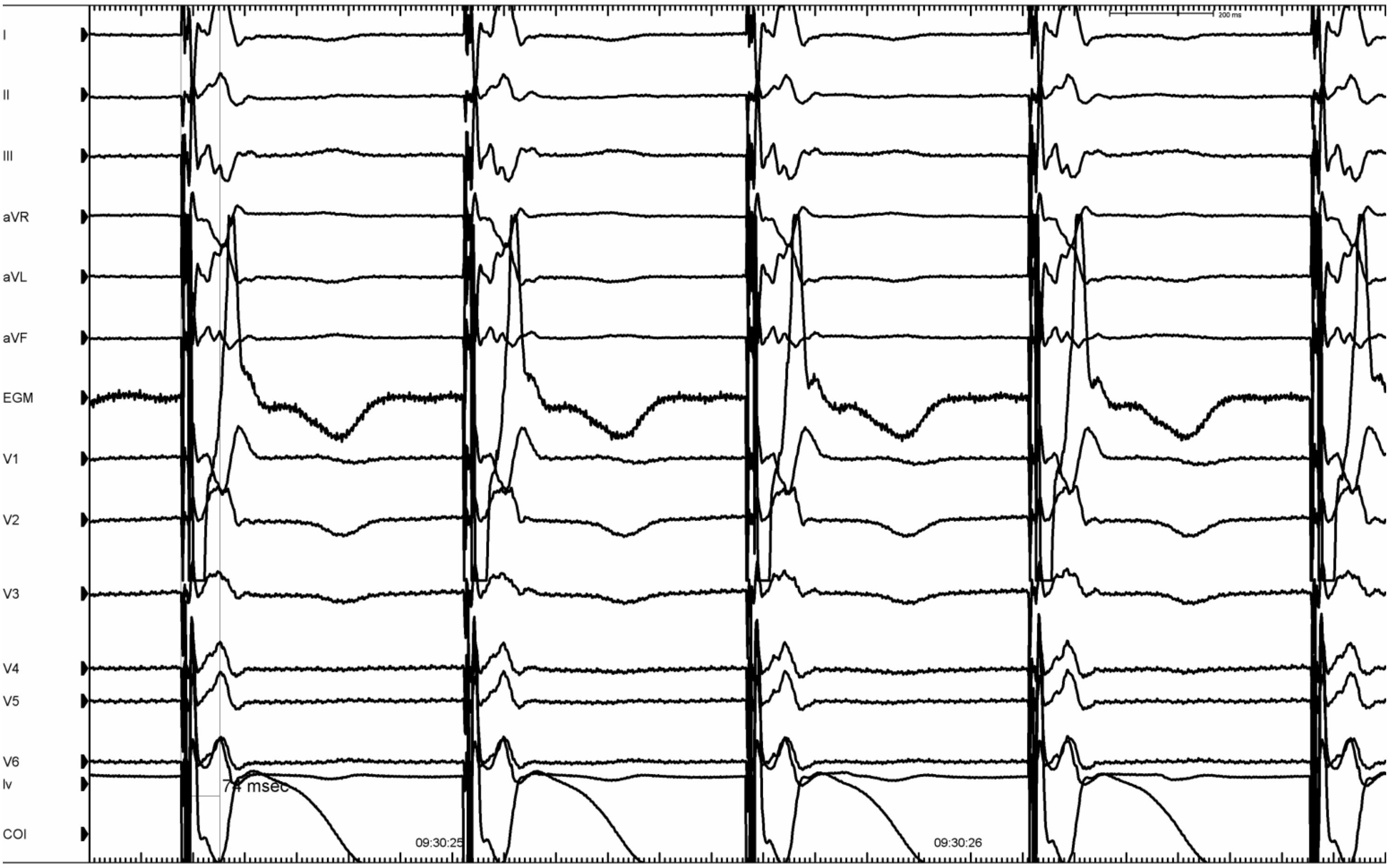

Patient 75:
Pre-ECG

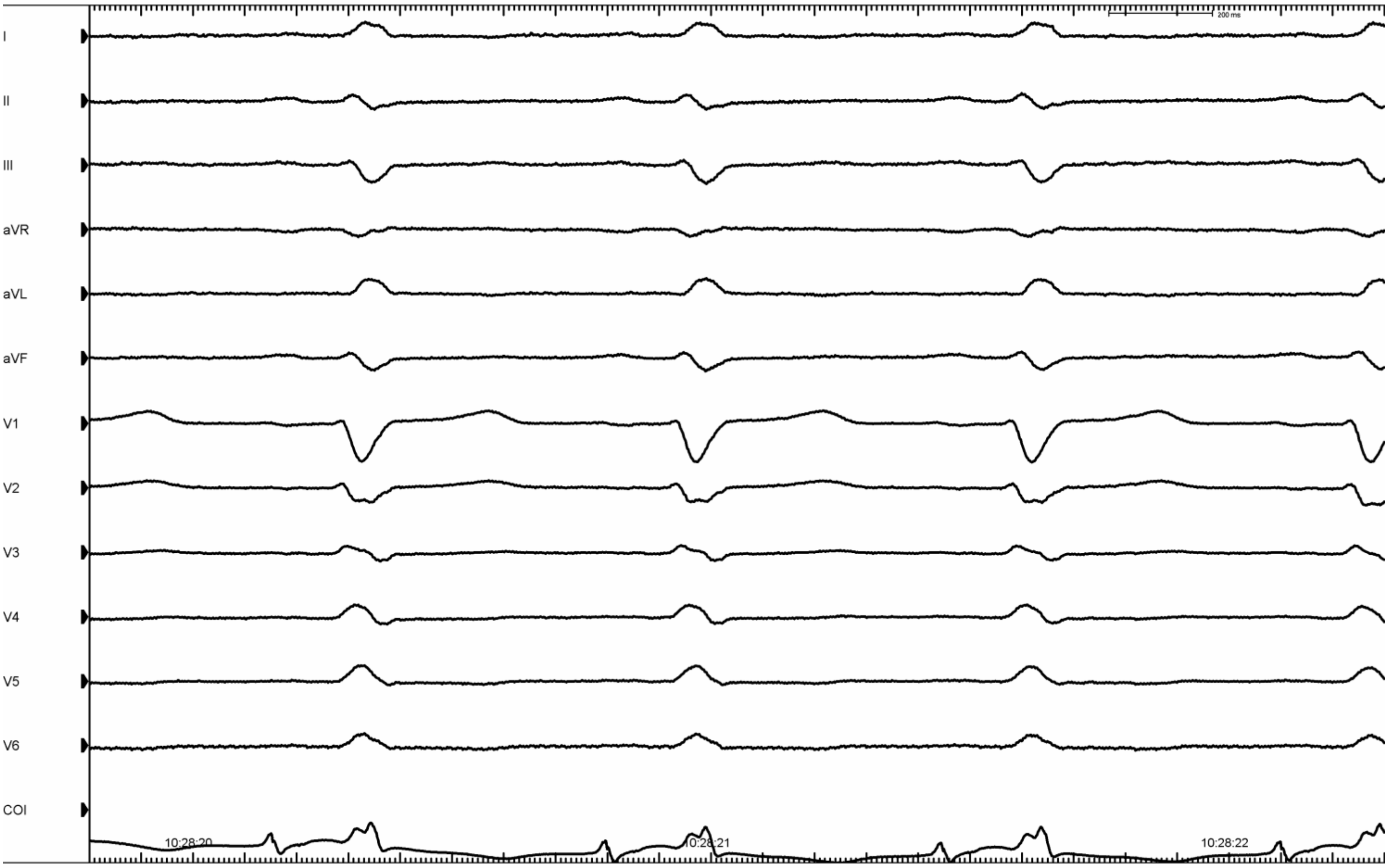

Post ECG

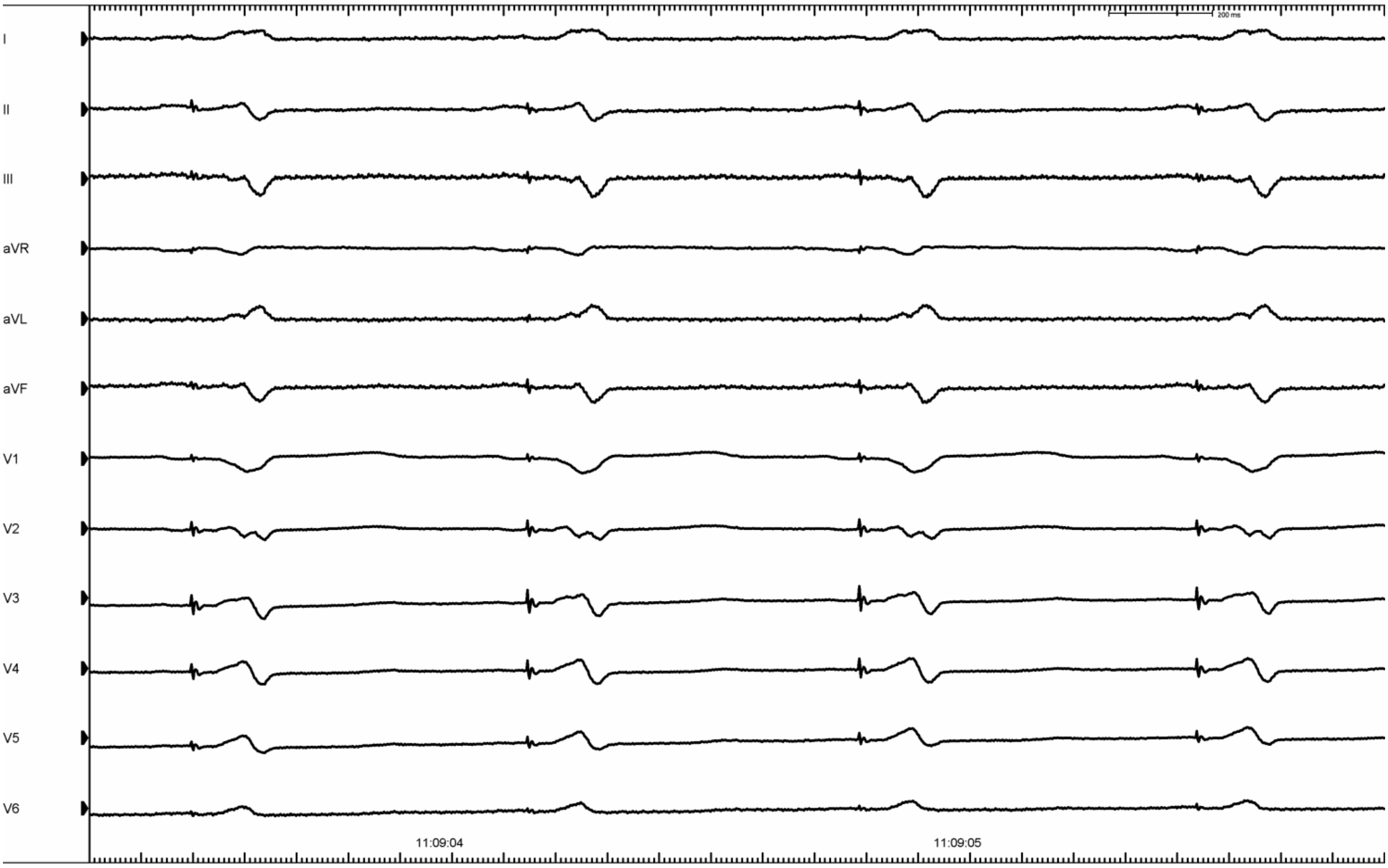

Patient 75:
Transitions

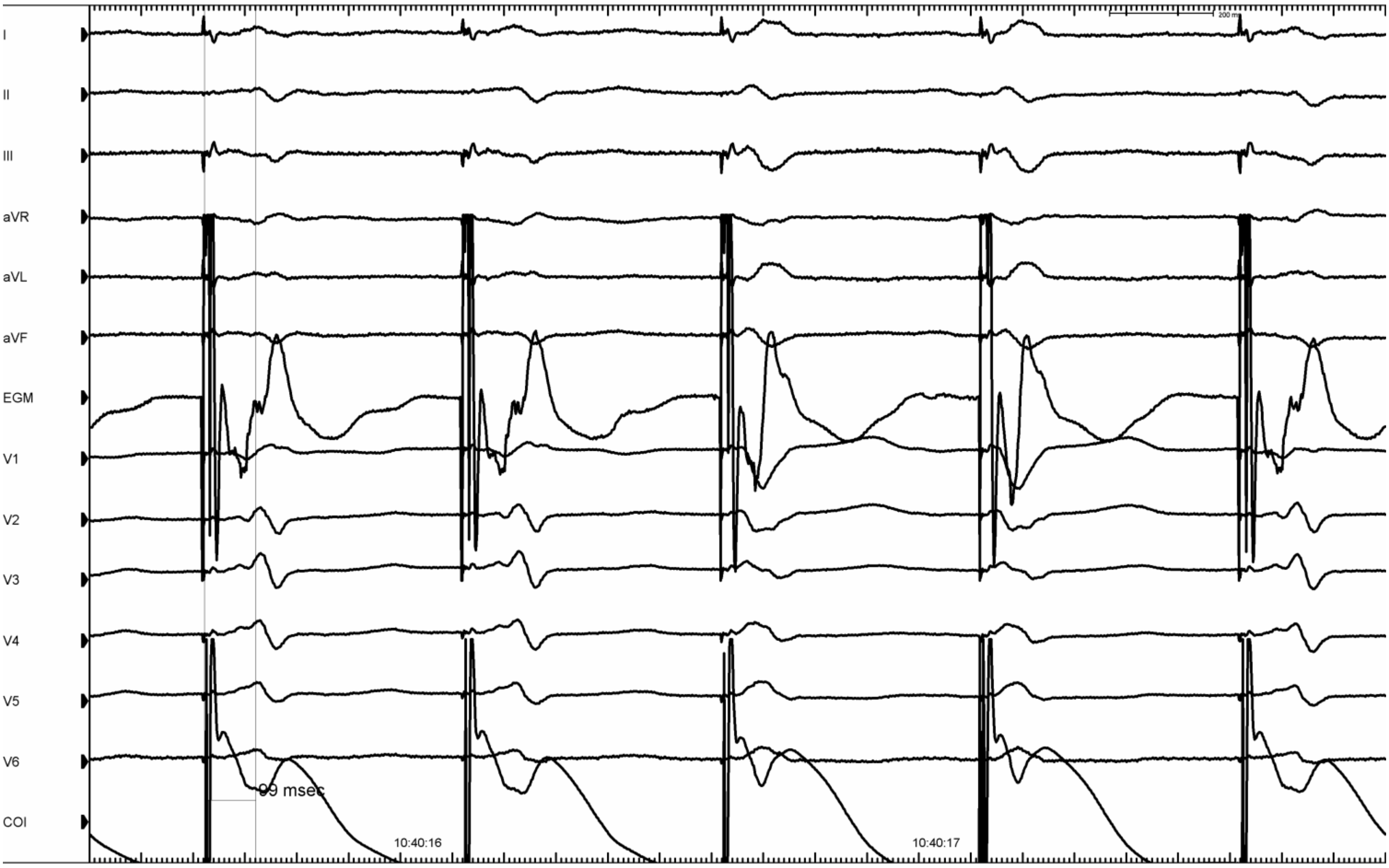

Transitions

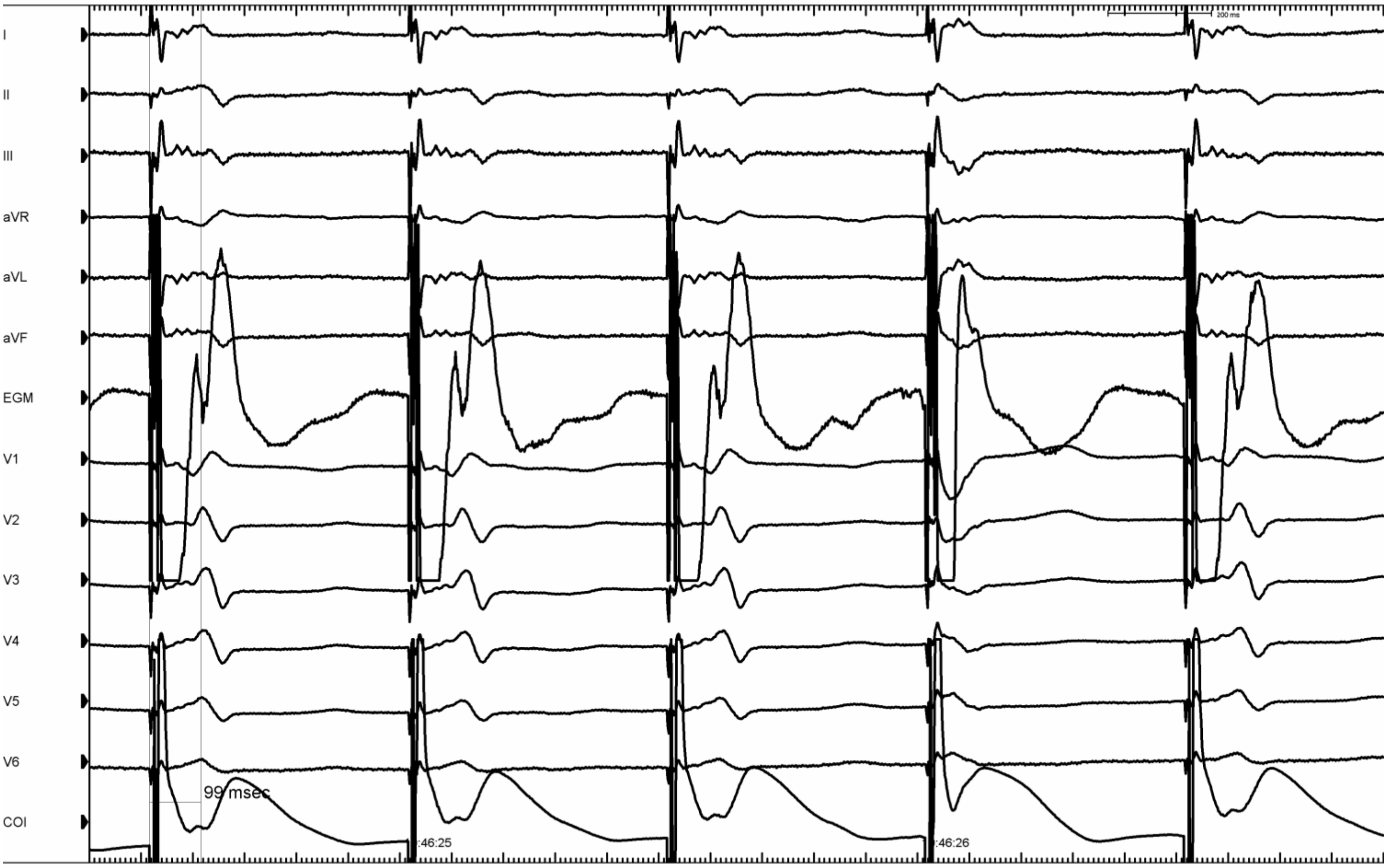

Patient 76:
Pre-ECG

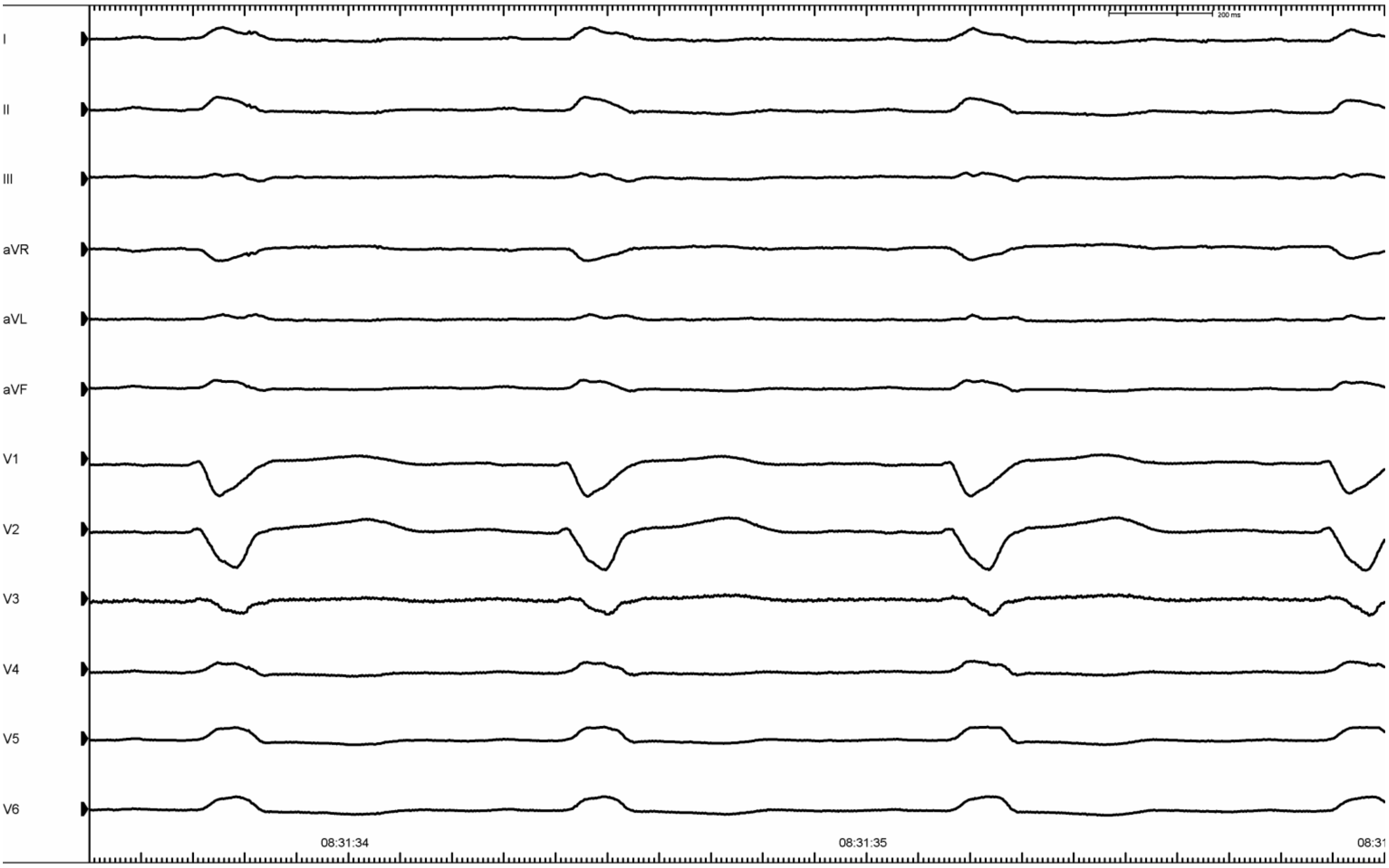

Post ECG

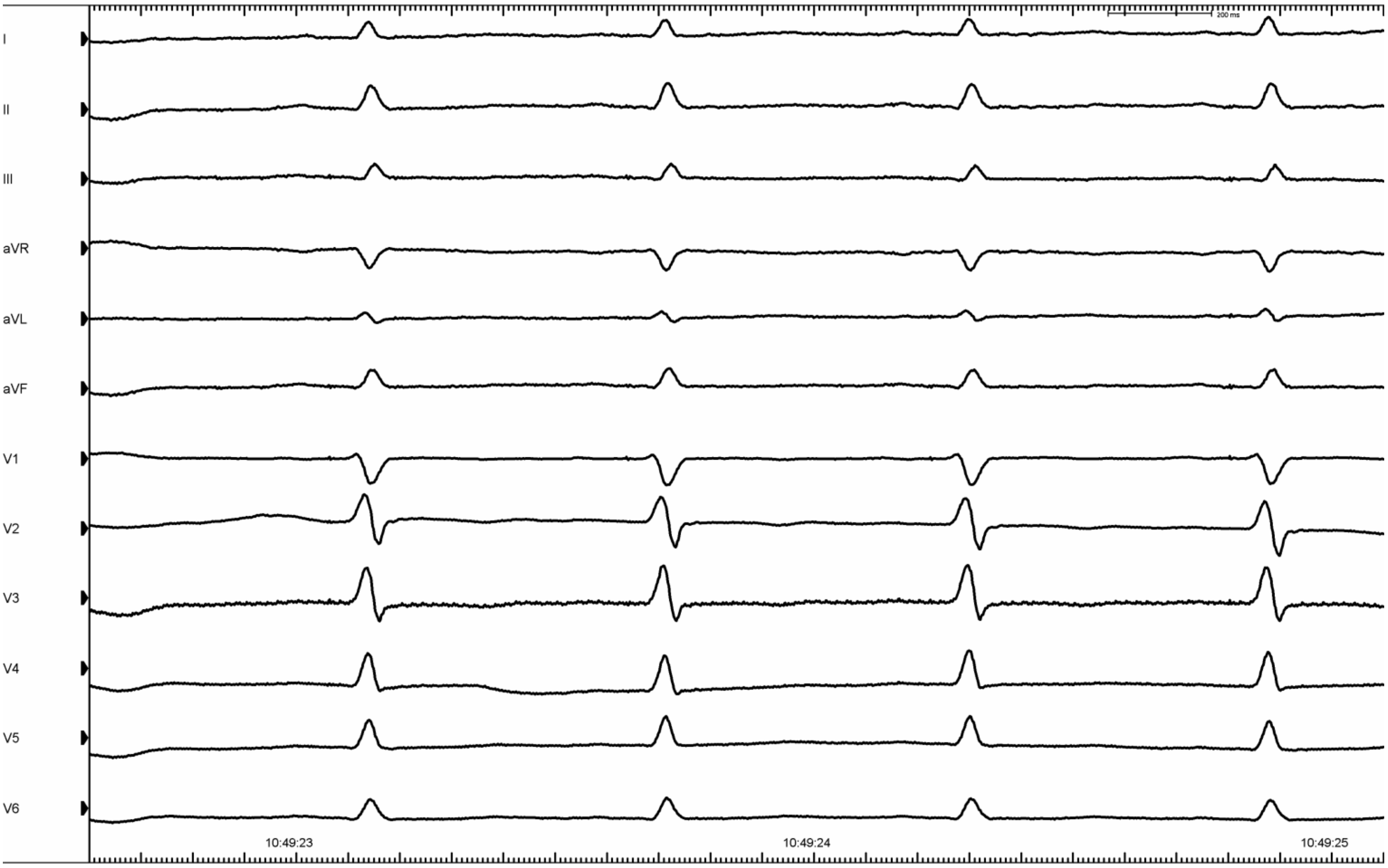

Patient 76:
Transitions

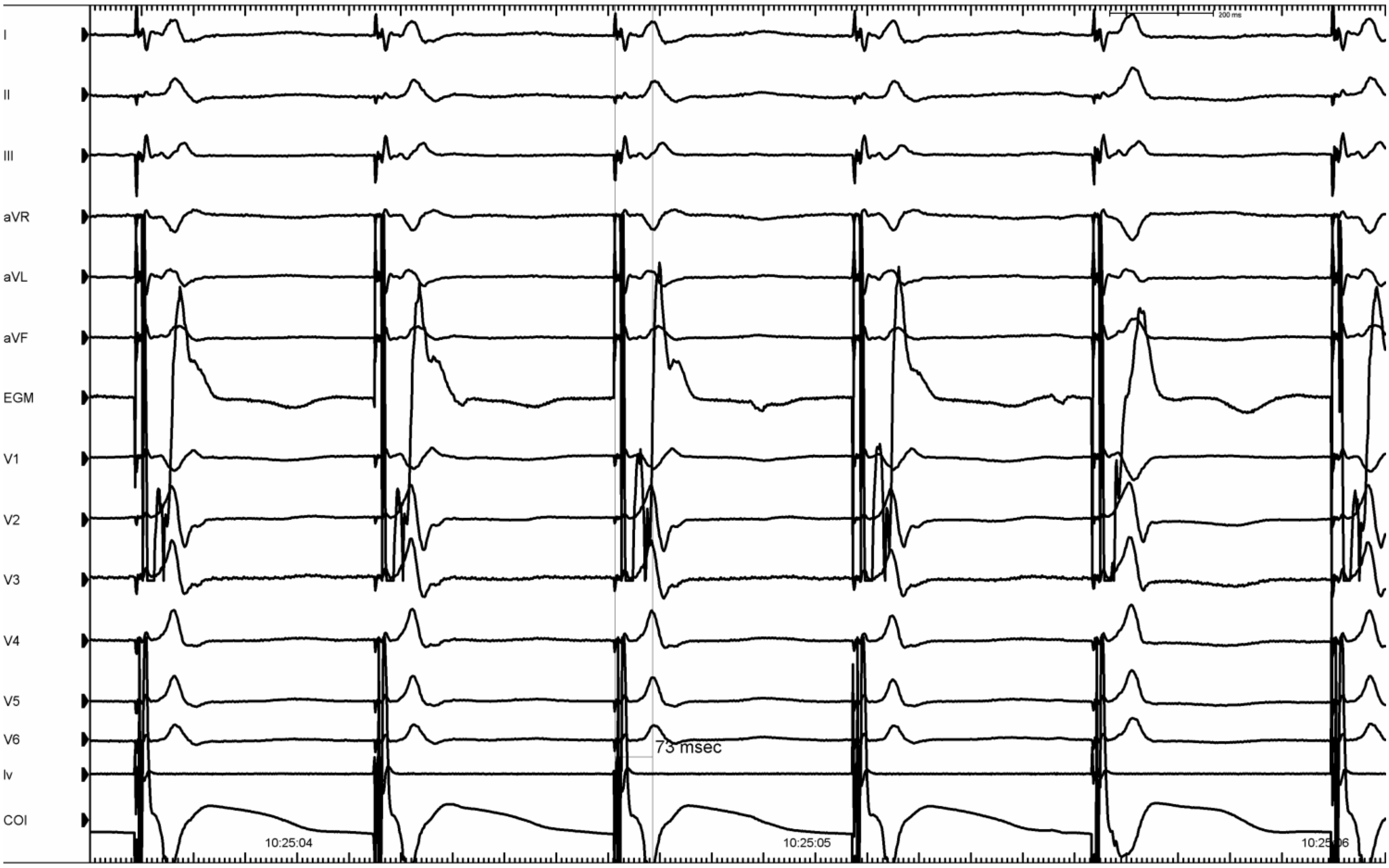

Transitions

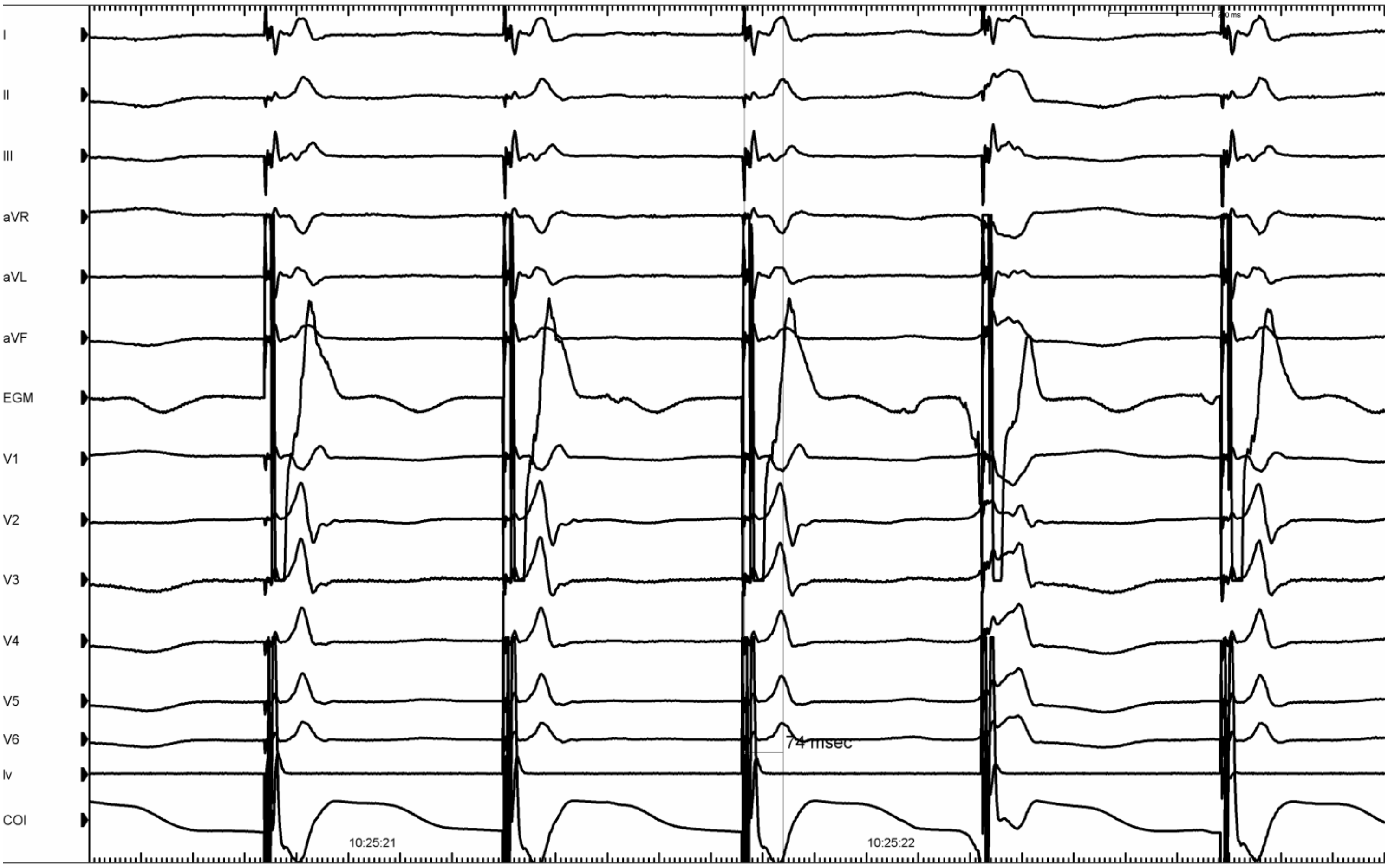

Patient 77:
Pre-ECG

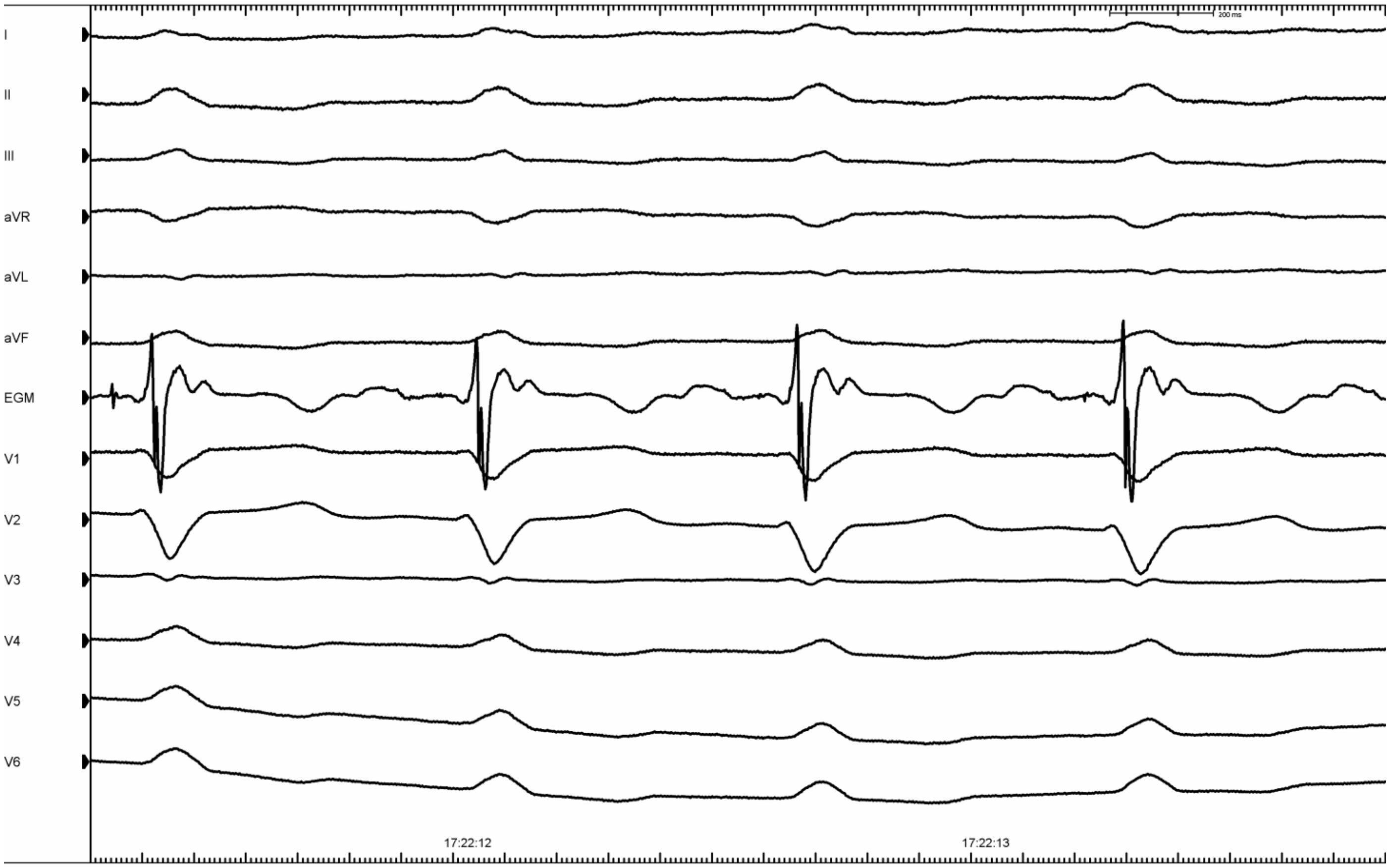

Post ECG

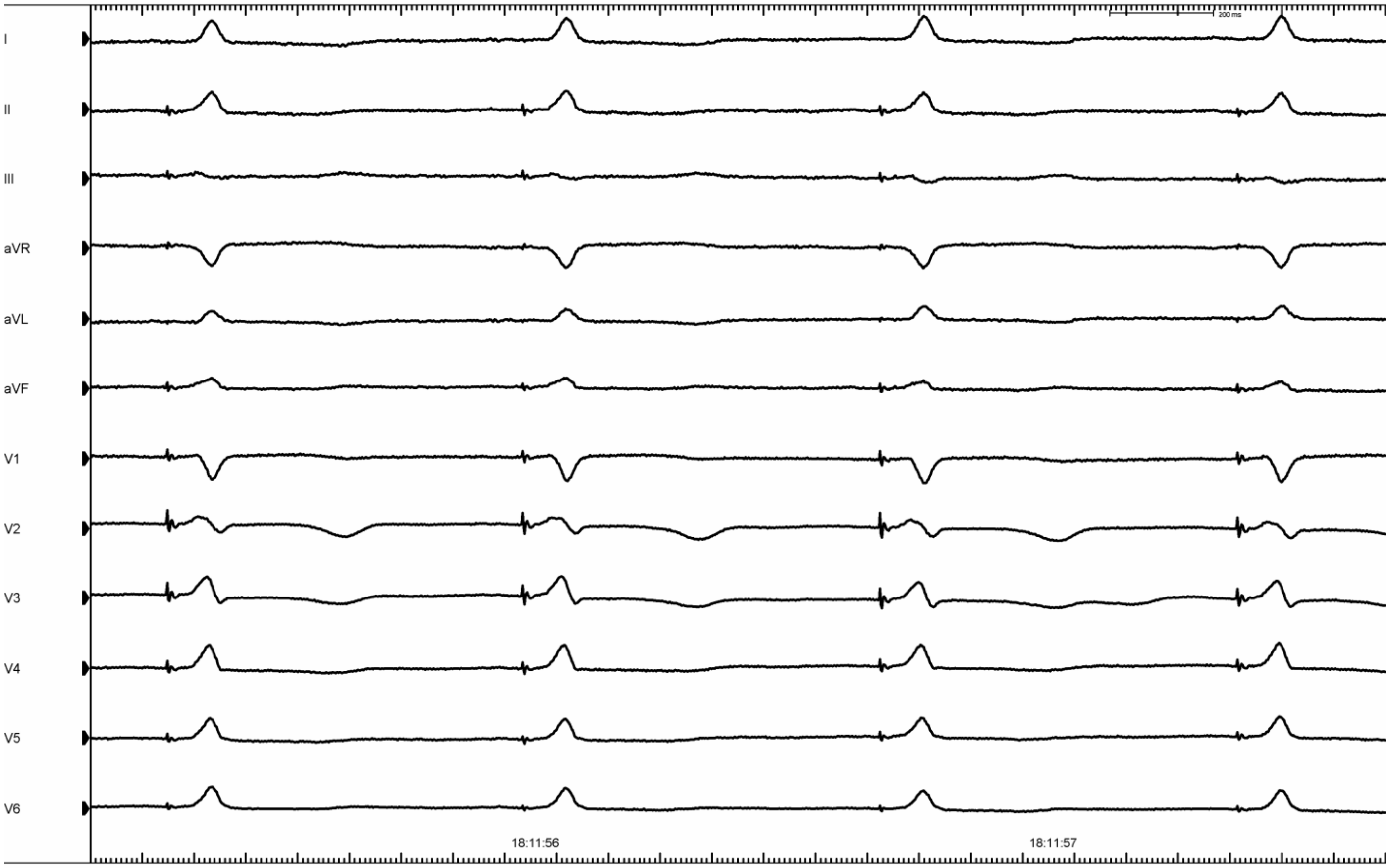

Patient 77:
Transitions

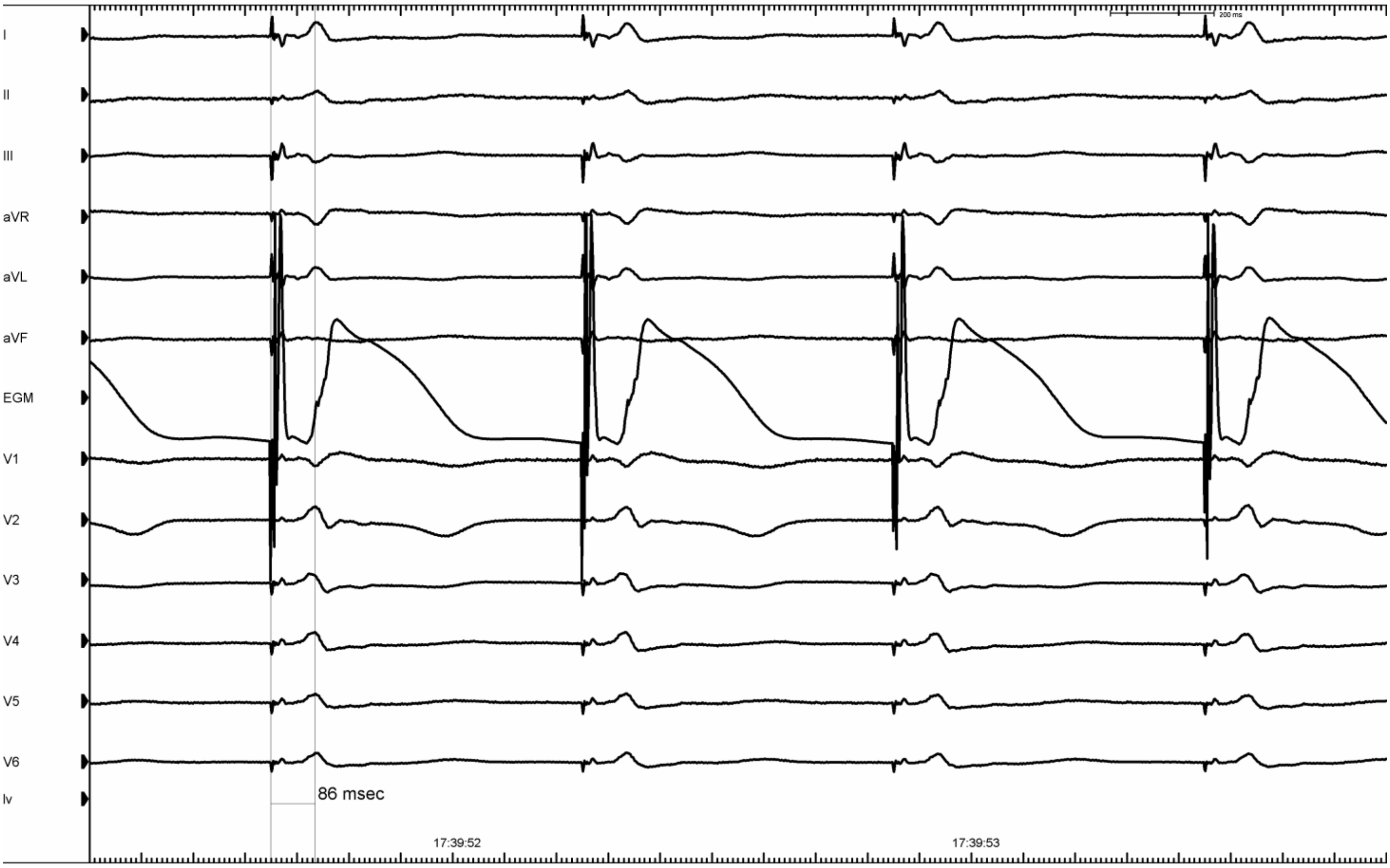

Transitions

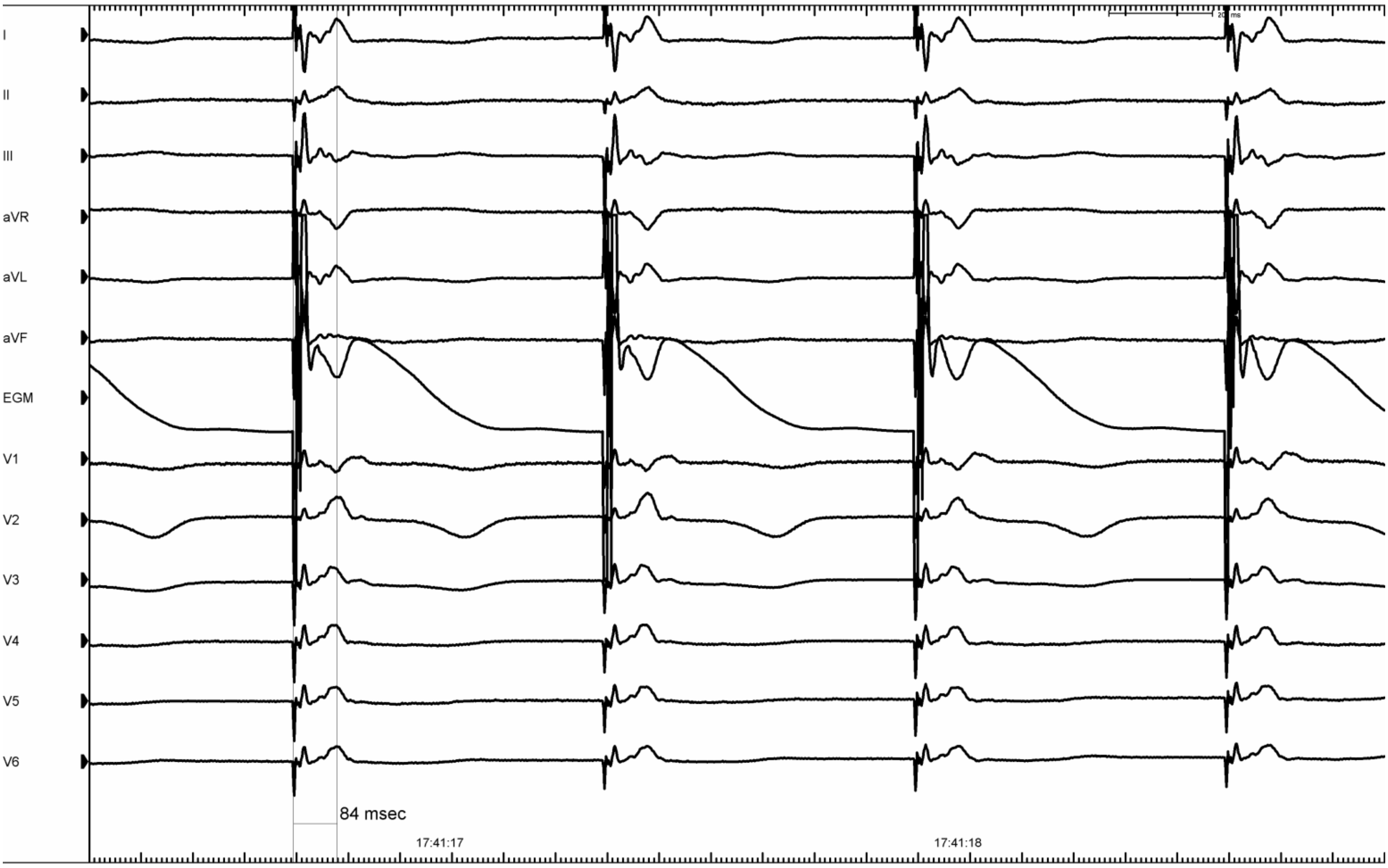

Patient 78:
Pre-ECG

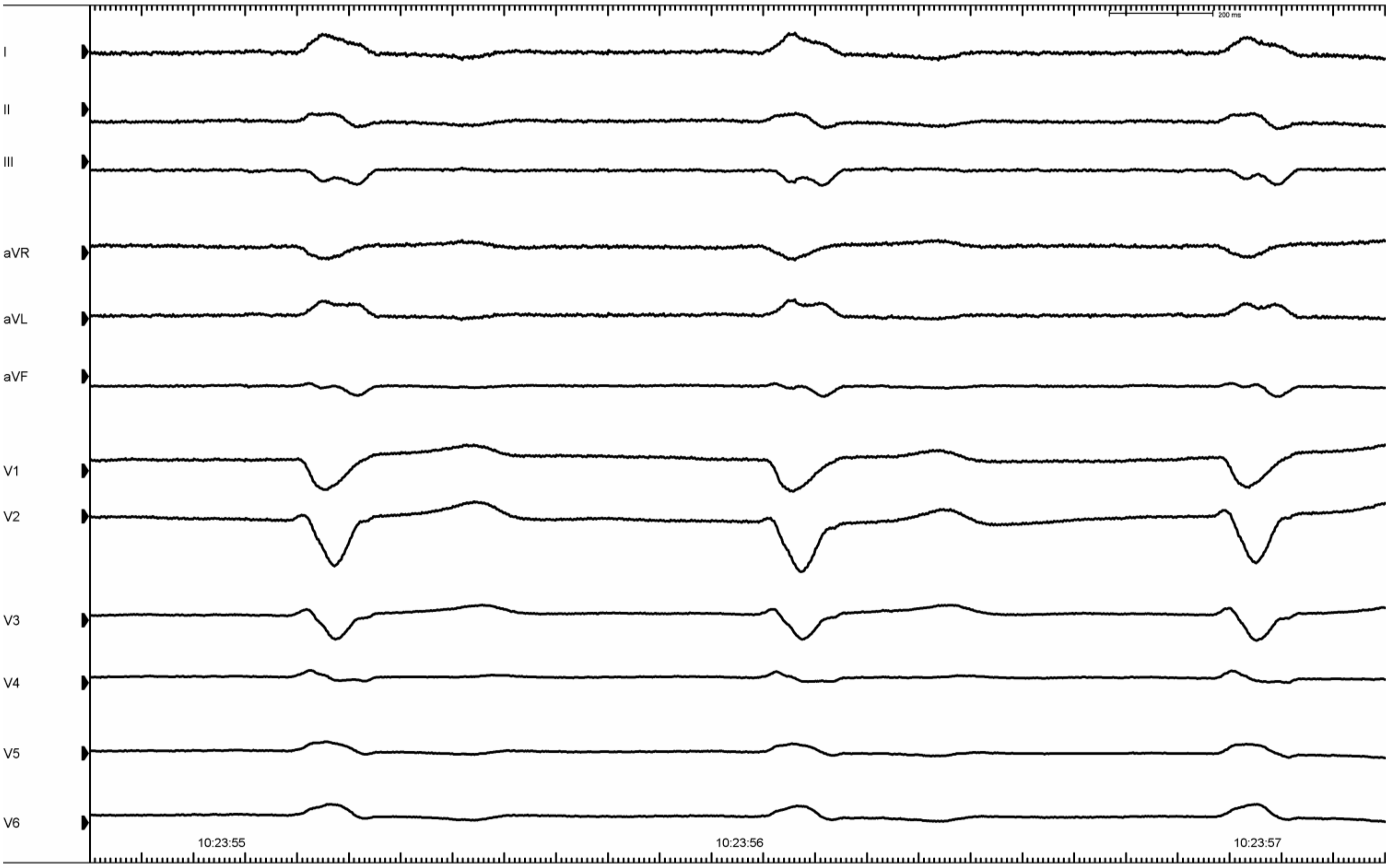

Post ECG

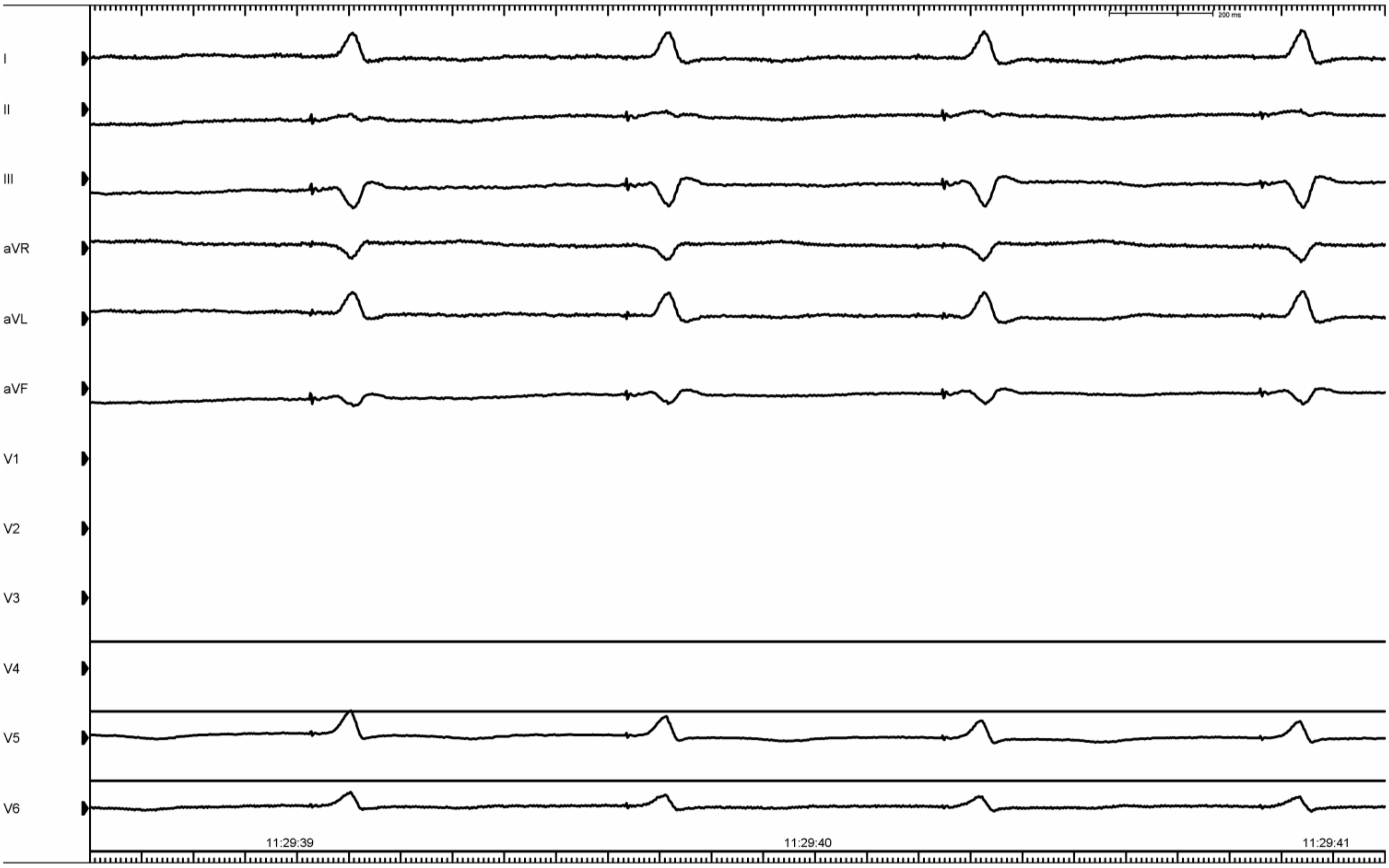

Patient 78:

Transitions

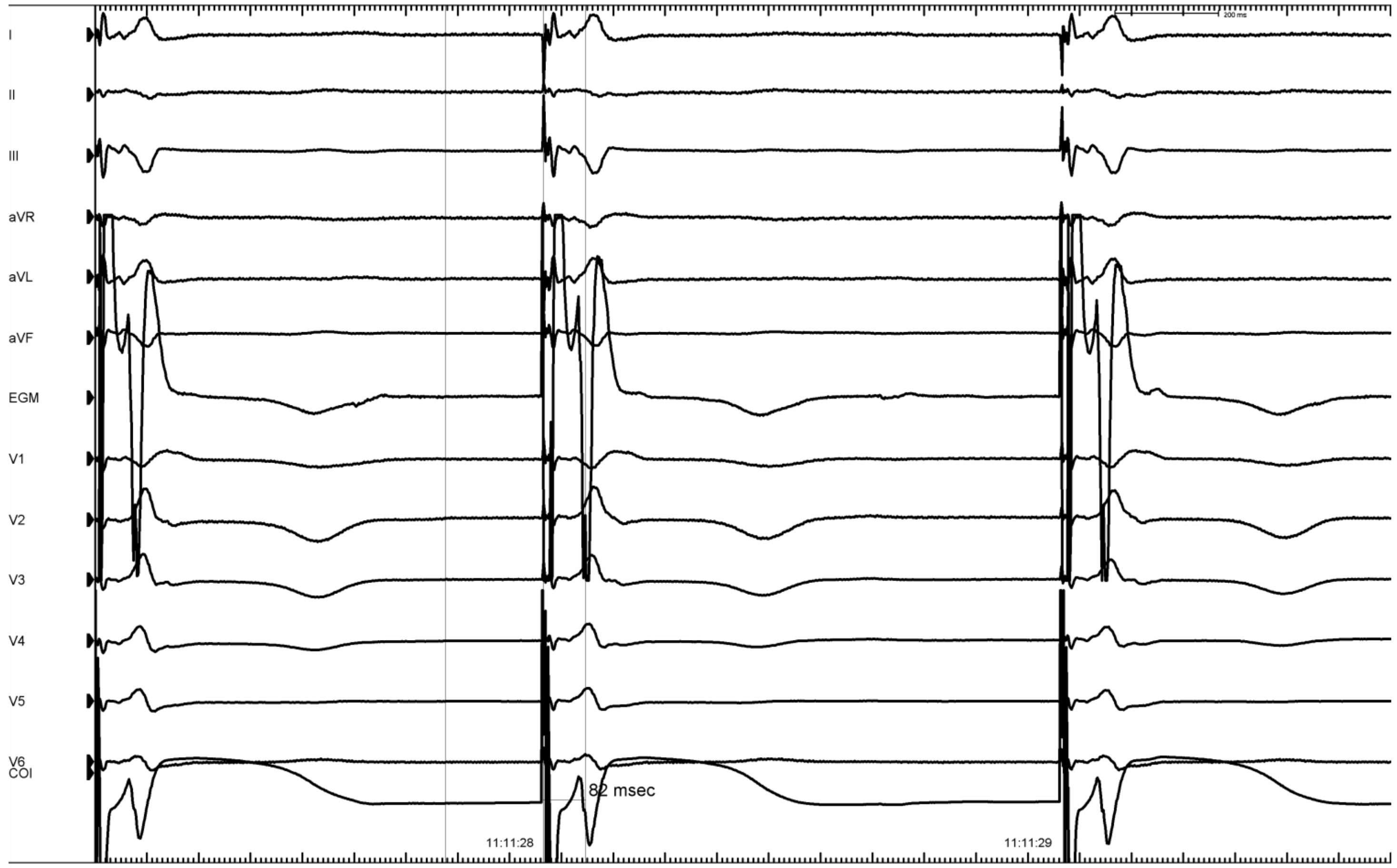

Transitions

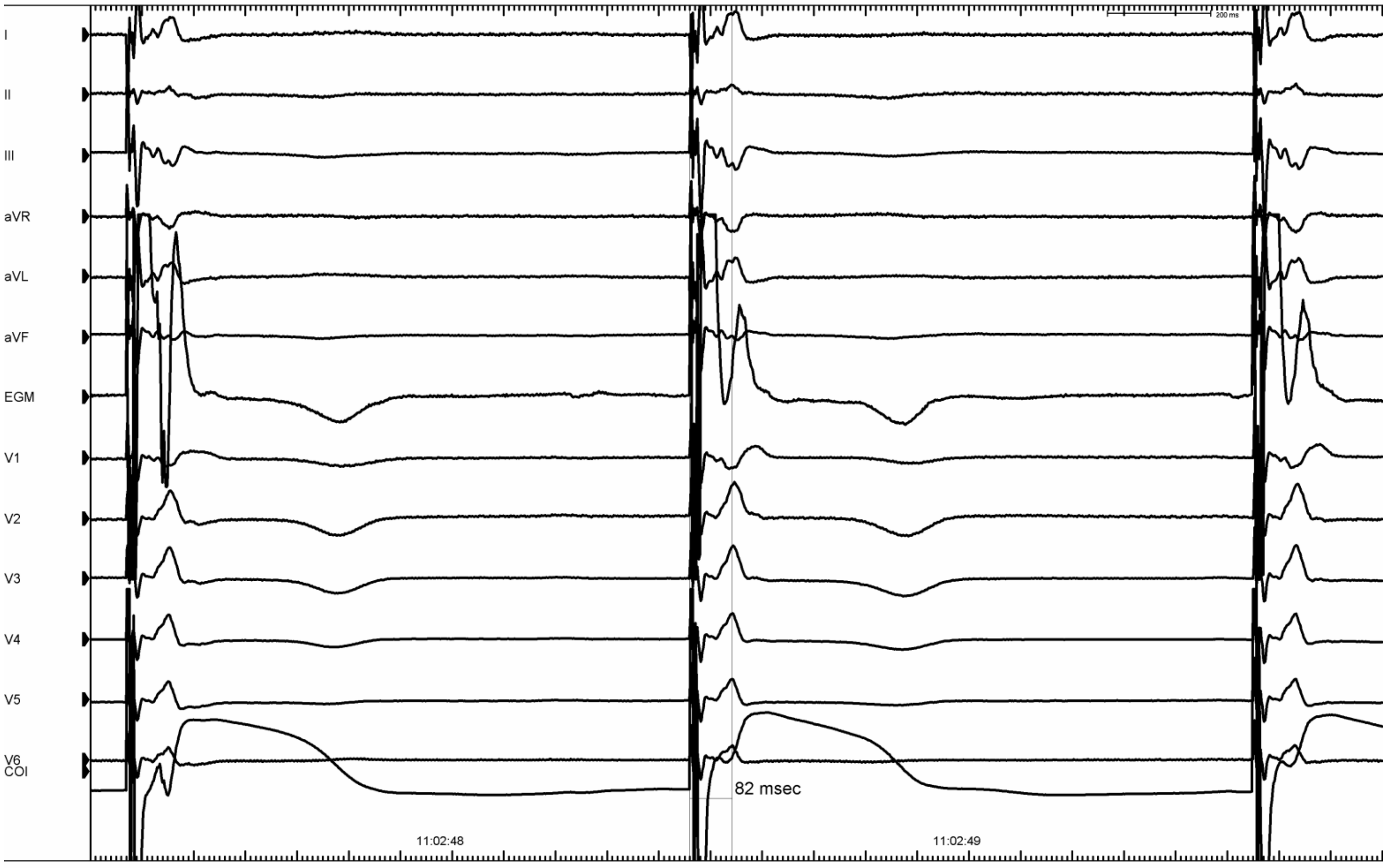

Patient 79:
Pre-ECG

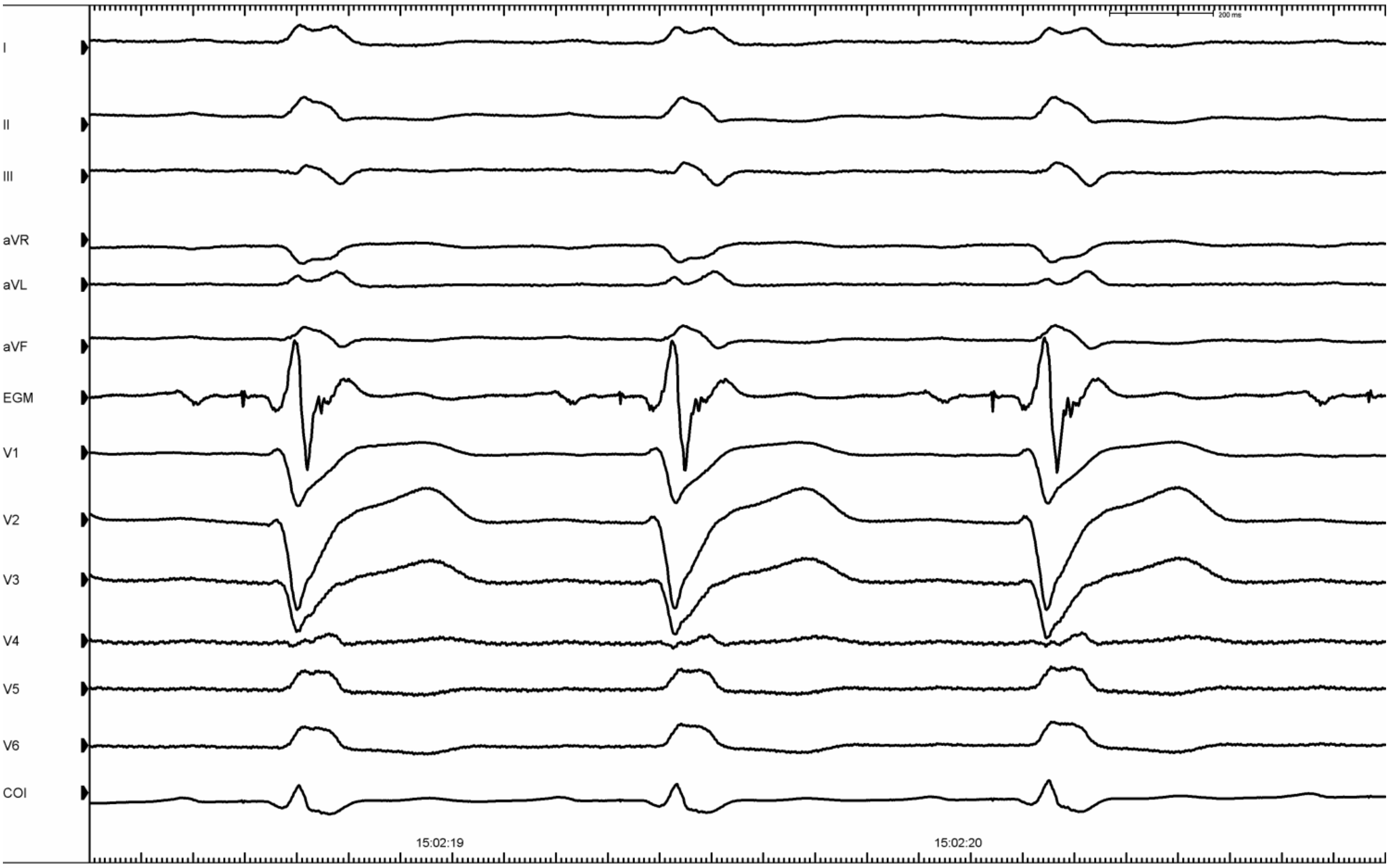

Post ECG

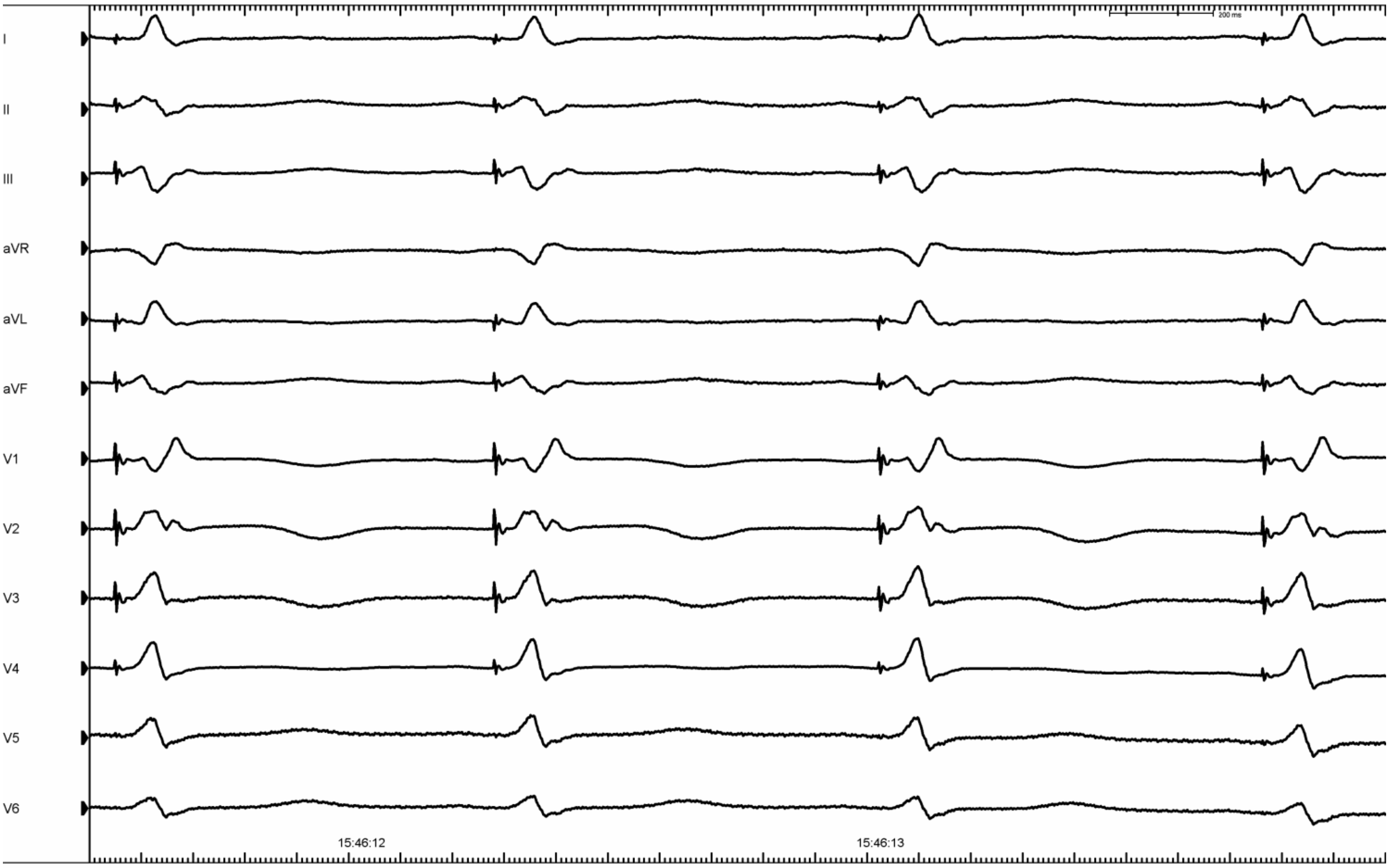

Patient 79:
Transitions

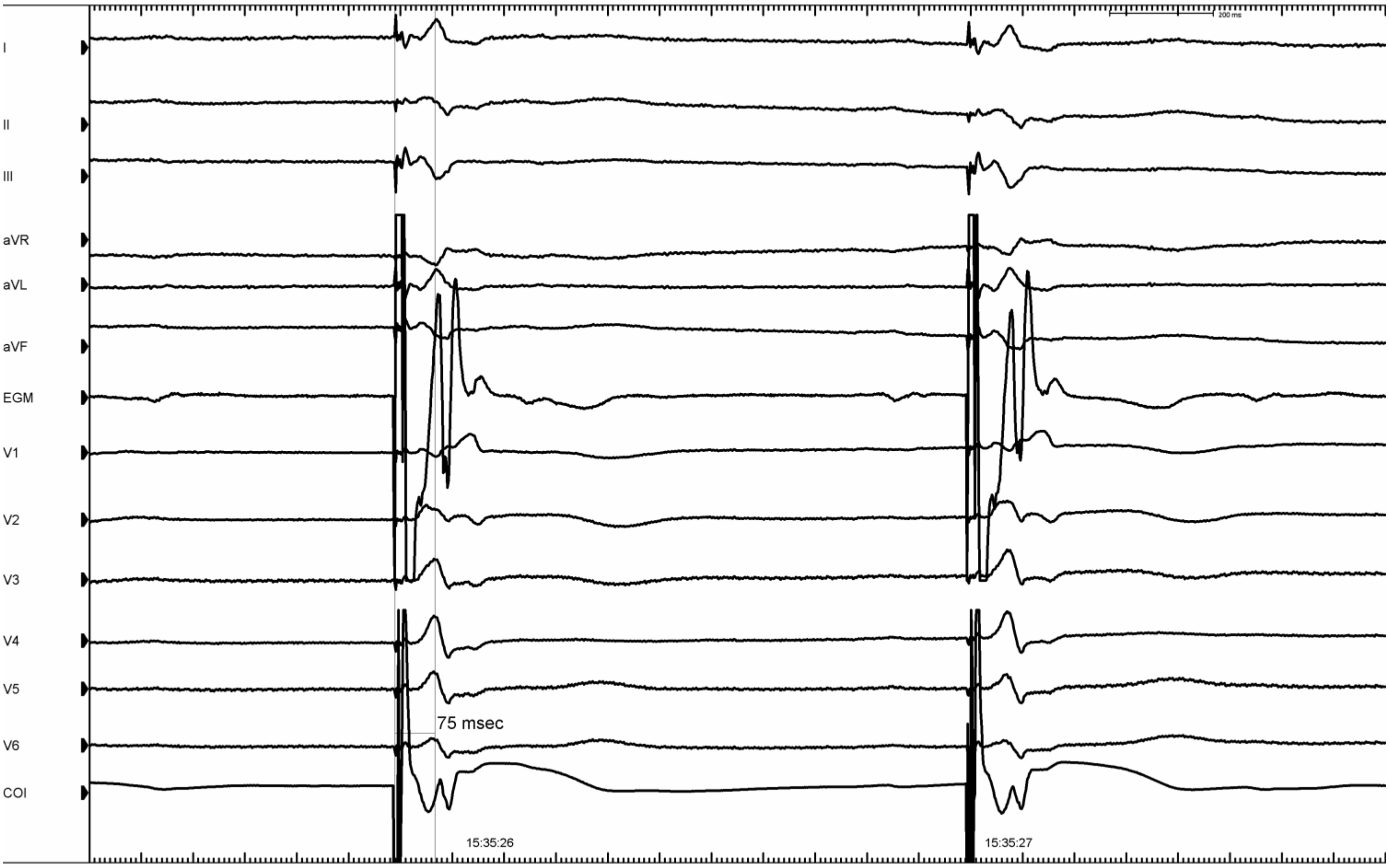

Transitions

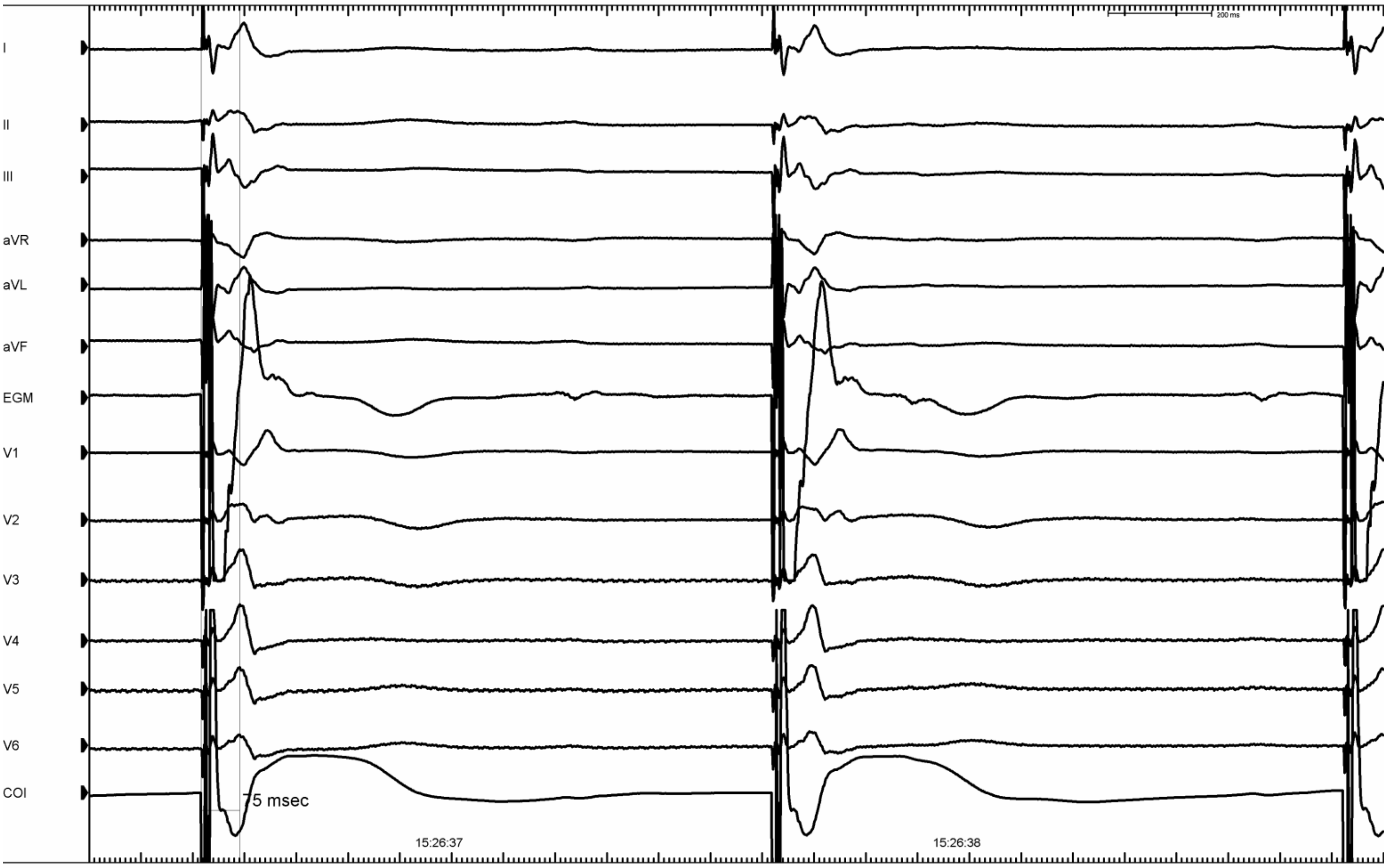

Patient 80:
Pre-ECG

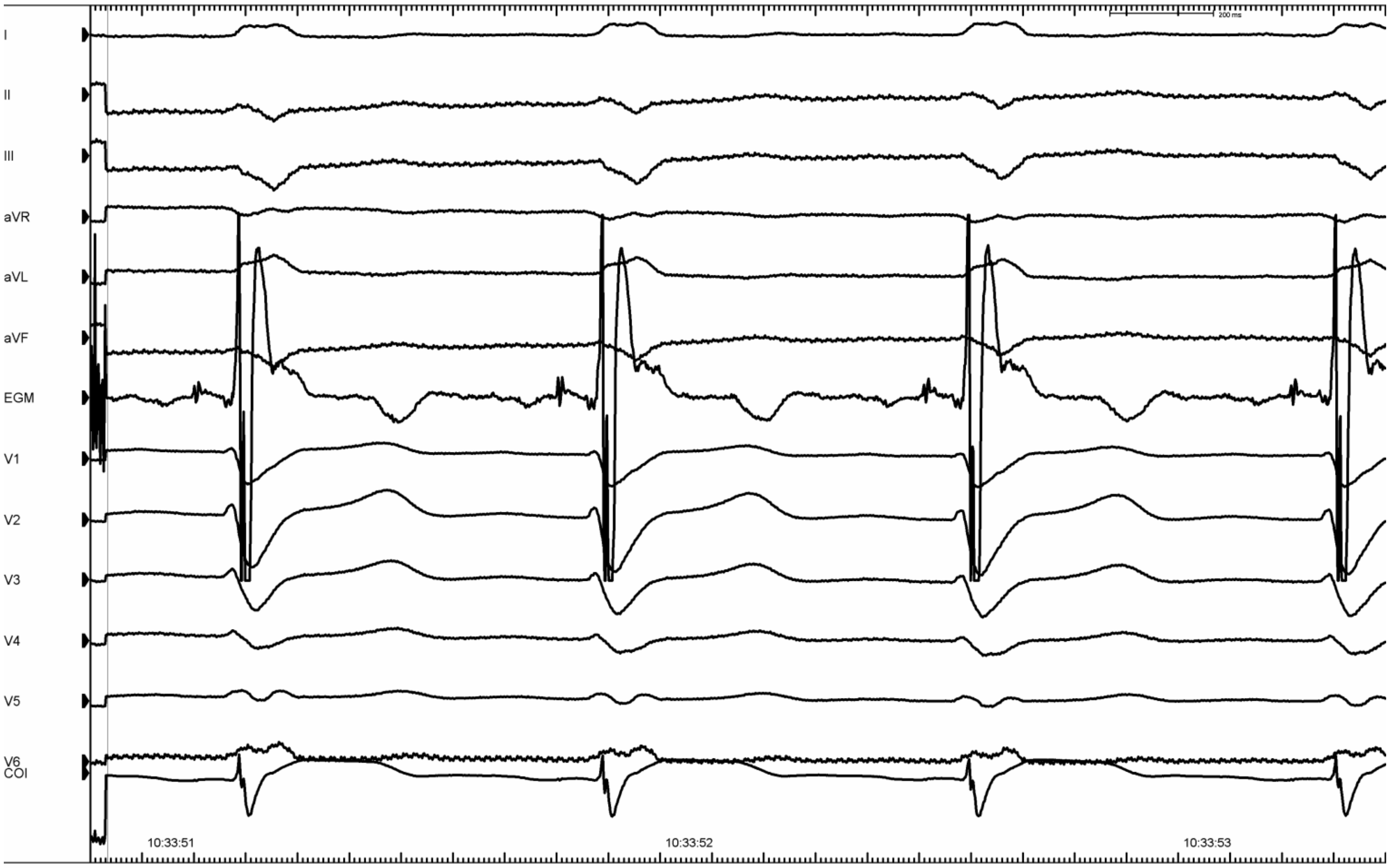

Post ECG

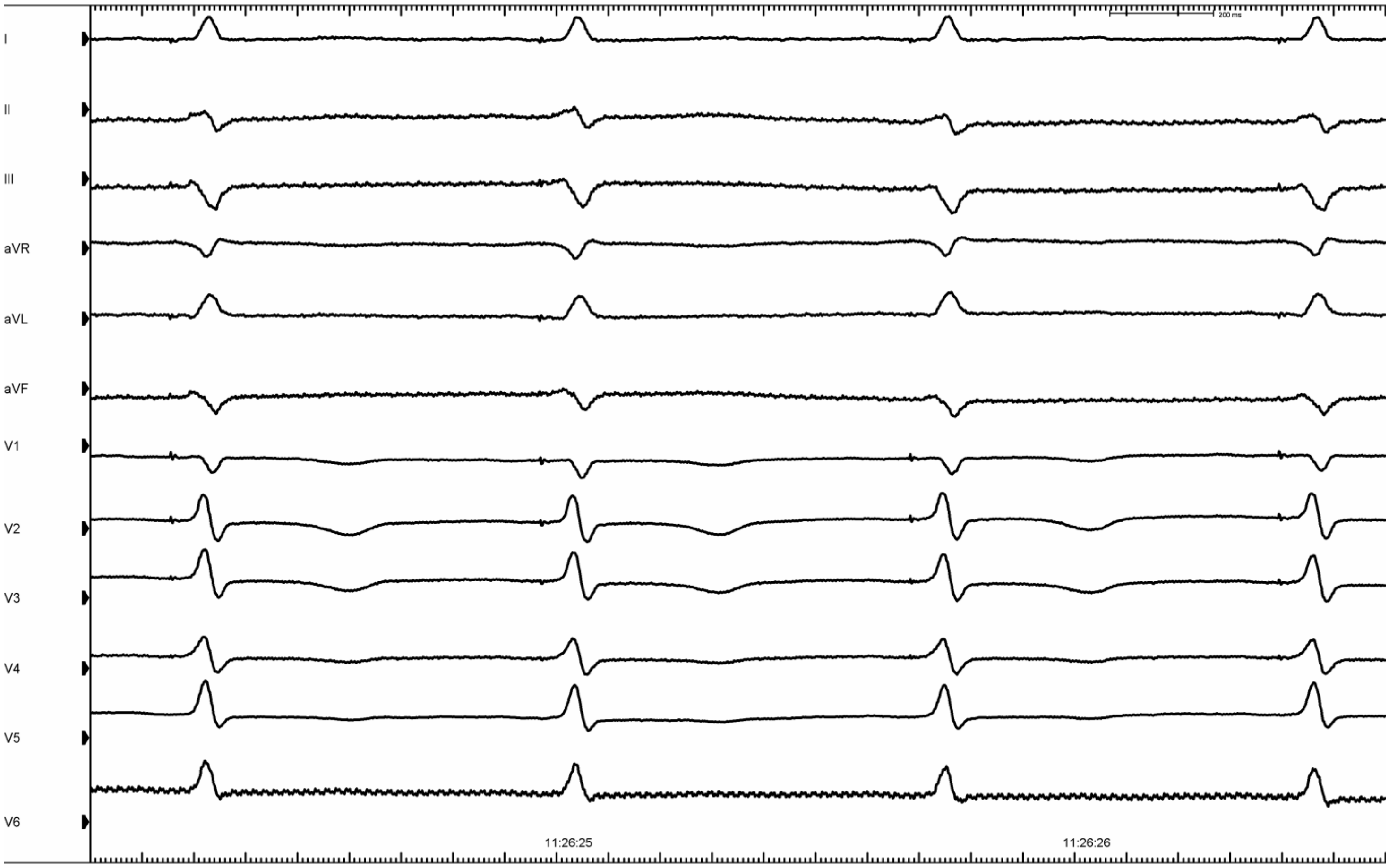

Patient 80:
Transitions

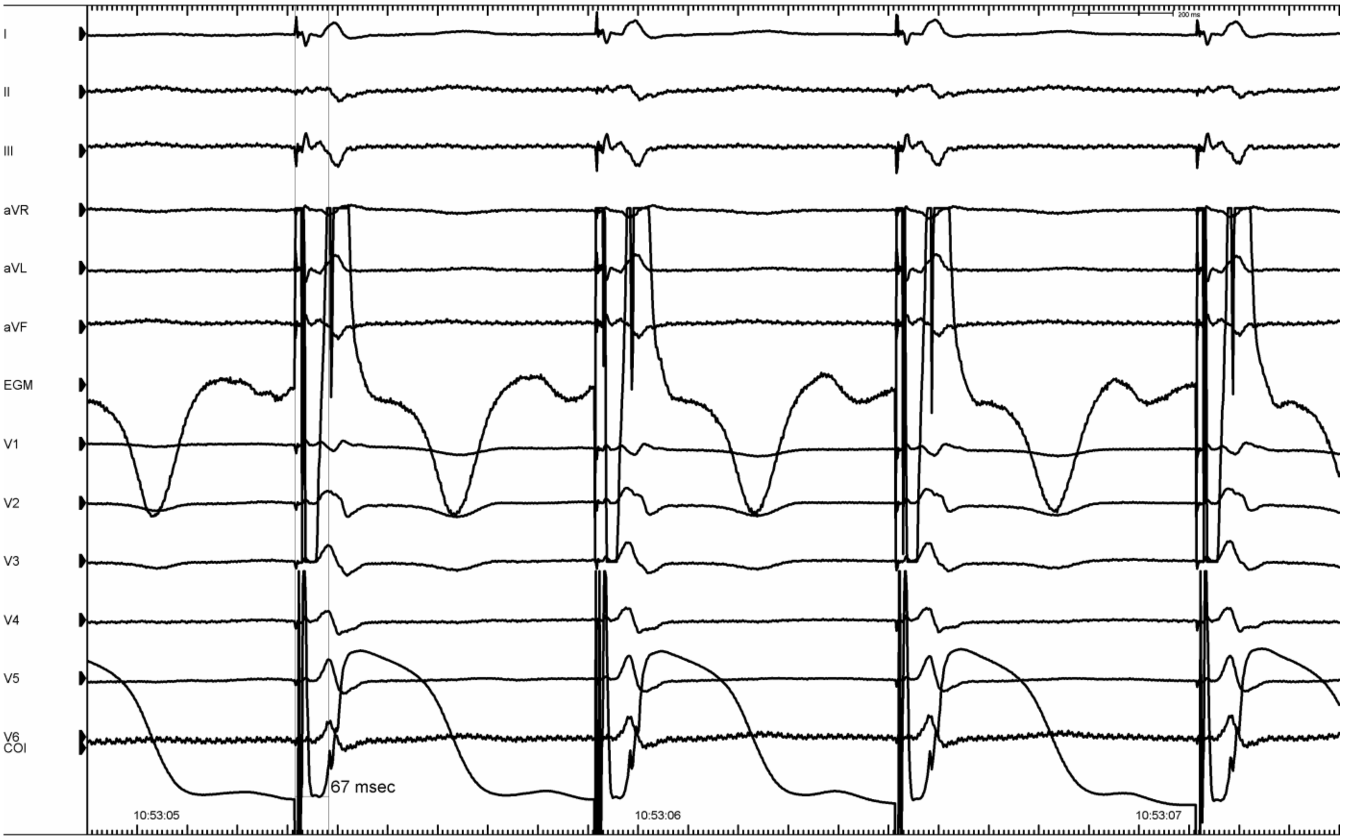

Transitions

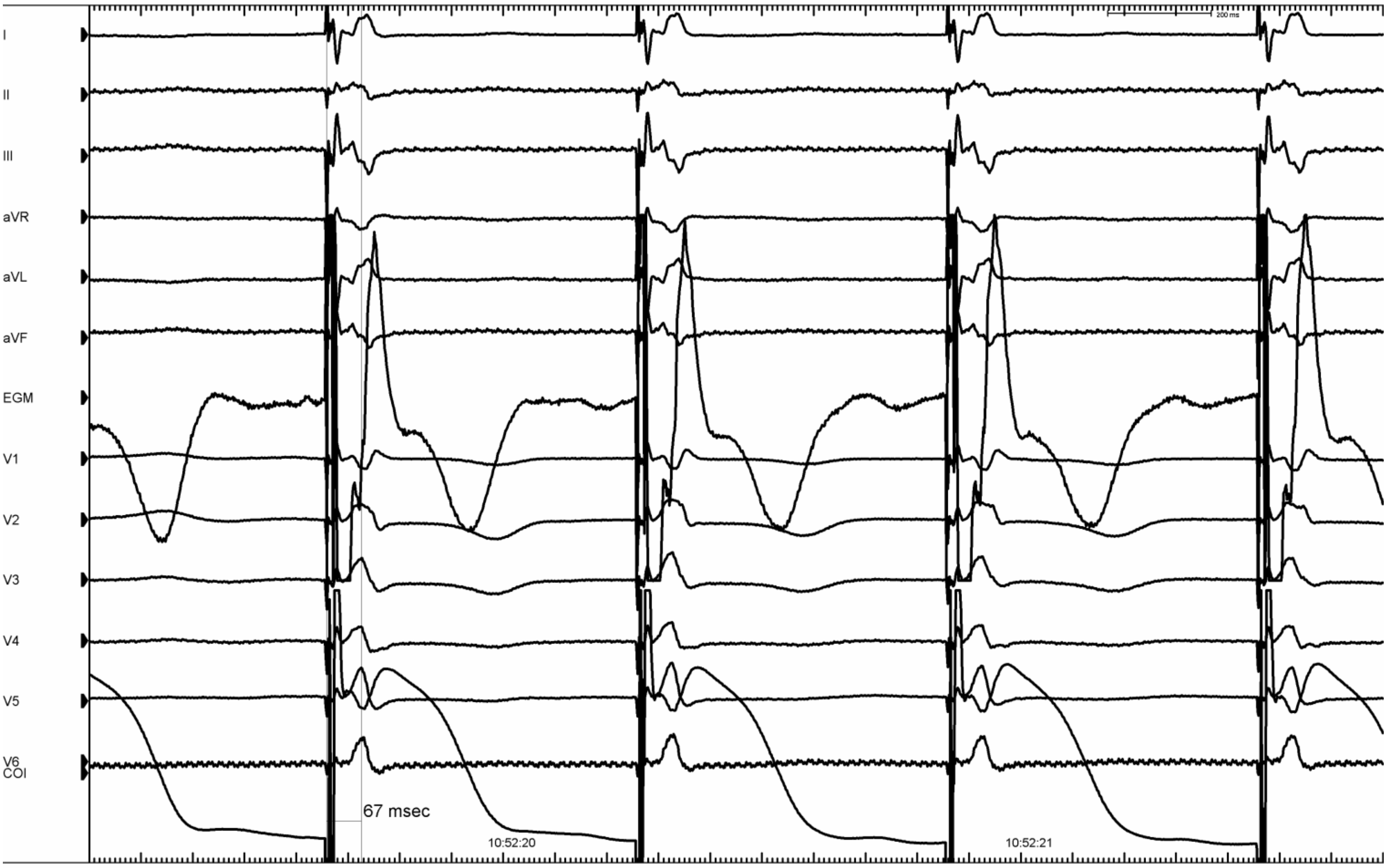

Patient 81:
Pre-ECG

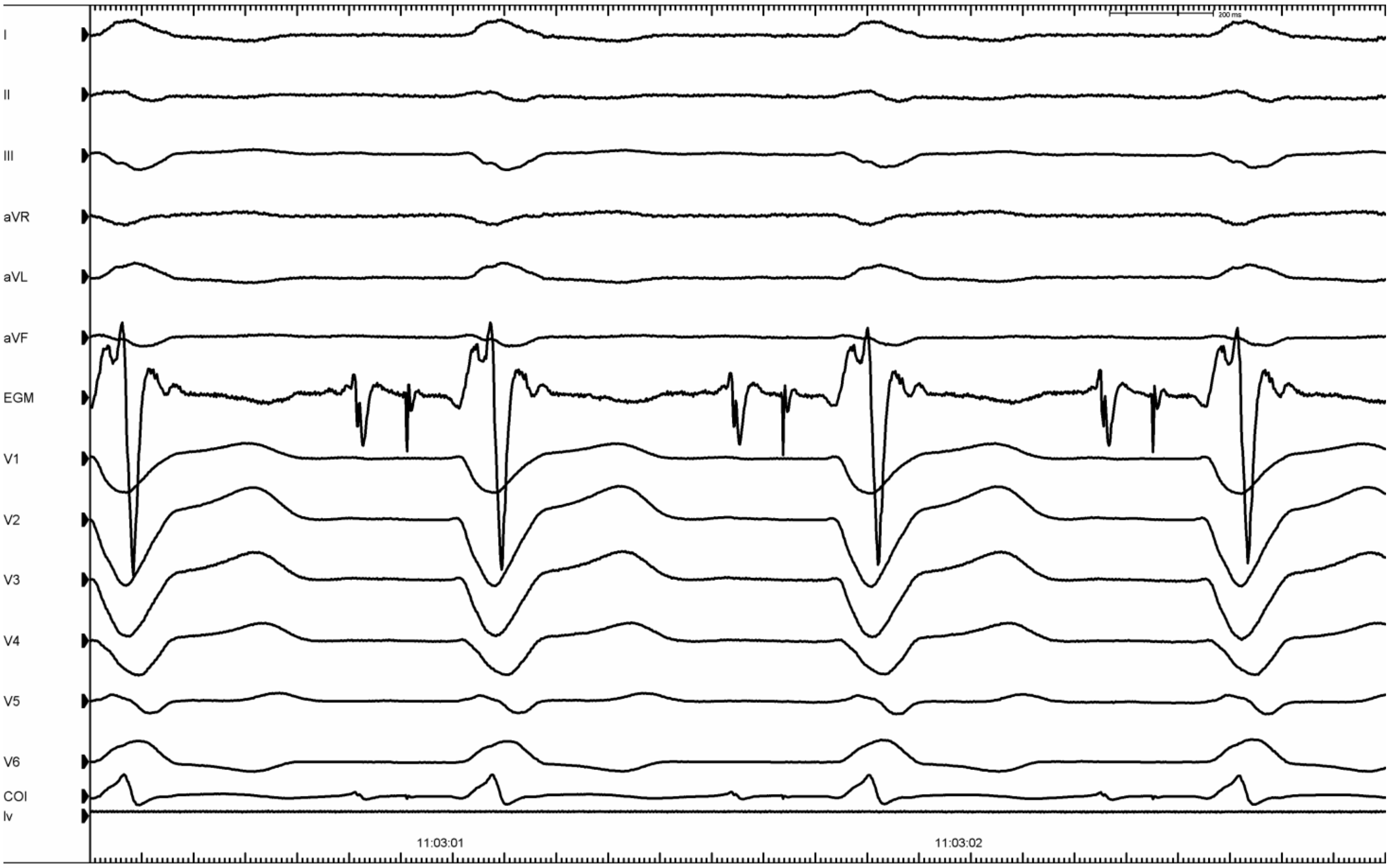

Post ECG

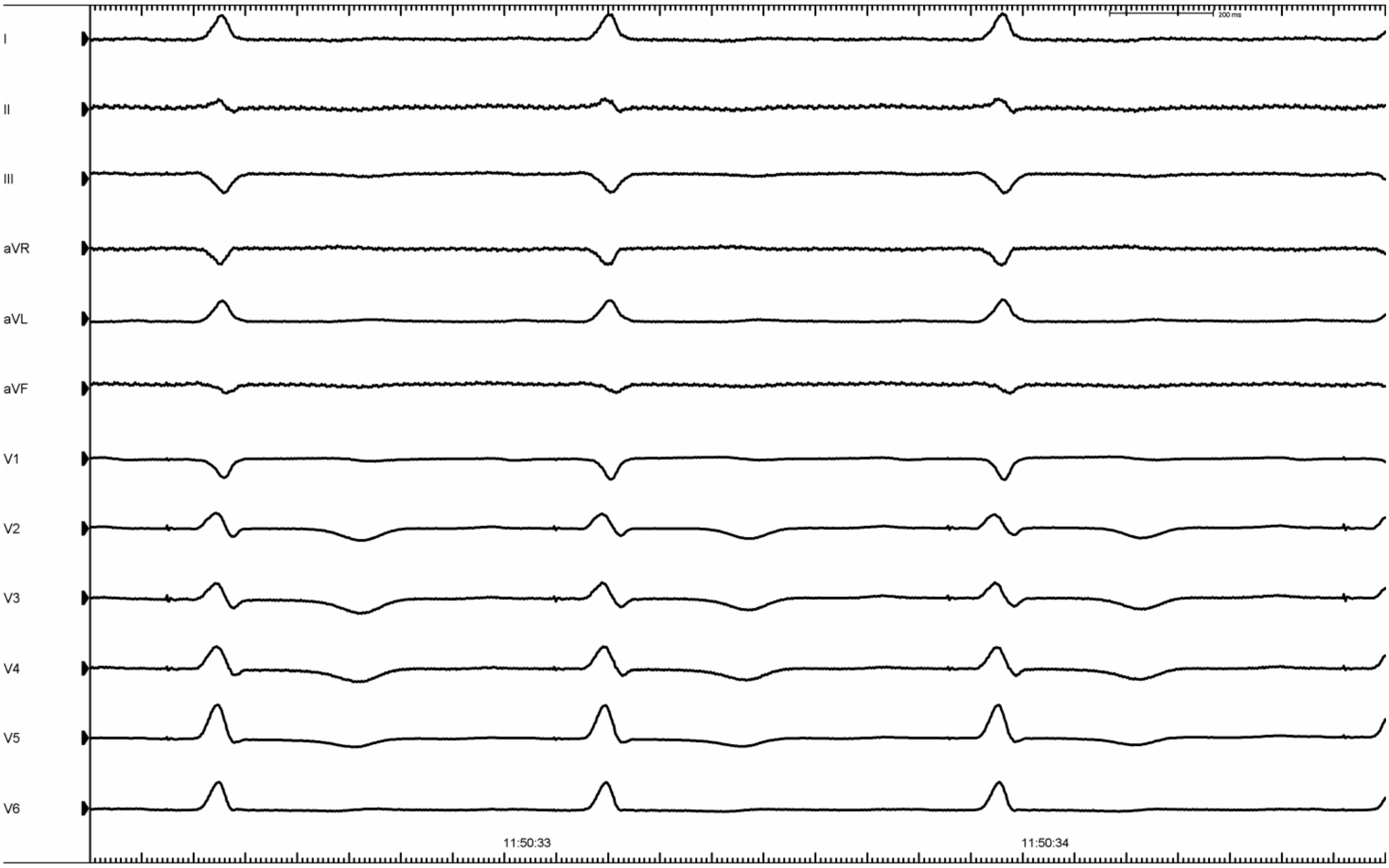

Patient 81: Transitions

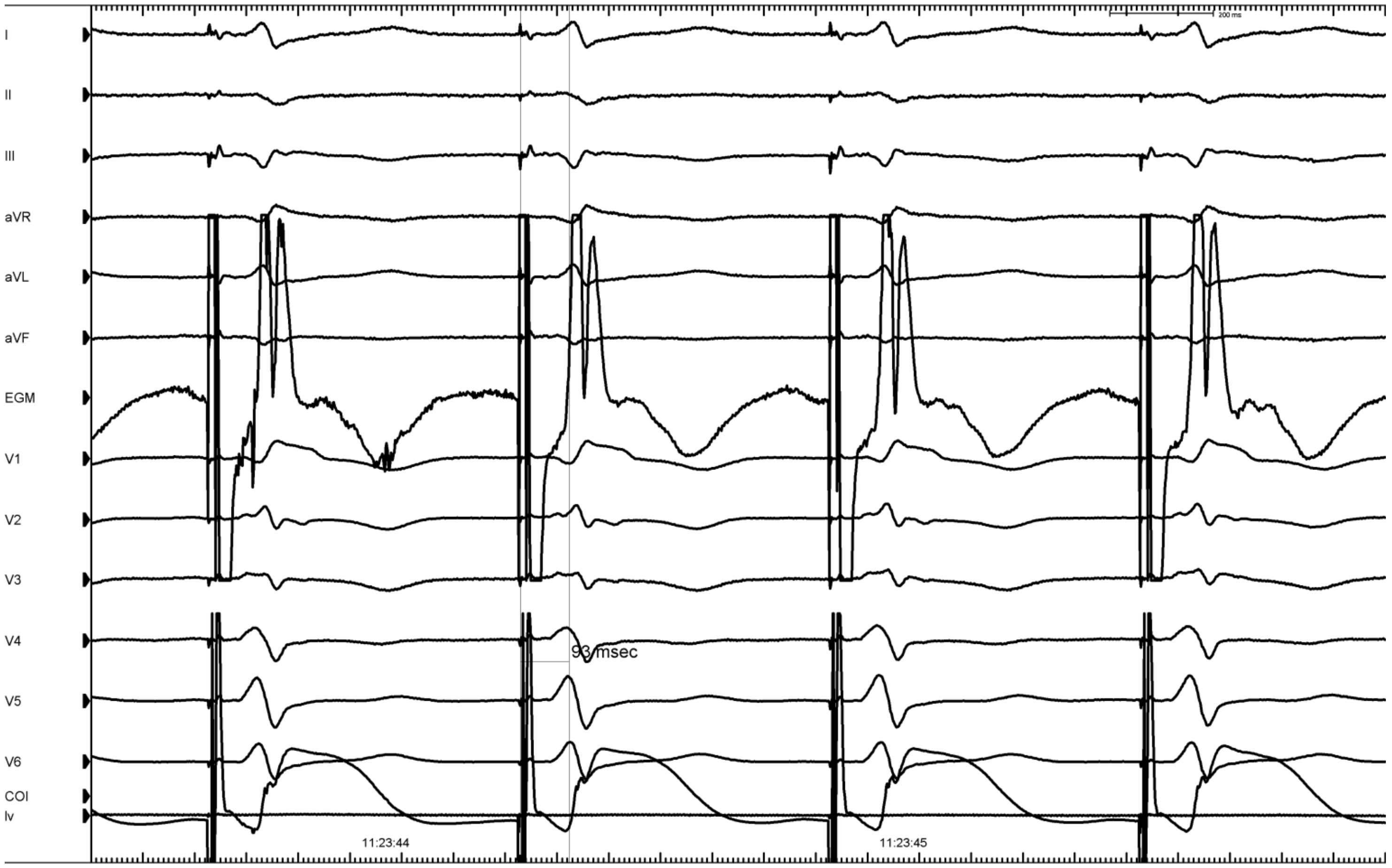

Transitions

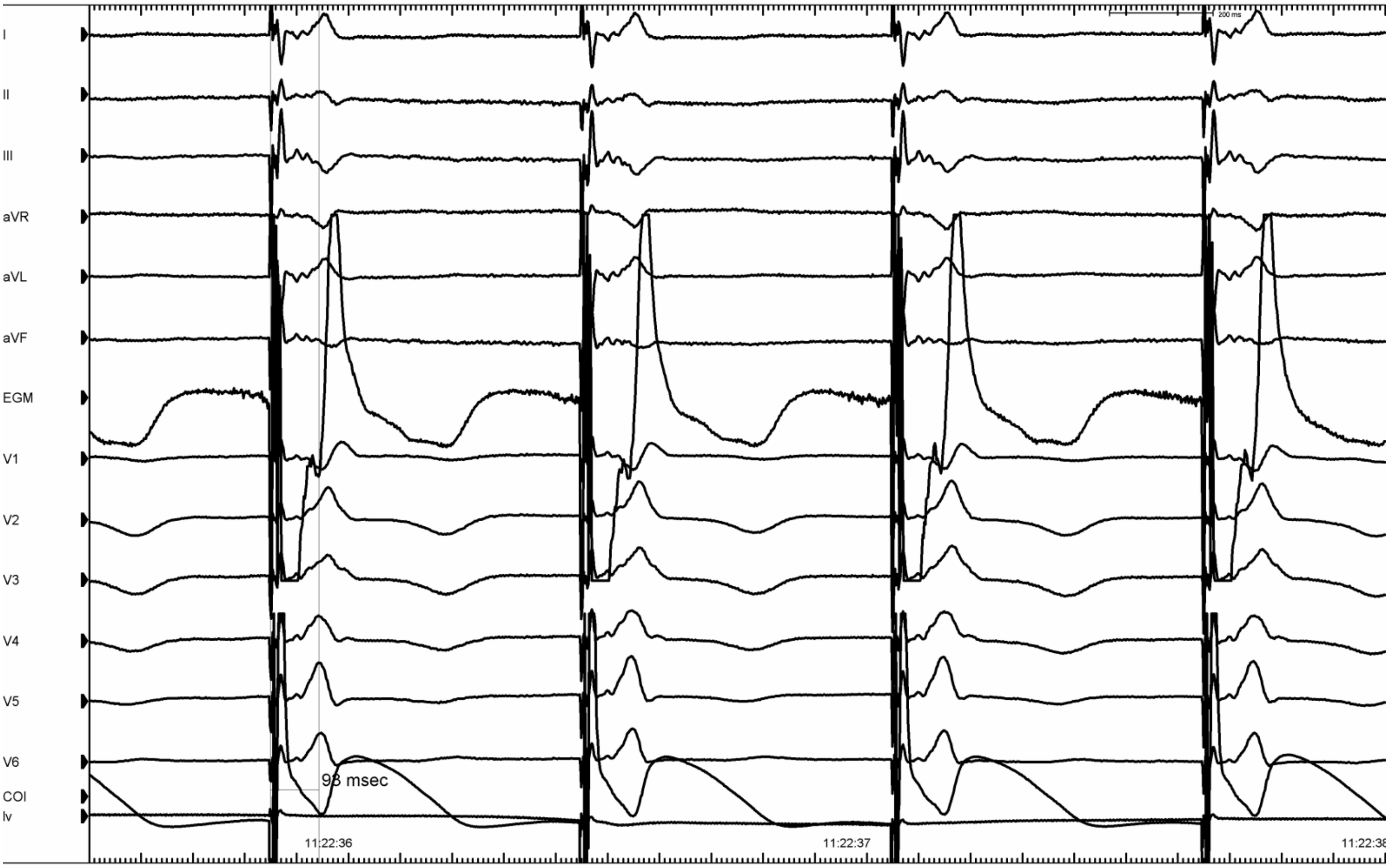

Patient 82:
Pre-ECG

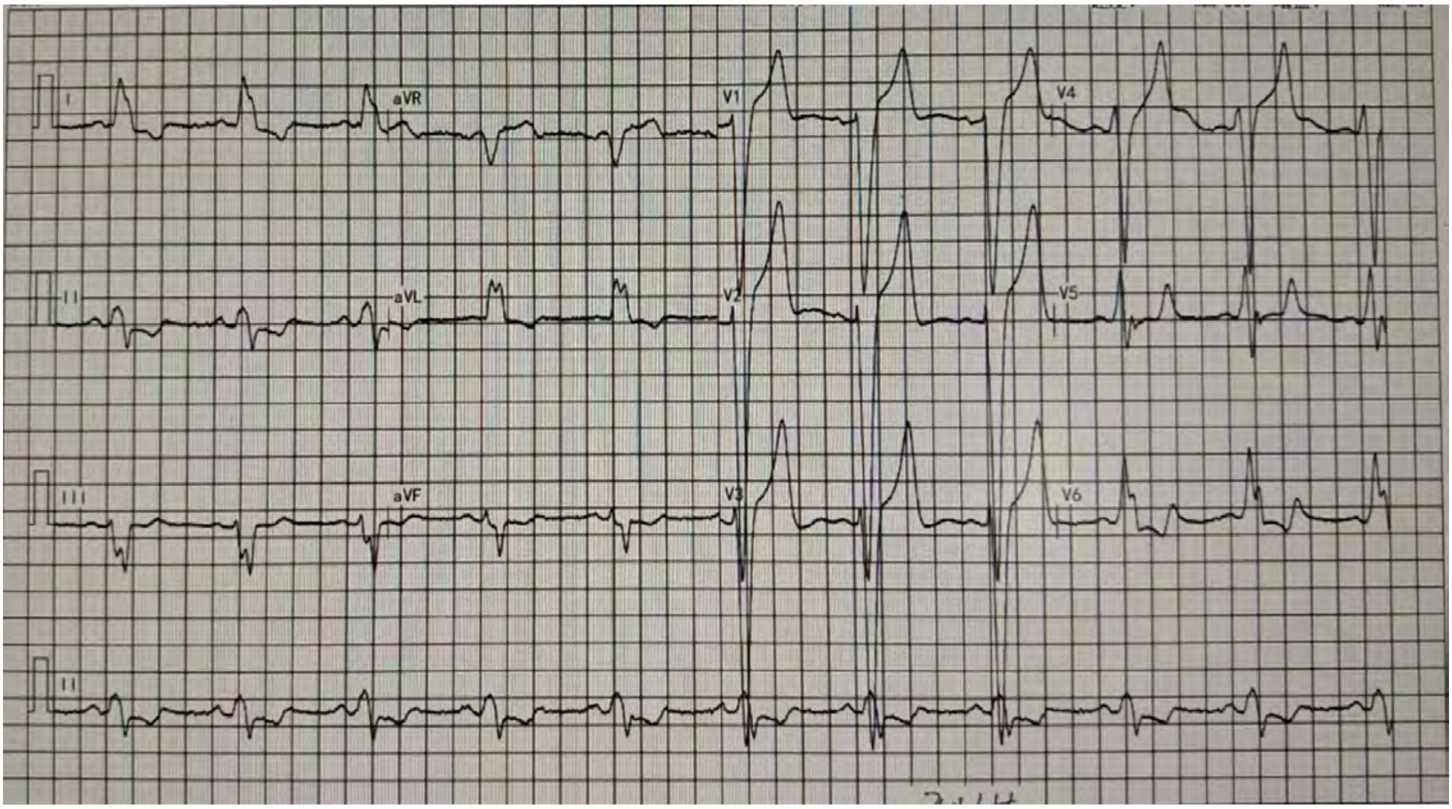

Post ECG

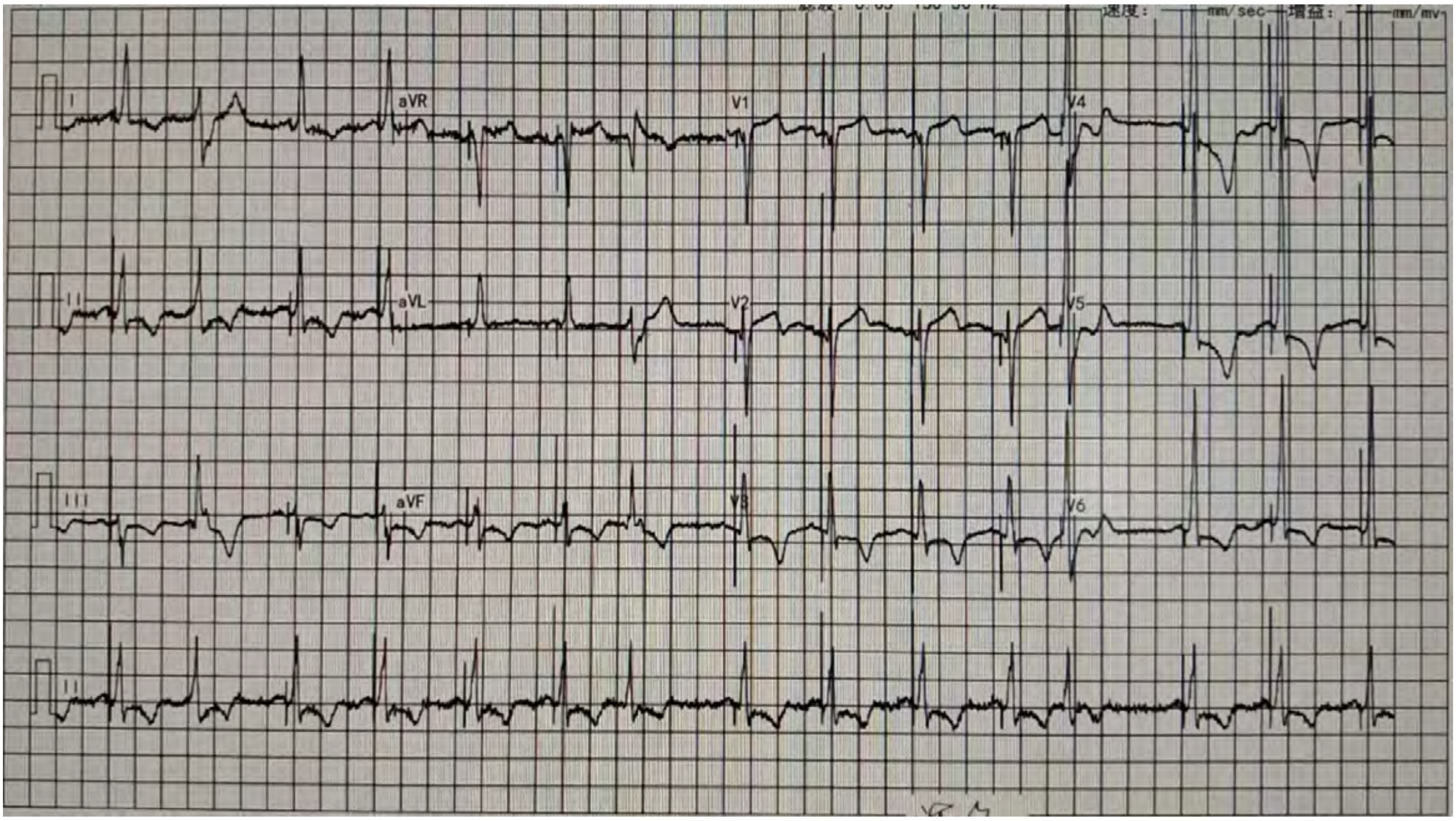

Patient 82:
Transitions

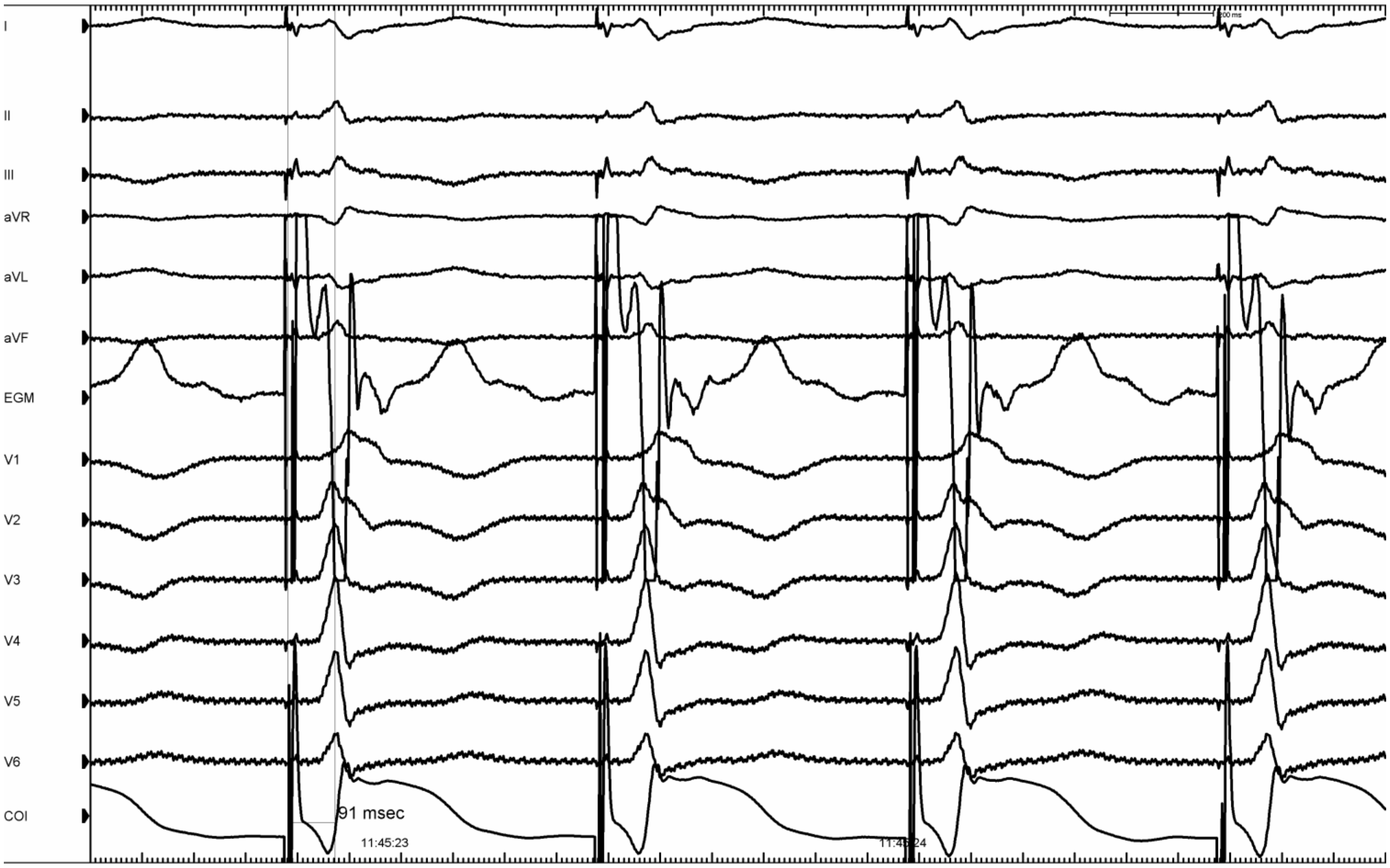

Transitions

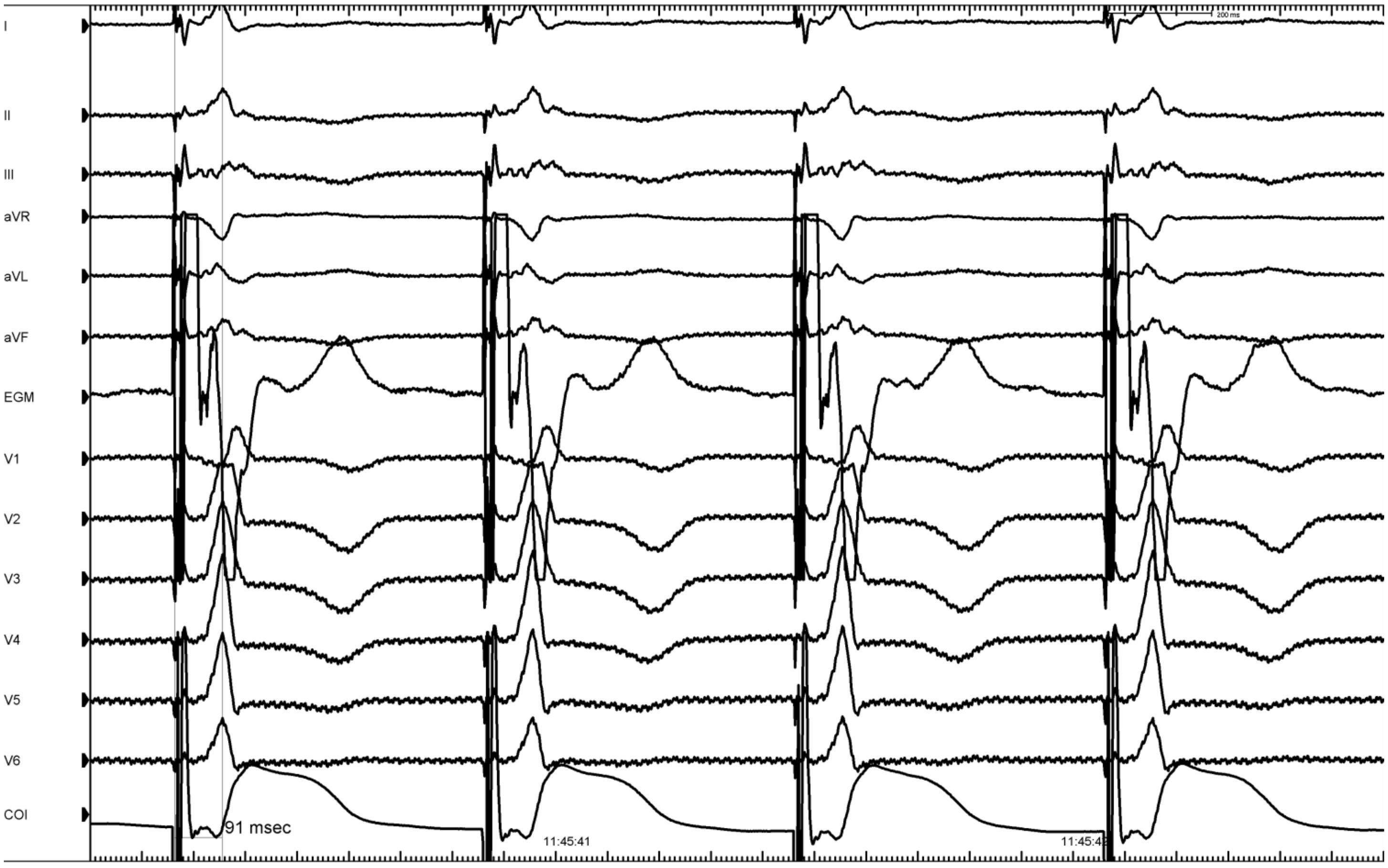

Patient 83:
Pre-ECG

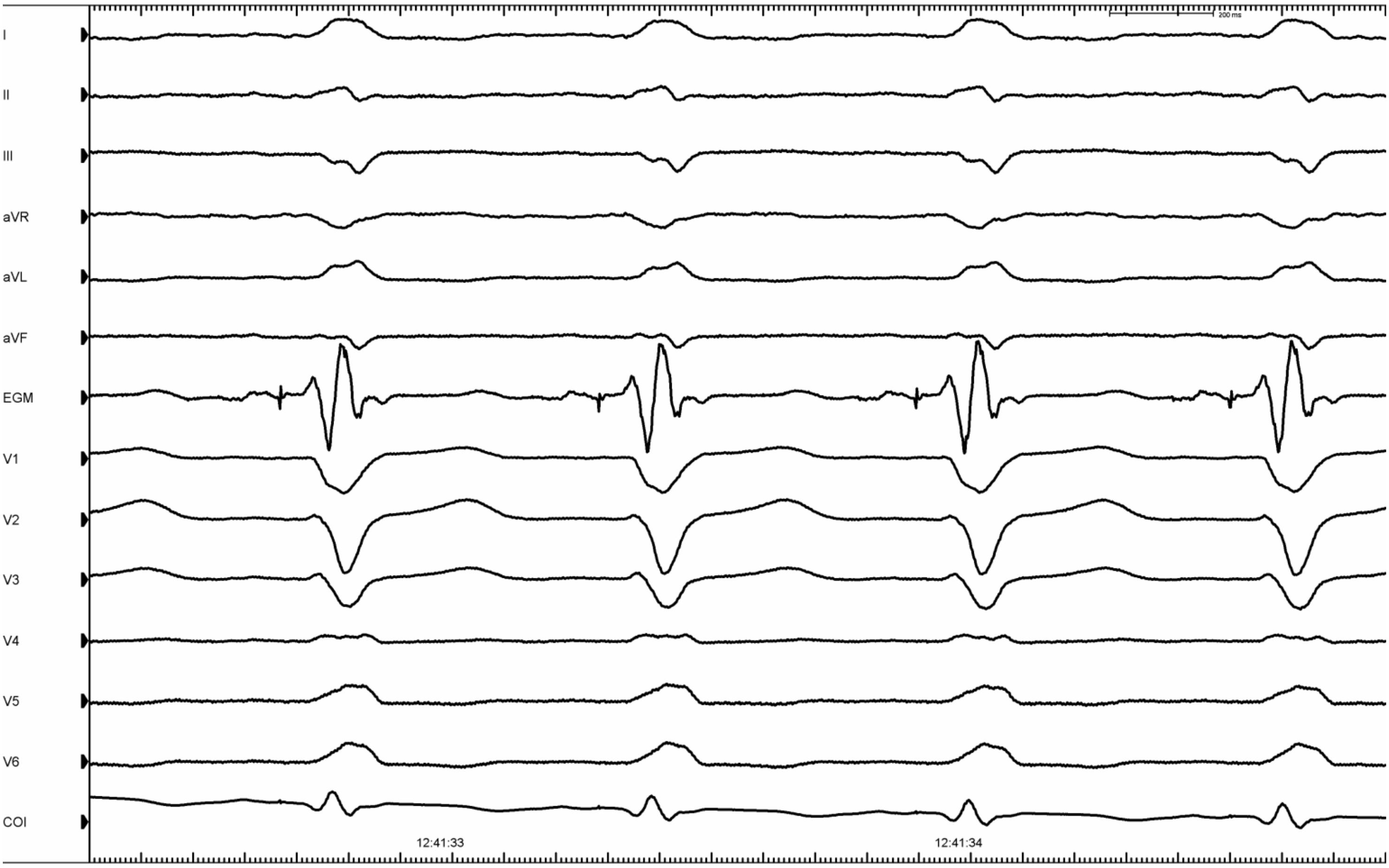

Post ECG

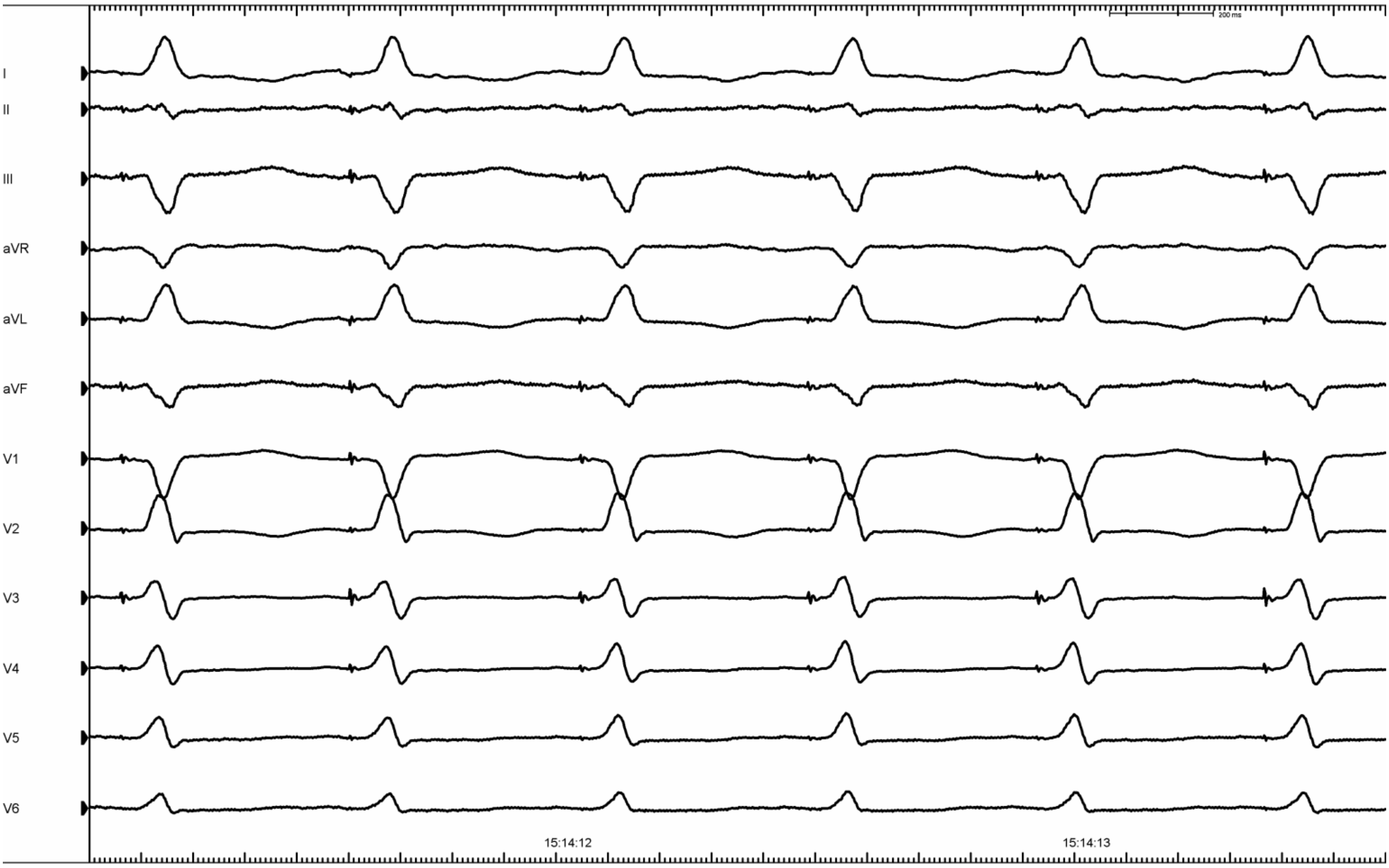

Patient 83: Transitions

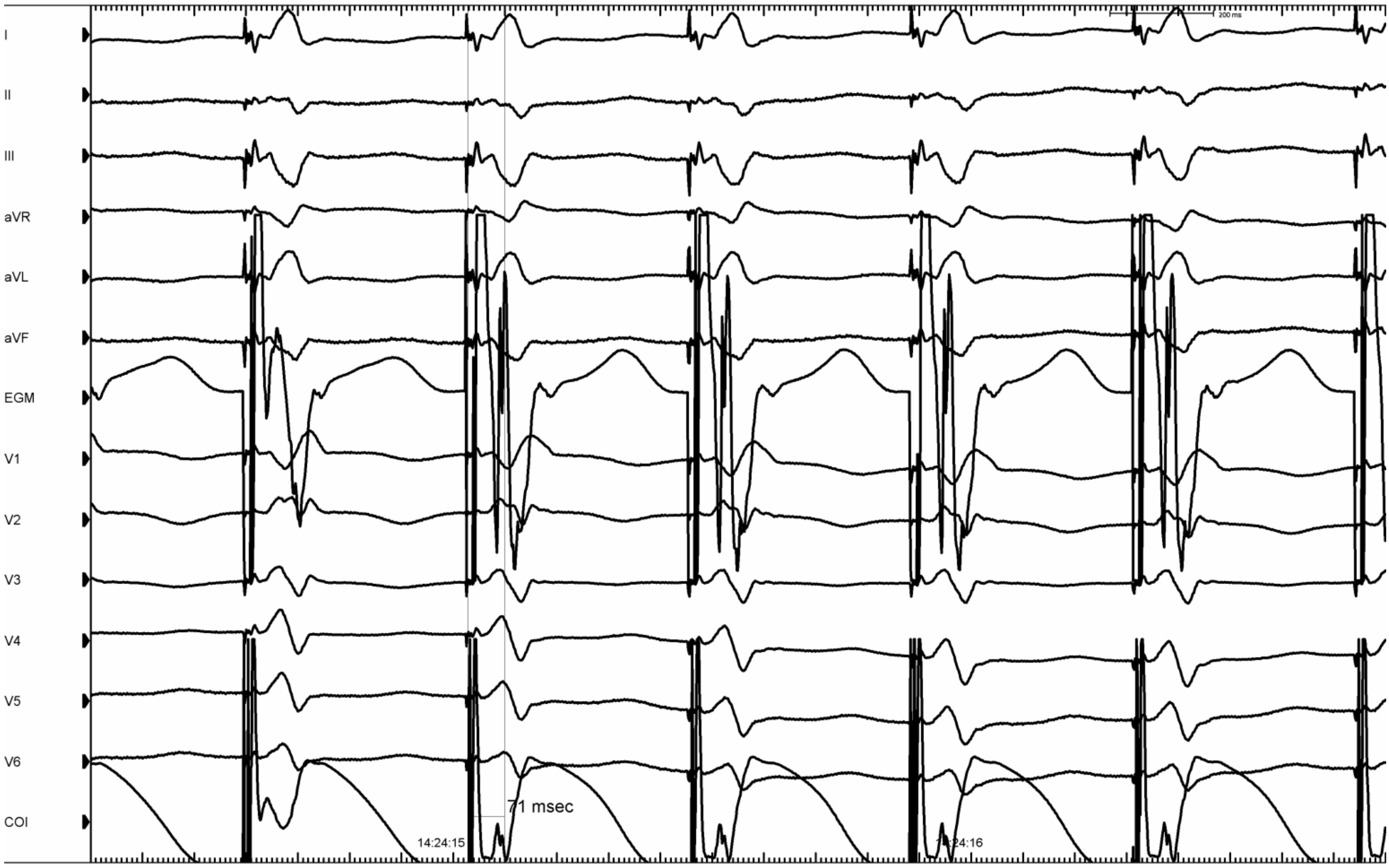

Transitions

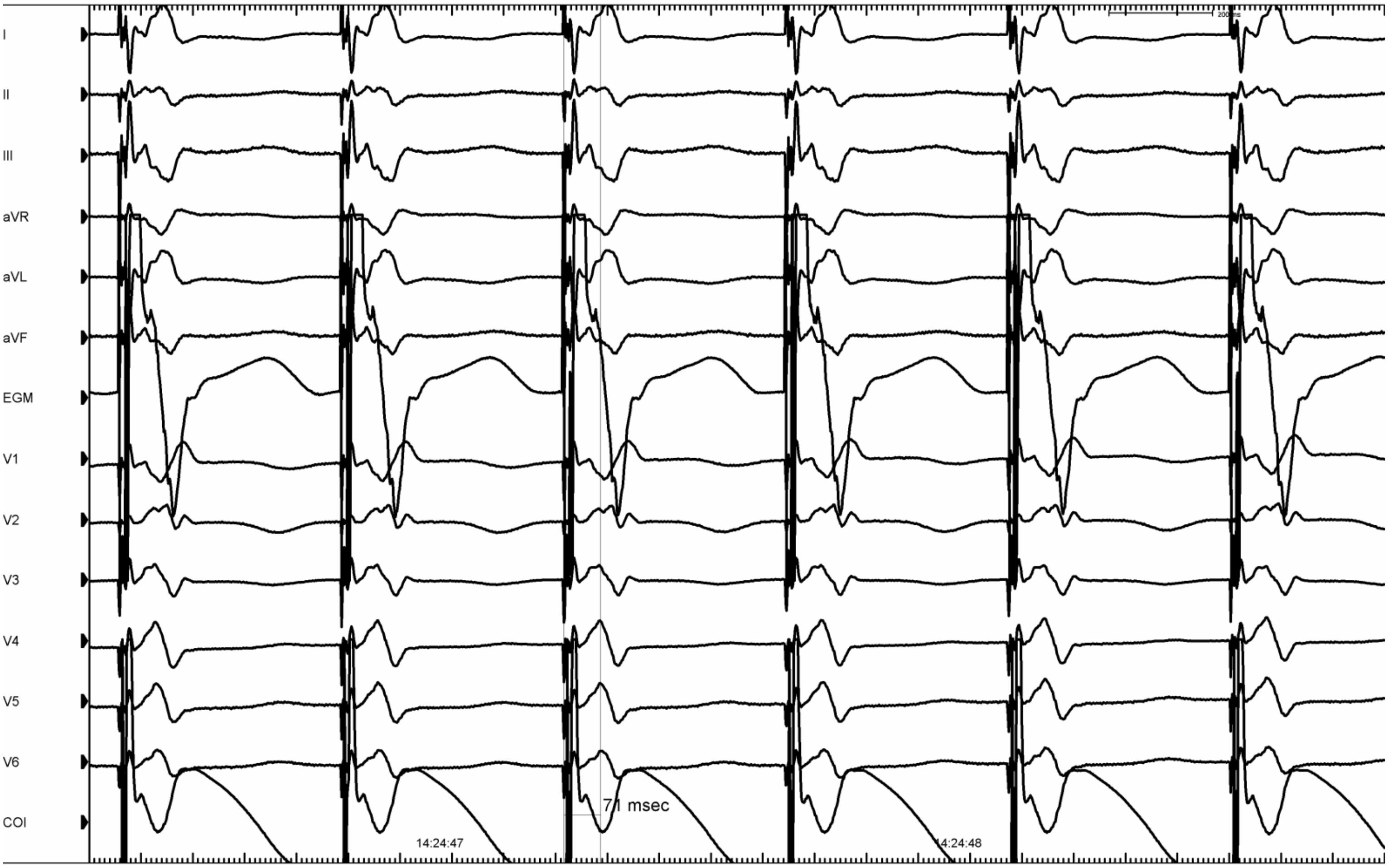

Patient 84:
Pre-ECG

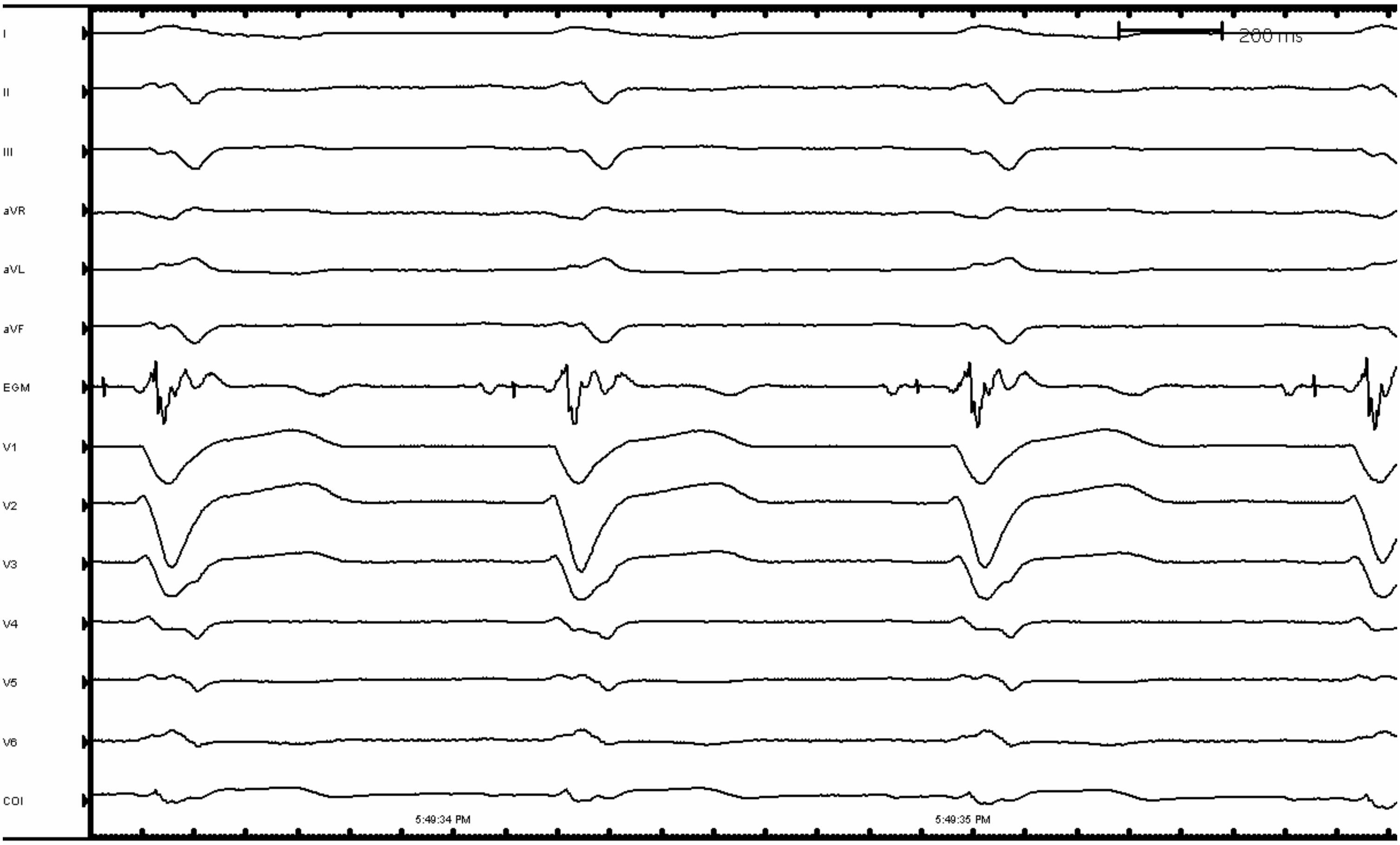

Post ECG

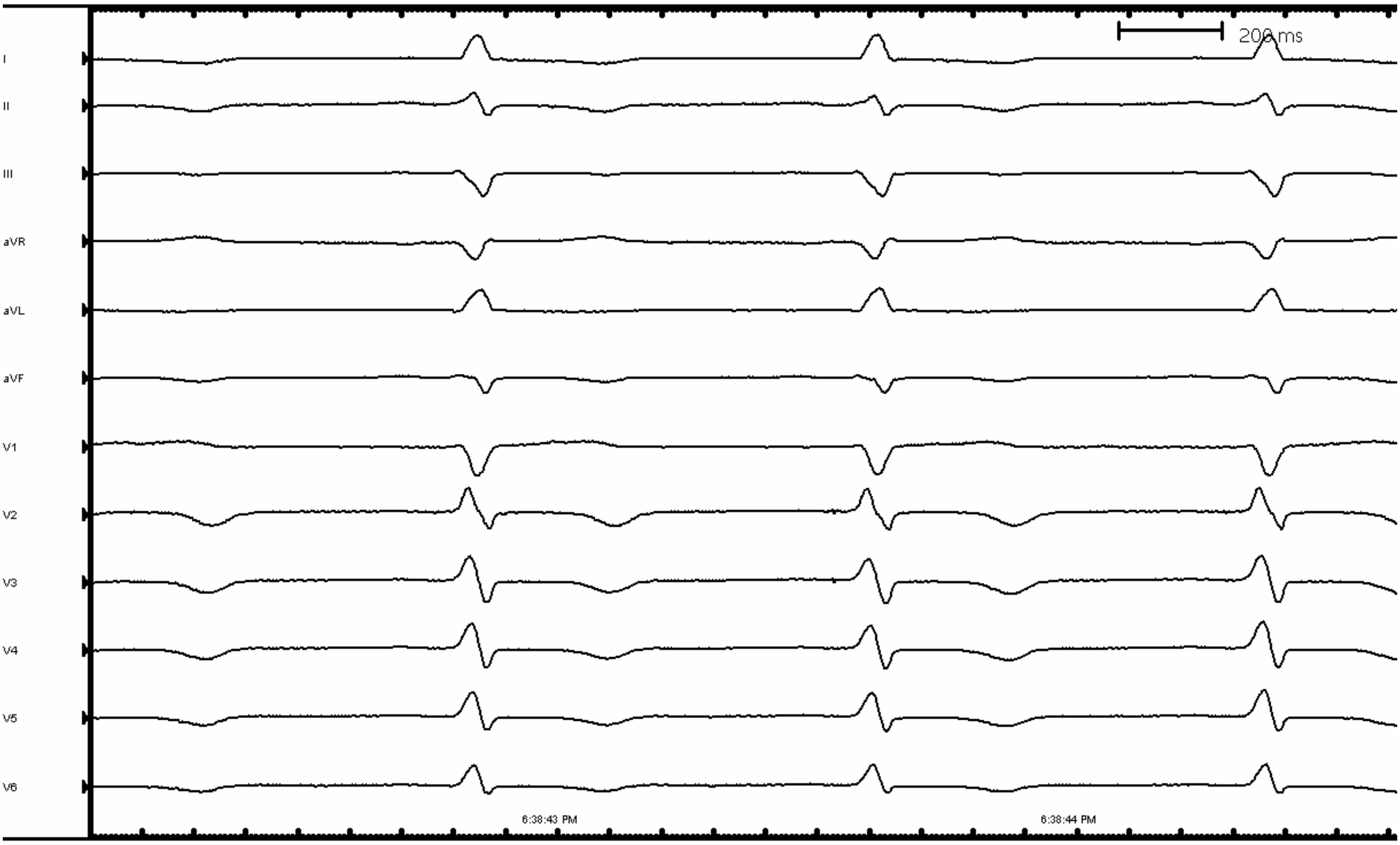

Patient 84:
Transitions

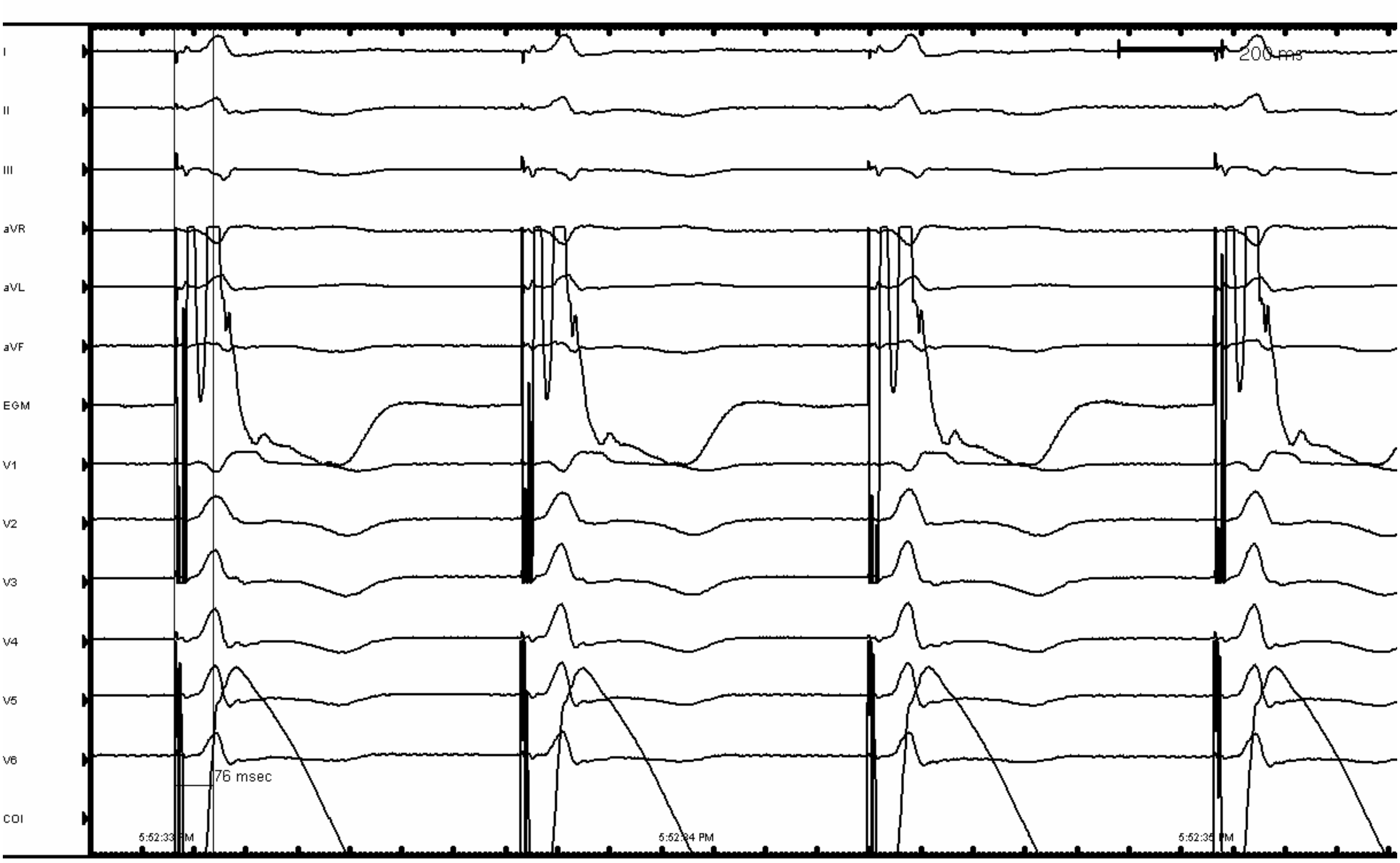

Transitions

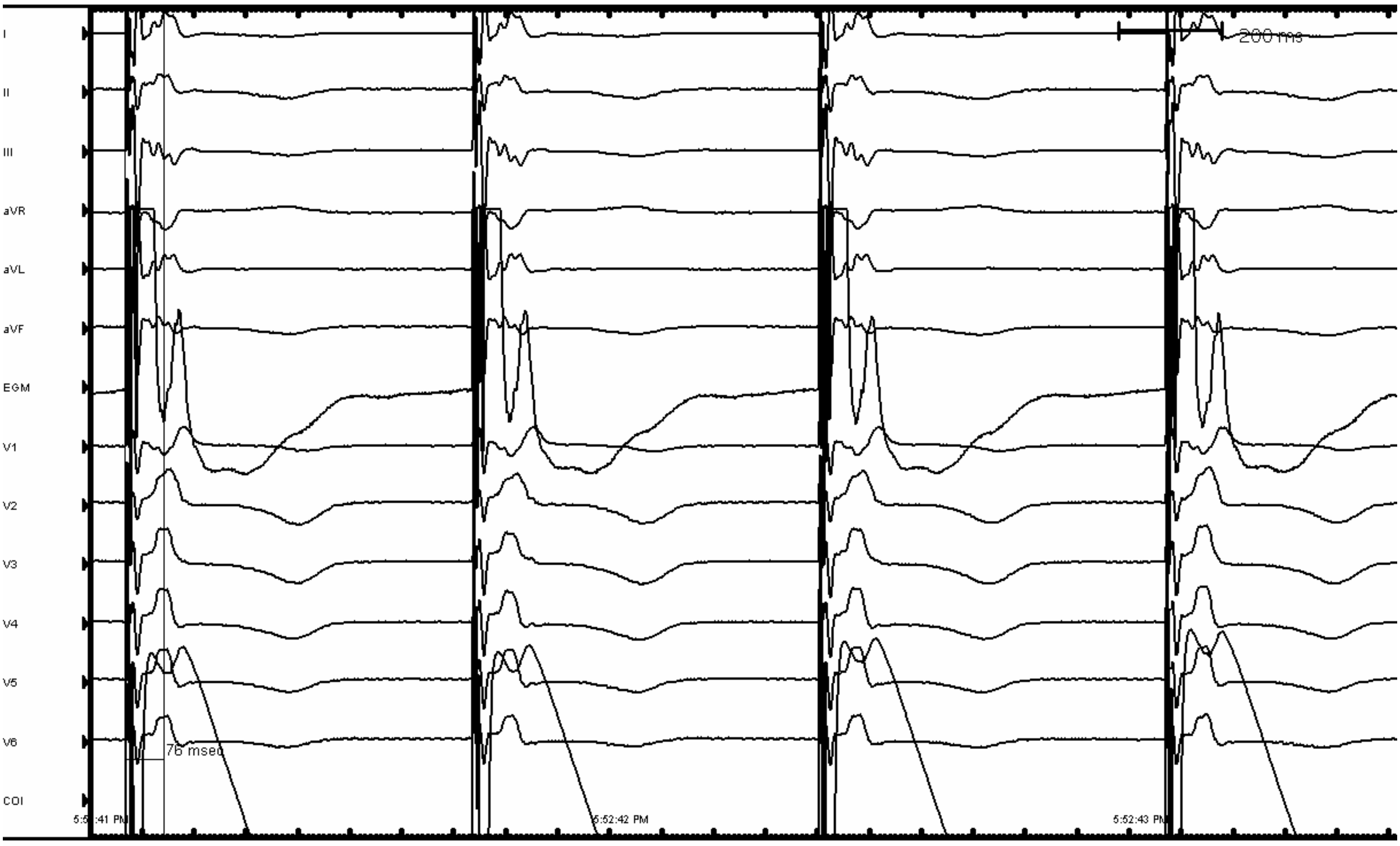

Patient 85:
Pre-ECG

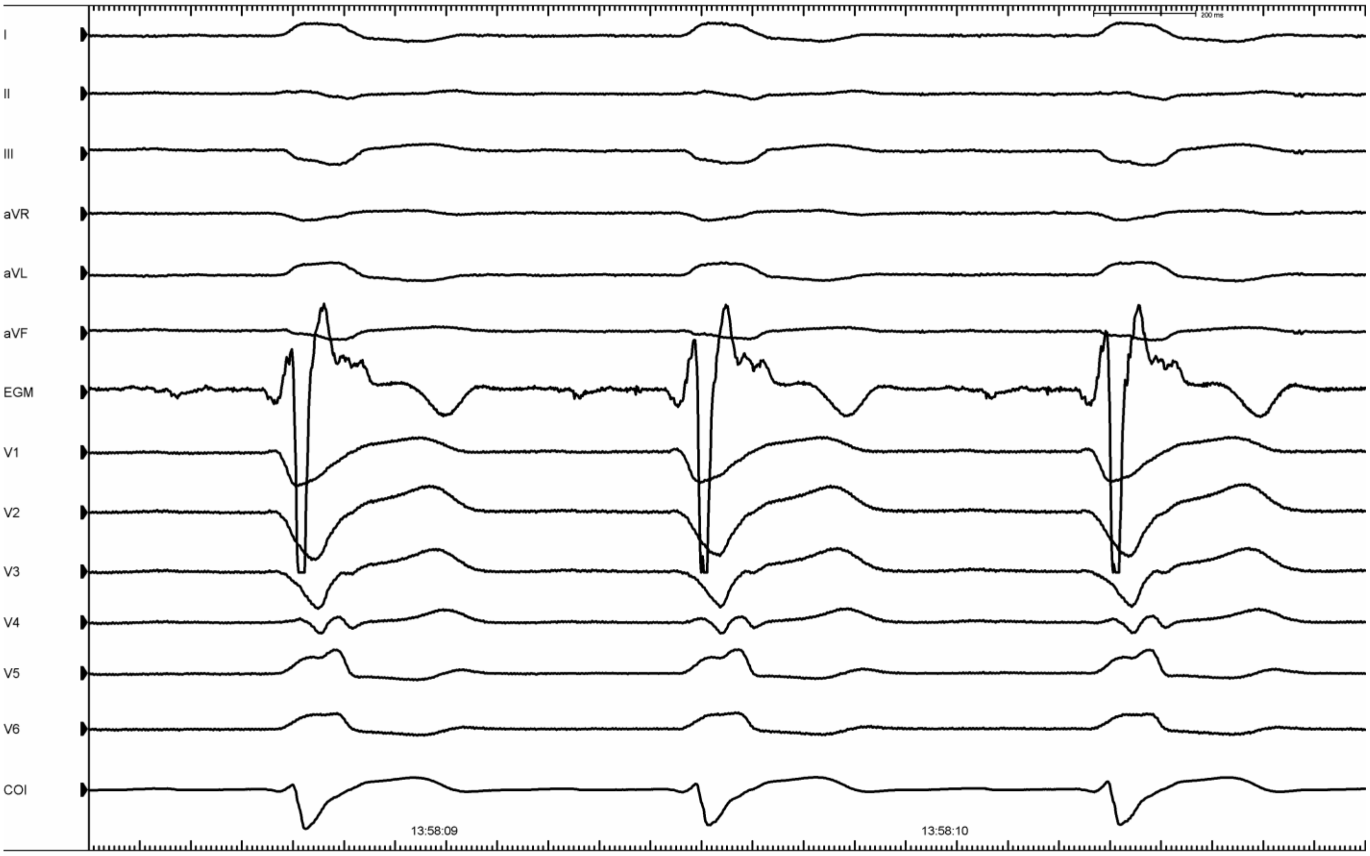

Post ECG

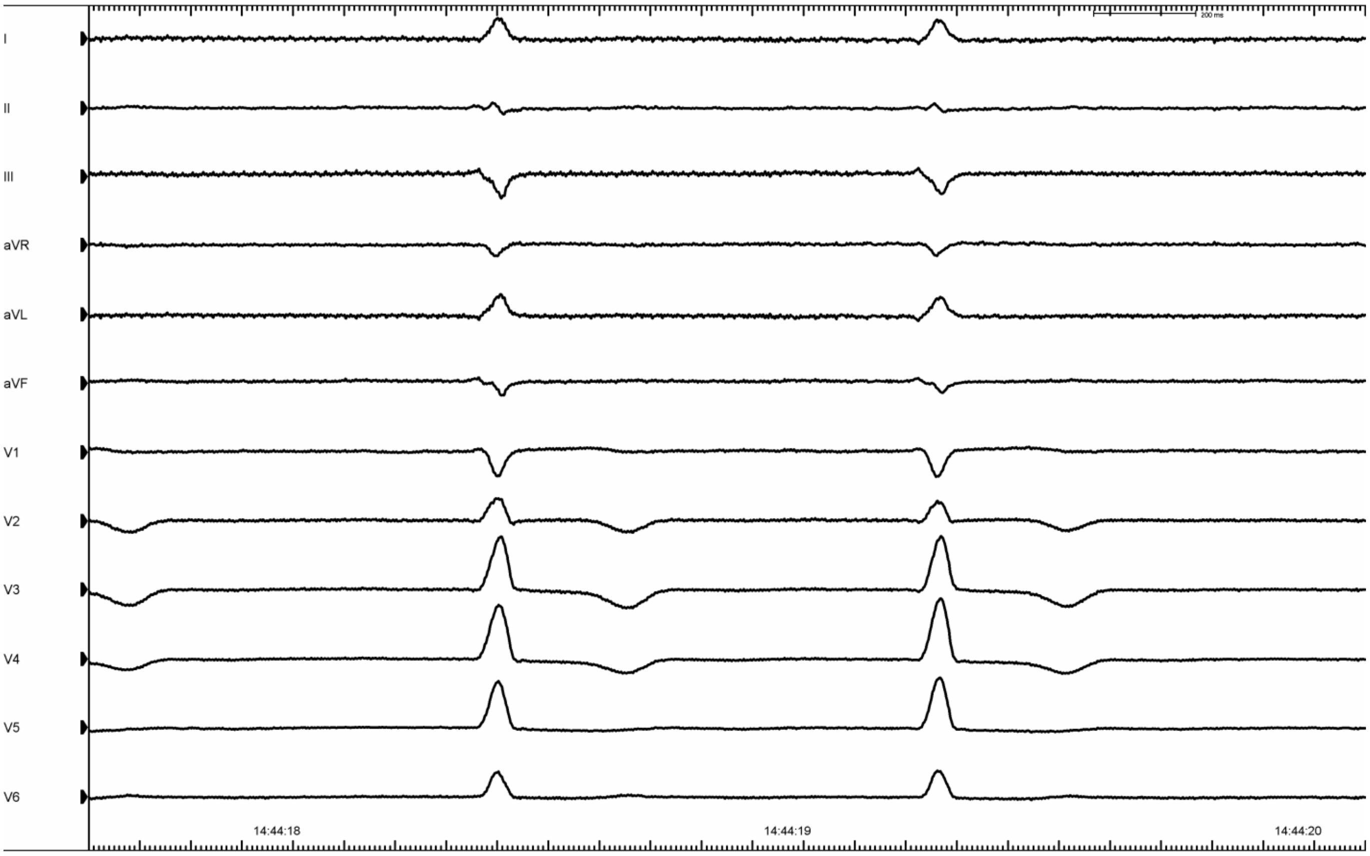

Patient 85:
Transitions

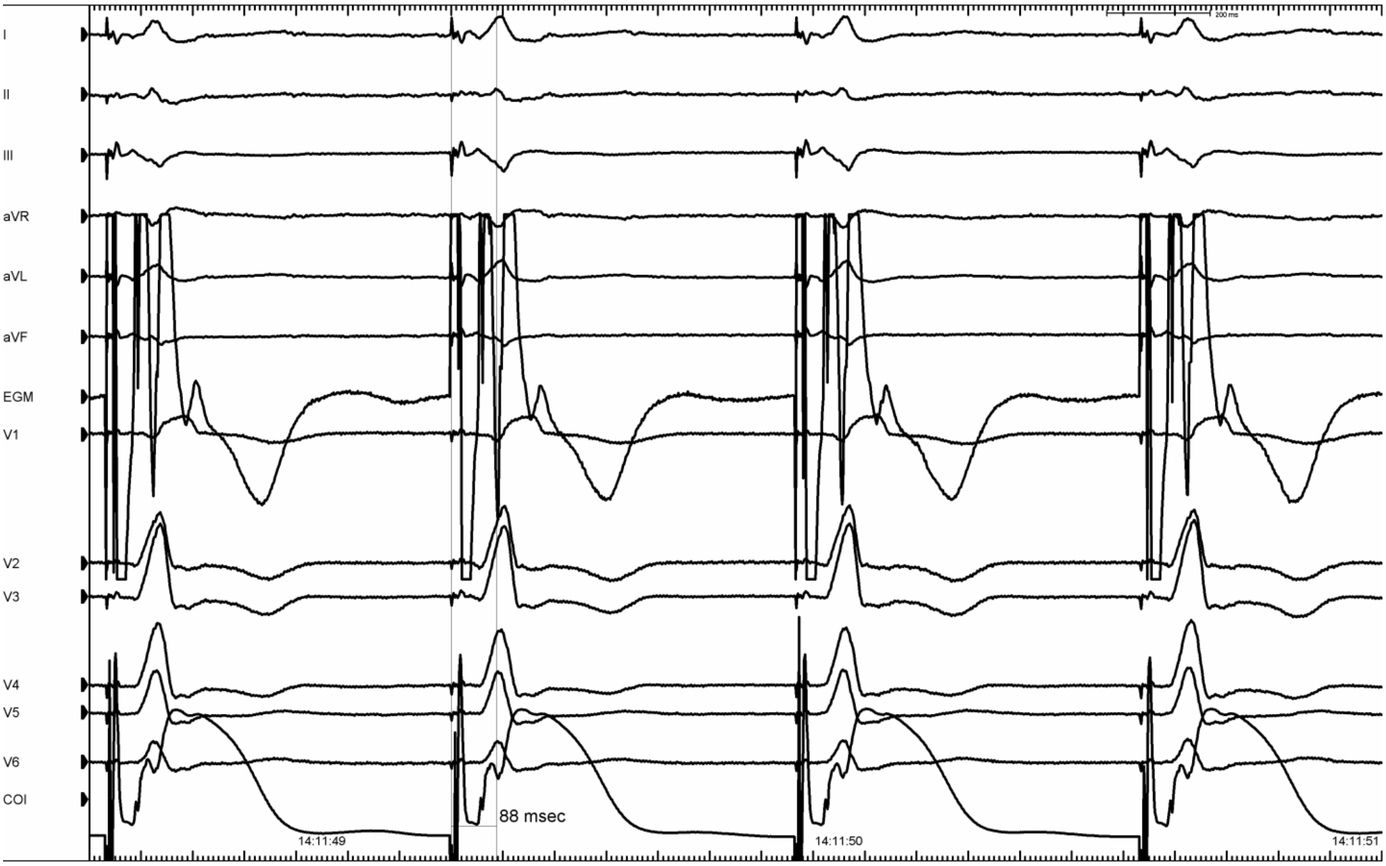

Transitions

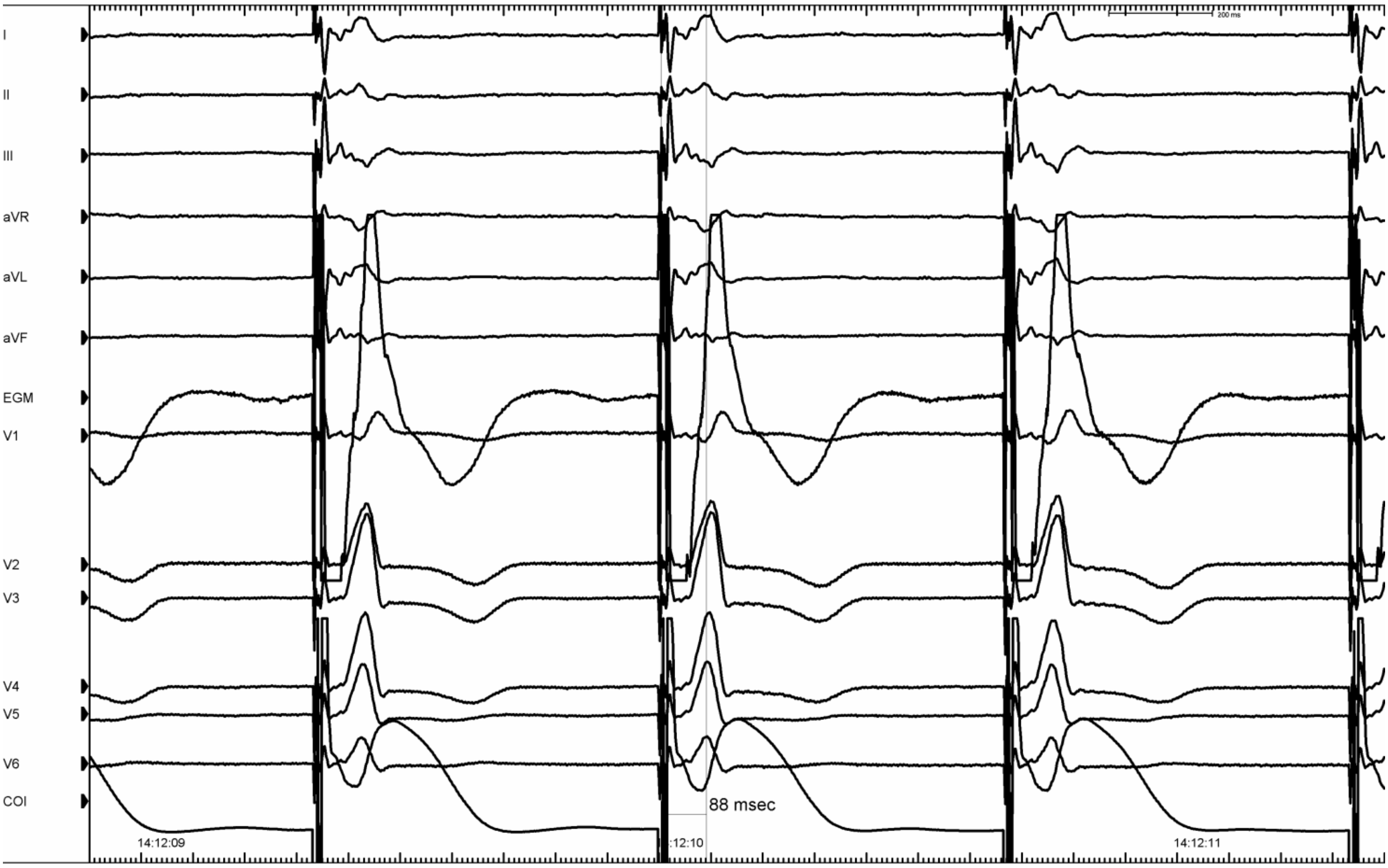

Patient 86:
Pre-ECG

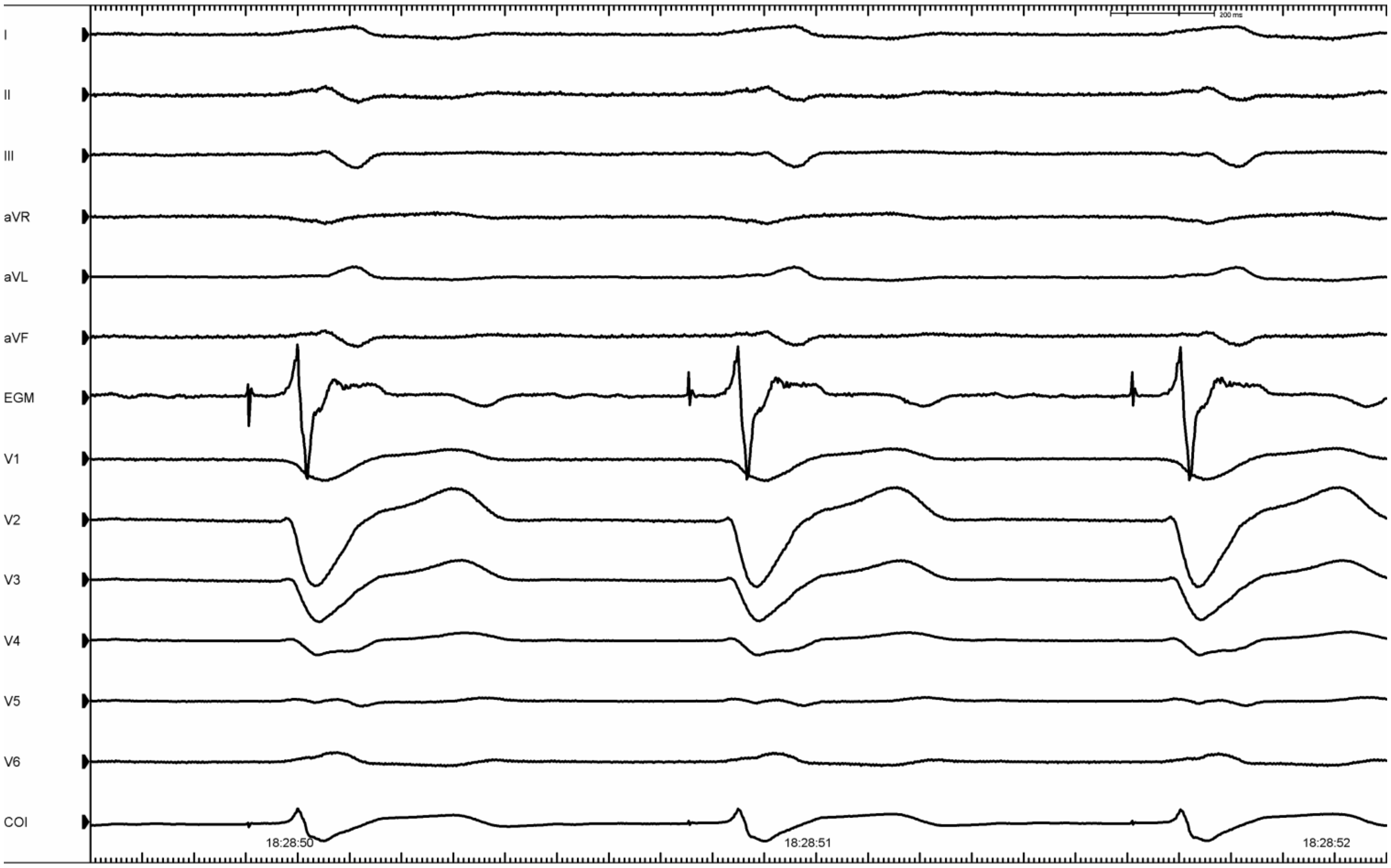

Post ECG

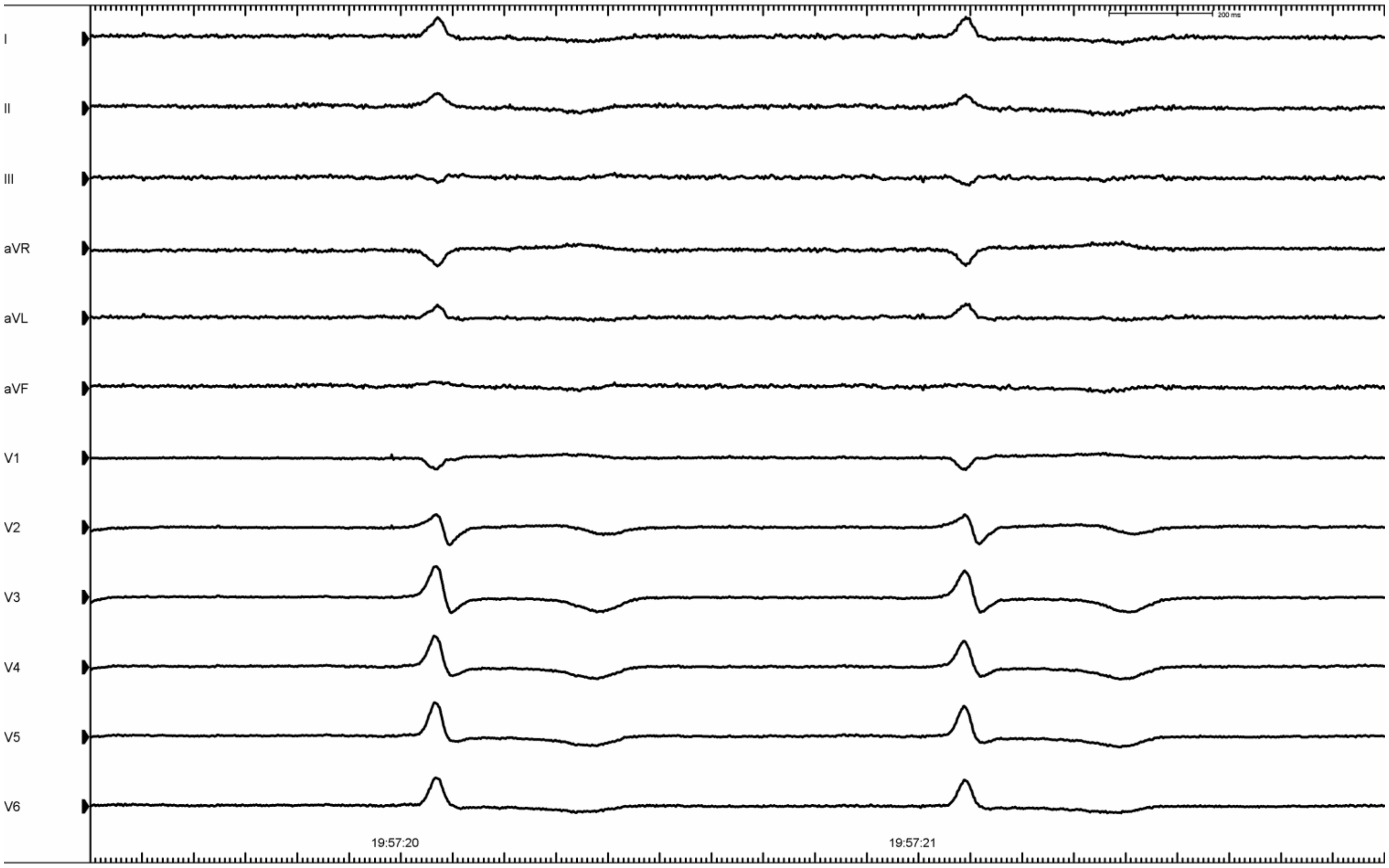

Patient 86:
Transitions

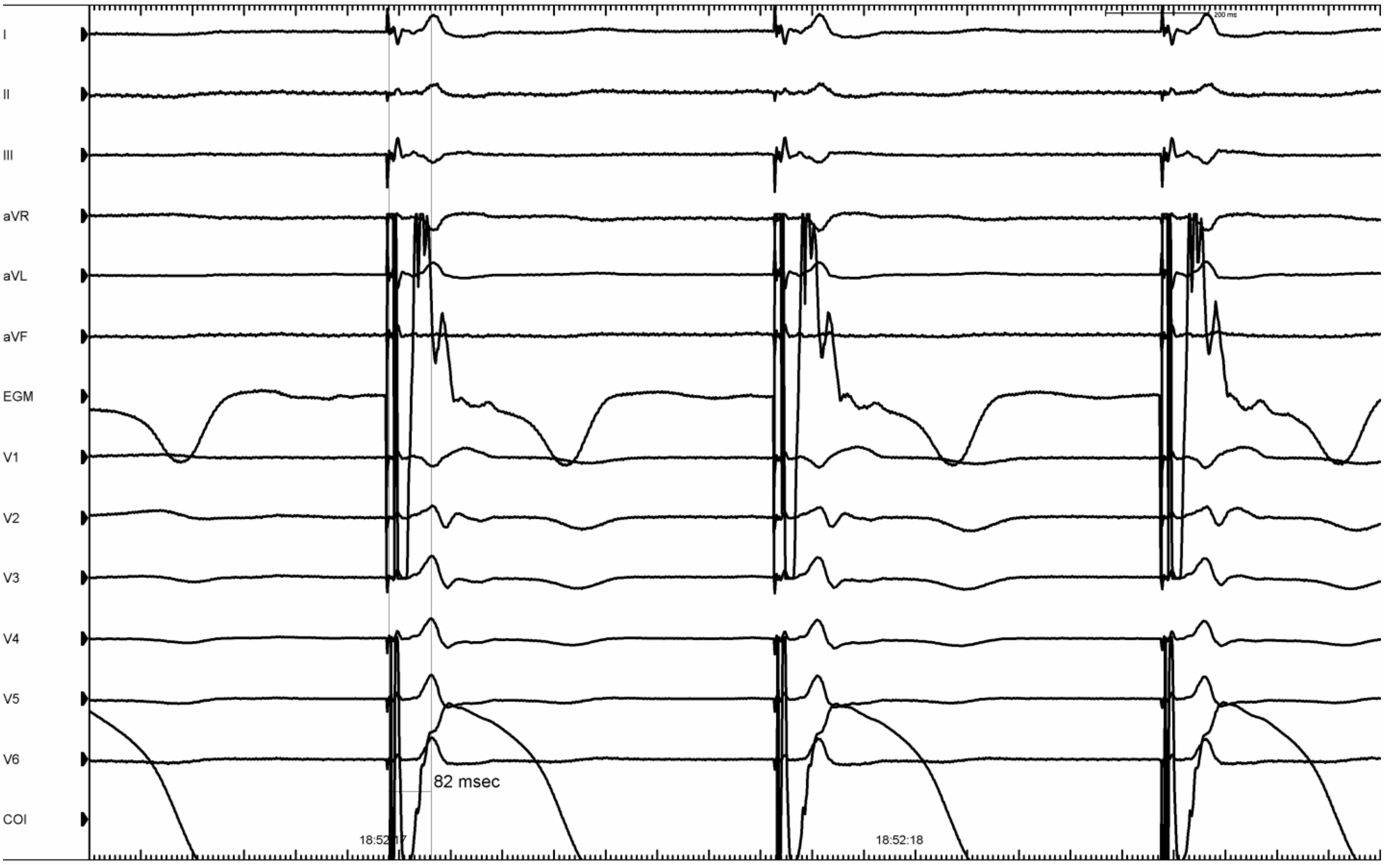

Transitions

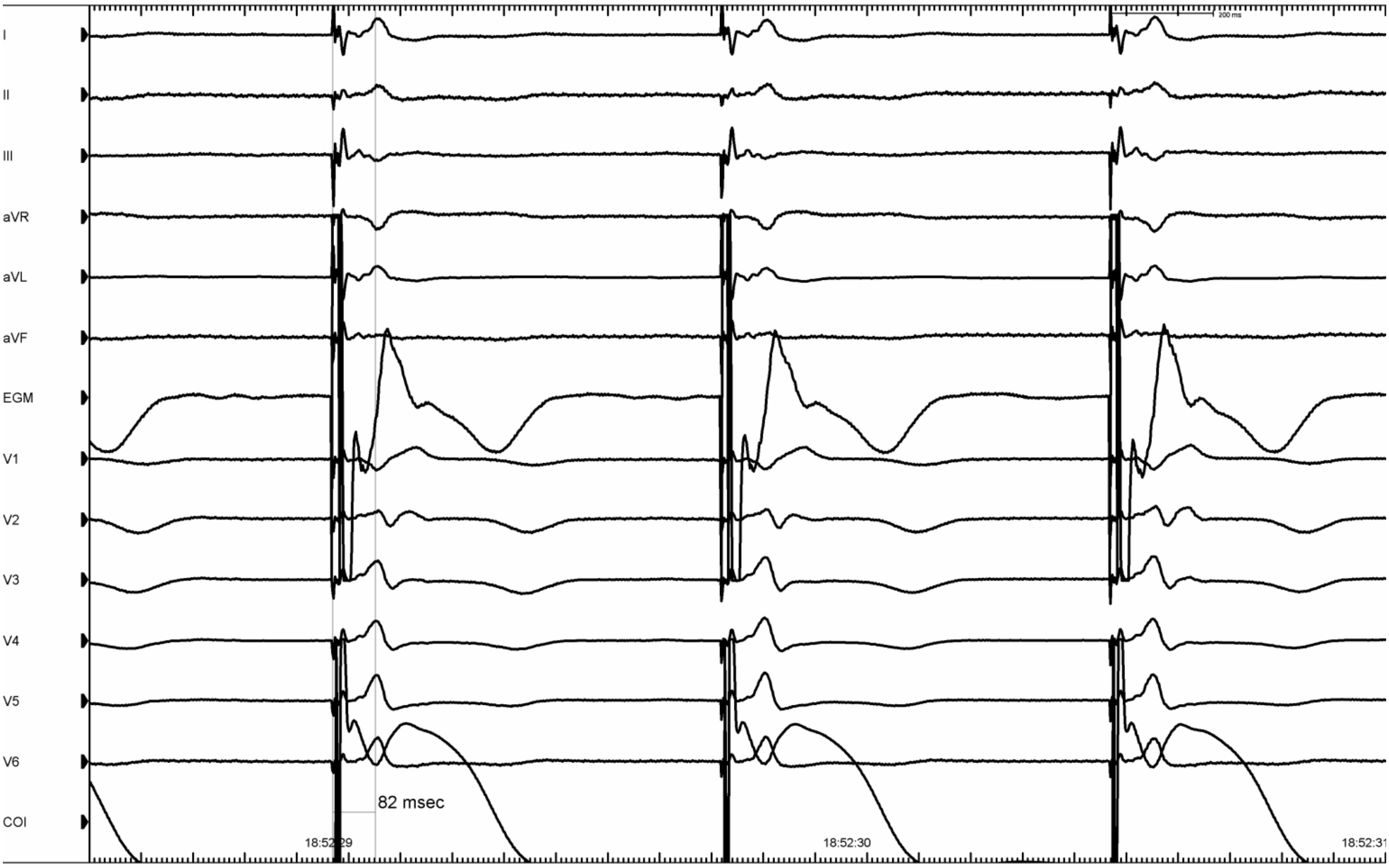

Patient 87:
Pre-ECG

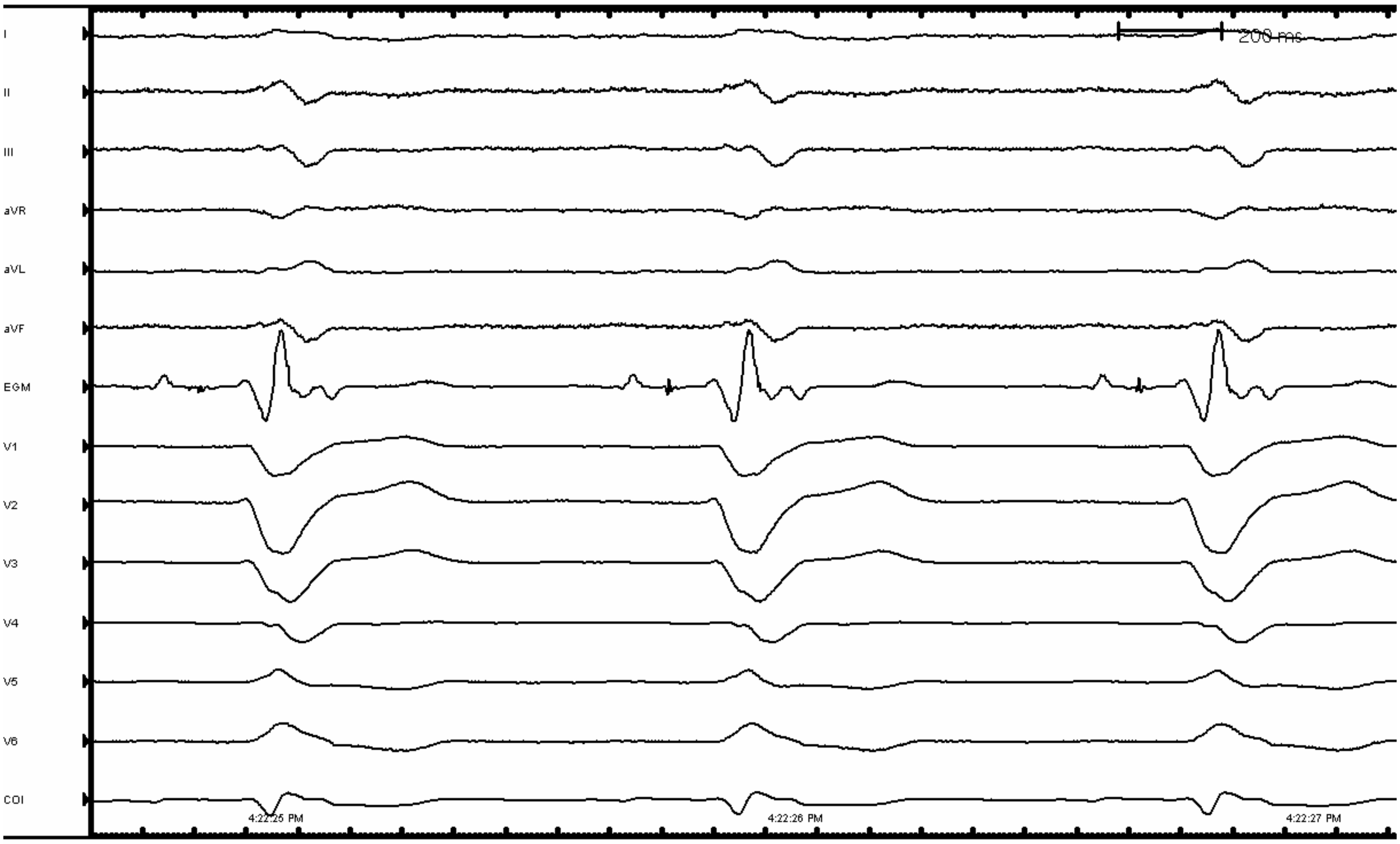

Post ECG

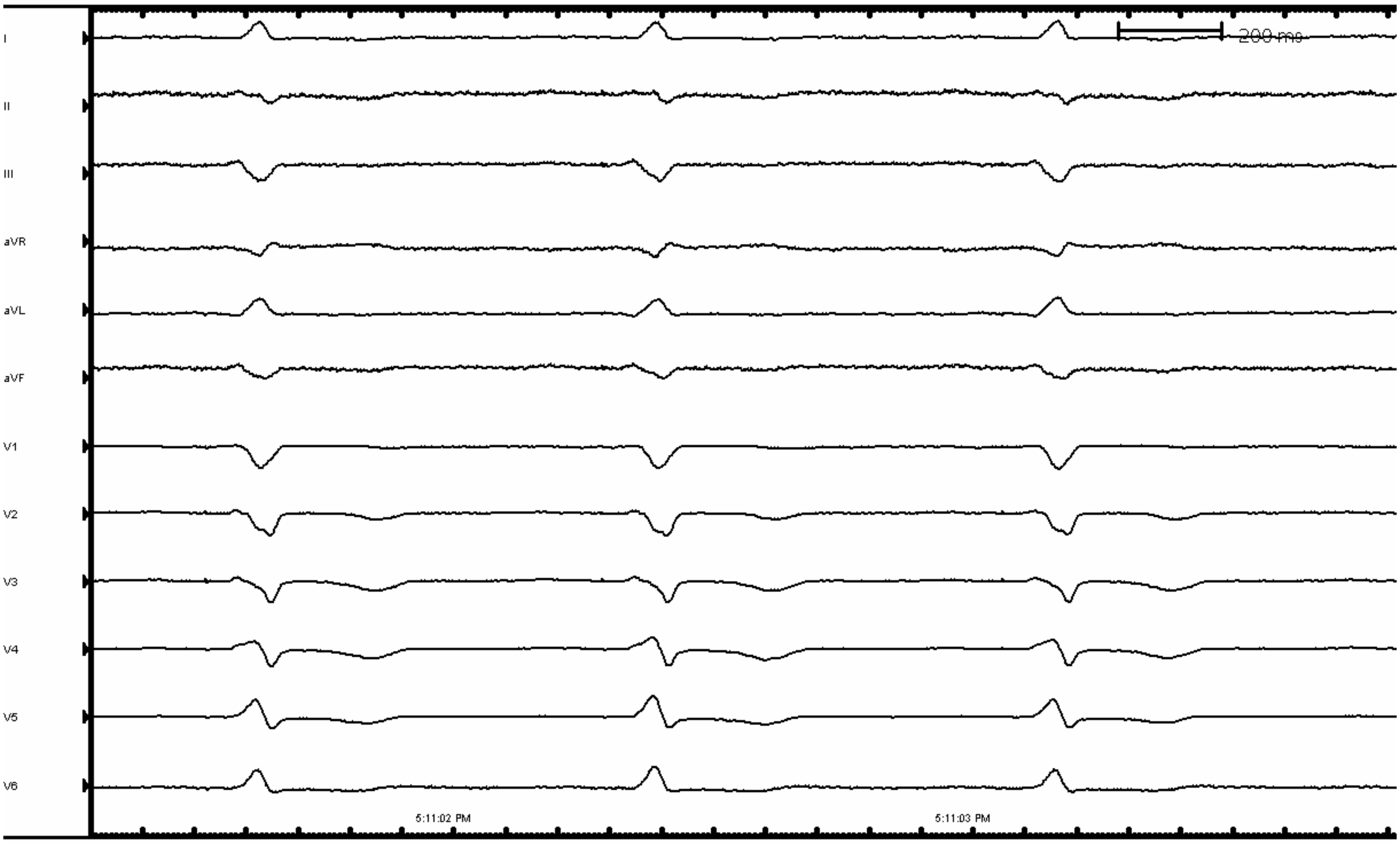

Patient 87:
Transitions

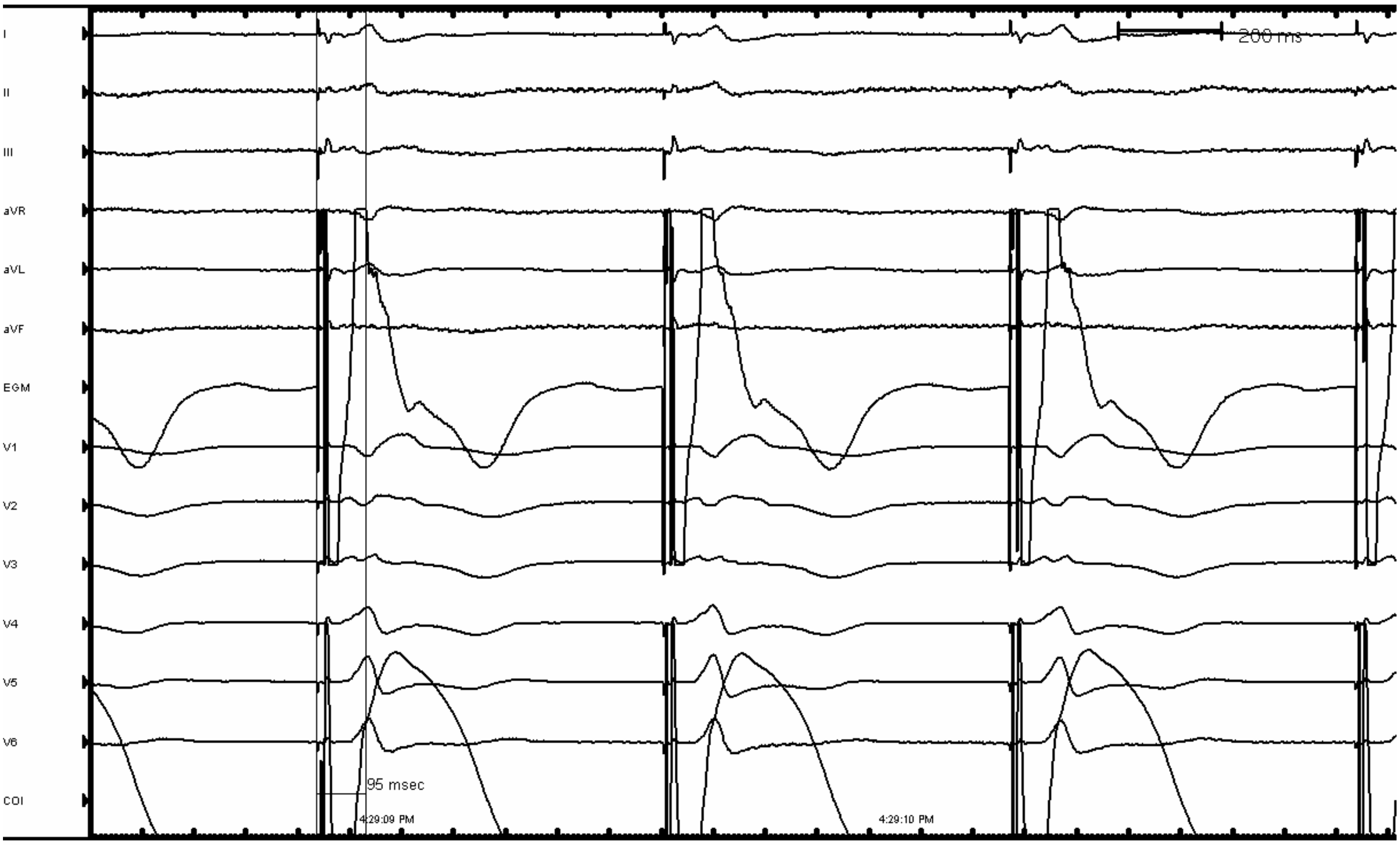

Transitions

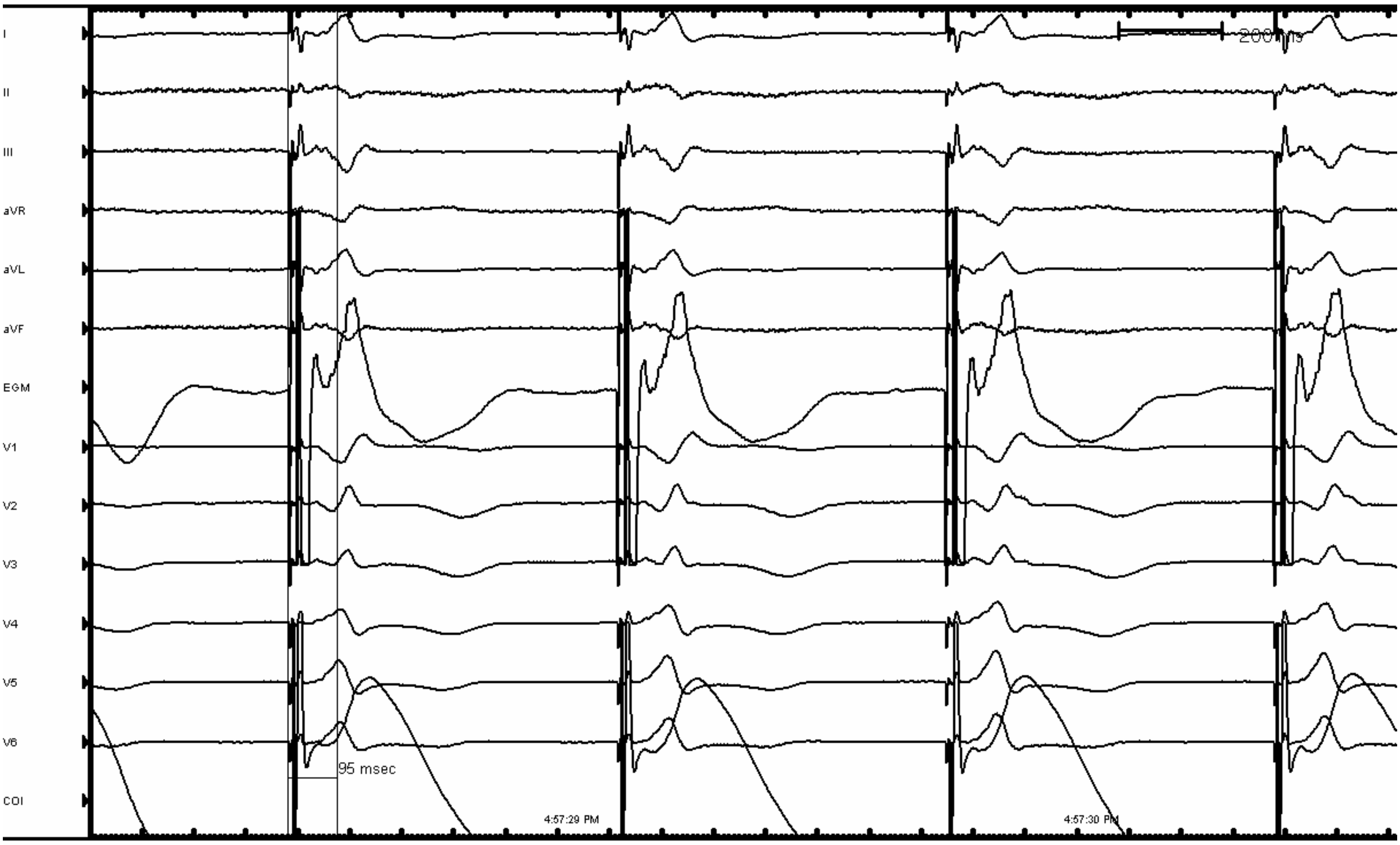

Patient 88:

Pre-ECG

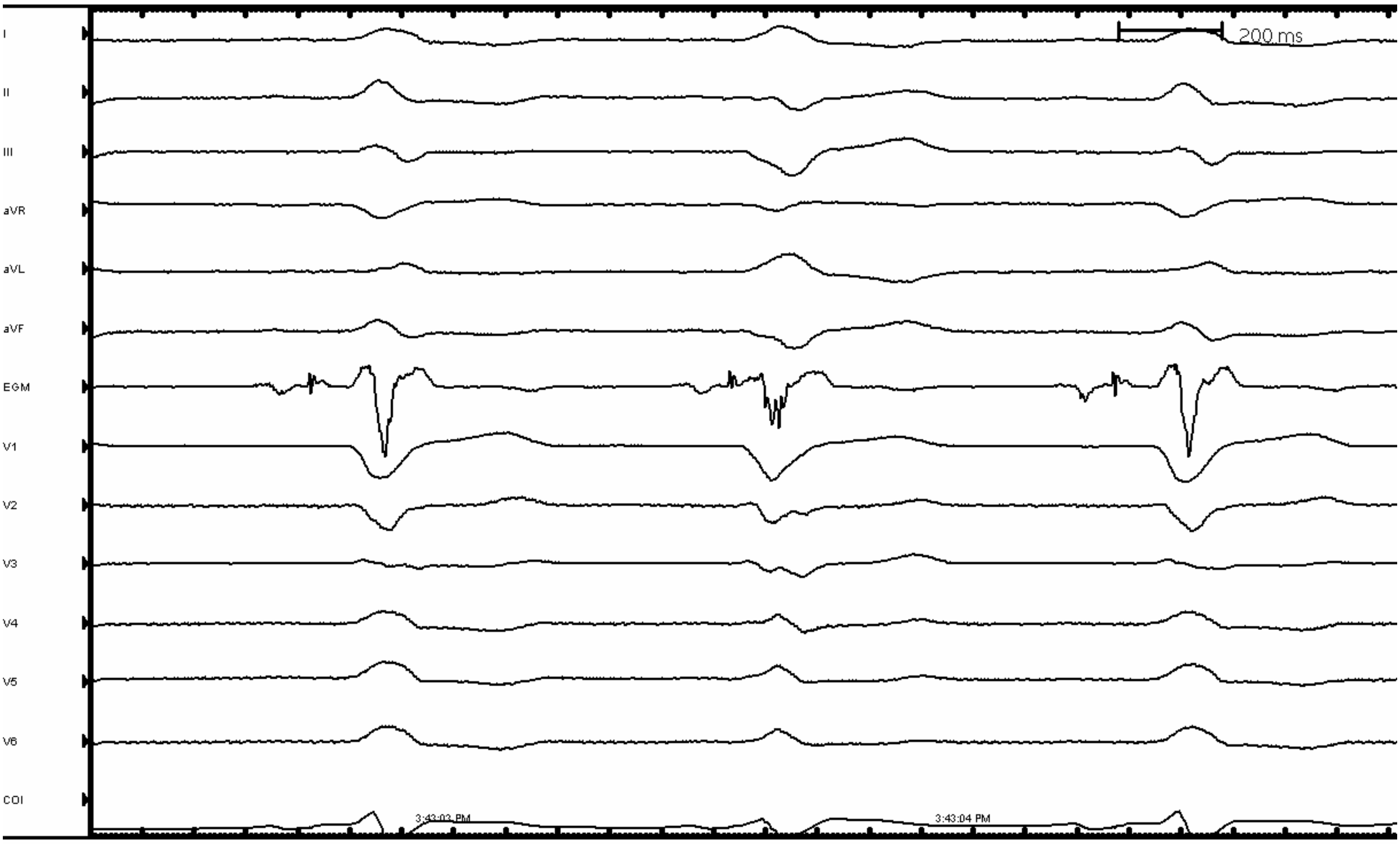

Post ECG

Patient 88:
Transitions

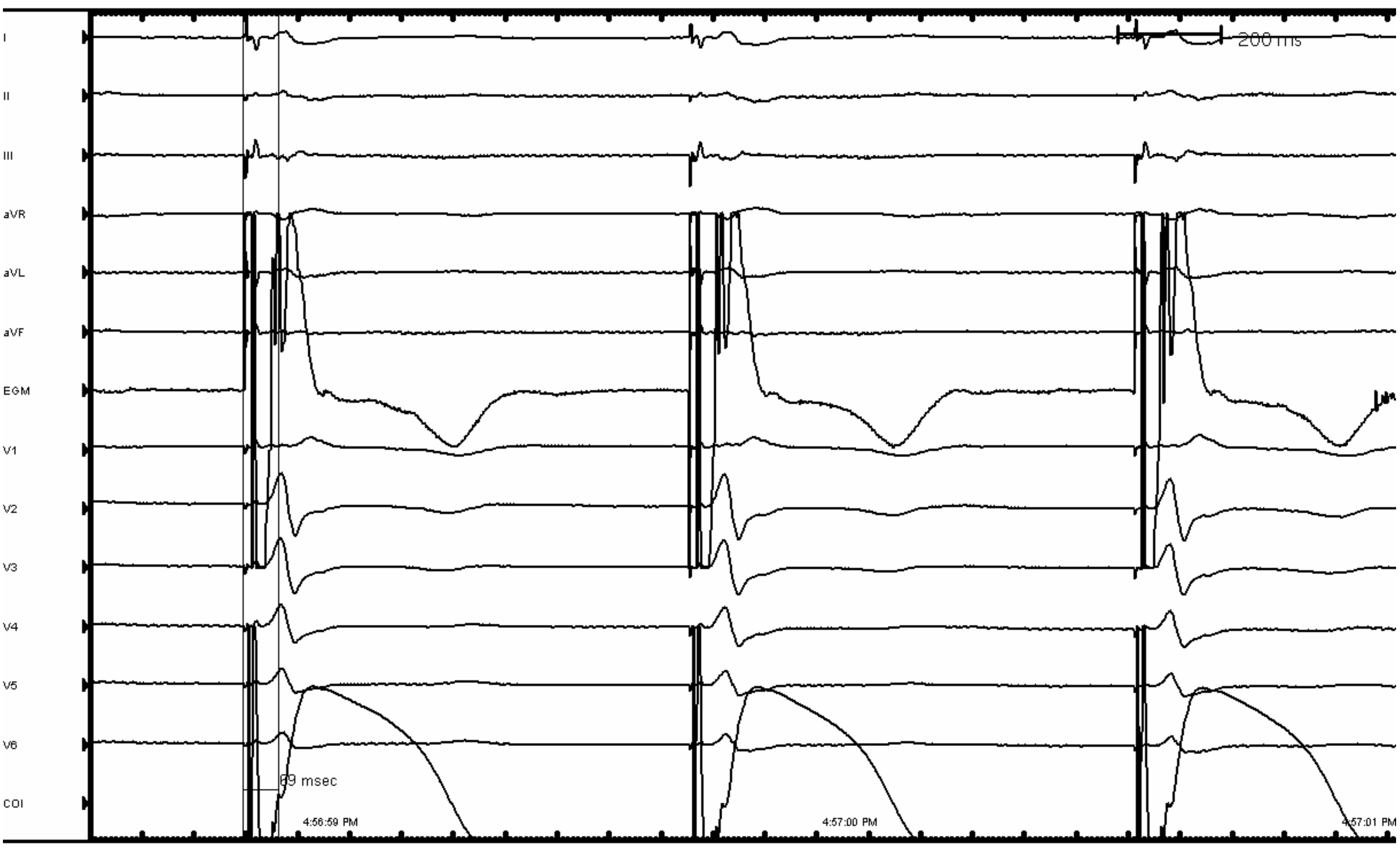

Transitions

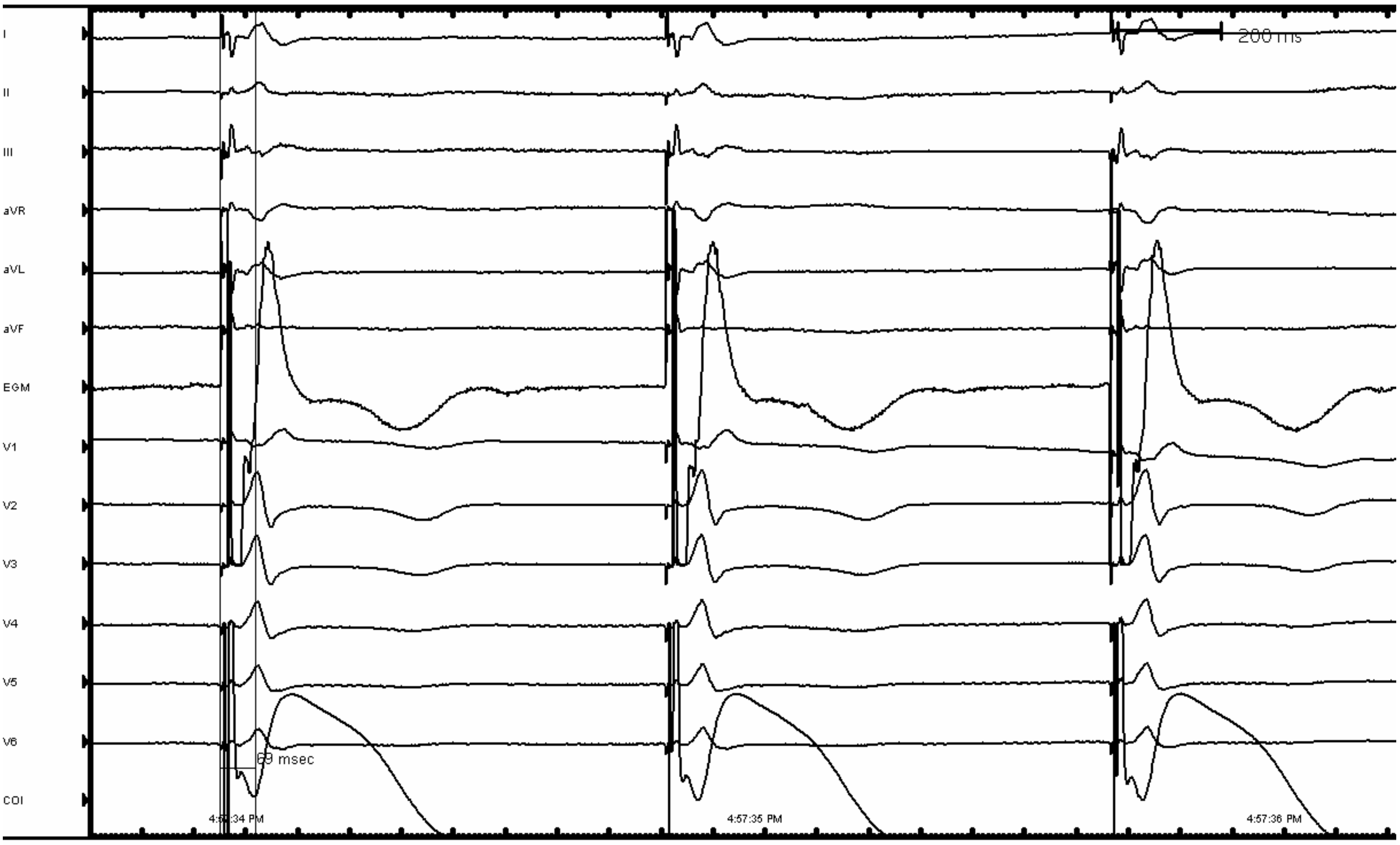

Patient 89:
Pre-ECG

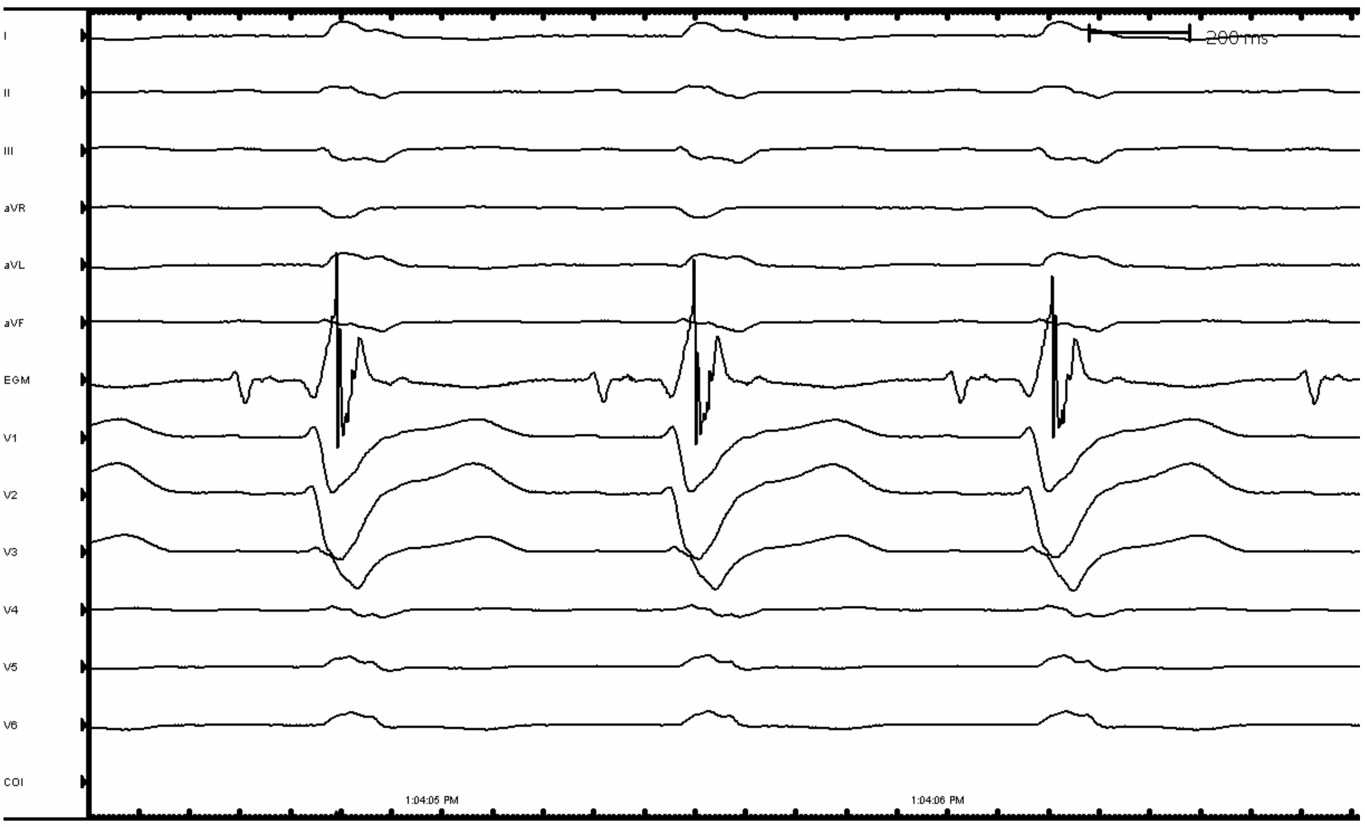

Post ECG

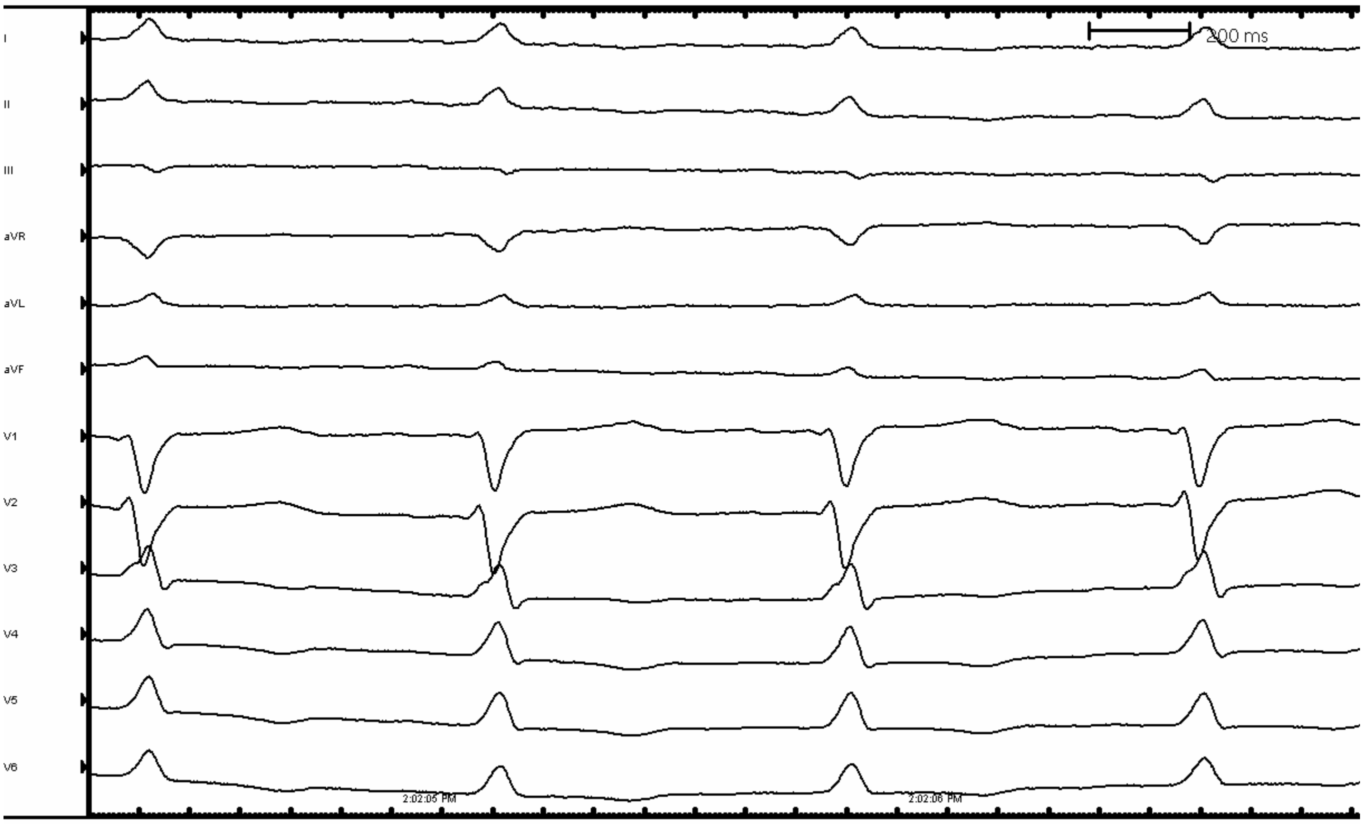

Patient 89:
Transitions

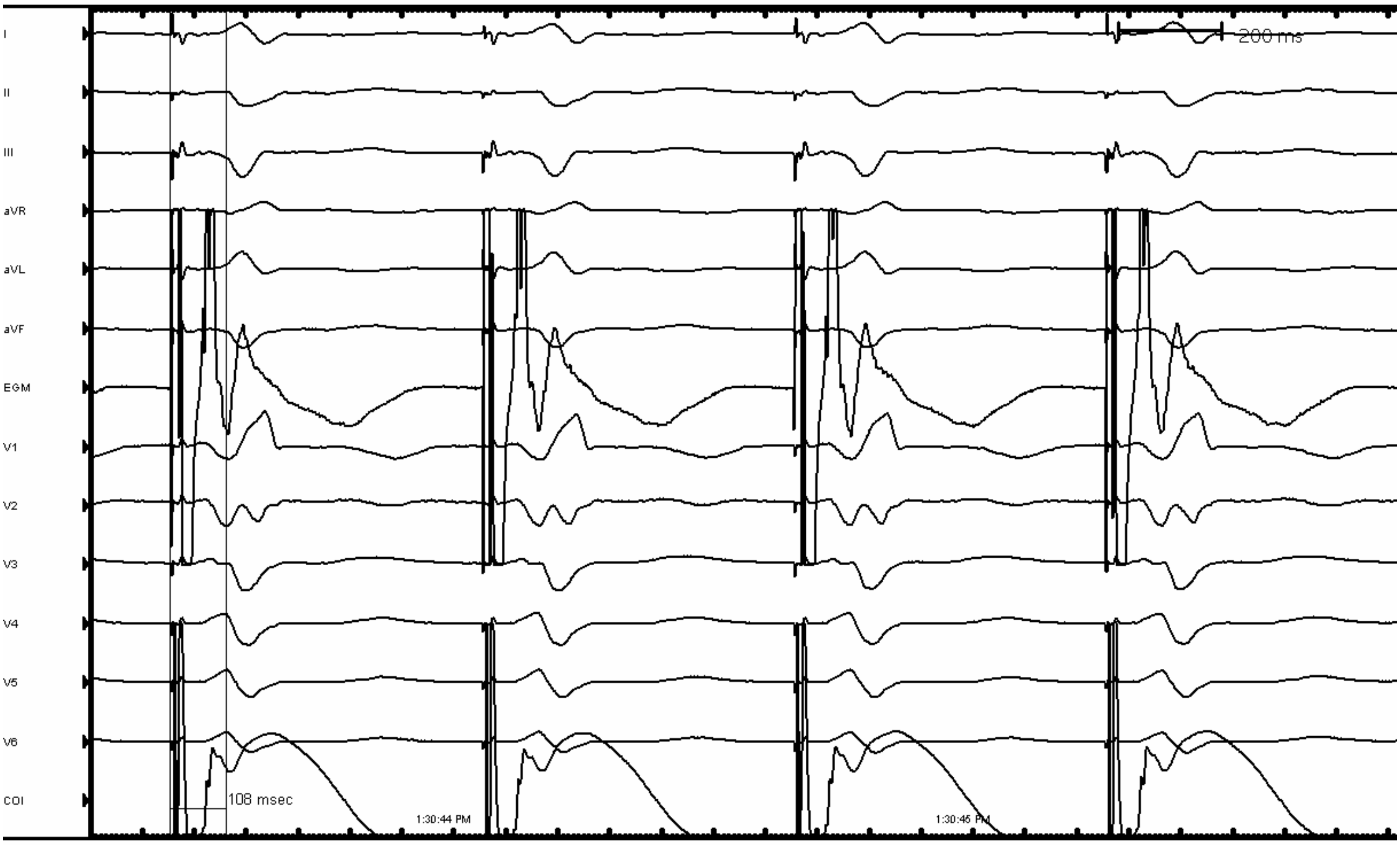

Transitions

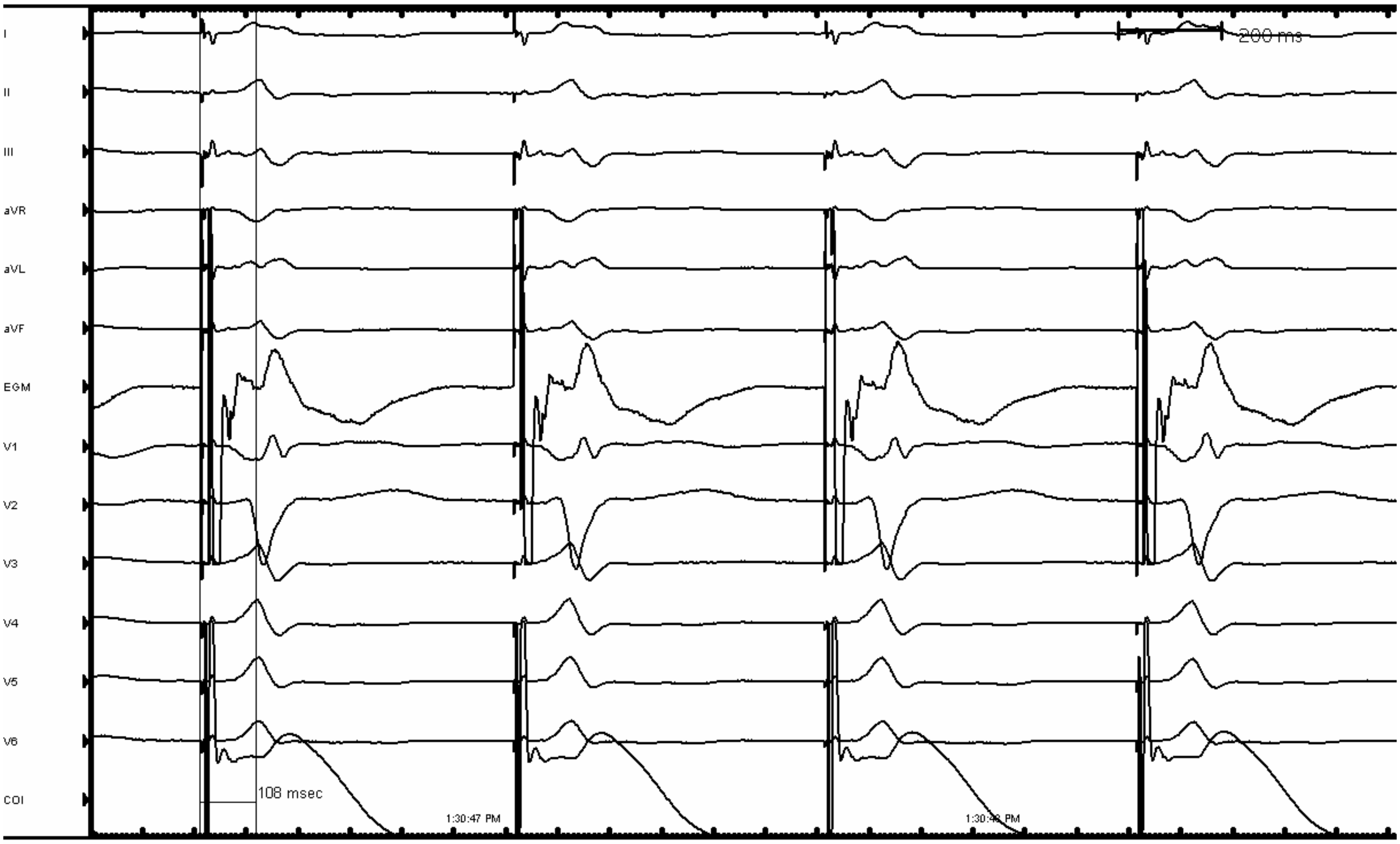

Patient 90:
Pre-ECG

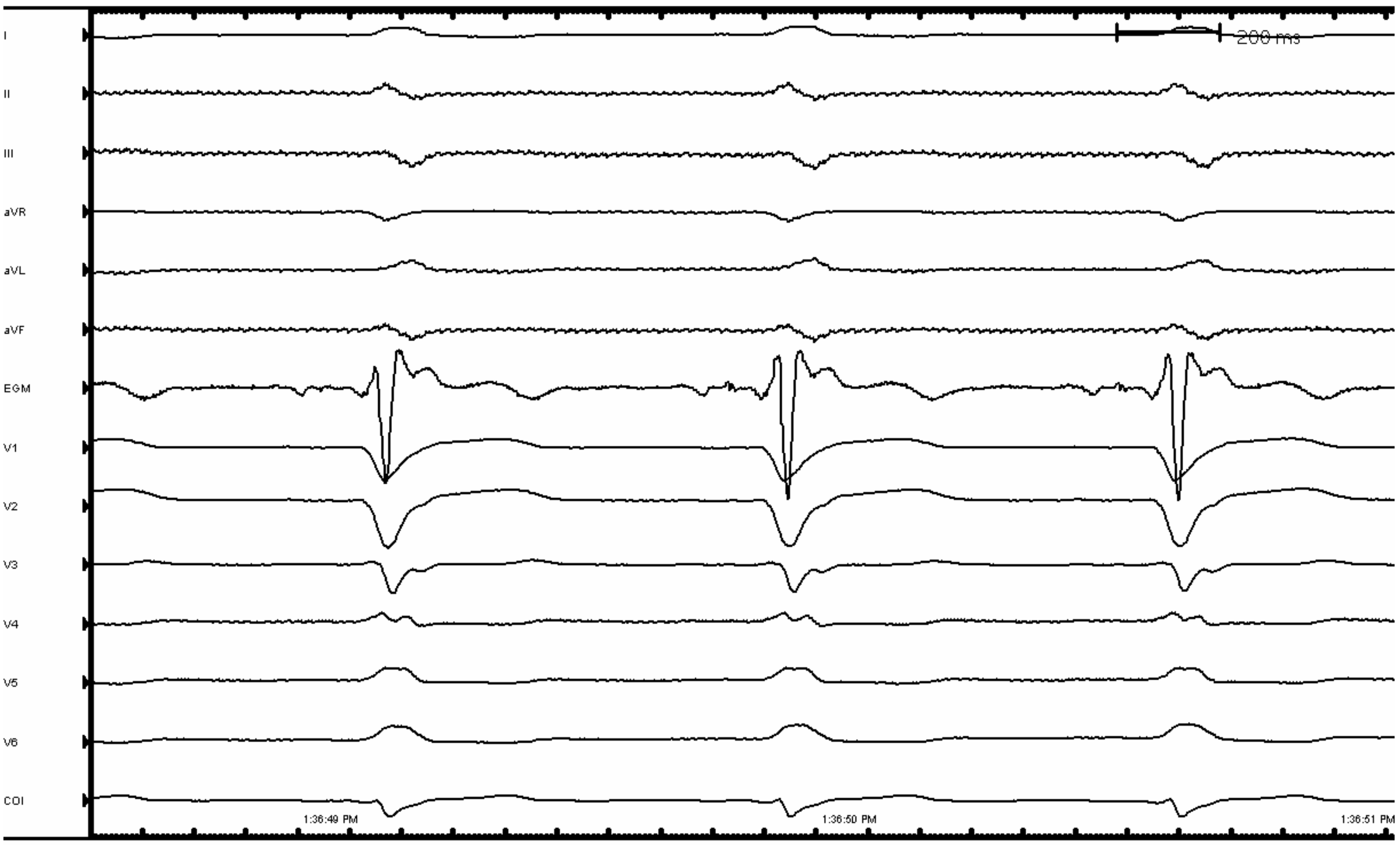

Post ECG

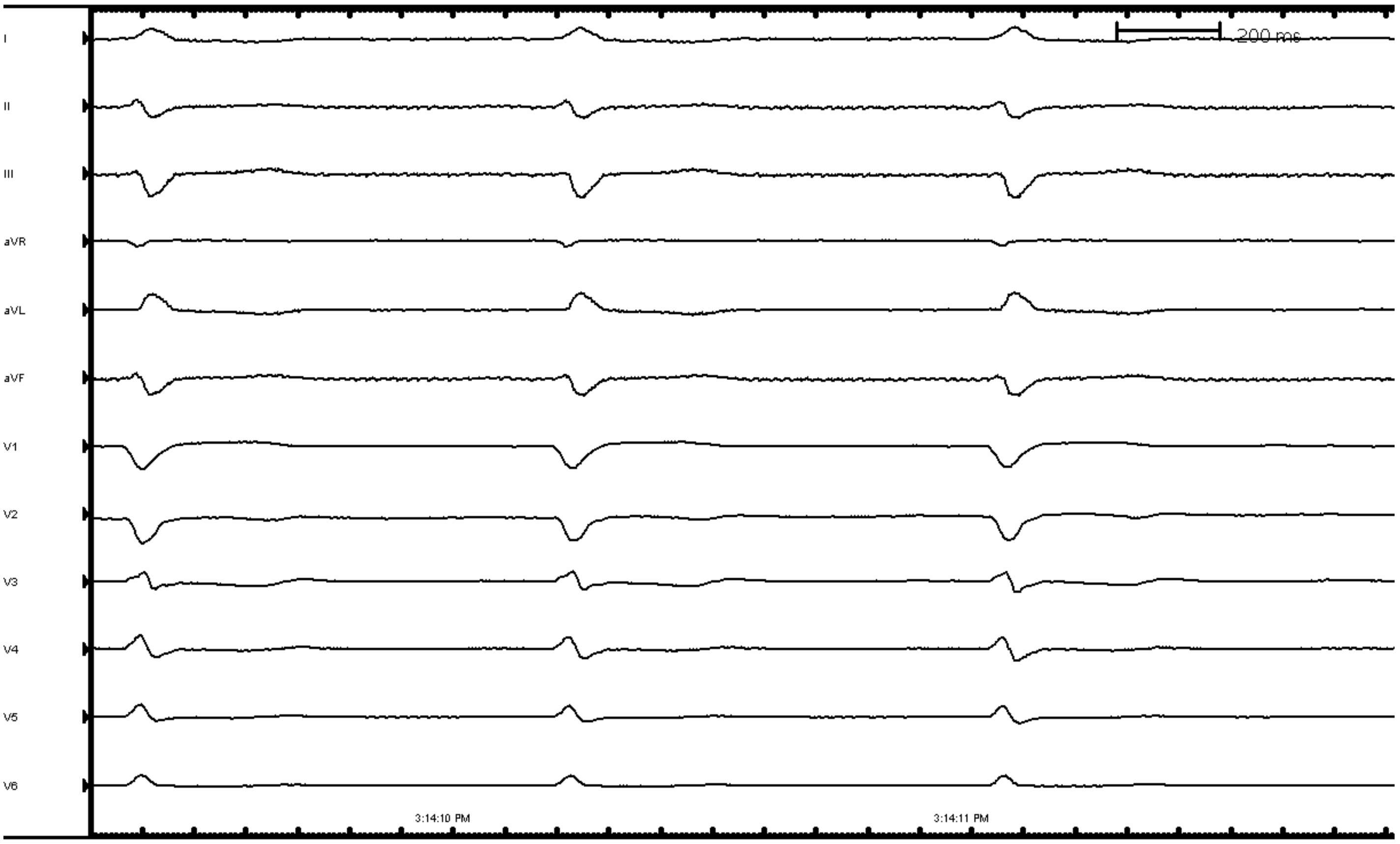

Patient 90:
Transitions

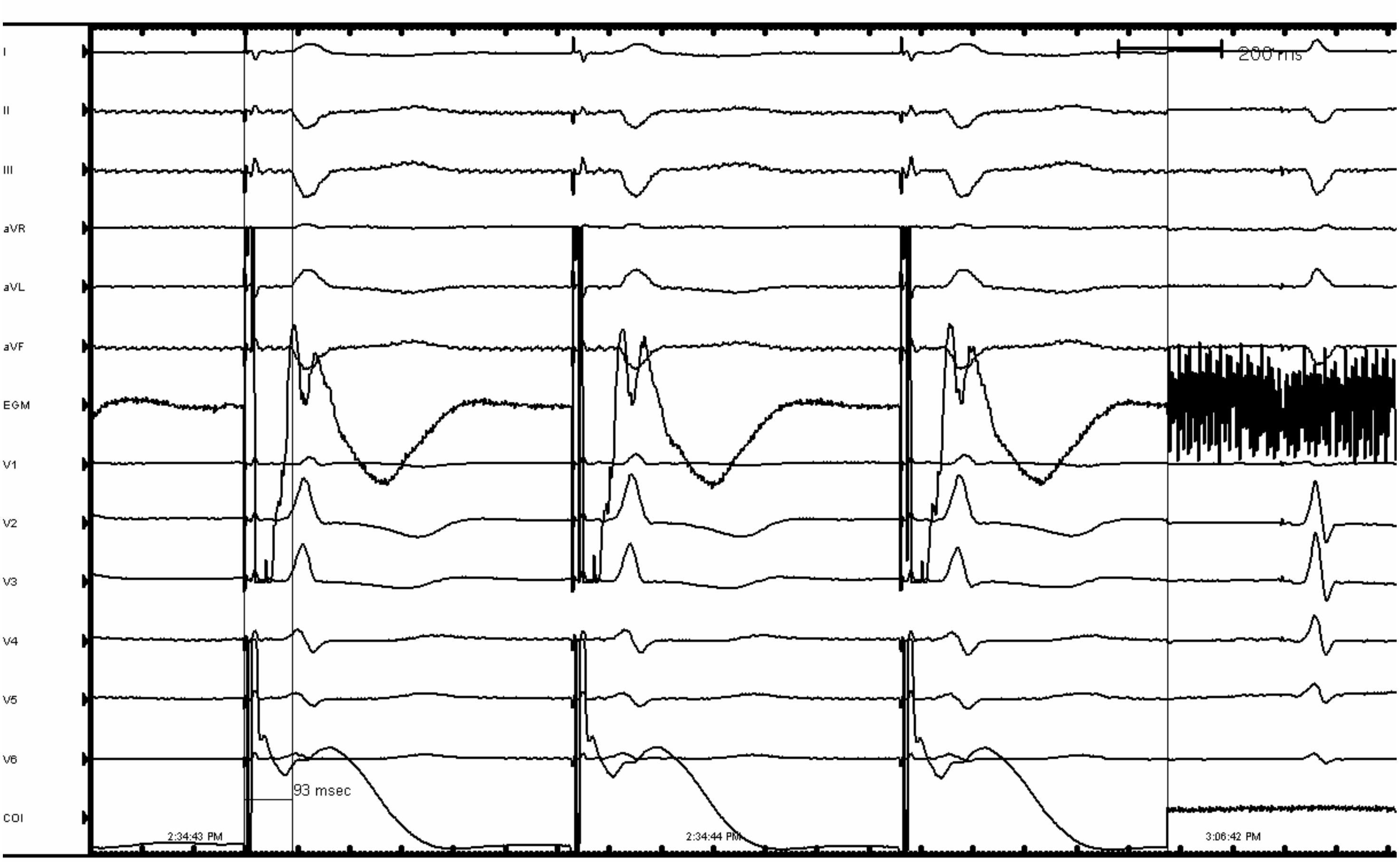

Transitions

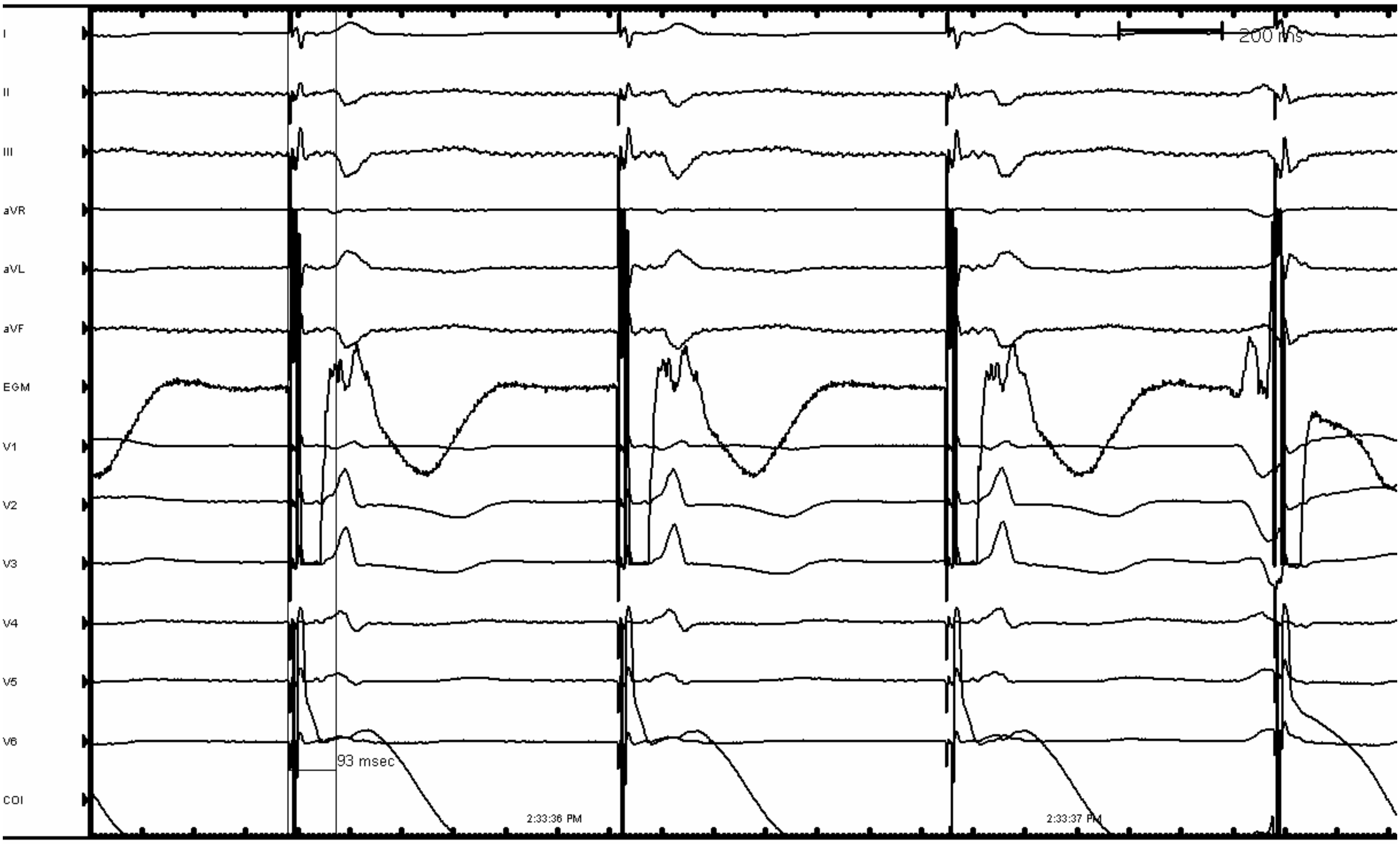

Patient 91:
Pre-ECG

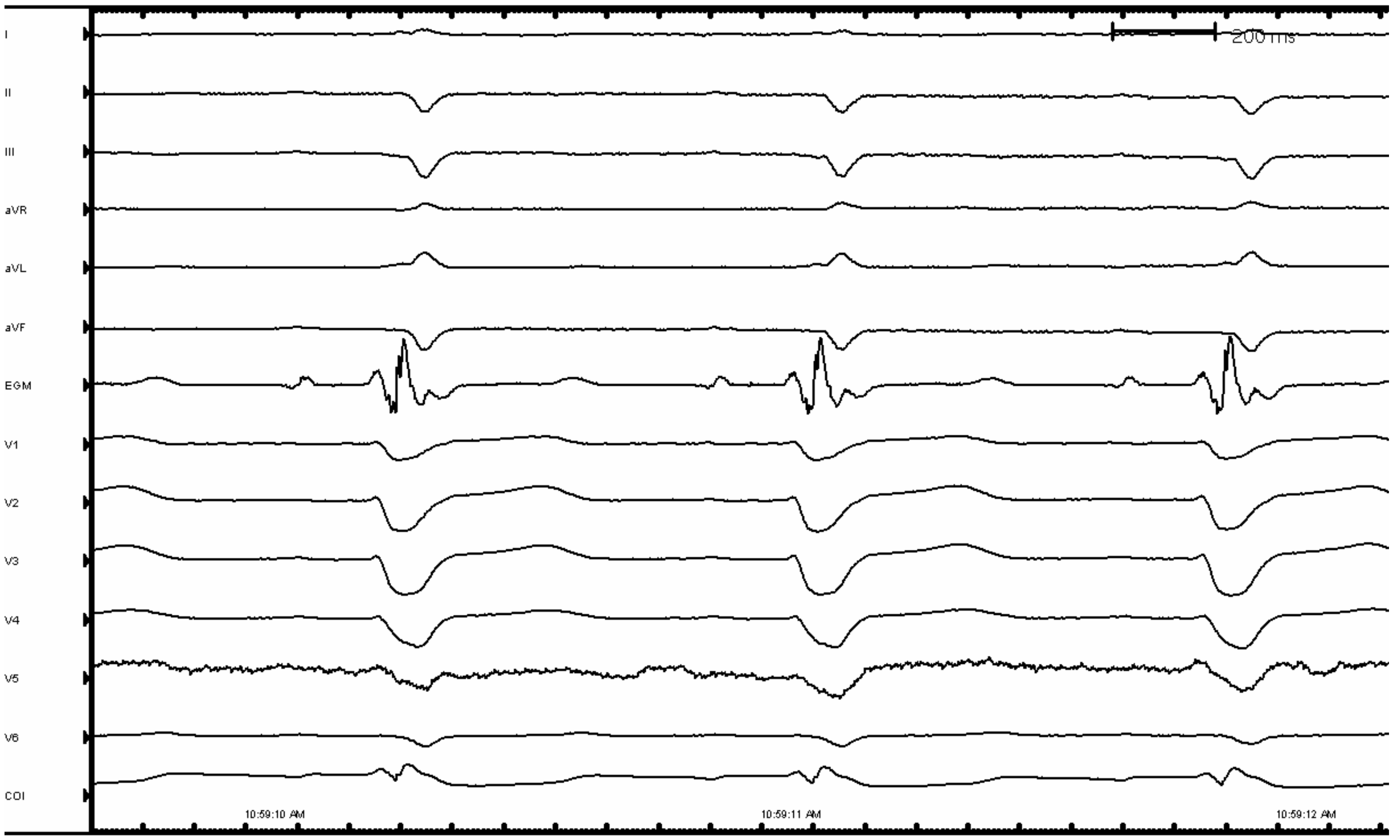

Post ECG

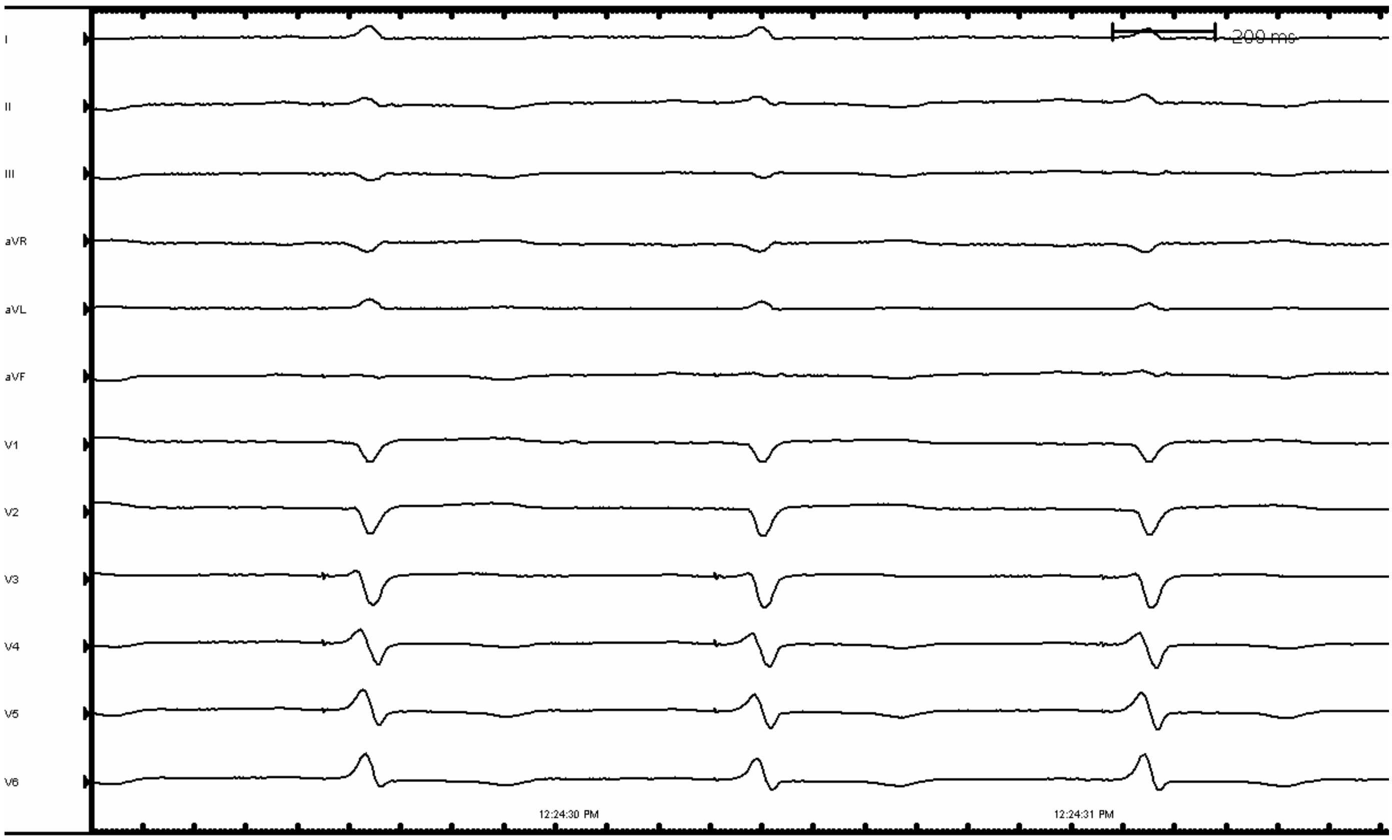

Patient 91:
Transitions

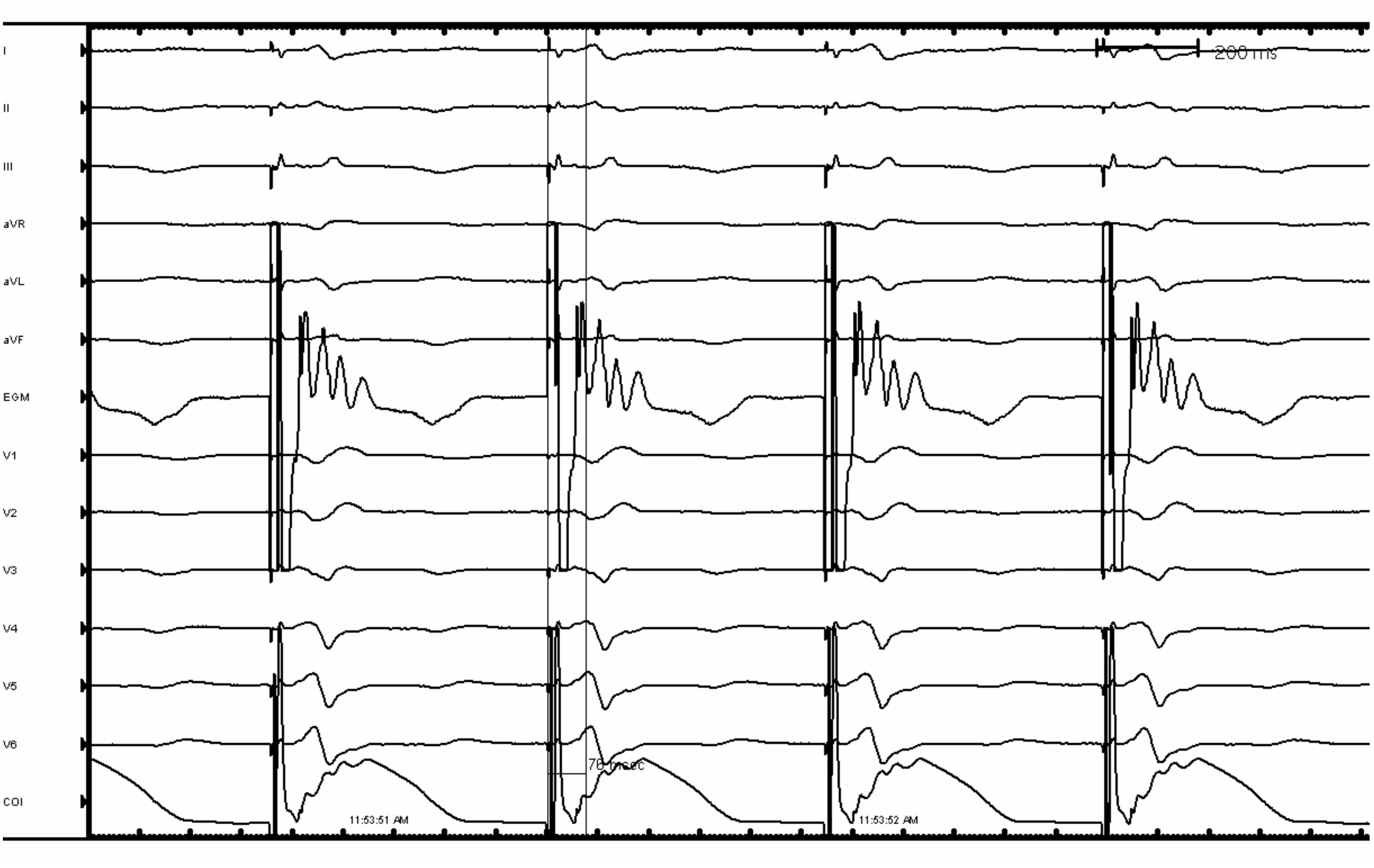

Transitions

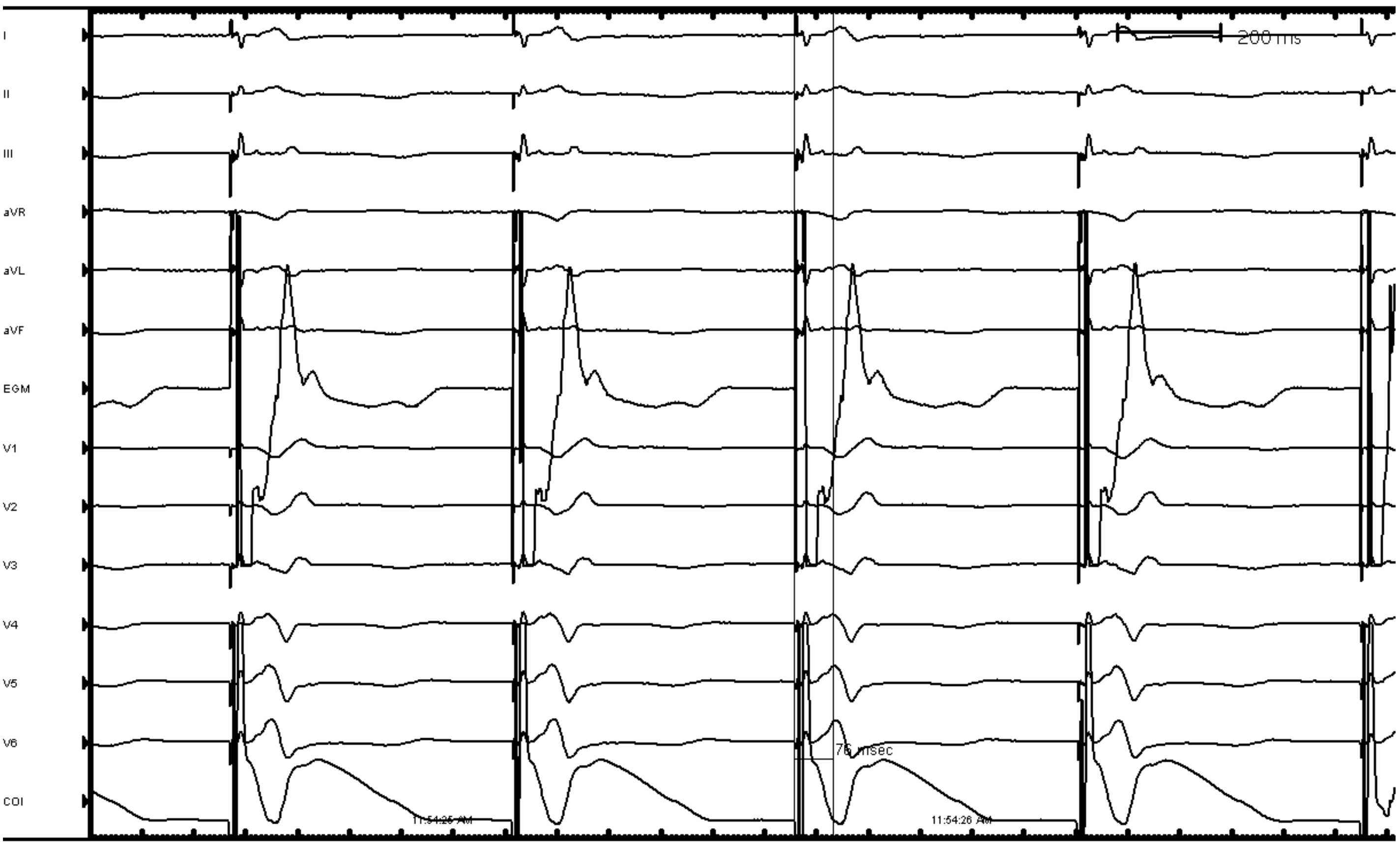

Patient 92:
Pre-ECG

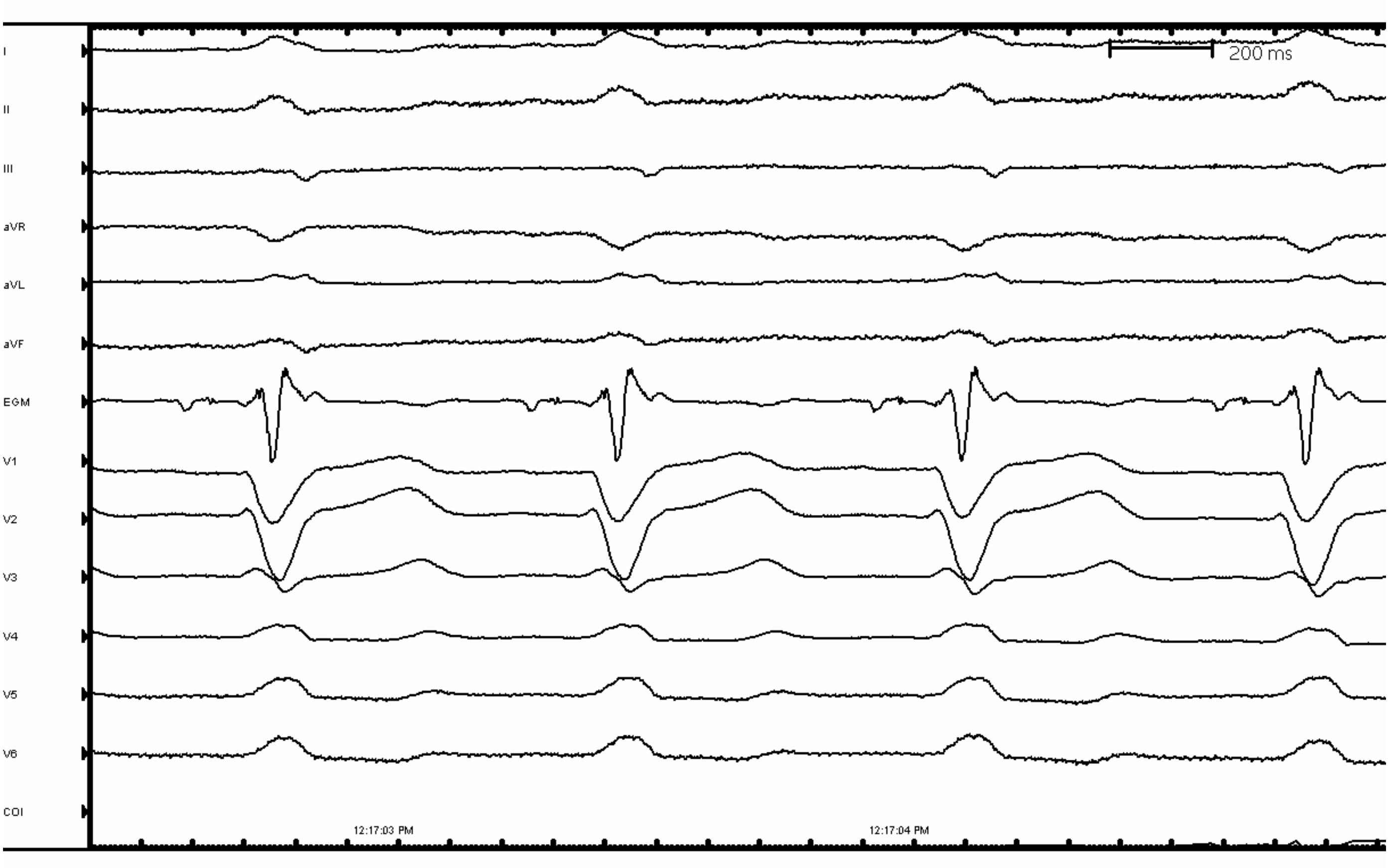

Post ECG

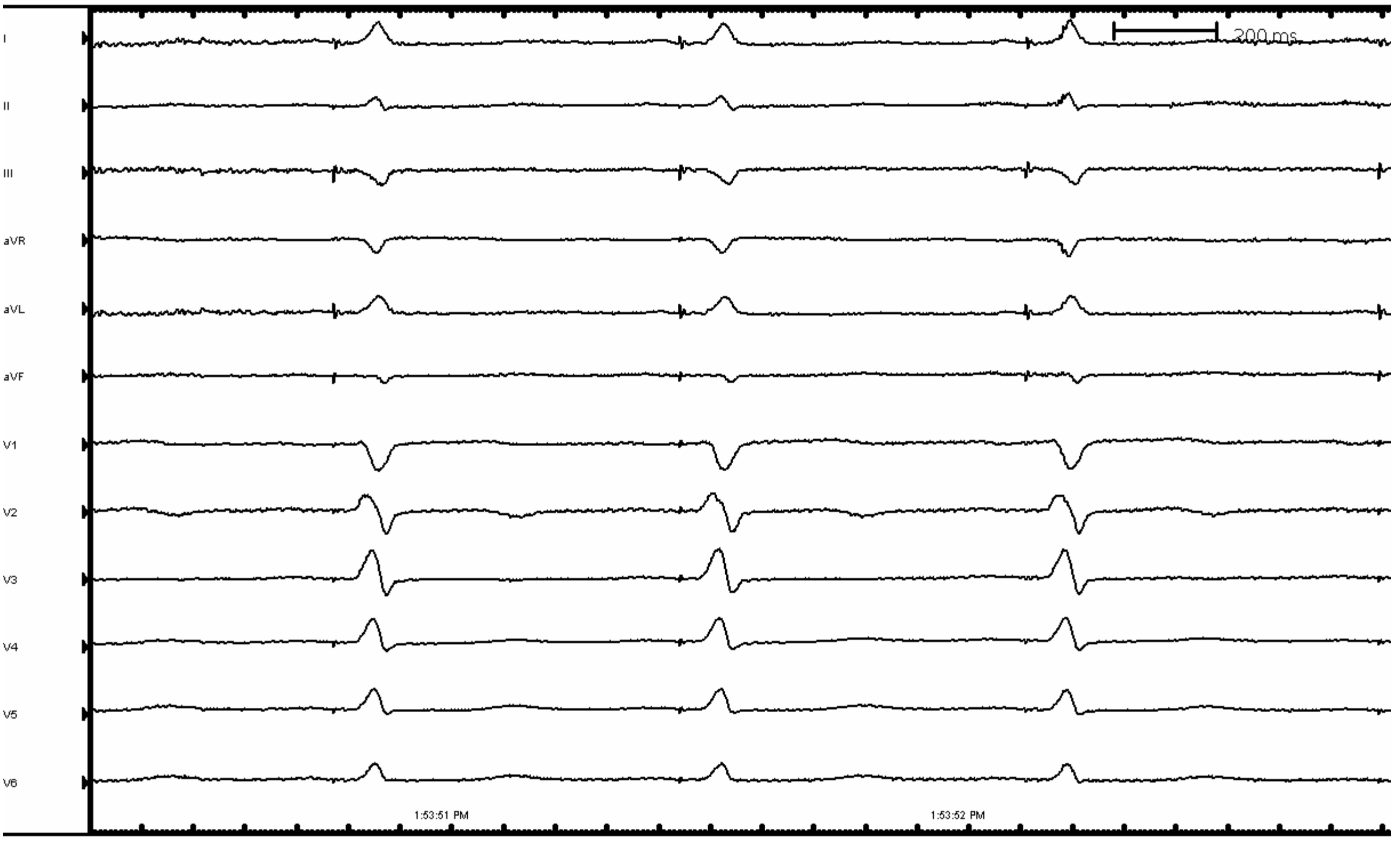

Patient 92:
Transitions

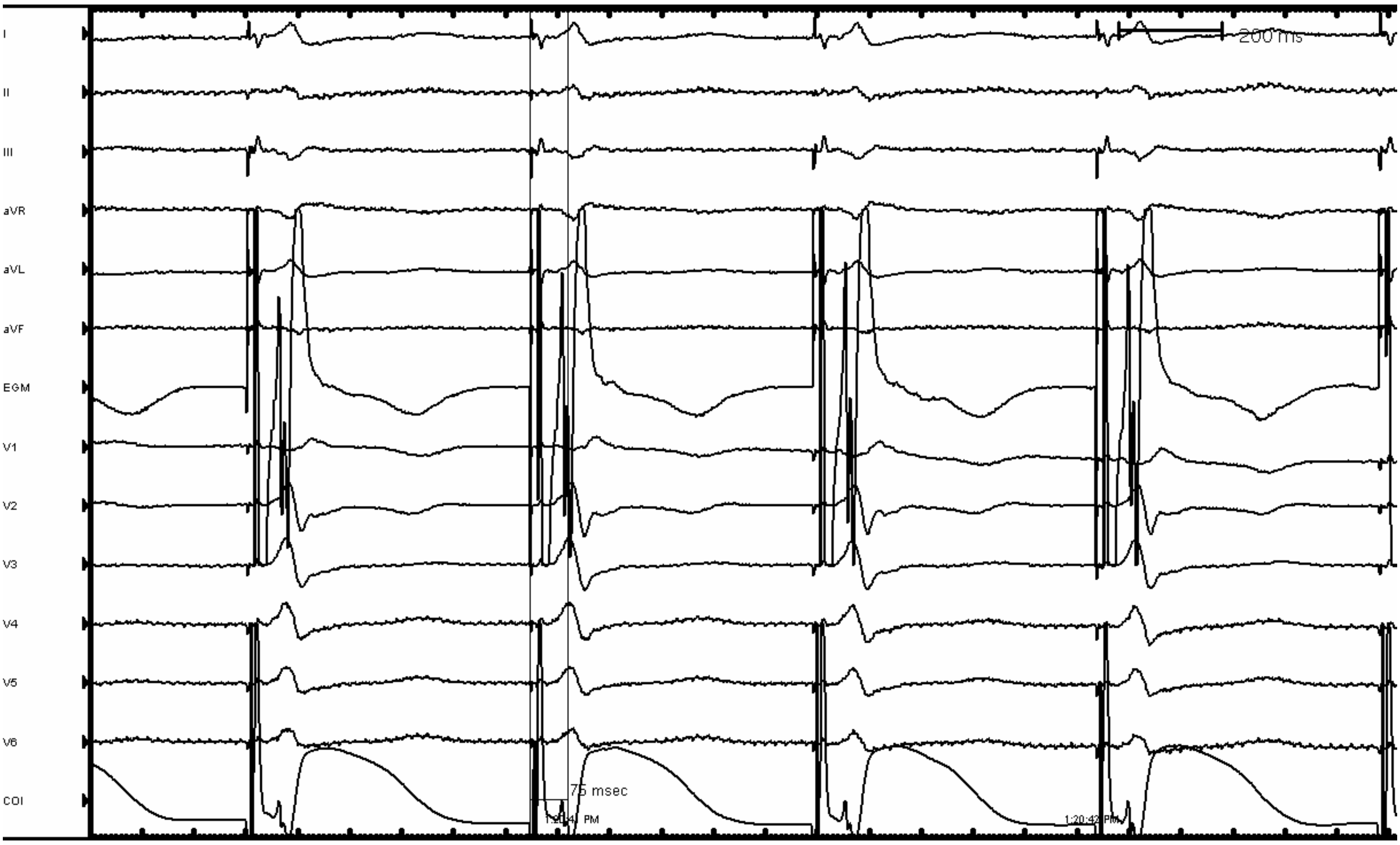

Transitions

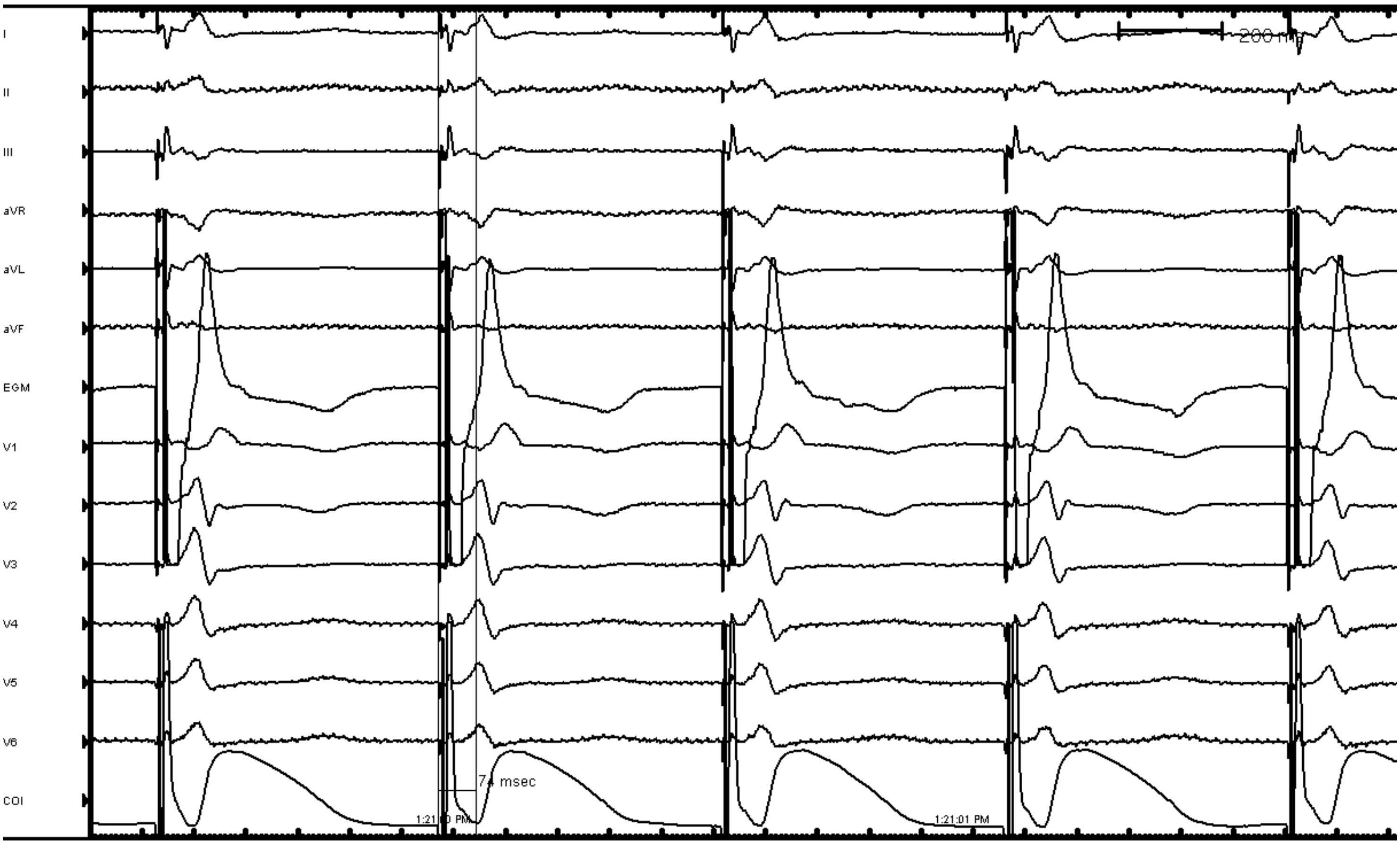

Patient 93:
Pre-ECG

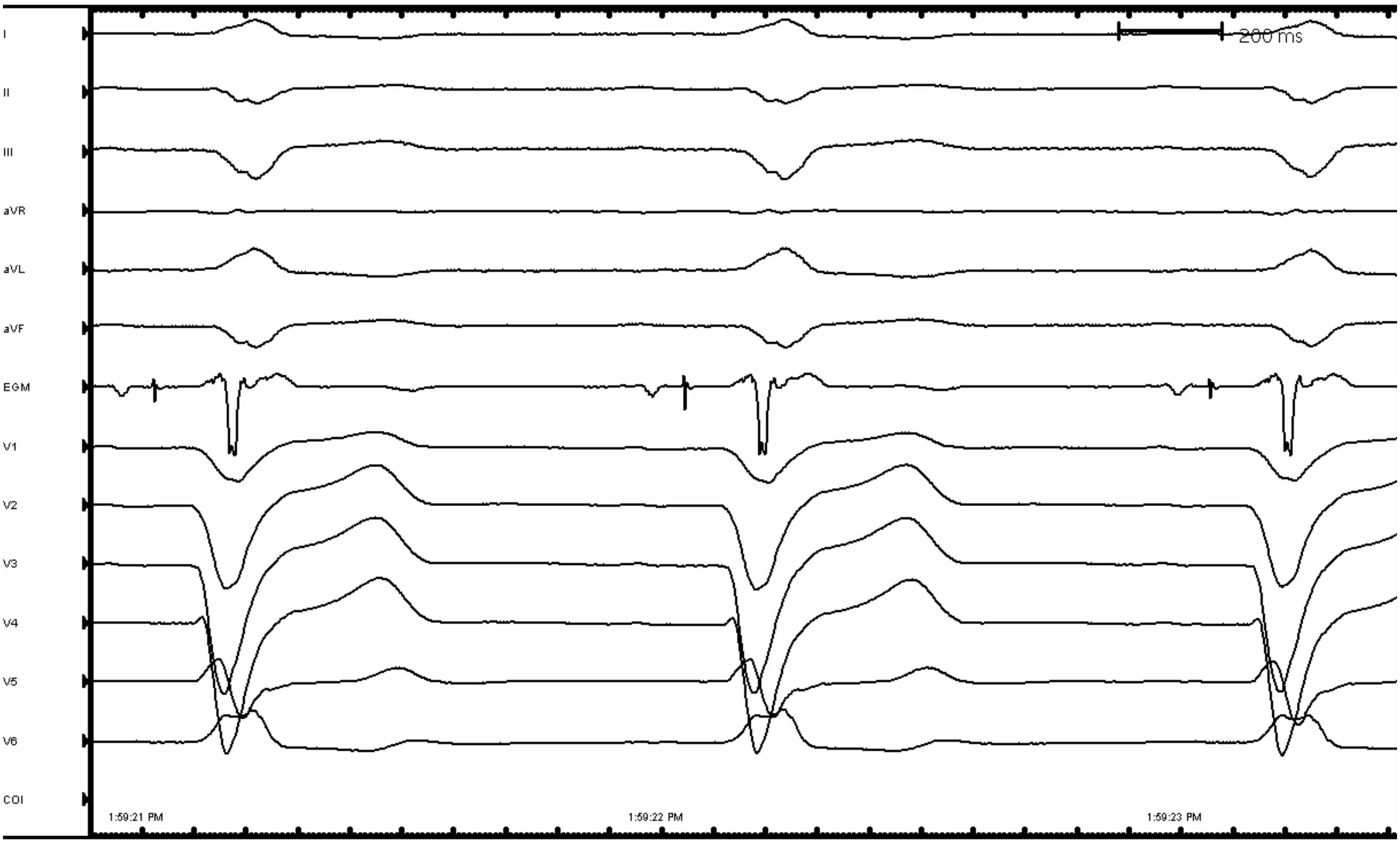

Post ECG

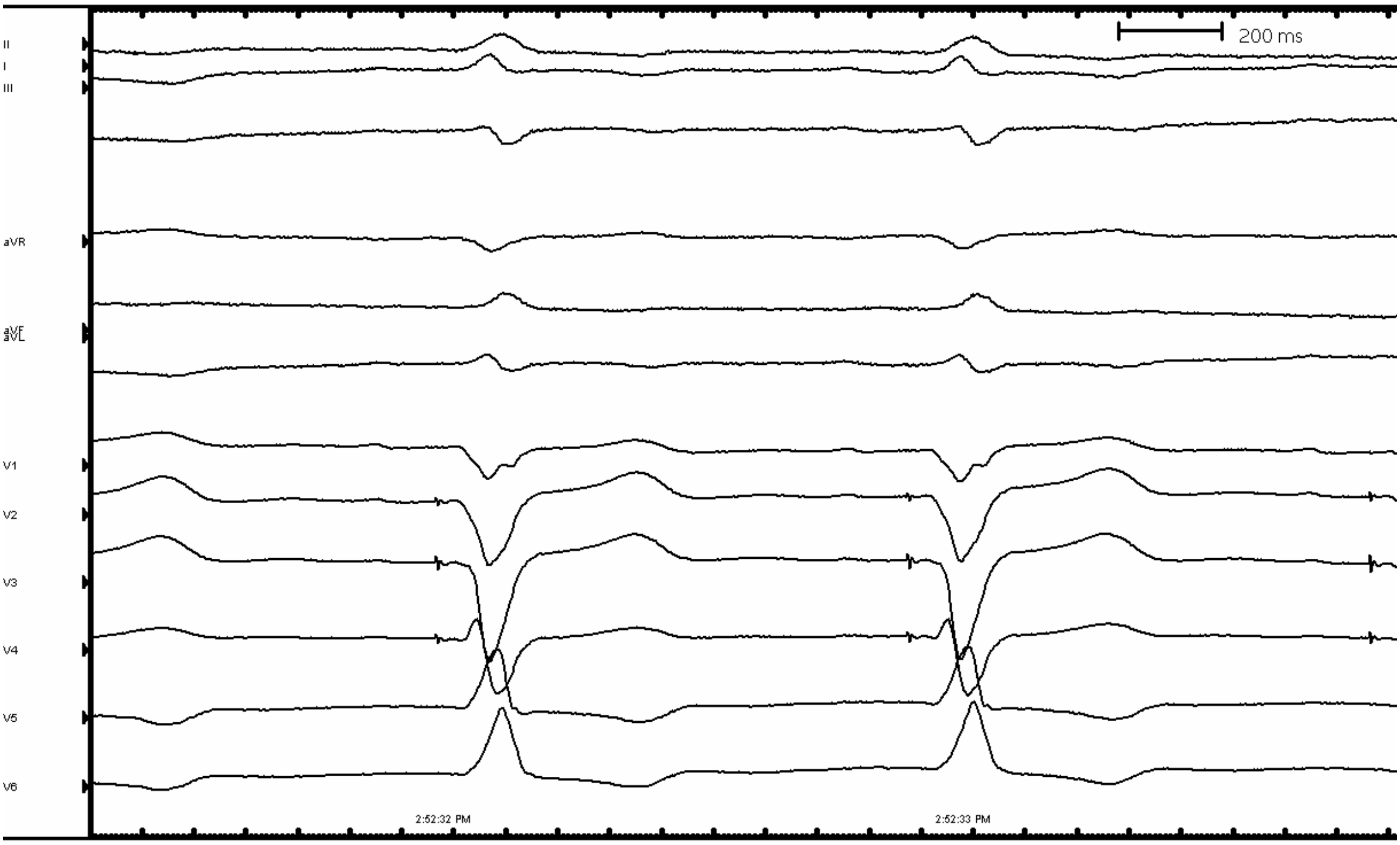

Patient 93:
Transitions

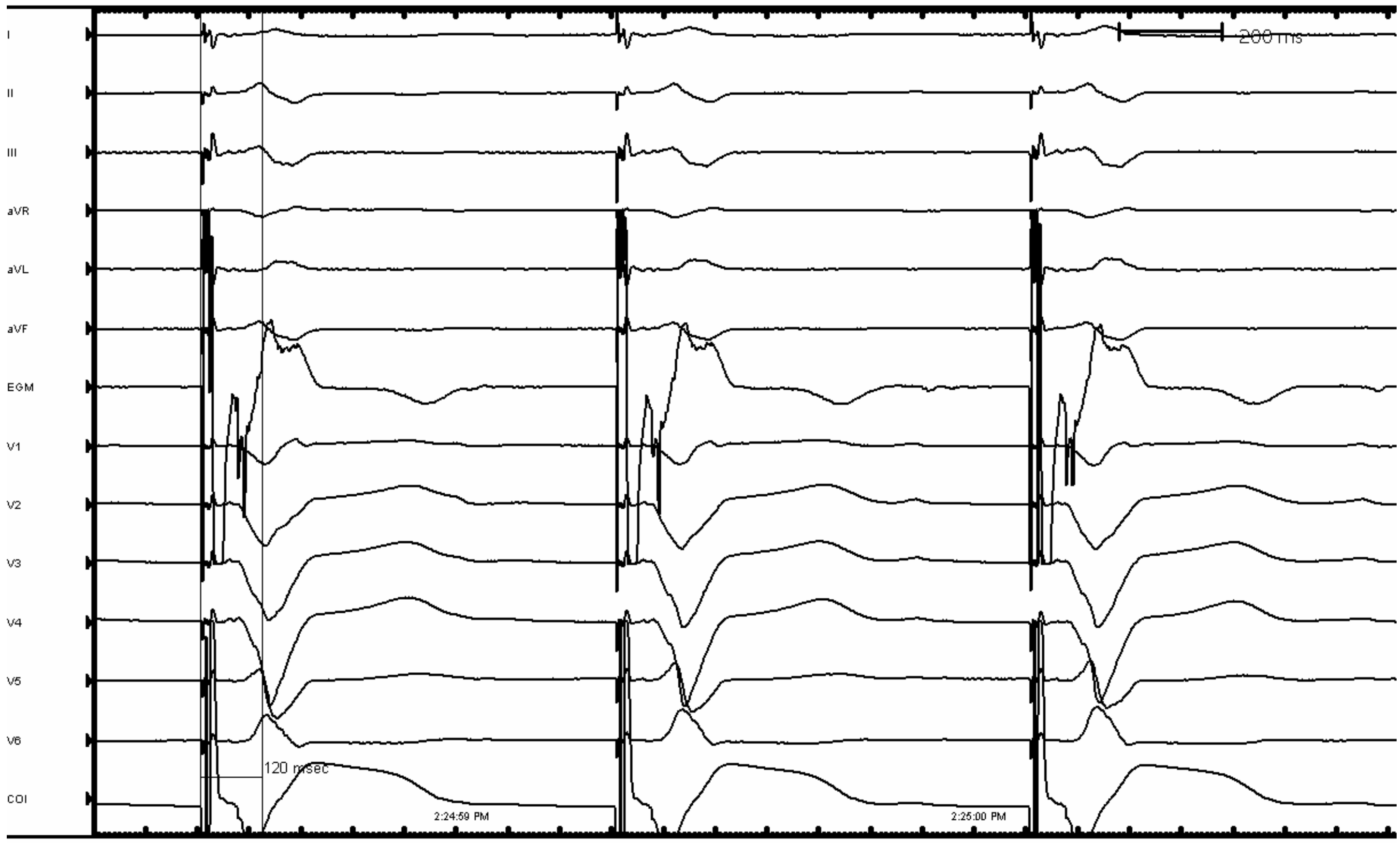

Transitions

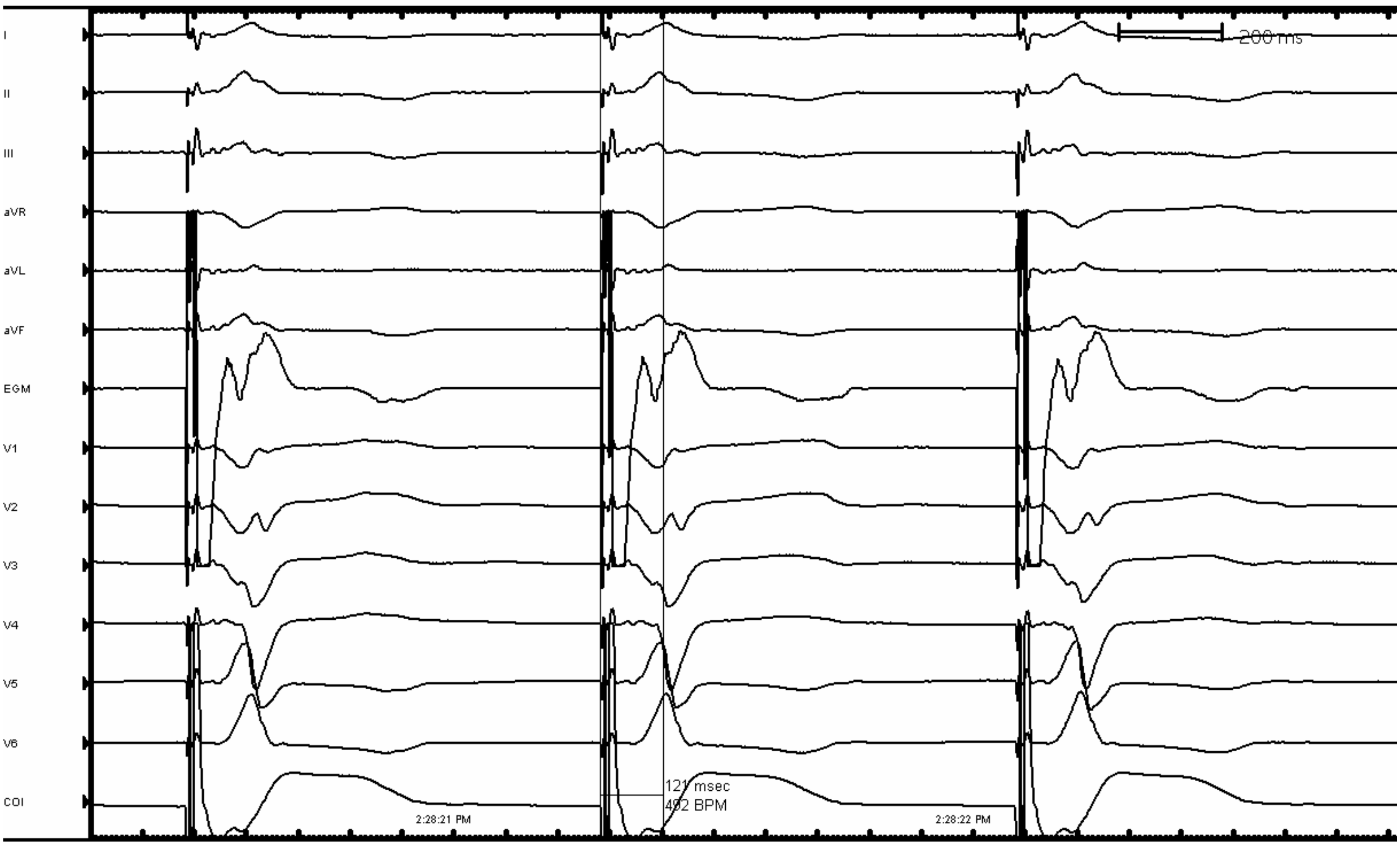

Patient 94:
Pre-ECG

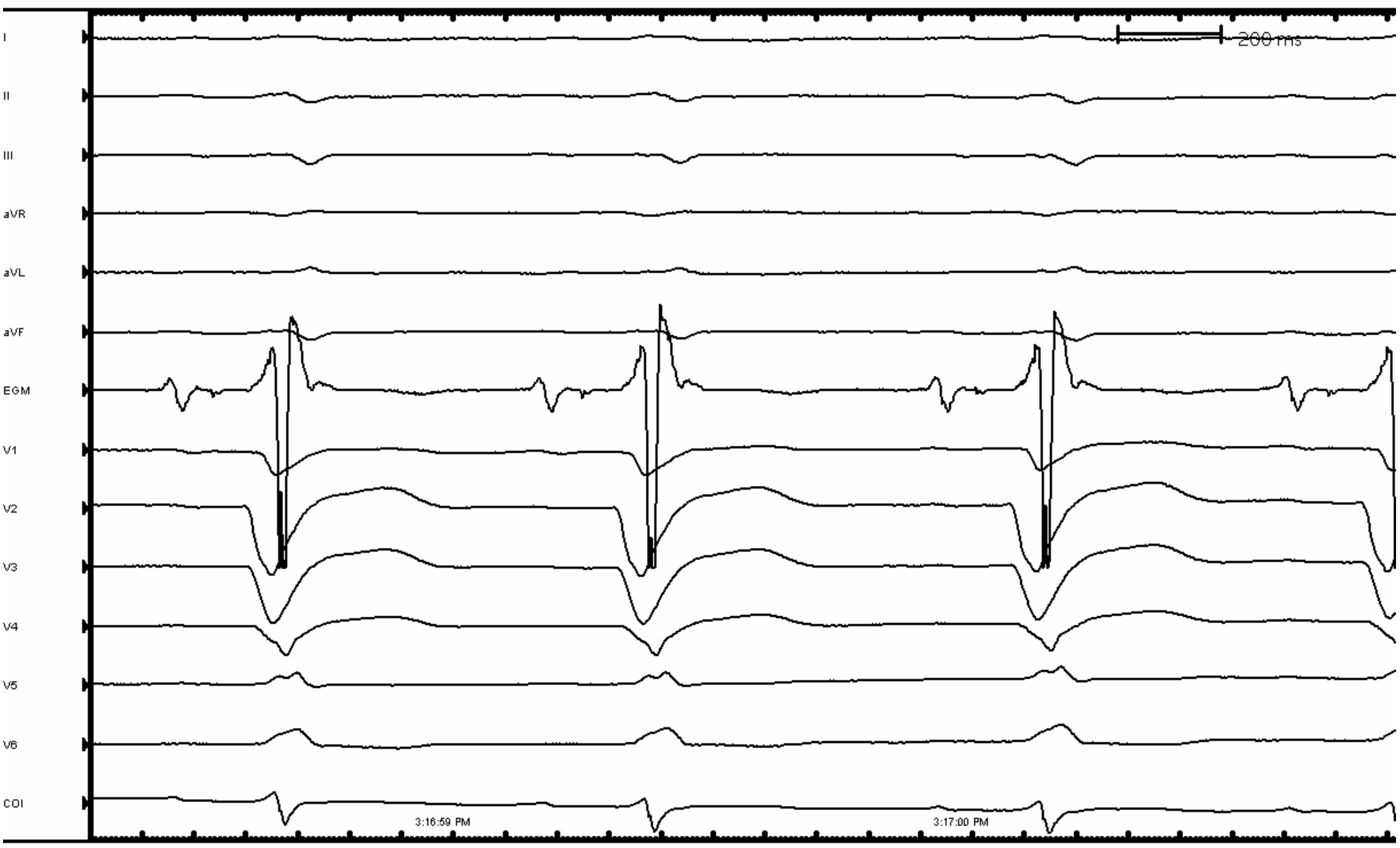

Post ECG

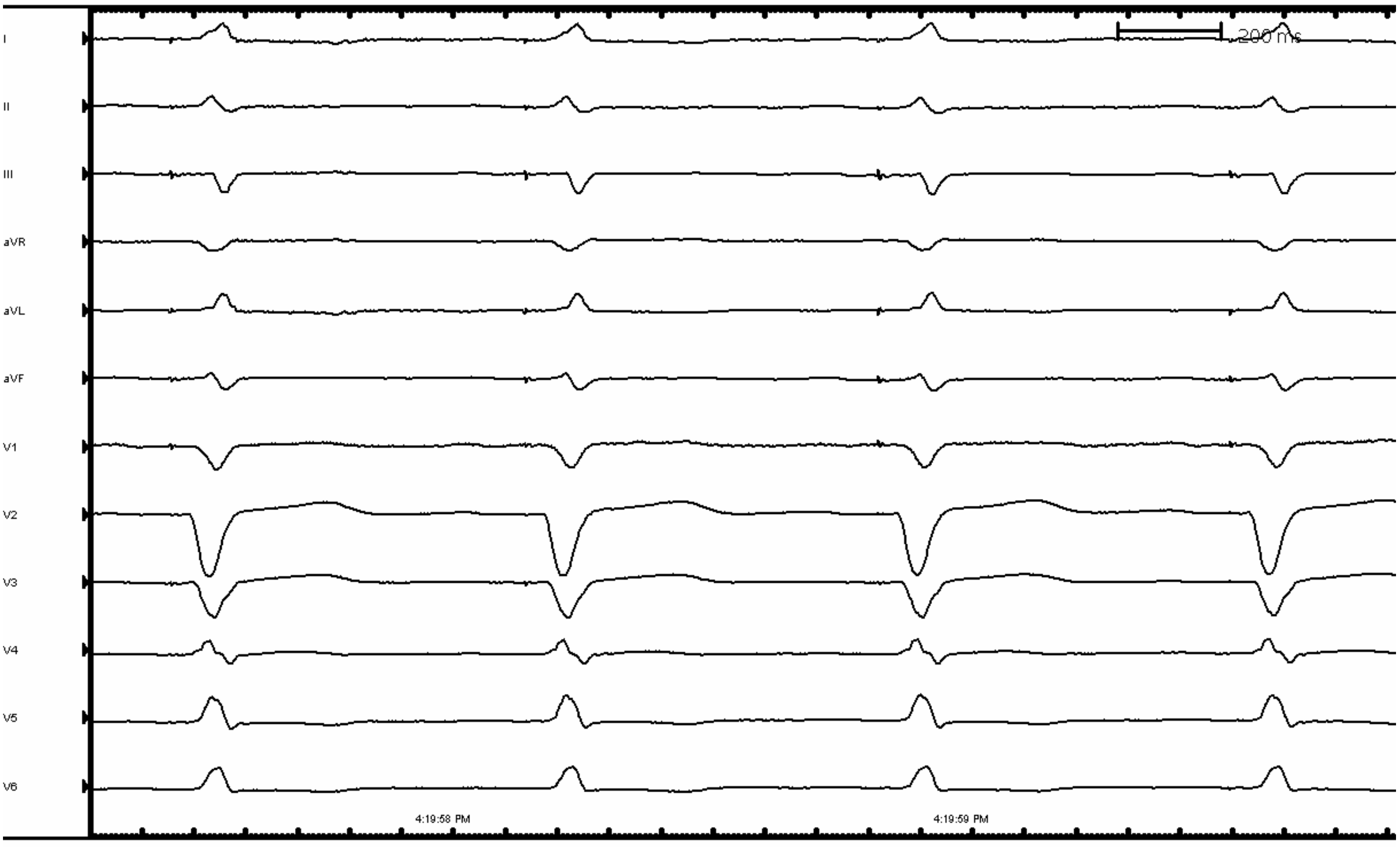

Patient 94:
Transitions

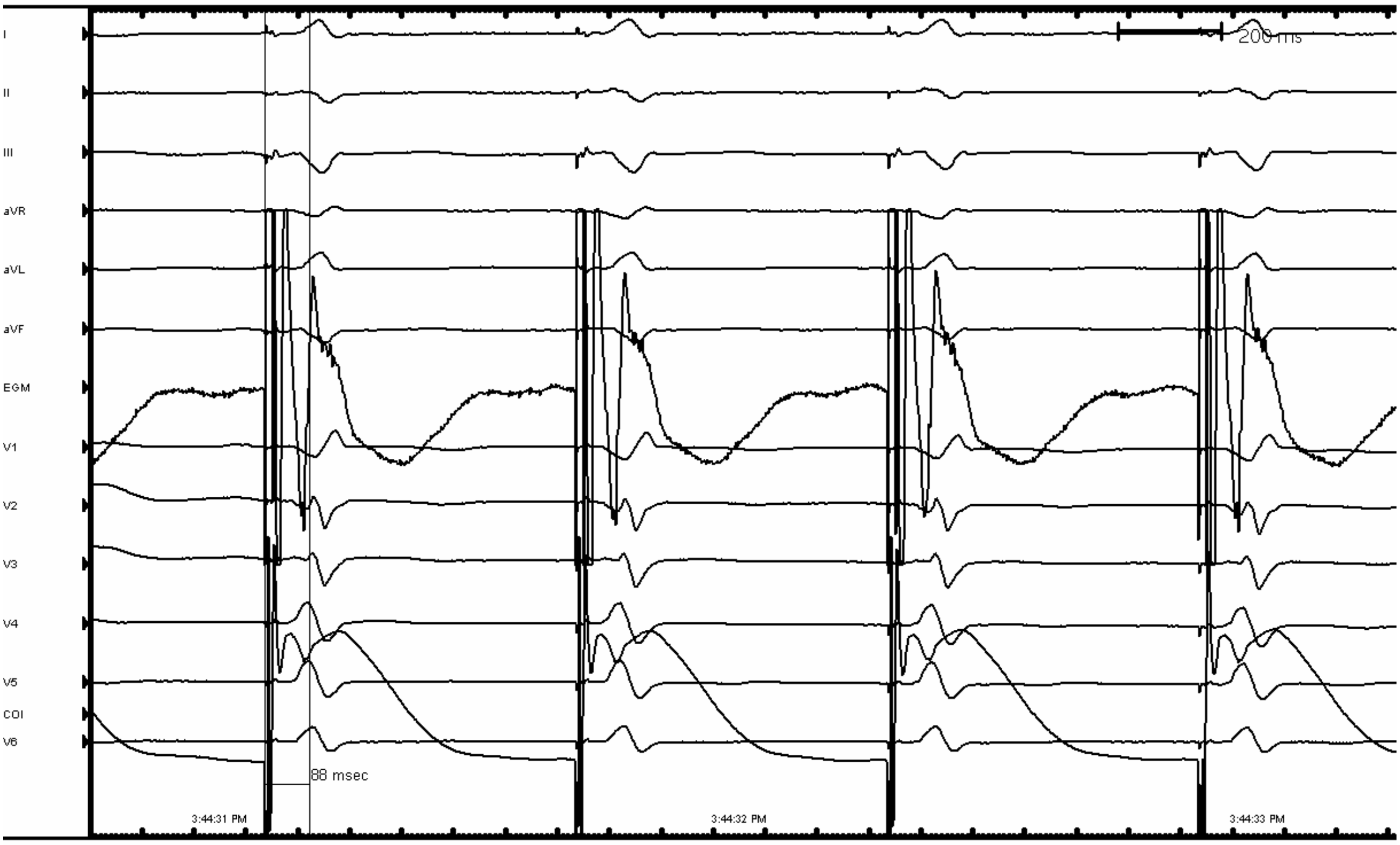

Transitions

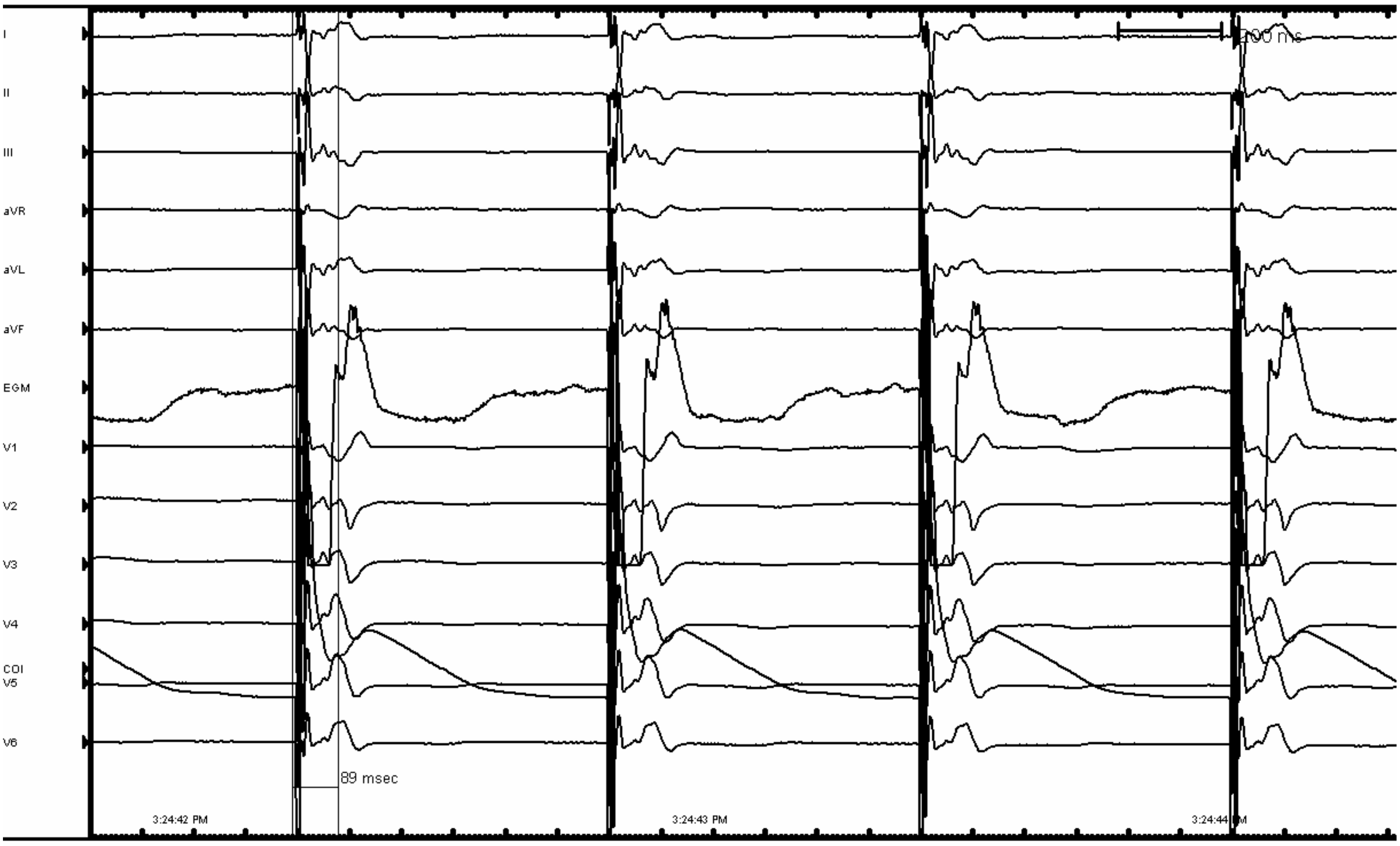

Patient 95:
Pre-ECG

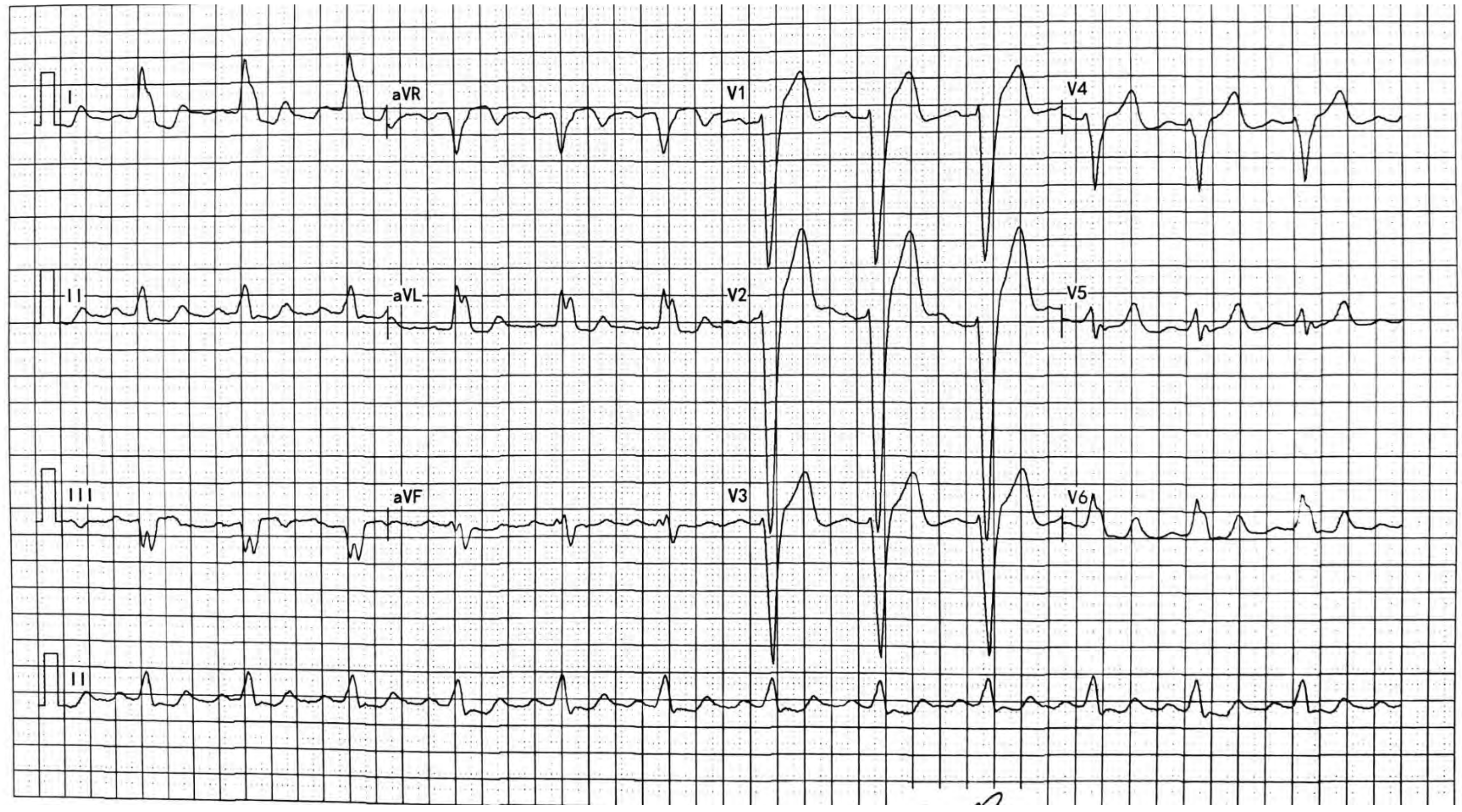

Post ECG

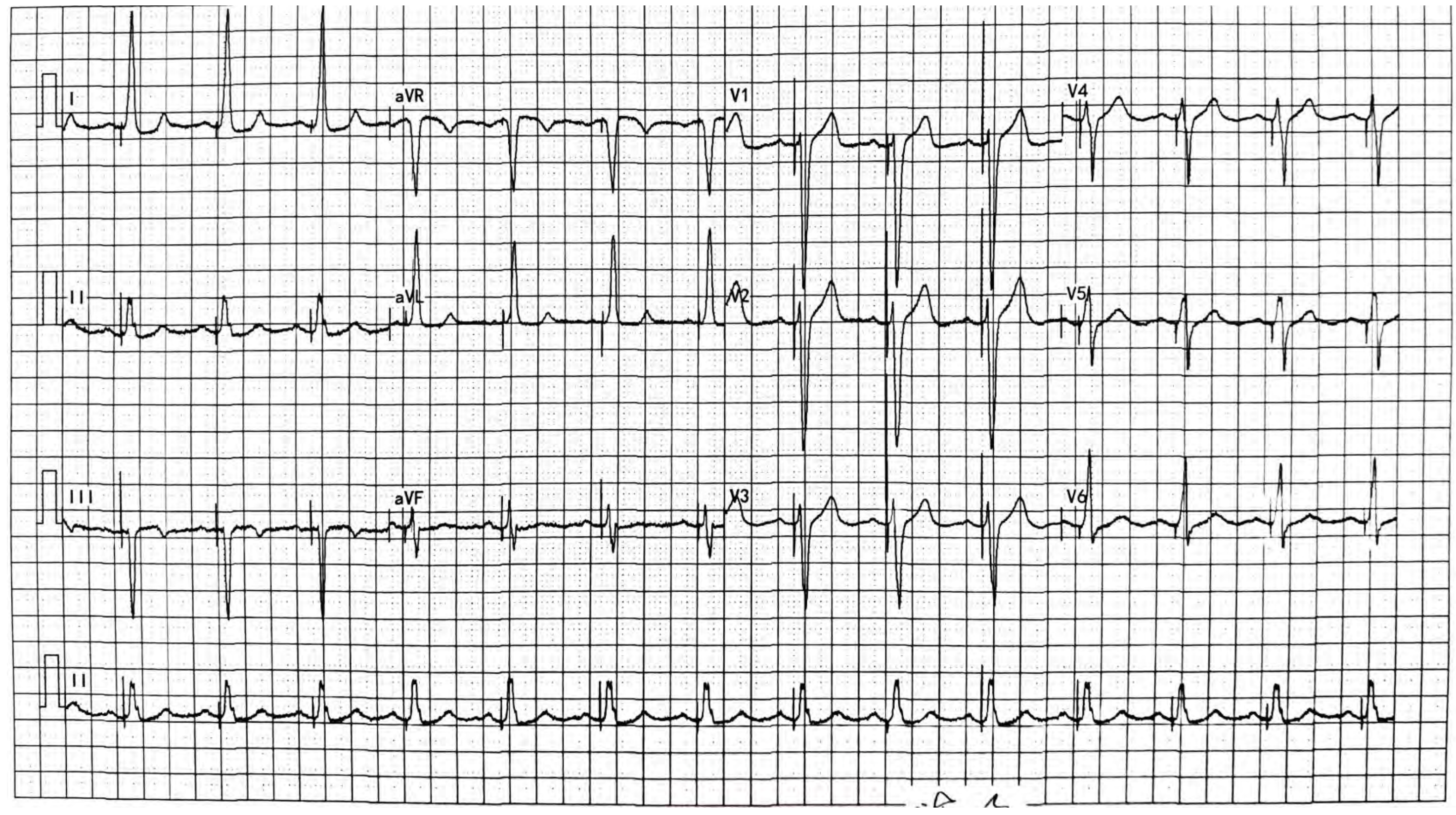

Patient 96:
Pre-ECG

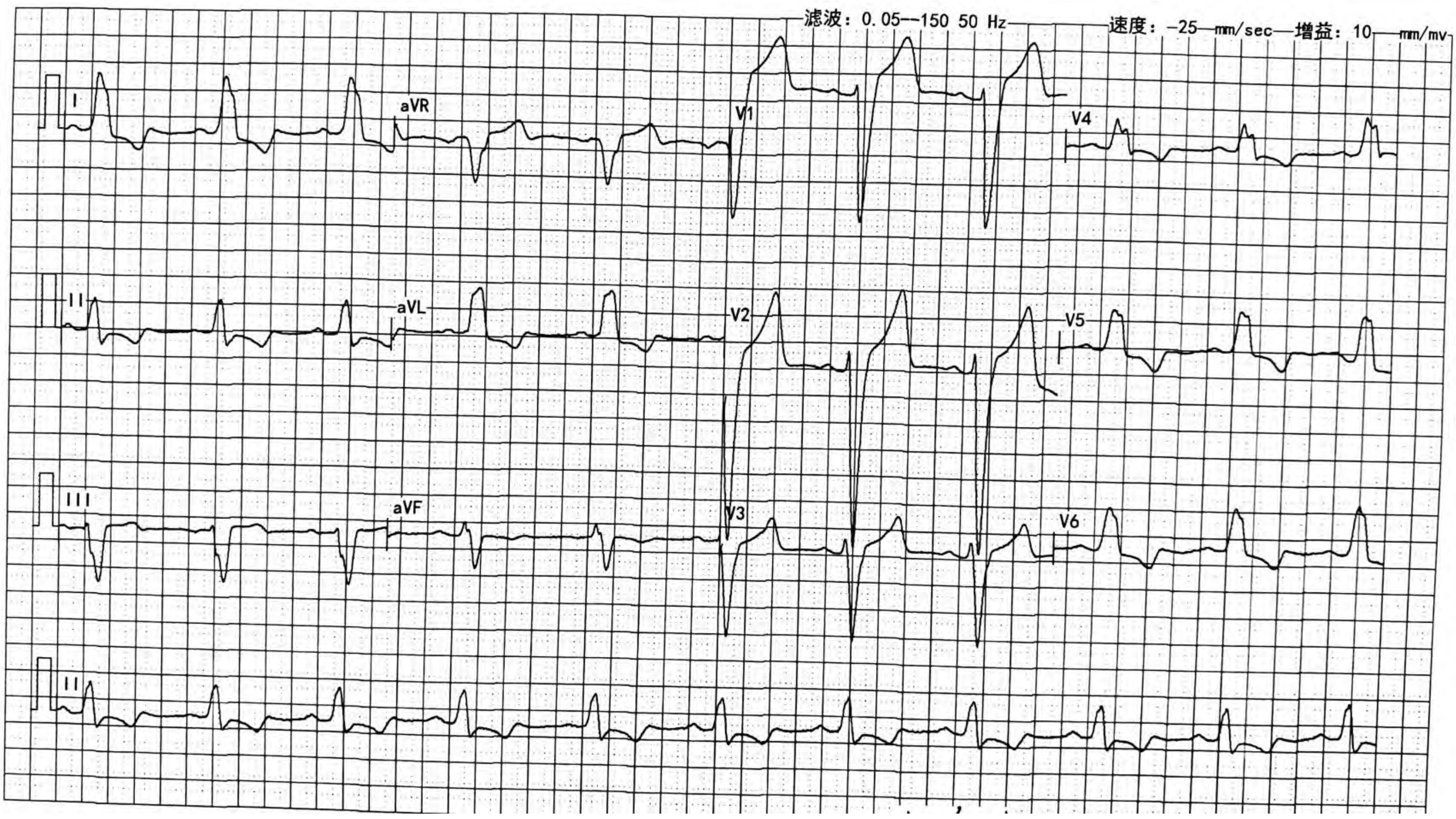

Post ECG

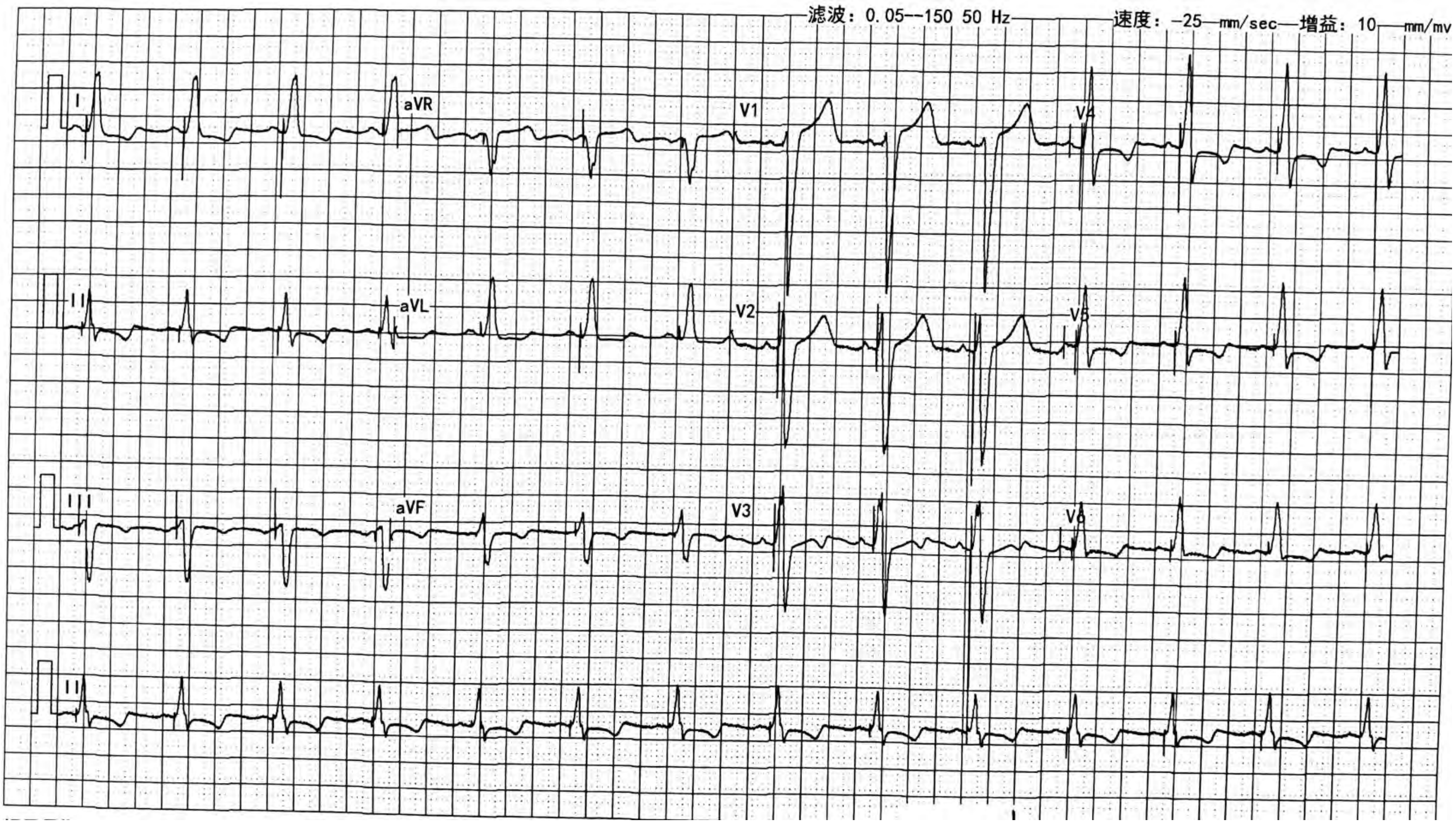

Patient 97:
Pre-ECG

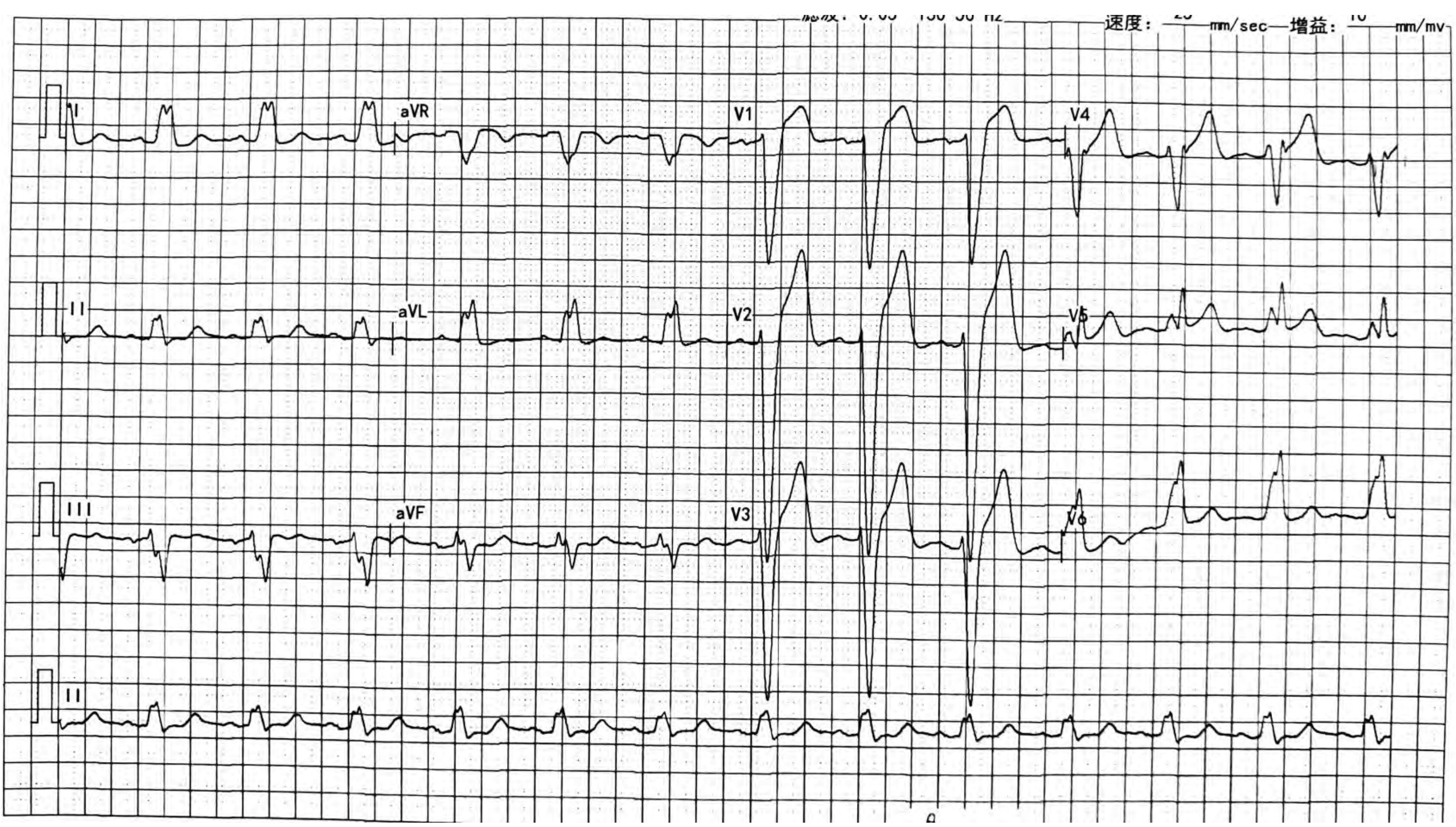

Post ECG

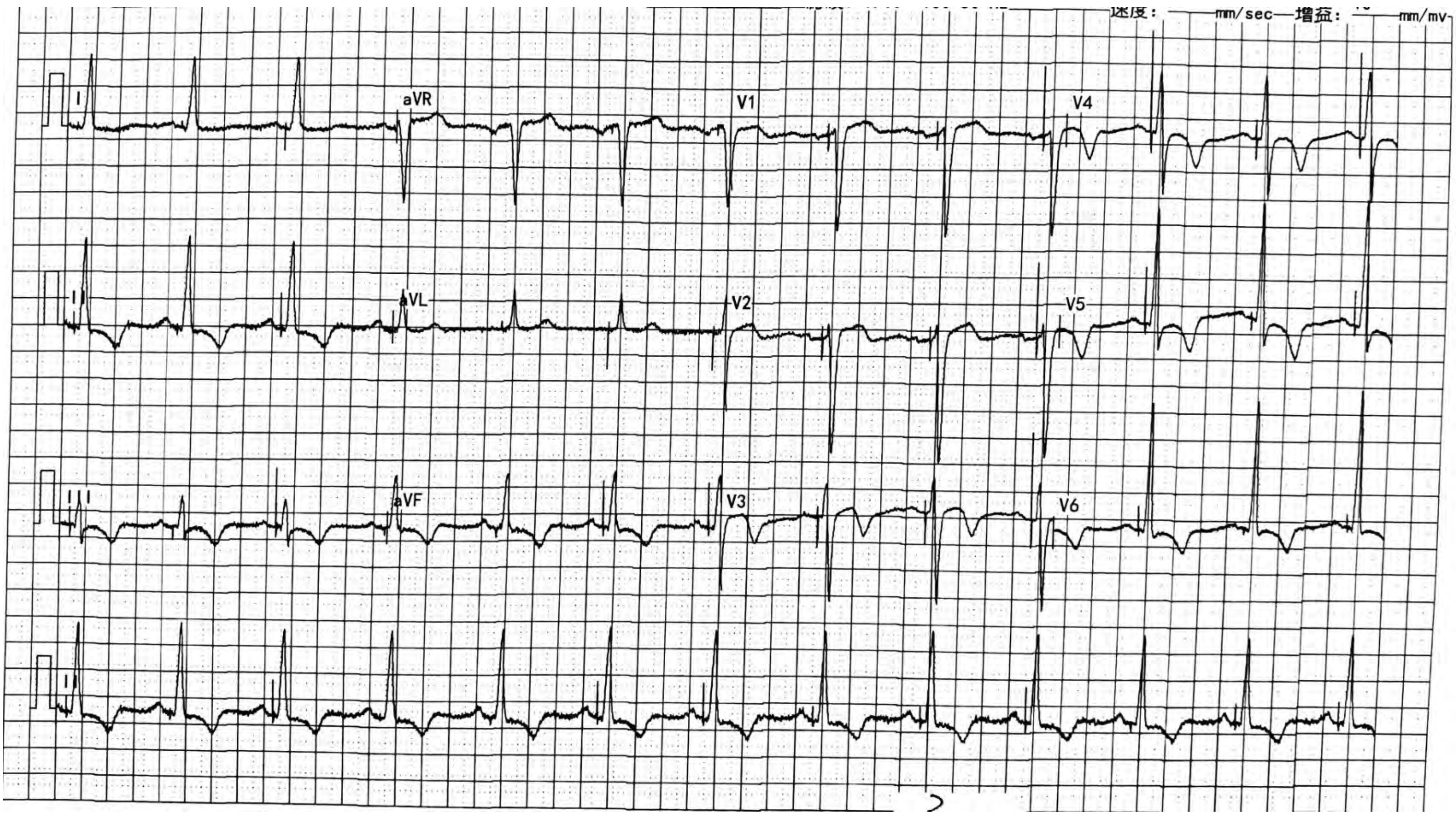

Patient 98:
Pre-ECG

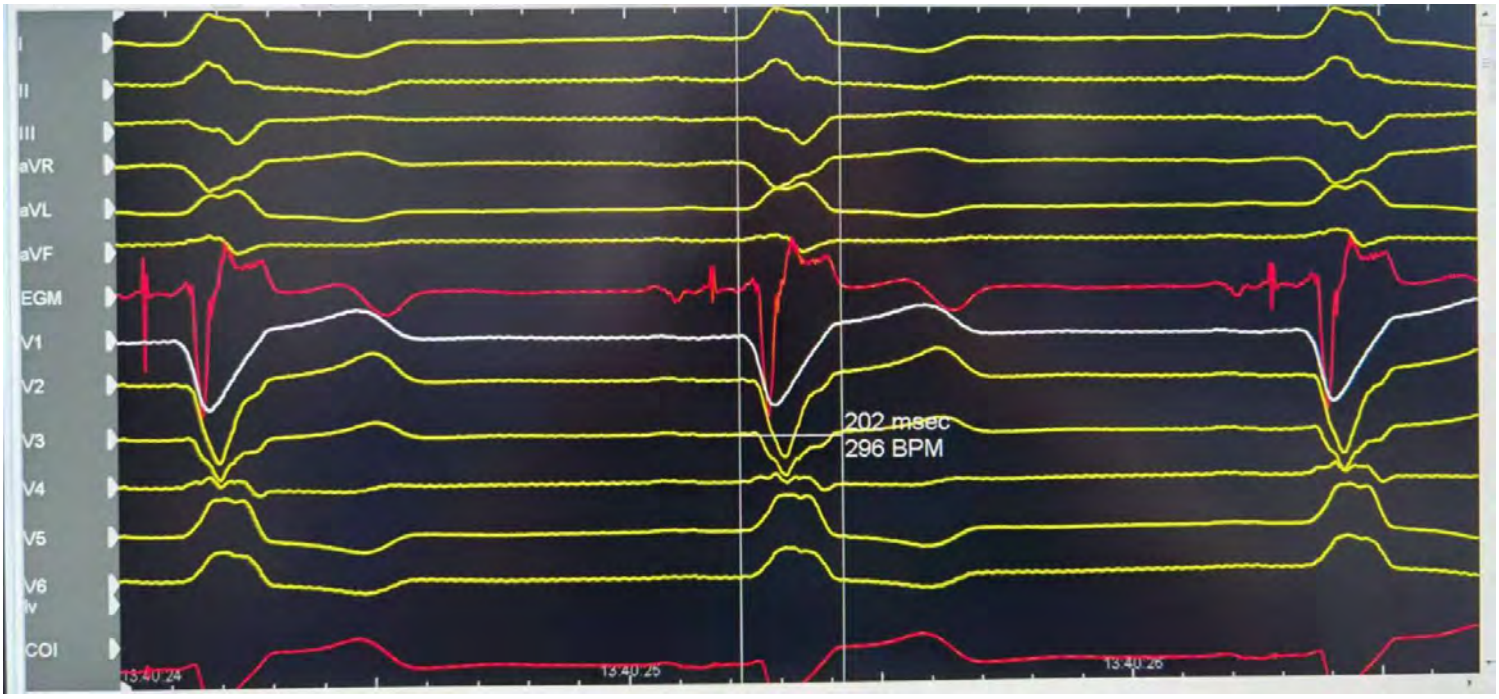

Post ECG

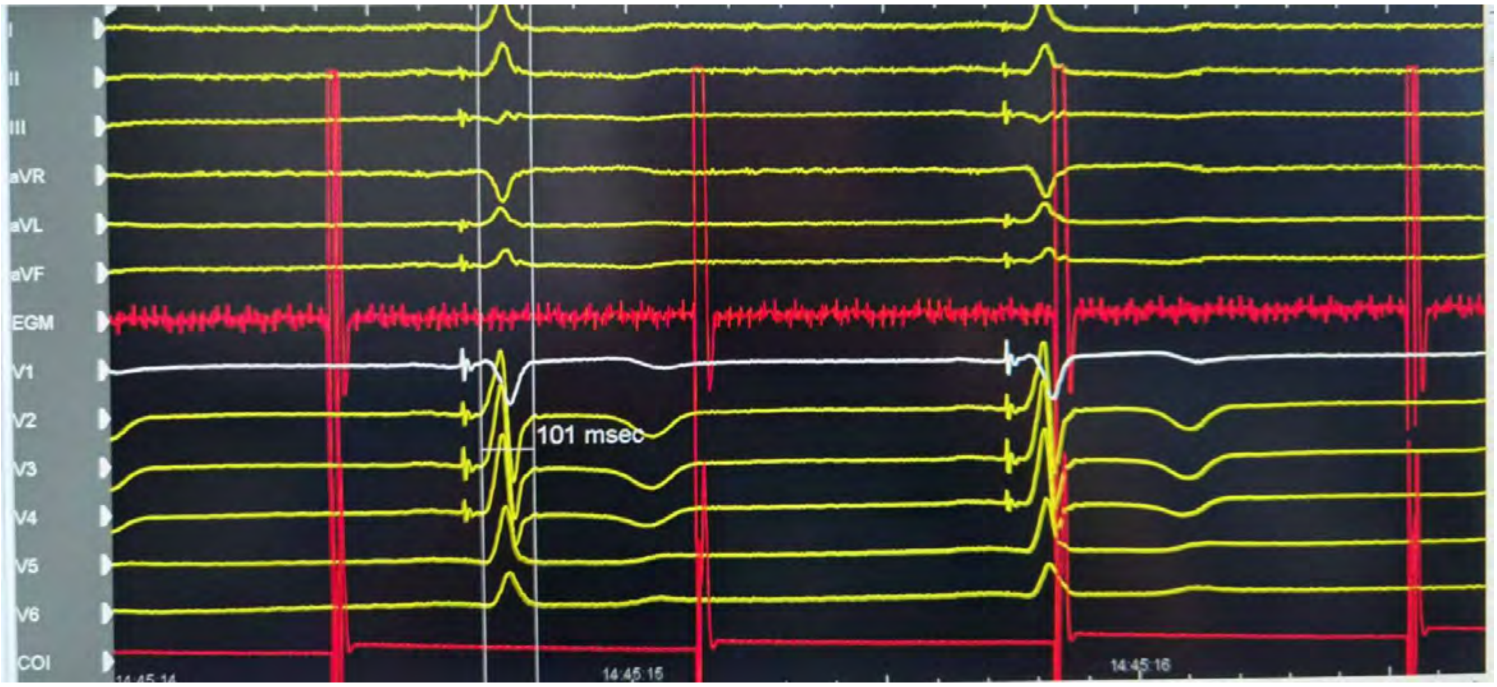

Patient 98:
Transitions

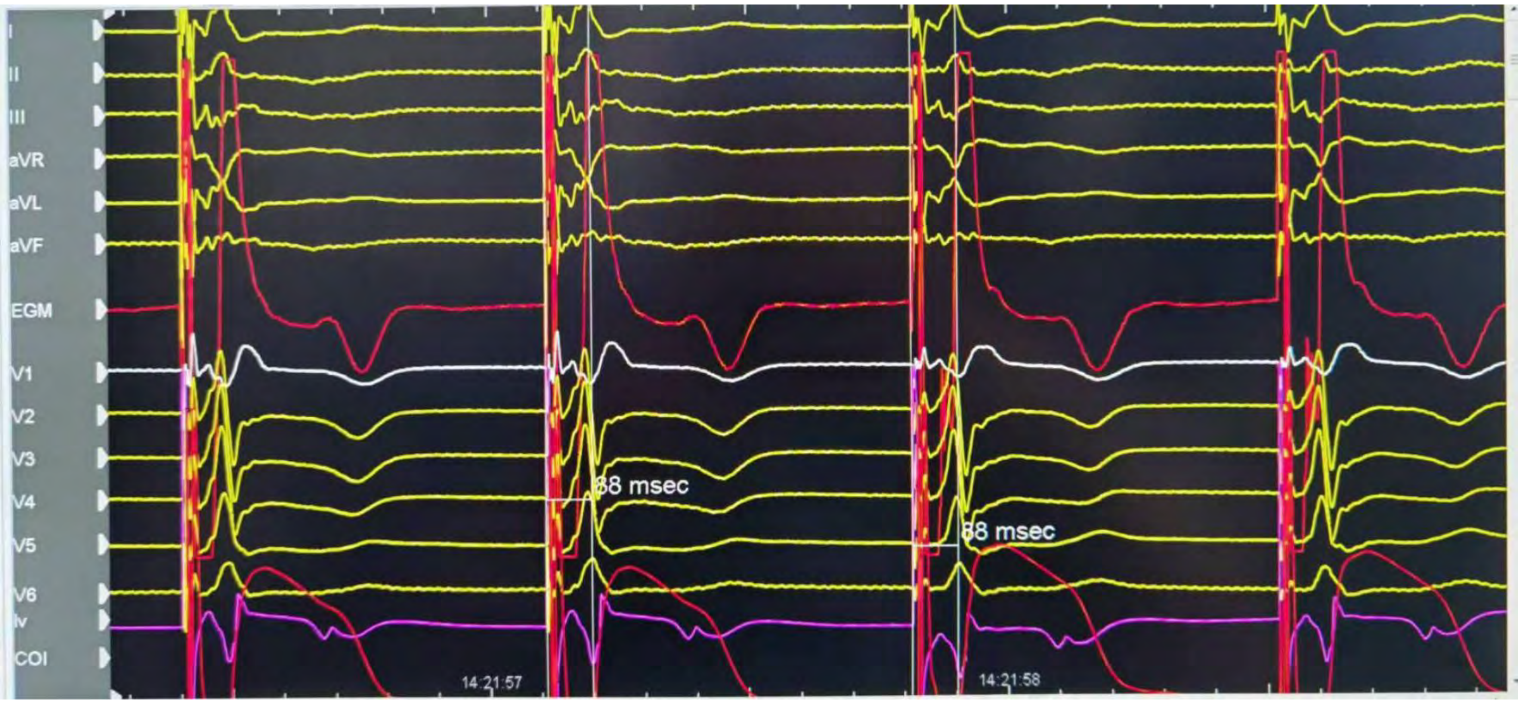

Transitions
