# Supplementary material for: Long-Term Outcomes of Left Bundle-Branch Pacing vs Biventricular Pacing in Heart Failure: The HeartSync-LBBP Randomized Clinical Trial
Source: JAMA Cardiol. 2026 Mar 11;11(4):352–9. doi: 10.1001/jamacardio.2026.0083 (PMC12980356; doi:10.1001/jamacardio.2026.0083)
Supplement: Supplement 2. — Trial Protocol and Statistical Analysis Plan [file jamacardiol-e260083-s002.pdf]

Protocol

Protocol for: Chen X, Liu X, Li R, et al. Long-term Outcomes of Left Bundle Branch Pacing versus Biventricular Pacing in Heart Failure: The HeartSync-LBBP Randomized Clinical Trial.

This trial protocol has been provided by the authors to give readers additional information about the work.

This supplement contains the following items:

1. Original protocol (V1.0, Page 3-19), summary of changes of protocol (Page 20) and final protocol (V1.1, Page 21-38).

2. Statistical analysis plan (V1.0, Page 39-43).

Study protocol

Title：Comparison of left bundle branch pacing and biventricular pacing in the treatment of chronic systolic heart failure with left bundle branch block: a multicenter, prospective, randomized, controlled trial

Primary investigator:

Yangang Su, MD

Department of Cardiology, Zhongshan Hospital of Fudan University, Shanghai Institute of Cardiovascular Diseases, National Clinical Research Center for Interventional Medicine, Shanghai, China

Table of Contents

1. **Summary** 2
2. **Background** 4
3. **Aim** 5
4. **Study design** 6
5. **Study participants** 6
6. **Interventional method** 7
7. **Data collection and follow-up** 8
8. **Safety assessment** 9
9. **Study outcomes** 9
10. **Data management** 10
11. **Statistical consideration** 10
12. **Enrolling center** 11
13. **Bias control** 12
14. **Ethics approval** 12
15. **Confidentiality measures** 13
16. **Proposed study timelines** 13
17. **Reference** 14

1. Summary

Title	Comparison of left bundle branch pacing and (LBBP) biventricular pacing (BVP) in the treatment of chronic systolic heart failure (HF) with left bundle branch block (LBBB): a multicenter, prospective, randomized, controlled trial
Aim	To evaluate the effectiveness and safety of LBBP versus BVP.
Study design	Multicenter, prospective, randomized, controlled trial.
Sample size	200
Patient selection	Inclusion criteria:<br><br>(1) Age 18 to 80 years; (2) sinus rhythm, complete LBBB; (3) Left ventricular ejection fraction (LVEF) ≤ 35% with New York Heart Association (NYHA) functional class II to IV despite receiving at least 3 months' guideline directed medical therapy; (4) expected survival time > 1 year; (5) The residence is stable and can meet the requirements of follow-up during the study period.<br><br>Exclusion criteria:<br><br>(1) Decline to participate or refuse to sign the written informed consent; (2) participate in other clinical studies; (3) persistent or permanent atrial fibrillation; (4) heart transplant patients or patients waiting for heart transplantation; (5) patients with previous pacemaker implantation; (6) patients with mechanical tricuspid valve implantation; (7) severe renal insufficiency; (8) severe liver dysfunction; (9) pregnancy.
Intervention	Randomly assigned to receive either LBBP or BVP in a 1:1 ratio.
Clinical outcomes	(1) Primary endpoint: the composite outcome of all-cause mortality

	and HF hospitalization (HFH).<br><br>(2) Secondary endpoints: individual outcomes of all-cause mortality, HFH, echocardiographic response, and super-response rates.
Safety assessment	Adverse events.
Follow-up plan	Follow-up visits will be conducted at 1 month, 3 months, 6 months, 12 months, 18 months, 24 months, 36 months after surgery.
Study execute time	From October 2020 to September 2024.

2. Background

Chronic heart failure (CHF) is a significant global disease burden. In HF patients combined with left bundle branch block (LBBB), the classic modality of cardiac resynchronization therapy (CRT) — biventricular pacing (BVP), can correct the asynchrony of myocardial contraction that occurs during the progression of heart failure (HF) by synchronizing the pacing of the left and right ventricles. This can improve ventricular filling pressure and filling time during the cardiac contraction phase, reduce mitral regurgitation, and increase ventricular ejection fraction and cardiac output, making it an effective treatment for HF. Large-scale randomized controlled studies have confirmed the effectiveness of BVP in improving CHF. However, approximately 30% of patients implanted with BVP do not show response to the treatment, and over 40% do not show reversal of left ventricular remodeling. Currently, left ventricular (LV) leads are implanted through the coronary sinus into the venous branches of the epicardium, and the final implantation site is influenced by the anatomical structure of the venous system, pacing parameters, stability of the lead, and phrenic nerve stimulation. Additionally, the LV lead implanted in the epicardial venous branches does not function as a physiological pacing modality: its electrical activation spreads from the epicardium to the endocardium, which is completely opposite to normal electrical conduction and may induce ventricular arrhythmias. Thus, improving the therapeutical effect and response rate of BVP has been a current research focus and challenge.

His bundle pacing (HBP) involves directly implanting leads in the His bundle area, allowing electrical impulses to be transmitted through the heart's normal conduction system, thereby maintaining the sequence of ventricular electrical activation and the synchrony of ventricular contraction. This method can achieve better anti-arrhythmic effects and hemodynamic outcomes, making it one of the current research hotspots in the field of pacing therapy. Recent small-scale studies have found that for patients

with HF combined with LBBB, HBP can correct LBBB and improve electrical synchrony, thereby alleviating HF. However, the target area for HBP is relatively small and is wrapped in an insulated fibrous sheath, making it more difficult to operate and resulting in issues such as lower sensing and higher pacing thresholds. For HF patients with LBBB, previous studies showed that the proportion of correcting LBBB using HBP is around 70%. However, the correction threshold is generally quite high (with an average threshold mostly > 2V). Although it can improve cardiac function, it may also lead to premature depletion of the pulse generator, forcing patients to face early replacement of the pulse generator, which creates an economic burden and potential risks of surgical complications. Additionally, the long-term stability of pacing parameters and the possibility of progression of conduction system disease in patients are limitations that restrict its clinical application.

Left bundle branch pacing (LBBP), which corrects LBBB by directly implanting the lead in the left bundle branch (LBB) area, can improve left ventricular synchrony and cardiac contractile function. Small-scale observational studies have reported a high success rate for implantation and good short- to mid-term safety. For patients with HF combined with LBBB, LBBP can significantly improve cardiac function, with lower and more stable thresholds for correcting LBBB. However, there are currently no large-scale, multicenter, randomized, controlled studies comparing the efficacy differences between LBBP and conventional BVP in HF patients with LBBB.

3. Aim

This a multicenter, prospective, randomized, controlled trial which enrolling patients with HF, left ventricular ejection fraction (LVEF) ≤ 35%, and LBBB. Patients will be randomly assigned in a 1:1 ratio to receive either LBBP or BVP. The clinical endpoints (including primary and secondary endpoints) will be compared between the

two groups to evaluate the effectiveness and safety, in order to provide new treatment methods for such patients and improve their prognosis.

4. Study design

4.1 Overall design

This a multicenter, prospective, randomized, controlled trial. HF patients with reduced LVEF and LBBB will be screened, and a total of 200 patients will be finally enrolled. Enrolled patients will be then randomly assigned to receive either LBBP or BVP in a 1:1 ratio. Follow-up visits will be conducted at 1 month, 3 months, 6 months, 12 months, 18 months, 24 months, 36 months after surgery to evaluate the clinical endpoints of the two groups.

4.2 Masking

The research assistants who collect study outcome data will be masked to the participants' intervention assignment. All the efficacy and safety end points will be independently adjudicated by a committee whose members are unaware of the trial-group assignments and the identity of the patients.

5. Study participants

5.1 Inclusion criteria

(1) Age 18 to 80 years; (2) sinus rhythm, complete LBBB; (3) LVEF ≤ 35% with New York Heart Association (NYHA) functional class II to IV despite receiving at least 3 months' guideline directed medical therapy; (4) expected survival time > 1 year; (5)

the residence is stable and can meet the requirements of follow-up during the study period.

5.2 Exclusion criteria

(1) Decline to participate or refuse to sign the written informed consent; (2) participate in other clinical studies; (3) persistent or permanent atrial fibrillation; (4) heart transplant patients or patients waiting for heart transplantation; (5) patients with previous pacemaker implantation; (6) patients with mechanical tricuspid valve implantation; (7) severe renal insufficiency; (8) severe liver dysfunction; (9) pregnancy.

5.3 Termination criteria

(1) Subjects ask to withdraw the informed consent form; (2) The investigator determines that the subject is not suitable for further study.

5.4 Enrolling center

All patients will be enrolled from 6 centers in China: (1) Zhongshan Hospital of Fudan University; (2) Shanghai Chest Hospital, Shanghai Jiao Tong University; (3) Shanghai Changhai Hospital, Naval Medical University; (4) Shanghai Changzheng Hospital, Second Affiliated Hospital of Naval Medical University; (5) Renji Hospital, Shanghai Jiao Tong University School of Medicine; (6) Shanghai Jiao Tong University Affiliated Sixth People's Hospital.

6. Interventional method

6.1 Procedural of LBBP

LBBP will be performed as following steps: (1) the His bundle (HB) potential will be first mapped with the pacing lead through the sheath; (2) then the lead is moved from the HB potential location towards the right ventricular apex for approximate 1 to 2 cm under the right anterior oblique (RAO) 30° fluoroscopic view and is deeply screwed into the interventricular septum (IVS); (3) LBBP is considered to be successful if the unipolar paced QRS morphology demonstrate a right bundle branch block (RBBB) pattern and meet any of the following criteria: 1) Paced morphology changes between selective LBBP and nonselective LBBP with constant left ventricular activation time (LVAT); 2) Paced morphology changes from left ventricular septal pacing (LVSP) to nonselective LBBP with LVAT abruptly shortening by increasing pacing output. Crossover will be allowed when LBB capture could not be achieved.

6.2 Procedural of BVP

BVP will be performed with a standard fashion and lateral or posterolateral branches are preferred choices for LV lead implantation, quadripolar LV lead is also used whenever feasible. Crossover will be allowed when the LV lead implant unsuccessfully due to anatomical variations, high pacing threshold, or phrenic nerve stimulus.

6.3 Device programming

In LBBP group, atrioventricular delay will be customized according to electrocardiogram to fuse with the intrinsic right bundle branch (RBB) conduction to achieve narrowest QRS duration (QRSd). In BiVP group, the optimization of AV and VV delay in each patient will be performed based on the paced QRSd.

7. Data collection and follow-up

Baseline data including age, gender, etiology (nonischemic cardiomyopathy or not), comorbidities (hypertension, diabetes mellitus and etc.), NYHA functional class and current medications (angiotensin-converting enzyme inhibitors/ angiotensin II receptor blocker/ angiotensin receptor-neprilysin inhibitor, beta-blockers, spironolactone) will be collected at enrollment.

Type of device (cardiac resynchronization therapy with defibrillator or with pacemaker) and electrocardiographic measurements (intrinsic QRSd and paced QRSd) will be collected at implantation, and the intrinsic QRSd and paced QRSd are measured at speed of 100 mm/s from the onset to the end of QRS complex.

All enrolled patients will be routinely followed up at 1 month, 3 months, 6 months, 12 months, 18 months, 24 months, 36 months after surgery in the device clinic. Pacing threshold is collected at implantation and follow-up. Echocardiographic measurements including LVEF, left ventricular end-diastolic diameter (LVEDD) and left ventricular end-systolic diameter (LVESD) will be collected at implantation and follow-up.

8. Safety assessment

Procedure related complications including pneumothorax, pocket infection, lead dislodgement requiring revision, increased pacing threshold > 3 V/0.5ms and pericardial tamponade will be documented as safety assessment during follow-up.

9. Study outcomes

9.1 Primary endpoint

The composite of all-cause mortality and HF hospitalization (HFH). HFH is defined as any urgent visit with HF signs or symptoms requiring intravenous diuretic therapy.

9.2 Secondary endpoints

Secondary endpoints including: (1) Individual outcomes of all-cause mortality; (2) individual outcomes of HFH; (3) Echocardiographic response/super-response rates at 6-month follow-up. Echocardiographic response is defined as an absolute improvement in LVEF ≥ 5%. Super-response is defined as an absolute improvement in LVEF ≥ 15% or improvement of LVEF to ≥ 50%.

10. Data management

10.1 Case report form

All data including screening assessments, examinations, therapeutic effects and safety assessments, will be documented in the participant's case report form (CRF). All CRFs for each patient should be collected and documented by study group member in a timely manner. Each CRF should be double-checked for missing data or potential errors, and will be stored in the study office.

10.2 Data entry

All data will be double-entered by two study group members, and will be collected at two databases for consistency. Whenever inconsistencies are found, the data will be rechecked by re-examination according to original CRFs or medical history.

11. Statistical consideration

11.1 Sample size calculating

There are no studies evaluating the long-term prognosis of LBBP, and results for the long-term prognosis of BVP vary among studies. Therefore, the data for the sample size estimation of this study are mainly derived from our preliminary clinical experience. A sample size of 190 patients is calculated to detect a statistically significant difference in the primary endpoint with 80% power and two-tailed alpha level of 5%, assuming 10% event rate in the LBBP and 25% in the BVP arm with 10% dropout and 5% crossover. To ensure sufficient statistical efficiency, the sample size is finally determined to be 100 for each group.

11.2 Statistical analysis

All analyses in this study will be conducted according to the intention-to-treat principle. Continuous variables are described as mean ± SD and categorical variables are described as frequencies or percentages. Student's t-test is performed for normally distributed continuous variables while Wilcoxon signed rank test is for non-normally distributed data. The chi-square test or Fisher exact test is used for categorical variables. For the clinical endpoint, Kaplan-Meier (KM) survival curve and log-rank test are performed to compare the time-to-event between LBBP and BVP. Subgroup analyses of the relationship between the primary endpoint and the baseline characteristics including age, sex, etiology, comorbidities, QRS duration, NYHA class, and left ventricular ejection fraction will be conducted. A two-sided P-value < 0.05 is considered statistically significant. All statistical analyses are performed using SPSS Statistics version 22.0 (IBM Corporation, Armonk, NY).

12. Enrolling center

Zhongshan Hospital of Fudan University	Primary center

Shanghai Changhai Hospital	Participating center
Shanghai Changzheng Hospital	Participating center
Shanghai Chest Hospital	Participating center
Shanghai Jiao Tong University Affiliated Sixth People's Hospital	Participating center
Renji Hospital	Participating center

13. Bias control

As this study is a prospective, randomized, controlled study, the randomization process should be strictly enforced to ensure the accuracy of the grouping. Follow-up members should avoid knowing the grouping results of each patient at the time of follow-up to prevent bias in the results.

14. Ethics approval

14.1 Review of the ethics committee

This study complies with the Declaration of Helsinki (revised in 2013) and the relevant regulations of Chinese clinical trials. The protocol and written informed consent must be submitted to the ethics committee, and written approval from the ethics committee must be obtained before the study can be formally conducted. The investigator must submit an annual report of the study to the ethics committee at least annually. The investigator must notify the ethics committee in writing when the study is discontinued and/or completed; the investigator must report to the ethics committee in a timely manner all changes occurring in the conduct of the study (e.g., revisions to the protocol and/or informed consent form) and must not implement these changes without first obtaining approval from the ethics committee, except for changes made

to eliminate obvious and immediate risks to subjects. The ethics committee will be informed when this occurs.

14.2 Informed consent

The investigator must provide the subject or his/her legal representative with a readily understandable and ethics committee - approved informed consent form and allow sufficient time for the subject or his/her legal representative to consider the study, and the subject will not be enrolled in the study until signed written informed consent has been obtained from the subject. Subjects will be provided with all updated versions of the informed consent form, along with written information, for the duration of the subject's participation. The informed consent form should be retained for review as an important document of the clinical trial.

15. Confidentiality measures

The results of the study may be published in medical journals, but we will maintain the confidentiality of the patients' information in accordance with the requirements of the law, and the personal information of the patients will not be disclosed unless it is required by the relevant laws. When necessary, the government administration and the hospital ethics committee and its related personnel can access the patients' information according to the regulations.

16. Proposed study timelines

This study is estimated to take about 4 years including patients' recruitment for 1.5 years. This study is expected to conducted between October 2020 and September 2024.

17. Reference

[1] Tops LF, Schalij MJ, Holman ER, van Erven L, van der Wall EE, Bax JJ. Right ventricular pacing can induce ventricular dyssynchrony in patients with atrial fibrillation after atrioventricular node ablation. J Am Coll Cardiol. 2006;48:1642–1648. doi: 10.1016/j.jacc.2006.05.072.
[2] Sweeney MO, Hellkamp AS, Ellenbogen KA, Greenspon AJ, Freedman RA, Lee KL, Lamas GA; MOde Selection Trial Investigators. Adverse effect of ventricular pacing on heart failure and atrial fibrillation among patients with normal baseline QRS duration in a clinical trial of pacemaker therapy for sinus node dysfunction. Circulation. 2003;107:2932–2937. doi: 10.1161/01.CIR.0000072769.17295.B1.
[3] Wilkoff BL, Cook JR, Epstein AE, Greene HL, Hallstrom AP, Hsia H, Kutalek SP, Sharma A. Dual-chamber pacing or ventricular backup pacing in patients with an implantable defibrillator: the Dual Chamber and VVI Implantable Defibrillator (DAVID) trial. JAMA. 2002;288:3115–3123.
[4] Shaan Khurshid BA, Andrew E. Epstein MD, FHRS, Ralph J. Verdino MD, David Lin MD, FHRS, Lee R. Goldberg MD, Francis E. Marchlinski MD, FHRS, David S. Frankel MD, FHRS, Incidence and Predictors of Right Ventricular Pacing-Induced Cardiomyopathy, Heart Rhythm, http://dx.doi.org/10.1016/j. hrthm.2014.05.040
[5] Kiehl EL, Makki T， Kumar R, Gumber D, Kwon DH, Rickard JW, Kanj M, Wazni OM, Saliba WI, Varma N, Wilkoff BL, Cantillon DJ. Incidence and predictors of right ventricular pacing-induced cardiomyopathy in patients with complete atrioventricular block and preserved left ventricular systolic function. Heart Rhythm. 2016 Dec;13(12):2272-2278. doi: 10.1016/j.hrthm.2016.09.027.
[6] Cho SW, Gwag HB, Hwang JK, Chun KJ, Park KM, On YK, Kim JS, Park SJ.Clinical features, predictors, and long-term prognosis of pacing-induced cardiomyopathy. Eur J Heart Fail. 2019;21(5):643-651.

[7] Incidence and predictors of right ventricular pacing-induced cardiomyopathy. Khurshid S, Epstein AE, Verdino RJ, Lin D, Goldberg LR, Marchlinski FE, Frankel DS.Heart Rhythm. 2014;11(9):1619-25.

[8] Curtis AB, Worley SJ, Adamson PB, Chung ES, Niazi I, Sherfesee L, Shinn T, Sutton MS;Biventricular versus Right Ventricular Pacing in Heart Failure Patients with Atrioventricular Block (BLOCK HF) Trial Investigators. Biventricular pacing for atrioventricular block and systolic dysfunction.

[9] Piotr Ponikowski, Adriaan A Voors, Stefan D Anker, Héctor Bueno, John G F Cleland, Andrew J S Coats, Volkmar Falk, José Ramón González-Juanatey, Veli-Pekka Harjola, Ewa A Jankowska, Mariell Jessup, Cecilia Linde, Petros Nihoyannopoulos, John T Parissis, Burkert Pieske, Jillian P Riley, Giuseppe M C Rosano, Luis M Ruilope, Frank Ruschitzka, Frans H Rutten, Peter van der Meer, ESC Scientific Document Group. 2016 ESC Guidelines for the Diagnosis and Treatment of Acute and Chronic Heart Failure: The Task Force for the Diagnosis and Treatment of Acute and Chronic Heart Failure of the European Society of Cardiology (ESC)Developed With the Special Contribution of the Heart Failure Association (HFA) of the ESC. Eur Heart J, 37 (27), 2129-2200.

[10] Funck RC, Mueller HH, Lunati M, Piorkowski C, De Roy L, Paul V, Wittenberg M, Wuensch D, Blanc JJ; BioPace study group. Characteristics of a large sample of candidates for permanent ventricular pacing included in the Biventricular Pacing for Atrio-ventricular Block to Prevent Cardiac Desynchronization Study (BioPace). Europace. 2014 Mar;16(3):354-62. doi: 10.1093/europace/eut343.

[11] Fred M Kusumoto, Mark H Schoenfeld, Coletta Barrett, James R Edgerton, Kenneth A Ellenbogen, Michael R Gold, Nora F Goldschlager, Robert M Hamilton, José A Joglar, Robert J Kim, Richard Lee, Joseph E Marine, Christopher J McLeod, Keith R Oken, Kristen K Patton, Cara N Pellegrini, Kimberly A Selzman, Annemarie Thompson, Paul D Varosy. 2018 ACC/AHA/HRS Guideline on the Evaluation and Management of Patients with Bradycardia and Cardiac Conduction Delay: A Report of the American College of Cardiology/American Heart Association Task Force on

Clinical Practice Guidelines and the Heart Rhythm Society. Circulation, 140 (8), e382-e482.

[12] Occhetta E, Bortnik M, Magnani A, et al. Prevention of ventricular desynchronization by permanent para-Hisian pacing after atrioventricular node ablation in chronic atrial fibrillation: a crossover, blinded, randomized study versus apical right ventricular pacing. J Am Coll Cardiol. 2006;47:1938-45.

[13] Zanon F, Bacchiega E, Rampin L, et al. Direct His bundle pacing preserves coronary perfusion compared with right ventricular apical pacing: a prospective, cross-over mid-term study. Europace. 2008;10:580-7

[14] Kronborg MB, Mortensen PT, Poulsen SH, et al. His or para-His pacing preserves left ventricular function in atrioventricular block: a double-blind, randomized, crossover study. Europace. 2014;16:1189-96

[15] Abdelrahman M, Subzposh FA, Beer D, Durr B, Naperkowski A, Sun H, Oren JW, Dandamudi G, Vijayaraman P, Clinical Outcomes of His Bundle Pacing Compared to Right Ventricular Pacing, Journal of the American College of Cardiology (2018), doi: 10.1016/ j.jacc.2018.02.048.

[16] Vijayaraman P, Bordachar P, Ellenbogen KA. The continued search for physiological pacing: where are we now? J Am Coll Cardiol. 2017;69(25):3099-3114.

[17] Huang W, Su L, Wu S, Xu L, Xiao F, Zhou X, Ellenbogen KA. A novel pacing strategy with low and stable output: pacing the left bundle branch immediately beyond the conduction block. Can J Cardiol. 2017;33(12):1731-1736.

[18] Vijayaraman P, Subzposh FA, Naperkowski A, Panikkath R, John K, Mascarenhas V, et al. Prospective evaluation of feasibility, electrophysiologic and echocardiographic characteristics of left bundle branch area pacing. Heart Rhythm. 2019.

[19] Hou X, Qian Z, Wang Y et al. Feasibility and cardiac synchrony of permanent left bundle branch pacing through the interventricular septum. Europace, 2019,doi:10.1093/europace/euz188.

Summary of changes of protocol

**Protocol 1.0**	**Protocol 1.1**
Follow-up visits were described as: “at 1 month, 3 months, 6 months, 12 months, 18 months, 24 months, 36 months after surgery”.	Follow-up visits were described as: “at 1 month, 3 months, 6 months and every 6 months thereafter after surgery”.
None.	Add “4.3 Study flowchart”

Study protocol

Title：Comparison of left bundle branch pacing and biventricular pacing in the treatment of chronic systolic heart failure with left bundle branch block: a multicenter, prospective, randomized, controlled trial

Primary investigator:

Yangang Su, MD

Department of Cardiology, Zhongshan Hospital of Fudan University, Shanghai Institute of Cardiovascular Diseases, National Clinical Research Center for Interventional Medicine, Shanghai, China

Version： 1.1
Date: 2021.03.15

Table of Contents

1. Summary 3
2. Background 5
3. Aim 6
4. Study design 7
5. Study participants 8
6. Interventional method 9
7. Data collection and follow-up 10
8. Safety assessment 11
9. Study outcomes 11
10. Data management 12
11. Statistical consideration 12
12. Enrolling center 13
13. Bias control 14
14. Ethics approval 14
15. Confidentiality measures 15
16. Proposed study timelines 15
17. Reference 15

1. Summary

Title	Comparison of left bundle branch pacing and (LBBP) biventricular pacing (BVP) in the treatment of chronic systolic heart failure (HF) with left bundle branch block (LBBB): a multicenter, prospective, randomized, controlled trial
Aim	To evaluate the effectiveness and safety of LBBP versus BVP.
Study design	Multicenter, prospective, randomized, controlled trial.
Sample size	200
Patient selection	Inclusion criteria:<br><br>(1) Age 18 to 80 years; (2) sinus rhythm, complete LBBB; (3) Left ventricular ejection fraction (LVEF) ≤ 35% with New York Heart Association (NYHA) functional class II to IV despite receiving at least 3 months' guideline directed medical therapy; (4) expected survival time > 1 year; (5) The residence is stable and can meet the requirements of follow-up during the study period.<br><br>Exclusion criteria:<br><br>(1) Decline to participate or refuse to sign the written informed consent; (2) participate in other clinical studies; (3) persistent or permanent atrial fibrillation; (4) heart transplant patients or patients waiting for heart transplantation; (5) patients with previous pacemaker implantation; (6) patients with mechanical tricuspid valve implantation; (7) severe renal insufficiency; (8) severe liver dysfunction; (9) pregnancy.
Intervention	Randomly assigned to receive either LBBP or BVP in a 1:1 ratio.
Clinical outcomes	(1) Primary endpoint: the composite outcome of all-cause mortality

	and HF hospitalization (HFH).<br><br>(2) Secondary endpoints: individual outcomes of all-cause mortality, HFH, echocardiographic response, and super-response rates.
Safety assessment	Adverse events.
Follow-up plan	Follow-up visits will be conducted at 1 month, 3 months, 6 months and every 6 months thereafter after surgery.
Study execute time	From October 2020 to September 2024.

2. Background

Chronic heart failure (CHF) is a significant global disease burden. In HF patients combined with left bundle branch block (LBBB), the classic modality of cardiac resynchronization therapy (CRT) — biventricular pacing (BVP), can correct the asynchrony of myocardial contraction that occurs during the progression of heart failure (HF) by synchronizing the pacing of the left and right ventricles. This can improve ventricular filling pressure and filling time during the cardiac contraction phase, reduce mitral regurgitation, and increase ventricular ejection fraction and cardiac output, making it an effective treatment for HF. Large-scale randomized controlled studies have confirmed the effectiveness of BVP in improving CHF. However, approximately 30% of patients implanted with BVP do not show response to the treatment, and over 40% do not show reversal of left ventricular remodeling. Currently, left ventricular (LV) leads are implanted through the coronary sinus into the venous branches of the epicardium, and the final implantation site is influenced by the anatomical structure of the venous system, pacing parameters, stability of the lead, and phrenic nerve stimulation. Additionally, the LV lead implanted in the epicardial venous branches does not function as a physiological pacing modality: its electrical activation spreads from the epicardium to the endocardium, which is completely opposite to normal electrical conduction and may induce ventricular arrhythmias. Thus, improving the therapeutical effect and response rate of BVP has been a current research focus and challenge.

His bundle pacing (HBP) involves directly implanting leads in the His bundle area, allowing electrical impulses to be transmitted through the heart's normal conduction system, thereby maintaining the sequence of ventricular electrical activation and the synchrony of ventricular contraction. This method can achieve better anti-arrhythmic effects and hemodynamic outcomes, making it one of the current research hotspots in the field of pacing therapy. Recent small-scale studies have found that for patients

with HF combined with LBBB, HBP can correct LBBB and improve electrical synchrony, thereby alleviating HF. However, the target area for HBP is relatively small and is wrapped in an insulated fibrous sheath, making it more difficult to operate and resulting in issues such as lower sensing and higher pacing thresholds. For HF patients with LBBB, previous studies showed that the proportion of correcting LBBB using HBP is around 70%. However, the correction threshold is generally quite high (with an average threshold mostly > 2V). Although it can improve cardiac function, it may also lead to premature depletion of the pulse generator, forcing patients to face early replacement of the pulse generator, which creates an economic burden and potential risks of surgical complications. Additionally, the long-term stability of pacing parameters and the possibility of progression of conduction system disease in patients are limitations that restrict its clinical application.

Left bundle branch pacing (LBBP), which corrects LBBB by directly implanting the lead in the left bundle branch (LBB) area, can improve left ventricular synchrony and cardiac contractile function. Small-scale observational studies have reported a high success rate for implantation and good short- to mid-term safety. For patients with HF combined with LBBB, LBBP can significantly improve cardiac function, with lower and more stable thresholds for correcting LBBB. However, there are currently no large-scale, multicenter, randomized, controlled studies comparing the efficacy differences between LBBP and conventional BVP in HF patients with LBBB.

3. Aim

This a multicenter, prospective, randomized, controlled trial which enrolling patients with HF, left ventricular ejection fraction (LVEF) ≤ 35%, and LBBB. Patients will be randomly assigned in a 1:1 ratio to receive either LBBP or BVP. The clinical endpoints (including primary and secondary endpoints) will be compared between the

two groups to evaluate the effectiveness and safety, in order to provide new treatment methods for such patients and improve their prognosis.

4. Study design

4.1 Overall design

This a multicenter, prospective, randomized, controlled trial. HF patients with reduced LVEF and LBBB will be screened, and a total of 200 patients will be finally enrolled. Enrolled patients will be then randomly assigned to receive either LBBP or BVP in a 1:1 ratio. Follow-up visits will be conducted at 1 month, 3 months, 6 months and every 6 months thereafter after surgery to evaluate the clinical endpoints of the two groups.

4.2 Masking

The research assistants who collect study outcome data will be masked to the participants' intervention assignment. All the efficacy and safety end points will be independently adjudicated by a committee whose members are unaware of the trial-group assignments and the identity of the patients.

4.3 Study flowchart

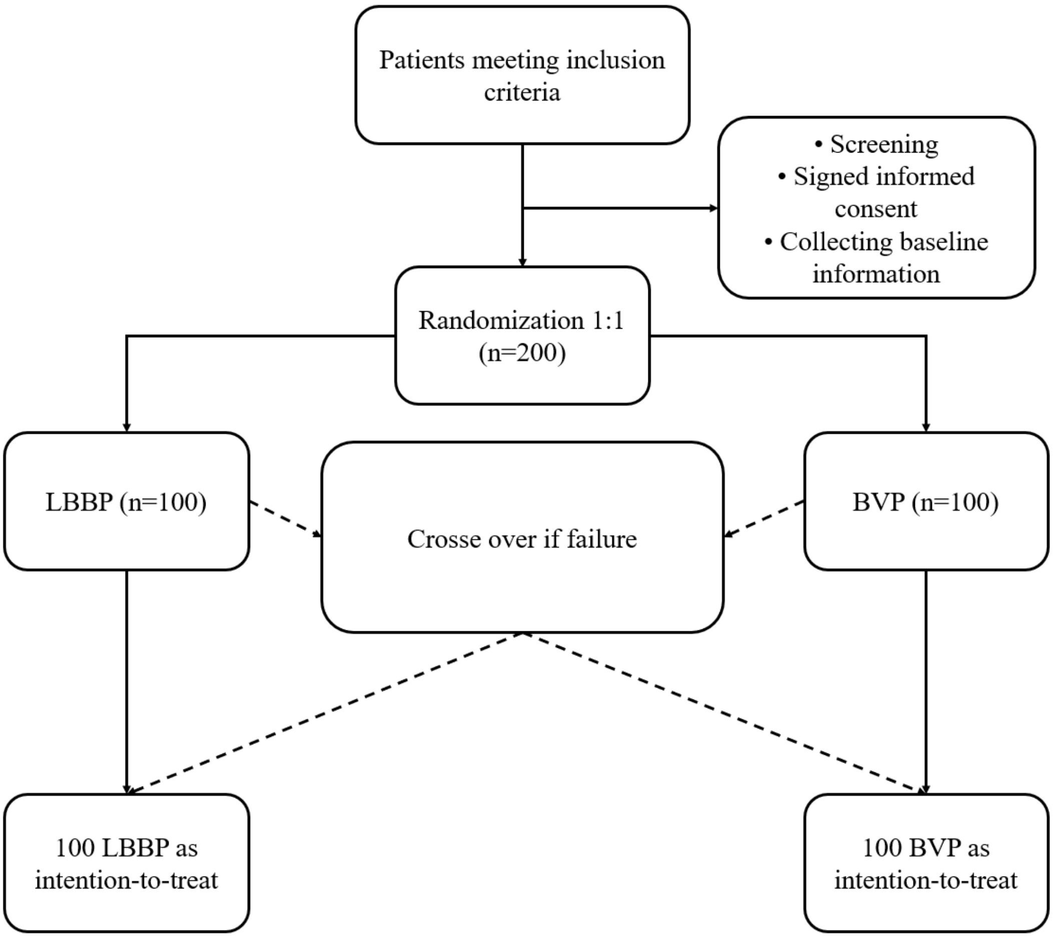

5. Study participants

5.1 Inclusion criteria

(1) Age 18 to 80 years; (2) sinus rhythm, complete LBBB; (3) LVEF ≤ 35% with New York Heart Association (NYHA) functional class II to IV despite receiving at least 3 months' guideline directed medical therapy; (4) expected survival time > 1 year; (5) the residence is stable and can meet the requirements of follow-up during the study period.

5.2 Exclusion criteria

(1) Decline to participate or refuse to sign the written informed consent; (2) participate in other clinical studies; (3) persistent or permanent atrial fibrillation; (4) heart transplant patients or patients waiting for heart transplantation; (5) patients with previous pacemaker implantation; (6) patients with mechanical tricuspid valve implantation; (7) severe renal insufficiency; (8) severe liver dysfunction; (9) pregnancy.

5.3 Termination criteria

(1) Subjects ask to withdraw the informed consent form; (2) The investigator determines that the subject is not suitable for further study.

5.4 Enrolling center

All patients will be enrolled from 6 centers in China: (1) Zhongshan Hospital of Fudan University; (2) Shanghai Chest Hospital, Shanghai Jiao Tong University; (3) Shanghai Changhai Hospital, Naval Medical University; (4) Shanghai Changzheng Hospital, Second Affiliated Hospital of Naval Medical University; (5) Renji Hospital, Shanghai Jiao Tong University School of Medicine; (6) Shanghai Jiao Tong University Affiliated Sixth People's Hospital.

6. Interventional method

6.1 Procedural of LBBP

LBBP will be performed as following steps: (1) the His bundle (HB) potential will be first mapped with the pacing lead through the sheath; (2) then the lead is moved from the HB potential location towards the right ventricular apex for approximate 1 to 2 cm under the right anterior oblique (RAO) 30° fluoroscopic view and is deeply screwed into the interventricular septum (IVS); (3) LBBP is considered to be successful if the

unipolar paced QRS morphology demonstrate a right bundle branch block (RBBB) pattern and meet any of the following criteria: 1) Paced morphology changes between selective LBBP and nonselective LBBP with constant left ventricular activation time (LVAT); 2) Paced morphology changes from left ventricular septal pacing (LVSP) to nonselective LBBP with LVAT abruptly shortening by increasing pacing output. Crossover will be allowed when LBB capture could not be achieved.

6.2 Procedural of BVP

BVP will be performed with a standard fashion and lateral or posterolateral branches are preferred choices for LV lead implantation, quadripolar LV lead is also used whenever feasible. Crossover will be allowed when the LV lead implant unsuccessfully due to anatomical variations, high pacing threshold, or phrenic nerve stimulus.

6.3 Device programming

In LBBP group, atrioventricular delay will be customized according to electrocardiogram to fuse with the intrinsic right bundle branch (RBB) conduction to achieve narrowest QRS duration (QRSd). In BiVP group, the optimization of AV and VV delay in each patient will be performed based on the paced QRSd.

7. Data collection and follow-up

Baseline data including age, gender, etiology (nonischemic cardiomyopathy or not), comorbidities (hypertension, diabetes mellitus and etc.), NYHA functional class and current medications (angiotensin-converting enzyme inhibitors/ angiotensin II receptor blocker/ angiotensin receptor-neprilysin inhibitor, beta-blockers, spironolactone) will be collected at enrollment.

Type of device (cardiac resynchronization therapy with defibrillator or with pacemaker) and electrocardiographic measurements (intrinsic QRSd and paced QRSd) will be collected at implantation, and the intrinsic QRSd and paced QRSd are measured at speed of 100 mm/s from the onset to the end of QRS complex.

All enrolled patients will be routinely followed up at 1-, 3-, and 6-month post procedure and every 6 months thereafter in the device clinic. Pacing threshold is collected at implantation and follow-up. Echocardiographic measurements including LVEF, left ventricular end-diastolic diameter (LVEDD) and left ventricular end-systolic diameter (LVESD) will be collected at implantation and follow-up.

8. Safety assessment

Procedure related complications including pneumothorax, pocket infection, lead dislodgement requiring revision, increased pacing threshold > 3 V/0.5ms and pericardial tamponade will be documented as safety assessment during follow-up.

9. Study outcomes

9.1 Primary endpoint

The composite of all-cause mortality and HF hospitalization (HFH). HFH is defined as any urgent visit with HF signs or symptoms requiring intravenous diuretic therapy.

9.2 Secondary endpoints

Secondary endpoints including: (1) Individual outcomes of all-cause mortality; (2) individual outcomes of HFH; (3) Echocardiographic response/super-response rates at

6-month follow-up. Echocardiographic response is defined as an absolute improvement in LVEF ≥ 5%. Super-response is defined as an absolute improvement in LVEF ≥ 15% or improvement of LVEF to ≥ 50%.

10. Data management

10.1 Case report form

All data including screening assessments, examinations, therapeutic effects and safety assessments, will be documented in the participant's case report form (CRF). All CRFs for each patient should be collected and documented by study group member in a timely manner. Each CRF should be double-checked for missing data or potential errors, and will be stored in the study office.

10.2 Data entry

All data will be double-entered by two study group members, and will be collected at two databases for consistency. Whenever inconsistencies are found, the data will be rechecked by re-examination according to original CRFs or medical history.

11. Statistical consideration

11.1 Sample size calculating

There are no studies evaluating the long-term prognosis of LBBP, and results for the long-term prognosis of BVP vary among studies. Therefore, the data for the sample size estimation of this study are mainly derived from our preliminary clinical experience. A sample size of 190 patients is calculated to detect a statistically

significant difference in the primary endpoint with 80% power and two-tailed alpha level of 5%, assuming 10% event rate in the LBBP and 25% in the BVP arm with 10% dropout and 5% crossover. To ensure sufficient statistical efficiency, the sample size is finally determined to be 100 for each group.

11.2 Statistical analysis

All analyses in this study will be conducted according to the intention-to-treat principle. Continuous variables are described as mean ± SD and categorical variables are described as frequencies or percentages. Student's t-test is performed for normally distributed continuous variables while Wilcoxon signed rank test is for non-normally distributed data. The chi-square test or Fisher exact test is used for categorical variables. For the clinical endpoint, Kaplan-Meier (KM) survival curve and log-rank test are performed to compare the time-to-event between LBBP and BVP. Subgroup analyses of the relationship between the primary endpoint and the baseline characteristics including age, sex, etiology, comorbidities, QRS duration, NYHA class, and left ventricular ejection fraction will be conducted. A two-sided P-value < 0.05 is considered statistically significant. All statistical analyses are performed using SPSS Statistics version 22.0 (IBM Corporation, Armonk, NY).

12. Enrolling center

Zhongshan Hospital of Fudan University	Primary center
Shanghai Changhai Hospital	Participating center
Shanghai Changzheng Hospital	Participating center
Shanghai Chest Hospital	Participating center
Shanghai Jiao Tong University Affiliated Sixth People's Hospital	Participating center
Renji Hospital	Participating center

13. Bias control

As this study is a prospective, randomized, controlled study, the randomization process should be strictly enforced to ensure the accuracy of the grouping. Follow-up members should avoid knowing the grouping results of each patient at the time of follow-up to prevent bias in the results.

14. Ethics approval

14.1 Review of the ethics committee

This study complies with the Declaration of Helsinki (revised in 2013) and the relevant regulations of Chinese clinical trials. The protocol and written informed consent must be submitted to the ethics committee, and written approval from the ethics committee must be obtained before the study can be formally conducted. The investigator must submit an annual report of the study to the ethics committee at least annually. The investigator must notify the ethics committee in writing when the study is discontinued and/or completed; the investigator must report to the ethics committee in a timely manner all changes occurring in the conduct of the study (e.g., revisions to the protocol and/or informed consent form) and must not implement these changes without first obtaining approval from the ethics committee, except for changes made to eliminate obvious and immediate risks to subjects. The ethics committee will be informed when this occurs.

14.2 Informed consent

The investigator must provide the subject or his/her legal representative with a readily understandable and ethics committee - approved informed consent form and allow

sufficient time for the subject or his/her legal representative to consider the study, and the subject will not be enrolled in the study until signed written informed consent has been obtained from the subject. Subjects will be provided with all updated versions of the informed consent form, along with written information, for the duration of the subject's participation. The informed consent form should be retained for review as an important document of the clinical trial.

15. Confidentiality measures

The results of the study may be published in medical journals, but we will maintain the confidentiality of the patients' information in accordance with the requirements of the law, and the personal information of the patients will not be disclosed unless it is required by the relevant laws. When necessary, the government administration and the hospital ethics committee and its related personnel can access the patients' information according to the regulations.

16. Proposed study timelines

This study is estimated to take about 4 years including patients’ recruitment for 1.5 years. This study is expected to conducted between October 2020 and September 2024.

17. Reference

[1] Tops LF, Schalij MJ, Holman ER, van Erven L, van der Wall EE, Bax JJ. Right ventricular pacing can induce ventricular dyssynchrony in patients with atrial

fibrillation after atrioventricular node ablation. J Am Coll Cardiol. 2006;48:1642–1648. doi: 10.1016/j.jacc.2006.05.072.
[2] Sweeney MO, Hellkamp AS, Ellenbogen KA, Greenspon AJ, Freedman RA, Lee KL, Lamas GA; MOde Selection Trial Investigators. Adverse effect of ventricular pacing on heart failure and atrial fibrillation among patients with normal baseline QRS duration in a clinical trial of pacemaker therapy for sinus node dysfunction. Circulation. 2003;107:2932–2937. doi: 10.1161/01.CIR.0000072769.17295.B1.
[3] Wilkoff BL, Cook JR, Epstein AE, Greene HL, Hallstrom AP, Hsia H, Kutalek SP, Sharma A. Dual-chamber pacing or ventricular backup pacing in patients with an implantable defibrillator: the Dual Chamber and VVI Implantable Defibrillator (DAVID) trial. JAMA. 2002;288:3115–3123.
[4] Shaan Khurshid BA, Andrew E. Epstein MD, FHRS, Ralph J. Verdino MD, David Lin MD, FHRS, Lee R. Goldberg MD, Francis E. Marchlinski MD, FHRS, David S. Frankel MD, FHRS, Incidence and Predictors of Right Ventricular Pacing-Induced Cardiomyopathy, Heart Rhythm, http://dx.doi.org/10.1016/j. hrthm.2014.05.040
[5] Kiehl EL, Makki T， Kumar R, Gumber D, Kwon DH, Rickard JW, Kanj M, Wazni OM, Saliba WI, Varma N, Wilkoff BL, Cantillon DJ. Incidence and predictors of right ventricular pacing-induced cardiomyopathy in patients with complete atrioventricular block and preserved left ventricular systolic function. Heart Rhythm. 2016 Dec;13(12):2272-2278. doi: 10.1016/j.hrthm.2016.09.027.
[6] Cho SW, Gwag HB, Hwang JK, Chun KJ, Park KM, On YK, Kim JS, Park SJ.Clinical features, predictors, and long-term prognosis of pacing-induced cardiomyopathy. Eur J Heart Fail. 2019;21(5):643-651.
[7] Incidence and predictors of right ventricular pacing-induced cardiomyopathy.
Khurshid S, Epstein AE, Verdino RJ, Lin D, Goldberg LR, Marchlinski FE, Frankel DS.Heart Rhythm. 2014;11(9):1619-25.
[8] Curtis AB, Worley SJ, Adamson PB, Chung ES, Niazi I, Sherfesee L, Shinn T, Sutton MS;Biventricular versus Right Ventricular Pacing in Heart Failure Patients with Atrioventricular Block (BLOCK HF) Trial Investigators. Biventricular pacing for

atrioventricular block and systolic dysfunction.
[9] Piotr Ponikowski, Adriaan A Voors, Stefan D Anker, Héctor Bueno, John G F Cleland, Andrew J S Coats, Volkmar Falk, José Ramón González-Juanatey, Veli-Pekka Harjola, Ewa A Jankowska, Mariell Jessup, Cecilia Linde, Petros Nihoyannopoulos, John T Parissis, Burkert Pieske, Jillian P Riley, Giuseppe M C Rosano, Luis M Ruilope, Frank Ruschitzka, Frans H Rutten, Peter van der Meer, ESC Scientific Document Group. 2016 ESC Guidelines for the Diagnosis and Treatment of Acute and Chronic Heart Failure: The Task Force for the Diagnosis and Treatment of Acute and Chronic Heart Failure of the European Society of Cardiology (ESC)Developed With the Special Contribution of the Heart Failure Association (HFA) of the ESC. Eur Heart J, 37 (27), 2129-2200.
[10] Funck RC, Mueller HH, Lunati M, Piorkowski C, De Roy L, Paul V, Wittenberg M, Wuensch D, Blanc JJ; BioPace study group. Characteristics of a large sample of candidates for permanent ventricular pacing included in the Biventricular Pacing for Atrio-ventricular Block to Prevent Cardiac Desynchronization Study (BioPace). Europace. 2014 Mar;16(3):354-62. doi: 10.1093/europace/eut343.
[11] Fred M Kusumoto, Mark H Schoenfeld, Coletta Barrett, James R Edgerton, Kenneth A Ellenbogen, Michael R Gold, Nora F Goldschlager, Robert M Hamilton, José A Joglar, Robert J Kim, Richard Lee, Joseph E Marine, Christopher J McLeod, Keith R Oken, Kristen K Patton, Cara N Pellegrini, Kimberly A Selzman, Annemarie Thompson, Paul D Varosy. 2018 ACC/AHA/HRS Guideline on the Evaluation and Management of Patients with Bradycardia and Cardiac Conduction Delay: A Report of the American College of Cardiology/American Heart Association Task Force on Clinical Practice Guidelines and the Heart Rhythm Society. Circulation, 140 (8), e382-e482.
[12] Occhetta E, Bortnik M, Magnani A, et al. Prevention of ventricular desynchronization by permanent para-Hisian pacing after atrioventricular node ablation in chronic atrial fibrillation: a crossover, blinded, randomized study versus apical right ventricular pacing. J Am Coll Cardiol. 2006;47:1938-45.

[13] Zanon F, Bacchiega E, Rampin L, et al. Direct His bundle pacing preserves coronary perfusion compared with right ventricular apical pacing: a prospective, cross-over mid-term study. Europace. 2008;10:580-7

[14] Kronborg MB, Mortensen PT, Poulsen SH, et al. His or para-His pacing preserves left ventricular function in atrioventricular block: a double-blind, randomized, crossover study. Europace. 2014;16:1189-96

[15] Abdelrahman M, Subzposh FA, Beer D, Durr B, Naperkowski A, Sun H, Oren JW, Dandamudi G, Vijayaraman P, Clinical Outcomes of His Bundle Pacing Compared to Right Ventricular Pacing, Journal of the American College of Cardiology (2018), doi: 10.1016/ j.jacc.2018.02.048.

[16] Vijayaraman P, Bordachar P, Ellenbogen KA. The continued search for physiological pacing: where are we now? J Am Coll Cardiol. 2017;69(25):3099-3114.

[17] Huang W, Su L, Wu S, Xu L, Xiao F, Zhou X, Ellenbogen KA. A novel pacing strategy with low and stable output: pacing the left bundle branch immediately beyond the conduction block. Can J Cardiol. 2017;33(12):1731-1736.

[18] Vijayaraman P, Subzposh FA, Naperkowski A, Panikkath R, John K, Mascarenhas V, et al. Prospective evaluation of feasibility, electrophysiologic and echocardiographic characteristics of left bundle branch area pacing. Heart Rhythm. 2019.

[19] Hou X, Qian Z, Wang Y et al. Feasibility and cardiac synchrony of permanent left bundle branch pacing through the interventricular septum. Europace, 2019,doi:10.1093/europace/euz188.

Statistical Analysis Plan

Title：Comparison of left bundle branch pacing and biventricular pacing in the treatment of chronic systolic heart failure with left bundle branch block: a multicenter, prospective, randomized, controlled trial

Primary investigator:

Yangang Su, MD

Department of Cardiology, Zhongshan Hospital of Fudan University, Shanghai Institute of Cardiovascular Diseases, National Clinical Research Center for Interventional Medicine, Shanghai, China

1. Introduction

This Statistical Analysis Plan (SAP) describes the statistical analyses to be performed for the data from the protocol of a clinical trial entitled “Comparison of left bundle branch pacing and biventricular pacing in the treatment of chronic systolic heart failure with left bundle branch block: a multicenter, prospective, randomized, controlled trial” sponsored by Department of Cardiology, Zhongshan Hospital of Fudan University, Shanghai Institute of Cardiovascular Diseases, National Clinical Research Center for Interventional Medicine, Shanghai, China. This SAP is written based on the Protocol Version 1.0 (Date: 09 Aug 2020).

2. Study Objectives

This study aims to evaluate the clinical outcomes of left bundle branch pacing (LBBP) versus biventricular pacing (BVP).

3. Study Design

This a multicenter, prospective, randomized, controlled trial which enrolling patients with HF, left ventricular ejection fraction (LVEF) ≤ 35%, and left bundle branch block (LBBB). Patients will be randomly assigned in a 1:1 ratio to receive either LBBP or BVP. Follow-up visits will be conducted at 1 month, 3 months, 6 months and every 6 months thereafter after surgery to evaluate the clinical endpoints (including primary and secondary endpoints) of the two groups.

4. Study Arms

All enrolled patients will receive either LBBP or BVP.

5. Randomization and Masking

This study is a prospective, multicenter, randomized, single-blind trial. All enrolled patients will be randomly assigned in a 1:1 ratio to receive either LBBP or BVP. The research assistants who collect study outcome data will be masked to the participants' intervention assignment. All the efficacy and safety end points will be independently adjudicated by a committee whose members are unaware of the trial-group assignments and the identity of the patients.

6. Study Endpoints

5.1 Primary endpoint

The primary endpoint is the composite of all-cause mortality and heart failure hospitalization (HFH). HFH is defined as any urgent visit with heart failure (HF) signs or symptoms requiring intravenous diuretic therapy.

5.2 Secondary endpoints

The Secondary endpoints including: (1) Individual outcomes of all-cause mortality; (2) individual outcomes of HFH; (3) Echocardiographic response/super-response rates at 6-month follow-up. Echocardiographic response is defined as an absolute improvement in left ventricular ejection fraction (LVEF) ⩾ 5%. Super-response is

defined as an absolute improvement in LVEF ⩾ 15% or improvement of LVEF to ⩾ 50%.

7. Data management

7.1 Case report form

All data including screening assessments, examinations, therapeutic effects and safety assessments, will be documented in the participant's case report form (CRF). All CRFs for each patient should be collected and documented by study group members in a timely manner. Each CRF should be double-checked for missing data or potential errors, and will be stored in the study office.

7.2 Data entry

All data will be double-entered by two study group members, and will be collected at two databases for consistency. Whenever inconsistencies are found, the data will be rechecked by re-examination according to original CRFs or medical history.

8. Statistical Consideration

8.1 Sample size calculating

There are no studies evaluating the long-term prognosis of LBBP, and results for the long-term prognosis of BVP vary among studies. Therefore, the data for the sample size estimation of this study are mainly derived from our preliminary clinical experience. A sample size of 190 patients is calculated to detect a statistically significant difference in the primary endpoint with 80% power and two-tailed alpha

level of 5%, assuming 10% event rate in the LBBP and 25% in the BVP arm with 10% dropout and 5% crossover. To ensure sufficient statistical efficiency, the sample size is finally determined to be 100 for each group.

8.2 Results description

Continuous variables are described as mean ± SD and categorical variables are described as frequencies or percentages.

8.3 Statistical analysis

All analyses in this study will be conducted according to the intention-to-treat principle. Student's t-test is performed for normally distributed continuous variables while Wilcoxon signed rank test is for non-normally distributed data. The chi-square test or Fisher exact test is used for categorical variables. A two-sided P-value < 0.05 is considered statistically significant.

8.4 Statistical analysis of the endpoints

For the clinical endpoint, Kaplan-Meier (KM) survival curve and log-rank test are performed to compare the time-to-event between LBBP and BVP.

8.5 Subgroup analysis

Subgroup analyses of the relationship between the primary endpoint and the baseline characteristics including age, sex, etiology, comorbidities, QRS duration, New York Heart Association (NYHA) class, and LVEF will be conducted.
