# Supplementary material for: Long-Term Outcomes of Left Bundle-Branch Pacing vs Biventricular Pacing in Heart Failure: The HeartSync-LBBP Randomized Clinical Trial
Source: JAMA Cardiol. 2026 Mar 11;11(4):352–9. doi: 10.1001/jamacardio.2026.0083 (PMC12980356; doi:10.1001/jamacardio.2026.0083)
Supplement: Supplement 3. — Data sharing statement [file jamacardiol-e260083-s003.pdf]

# Data Sharing Statement

Chen. Long-Term Outcomes of Left Bundle-Branch Pacing vs Biventricular Pacing in Heart Failure. *JAMA Cardiol*. Published March 11, 2026. doi:10.1001/jamacardio.2026.0083

**Data**
**Additional Information:** This study was registered at Chinese Clinical Trial Registry (URL: https://www.chictr.org.cn/showproj.html?proj=59660; Registration number: ChiCTR2000036554).
**Data available:** No

**Additional Information**
**Explanation for why data not available:** All data are available from the corresponding author on reasonable request.
